# Proceedings of Reanimation 2021, the French Intensive Care Society International Congress

**DOI:** 10.1186/s13613-021-00862-0

**Published:** 2021-06-29

## Acknowledgements

### I-1 This abstract report was edited and corrected by the members of the Congress Committee of the French Intensive Care Society.

#### Hafid Ait-Oufella^1^, Pierre Asfar^2^, Cécile Aubron^3^, Emmanuel Canet^4^, Guillaume Carteaux^5^, Alexandre Demoule^6^, Stephan Ehrmann^7^, Jean Pierre Frat^8^, Guillaume Geri^9^, Julie Helms^10^, Saad Nseir^11^, Meehdi Oualha^12^, Frédéric Pène^13^, Nicolas Weiss^6^

##### ^1^Hôpital Saint-Antoine, Assistance-Publique des Hôpitaux de Paris (AP-HP), Paris, France; ^2^Centre Hospitalier Universitaire (CHU) d’Angers, Angers, France ; ^3^Hôpital de la Cavale Blanche, CHU et Université de Bretagne Occidentale, Brest, France; ^4^CHU de Nantes, Nantes, France; ^5^Hôpital Henri Mondor, AP-HP, Créteil, France ; ^6^Groupe Hospitalier Pitié-Salpêtrière Charles Foix, AP-HP, Paris, France; ^7^CHU de Tours, Tours, France; ^8^CHU de Poitiers, Poitiers, France ; ^9^Hôpital Ambroise Paré, AP-HP, Boulogne-Billancourt, France ; ^10^CHU de Strasbourg, Strasbourg,France; ^11^CHRU de Lille, Lille, France; ^12^Hôpital Necker-Enfants Malades, AP-HP, Paris, France ; ^13^Hôpital Cochin, AP-HP, Paris, France.

## Oral communications

### CO-001 Effects of prone position in acute respiratory distress syndrome in children: retrospective analysis of a 16-year cohort

#### Sophie Beldjilali, Fabrice Michel

##### APHM, Marseille, France

**Correspondence:** Sophie Beldjilali - sophie.beldjilali@ap-hm.fr

*Annals of Intensive Care* 2021, **11(Suppl 1):**CO-001

**Rationale:** Acute Respiratory Distress Syndrome (ARDS) in children is a rare and serious pathology. The management is based on results of adult studies. Prone positioning (PP) is one of the main axes of the management in the acute phase, but its effectiveness on gas exchanges and its impact on outcome remain poorly documented. The main objective of this work was to evaluate the effectiveness of PP in pediatric ARDS. The second objective was to determine the response criteria on oxygenation.

**Patients and methods/materials and methods:** We led a retrospective bicentric study in two pediatric intensive care unit (PICU). Inclusion criteria were children between 28 days and 18 years of age hospitalized in PICU and having presented moderate or severe ARDS defined as Index Oxygenation (IO) ≥ 8 and/or PaO_2_/FiO_2_ < 200. Period of inclusion was 2003 to 2018. We collected demographic, etiological, biological and therapeutic parameters, including PP positioning and the duration of each PP periods.

**Results:** Out of 167 patients, 54 (32%) were positioned in PP during the study. Sixty-nine percent of them were qualified as responders on the improvement of oxygenation (defined as an increase of 20% or more in PaO_2_/FiO_2_ ratio between the first or second period of PP). There was no significant difference in mortality between patients positioned in PP and others. Two factors were significantly associated with a better response to PP: a heavier weight with a cut-off at 4.5 kg (OR 1.1; *p* = 0.037; IC95% [1.006; 1.195]) and the mean duration of the first two sessions of PP with a cut-off at 11.5 h (OR 1.6; *p* = 0.003; IC95% [1.159; 2.09]).

**Conclusion:** This is the largest French retrospective cohort about PP during pediatric ARDS. PP improved oxygenation in 69% of cases. The mechanisms of improvement by PP and the factors associated with a better response have yet to be evaluated in a prospective study.

**Compliance with ethics regulations:** Yes in clinical research.

### CO-002 Implementation and evaluation of a nurse-driven noninvasive ventilation weaning protocol in infants with severe bronchiolitis: the SEVENT study

#### Julie Cassibba, Marie Chevalier, Isabelle Pin, Brigitte Fauroux, Guillaume Mortamet

##### CHU Grenoble Alpes (CHUGA), Grenoble, France

**Correspondence:** Julie CASSIBBA - jcassibba@chu-grenoble.fr

*Annals of Intensive Care* 2021, **11(Suppl 1):**CO-002

**Rationale:** Noninvasive ventilation (NIV) is the first-line therapy in infants with bronchiolitis-related acute respiratory failure. However, there is a lack of data regarding weaning from NIV in this setting. This study aims to evaluate a nurse-driven weaning protocol in this homogenous population.

**Patients and methods/materials and methods:** A retrospective single-center study with pre- versus post-comparative design in a tertiary center. Data from all infants aged ≤ 6 months admitted to the PICU during 2 seasons with a clinical diagnosis of bronchiolitis and requiring any type of noninvasive ventilatory support on admission were analyzed. We compared the outcomes of infants treated with standard and nurse-driven protocols (Fig. 1).

**Results:** A total of 187 infants (95 with standard and 92 with nurse-driven protocols) were included; the median age was 47 days (IQR 24–75) at baseline and 31 days (19–58) in patients admitted after implementation of the protocol. There was no difference in terms of weaning failure between the two periods (11 (12%) versus 14 (15%), *p* = 0.46). At baseline, the ventilatory support duration was 70 h (IQR 54–104) versus 56 h (IQR 29–83) during the nurse-driven protocol period (*p* = 0.29). PICU and hospital lengths of stay did not differ between the two periods. No complication related to NIV occurred in the two periods.

**Conclusion:** In patients with bronchiolitis supported by NIV, the nurse-driven weaning management as opposed to physician-driven was not associated with a significantly higher proportion of weaning failure cases.

**Compliance with ethics regulations:** Yes in clinical research. 
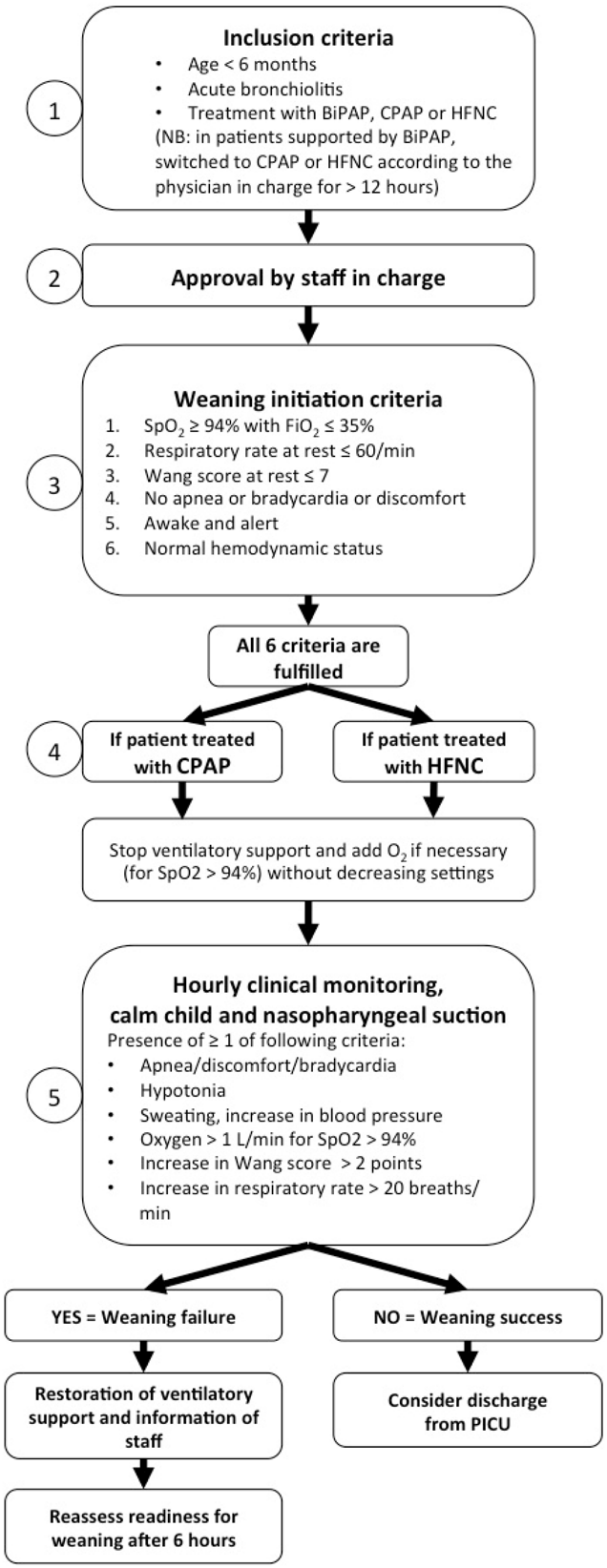


Nurse-driven weaning protocol. CPAP: Continuous positive airway ventilation; HFNC: High-flow nasal cannula; PICU: Pediatric intensive care unit

### CO-003 Proton pump inhibitors prescription patterns and associated complications in the pediatric intensive care unit—a propensity score matching analysis

#### Isabelle Goyer, Julien Montreuil, Edouard Lacotte, Pascal Thibon, Anais Briant, Claire Dupont, David Brossier

##### CHU de Caen, Caen, France

**Correspondence:** Isabelle Goyer - goyer-i@chu-caen.fr

*Annals of Intensive Care* 2021, **11(Suppl 1):**CO-003

**Rationale:** Proton pump inhibitors (PPIs) are regularly prescribed in the Pediatric Intensive Care Unit (PICU) for prevention of stress ulcer and upper gastro-intestinal bleeding (UGIB). There is no clinical consensus regarding PPI use in the PICU. The reported incidence of UGIB in the PICU is low (0.4–5%). Having ≥ 2 risk factors has been associated with a higher risk of clinically relevant UGIB in the PICU (severity score at admission PRISM > 10, coagulopathy, mechanical ventilation). Despite identification of these risk factors, studies failed to show that stress ulcer prophylaxis (SUP) significantly decreases UGIB in the PICU. Moreover, recent studies, mostly including adult patients, have associated PPIs in the acute setting to a higher risk of nosocomial infections and hyponatremia.

**Patients and methods/materials and methods:** The aim of this study was to describe PPI prescription patterns in the PICU and explore the potentially associated complications (nosocomial infections and hyponatremia). This was a single-centre retrospective cohort study from January 1st, 2017 to December 31st, 2018.

**Results:** 768 patients were included of which 234 received a PPI (30.6%). PPI-exposed patients were younger (*p* < 0.05), with a lower weight (*p* < 0.05) and were more likely to have had surgery (*p* < 0.05), a central venous access (*p* < 0.05), parenteral nutrition (*p* < 0.05), coagulopathy (*p* < 0.05), mechanical ventilation (*p* < 0.05) and had a longer PICU-stay (*p* < 0.05). The most common indication for PPI was stress ulcer prophylaxis (*n* = 178, 76.1%), but only 12.4% (*n* = 22) had ≥ 2 UGIB risk factors. Nosocomial infection rate was 9.4% in the PPI group vs 2.2% in the non-exposed group (RR = 3.40 [IC95% 1.76–6.57], *p* < 0.05). Once adjusted for confounding variables, PPI exposure was independently associated with a higher risk for nosocomial infection (ORa = 2.42 [IC95% 1.17–5.14], *p* = 0.02). Propensity score matching analysis retrieved a significantly higher risk for nosocomial infection in the PPI-exposed group (*p* = 0.031). PPI exposure was associated with an increased risk of hyponatremia (RR = 5.18 [IC95% 2.16–12.43], *p* < 0.05).

**Conclusion:** Our study shows an overuse of PPIs with poorly documented indications. PPIs were statistically and independently associated with an increased risk for nosocomial infections in our population. Prospective randomized trials are needed to evaluate the risk–benefit ratio of PPIs in the PICU. Our results suggest the need for a more rational use of PPIs in the PICU and highlight the lack of clinical guidelines and safety data regarding SUP in critically ill children.

**Compliance with ethics regulations:** Yes in clinical research.

### CO-004 Pain management via music therapy during cleaning cares in pediatric intensive care unit

#### Sophie Mounier, Christophe Milesi, Julien Baleine, Manon Le Roux, Christiane Prad, Severine Assie, Gilles Cambonie

##### CHU Arnaud de Villeneuve, Montpellier, France

**Correspondence:** Christophe Milesi - c-milesi@chu-montpellier.fr.

*Annals of Intensive Care* 2021, **11(Suppl 1):**CO-004

**Rationale:** Pain and discomfort are frequent in critically ill children hospitalized in pediatric intensive care unit. Music therapy is a non-pharmacological intervention that can alleviate discomfort. The aim of this study was to evaluate the effectiveness of passive music therapy intervention to reduce discomfort during cleaning cares in critically ill children.

**Patients and methods/materials and methods:** We conducted a prospective crossover clinical study with random ordering of the intervention and blind assessment of the primary outcome. We included children between 6 months old and 15 years old, admitted in the Pediatric Intensive Care Unit of University Hospital in Montpellier, France. We used a specific music therapy program, “Music Care©”. The primary outcome was the difference between the Face Legs Activity Cry Consolability scale’s score before and during the cleaning cares with and without music therapy. The secondary outcomes were the variations of physiological parameters such as heart rate, mean blood pressure, respiratory rate, and the use of pharmacological drugs.

**Results:** 50 children were consecutively included from May 2019 to May 2020. The pain score variation before and during cleaning cares was lower with music therapy (1.58 (1.55) point versus 2.14 (1.87) points (*p* = 0.02)). The comfort FLACC score during cleaning was lower with music therapy (2.21 (1.92) vs 3.46 (2.68); *p* < 0.001). The rise of heart rate was attenuated with music therapy (8 (14) vs 17 (13), *p* = 0.002).

**Conclusion:** This study shows the efficacy of music therapy in improving comfort during children’s cleaning cares in the Pediatric Intensive Care Unit.

**Compliance with ethics regulations:** Yes in clinical research.

### CO-005 Neurological outcome and health-related quality of life in children undergoing extracorporeal membrane oxygenation

#### Judith Chareyre, Alizée Michel, Michael Thy, Meryl Vedrenne, Raphael Levy, Marion Grimaud, Florence Moulin, Manoëlle Kossorotoff, Anna Kaminska, Isabelle Desguerre, Sylvain Renolleau, Mehdi Oualha

##### APHP, Necker- Enfants Malades, Paris, France

**Correspondence:** Judith Chareyre - judith.chareyre@aphp.fr

*Annals of Intensive Care* 2021, **11(Suppl 1):**CO-005

**Rationale:** Extracorporeal membrane oxygenation (ECMO) is a supportive technique required in patients suffering from refractory respiratory and/or circulatory failure despite adequate conventional treatments. Its increasing use in pediatric intensive care is associated with several brain injuries. The possible consequences on neurological development and health-related quality of life (HRQoL) are poorly described. Our main aim was to describe short and long-term neurological outcome of surviving patients and their HRQoL. Our secondary objectives were to identify factors associated with a poor outcome.

**Patients and methods/materials and methods:** Forty surviving patients aged less than 18 years receiving peripheral veno-venous (VV) or veno-arterial (VA) ECMO from October 2014 to December 2019 were included. First, we collected retrospectively demographic and ECMO data for all patients. We defined “poor short-term outcome” by a pathological neurological clinical examination at PICU discharge. Secondarily, we conducted interviews in March 2020 to assess HRQoL (using PedsQL questionary). During these interviews, two points of psychomotor development were assessed using Denver II test (DT II), with parents and health records, for children under 3 years old: retrospectively at 1 year after ECMO withdrawal and in March 2020. Neurological outcome of children older than 3 years was evaluated with Pediatric Overall/Cerebral Performance Category scores (POPC/PCPC).

**Results:** Median age at ECMO’s initiation was 1.4 years [0.4–6]. There were 35 (88%) VA-ECMO. With respect to short-term outcome, 17/40 patients (43%) had an abnormal neurological examination at PICU discharge. Half of these patients (47%) presented hemiparesis. Carotid cannulation was identified as a main risk factor of poor short-term outcome (OR = 9.7 [1–234] *p* = 0.04). With respect to HRQoL and long-term outcome, PedsQL scores were superior to 70/100 for all patients. Older patients presented the highest scores. Emotional, social and cognitive dimensions were mostly affected. PedsQL scores were similar to those published in patients suffering from chronic diseases. The DTII four domains evaluations were normal for 50%(*n* = 15) children 1 year after ECMO withdrawal, and for more than 70% in March 2020, except for the language, which was still impaired for 40% of them. POPC and PCPC scores were inferior to 3 in more than 80% (*n* = 25) of children. The patients without neurological impairment at PICU discharge presented better PCPC scores in March 2020.

**Conclusion:** Our study showed comforting results in terms of HRQoL and neurological development. Cognitive difficulties remain a real concern. Larger studies could help to define a proper follow-up of this high-risk population in order to provide appropriate rehabilitation.

**Compliance with ethics regulations:** Yes in clinical research.

### CO-006 Low-dosing norepinephrine effects on cerebral oxygenation and perfusion during pediatric shock

#### Meryl Vedrenne-Cloquet^1^, Judith Chareyre^1^, Pierre-Louis Léger^3^, Mathieu Genuini^2^, Sylvain Renolleau^1^, Mehdi Oualha^1^

##### ^1^CHU Necker-Enfants Malades, Paris, France; ^2^CHU Robert Debré, Paris, France; ^3^CHU Armand Trousseau-La Roche Guyon, Paris, France

**Correspondence:** Meryl Vedrenne-Cloquet - meryl.vedrenne-ext@aphp.fr

*Annals of Intensive Care* 2021, **11(Suppl 1):**CO-006

**Rationale:** Sepsis may alter cerebral hemodynamics leading to septic-associated encephalopathy (1). Norepinephrine is used to restore systemic and regional circulation, but its cardiovascular and cerebral effects may be unpredictable. While increasing mean arterial pressure (MAP), norepinephrine restores cerebral blood flow without improving cerebral oxygenation in adults (2). To date, cerebral effects of norepinephrine are unknown during pediatric shock. We aim to assess cerebral oxygenation and perfusion effects of norepinephrine in children with shock.

**Patients and methods/materials and methods:** We conducted a prospective multicenter study in 3 Pediatric Intensive Care Units in Paris. Patients less than 18 years old were included if they needed norepinephrine because of shock. Systemic (blood pressure, heart rate (HR) and cardiac output (CO)) and cerebral hemodynamics were compared between the time of initiation of norepinephrine infusion (T0), and the steady-state, at least 30 min following the start of treatment or the last dosing modification (Tss). Cardiac output (CO) was assessed using transthoracic cardiac ultrasound. Bilateral middle cerebral artery (MCA) velocities, pulsatility index (PI) and resistance index (RI) were assessed using transcranial Doppler ultrasound. Cerebral tissue oxygen saturation (rScO_2_) was bilaterally recorded (INVOS 5100CTM, Medtronics) and Cerebral Fractional Tissue Oxygen Extraction (cFTOE) was calculated as cFTOE = SpO_2_–rScO_2_/SpO_2_.

**Results:** Fourteen children (median [IQR] age of 3.5 [1; 13.5]) were included. Median [IQR] lactate was 2.4 [1.3; 3.5] mmol/L. At T0, mean left/right rScO_2_ were 67 ± 14%/65 ± 12%, mean left/right cFTOE were 32 ± 13%/35 ± 10%. Median left/right MCA mean velocities were 52 [45; 62] cm/s/47 [38; 64] cm/s, mean left/right PI were 1.4 ± 0.6/1.4 ± 0.6, and mean left/right RI were 0.7 ± 0.2/0.7 ± 0.2. Norepinephrine dosing was 0.2 [0.1; 0.32] μg/kg/min at Tss. Norepinephrine significantly increased arterial blood pressure (mean MAP of 63 ± 14 mmHg at Tss versus 51 ± 12 mmHg at T0, *p* = 0.001) without change in CO nor in HR. Mean/median MCA velocities, PI, RI, rScO_2_ and cFTOE did not significantly change between T0 and Tss (Fig.). Cerebral perfusion time-course evolution showed variability between subjects, attested by an increase of Vm and a decrease of PI at Tss, which slightly improved in 6 patients, remained unchanged in 6, and was altered in 2.

**Conclusion:** This is the first study assessing cerebral effects of norepinephrine in children with shock. In critically ill hypotensive children, low-dose norepinephrine, despite a homogeneous and significant increase in arterial blood pressure, had little and variable effects on cerebral perfusion and oxygenation.

**References**

1. de Azevedo DS, Salinet ASM, de Lima Oliveira M, et al. Cerebral hemodynamics in sepsis assessed by transcranial Doppler: a systematic review and meta-analysis. J Clin Monit Comput. 2017; 31:1123–32.
2. Berg RMG, Plovsing RR, Ronit A, et al. Disassociation of static and dynamic cerebral autoregulatory performance in healthy volunteers after lipopolysaccharide infusion and in patients with sepsis. Am J Physiol Regul Integr Comp Physiol. 2012; 303:R112.

**Compliance with ethics regulations:** Yes in clinical research. 
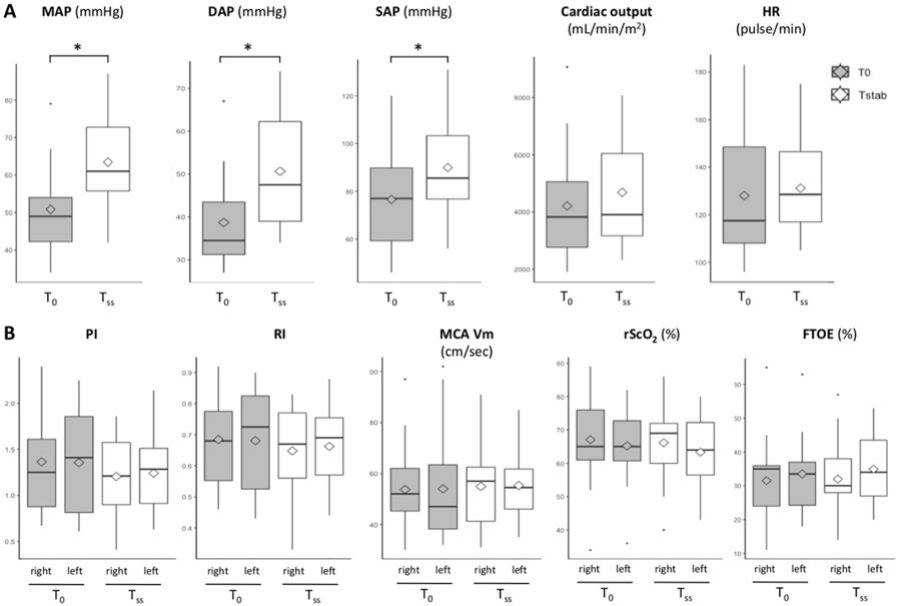


Fig. Evolution of **A** systemic and **B** cerebral hemodynamics during norepinephrine infusion. The central bars and diamonds represent the medians and means, respectively; the lower and upper ends of the box represent the 1st and 3rd quartile, respectively

### CO-007 Neuroprognostication of hypoglycemic encephalopathy: a behavioral, electrophysiological and structural brain imaging study

#### Clémence Marois^1,2^, Aude Sangare^2^, Nadya Pyatigorskaya^1^, Mélanie Valente^2^, Julie Zyss^1^, Virginie Lambrecq^1,2^, Alaina Borden^1^, Loic Le Guennec^1,3^, Nicolas Weiss^1,3^, Benjamin Rohaut^1,2,3^, Sophie Demeret^1^, Louis Puybasset^1,3^, Alexandre Demoule^1,3^, Lionel Naccache^1,2,3^

##### ^1^Pitié Salpêtrière, Paris, France; ^2^ICM: institut cerveau moelle, Paris, France; ^3^Sorbonne Université, Paris, France

**Correspondence:** Clémence Marois - clemence.marois@aphp.fr

*Annals of Intensive Care* 2021, **11(Suppl 1):**CO-007

**Rationale:** Predicting the functional recovery of patients with disorders of consciousness (DOC) due to severe hypoglycemia is challenging. Data on outcome, neurophysiological, or imaging studies are scarce. The aim of this study was to determine to what extent clinical examination, neuroimaging, and neurophysiology could predict the neurological outcome of these patients.

**Patients and methods/materials and methods:** Consecutive patients with DOC related to hypoglycemia admitted to an expert center for neuroprognostication from 2010 to 2020 were included. Multimodal neurological assessment included clinical examination encompassing Coma Recovery Scale Revised (CRS-R), electroencephalography (EEG), somatosensory and cognitive evoked potentials, morphological MRI and diffusion tensor imaging with the quantification of fractional anisotropy (FA) in the white matter and deep ganglia. Patients’ functional outcome was evaluated using the extended Glasgow Outcome Scale (GOSe) at 1 year.

**Results:** Twenty-one patients were screened, 2 were excluded because of another possible cause of encephalopathy. Nineteen patients were analysed, with an age of 52 years [36–58]. The assessment was performed with a median delay of 66 days [29–106] after hypoglycemia. Median CRS-R was 7.5 [6–10] on the day of multimodal assessment. A standard EEG was performed in all patients, and showed a diffuse slowing of background activity, associated with triphasic slow waves in 26%, asymmetrical patterns in 34% and epileptic activities in 26% of EEGs. Somatosensory and cognitive evoked potentials were performed, respectively, in 5 and 17 patients. All patients had brain MRI that revealed transient or irreversible lesions. The diffusion tensor imaging was performed in 13 patients. Median global FA was 0.73 [0.6–0.8]. At 1 year, 13 (68%) patients were dead, 1 (5%) remained unresponsive, 3 (15%) were in a minimally conscious state and 2 (10%) were conscious with severe disabilities (GOSe 3 and 4). All patients had abnormal EEG, but without specific pattern correlated with outcome. Evoked potentials were not correlated with neurological prognosis. The size and locations of MRI lesions were not correlated to clinical outcome. A global FA value lower than 0.91 had a negative predictive value of 100% and a positive predictive value of 92% for the prediction of being conscious at 1 year.

**Conclusion:** At 1 year, 90% of patients admitted to an intensive care unit for DOC due to severe hypoglycemia were dead or had not recovered consciousness. Neurophysiological prognostic markers were poorly predictive of neurological outcome, whereas MRI brain structural connectivity analysis showed much better performance. Larger studies are needed to confirm those preliminary results.

**Reference**

1. Barbara G et al. Functional outcome of patients with prolonged hypoglycemic encephalopathy. Ann Intensive Care. 2017; 7(1):54. 10.1186/s13613-017-0277-2.

**Compliance with ethics regulations:** Yes in clinical research.

### CO-008 Increasing accessibility of somatosensory-evoked potentials in the ICU: a new method of screening for cortical responses

#### Aude Sangare^1^, Benjamin Rohaut^1^, Alaina Borden^1^, Julie Zyss^1^, Kevin Doyle^2^, Angela Velasquez^2^, Lionel Naccache^1^, Jan Claasen^2^

##### ^1^Hopital Pitié Salpêtrière, Paris, France; ^2^Columbia University, New York, Etats-Unis

**Correspondence:** Aude Sangare - aude.sangare@icm-institute.org

*Annals of Intensive Care* 2021, **11(Suppl 1):**CO-008

**Rationale:** Somatosensory evoked potentials (SSEP) are known to accurately predict unfavorable outcome in survivors after cardiac arrest and are also used as an aid in determining prognosis in other acute brain injuries. However, SSEP are not universally accessible. Here, we described a simple approach to screening for cortical SSEPs using a widely available, inexpensive peripheral nerve stimulator used for monitoring of paralysis in critical care and anaesthesia (“train of four” [TOF]) along with a standard electroencephalographic (EEG) recording.

**Patients and methods/materials and methods:** The peripheral nerve stimulator was applied to the median nerve at the wrist with a TOF at 1 Hz over 10 min on both sides and recorded with a standard 21-channel EEG system (10–20 montage). Two additional bipolar electrodes were applied at the wrist to detect the TOF stimulation. To facilitate the interpretation of SSEP responses, 3 methods of analysis were combined. Components were identified and labelled based on visual inspection of the averaged signal. Parametric analyses using t-scores against baseline were used to evaluate the presence or the absence of components, and support vector machine (SVM) classifiers between left and right stimulations were computed. Data analysis was performed with Brainstorm, an open-source toolbox. The method was validated in 12 healthy volunteers and then applied to 10 comatose patients. In patients, this new screening method was compared with the standard reference method according to international SSEP acquisition guidelines.

**Results:** The short latency components (N20, N30) and middle latency components (P45, N60) were statistically detected in all healthy subjects (*p* < 0.05 after correction by the false discovery rate for multiple comparisons over time and channel dimensions). With the SVM, the area under curve (AUC) from the receiver operating characteristic (ROC) was superior to 90% to detect short middle and late latency components. On the 10 comatose patients, the screening method and the “standard reference method” showed the same performance in detecting N20 components (N20 components were unilaterally present in two patients, bilaterally absent in one patient and bilaterally present in 7 patients).

**Conclusion:** SSEP can reliably be detected using a simple nerve stimulator using routine clinical EEG recordings in the ICU. We propose a simplified and accessible alternative procedure to screen for SSEP cortical responses in the ICU. Diagnostic and prognostic performance of this new screening method need to be evaluated in a larger cohort.

**Compliance with ethics regulations:** Yes in clinical research.

### CO-009 EEG findings in COVID-19 critically ill patients with ARDS: a prospective multicentric observational study

#### Bertrand Hermann^1,5,6^, Jean-Luc Diehl^1,5,7,9^, Alain Cariou^2,5,8^, Angela Marchi^4,5^, Martine Gavaret^4,5,10^, Jean-Loup Augy^1,5^, Julien Charpentier^2^, Frédéric Pène^2,5^, Jean-Paul Mira^2,5^, Caroline Hauw-Berlemont^1^, Tarek Sharshar^3,5^, Sarah Benghanem^2,5^

##### ^1^Médecine Intensive Réanimation, Hôpital Européen Georges Pompidou, Assistance Publique - Hôpitaux de Paris-Centre (APHP-CUP), Paris, France; ^2^Médecine Intensive Réanimation, Hôpital Cochin Assistance Publique - Hôpitaux de Paris-Centre (APHP-CUP), Paris, France; ^3^Neuroréanimation, GHU Sainte Anne, Paris, France; ^4^Neurophysiologie Clinique, GHU Sainte Anne, Paris, France; ^5^Faculté de Médecine, Université de Paris, Paris, France; ^6^Institut du Cerveau et de la Moelle Epinière, INSERM U1127, Paris, France; ^7^Innovative Therapies in Haemostasis, INSERM U1140, Paris, France; ^8^Paris-Cardiovascular-Research-Center, INSERM U970, Paris, France; ^9^Biosurgical Research Lab (Carpentier Foundation), Paris, France; ^10^Institut de Psychiatrie et Neuroscience de Paris, INSERM U1266, Paris, France

**Correspondence:** Bertrand Hermann - bertrand.hermann@aphp.fr

*Annals of Intensive Care* 2021, **11(Suppl 1):**CO-009

**Rationale:** EEG abnormalities have been reported in COVID-19 patients but few data exist on critically ill patients and on the association of EEG with neurological outcomes. We aimed to describe qualitative EEG patterns in critically ill mechanically ventilated COVID-19 patients.

**Patients and methods/materials and methods:** A prospective bicentric observational study was conducted in two French ICUs between April and December 2020, including mechanically ventilated moderate-to-severe COVID-19 ARDS patients. EEG were performed at two timepoints, first under sedation and second 4 to 7 days after sedation discontinuation. Primary endpoints were the incidence of EEG abnormalities. Secondary outcomes were the univariate association of EEG abnormalities with day-28 outcomes.

**Results:** Fifty-two patients were included. Median duration of coma was 14 [5–26] days. Delirium was present in 32 (62%) patients, representing 74% of patients who awoke from coma, with a predominance of mixed delirium (62%), and median duration of 5 [3–8] days. The first EEG was performed after a median delay of 4 [3–7] days from ICU admission. EEG were mostly symmetric (96%) with a dominant background rhythm in the theta range (4–8 Hz) in 58% patients and in the delta range (1–4 Hz) in 35%. Discontinuous and/or suppressed background activity was observed in 25 (48%) patients and EEG was unreactive in 17 (33%) patients. Paroxysmal activity with bi-frontal slow waves was observed in 17 (33%) patients, and only 1 (1.9%) patient presented a seizure during the recording. The second EEG was performed in 39 (92%) out of the 42 patients alive 4 to 7 days after sedation weaning at a median of 17 [10–24] days from ICU admission. Theta and delta dominant background were observed in 17 (44%) and 2 (5%) of patients, respectively. Absence of EEG reactivity was observed in 6 patients (15%), discontinuous background in 1 (2.6%) and bi-frontal slow waves in 12 (31%). During the first EEG, a discontinuous background was associated with lower day-28 ventilator-free days (0 [0–10] vs. 10 [0–17], *p* = 0.037), coma-free days (3 [1–11] vs. 15 [3–22]), *p* = 0.047, delirium-free days (3 [0–10] vs. 13 [2–20], *p* = 0.044) and higher mortality (10 (40%) vs. 1 (3.7%), *p* = 0.004) while bi-frontal slow waves were associated with higher coma-free days (3 [1–16] vs. 18 [6–22], *p* = 0.023) and delirium-free days (3 [0–11] vs. 15 [5–21], *p* = 0.043).

**Conclusion:** Early severe EEG abnormalities such as discontinuous background activity are frequent in critically ill COVID-19 and are associated with day-28 outcome, with some EEG abnormalities persisting long after ICU admission.

**Compliance with ethics regulations:** Yes in clinical research.

### CO-010 Frontal EEG vs. standard EEG for prediction of mortality and neurologic outcome in adults under VA-ECMO

#### Cyril Touchard^1^, Jérôme Cartailler^1^, Geoffroy Vellieux^2^, Etienne De Montmollin^2^, Ruben Wanono^2^, Jean Reuter^2^, Marylou Para^2^, Lila Bouadma^2^, Jean-François Timsit^2^, Marie-Pia D’Ortho^2^, Nathalie Kubis^1^, Anny Rouvel Tallec^2^, Romain Sonneville^2^

##### ^1^Hôpital Lariboisière, Paris, France; ^2^Hôpital Bichat, Paris, France

**Correspondence:** Cyril Touchard - cyriltouchard@hotmail.fr

*Annals of Intensive Care* 2021, **11(Suppl 1):**CO-010

**Rationale:** EEG-based prognostication studies in the ICU often rely on 21-electrode recording, which requires substantial human, technical, and financial resources. Here, we compared performances between a simplified 4 frontal electrode montage (4-frontEEG) and a standard one with 21 electrodes (stdEEG) to predict short-term mortality and neurological outcomes in adult patients under veno-arterial extracorporeal membrane oxygenation (VA-ECMO).

**Patients and methods/materials and methods:** Continuous 30-min standard EEG recorded in 118 adult patients with refractory cardiogenic shock that required VA-ECMO. EEG patterns of interest, including EEG reactivity, continuity, background rhythm and the Synek score, were all assessed by an intensivist on the 4-frontEEG montage and then compared to an expert’s interpretation using standard stdEEG recordings. Age, Sepsis-related Organ Failure Assessment (SOFA) score at the time of ECMO cannulation and EEG criteria were used to predict 28-day mortality and 90-day poor functional outcome (i.e. a score of 4–6 on the modified Rankin scale).

**Results:** The detection of EEG patterns using 4-frontEEG was statistically comparable with the stdEEG for background rhythm (rho = 0.66, *p* < 0.001, Spearman rank test), discontinuity (= 0.955 Cohen’s kappa), reactivity (= 0.739) and the Synek score (rho = 0.794, *p* < 0.001, Spearman rank test). Using Synek’s classification from the simplified 4-electrode montage, we found that a higher score was a risk factor independent of age and SOFA score for both 28-day mortality (adjusted OR = 1.57 [1.17, 2.11], *p* = 0.003) and 90-day poor functional outcome (adjusted OR = 1.75 [1.21, 2.53], *p* = 0.003).

**Conclusion:** A simplified EEG montage using 4 frontal electrodes satisfactorily predicted short-term mortality and functional outcomes in adult patients under VA-ECMO, with a performance similar to that of standard EEG montage. This simplified montage could be implemented as part of a multimodal evaluation for bedside prognostication.

**Compliance with ethics regulations:** Yes in clinical research. 
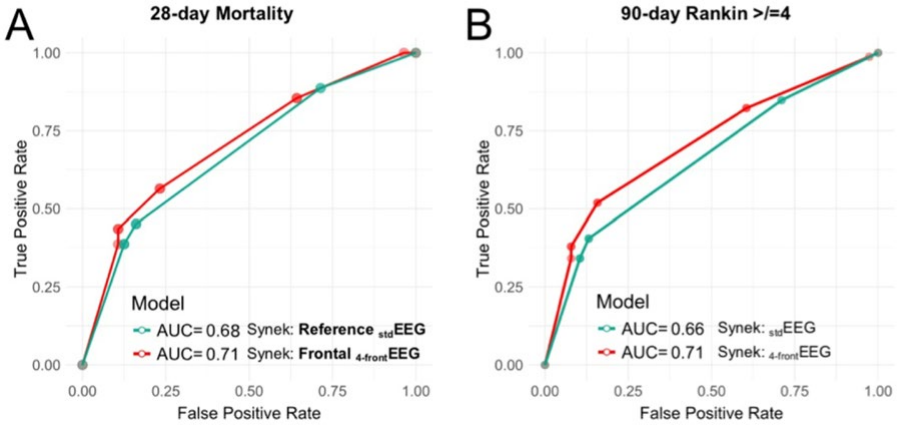


AUC of reference (stdEEG) and frontal (4-frontEEG) montage for 28-day mortality and 90-day poor functional outcome (Rankin score ≥ 4)

### CO-011 Multimodal FDG-PET and EEG assessment improves diagnosis and prognostication of disorders of consciousness

#### Bertrand Hermann^1,2^, Johan Stender^2^, Marie-Odile Habert^2,3^, Federico Raimondo^4^, Pauline Perez^2^, Benjamin Rohaut^2,3^, Jacobo Sitt^2^, Lionel Naccache^2,3^

##### ^1^Hôpital Européen Georges Pompidou, APHP, Paris, France; ^2^Institut du Cerveau et de la Moelle Epinière, Inserm 1127, Paris, France; ^3^Groupe hospitalier Pitié-Salpêtrière, APHP, Paris, France; ^4^Coma Science Group, GIGA Consciousness, Université de Liège, Paris, Belgique

**Correspondence:** Bertrand Hermann - bertrand.hermann@aphp.fr

*Annals of Intensive Care* 2021, **11(Suppl 1):**CO-011

**Rationale:** Functional brain-imaging techniques have revealed that clinical examination of disorders of consciousness (DoC) can underestimate the level of consciousness of patients. FDG-PET metabolic index of the best preserved hemisphere (MIBH) has been reported as a promising measure of consciousness, but has never been externally validated and compared with other brain-imaging diagnostic procedures such as quantitative EEG. Moreover, while advocated by guidelines, data are lacking on the combination of diagnostic and prognostic procedures.

**Patients and methods/materials and methods:** FDG-PET, quantitative EEG and cognitive evoked potential using the auditory oddball local–global paradigm were performed in minimally conscious state (MCS) and vegetative state/unresponsive wakefulness syndrome (VS/UWS) patients. We compared out-sample diagnostic and prognostic performances of PET-MIBH and EEG-based classification (using a support vector machine algorithm classifier) of conscious state to the current behavioral gold-standard, the Coma Recovery Scale—revised (CRS-R). Lastly, we explored the potential benefit of combining imaging techniques for both the diagnosis and prognostication of DoC.

**Results:** Between January 2016 and October 2019, 52 patients were included: 21 VS/UWS and 31 MCS. PET-MIBH had an AUC of 0.816 [0.681–0.928], sensitivity of 84% [66–95] and specificity of 76% [53–92], not significantly different from the quantitative EEG classifier AUC 0.770 [0.619–0.896] (*p* = 0.628). Their combination accurately identified almost all MCS patients with a sensitivity of 94% [79–99%] and specificity of 67% [43–85]. Multimodal assessment also identified VS/UWS patients with neural correlate of consciousness on the local–global cognitive evoked potentials (4/7 (57%) vs. 1/14 (7%), *p* = 0.025) (Figure). Lastly, PET and EEG combination was significantly associated with 6-month recovery of command-following in initially unresponsive patients (recovery in 9/24 (38%) of patients with high FDG-PET metabolism and/or rich EEG activity vs. in 0/16 (0%) of patients with low-level FDG-PET metabolism and EEG activity, Fisher’s exact test *p* = 0.006), outperforming each technique taken in isolation.

**Conclusion:** FDG-PET MIBH is an accurate and robust procedure across sites to diagnose MCS. Its combination with EEG-based classification of conscious state not only optimizes diagnostic performances, but also allows to detect covert cognition and to predict 6-month command-following recovery, demonstrating the added value of multimodal assessment of DoC.

**Compliance with ethics regulations:** Yes in clinical research. 
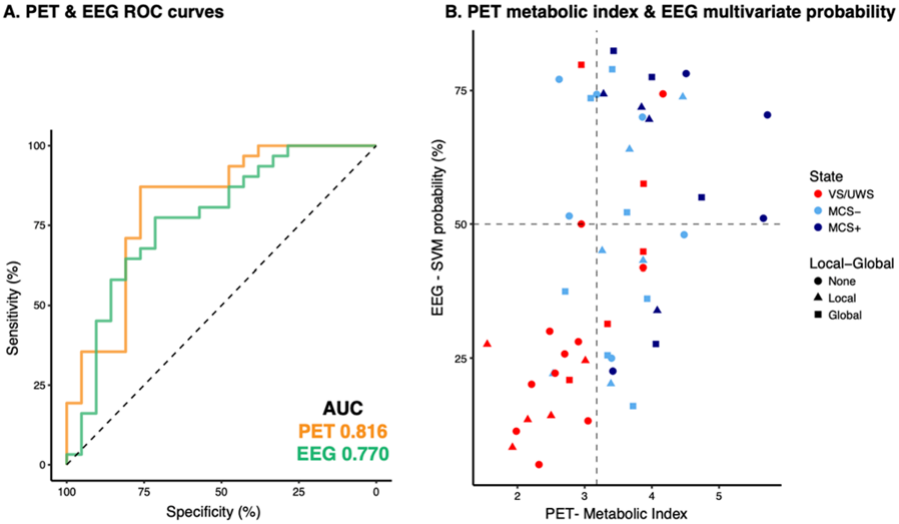


FDG-PET and EEG out-of-sample diagnostic performances. **A** ROC curves of out-of-sample diagnostic performances of FDG-PET (yellow) and EEG-based classification (green) with corresponding discrimination area under the curve (AUC). **B** Scatterplot representation

### CO-012 Neurological follow-up findings in adult survivors of COVID-19-associated acute respiratory distress syndrome invasively ventilated in the ICU: a prospective two-center study

#### Pierre Jaquet^1^, Camille Legouy^2^, Lucie Lefevre^1^, Juliette Patrier^1^, Etienne De Montmollin^1^, Paul-Henri Wicky^1^, Antoine Shenouda^1^, Serafima Vledouts^1^, Lila Bouadma^1^, Jean-François Timsit^1^, Tarek Sharshar^2^, Romain Sonneville^1^

##### ^1^Hôpital Bichat Claude Bernard, Paris, France; ^2^Hôpital Saint-Anne, Paris, France

**Correspondence:** Pierre Jaquet - pierre.jaquet@aphp.fr

*Annals of Intensive Care* 2021, **11(Suppl 1):**CO-012

**Rationale:** As COVID-19 pandemic started in March 2020, the long-term health consequences remain largely unclear. Acute respiratory distress syndrome (ARDS) largely affects the nerves, muscles, and central nervous system, leading to long-term motor and cognitive impairment. The aim of this study was to describe 3- to 6-month neurocognitive and functional outcomes of survivors of COVID-19-associated ARDS invasively ventilated in the ICU during the first wave of the pandemic.

**Patients and methods/materials and methods:** This was a prospective study conducted in two ICUs of academic hospitals in the Paris area, France. All ARDS survivors were eligible for a dedicated neurological consultation performed between 3- and 6-months post-ICU discharge. Two neurologists assessed neurocognitive and functional outcomes using standardized scales: the Medical Research Council (MRC) scale, the Montreal Cognitive Assessment (MOCA) scale, the Hospital Anxiety and Depression (HAD) scale, the quick inventory of depressive symptomatology (qids-sr16) scale, the modified Rankin (mRS) scale and the instrumental activity of daily living (IADL) scale. Critical illness neuromyopathy was defined by a MRC score < 46, mild cognitive impairment was defined by a MOCA score < 26, and a poor functional outcome was defined by a mRS > 2 (moderate-to-severe disability). Data are presented as number (percentages) or median [interquartile range].

**Results:** Between March and June 2020, 160 patients were hospitalized in ICU for SARS-CoV-2-associated pneumonia. Of them, 97 required invasive mechanical ventilation and 50 (51.5%) patients died in ICU. From 47 eligible survivors, 10 (21%) patients were lost to follow-up and 37 patients were included. The median time between ICU discharge and follow-up consultation was 4 (3.6–5.6) months. Main neurological findings included critical illness neuromyopathy in 4 (12%) cases, mild cognitive impairment in 16/29 (55%) patients, and moderate-to-severe functional disability in 7 (19%) patients. HAD anxiety score was 5 [3; 7] and HAD depression was 3.5 [1; 8.25], and qids-sr16 was 0 [0; 0]. The IADL was 7 [3–8] in women and 5 [5–5] in men. During the post-intensive care consultation, intervention was required for 26 (68%) patients. The main prescriptions during neurological consultation included targeted physiotherapy (*n* = 20, 59%), speech therapy (*n* = 5, 15%), psychotherapy (*n* = 13, 38%), and cognitive rehabilitation for 11 (32%) patients (Table).

**Conclusion:** A follow-up neurological consultation in survivors of COVID-19-associated ARDS revealed a high burden of neurological impairment, including mild cognitive impairment and moderate-to-severe functional disability in one-in-two and one-in-five patients, respectively.

**Reference**

1. Herridge MS, Diaz-Granados N, Cooper A, et al. Functional disability 5 years after acute respiratory distress syndrome. N Engl J Med. 2011; 12.

**Compliance with ethics regulations:** Yes in clinical research. 
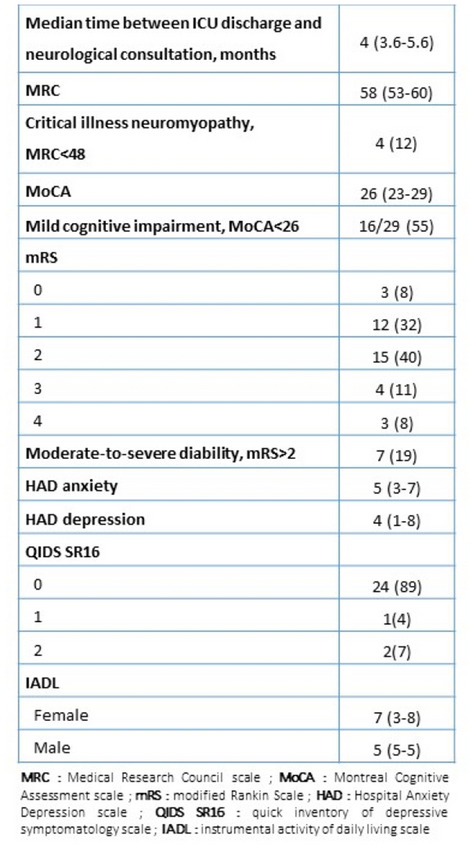


Neurological follow-up in survivors of COVID-19-associated acute respiratory distress syndrome

### CO-013 Blood fibrocytes are associated with severity and prognosis in COVID-19 pneumonia

#### Mada Ghanem^1,2^, Méline Homps-Legrand^1^, Lise Morer^2^, Tiphaine Goletto^2^, Justine Frija^2^, Paul Henry Wicky^3^, Pierre Jaquet^3^, Madeleine Jaillet^1^, Arnaud Mailleux^1^, Etienne De Montmollin^3^, Catherine Neukirch^1,2^, Raphael Borie^1,2^, Camille Taille^1,2^, Bruno Crestani^1,2^

##### ^1^Inserm U1152, Université de Paris, Paris, France; ^2^Service de Pneumologie, Hôpital Bichat, APHP, Paris, France; ^3^Service de Réanimation Médicale et Infectieuse, Hôpital Bichat, APHP, Paris, France; ^4^Service d’Anesthésie-Réanimation chirurgicale, Hôpital Saint-Antoine, Hôpital Bichat, APHP, Paris, France

**Correspondence:** Mada Ghanem - mada.ghanem@yahoo.fr

*Annals of Intensive Care* 2021, **11(Suppl 1):**CO-013

**Rationale:** COVID-19 can lead to severe acute pneumonia in 15–20% of patients. Circulating fibrocytes are fibroblasts precursors involved in the repair process. Increased blood fibrocytes count is associated with a poor prognosis in fibrotic lung diseases and acute respiratory distress syndrome (ARDS). We aimed to quantify the percentage of circulating fibrocytes in patients hospitalized for COVID-19 and included in the French COVID cohort, in order to determine their prognostic value in this disease.

**Patients and methods/materials and methods:** COVID-19 was confirmed by PCR in all patients. Blood fibrocytes were quantified by flow cytometry as CD45+/CD15−/CD34+/Collagen-1+. Clinical and imaging data were obtained at inclusion and at 3-month follow-up. In a subgroup of patients admitted in ICU, we quantified fibrocytes in blood and broncho-alveolar lavage fluid (BALF). Serum amyloid protein (SAP), a known regulator of fibrocyte differentiation, was quantified by ELISA in serum samples.

**Results:** We included 57 patients admitted for hypoxemic COVID-19 pneumonia (mean age 59 years [23–87]) and 16 healthy controls. Samples were taken 11 days [4–31] after the first symptoms. The median percentage of circulating fibrocytes was higher in patients compared to controls (3.6% vs 2.1%, *p* = 0.02). The percentage was lower in patients who died of COVID-19 (6/57) as compared to survivors (1.6% versus 3.7%, *p* = 0.03). Fibrocyte percentage did not correlate with biological severity markers (lymphocytes, LDH, ferritin, CRP). Thirty-two patients were evaluated 3 months after admission. A complete resolution of CT abnormalities was observed in 13 patients (40%) and was associated with a significantly higher initial fibrocyte count compared with patients with an incomplete resolution (4.5% vs 3.4%, *p* = 0.03). SAP concentration in serum was higher in COVID-19 patients compared to controls (96.3 vs 65.0 mg/L, *p* = 0.0021). SAP concentration did not correlate with fibrocyte count. In 7 ICU patients (mean age 62 years [50–73]), median blood fibrocyte count was 0.94% while median BALF fibrocyte count was 6.7%, suggesting a recruitment of fibrocytes to the lung in severe cases.

**Conclusion:** Circulating fibrocytes were increased in patients with hypoxemic COVID-19 pneumonia. Lower fibrocyte count were associated with an increased risk of in-hospital death and a slower resolution of lung CT opacities, and may be due to the recruitment of fibrocytes to the lung in the most severe cases.

**Compliance with ethics regulations:** Yes in clinical research.

### CO-014 Delayed inflammation control is associated with mortality in tocilizumab-treated critically ill SARS-CoV-2 patients: a matched cohort analysis

#### Tomas Urbina^1^, Jean-Rémi Lavillegrand^1^, Marc Garnier^1^, Arsene Mekinian^1^, Jérôme Pacanowski^1^, Nathalie Mario^1^, Guillaume Dumas^2^, Geoffroy Hariri^1^, Antoine Pilon^1^, Lucie Darrivière^1^, Muriel Fartoukh^3^, Bertrand Guidet^1^, Eric Maury^1^, Judith Leblanc^1^, Yannick Chantran^1^, Olivier Fain^1^, Karine Lacombe^1^, Guillaume Voiriot^3^, Hafid Ait-Oufella^1^

##### ^1^Hôpital Saint-Antoine, Assistance-Publique des Hôpitaux de Paris (AP-HP), Paris, France; ^2^Hôpital Saint-Louis, Assistance-Publique des Hôpitaux de Paris (AP-HP), Paris, France; ^3^Hôpital Tenon, Assistance-Publique des Hôpitaux de Paris (AP-HP), Paris, France

**Correspondence:** Tomas Urbina - tomas.urbina@aphp.fr

*Annals of Intensive Care* 2021, **11(Suppl 1):**CO-014

**Rationale:** SARS-CoV-2 infection triggers a high production of interleukin-6 (IL-6), which may participate in lung damage. No convincing evidence for a survival benefit of anti-IL-6 receptor treatment such as tocilizumab is yet available. Whether identifying a subset of responders, optimal timing or need for higher doses or re-injection would alter survival needs a better understanding of the immuno-inflammatory response to tocilizumab.

**Patients and methods/materials and methods:** We conducted a retrospective multicenter matched cohort analysis in three ICUs in 2 tertiary care hospitals. Critically ill SARS-CoV-2 patients with plasma inflammatory biomarker measurements between March and April 2020 were included. Tocilizumab-treated patients were matched on a 1:2 ratio to non-treated patients based on gender and SAPS II. Biomarkers including IL-6, C-reactive protein (CRP), and fibrinogen were collected within the first days of admission (T1), 3 days (T2) and 7 days (T3) later. Outcome was day-60 censored survival.

**Results:** Twenty-one tocilizumab-treated patients and 42 matched controls were included. PaO_2_/FiO_2_ ratios were, respectively, 133 [93–169] vs 140 [114–224] mmHg. During ICU stay, patients received mechanical ventilation in 76% vs. 79% and prone positioning in 33% vs. 52%. When compared to matched controls, tocilizumab-treated patients had persistently higher IL-6 plasma levels (T1: 1244 [712–2561] vs 72 [32–195], T2: 1348 [486–2768] vs 36 [18–75], T3: 751 [630–1798] vs 82 [50–104] pg/mL, *p* < 0.001) and persistently lower CRP and fibrinogen levels. Among tocilizumab-treated patients, baseline levels of inflammatory biomarkers were not different according to outcome but CRP and fibrinogen decrease was delayed in non-survivors (Fig. 1). CRP decreased at T1 in survivors (45 [30–98] vs 170 [69–204] mg/L, *p* < 0.001), but only at T2 in non-survivors (37 [13–74] vs 277 [235–288], *p* = 0.03). Fibrinogen decreased at T2 in survivors (4.11 [3.58–4.69] vs 6.14 [5.61–7.85] g/L, *p* = 0.005), but not in non-survivors (4.79 [4.12–7.58] vs 7.24 [6.22–9.24] g/L, *p* = 0.125).

**Conclusion:** Tocilizumab leads to persistent increase in plasma IL-6, and decrease in both CRP and fibrinogen. Among tocilizumab-treated patients, the decrease in inflammatory biomarker was delayed in non-survivors

**Compliance with ethics regulations:** Yes in clinical research. 
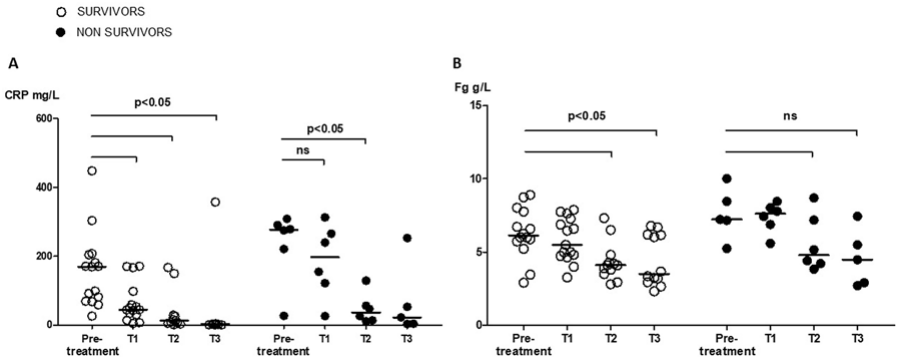


Fig. 1 **A** CRP and **B** Fibrinogen plasma level kinetics amongst patients treated with tocilizumab, according to survival.

### CO-015 Head-to-head comparison of coagulation, platelet activation and NETosis in septic shock and critical Covid-19

#### Melanie Dechamps^1,2^, Julien De Poortere^1^, Pierre-François Laterre^2^, Sandrine Horman^1^, Christophe Beauloye^1,2^

##### ^1^Institut de Recherche Experimentale et Clinique UCLouvain, Bruxelles, Belgique; ^2^Cliniques Universitaires Saint-Luc UCLouvain, Bruxelles, Belgique

**Correspondence:** Melanie Dechamps - melanie.dechamps@uclouvain.be

*Annals of Intensive Care* 2021, **11(Suppl 1):**CO-015

**Rationale:** Septic shock is a dysregulated response to infection, generating an important inflammatory reaction, endothelial activation and a procoagulant state leading to microvascular thrombosis and subsequent organ impairment^1^. Similarly, a severe inflammatory reaction and a coagulopathy with pulmonary micro-thrombosis eventually leading to acute lung injury, is a typical feature of critical forms of Coronavirus disease 2019 (Covid-19)^2^. However, the etiology and clinical presentation of these two diseases substantially differ, suggesting that different mechanisms may underlie the procoagulant state in septic shock versus critical Covid-19. Our aim was to compare coagulation, platelet activation and platelet–neutrophil interplay between control, septic shock and critical Covid-19 patients.

**Patients and methods/materials and methods:** A total of 118 patients were included in our prospective, monocentric, observational study between February 2019 and June 2020. Ethics committee approved the study protocol and all patients signed an informed consent (B403201938590, NCT04107402). Control patients (*n* = 48) were recruited at central lab consultation. Septic shock (*n* = 48) and Covid-19 (*n* = 22) patients were consecutively included at admission in our ICU department.

**Results:** Septic shock patients had worse severity scores due to multiple organ failure (assessed by APACHE II and SOFA score), whereas Covid-19 patients had more severe respiratory failure and a longer ICU length-of-stay. At the time of inclusion, CRP and lymphocyte count were comparable between septic shock and Covid-19 patients. White cell count and neutrophil count were higher for septic shock patients. Analysis of coagulation showed prolonged INR, TT and aPTT in septic shock although only INR was prolonged in Covid-19. Thrombin–antithrombin complex (TATc) formation was similar in both pathologies, whereas consumption of antithrombin III (ATIII) and D-dimers formation was more pronounced in septic shock. Platelet count was lower in septic shock and platelet activation, assessed via plasmatic levels of soluble P-selectin (sCD62P) and Trem-like transcript 1 (sTLT-1) was more important in septic shock. Neutrophil activation and NETosis, evaluated by levels of circulating myeloperoxidase (MPO) and citrullinated histone 3 (H3-Cit), was similarly increased in both groups.

**Conclusion:** This study confirmed an activation of coagulation cascade, platelet activation and NETosis in both septic shock and critical Covid-19, compared with control patients. Importantly, the extent of these changes was similar or less pronounced in critical Covid-19 compared with septic shock.

**References**

1. Hotchkiss RS et al. Sepsis and septic shock. Nat Rev Dis Primers. 2016; 2: 16045.
2. Iba T et al. The unique characteristics of COVID-19 coagulopathy. Crit Care. 2020; 24(1): 360.

**Compliance with ethics regulations:** Yes in clinical research. 
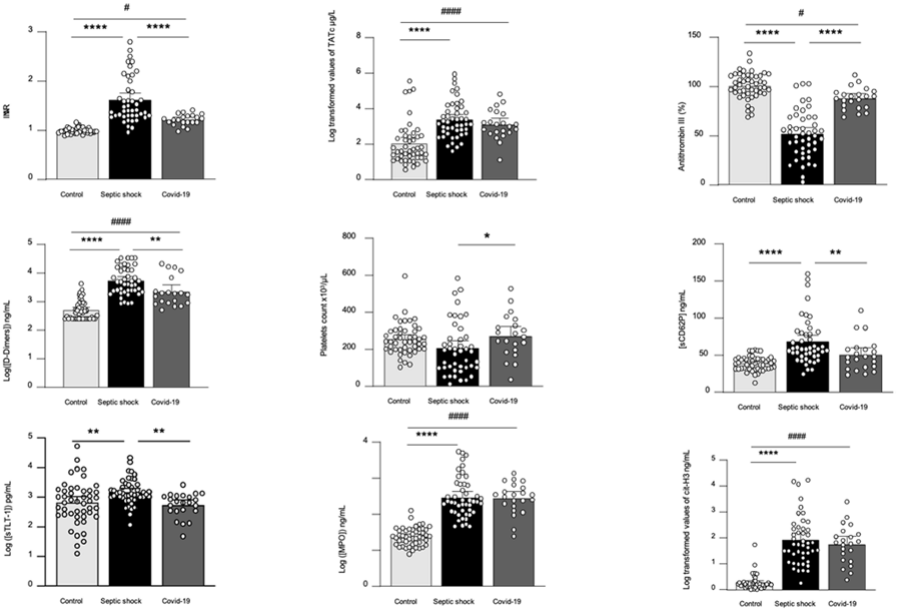


Comparison of coagulation, platelet activation and NETosis in septic shock and critical Covid-19

### CO-016 Elevated plasma IL-6 and CRP levels are associated with adverse clinical outcomes and death in critically ill SARS-CoV-2 patients: inflammatory response of SARS-CoV-2 patients

#### Jean-Rémi Lavillegrand^1^, Marc Garnier^1^, Agathe Spaeth^1^, Nathalie Mario^1^, Geoffroy Hariri^1^, Antoine Pilon^1^, Enora Berti^2^, Fabienne Fieux^1^, Sara Thietart^1^, Tomas Urbina^1^, Matthieu Turpin^2^, Lucie Darrivere^1^, Muriel Fartoukh^2^, Franck Verdonk^1^, Guillaume Dumas^3^, Alain Tedgui^4^, Bertrand Guidet^1^, Eric Maury^1^, Yannick Chantran^1^, Guillaume Voiriot^2^, Hafid Ait-Oufella^1^

##### ^1^APHP-CHU Saint Antoine, Paris, France; ^2^APHP-CHU Tenon, Paris, France; ^3^APHP-CHU Saint Louis, Paris, France; ^4^Centre de recherche cardiovasculaire de Paris, Equipe 5, U970, Paris, France

**Correspondence:** Jean-Rémi Lavillegrand - jrlavillegrand@gmail.com

*Annals of Intensive Care* 2021, **11(Suppl 1):**CO-016

**Rationale:** SARS coronavirus 2 (SARS-CoV-2) is responsible for high morbidity and mortality worldwide, mostly due to the exacerbated inflammatory response observed in critically ill patients. However, little is known about the kinetics of the systemic immune response and its association with survival in SARS-CoV-2-positive patients admitted in ICU. We aimed to compare the immuno-inflammatory features according to organ failure severity and in-ICU mortality.

**Patients and methods/materials and methods:** This was a 6-week tricentric study including SARS-CoV-2-positive patients admitted in ICU. Analysis of plasma biomarkers at days 0 and 3–4 were performed according to organ failure worsening (increase in SOFA score) and 60-day mortality.

**Results:** 101 patients were included. Patients had severe respiratory diseases with PaO_2_/FiO_2_ of 155 [111–251] mmHg), SAPS II of 37 [31–45] and SOFA score of 4 [3–7]. Eighty-three patients (83%) required endotracheal intubation/mechanical ventilation and among them, 64% were treated with prone position. IL-1β was barely detectable. Baseline IL-6 levels positively correlated with organ failure severity. Baseline IL-6 and CRP levels were significantly higher in patients in the worsening group than in the non-worsening group (278 [70–622] vs. 71 [29–153] pg/mL, *P* < 0.01; and 178 [100–295] vs. 100 [37–213] mg/L, *P* < 0.05, respectively). Baseline IL-6 and CRP levels were significantly higher in non-survivors compared to survivors, but fibrinogen levels and lymphocyte counts were not different between groups. After adjustment on SOFA score and time from symptom onset to first dosage, IL-6 and CRP remained significantly associated with mortality. IL-6 changes between Day 0 and Day 3–4 were not different according to the outcome. On the contrary, kinetics of CRP and lymphocyte count were different between survivors and non-survivors.

**Conclusion:** In SARS-CoV-2-positive patients admitted in ICU, a systemic pro-inflammatory signature was associated with clinical worsening and 60-day mortality.

**Compliance with ethics regulations:** Yes in clinical research

### CO-017 Subcutaneous interferon-β-1b in COVID-19 ARDS: a quasi-experimental study

#### Syrine Maatouk, Manel Lahmar, Zeineb Hamouda, Wiem Nouira, Saoussen Benabdallah, Fahmi Dachraoui, Lamia Ouanes-Besbes, Fekri Abroug

##### CHU F. Bourguiba, Monastir, Tunisie

**Correspondence:** Fekri Abroug - ekri.abroug@gmail.com

*Annals of Intensive Care* 2021, **11(Suppl 1):**CO-017

**Rationale:** Severe Covid-19 has been linked to an insufficient production of type I interferon (IFN) which has in vitro activity against SARS-CoV-2. We conducted a quasi-experimental study evaluating a subcutaneous course of interferon β-1b in ICU patients requiring respiratory support.

**Patients and methods/materials and methods:** Consecutive ICU patients with Covid-ARDS ventilated by high-flow nasal cannula (HFNC) were included. Interferon β-1b was randomly administered subcutaneously in addition to standard of care. The main outcome was the need of intubation based on standardized criteria.

**Results:** Thirty-two patients (24 men, median (IQR) age: 60.5 (50–70) years), were included in the study. The disease severity is reflected by SpO_2_ on ambient air (75% (66–78)), PaO_2_/FiO_2_ (87 (63–120). Ten of the included patients received interferon β-1b for an average duration of 6 ± 2 days. The study groups were well-balanced and no single variable was statistically different. More patients from the control group met the criteria of tracheal intubation: 14 (63.6%) vs 2 (20%) in the control and treatment groups, respectively; *p* = 0.02. Intubation was also significantly delayed (log-rank test *p* = 0.03). Untreated patients had more frequent bacterial superinfections. Septic shock occurred more frequently in patients who did not receive interferon β-1b (50% vs 10%, respectively; *p* = 0.04). Overall, in-hospital mortality was more frequent in patients who did not receive interferon β-1b, 13 (59%) vs 2 (20%) in treated patients; *p* = 0.04.

**Conclusion:** A short subcutaneous course of interferon β-1b in ICU patients managed with HFNC, reduced the need for invasive mechanical ventilation, the rate of bacterial superinfections and that of in-hospital mortality.

**Compliance with ethics regulations:** Yes in clinical research. 
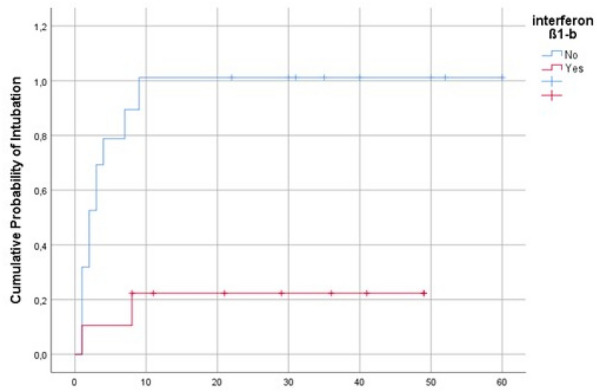


Cumulative incidence of intubation between Interferonβ-1b-treated and untreated patients. Log rank = 0.03

### CO-018 Dexamethasone does not increase the incidence of ventilator-associated pneumonia and bloodstream infections in COVID-19 patients requiring mechanical ventilation

#### Ines Gragueb Chatti^1,2^, Alexandre Lopez^3^, Dany Hamidi^4^, Christophe Guervilly^1,2^, Anderson Loundou^2^, Florence Daviet^1,2^, Nadim Cassir^5^, Laurent Papazian^1,2^, Jean-Marie Forel^1,2^, Marc Leone^3^, Jean Dellamonica^4^, Sami Hraiech^1,2^

##### ^1^Assistance Publique - Hôpitaux de Marseille, Hôpital Nord, Médecine Intensive Réanimation, Marseille, France; ^2^Centre d’Etudes et de Recherches sur les Services de Santé et qualité de vie EA 3279, Marseille, France; ^3^Aix-Marseille Université, Assistance Publique Hôpitaux de Marseille, Service d’Anesthésie et de Réanimation, Marseille, France; ^4^Service de Médecine Intensive Réanimation CHU de Nice et UR2CA Université Cote d’Azur, Nice, France; ^5^Institut Hospitalo-Universitaire Méditerranée Infection, Nice, France

**Correspondence:** Ines Gragueb Chatti - ines.gragueb-chatti@ap-hm.fr

*Annals of Intensive Care* 2021, **11(Suppl 1):**CO-018

**Rationale:** Dexamethasone decreases mortality in patients with severe coronavirus disease 2019 (COVID-19) and has become the standard of care during the second wave of pandemic. The role of dexamethasone in the high incidence of ventilator-associated pneumonia (VAP) and bloodstream infections (BSI) observed in intensive care units (ICUs) deserves to be investigated.

**Patients and methods/materials and methods:** An observational retrospective study in 3 French centers. Patients admitted from March to November 2020 for a documented COVID-19 and under mechanical ventilation for ≥ 48 h were included. The main outcomes were the incidence of VAP and BSI according to the use of dexamethasone. Secondary outcomes were day-28 and day-60 ventilator-free days (VFD), ICU and hospital length of stay and mortality.

**Results:** Among the 151 patients included, 84 were treated with dexamethasone. The incidences of VAP and BSI in patients treated or not with dexamethasone were, respectively, 63 vs. 57% (*p* = 0.43) and 29 vs. 30% (*p* = 0.86). The cumulative incidence of VAP, considering competing events, was similar in the 2 groups (*p* = 0.59). The first VAP occurred earlier in the dexamethasone group (*p* = 0.02). Patients receiving dexamethasone had more Gram-negative bacteraemia (*p* = 0.02). Mortality did not differ between groups. Patients receiving dexamethasone had more VFD at day 28 (*p* = 0.009) and a shorter ICU length of stay (*p* = 0.01).

**Conclusion:** In this cohort of COVID-19 patients requiring invasive MV, dexamethasone did not increase the incidence of VAP or BSI. Dexamethasone might not explain the high rates of VAP and BSI observed in critically ill COVID-19 patients.

**Compliance with ethics regulations:** Yes in clinical research. 
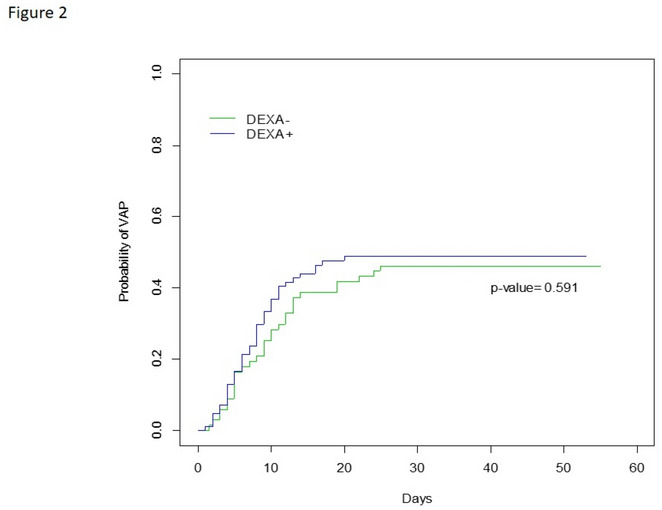


Probability of ventilator-associated pneumonia according to treatment with dexamethasone

### CO-019 Pulmonary and non-pulmonary sepsis differentially modulate lung immunity towards secondary bacterial pneumonia

#### Jean-François Llitjos^1,3^, Edwige Péju^2,3^, Cédric Auffray^4^, Christophe Rousseau^3^, Clémence Martin^3,5^, Pierre-Régis Burgel^3,5^, Jean-Paul Mira^2,3^, Jean-Daniel Chiche^2,3^, Maha-Zohra Ladjemi^3^, Bruno Lucas^4^, Frédéric Pène^2,3^

##### ^1^Institut Gustave Roussy, Villejuif, France; ^2^Service de Médecine Intensive et Réanimation, AP-HP, Hôpital Cochin, Paris, France; ^3^Institut Cochin, INSERM U1016, CNRS UMR 8104, Paris, France; ^4^Paris Descartes Université, Sorbonne Paris Cité, Institut Cochin, CNRS UMR8104, INSERM U1016, Paris, France; ^5^Hôpital Cochin, Respiratory Medicine and Cystic Fibrosis National Reference Center Service de Pneumologie, AP-HP, Paris, France

**Correspondence:** Jean-François Llitjos - jllitjos@gmail.com

*Annals of Intensive Care* 2021, **11(Suppl 1):**CO-019

**Rationale:** Septic shock patients with pneumonia exhibit a high risk of ICU-acquired pneumonia, suggesting that a primary pulmonary insult may drive profound alterations in lung defence towards secondary infections (1). The objective of this experimental study is to address the impact of primary pulmonary or non-pulmonary infectious insults on lung immunity.

**Patients and methods/materials and methods:** C57BL/6J mice were first subjected either to polymicrobial peritonitis induced by caecal ligation and puncture (CLP), or to bacterial pneumonia induced by intra-tracheal (i.t.) instillation of *Escherichia coli*. Respective control mice were subjected to sham surgery or intra-tracheal instillation of phosphate-buffered saline. Seven days later, mice that survived the primary insult were subjected to i.t. instillation of *Pseudomonas aeruginosa* (PAO1 strain). We assessed survival and pulmonary bacterial clearance of after *P. aeruginosa* pneumonia, as well as quantitative and functional changes in lung immune cells.

**Results:** When compared to sham-operated mice, post-CLP animals exhibited increased susceptibility to secondary *P. aeruginosa* pneumonia as demonstrated by defective lung bacterial clearance and increased mortality rate (50% vs. 0%, *p* < 0.05). In contrast, all post-pneumonia mice survived and even exhibited improved bacterial clearance as compared to their control counterparts. When addressing whole-lung immune cell distribution prior to second hit (day 7), amounts of alveolar macrophages (AM) were decreased in post-CLP mice while increased in post-pneumonia mice. In contrast to post-CLP, AM from post-pneumonia mice expressed high MHC-II, a defence-ready transcriptomic signature and promoted antigen-specific CD4 T cells proliferation. Additionally, we observed a TLR2-dependent increase in regulatory T cells (Tregs) proportion among CD4 T cells in the lung of post-CLP mice at day 7 when compared to controls and post-pneumonia mice. CD25-mediated depletion of Tregs prior to *P. aeruginosa* pneumonia restored survival and bacterial clearance in post-CLP mice. Tregs depletion was associated with restoration of numbers and functions of AM in post-CLP mice.

**Conclusion:** Polymicrobial peritonitis and bacterial pneumonia, respectively, confer susceptibility or resistance to secondary Gram-negative pneumonia. Pulmonary immune patterns in post-CLP mice suggest a TLR2-dependent cross-talk between T-regs and AM.

**Reference**

1. Llitjos JF et al. Ann Intensive Care 2019.

**Compliance with ethics regulations:** Yes in animal testing.

### CO-020 GLP1 is associated with mortality in the intensive care unit: a substudy of the French IVOIRE cohort

#### Marine Jacquier^1^, Annabelle Tavernier^4^, Eléa Ksiazek^1^, Isabelle Fournel^1^, Jacques Grober^1^, Jean-Pierre Quenot^1,2,3,4^

##### ^1^CHU Dijon, Dijon, France; ^2^INSERM, U1231, Equipe Lipness, Dijon, France; ^3^Université Bourgogne-Franche-Comté, UMR1231 Lipides, Nutrition, Cancer, Dijon, France; ^4^LipSTIC LabEx, Fondation de coopération scientifique Bourgogne-Franche-Comté, Dijon, France

**Correspondence:** Jean-Pierre Quenot - jean-pierre.quenot@chu-dijon.fr

*Annals of Intensive Care* 2021, **11(Suppl 1):**CO-020

**Rationale:** Septic shock remains a major cause of mortality and early detection is important in emergency services and the intensive care unit (ICU). GLP1 is a hormone released from enteroendocrine L cells and could be a prognostic biomarker in sepsis. Indeed, some studies have shown release of GLP1 in response to IL-6 (1). LPS injection or gut barrier injury causes GLP1 count to increase in human studies, and other studies have shown increased GLP1 in intestinal ischemia (2).

**Patients and methods/materials and methods:** Among 1294 patients included in the multicenter, observational IVOIRE cohort between June 2013 and January 2016, GLP-1 concentrations were determined in 556 patients (49 healthy volunteers and 507 patients from intensive care unit) (43%) by ELISA (ALPCO, Salem, NH). Distribution of GLP1 level was compared using the Kruskal–Wallis test between healthy volunteers, patients classified as no systemic inflammatory response syndrome (SIRS), SIRS, severe sepsis and septic shock. In patients, GLP1 effects on mortality at 90 days were analyzed by univariate and multivariable logistic models adjusted for sex, age, BMI, Charlson index and SOFA score.

**Results:** GLP1 was assessed in 49 healthy volunteers and 507 patients, of whom 296 had septic shock. In the patient group, mean SOFA score was 9.3 ± 3.9. Mean GLP1 concentration was 4.94 ± 4.19 ng/mL in healthy volunteers and 16.93 ± 20.15 ng/mL in the sepsis group. In the septic shock group, mean GLP1 was 18.94 ± 21.78 ng/mL (*p* < 0.01 across groups). By quartiles of GLP1 concentration at day 1, mortality increased with increasing GLP1 (from quartile 1 to 4 (*p* < 0.001)) (Fig. 1). By multivariable analysis, GLP1 level was significantly associated with 90-day mortality: an increase of one unit of GLP1 was associated with an adjusted-OR of 1.017 (95%CI [1.005;1.029], *p* < 0.01).

**Conclusion:** Elevated GLP1 concentrations are observed in sepsis patients, particularly those with septic shock. GLP1 could be a prognostic biomarker for patients hospitalized in ICU. However, this finding requires confirmation in further studies.

**References**

1. Ellingsgaard H et al. Nat Med. 2011;17(11):1481–9. 10.1038/nm.2513.
2. Lebrun LJ et al. Cell Rep. 2017;21(5):1160–8. 10.1016/j.celrep.2017.10.008.

**Compliance with ethics regulations:** Yes in clinical research. 
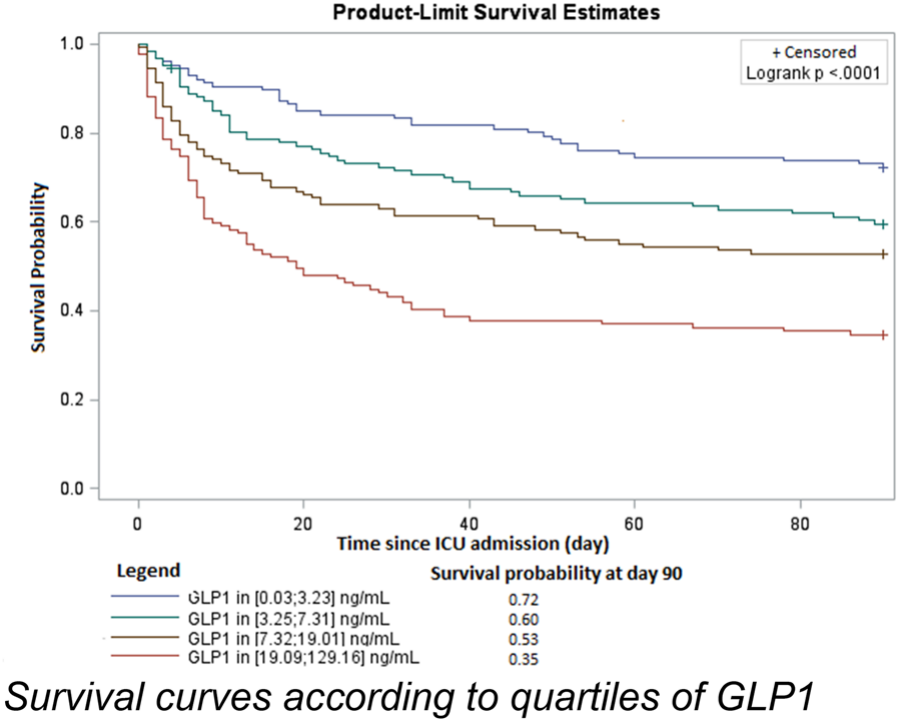


Survival curves according to quartiles of GLP1

### CO-021 Interest of procalcitonin-guided antibiotic therapy in mechanically ventilated comatose patients

#### Guylaine Labro^2^, François Aptel^1^, Marc Puyraveau^2^, Sébastien Pily Floury^2^, Jonathan Paillot^2^, Hamid Merdji^3^, Ferhat Meziani^3^, Gaël Piton^2^, Khaldoun Kuteifan^4^, Jean-Pierre Quenot^1^, Gilles Capellier^2^

##### ^1^CHU Dijon, Dijon, France; ^2^CHU de Besançon, Besançon, France; ^3^Hôpitaux Universitaires de Strasbourg, Strasbourg, France; ^4^Centre Hospitalier de Mulhouse, Mulhouse, France

**Correspondence:** Jean-Pierre Quenot - jean-pierre.quenot@chu-dijon.fr

*Annals of Intensive Care* 2021, **11(Suppl 1):**CO-021

**Rationale:** In comatose patients receiving oro-tracheal intubation for mechanical ventilation (MV), the risk of aspiration is increased. Aspiration can lead to chemical pneumonitis (inflammatory reaction to gastric content) or aspiration pneumonia (infection caused by aspiration of micro-organisms). Distinguishing between the two types is challenging. We tested the interest of using a decisional algorithm based on procalcitonin (PCT) values to guide initiation and discontinuation of antibiotic therapies in intubated patients.

**Patients and methods/materials and methods:** The PROPASPI (PROcalcitonin Pneumonia/pneumonitis Associated with ASPIration) trial is a multicentre, prospective, randomized, controlled, single-blind, superiority study comparing two strategies: (1) an intervention group where threshold PCT values were used to guide initiation and discontinuation of antibiotics (PCT group); and (2) a control group where antibiotic therapy was managed at the discretion of the physician (aspiration pneumonia should be suspected in the presence of clinical, biological (apart from PCT) and/or radiological criteria). Patients aged 18 years or over, intubated for coma (Glasgow score ≤ 8), with MV initiated within 48 h after admission were eligible. The primary endpoint was the duration of antibiotic treatment during the first 15 days after admission to the ICU.

**Results:** From 24/2/2015 to 28/8/2019, 1721 patients were intubated for coma in the 5 participating centres, of whom 166 were included in the study. Data from 159 were available for intention-to-treat analysis: 81 in the PCT group, and 78 in the control group. Overall, 67 patients (43%) received antibiotics in the intensive care unit (ICU); there was no significant difference between groups (37 (46%) vs 30 (40%) for PCT vs control, *p* = 0.432). The mean duration of antibiotic treatment during the first 15 days in the ICU was 2.7 ± 3.8 days; there was no significant difference between groups (3.0 ± 4.1 days vs 2.3 ± 3.4 days for PCT vs control, *p* = 0.311). The mean number of days under MV was significantly higher in the PCT group (3.7 ± 3.6 days) than in controls (2.7 ± 2.5 days, *p* = 0.033). The duration of ICU stay was also significantly longer in the PCT group: 6.4 ± 6.5 days v 4.6 ± 3.5 days in the control group (*p* = 0.043).

**Conclusion:** The use of PCT values to guide therapy, in comparison to the use of clinical, biological (apart from PCT) and radiological criteria, does not modify exposure to antibiotics in patients intubated for coma.

**Compliance with ethics regulations:** Yes in clinical research

### CO-022 A prospective multicenter observational study of insertion and use of central lines in the intensive care unit

#### Agnès Petiteau, Florent Goube, Mathilde Farizon, Sylvie Baune, Nathalie Van Der Mee-Marquet, For The Spiadi Icu Group

##### CHRU, Tours, France

**Correspondence:** Nathalie Van Der Mee-Marquet - n.vandermee@chu-tours.fr

*Annals of Intensive Care* 2021, **11(Suppl 1):**CO-022

**Rationale:** In 2020, a nationwide 3-month survey conducted in 207 ICUs showed median incidence rates of central line-associated bloodstream infections (CLABSI) ranging from 0 to 0.97/1000 catheter-days, according to device and hospital type (Fig. 1). 251 CLABSIs were identified, 56.6% involving a central venous catheter (CVC), 35.9% an arterial catheter (AC) and 7.5% a dialysis catheter (DC); 54.1% were associated with a *Staphylococcus*, including one-third with *S. aureus*. The median time lag between catheter insertion and appearance of the clinical signs of BSI was 10 days. CLABSIs are associated with high morbidity and mortality in ICUs. Their prevention is a priority. Our study was carried out to detect the differences between field practices and the national guidelines for the management of central lines.

**Patients and methods/materials and methods:** Infection control teams conducted direct observation of insertion and use of central lines in ICUs. Using a standardized questionnaire, they documented patient and professional dress (gown, mask, cap, gloves), hand hygiene before catheter insertion or use, skin cleansing and disinfection (antiseptic used, spontaneous drying) and pots disinfection. The collected data were analyzed at national level.

**Results:** 33 ICUs took part in the study (4 university/regional hospitals, 3 military hospitals, 17 general hospitals, 9 private clinics). 245 observations were conducted: 79 insertions (50 CVC, 22 AC, 7 DC), 67 proximal manipulations (44 CVC, 12 CA, 11 DC), 99 distal manipulations (88 CVC, 4 AC, 7 DC). The results are presented on Table 1. Dressing was in accordance with guidelines, except for sterile gown which lacked for 10% of catheter insertions. By contrast, cap and mask for non-intubated patients were frequently lacking. Disinfection was preferentially performed with povidone-iodine/alcohol (69.2% for insertion, 60.9% for proximal manipulations, 46.9% for distal manipulations), antiseptic spontaneous drying lacked in 10% of cases, and valve disinfection before use lacked or was inadequate in > 50% of cases. Regarding hand hygiene and gloves issues, rubbing was predominant, but hand hygiene lacked or was not performed correctly in 21.5% of cases before sterile gloving, nor in 17.9% and 36.4% of the cases before proximal and distal manipulations, respectively. Steriles gloves were missed for 25% of proximal manipulations.

**Conclusion:** Our data indicate several issues that may result in catheter contamination by bacteria from the skin flora, and that should be improved. We believe our observation tool may help to raise the ICU staff’s awareness of the risk to contaminate catheter and provide a baseline to drive a quality improvement strategy.

**Compliance with ethics regulations:** Yes in clinical research.
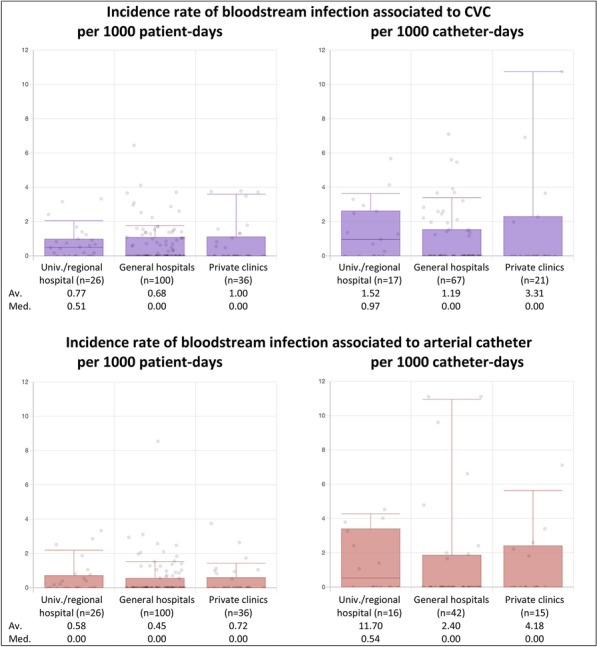


Fig. 1 Incidence rates of central venous catheter- and arterial catheter-related bloodstream infections according to the type of hospital (data from the nationwide prospective SPIADI survey, 2020).
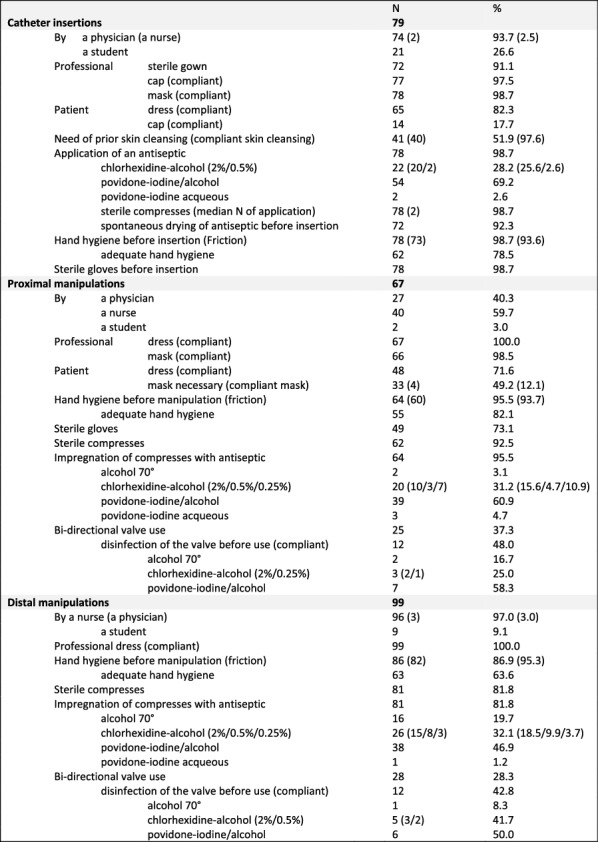


Table 1 Major results from the 245 practice observations

### CO-023 Menstrual toxic shock syndrome in the ICU: a French nationwide multicenter retrospective study

#### Damien Contou^1^, Gwenhaël Colin^24^, Brendan Travert^3^, Sébastien Jochmans^4^, Marie Conrad^5^, Jean-Baptiste Lascarrou^3^, Benoit Painvin^6^, Alexis Ferré^7^, David Schnell^8^, Béatrice La Combe^9^, Rémi Coudroy^10^, Stephan Ehrmann^11^, Jérôme Rambaud^13^, Arnaud Wiedemann^14^, Pierre Asfar^15^, Pierre Kalfon^16^, Emmanuel Guérot^17^, Sébastien Préau^18^, Laurent Argaud^12^, Florence Daviet^19^, Jean Dellamonica^20^, Audrey Dupont^20^, Muriel Fartoukh^21^, Toufik Kamel^22^, Gaëtan Béduneau^23^, Emmanuelle Boutin^2^, Gérard Lina^12^, Armand Mekontso Dessap^2^, Anne Tristan^12^, Nicolas De Prost^2^

##### ^1^Centre Hospitalier Victor Dupouy, Argenteuil, France; ^2^Hôpital Henri Mondor, Créteil, France; ^3^Centre Hospitalier Universitaire de Nantes, Nantes, France; ^4^Centre Hospitalier de Melun, Melun, France; ^5^Centre Hospitalier Universitaire de Nancy, Nancy, France; ^6^Centre Hospitalier Universitaire de Rennes, Rennes, France; ^7^Centre Hospitalier Le Chesnay, Versailles, France; ^8^Centre Hospitalier d’Angoulême, Angoulême, France; ^9^Hôpital du Scorff - Groupe Hospitalier Bretagne Sud Lorient, Lorient, France; ^10^Centre Hospitalier Universitaire de Poitiers, Poitiers, France; ^11^Centre Hospitalier Universitaire Tours, Tours, France; ^12^Centre Hospitalier Universitaire de Lyon, Lyon, France; ^13^Centre Hospitalier Universitaire Trousseau - APHP, Paris, France; ^14^Centre Hospitalier Universitaire de Nancy, Nancy, France; ^15^Centre Hospitalier Universitaire d’Angers, Angers, France; ^16^Centre Hospitalier Universitaire de Chartres, Le Coudray, France; ^17^Centre Hospitalier Universitaire HEGP - APHP, Paris, France; ^18^Centre Hospitalier Universitaire de Lille, Lille, France; ^19^Centre Hospitalier Universitaire de Marseille - Hôpital Nord APHM, Marseille, France; ^20^Centre Hospitalier Universitaire de Nice, Nice, France; ^21^Centre Hospitalier Universitaire Tenon - APHP, Paris, France; ^22^Centre Hospitalier d’Orléans, Orléans, France; ^23^Centre Hospitalier Universitaire de Rouen, Rouen, France; ^24^Centre Hospitalier Départemental de Vendée, La-Roche-Sur-Yon, France

**Correspondence:** Damien Contou - damien.contou@ch-argenteuil.fr

*Annals of Intensive Care* 2021, **11(Suppl 1):**CO-023

**Rationale:** Studies describing the clinical features and the short-term prognosis as well as the organ failures of patients admitted to the intensive care unit (ICU) for menstrual toxic shock syndrome (m-TSS) are lacking. We aimed at (1) describing the clinical and microbiological features, including rate of super-antigenic toxin; (2) assessing the 2011 CDC diagnostic criteria upon ICU admission, as well as (3) reporting on the short-term prognosis of patients admitted to the ICU for an m-TSS.

**Patients and methods/materials and methods:** Multicenter retrospective cohort study including patients with a clinical diagnosis of m-TSS admitted between January 1st, 2005 and December 31st, 2020 in 43 French pediatric (*n* = 7) or adult (*n* = 36) ICUs. An m-TSS was defined by the association of an acute circulatory failure with fever (temperature > 38.3 °C) occurring in a context of menstruations (i.e., within 3 days of the beginning or end of menses) with use of tampon together with the absence of other source of sepsis. The 2011 CDC diagnostic criteria [1] were not used for the definition and inclusion of a patient with a suspected m-TSS at ICU admission. Factor Analysis for Mixed Data (FAMD) was used in an attempt to identify different clinical phenotypes of m-TSS.

**Results:** From 2005 to 2020, 102 patients with m-TSS (median age: 18 [16–24] years; no previous comorbidity: *n* = 91/102 (89%)) were admitted to one of the participating ICUs. All blood cultures (*n* = 102) were sterile. Ninety-two of the 96 (96%) vaginal samples obtained grew methicillin-sensitive *Staphylococcus aureus*. Screening for super-antigenic toxin gene sequences was performed in 76 of the 92 (83%) vaginal samples growing *Staphylococcus aureus* and isolated TSST-1 in 66 (87%) strains or a staphylococcal enterotoxin in 41 (54%). Upon ICU admission, no patient met the 2011 CDC criteria for a confirmed m-TSS while only 53 (52%) fulfilled the criteria for a probable m-TSS. FAMD analysis did not isolate a specific pattern of variables highlighting the homogeneity of the patients. Eighty-one patients (79%) were treated with anti-toxinic antibiotic therapy and eight patients (8%) received intravenous immunoglobulins. Eighty-six (84%) and 21 (21%) patients needed vasopressors and tracheal intubation, respectively. No patient required limb amputation or died in the ICU.

**Conclusion:** In this large multicenter series of patients included in ICUs for m-TSS, we show that despite high rates of vasopressor and tracheal intubation, none of them died or required limb amputation. The CDC criteria should not be used for the clinical diagnosis upon ICU admission.

**References**

1. Toxic Shock Syndrome (Other Than Streptococcal)| 2011 Case Definition./nndss/conditions/toxic-shock-syndrome-other-than-streptococcal/case-definition/2011/.
2. Berger S, Kunerl A, Wasmuth S, et al. Menstrual toxic shock syndrome: case report and systematic review of the literature. Lancet Infect Dis. 2019; 19:e313–21. 10.1016/S1473-3099(19)30041-6.

**Compliance with ethics regulations:** Yes in clinical research.
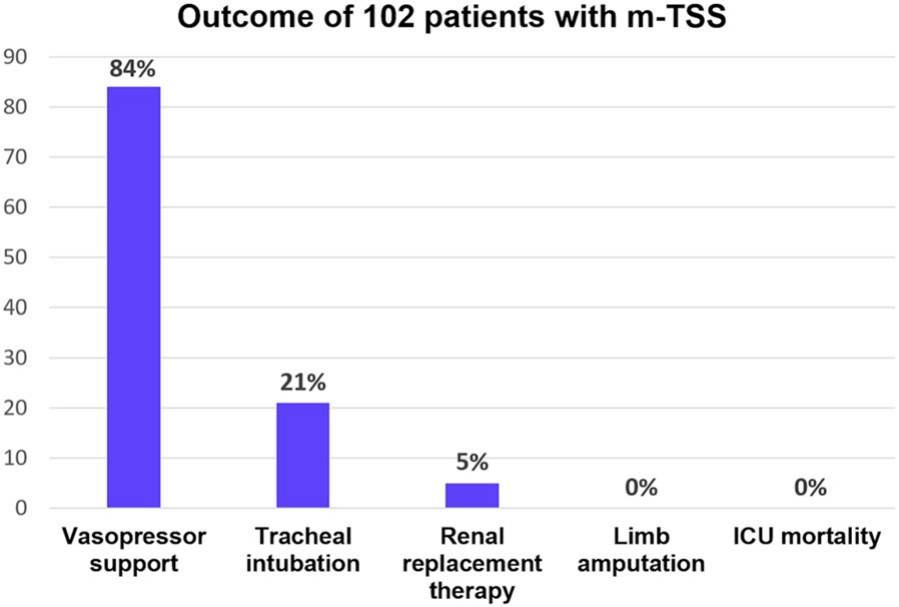


Outcome of 102 patients with menstrual toxic shock syndrome (m-TSS) in the intensive care unit (ICU)

### CO-024 Lower respiratory tract infection with *Staphylococcus aureus* in severe acute chest syndrome in adult sickle-cell patients

#### Alexandre Elabbadi^1^, Guillaume Voiriot^1^, Anne Tristan^2^, Aude Gibelin^1^, Charlotte Verdet^3^, Michel Djibré^1^, Aline Santin^1^, Étienne-Marie Jutant^1^, Julien Lopinto^1^, François Vandenesch^2^, François Lionnet^4^, Muriel Fartoukh^1^

##### ^1^Assistance Publique - Hôpitaux de Paris, Service de Médecine Intensive Réanimation, Hôpital Tenon, Paris, France; ^2^Hospices Civils de Lyon, Centre de biologie Est, Laboratoire de bactériologie, Lyon, France, Lyon, France; ^3^Assistance Publique - Hôpitaux de Paris, Service de Bactériologie, Hôpital Saint-Antoine, Paris, France; ^4^Assistance Publique - Hôpitaux de Paris, Service de Médecine Interne, Hôpital Tenon, Paris, France

**Correspondence:** Alexandre Elabbadi - alexandre.elabbadi@aphp.fr

*Annals of Intensive Care* 2021, **11(Suppl 1):**CO-024

**Rationale:** Acute lower respiratory tract infection is a major contributing factor of acute chest syndrome, one of the most severe complications of sickle cell disease. Pathophysiology of acute chest syndrome is complex and multifactorial. However, there are three main and often interlinked causes: fat embolism, pulmonary vascular thrombosis and lung infection. Lung infection may account for 30% of the causes of acute chest syndrome. The causal relationship between *Staphylococcus aureus* and acute chest syndrome has been poorly assessed.

**Patients and methods/materials and methods:** We conducted a monocentric retrospective study of sickle cell disease adult patients with acute chest syndrome and documented *S. aureus* lower respiratory tract infection (*S. aureus* group) admitted to the intensive care unit from 2015 to 2017, compared to acute chest syndrome patients in whom either another microorganism or no microorganism was identified despite a complete microbiological investigation (control group) during the same period. *S. aureus* strains were sent to the National Staphylococcus Reference Center for genotypic analysis.

**Results:** During the study period, 119 episodes of acute chest syndrome (106 patients) were recorded, among which 29 (24%) were associated with *S. aureus*, including two methicillin-resistant strains. A history of *S. aureus* infection was more common in the *S. aureus* group. At least one toxin gene was found in 13 *S. aureus* isolates (62%), 2 of which (10%) had the genes coding for Panton–Valentine leucocidin. Short-term outcomes did not differ between the two groups. Higher number of readmissions for an acute vaso-occlusive event and shorter time to hospital readmission were marginally observed in the *S. aureus* group.

**Conclusion:** A high proportion of acute chest syndrome episodes were microbiologically documented with *S. aureus*, harboring a wide clonal diversity. A history of *S. aureus* infection was associated with the identification of *S. aureus* during the index episode of acute chest syndrome. Short-term outcomes did not differ between the two groups.

**Compliance with ethics regulations:** Yes in clinical research.

### CO-025 Characteristics and outcomes of undocumented migrants admitted in French intensive care units from 2011 to 2018

#### Sami Hraiech^1^, Vanessa Pauly^1^, Veronica Orleans^1^, Laurent Papazian^1^, Elie Azoulay^2^

##### ^1^APHM, Marseille, France; ^2^APHP, Paris, France

**Correspondence:** Sami Hraiech - sami.hraiech@ap-hm.fr

*Annals of Intensive Care* 2021, **11(Suppl 1):**CO-025

**Rationale:** A growing number of people immigrate illegally in France for economic or political reasons. Migrants’ health can be endangered by their journey and their living conditions in destination country. The question of severe illness and intensive care unit (ICU) admissions in this precarious population has been poorly studied.

**Patients and methods/materials and methods:** We performed a nationwide-based cohort study using the French national hospital database to identify adult migrants patients admitted to the ICU during a 8-year period. Migrants and general population were compared according to demographic characteristics, comorbidities, reason for ICU admission, invasive supportive therapies, mortality, duration of mechanical ventilation, ICU and hospital stay and costs. We performed a crude analysis on the whole population then a matched-controlled study.

**Results:** From 2011 to 2018, we identified 14 554 migrants ICU stays, this number doubling across the study period. Migrants were younger (47 ± 17 vs. 65 ± 17 years old) and more severe (SAPS II without age 20 ± 8 vs. 17 ± 17) than the general ICU population. Cardiovascular diseases and post-operative care were the 2 main reasons for ICU admission in both groups. Trauma and violence (3rd cause, 10.5% of admissions), infections (4th cause, 9.3%) and obstetrical complications (10th cause, 4.3%) were more frequent among migrants. Migrants more frequently needed invasive procedures but also more palliative care. In the crude analysis, migrants had lower ICU and hospital mortality than the general population (6 vs 8.5% and 7.1 vs. 10.5%, respectively) but longer ICU and hospital stays, resulting in higher costs. In the matched population analysis, ICU and hospital mortality among migrants and general population did not differ (5.6% vs. 5.7%; *p* = 0.66; 6.7% vs. 6.6%; *p* = 0.69).

**Conclusion:** Admissions of migrants in French ICUs increase faster than for general population. Trauma, violence, infections and obstetrical complications-related admissions call attention about the need of improving migrants access to primary care and conditions of living.

**Compliance with ethics regulations:** Yes in clinical research.

### CO-026 Children may be impacted by adult hospitalizations in ICUs

#### Caroline Hauw-Berlemont, Benoît Champigneulle, Aurore Imbert, Cindy Davagnar, Florence Bellenfant, Isabelle Caminade, Ana Novara, Alexandra Monnier, Nadia Aissaoui, Jean-Luc Diehl, Emmanuel Guerot

##### Hôpital Européen Georges Pompidou, Paris, France

**Correspondence:** Caroline Hauw-Berlemont - caroline.hauw-berlemont@aphp.fr

*Annals of Intensive Care* 2021, **11(Suppl 1):**CO-026

**Rationale:** Policies regarding children’s visits remain restrictive in many adult intensive care units (ICUs). Children are not considered as family members in recommendations (1), and nothing is known about the number of children who could be concerned by adult relative hospitalizations in ICUs (2) and how many of them would like to come to visit their relative. We aimed to assess the number of children who may be concerned by adult hospitalizations.

**Patients and methods/materials and methods:** In this prospective study, we surveyed 100 consecutive families of patients hospitalized in two ICUs of a French teaching hospital during 3 months. An adult within each family was surveyed: the main objective was to evaluate the number of children concerned by adult hospitalizations in ICUs. The secondary objectives were (1) to objectify families’ difficulties regarding the communication with those children about the patient’s life-threatening condition, using a visual analog scale; (2) to understand if the adults told their children about serious conditions of the patients; (3) to evaluate the number of children who wanted to visit the hospitalized adults.

**Results:** Among one hundred collected surveys, 39% of patients had at least one child in their close family, with a total of 64 children who could have been impacted by these adult hospitalizations. The children were 8.5 (± 4.3) years old on average. In 87.2%, patients/families claimed some difficulties to explain the situation to the child. On a visual analog scale, the mean score of difficulty to communicate with children was 4/10. In less than 30%, the child was aware of the hospitalization and its severity; in the majority of cases (59.4%), she/he was aware of the hospitalization but not of its severity. Fifty-five percent of children wanted to come to visit while only 23.4% actually came. Fifty-one percent of families claimed the need for help to handle the situation with their children (Table 1).

**Conclusion:** This study showed that many children may be impacted by adult hospitalizations in ICUs, that a majority of concerned children wanted to come to visit and that a large majority of families explained some difficulties to communicate with the children regarding a serious life-threatening condition. These data support the need of larger studies to evaluate children’s expectations and needs by surveying them directly and the potential consequences of children visitations on children themselves but also on patients, families, and providers, to make our ICUs policies more flexible.

**Reference**

1. Davidson JE, Aslakson RA, Long AC, Puntillo KA, Kross EK, Hart J, Cox CE, Wunsch H, Wickline MA, Nunnally ME, Netzer G, Kentish-Barnes N, Sprung CL, Hartog CS, Coombs M, Gerritsen RT, Hopkins RO, Franck LS, Skrobik Y, Kon AA, Scruth EA, Harvey MA, Lewi.
2. Laurent A, Leclerc P, Nguyen S, Capellier G. The effect visiting relatives in the adult ICU has on children. Intensive Care Med. 2019;45(10):1490–2.

**Compliance with ethics regulations:** Yes in clinical research.
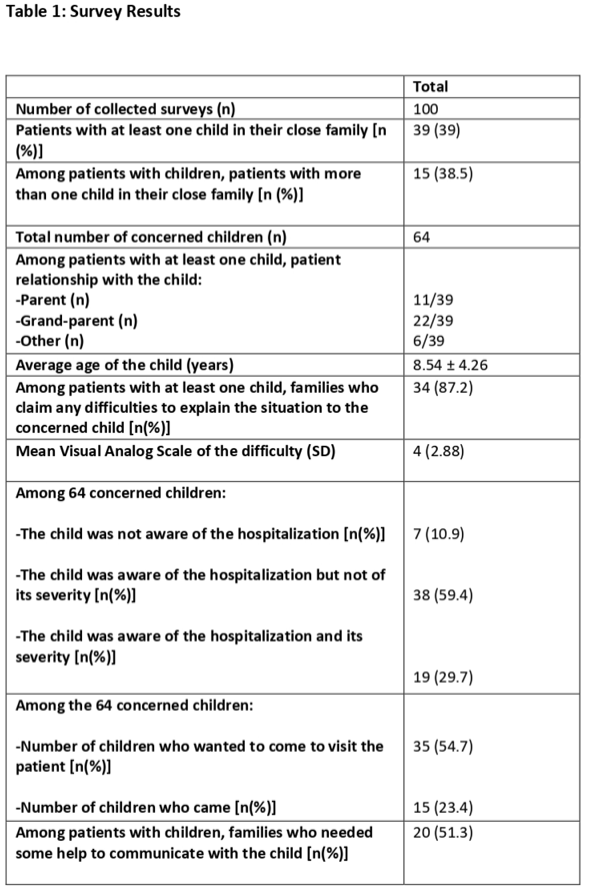


Table 1 Survey results

### CO-027 Characteristics and determinants of ICU admission decision during the COVID-19 crisis: a prospective multicenter study

#### Souphone Mongkhoun^1^, Pascal Beuret^2^, Vincent Gauthier^5^, Laurent Gergelet^6^, Jean-Paul Chaussinand^4^, Véronique Porot^3^, Jérôme Morel^1^, Guillaume Thiery^1^

##### ^1^CHU de St Etienne, St Priest En Jarez, France; ^2^Centre Hospitalier de Roanne, Roanne, France; ^3^Centre Hospitalier Nord Ardèche, Annonay, France; ^4^Centre Hospitalier du Forez, Montbrison, France; ^5^Clinique Mutualiste, St Etienne, France; ^6^Hôpital Privé de la Loire, St Etienne, France

**Correspondence:** Guillaume Thiery - guillaume.thiery@chu-st-etienne.fr

*Annals of Intensive Care* 2021, **11(Suppl 1):**CO-027

**Rationale:** The surge of patients with severe COVID-19 requiring ventilator support has led ICU (intensive care unit) physicians to triage ICU admission in a context of limited ICU capacity. Thus, we aimed to evaluate factors that influenced ICU admission decisions of critically ill COVID-19 patients.

**Patients and methods/materials and methods:** This multicenter prospective study was conducted in 6 hospitals (1 University Hospital, 3 public general hospitals and 2 private hospitals) in March and April 2020, during the first wave of COVID-19 epidemics. All patients for whom ICU admission was requested were included. For each admission proposal, the triaging intensivist completed a questionnaire and the following data were collected: reason for ICU proposal, demographic characteristics, comorbidities, Instrumental Activity of Daily Living (IADL) score, Clinical Frailty Score (CFS) (1). Frailty was defined as CFS ≥ 5. Clinicians were asked which factors influenced their decision.

**Results:** During the study period, 127 patients were proposed for ICU admission because of acute respiratory failure. Median age was 71 [64–78] years. Main comorbidities were hypertension (64%), obesity (36%), diabetes (30%). CFS ≥ 5 was found in 17% of patients. Eighty-eight percent of decisions were taken with another physician. The attending physician was involved in 67% of decisions, another intensivist was also involved within in 30%. In 44% of cases, the decision was anticipated and taken before the patient’s condition worsened. Only 6.8% of patients had known advanced directories. The decision was said to be very difficult in 19% of cases, not very difficult in 26% and easy in 55%. Thirty-one percent of patients were refused admission to the ICU. Among the 18 patients > 80 years old, 2 were admitted to the ICU. In multivariate analysis, factors associated with ICU admission were malnutrition (OR 0.04 [95%CI 0.00–0.29], *p* = 0.002) and CFS ≥ 5 (OR 0.03 [0.01; 0.13], *p* < 0.001). For each decision, the following factors were considered to be important in the decision-making process: age (80%), comorbidities (76%), dependency (83%), cognitive impairment (68%). Bed occupancy was of little importance in 77% of the decisions.

**Conclusion:** ICU triage is a major challenge since the beginning of COVID-19 crisis. In our study, 40% of the decisions were considered to be difficult, but decisions were most rarely taken alone. Advanced directories were almost never known. Clinical frailty score ≥ 5 and malnutrition were strongly associated with ICU refusal. Despite the surge of COVID-19 patients, bed occupancy rarely influenced the decision-making process.

**Reference**

1. Validation of the clinical frailty score (CFS) in French language. Abraham P, Courvoisier DS, Annweiler C, Lenoir C, Millien T, Dalmaz F, Flaatten H, Moreno R, Christensen S, de Lange DW, Guidet B, Bendjelid K, Walder B, Bollen Pinto B. BMC Geriatr. 2019.

**Compliance with ethics regulations:** Yes in clinical research.

### CO-028 Continuous traumatic stress in ICU during the pandemic period COVID-19: French multicentric study

#### Alexandra Laurent^1,2^, Alicia Fournier^1^, Florent Lheureux^3^, Anne-Laure Poujol^4^, Victoire Deltour^1^, Fiona Escarnot^5^, Nicolas Meunier^6^, Mélanie Loiseau^8^, Christine Binquet^1,6^, Jean-Pierre Quenot^1,8,9,10^

##### ^1^Université de Bourgogne, Dijon, France; ^2^Department of Anaesthesiology and Critical Care Medicine, Dijon University Medical Centre, Dijon, France; ^3^Laboratoire de Psychologie, Université de Bourgogne Franche-Comté, Besançon, France; ^4^Multidisciplinary Intensive Care Unit, Department of Anesthesiology and Critical Care, La Pitié-Salpêtrière Hospital, Assistance Publique-Hôpitaux de Paris, Sorbonne University, Paris, France; ^5^Department of Cardiology, University Hospital, Besançon, and EA3920, University of Burgundy-Franche-Comté, Besançon, France; ^6^Inserm CIC1432, module Epidémiologie Clinique (CIC-EC)- CHU Dijon-Bourgogne, UFR des Sciences de Santé, Dijon, France; ^7^Service de Médecine Légale CHU Dijon; Cellule d’Urgence Médico-Psychologique de Bourgogne Franche-Comté, Dijon, France; ^8^Service de Médecine Intensive-Réanimation, CHU Dijon-Bourgogne, Dijon, France; ^9^Equipe Lipness, centre de recherche INSERM UMR1231 et LabEx LipSTIC, Université de Bourgogne-Franche Comté, Dijon, France; ^10^Espace de Réflexion Éthique Bourgogne Franche-Comté (EREBFC), Dijon, France

**Correspondence:** Alexandra Laurent - alexandra.laurent@u-bourgogne.fr

*Annals of Intensive Care* 2021, **11(Suppl 1):**CO-028

**Rationale:** Intensive care health professionals are strongly affected by the COVID-19 epidemic and face multiple and intense stressors. The aims of this study were (1) to measure the prevalence of post-traumatic stress disorder (PTSD) in the healthcare professionals in intensive care units at 3 months after the peak of the March 2020 health crisis in France; (2) to identify the risk factors associated with traumatic disorders; and the support from which the professionals were able to benefit.

**Patients and methods/materials and methods:** This is a prospective study conducted in 77 French ICUs from 01/04/20 to 07/06/20. In phase 1, Sources of stress during the crisis were assessed using the Perceived Stressors in Intensive Care Units (PS-ICU) (Laurent et al. 2021). In phase 2, IESR scale and support questionnaire were added.

**Results:** Out of 2153 respondents (physicians, residents, nurses, nurses’ aides, students and nursing managers), 20.6% showed symptoms of PTSD, most of them reviviscences. All professional categories were affected. The stress factors associated with the risk of PTSD are workload and human resource issues, patient- and family-related emotional load, and items specific to COVID-19. The more stress caregivers perceived during the crisis, the more they turned to support from their colleagues, families and/or a psychologist. The telephone hotlines run by psychologists were very little used by professionals.

**Conclusion:** Many healthcare professionals in intensive care units suffer from a PTSD. The continuity of the stress factors experienced during the crisis led to the discussion of the notion of continuous traumatic stress and the need to set up adapted and long-term support systems for resuscitation workers.

**Reference**

1. Laurent A, Fournier A., Lheureux F, Cruz Martin Delgado M, Bocci MG, Prestifilippo A, Aslanian P., Henriques J., Paget-Bailly S., Constantin JM, Besch B, Quenot JP, Anota A, Bouhemad B., Capellier G. A new tool to measure stress factors in intensive care.

**Compliance with ethics regulations:** Yes in clinical research.

### CO-029 Case fatality inequalities of critically ill COVID-19 patients according to patient-, hospital- and region-related factors: a French nationwide study

#### Antoine Guillon^1^, Emeline Laurent^1^, Antoine Duclos^2^, Lucile Godillon^1^, Pierre-François Dequin^1^, Nelly Agrinier^3^, Antoine Kimmoun^3^, Leslie Grammatico-Guillon^1^

##### ^1^CHRU Tours, Tours, France; ^2^Hospices Civils de Lyon, Lyon, France; ^3^CHU Nancy, Nancy, France

**Correspondence:** Antoine Guillon - antoine.guillon@univ-tours.fr

*Annals of Intensive Care* 2021, **11(Suppl 1):**CO-029

**Rationale:** The COVID-19 sanitary crisis inflicted different challenges regarding the reorganization of the human and logistic resources, particularly in intensive care unit (ICU). Interdependence between regional pandemic burden and individual outcome remains unknown. The study aimed to assess the association between ICU bed occupancy and case fatality rate of critically ill COVID-19 patients.

**Patients and methods/materials and methods:** A cross-sectional study was performed in France, using the national hospital discharge databases from March to May, 2020. All patients admitted in ICU for COVID-19 were included. Case fatality was described according to: (i) patient’s characteristics (age, sex, comorbid condition, ICU interventions); (ii) hospital’s characteristics (baseline ICU experience assessed by the number of ICU stays in 2019, number of ICU physicians per bed), and (iii) the regional outbreak-related profiles (workload indicator based on ICU bed occupancy). The determinants of lethal outcome were identified using a logistic regression model.

**Results:** 14,513 COVID-19 patients were admitted in ICU; 4,256 died (29.3%), with important regional inequalities in case fatality (from 17.6% to 33.5%). Older age, multimorbidity and clinical severity were associated with higher mortality, as well as a lower baseline ICU experience of the health structure. Regions with more than 10 with ≥ 75% of ICU occupancy by COVID-19 experienced an excess of mortality (up to adjusted OR = 2.2 [1.9–2.6] for region with the highest occupancy rate of ICU beds).

**Conclusion:** The COVID-19 sanitary crisis has led to up to 2.2-fold increase of adjusted death rate in region experiencing the highest burden of care in ICU.

**Compliance with ethics regulations:** N/A.

### CO-030 The experience of two French temporary weaning centers created in order to tackle the first wave of Covid-19 pandemic effect on ICU saturation

#### Laura Federici^1^, Constance Vuillard^1^, Pierre-Julien Cungi^2^, Matthieu Dubois^1^, Philippe Goutorbe^2^, Jean-Damien Ricard^1^, Valeria Velea^1^, Eric Meaudre^2^, Damien Roux^1^

##### ^1^CHU Louis Mourier, Colombes, France; ^2^Hôpital d’Instruction des armées SAINTE ANNE, Toulon, France

**Correspondence:** Laura Federici - laura.federici85@gmail.com

*Annals of Intensive Care* 2021, **11(Suppl 1):**CO-030

**Rationale:** During the first wave of the Covid-19 pandemic, intensive care units (ICU) were quickly saturated. Two weaning centers (WC) were created to free ICU beds. Medical and nursing staffs were unexperienced for care of critically ill patients. An intensivist was available 24/7 in case of patient deterioration. The present study described patients admitted to the WC and their outcome.

**Patients and methods/materials and methods:** Retrospective study in two units. The population was adult patients admitted to ICU between March 17 and April 3, 2020 for Covid-19-related acute respiratory distress syndrome (ARDS). Patients were transferred to the WC if mandatory pre-specified criteria were all present: tracheotomy for > 24 h, respiratory failure (with FiO2 <=50% and PEP <=10) with no other organ failure and ventilation with a home ventilator for > 12 h, RASS between 0 and − 1, and no neuromuscular blockers or prone positioning for > 72 h. Participants were informed of the research purpose and their right to decline participation. Patients characteristics and data were collected.

**Results:** Fifteen patients were included: 5 women and 10 men. The median age was of 67[49–79] years and median IGSII of 60[24–77]. The most frequent comorbidity was hypertension (9 patients). Median time between first COVID-19 symptoms and ICU admission was 7 days [3–17]. All patients received mechanical ventilation in whom 12 during the first 24 h. Based on the Berlin definition, severe ARDS was reported in five patients. Neuromuscular blockers and prone positioning were used in 14 and 10 patients, respectively. One patient was placed on extracorporeal membrane oxygenation. The tracheotomy was performed after a median of 17 days post-intubation. The median length of ICU stay before transfer was 24 days. In the WC, 12 patients were weaned and 10 decannulated during their stay. Median total duration (ICU + WC) of mechanical ventilation was 27 days. Six patients walked and 13 ate orally by the end of their WC stay. The median length of WC stay was 16 days. Two serious complications required ICU readmission (1 bleeding, 1 cannula obstruction). Ventilator-associated pneumonia was diagnosed in 4 patients in the WC. Ninety days after ICU admission, 14 patients were alive and 12 returned home.

**Conclusion:** These two temporary WC allowed safe care of tracheotomized patients still requiring mechanical ventilation supply, with no other organ failure. This allowed ICU to accept new acute patients. This strategy is reliable when ICU beds are overwhelmed.

**Reference**

1. Jan Bauer ad al. Access to intensive care in 14 European countries: a spatial analysis of intensive care need and capacity in the light of COVID-19. Intensive Care Med. 2020; 46(11): 2026–34. 10.1007/s00134-020-06229-6.

**Compliance with ethics regulations:** Yes in clinical research.

### CO-031 Noninvasive ventilation vs. high-flow nasal cannula oxygen for preoxygenation before intubation in critically ill obese patients

#### Maeva Rodriguez^1^, Stéphanie Ragot^1^, Rémi Coudroy^1^, Jean-Pierre Quenot^2^, Philippe Vignon^3^, Jean-Marie Forel^4^, Alexandre Demoule^5^, Jean-Paul Mira^6^, Jean-Damien Ricard^7^, Saad Nseir^8^, Gwenhael Colin^22^, Bertrand Pons^9^, Pierre-Eric Danin^10^, Jérome Devaquet^11^, Gwenael Prat^12^, Hamid Merdji^13^, Franck Petitpas^1^, Emmanuel Vivier^14^, Armand Mekontso-Dessap^15^, Mai-Anh Nay^16^, Pierre Asfar^17^, Jean Dellamonica^10^, Laurent Argaud^18^, Stephan Ehrmann^19^, Muriel Fartoukh^20^, Christophe Girault^21^, René Robert^1^, Arnaud W. Thille^1^, Jean-Pierre Frat^1^

##### ^1^CHU Poitiers, Poitiers, France; ^2^CHU Dijon Bourgogne, Dijon, France; ^3^CHU Dupuytren, Limoges, France; ^4^AP-HM Hopital Nord, Marseille, France; ^5^AP-HP, Groupe Hospitalier Pitié-Salpêtrière Charles Foix, Paris, France; ^6^Assistance Publique des Hôpitaux de Paris, Groupe Hospitalier Universitaire de Paris Centre, Hôpital Cochin, Paris, France; ^7^AP-HP, Hôpital Louis Mourier, Colombes, France; ^8^CHRU de Lille, Lille, France; ^9^CHU Point-à-Pitre, Point-À-Pitre, France; ^10^CHU de Nice, Nice, France; ^11^Hopital Foch, Suresnes, France; ^12^CHU de la Cavale Blanche, Brest, France; ^13^Nouvel Hôpital Civil, Strasbourg, France; ^14^Centre Hospitalier Saint Joseph-Saint Luc, Lyon, France; ^15^Assistance Publique des Hôpitaux de Paris, CHU Henri Mondor, Créteil, France; ^16^Centre Hospitalier Régional d’Orléans, Orléans, France; ^17^CHU d’Angers, Angers, France; ^18^Hospices Civils de Lyon, Groupement Hospitalier Universitaire Edouard Herriot, Lyon, France; ^19^CHRU de Tours, Tours, France; ^20^AP-HP, Hôpital Tenon, Paris, France; ^21^CHU de Rouen, Rouen, France; ^22^10 Centre Hospitalier Départemental de La Roche sur Yon, La Roche Sur Yon, France

**Correspondence:** Jean-Pierre Frat - jean-pierre.frat@chu-poitiers.fr

*Annals of Intensive Care* 2021, **11(Suppl 1):**CO-031

**Rationale:** Critically ill obese patients may have an increased risk of difficult intubation and subsequent severe hypoxemia. We hypothesized that pre-oxygenation with noninvasive ventilation before intubation as compared with high-flow nasal cannula oxygen may decrease the risk of severe hypoxemia in obese patients.

**Patients and methods/materials and methods:** Post hoc subgroup analysis of critically ill obese patients (body mass index ≥ 30 kg m^−2^) from a multicenter randomized controlled trial comparing preoxygenation with noninvasive ventilation and high-flow nasal oxygen before intubation of patients with acute hypoxemic respiratory failure (PaO2/FIO2 < 300 mmHg). The primary outcome was the occurrence of severe hypoxemia (pulse oximetry < 80%) during the intubation procedure according to the preoxygenation strategy.

**Results:** Among the 313 patients included in the original trial^1^, 91 (29%) were obese with a mean body mass index of 35 ± 5 kg m^−2^. Obese patients were more likely to experience an episode of severe hypoxemia during intubation procedure than non-obese patients: 34% (31/91) vs. 22% (49/222); difference, 12%; 95% CI 1 to 23%; *P* = 0.03. Among obese patients, 40 received preoxygenation with noninvasive ventilation and 51 with high-flow nasal oxygen. Severe hypoxemia occurred in 15 patients (37%) with noninvasive ventilation and 16 patients (31%) with high-flow nasal oxygen (difference, 6%; 95% CI −13 to 25%; *P* = 0.54). The lowest pulse oximetry values during intubation procedure were 87% [interquartile range, 77–93] with noninvasive ventilation and 86% [78–92] with high-flow nasal oxygen (*p* = 0.98). After multivariable analysis, factors independently associated with severe hypoxemia in obese patients were intubation difficulty scale > 5 points and respiratory primary failure as reason for admission.

**Conclusion:** Obese patients with acute hypoxemic respiratory failure had an increased risk of severe hypoxemia during intubation procedure as compared to non-obese patients. However, preoxygenation with noninvasive ventilation did not reduce this risk compared with high-flow nasal oxygen.

**Reference**

1. Frat JP et al. Lancet Respir Med 2019; 7: 303–12.

**Compliance with ethics regulations:** Yes in clinical research. 
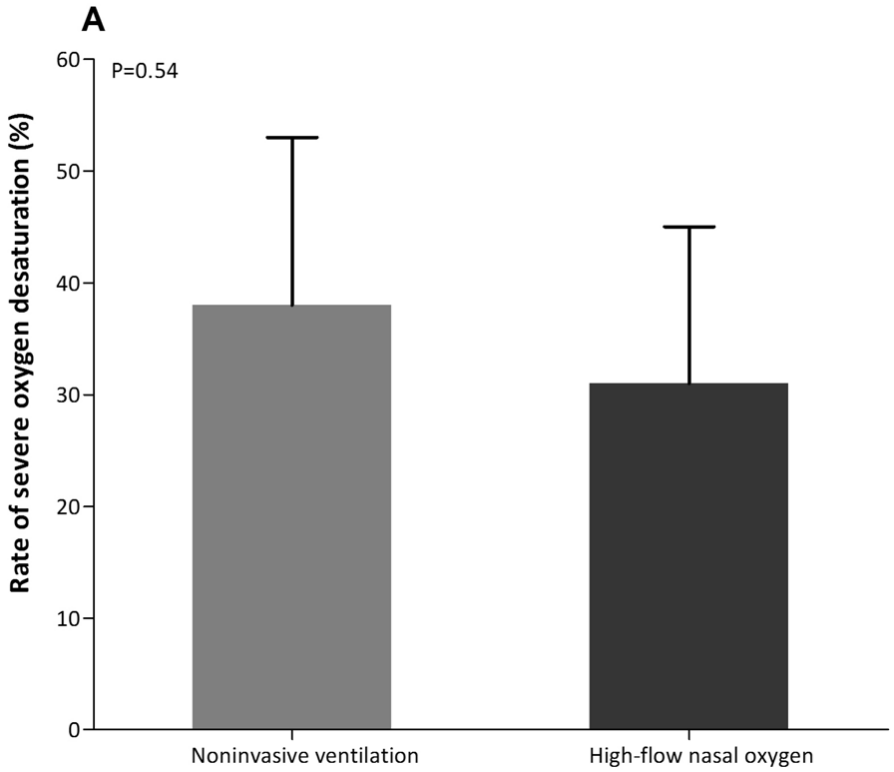


Rates of severe hypoxemia during intubation procedure in obese patients after preoxygenation with non-invasive ventilation (grey bar) and high-flow nasal cannula oxygen therapy (dark bar).

### CO-032 Capnogram interpretation to guide ventilation during Cardiopulmonary Resuscitation

#### Arnaud Lesimple^1,2^, Caroline Fritz^7^, Corentin Brocard^1^, François Morin^3^, Nathan Prouvez^1^, Bilal Badat^1^, Dominique Savary^3,8^, Renaud Tissier^9^, Alain Mercat^4^, Laurent Brochard^5,6^, François Beloncle^2,4^, Jean-Christophe Richard^4^

##### ^1^Med2Lab, Air Liquide Medical Systems (ALMS), Antony, France; ^2^CNRS, INSERM 1083, MITOVASC, Université d’Angers, Angers, France; ^3^Emergency Department, University Hospital of Angers, VentLab, Angers, France; ^4^Critical Care Department, Angers University Hospital, VentLab, Angers, France; ^5^Keenan Research Centre for Biomedical Science, Li Ka Shing Knowledge Institute, St. Michael’s Hospital, Toronto, Canada; ^6^Interdepartmental Division of Critical Care Medicine, University of Toronto, Toronto, Canada; ^7^Department of Anesthesia and Intensive Care Medicine, Hôpital Européen Georges Pompidou, AP-HP, Paris, France; ^8^Inserm, EHESP, University of Rennes, Irset (Institut de Recherche en Santé, Environnement et Travail) - UMR_S 1085, Rennes, France; ^9^U955 - IMRB, Inserm, UPEC, Ecole Nationale Vétérinaire d’Alfort, Créteil, France

**Correspondence:** Arnaud Lesimple - arnaud.lesimple@airliquide.com

*Annals of Intensive Care* 2021, **11(Suppl 1):**CO-032

**Rationale:** During cardio-pulmonary resuscitation (CPR), chest compressions (CC) tend to decrease lung volume below the functional residual capacity (FRC). This phenomenon may induce intrathoracic airway closure [1], and possibly impact both ventilation and circulation. On the opposite, large manual bag insufflations above FRC (“thoracic distension”) may excessively increase intra-thoracic pressure and thus jeopardize circulation. These two phenomena impact differently capnogram during CC. When there is no thoracic airway closure nor thoracic distension, ventilation could become a “targeted ventilation”. The objective of the present study was to develop an algorithm to identify thoracic airway closure, thoracic distension and targeted ventilation based on the analysis of capnogram (CO2 patterns) recorded during CC from clinical data.

**Patients and methods/materials and methods:** Anonymized capnograms from 122 out-of-hospital cardiac arrest (OHCA) patients obtained immediately after intubation (LIFEPACK 15, Physio-Control, WA 98052) were analyzed (RENAU registry). An algorithm was developed on Python to classify a ventilation cycle into thoracic airway closure, thoracic distension or targeted ventilation (Fig. 1). In order to validate the algorithm, three physicians independently identified those clinical cases based on capnogram visual inspection and comparison was performed. Krippendorff’s alpha score was computed to test physicians’ classification agreement (between physicians and in comparison with algorithm classification).

**Results:** Classification is illustrated on Fig. 1. For each situation, the upper image displays a typical capnogram from clinical recordings. Lower images were obtained after numerical data were extracted with a dedicated application. Each image shows one ventilation cycle composed of expiration and insufflation. From the 122 patients included in the study, 34% of patients showed airway closure, 29% displayed thoracic distension pattern and 37% showed targeted ventilation. Krippendorff’s scoring reached 0.79, suggesting excellent agreement.

**Conclusion:** EtCO2 interpretation during CC remains challenging. Its absolute value may not be adapted during CPR as CC may complexify its interpretation. In this context, we propose an original approach to identify and interpret more accurately expired CO2 during CC. Both thoracic airway closure and thoracic distension can be identified using CO2 patterns. This study is a first step in guiding CPR using capnograms [2].

**References**

1. Grieco DL et al. Intrathoracic Airway Closure Impacts CO2 Signal and Delivered Ventilation during Cardiopulmonary Resuscitation. Am J Respir Crit Care Med. 2019; 199:728–37.
2. Cordioli RL et al. New physiological insights in ventilation during cardiopulmonary resuscitation. Current Opinion in Critical Care 2019; 25:37–44.

**Compliance with ethics regulations:** Yes in clinical research.
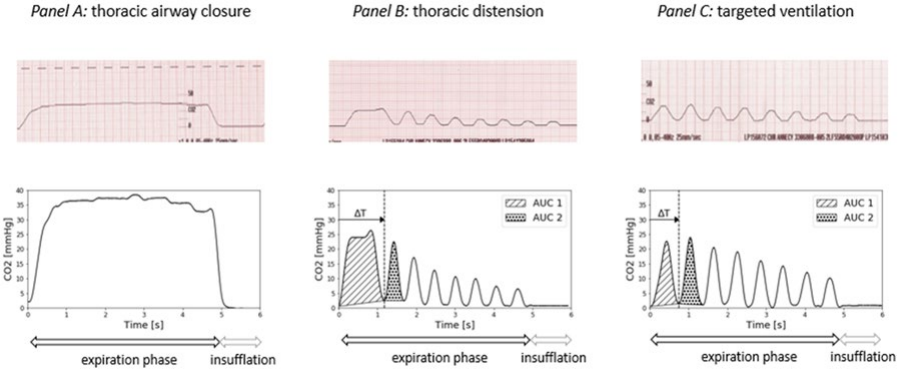


This figure shows examples of typical capnograms obtained in OHCA patients illustrating: thoracic airway closure (**A**), thoracic distension (**B**) and targeted ventilation (panel C).

### CO-033 Survival in immunocompromised patients ultimately requiring invasive mechanical ventilation: a pooled individual patient data analysis

#### Guillaume Dumas^1^, Virgine Lemiale^1^, Nisha Rathi^2^, Andrea Cortegiani^3^, Frédéric Pène^4^, Vincent Bonny^1^, Jorge Salluh^5^, Guillermo Muniz^6^, Marcio Soares^5^, Ayman O. Soubani^7^, Emmanuel Canet^22^, Tarik Hanane^8^, Achille Kouatchet^9^, Djamel Mokart^10^, Pia Lebiedz^11^, Melda Turkoglu^12^, Remi Coudroy^13^, Kyeongman Jeon^14^, Alexandre Demoule^15^, Geeta Mehta^16^, Pedro Caruso^17^, Jean-Pierre Frat^13^, Kuang-Yao Yang^18^, Oriol Roca^19^, John Laffey^20^, Jean-François Timsit^21^, Elie Azoulay^1^, Michael Darmon^1^

##### ^1^Hopital Saint louis, Paris, France; ^2^Department of Critical Care, MD Anderson Cancer Center, Houston, Etats-Unis; ^3^Department of Surgical, Oncological and Oral Sciences, Palerme, Italie; ^4^Medical Intensive care Unit, Cochin Teaching Hospital, Paris, France; ^5^The Department of Critical Care and Graduate Program in Translational Medicine, D’Or Institute for Research and Education, Programa de Pós-Graduação em Clínica Médica, Rio De Janeiro, Bresil; ^6^Instituto de Investigación Sanitaria del Principado de Asturias, Unidad de Cuidados Intensivos Cardiológicos. Hospital Universitario Central de Asturias. Instituto Universitario de Oncología del Principado de Asturias. Oviedo, Spain. CIBER-Enfermedades re, Oviedo, Espagne; ^7^Division of Pulmonary, Critical Care and Sleep Medicine, Wayne State University School of Medicine, Detroit, Etats-Unis; ^8^Department of Critical Care, Cleveland clinic, Cleveland, Etats-Unis; ^9^Medical Intensive care Unit, Angers Teaching hospital, Angers, France; ^10^Intensive care unit, Institut Paoli Calmettes, Marseille, France; ^11^Intensive care unit, Evangelisches Krankenhaus, Oldenburg, Allemagne; ^12^Medical Intensive care unit, Gazi University School of Medicine, Ankara, Turquie; ^13^CHU de Poitiers, Médecine Intensive Réanimation, Poitiers, France; INSERM CIC1402, groupe ALIVE, Université de Poitiers, Poitiers, France; ^14^Department of Critical Care Medicine and Division of Pulmonary and Critical Care Medicine, Department of Medicine, Samsung Medical Center, Sungkyunkwan University School of Medicine, Irwon-ro, Gangnam-gu, Seoul, Republique De Coree; ^15^APHP Sorbonne Université site Pitié-Salpêtrière, Service de Pneumologie, Médecine Intensive et Réanimation (Département R3S) and INSERM, UMRS1158 neurophysiologie respiratoire expérimentale et clinique, Sorbonne Université, Paris, France; ^16^Medical Surgical ICU, Mount Sinai Hospital, University of Toronto, Toronto, Canada; ^17^Intensive Care Unit, AC Camargo Cancer Center, São Paulo, Bresil; ^18^Department of Chest Medicine, Taipei Veterans General Hospital; School of Medicine, National Yang-Ming University, Taipei, Taiwan; ^19^Servei de Medicina Intensiva, Hospital Universitari Vall d’Hebron, Institut de Recerca Vall d’Hebron, Barcelona, Spain. Departament de Medicina, Universitat Autònoma de Barcelona, Bellaterra, Spain. Ciber Enfermedades Respiratorias (Ciberes), Instituto de, Madrid, Espagne; ^20^Departments of Anesthesia and Intensive Care Medicine, NUI Galway, Galway, Irlande; ^21^Medical and Infectious Diseases ICU, Bichat-Claude Bernard Hospital, UMR 1137 Inserm, Paris, France; ^22^Medical Intensive care Unit, Nantes Teaching hospital, Nantes, France, Nantes, France

**Correspondence:** Guillaume Dumas - dumas.guillaume1@gmail.com

*Annals of Intensive Care* 2021, **11(Suppl 1):**CO-033

**Rationale:** Acute respiratory failure (ARF) is associated with high mortality in immunocompromised patients, particularly when invasive mechanical ventilation is needed. Therefore, noninvasive oxygenation strategies have been developed to avoid intubation, with uncertain impact on mortality, especially when intubation is delayed. We sought to report trends of survival over time in immunocompromised patients receiving intubation and mechanical ventilation. The impact of late intubation after failure of noninvasive strategies was also assessed

**Patients and methods/materials and methods:** Systematic review and meta-analysis using individual data (IPD) of studies which focused on immunocompromised adult patients with ARF requiring invasive mechanical ventilation. Studies published in English were identified through PubMed, Web of science, and the Cochrane Central (2008–2018). IPD were requested to corresponding authors for all identified studies. We used mixed-effect models to estimate the effect of late intubation on hospital mortality and described adjusted mortality rates over time.

**Results:** Overall, 11 087 patients were included (24 studies), including 7705 (74%) who were intubated within 24 h of ICU admission (early intubation). Crude mortality rate was 53.2%. Adjusted survivals improved over time (OR for hospital mortality per year: 0.96[0.95–0.97]). For each elapsed day between ICU admission and intubation, mortality increased (*p* < 0.001). Early intubation was significantly associated with decreased mortality (OR: 0.80 [0.70–0.93]), regardless of initial oxygenation strategy

**Conclusion:** In immunocompromised intubated patients, survival has improved over time. Time between ICU admission and intubation is a strong predictor of mortality, suggesting a detrimental effect of initial oxygenation failure.

**Compliance with ethics regulations:** Yes in clinical research.

### CO-034 Short- and long-term outcomes of patients with lung cancer and life-threatening complications: a 12-year retrospective study

#### Clara Vigneron^1^, Julien Charpentier^1^, Marie Wislez^1^, Jean-Paul Mira^1^, Aurélie Lefebvre^1^, Ludovic Fournel^1^, Matthieu Jamme^2^, Frédéric Pène^1^

##### ^1^Hôpital Cochin, Paris, France; ^2^Centre Hospitalier Poissy Saint Germain en Laye, Poissy, France

**Correspondence:** Clara Vigneron - claravigneron@hotmail.fr

*Annals of Intensive Care* 2021, **11(Suppl 1):**CO-034

**Rationale:** Lung cancer accounts for the most common malignancy in patients with solid tumors requiring unplanned ICU admission. Recent therapeutic advances through immunotherapy and targeted therapy have resulted in improved survival, but may also induce new toxicities. How such changes in the landscape of lung cancer may impact on indications for ICU admissions and the eventual outcomes remains unknown. The objective of this study was to address the trends in ICU-admission patterns and prognosis of lung cancer patients with life-threatening complications.

**Patients and methods/materials and methods:** We conducted a retrospective monocenter study including patients with lung cancer (diagnosed before or during the ICU stay, all stages and histologic types) and requiring unplanned admission over a 12-year period (2007–2018). Independent predictors of short-term (in-ICU) and long-term (1-year) outcome were addressed by a cause-specific multivariate Cox regression analysis.

**Results:** 379 patients were included in the study. The number of patients dramatically increased over the study period, though likely linked to the development of the thoracic oncology department in our hospital. Non-small cell lung cancer accounted for the large majority (75%) of cancer subsets. The proportion of patients with advanced metastatic disease increased over time, as well as the number of patients under immunotherapy or targeted therapy. ICU-admissions related to medical and procedural adverse events increased over time from 4.0% in 2007–2008 to 11.6% in 2017–2018 (*p* = 0.02). ICU, hospital and one-year mortality rates were 32.2%, 53.3% and 68.9% and did not significantly change over the study period despite increasingly advanced malignancy. Factors independently associated with ICU mortality were a baseline Performance Status (PS) ≥ 3 and the requirements for invasive and non-invasive mechanical ventilation. One-year mortality was independently associated with advanced or metastatic cancer stage, baseline PS ≥ 3, increased acute severity at the time of ICU admission as assessed by the Simplified Acute Physiology Score 2, decisions to forgo life-sustaining therapies at the time of ICU discharge and continuation of chemotherapy or immunotherapy or both after ICU discharge (Fig. 1).

**Conclusion:** Therapeutic advances in lung cancer seemingly prompted broader ICU admission policy. Despite the trend towards more advanced malignancy stages, both short-term and long-term survival rates remained stable over the study period. Continuation of chemotherapy after ICU discharge is a major prognostic factor of one-year survival.

**Compliance with ethics regulations:** Yes in clinical research.
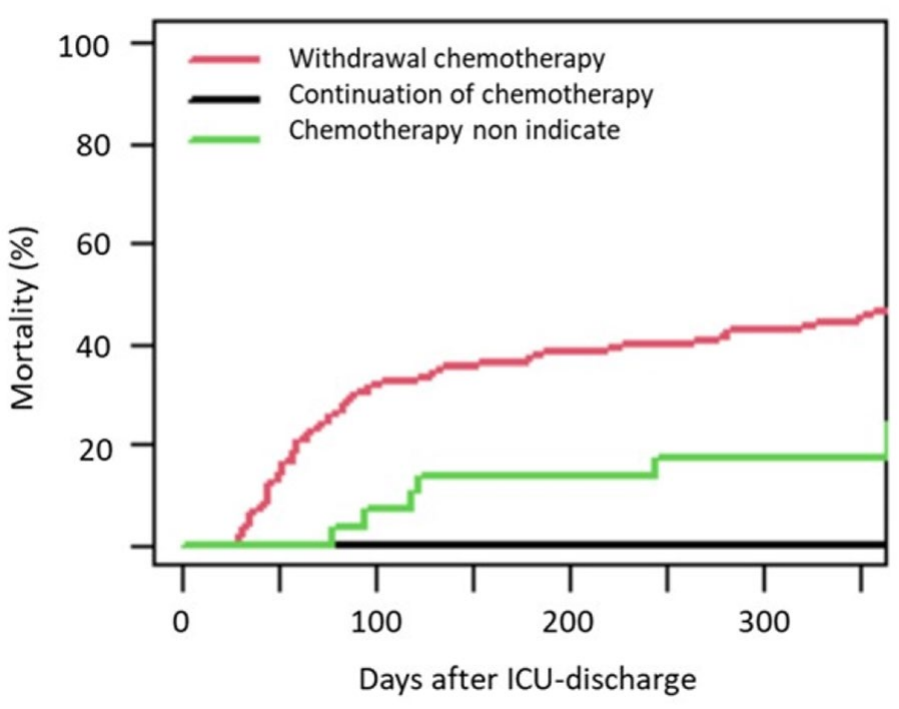


Fig. 1 One year-mortality in ICU-survivors according to the ability to receive oncologic treatment (landmark set at 1 month following ICU-discharge)

### CO-035 Problem of mechanical ventilation in the resuscitation service of the University Hospital Gabriel Touré in Bamako, Mali

#### Thierno Madane Diop^1^, Moustapha Issa Mangane^1^, Abdoul Hamidou Almeimoune^1^, Mama Daou^1^, André Kassogue^1^, Mahamadoun Coulibaly^3^, Aminata Dabo^3^, Kassoum Ouattara^1^, Aladji Saidou Dembele^2^, Mahamane Diango Djibo^1^

##### ^1^CHU Gabriel Toure, Bamako, Mali; ^2^CHU IOTA, Bamako, Mali; ^3^CHU Mére Enfant Le Luxembourg, Bamako, Mali

**Correspondence:** Thierno Madane Diop - madane.diop@gmail.com

*Annals of Intensive Care* 2021, **11(Suppl 1):**CO-035

**Rationale:** In sub-Saharan Africa, and particularly in Mali, very few data are available on the practice of artificial ventilation (AV) in intensive care. This study aimed to evaluate practice of AV in the intensive care unit of the Gabriel Touré CHU.

**Patients and methods/materials and methods:** Descriptive and analytical study with prospective collection, from March 1, 2020 to December 31, 2020. All patients admitted to intensive care who underwent artificial ventilation for at least 6 h were included.

**Results:** Among 365 patients admiited in ICU, 72 patients (19.72%) were put on AV. Mean age was 36.47 ± 18.77 years. The sex ratio was 0.95. The patients were mainly admitted from the emergency department (37.5%). Severe head trauma (25%), was the most frequent cause of ICU admission followed by eclampsia (13.9%). The MV indication was acute respiratory failure 43.1%. Midazolam + fentanyl were combined as sedative drugs in 57.1% of the cases. The volumetric mode was used in 82% of patients with a tidal volume of 6–8 ml/kg according to the theoretical ideal weight. The mean duration of sedative drug infusion was 2.27 ± 1.2 days. The mean duration of AV was 4.51 ± 3.7 days. Complications were recorded: PAVM 77.8%, pneumothorax 11.1% and atelectasis 11.1%. We have encountered incidents in 55.6% of the type of O2 pressure drop from the power plant O2 (42%); 37% mucosal plug obstructions. Weaning has been initiated in 61.1% of patients and 38.6% patients has mechanical ventilation withdrawal difficulties. Mortality was 58%.

**Conclusion:** The practice of mechanical ventilation in our context comes up against technical difficulties and high morbidity and mortality, linked to the occurrence of complications and maintenance problems.

**Reference**

1. Gay R. Réanimation et ventilation artificielle. Survol historique jusqu’en 1950. Cah Anesthesiol 2005;53:231–48.

**Compliance with ethics regulations:** Yes in clinical research.

### CO-036 The predictive value of diaphragm thickness fraction on postoperative pulmonary complications after digestive cancer surgery

#### Oussama Ssouni^1^, Abdelilah Ghannam^1^, Brahim El Ahmadi^1^, Zakaria Belkhadir^1^, Amal Bouziane^2^, Redouane Abouqal^3,4^

##### ^1^Department of intensive care, National Institute of Oncology, Faculty of Medicine and Pharmacy, Mohammed V University in Rabat, Morocco., Rabat, Maroc; ^2^Department of periodontology, Faculty of Dental Medicine, Mohammed V University in Rabat, Morocco., Rabat, Maroc; ^3^Acute Medical Unit, Ibn Sina University Hospital, Rabat, Morocco., Rabat, Maroc; ^4^Laboratory of Biostatistics, Clinical Research and Epidemiology, Mohammed V University in Rabat, Morocco., Rabat, Maroc

**Correspondence:** Oussama Ssouni - ssounioussama@gmail.com

*Annals of Intensive Care* 2021, **11(Suppl 1):**CO-036

**Rationale:** Postoperative pulmonary complications (PPCs) increase mortality, as well as the duration and costs of hospitalization. The diaphragm is the main muscle of respiration. Its function can be characterized by the measurement of its thickness fraction (TFdi) at its attachment zone using ultrasonography. We, therefore, sought to determine if a low preoperative TFdi determined by ultrasonography could help to predict the occurrence of postoperative pulmonary complications.

**Patients and methods/materials and methods:** Diaphragmatic ultrasound was performed 24 to 48 h before the operation, then repeated postoperatively (within 24 h after his admission to intensive care, then at day 3). We measured the thickness of the right and left hemidiaphragms at their zone of apposition at end-expiration (TEE) and peak-inspiration (TPI). The maximal thickening fraction of the diaphragm during inspiration was calculated using the following formula: TFdi, max = (TPI–TEE)/TEE. We evaluated other potential risk factors including demographic and biological parameters, comorbidities, ASA Physical Status Classification, estimated functional capacity in METs. We also collected information related to surgical and anesthetic procedures.

**Results:** 55 patients (34.6%) of the 159 included in the study developed PPCs. The length of in ICU stay was significantly longer in patients who developed PPCs with significantly higher mortality. TFdi decreased postoperatively and remained lower in patients with PPCs [44.83% ± 11.07 vs 31.54% ± 8.45; *p* < 0.001]. In multiple logistic regression, the preoperative risk factor independently related to the occurrence of PPCs were supramesocolic surgery [OR: 9.94; 95%CI: 3.62–27.29; *p* < 0.001] and a TFdi,max less than 37% [OR: 7.10; 95%CI: 1.71–18.60; *p* < 0.001]. Performing an epidural procedure was identified as a protective factor [OR: 0.21; 95%CI: 0.052–0.87; *p* = 0.031].

**Conclusion:** A low preoperative TFdi,max can help to identify patients at increased risk of postoperative pulmonary complications after digestive cancer surgery. This marker of diaphragmatic weakness could help to identify vulnerable patients who would benefit most from preventive strategies such as preoperative training of the inspiratory muscles.

**Compliance with ethics regulations:** Yes in clinical research.
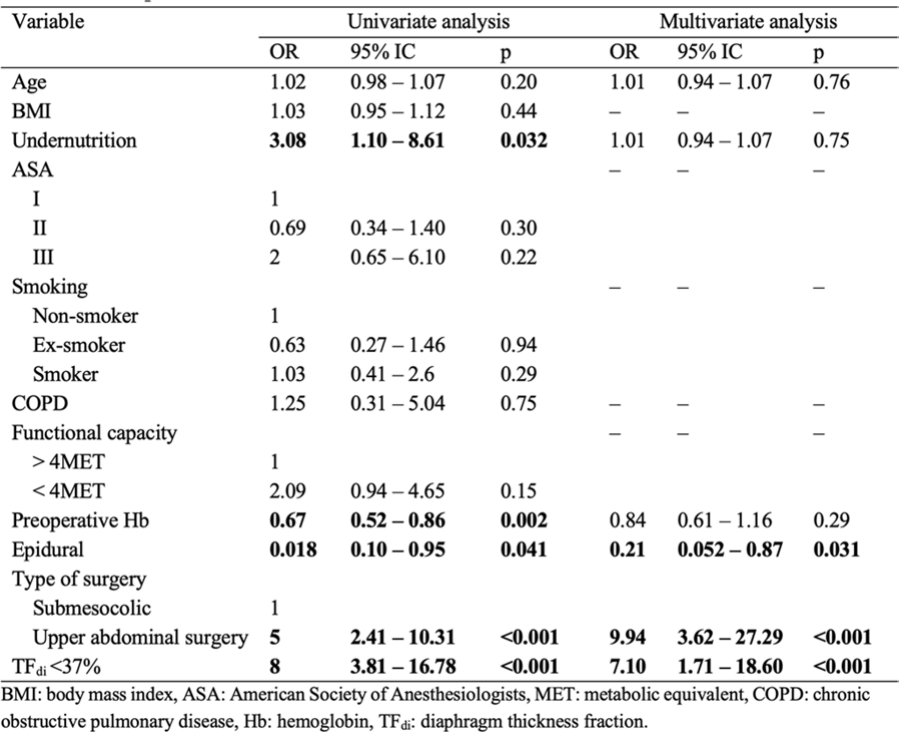


Preoperative risk factors of postoperative pulmonary complications (PPCs)

### CO-037 Efficacy and safety of awake prone positioning in severe critically ill COVID-19

#### Fatma Essafi^1,2^, Rihab Rajah^1^, Imen Talik^1,2^, Khaoula Ben Ismail^1,2^, Moez Kaddour^1,2^, Takoua Merhanbene^1,2^

##### ^1^Intensive care unit, Regional Hospital, Zaghouan, Tunisie; ^2^Faculty of Medicine of Tunis, University Tunis El Manar, Tunis, Tunisie

**Correspondence:** Fatma Essafi - fatma.essafi@fmt.utm.tn

*Annals of Intensive Care* 2021, **11(Suppl 1):**CO-037

**Rationale:** Prone positioning (PP) is now recommended for patients with moderate-to-severe acute respiratory distress syndrome (ARDS) receiving invasive mechanical ventilation in addition to sedation and neuromuscular blockers. Recently, use of PP has been extended to spontaneously breathing patients affected with COVID-19 ARDS. The aim of our study was to assess if prone position can reduce the rate of intubation.

**Patients and methods/materials and methods:** This study was performed at the Zaghouan’s hospital ICU, a 10-bed tertiary ICU in Tunisia. It was a retrospective study between 1st May 2020 and 31st January 2021. All consecutive patients with a confirmed diagnosis of severe COVID-19 pneumonia (PaO2/FiO2 ≤ 300 mmHg) who did not receive invasive mechanical ventilation for the first 24 h of ICU stay were included. Patients were helped to turn on the PP, which was maintained for a minimum duration of consecutive 3 h. Patients were classified into 2 groups: group (G) 1 = patients who benefit of awake PP and group (G) 2 = supine group. We compared demographics, clinical, paraclinic and evolution data.

**Results:** During the study period, 121 patients with COVID-19-related pneumonia were admitted, 113 met the inclusion criteria. PP was performed in 74 patients (65.5%) and was impractical in 39 patients. comorbidities were similar between the 2 groups. Mean age was 60 ± 12.5 years in group 1 and 66.3 ± 11.8 years in group 2 (*p* = 0.013). Means SAPS II and APACHE II scores were, respectively, 24 ± 7 vs 27 ± 9 (*p* = 0.026) and 7 ± 4 VS 9 ± 3 (*p* = 0.06). Obesity was noted in 71.6% in G1 vs 56.4% (*p* = 0.1) in G2. PaO2/FiO2 was lower in G1 (156 ± 110.7 mmHg vs 167.3 ± 87.5 mmHg, *p* = O.7). Neuropsychiatric signs were more frequent in G2 (19% vs 43%; *p* = 0.005). On ICU admission, non-invasive ventilation associated to high-flow nasal oxygen therapy was used in 90.5% in G1 vs 89.7% (*p* = 0.19) in G2. PP was applied in all patients of G1 on the first admission day. Median duration of daily PP session and total length of PP were, respectively, 12 h/day and 7 ± 4 [1–19] days. Need for tracheal intubation was lower in G1 (27% vs 56.4%, *p* = 0.002). Means duration of total mechanical ventilation and ICU length of stay were comparable. In-ICU mortality was significantly higher in G 2 (64% vs 28%; *p* < 0.05). No relevant side-effects or complications of PP were observed.

**Conclusion:** Spontaneously PP is feasible and effective in severe COVID-19 patients. Our study showed that it can reduce need for tracheal intubation and mortality without affecting length of in-ICU stay.

**Compliance with ethics regulations:** N/A.

### CO-038 Feasibility and efficacy of HFNC and prone positioning in patients with acute respiratory failure due to COVID-19: a Tunisian series

#### Wiem Nouira, Zeineb Hammouda, Manel Lahmar, Syrine Maatouk, Sourour Belhajyoussef, Saoussen Benabdallah, Fahmi Dachraoui, Fekri Abroug, Lamia Ouanes-Besbes

##### CHU F.Bourguiba, Monastir, Tunisie

**Correspondence:** Fekri Abroug - fekri.abroug@gmail.com

*Annals of Intensive Care* 2021, **11(Suppl 1):**CO-038

**Rationale:** There is a change of paradigm relating to ventilation support therapy use of hypoxemic patients with COVID-19, spontaneous ventilation is now recommended after being discouraged during the first wave. Prone positioning is a gold standard in mechanically ventilated severe ARDS while less experience is available in spontaneously breathing hypoxemic patients. The aim of the study is to investigate the feasability and efficacy of prone positioning in non-intubated patients with acute respiratory failure due to COVID-19.

**Patients and methods/materials and methods:** Between September 02, and December 03, 2020, consecutively admitted patients with confirmed COVID-19 and acute hypoxemic respiratory failure, who required non-invasive ventilation by high-flow nasal cannula (HFNC) were considered for inclusion. The following information was collected on ICU admission: age, gender, time between the onset of symptoms and hospital admission, coexisting morbidities. Respiratory variables recorded were baseline peripheral oxygen saturation (SpO2 on AA), respiratory rate (RR), heart rate (HR), the ROX index and PaO2/FiO2 ratio before and after the first prone positioning session, the number and duration of prone positioning sessions. Failure of HFNC was defined as the need for intubation which was based on standardized criteria. Duration of in-ICU stay and the discharge status (dead or alive) were recorded.

**Results:** During the study period, 84 patients were admitted to the medical ICU with severe acute hypoxemic respiratory failure due to COVID-19. Of these, 11 were invasively ventilated, and 73 patients received HFNC and were included in the study. Their mean age was 62 years, mainly male (75%). The mean SAPS II score was 29, the mean BMI was 28.82 kg/m^2^. 38.4% of patients had diabetes mellitus and 48% had hypertension. The mean duration of stay in the ICU was 10.55 days. The mean pulse oxygen saturation (SpO2 AA) was 79 ± 10%. Fifty-six patients could stand the prone position and 17 could not. In patients who had prone position, the mean baseline ROX index and PaO2/FiO2 Ratio were, respectively, 4.32 ± 2 and 99 ± 50 and both variables improved after the first prone positioning session (respectively, 5.97 and 136; Fig. 1). The mean number of total prone positing sessions was 3.75 and the mean duration of the first prone positioning session was 14.4 h. In patients managed with HFNC and prone position, 20 patients (35.7%) were eventually intubated and mechanically ventilated.

**Conclusion:** Our study confirms suggested feasibility and efficacy of HFNC and prone positioning in non-intubated patients with respiratory failure due to COVID-19.

**Compliance with ethics regulations:** Yes in clinical research.
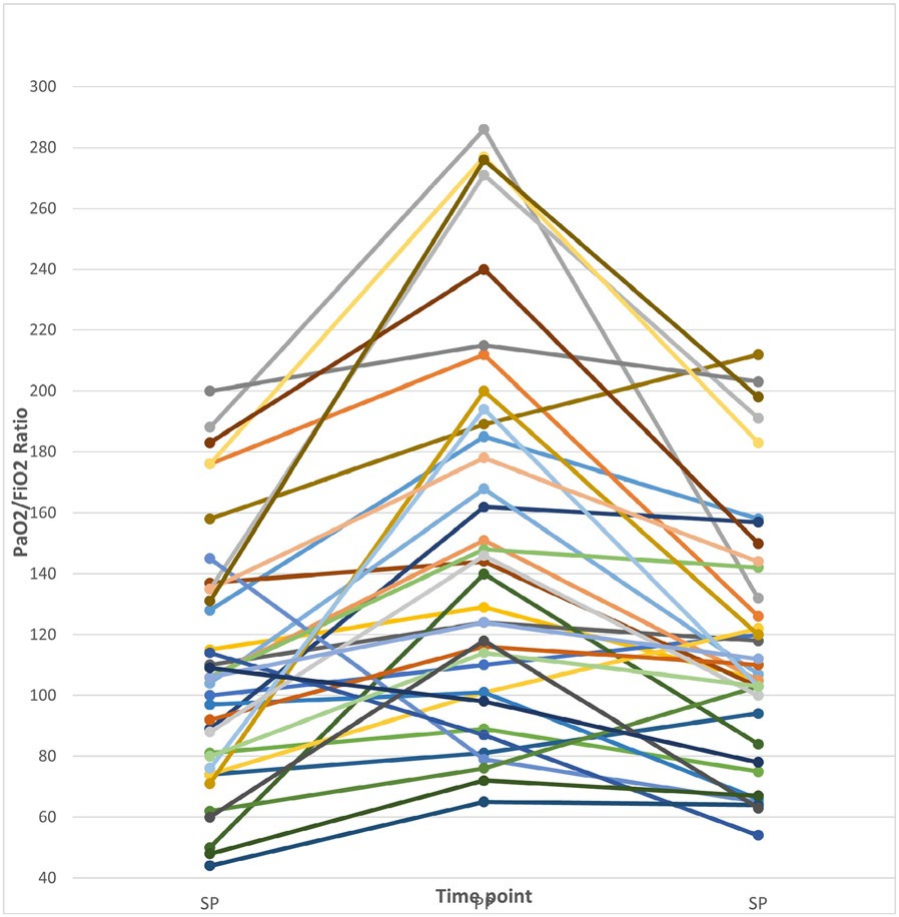


Change in the PaO2/FiO2 ratio in supine and prone position

### CO-039 Use of prone positioning in non-intubated patients with COVID-19 and hypoxemic acute respiratory failure

#### Xavier Elharrar^2^, Youssef Trigui^2^, Anne-Marie Dols^3^, Francois Touchon^2^, Stephanie Martinez^2^, Eloi Prud’homme^1^, Laurent Papazian^1,4^

##### ^1^Assistance Publique - Hôpitaux de Marseille, Médecine Intensive Réanimation, Marseille, France, Marseille, France; ^2^Centre Hospitalier d’Aix-en-Provence, Service des Maladies Respiratoires, Aix-en-Provence, France, Aix-En-Provence, France; ^3^Université Grenoble Alpes, Faculté de médecine, Grenoble, France, Grenoble, France; ^4^Aix-Marseille Université, Centre d’Etudes et de Recherches sur les Services de Santé et qualité de vie EA 3279, Marseille, France, Marseille, France

**Correspondence:** Eloi Prud’homme - eloiprudhomme@gmail.com

*Annals of Intensive Care* 2021, **11(Suppl 1):**CO-039

**Rationale:** Awake PP (prone positioning) may be useful to improve oxygenation and prevent ICU transfer in patient with COVID-19 requiring oxygen supplementation^1^. The objective of the study was to evaluate the feasibility, efficacy and tolerance of PP in awake COVID-19 patients hospitalized outside the ICU.

**Patients and methods/materials and methods:** This prospective, single-center, before–after study was done in awake, non-intubated, spontaneously breathing COVID-19 patients with hypoxemic acute respiratory failure requiring oxygen supplementation, from March 27th to April 8th, 2020. Patients with confirmed COVID-19 were eligible if they required oxygen supply. The same oxygen supply (device and FiO2) was maintained during the study period. Arterial blood gases were performed just before, during PP, and 6 to 12 h after resupination. The main outcome was the proportion of responders (PaO2 increase ≥ 20% between before and during PP). Secondary outcomes included PaO2 and PaCO2 variation (difference in PaO2 or PaCO2 between before and during PP or after resupination), feasibility (proportion of patients sustaining PP ≥ 1 h and ≥ 3 h) and proportion of persistent responders (PaO2 increase ≥ 20% between before PP and after resupination). Tolerance was monitored with 10-cm Visual Analog Scales. IRB approval was obtained.

**Results:** A total of 88 COVID-19 patients were admitted during the period. Among the 25 eligible, 24 agreed to participate; 4 (17%) did not tolerate PP for more than 1 h; 5 (21%) tolerated it for 1 to 3 h; 15 (63%) tolerated it for more than 3 h. Neither sedation nor anxiolytics were used. Six patients were responders to PP, representing 25% [95%CI, 12–45] of the 24 patients included and representing 40% (6/15) [95%CI, 20–64] of the patients who sustained PP ≥ 3 h. Three patients were persistent responders. Among patients who sustained PP ≥ 3 h, PaO2 increased from 73.6 mmHg (± 15.9) before, to 94.9 mmHg (± 28.3) during PP (median difference, 10 [95%CI, 2–41]; *p* = 0.006).No significant difference was found between PaO2 before PP and PaO2 after resupination (*p* = 0.53). None of the included patients experienced major complications. Back pain was reported by 10 patients (42%). At the end of the follow-up period, 5 patients required invasive mechanical ventilation. Four of them did not sustain PP ≥ 1 h and required intubation within 72 h.

**Conclusion:** In this study on COVID-19 patients with hypoxemic respiratory failure managed outside the ICU, 63% were able to tolerate PP for more than 3 h. However, oxygenation increased during PP in only 25% and was not sustained in half of those after resupination.

**Reference**

1. Sun Q, Qiu H, Huang M, Yang Y. Lower mortality of COVID-19 by early recognition and intervention: experience from Jiangsu Province. Annals of Intensive Care. 2020;10(1):33. 10.1186/s13613-020-00650-2.

**Compliance with ethics regulations:** Yes in clinical research.
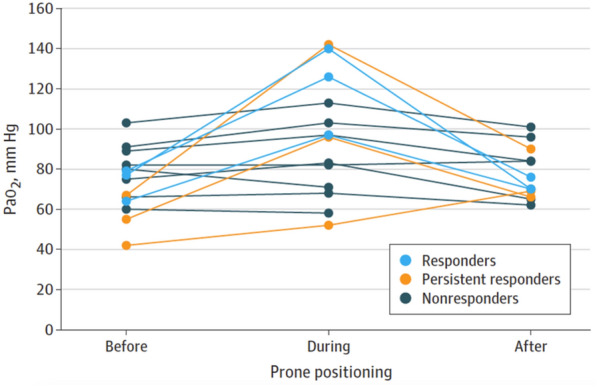


Individual partial pressure of arterial oxygen (PaO2) variation for patients who sustained prone positioning (PP) for at least 3 hours

### CO-040 Impact of prone position in non-intubated spontaneously breathing patients admitted to the ICU for severe acute respiratory failure ventilation due to COVID-19

#### Romain Jouffroy^1^, Michael Darmon^2,3^, Foucauld Isnard^1^, Guillaume Geri^1,6^, Alexandra Beurton^4^, Muriel Fartoukh^5^, Jean-Jacques Tudescq^2^, Safaa Nemlaghi^4^, Alexandre Demoule^4^, Elie Azoulay^2,3^, Antoine Vieillard-Baron^1,6^

##### ^1^APHP - Hôpital Ambroise Paré - Service de Médecine intensive et réanimation, Boulogne Billancourt, France; ^2^Service de Médecine intensive et réanimation, hôpital Saint-Louis, Paris, France; ^3^Université de Paris, Paris, France; ^4^Service de Médecine intensive et réanimation, hôpital Pitié Salpêtrière, Assistance Publique Hôpitaux de Paris, Paris, France; ^5^Service de Médecine intensive et réanimation, hôpital Tenon, Assistance Publique Hôpitaux de Paris, Paris, France; ^6^INSERM UMR 1018, Clinical Epidemiology Team, CESP, Université de Paris Saclay, Villejuif, France

**Correspondence:** Romain Jouffroy - romain.jouffroy@aphp.fr

*Annals of Intensive Care* 2021, **11(Suppl 1):**CO-040

**Rationale:** Studies performed in spontaneously breathing patients with mild-to-moderate respiratory failure outside the intensive care unit (ICU) suggested that prone position (PP) in COVID-19 could be beneficial.

**Patients and methods/materials and methods:** In this retrospective, multicenter, observational study, consecutive critically ill patients with RT-PCR confirmed SARS-CoV-2 infections were recruited in four ICUs. PP sessions lasted at least 3 h each and were performed twice daily. A Cox proportional hazard model identified factors associated with the need of intubation and invasive ventilation. A propensity score overlap weighting analysis was performed to assess the association between spontaneous breathing PP (SBPP) and intubation.

**Results:** 379 patients were consecutively enrolled among which 40 (11%) underwent SBPP. Oxygenation was achieved by high-flow nasal canula in all but three patients. At ICU-admission PaO2/FiO2 was 90 [71;125] mmHg. Proning sessions were performed during 2.5 [1.6;3.4] days. SBPP was well tolerated hemodynamically (no change in heart rate, blood pressure and serum lactate), increased PaO2/FiO2 (78 [68;96] versus 63 [53;77] mmHg, *p* = 0.004) and increased PaCO2 (38 [34;43] versus 35 [32;38] mmHg, *p* = 0.005). After weighting for factors that may have influenced use of PP, neither day-28 survival (HR 0.51, 95% CI 0.16–1.16] nor risk of invasive ventilation [sHR 0.96; 95% CI 0.49;1.88] differed between patients who underwent PP and others.

**Conclusion:** SBPP in COVID-19 is feasible and well tolerated in severe hypoxemic patients. It did not induce beneficial or harmful effects on risk of invasive ventilation and day-28 mortality.

**Compliance with ethics regulations:** Yes in clinical research.

### CO-041 Effect of prone positioning on the respiratory support of non-intubated patients with COVID-19 and acute hypoxemic respiratory failure: a retrospective matching cohort study

#### Eloi Prud’homme^1^, Youssef Trigui^2^, Xavier Elharrar^2^, Marie Gaune^3^, Anderson Loundou^4^, Samuel Lehingue^5^, Arnaud Boyer^6^, Laurent Lefebvre^7^, Anne-Marie Dols^8^, Pascal Chanez^3^, Laurent Papazian^1,9^, Jean-Marie Forel^1,9^

##### ^1^Assistance Publique - Hôpitaux de Marseille, Médecine Intensive Réanimation, Marseille, France, Marseille, France; ^2^Centre Hospitalier d’Aix-en-Provence, Service des Maladies Respiratoires, Aix-en-Provence, France, Aix-En-Provence, France; ^3^Assistance Publique - Hôpitaux de Marseille, Service de Pneumologie, Clinique des Bronches, de l’Allergie et du Sommeil, Marseille, France, Marseille, France; ^4^Unité d’Aide Méthodologique à la Recherche Clinique, AP-HM/EA 3279, Faculté de Médecine, 27 Boulevard Jean Moulin 13005 Marseille, Marseille, France; ^5^Hôpital Saint-Joseph, Service de Réanimation Polyvalente, Marseille, France, Marseille, France; ^6^Hôpital Saint-Joseph, Service de Pneumologie, Marseille, France, Marseille, France; ^7^Centre Hospitalier d’Aix-en-Provence, Réanimation Polyvalente, Aix-en-Provence, France, Marseille, France; ^8^Faculté de médecine de Grenoble, Grenoble, France, Grenoble, France; ^9^Aix-Marseille Université, Centre d’Études et de Recherches sur les Services de Santé et qualité de vie EA 3279, Marseille, France, Marseille, France

**Correspondence:** Eloi Prud’homme - eloiprudhomme@gmail.com

*Annals of Intensive Care* 2021, **11(Suppl 1):**CO-041

**Rationale:** Awake PP (prone positioning) is feasible, improves oxygenation in some patients and may prevent respiratory worsening^1–2^. The main objective of the study was to evaluate the effect of PP on the outcome of spontaneously breathing COVID-19 patients with acute respiratory failure.

**Patients and methods/materials and methods:** We designed an exposed/non-exposed bicentric retrospective matched cohort study to assess the effectiveness of PP for patients hospitalized outside Intensive Care Unit (ICU) with COVID-19 requiring oxygen. Inclusion criteria were: hypoxemic respiratory failure requiring oxygen supplementation and PCR-confirmed SARS-CoV-2. The study received approval by the appropriate IRB (n° MR 3514070520). Two treatment strategies were compared: awake PP for at least 3 h a day during 3 consecutive days (PP group) and no instruction regarding positioning or no tolerance of PP during hospitalization (no-PP group). The oxygen supplementation strategy was to initiate first oxygen therapy, second HFNC, third pressure support with non-invasive ventilation (NIV), and finally invasive mechanical ventilation (IMV).The primary outcome of the study was the “upgrading of oxygen delivery method” evaluated on day 14 (D14) and defined by reaching at least 6 L/min with a doubling of the initial oxygen flow for usual oxygen supplementation, or initiating HFNC, or initiating NIV or IMV. The secondary outcome was death at D14.

**Results:** A total of 414 confirmed COVID-19 patients were admitted during the study period. Among the 168 patients eligible for analysis, 48 received PP for at least 3 h a day for 3 days and 120 did not. After performing a propensity score matching, 96 patients were analyzed (48 patients in each group). For the primary outcome, 15 (31.2%) patients in the PP group had an upgrading of oxygen delivery method at D14, compared with 25 (52.1%) in the no PP group (*p* = 0.038) with a Hazard Ratio (HR) of 2.03 (95% CI, 1.07–3.86), *p* = 0.003. For the secondary outcome, 4 (8.3%) patients in the PP group and 6 (12.5%) of the non-PP group died at D14 (*p* = 0.74). No patients in the PP-group died or required invasive mechanical ventilation within the first three days. No major adverse event was observed.

**Conclusion:** PP for at least 3 h a day during 3 consecutive days may be associated with a clinical benefit by preventing the upgrading of oxygen delivery method on awake, non-intubated, spontaneously breathing COVID-19 patients with acute hypoxemic respiratory failure requiring oxygen supplementation.

**References**

1. Elharrar X, Trigui Y, Dols A-M, et al. Use of Prone Positioning in Nonintubated Patients With COVID-19 and Hypoxemic Acute Respiratory Failure. JAMA. Published online May 15, 2020. 10.1001/jama.2020.8255.
2. Coppo A, Bellani G, Winterton D, et al. Feasibility and physiological effects of prone positioning in non-intubated patients with acute respiratory failure due to COVID-19 (PRON-COVID): a prospective cohort study. The Lancet Respiratory Medicine. 2020;8(8).

**Compliance with ethics regulations:** Yes in clinical research.
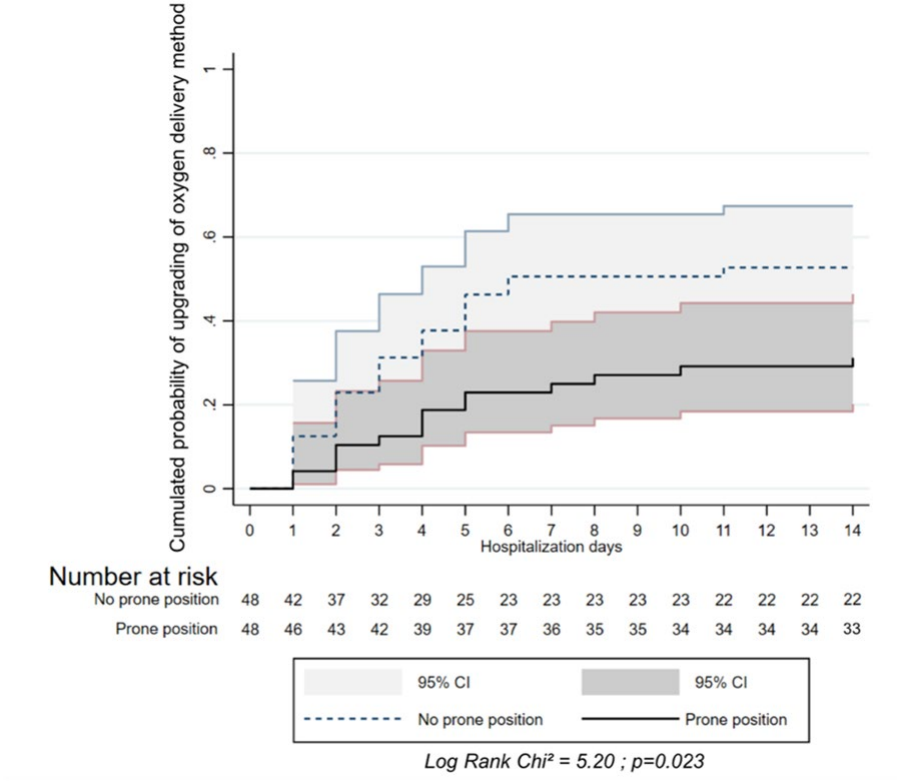


Cumulated probability of upgrading of oxygen delivery methdod

### CO-042 Awake prone position improves respiratory function without reducing inhomogeneity of ventilation in COVID-19-related acute respiratory failure: a prospective cross-over study

#### Thomas Brunelle^1^, Eloi Prud’homme^1^, Jean-Emmanuel Alfonsine^2^, Karine Baumstarck^3^, Saida Salmi^1^, Céline Sanz^1^, Sami Hraiech^1,3^, Jean-Marie Forel^1,3^, Laurent Papazian^1,3^, Christophe Guervilly^1,3^

##### ^1^Assistance Publique- Hôpitaux de Marseille, Marseille, France; ^2^Hôpital Européen, Marseille, France; ^3^Aix-Marseille Université, Marseille, France

**Correspondence:** Christophe Guervilly - christophe.guervilly@ap-hm.fr

*Annals of Intensive Care* 2021, **11(Suppl 1):**CO-042

**Rationale:** Awake prone position (aPP) has been proposed in COVID-19 patients with acute respiratory failure (ARF) to improve the oxygenation and thus to avoid mechanical ventilation in some cases. Therefore, we plan to investigate the physiological effects of aPP. We postulate that aPP reduces global inhomogeneity (GI) index of ventilation measured by electrical impedance tomography (EIT).

**Patients and methods/materials and methods:** Design and setting: This is a prospective, cross-over study in a French tertiary center. Patients: We screened all patients aged > 18 years admitted in the intensive care unit with RT-PCR confirmed COVID-19 disease. We included awake spontaneously breathing patients with ARF and hypoxemia (100 < PaO2:FiO2 < 200 mmHg) requiring O2 supply. Interventions: After baseline measurements of oxygenation parameters, respiratory rate, Borg scale and 30-min EIT recordings in the supine position (SP), patients were randomized into two sequences either SP-PP, or PP-SP. At the end of each 2-h step of the sequence, measurements of oxygenation parameters, respiratory rate, Borg scale and 30-min EIT recordings were repeated.

**Results:** Twenty patients, mean age of 60 ± 15 years, mainly male (75%) with ARF (mean PaO2:FiO2 of 135 ± 44 mmHg) were included in the study. Ten patients were randomized in the SP-PP sequence and 10 in the PP-SP sequence. Concerning GI index, we did not find any interaction between sequence and position (*p* = 0.053). Then, we find no significant change in GI index with comparison of each time point in the SP-PP group (baseline 74 ± 20%, end of SP 78 ± 23% and end of PP 72 ± 20%, *p* = 0.85) and in the PP-SP group (baseline 59 ± 14%, end of PP 59 ± 15% and end of SP 54 ± 13%, *p* = 0.67). PaO2/FiO2 increased at the end of PP (from 135 ± 44 at baseline to 183 ± 66 mmHg, *p* = 0.003), and decreased after back to SP (from 183 ± 66 mmHg at the end of PP to 130 ± 49 at the end of SP, *p* = 0.027). Respiratory rate decreased from 27 ± 6 at baseline to 22 ± 5 at the end of PP, (*p* = 0.002) and also as compared to the end of SP (23 ± 5, *p* = 0.003). Borg scale ranking did not differ at each time point and remained relatively low, 2.5 ± 2 at baseline, 2 ± 1.5 at end of PP and 2 ± 2 at end of SP (*p* = 0.647).

**Conclusion:** Despite no reduction of inhomogeneity of ventilation, aPP was associated with improvement in respiratory parameters in COVID-19 patients with acute respiratory failure. Dyspnea assessed with Borg scale was quite low both in prone and supine positions.

**Compliance with ethics regulations:** Yes in clinical research.

### CO-043 Echocardiographic patterns in critically ill patients during the Covid-19 pandemic

#### Ségolène Tran, Cyril Charron, Mathieu Godement, Xavier Repessé, Amelie Prigent, Pierre-Alexandre Haruel, Samuel Castro, Lola Girodias, Emilie Charbit, Foucault Isnard, Mohamed Saleh, Koceila Bouferrache, Bernard Page, Romain Jouffroy, Guillaume Geri, Antoine Vieillard-Baron

##### ^1^Hôpital Ambroise Paré, Boulogne-Billancourt, France

**Correspondence:** Ségolène Tran - segolene.tran@gmail.com

*Annals of Intensive Care* 2021, **11(Suppl 1):**CO-043

**Rationale:** Many studies were published on respiratory strategy and management in COVID-19 critically ill patients, while little is known on hemodynamics. The aim of our study was to report echocardiographic patterns in critically ill COVID-19 patients.

**Patients and methods/materials and methods:** Observational and descriptive study in COVID-19 patients admitted to in a single ICU between March 12 and November 27, 2020. Critical care echocardiography (CCE) was performed and retrospectively analyzed off-line. Echo patterns are reported during the first week (D_1–7_) following the admission, distinguished between the first 2 days (D_1–2_) and afterwards (D_3–7_).

**Results:** 146 patients were admitted during this period, among them 130 had at least 1 echocardiography during the first week which represents the population of interest. 77.9% were males, median age was 65 (56–73), body mass index 27.8 (24.8–31.8), SOFA score at admission 4 (3–5). 44.3% of patients were invasively ventilated and 36.6% required catecholamine infusion during the first week. The in-ICU mortality was 31.3%. CCE were performed for 80 patients (62%) by a transthoracic route (TTE) and in 50 patients (38%) by an esophageal route (TEE). TEE was only performed in intubated patients and in 91% of the patients who required catecholamines. Echo parameters are reported in the table in median. Among patients with catecholamines, we observed an LV ejection fraction (LVEF) < 45% in 15 patients (28.3%). Significant respiratory variation of the superior vena cava (deltaSVC > 20%) was observed in 6 patients (11.3%), suggesting hypovolemia. 14 patients (26.5%) had an acute cor pulmonale (ACP) (RV/LV end-diastolic area > 0.6 associated with paradoxical septal motion). In the remaining 18 patients (34%), echo had a normal pattern, suggesting vasoplegia. For all of them, pulmonary acceleration time was shortened (81 [66–103]cm) suggesting pulmonary hypertension in these patients. Incidence of ACP and normal echo pattern significantly increased between D_1–2_ and D_3–7_, from 19% to 32% for ACP, and from 32% to 50% for normal echo pattern. In the same time, echo pattern suggesting hypovolemia and low LVEF were less frequent. In the 14 patients with ACP, 6 had a CT-scan in which pulmonary embolism was diagnosed in 3 patients. TEE visualized a clot into pulmonary arteries in 4 more patients. Echo parameters were normal in patients without catecholamines.

**Conclusion:** Different echocardiographic patterns were observed during the first week following ICU admission in COVID-19 patients, while echo suggested pulmonary hypertension in most patients. ACP was frequent, especially after few days, while LV systolic dysfunction was the most abnormal echo pattern at admission.

**Compliance with ethics regulations:** Yes in clinical research.
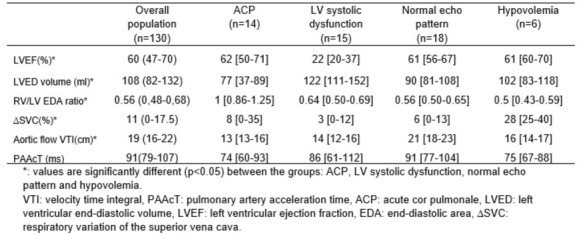


Main parameters of echocardiography when performed during D1–7 for the overall population and in the 53 patients who needed catecholamines

### CO-044 COVID-19 ARDS is characterized by higher lung water and permeability than non-COVID-19 ARDS. The PiCCOVID study

#### Rui Shi^1^, Christopher Lai^1^, Jean-Louis Teboul^1^, Martin Dres^2^, Francesca Moretto^1^, Nello De Vita^2^, Tài Pham^1^, Vincent Bonny^2^, Julien Mayaux^2^, Rosanna Vaschetto^3^, Alexandra Beurton^2^, Xavier Monnet^1^

##### ^1^Université Paris-Saclay, AP-HP, Service de médecine intensive-réanimation, Hôpital de Bicêtre, DMU CORREVE, Inserm UMR S_999, FHU SEPSIS, Groupe de recherche clinique CARMAS, Le Kremlin-Bicêtre, France, Le Kremlin-Bicêtre, France; ^2^AP-HP, Groupe Hospitalier Universitaire APHP-Sorbonne Université, site Pitié-Salpêtrière, Paris, France; ^3^Università del Piemonte Orientale, Anestesia e Terapia Intensiva, Azienda Ospedaliero Universitaria “Maggiore Della Carità”, Novara, Italie

**Correspondence:** Rui Shi - rui.shi@u-psud.fr

*Annals of Intensive Care* 2021, **11(Suppl 1):**CO-044

**Rationale:** The characteristics of COVID-19-related acute respiratory distress syndrome (ARDS) regarding extravascular lung water index (EVLWi) and pulmonary vascular permeability index (PVPI) have been scarcely described, although these parameters could be related to respiratory severity and fluid management. The aim of our study is to compare EVLWi, PVPI, respiratory mechanics and hemodynamics in patients with COVID-19 vs. non-COVID-19 ARDS (Trials registration: NCT04337983).

**Patients and methods/materials and methods:** This is an observational study conducted in three intensive care units (ICUs) from different university hospitals between March and October 2020. Sixty patients with COVID-19 ARDS monitored by transpulmonary thermodilution were consecutively included. In the non-COVID-19 group, we retrospectively selected 60 patients as in the COVID-19 group, consecutively hospitalized immediately before the first COVID-19 patient. Measurements were performed at least once a day. In all patients, we collected the transpulmonary thermodilution and mechanical ventilation variables corresponding to the maximum value of EVLWi and PVPI measured in the day.

**Results:** Driving pressure and the respiratory system compliance was similar between COVID-19 and non-COVID-19 ARDS, at baseline as during the study period. Extravascular lung water index was higher in COVID-19 than in non-COVID-19 ARDS, both at the baseline (17 (14–21) vs. 15 (11–19) mL/kg, respectively, *p* = 0.03) and at the time of its maximal value during the study period (24 (18–27) vs. 21 (15–24) mL/kg, respectively, *p* = 0.01). Similar results were observed for PVPI (baseline: 3.5 (2.9–4.5) vs. 2.8 (2.2–3.7), *p* = 0.01; maximal values: 4.6 (3.8–5.7) vs. 4.1 (3.3–4.8), *p* = 0.01). In COVID-19 patients, the worst ratio between arterial oxygen partial pressure over oxygen inspired fraction was lower (81 (70–109) vs. 100 (80–124), respectively, *p* = 0.02) and prone positioning (50 (83%) vs. 39 (65%), *p* = 0.04) and ECMO (17 (28%) vs. 7 (12%), *p* = 0.04) were more frequently used. COVID-19 patients had lower maximal lactate level (2.0 (1.6–2.7) vs. 2.7 (2.1–4.3), *p* < 0.01) and lower maximal norepinephrine dose (0.51 (0.16–0.89) vs. 1.01 (0.52–1.74), *p* < 0.01) than non-COVID-19 patients. Mortality was similar between two groups (57% vs. 65%, respectively, *p* = 0.45).

**Conclusion:** Compared to patients with non-COVID-19 ARDS, patients with COVID-19 ARDS had similar lung mechanics, but higher EVLWi and PVPI values from the beginning of the disease. This was associated with worse oxygenation and with more prone positioning and ECMO use and is compatible with the specific lung inflammation and severe diffuse alveolar damage in COVID-19 ARDS. By contrast, patients with COVID-19 ARDS had fewer hemodynamic derangements. Mortality was not different between groups.

**Compliance with ethics regulations:** Yes in clinical research.

### CO-045 90-day mortality and complications of right axillary versus femoral artery cannulation for veno-arterial extracorporeal membrane oxygenation: a propensity score analysis

#### Julien Do Vale, Grégory Papin, Elie Kantor, Romain Sonneville, Walid Ghodhbane, Angelo Pisani, Dan Longrois, Philippe Montravers, Sophie Provenchère

##### Hôpital Bichât - Claude Bernard, Paris, France

**Correspondence:** Julien Do Vale - julien.dovale@gmail.com

*Annals of Intensive Care* 2021, **11(Suppl 1):**CO-045

**Rationale:** Femoral artery cannulation is the preferential site for veno-arterial extracorporeal membrane oxygenation (femoro-femoral: FF-VA-ECMO) implantation. It leads to a retrograde aortic flow, which increases left ventricular afterload and could result in severe pulmonary oedema and thrombosis of cardiac chambers [1]. Right axillary artery cannulation (femoral–axillary: FA-VA-ECMO) provides an anterograde aortic flow [2], which could prevent some of these complications. The aim of this study was to compare 90-day mortality and complications rate of FA-VA-ECMO and FF-VA-ECMO.

**Patients and methods/materials and methods:** All consecutive adult patients with cardiogenic shock and peripheral VA-ECMO between 2013 and 2019 at our institution were retrospectively included. Exclusion criteria were refractory cardiac arrest, second VA-ECMO implantation for vascular access changes, weaning failure or ICU readmission. The probability of femoral *versus* axillary cannulation was modeled via logistic regression considering baseline characteristics. Inverse probability of treatment weighting (IPTW) was used to adjust for differences between groups for all outcome assessments. The primary endpoint was 90-day mortality. Secondary endpoints were vascular access complications, strokes, and other complications associated with retrograde blood flow. Outcomes were estimated by logistic regression. Results are expressed as OR (95%CI).

**Results:** VA-ECMO was implanted in 555 patients. Patients in refractory cardiac arrest (*n* = 77(14%)) and those assisted by a second VA-ECMO (*n* = 145(26%)) were excluded. Out of the studied 333 patients (*n* = 209 FA-VA-ECMO; *n* = 124 FF-VA-ECMO), main indications for VA-ECMO implantation were: post cardiotomy 33% (*n* = 109), dilated cardiomyopathy 20% (*n* = 66), post cardiac transplantation 15% (*n* = 50), acute myocardial infarction 14% (*n* = 46) and other etiologies 18% (*n* = 62). The median SOFA score was 9[7–11] and the crude 90-day mortality was 53% (*n* = 175). After IPTW, 90-day mortality was similar in FA-VA-ECMO and FF-VA-ECMO groups (59% versus 54%, IPTW-OR = 0.84[0.54–1.29], *p* = 0.55). Secondary outcomes are presented in Table 1.

**Conclusion:** As compared to FF-VA-ECMO, axillary cannulation was associated with similar 90-day mortality, but significantly fewer local infections, limb ischemia, bowel ischemia and pulmonary oedema, and higher risk of stroke. The higher rate of stroke with FA-VA-ECMO requires further exploration.

**References**

1. Combes A, et al. Lancet Lond Engl. 2020;396(10245):199–212.
2. Andrei S, et al. Artif Organs. 2019;43(7):647–55.

**Compliance with ethics regulations:** Yes in clinical research.
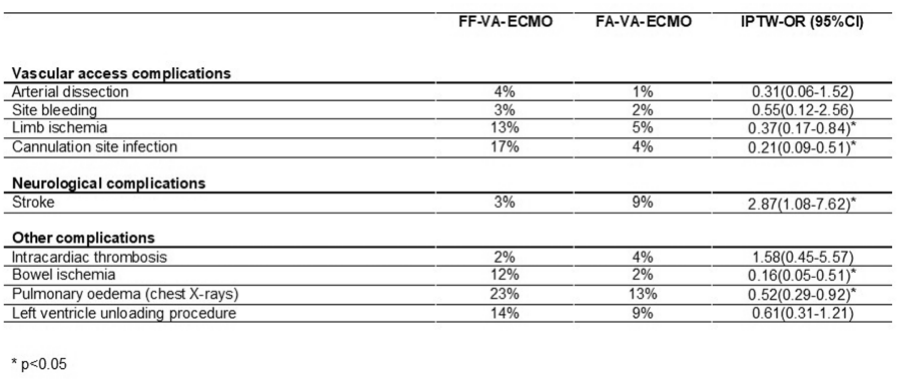


Table 1 VA-ECMO-related secondary outcomes in the IPTW population

### CO-046 Epinephrine versus norepinephrine for patients with shock after cardiac arrest

#### Kaci Slimani^1^, Wulfran Bougouin^1,2^, Marie Renaudier^2^, Yannick Binois^2,9^, Marine Paul^3^, Florence Dumas^2^, Lionel Lamhaut^2^, Thomas Loeb^4^, Eric Lecarpentier^5^, Sofia Ortuno^9^, Nicolas Deye^6^, Sebastian Voicu^6^, Frankie Beganton^2^, Daniel Jost^7^, Armand Mekontso-Dessap^8^, Eloi Marijon^2^, Xavier Jouven^2^, Nadia Aissaoui^9^, Alain Cariou^2^

##### ^1^Hôpital Jacques Cartier, Massy, France; ^2^Paris Sudden Death Expertise Center, Paris, France; ^3^Centre Hospitalier de Versailles - Site André Mignot, Le Chesnay, France; ^4^SAMU des Hauts-de-Seine, APHP – Université Paris-Saclay, Hôpital Raymond Poincaré, Garches, France; ^5^Hôpital Henri Mondor, SAMU 94, Créteil, France; ^6^Hôpital Lariboisière, Paris, France; ^7^BSPP, Paris, France; ^8^Hôpital Henri Mondor, Créteil, France; ^9^HEGP, Paris, France

**Correspondence:** Wulfran Bougouin - wulfran.bougouin@gmail.com

*Annals of Intensive Care* 2021, **11(Suppl 1):**CO-046

**Rationale:** Despite a return of spontaneous circulation (ROSC), 50 to 70% of patients admitted alive to ICU after out-of-hospital cardiac arrest (OHCA) die during their ICU stay. Apart from anoxo-ischemic brain damage, many deaths result from post-resuscitation shock and multiple organ failure (MOF). So far, no study compared vasopressors in the setting of post-OHCA shock. Accordingly, we compared outcome of OHCA patients with post-resuscitation shock, according to the vasopressor used (epinephrine or norepinephrine).

**Patients and methods/materials and methods:** We identified patients admitted after OHCA in 5 hospitals between May 2011 and May 2018. We included all consecutive adult patients with post-resuscitation shock (arterial hypotension requiring a continuous infusion of epinephrine or norepinephrine). Exclusion criteria were extracardiac cause of OHCA, refractory OHCA, treatment with extracorporeal membrane oxygenation, patients who received neither epinephrine nor norepinephrine, or patients treated with both treatments simultaneously. Primary endpoint was in-hospital all-cause mortality. Secondary endpoints were cardiovascular-specific mortality (recurrent cardiac arrest or refractory shock and MOF), and unfavorable neurological status at hospital discharge (CPC 3–5).

**Results:** Among 4918 OHCA included in the registry over the study period, 1421 were admitted in participating hospitals and developed a post-resuscitation shock. Finally, 766 patients were included, 481 (63%) treated with norepinephrine, and 285 (37%) with epinephrine. 73% of patients were male, and median age was 64 (IQR = 52–75). Median no-flow and low-flow times were 5 min and 22 min. Patients treated with epinephrine infusion had lower proportion of shockable rhythm (44% vs 57%, *P* < 0.001), longer low-flow time (25 vs 20 min, *P* < 0.0001), lower arterial pH at admission (7.17 vs 7.23, *P* < 0.0001). Overall, 235 patients survived to hospital discharge (31%), with favorable neurological outcome in 98% of survivors. As compared with norepinephrine, patients treated with epinephrine infusion had higher all-cause mortality (83% vs 61%, *P* < 0.001), higher cardiovascular-specific mortality (44% vs 11%, *P* < 0.001) and higher rates of unfavorable neurological outcome (85% vs 63%, *P* < 0.001), Fig. After multivariable adjustment for confounders (sex, age, bystander CPR, initial shockable rhythm, no-flow, low-flow, epinephrine dose during resuscitation (before ROSC), arterial pH, myocardial dysfunction, targeted temperature management, percutaneous coronary intervention), epinephrine infusion was independently associated with all-cause mortality (OR = 2.6, 95%CI = 1.4–4.7, *P* = 0.002), cardiovascular-specific mortality (OR = 5.5, 95%CI = 3.0–10.3, *P* < 0.001), and unfavorable neurological outcome (OR = 3.0, 95%CI = 1.6–5.7, *P* = 0.001). Similar results were found after exclusion of moribund patients (deaths < 12 h after admission), and after propensity-score analysis.

**Conclusion:** In this population-based study, using epinephrine infusion to manage post-resuscitation shock was associated with an increased all-cause and cardiovascular-specific mortality, whatever the methodological approach.

**Compliance with ethics regulations:** Yes in clinical research.
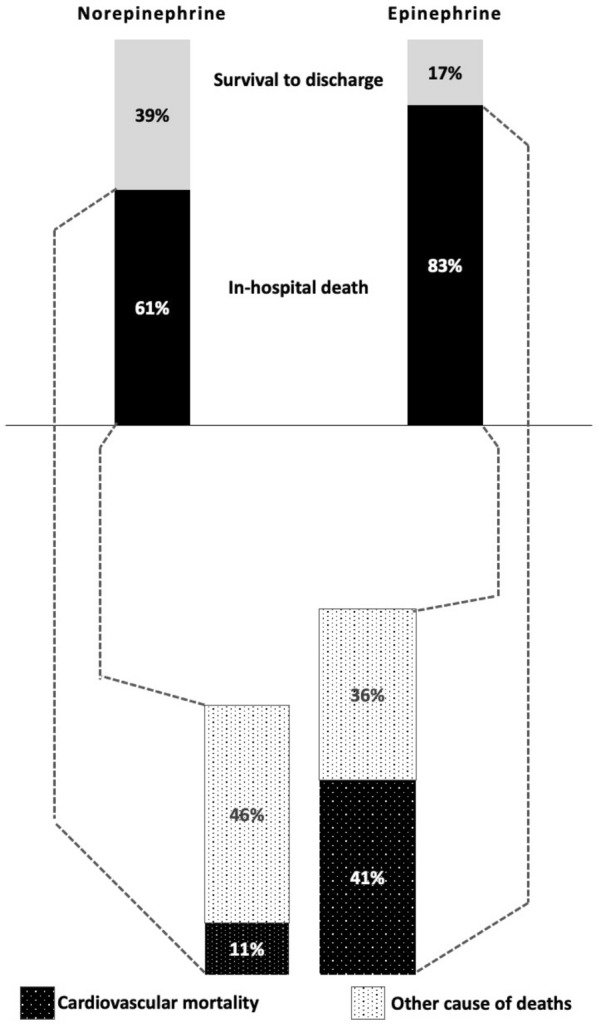


Short-term outcome of patients according to vasoactive medication used

### CO-047 Association between mean arterial pressure level and acute kidney injury in patients with out-of-hospital cardiac arrest

#### Vincent Dupont^1,6^, Anne-Sophie Bonnet-Lebrun^2^, Alice Boileve^3^, Julien Charpentier^4^, Jean-Paul Mira^4,7^, Guillaume Geri^5,8,9^, Alain Cariou^4,7,10,11,12^, Mathieu Jozwiak^4,7^

##### ^1^Centre Hospitalier Universitaire de Reims, Reims, France; ^2^British Antarctic Survey, Cambridge, Royaume-Uni; ^3^Institut Gustave Roussy, Villejuif, France; ^4^Hôpital Cochin, Paris, France; ^5^Hôpital Ambroise Paré, Reims, France; ^6^French Clinical Research Infrastructure Network, Investigation Network Initiative–Cardiovascular and Renal Clinical Trialists (F-CRIN INI-CRCT), Nancy, France; ^7^Université de Paris, Paris, France; ^8^Université Paris-Saclay, Paris, France; ^9^INSERM UMR1018, Centre de recherche en Epidémiologie et Santé des Populations, Villejuif, France; ^10^INSERM U970, Paris-Cardiovascular-Research-Center, Paris, France; ^11^Paris Sudden-Death-Expertise-Centre, Paris, France; ^12^AfterROSC Network Group, Paris, France

**Correspondence:** Vincent Dupont - vince_zen@hotmail.com

*Annals of Intensive Care* 2021, **11(Suppl 1):**CO-047

**Rationale:** The post-resuscitation syndrome after out-of-hospital cardiac arrest (OHCA) results in multiple organ failure and increases both morbidity and mortality among survivors of OHCA. Acute kidney injury (AKI) occurs in 10 to 80% of patients with OHCA and is associated with long-term occurrence of chronic kidney disease as well as poor neurological outcomes and increased mortality. The previously described risk factors of AKI in patients with OHCA are not modifiable once the patient is admitted in the ICU which limits the development of interventional strategies (Reference 1). Nevertheless, the optimal mean arterial pressure (MAP) target after OHCA in terms of renal function remains to be established (Reference 2). We aimed to evaluate the effects of early MAP level on AKI incidence within the first 48 h after intensive care unit (ICU) admission in patients with OHCA

**Patients and methods/materials and methods:** In 568 consecutive patients with OHCA, the percentage time spent below a given MAP threshold and the area below threshold (ABT), which reflects both the duration and the severity of hypotension were calculated from continuous MAP measurement. Both MAP-derived variables were calculated for different MAP thresholds (65, 75 and 85 mmHg) and time periods (within the first 6, 12 and 24 h after ICU admission). AKI was defined as stage 3 of the Acute Kidney Injury Network classification

**Results:** Among the 568 patients included in the analysis (71% men, median age 59), 274 (48%) developed AKI within the first 48-h after ICU admission. Both ABT and percentage time were independently associated with AKI, regardless of MAP thresholds and time periods (Table 1). Within the first 24 h, every 100 mmHg/h increase in ABT, the risk of AKI increased by 17% (OR = 1.17; 95%CI: 1.08–1.29; *p* < 0.01), 2% (OR = 1.02; 95%CI: 1.01–1.03; *p* < 0.01) and 1% (OR = 1.01; 95%CI: 1.00–1.01; *p* < 0.01) for MAP thresholds of 65, 75 and 85 mmHg. Every 36 min (i.e. every 10% increase in percentage time) spent under MAP thresholds of 65, 75 and 85 mmHg increased the risk of AKI by 23% (OR = 1.23; 95%CI: 1.09–1.38; *p* < 0.01), 13% (OR = 1.13; 95%CI: 1.05–1.21; *p* < 0.01) and 8% (OR = 1.08; 95%CI: 0.99–1.18; *p* = 0.10)

**Conclusion:** Both severity and duration of hypotension within the first 24 h after ICU admission were independently associated with AKI for MAP thresholds ranging from 65 to 85 mmHg

**References**

1. Geri G, Guillemet L, Dumas F, et al. Acute kidney injury after out-of-hospital cardiac arrest: risk factors and prognosis in a large cohort. Intensive Care Med. 2015; 41:1273–1280. 10.1007/s00134-015-3848-4.
2. Jozwiak M, Bougouin W, Geri G, et al. Post-resuscitation shock: recent advances in pathophysiology and treatment. Ann Intensive Care. 2020; 10:170. 10.1186/s13613-020-00788-z.

**Compliance with ethics regulations:** Yes in clinical research.
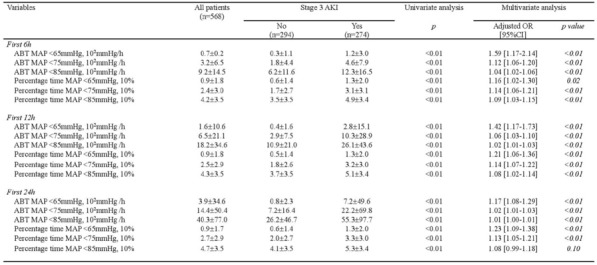


Variables are expressed as mean ± standard deviation. AKI: acute kidney injury, ABT: area below threshold, CI: confidence interval, MAP: mean arterial pressure

### CO-048 Post-cardiac arrest myocardial dysfunction: prevalence and influence on long-term outcome

#### Sofia Ortuno^1^, Wulfran Bougouin^2^, Marine Paul^4^, Jean Baptiste Lascarrou^3^, Florence Dumas^1^, Nicole Karam^1^, Eloi Marijon^1^, Xavier Jouven^1^, Alain Cariou^1^, Nadia Aissaoui^1^

##### ^1^APHP, Paris, France; ^2^Hôpital Jacques Cartier, Massy, France; ^3^CHU Nantes, Nantes, France; ^4^Hôpital André Mignot, Versailles, France

**Correspondence:** Sofia Ortuno - ortunosofia1@gmail.com

*Annals of Intensive Care* 2021, **11(Suppl 1):**CO-048

**Rationale:** Post-resuscitation shock is a frequent complication of cardiac arrest and is responsible for about half of in-hospital mortality. This shock is defined by a vasoplegic state associated with a myocardial dysfunction which is supposed to recover without sequelae. We aimed to assess the prevalence of myocardial dysfunction in out-of-hospital cardiac arrest patients, discharged alive from hospital and the long-term prognosis consequences in terms of cardiovascular events and mortality at 5 years.

**Patients and methods/materials and methods:** Using the Sudden Death Expertise Center registry (SDEC, Great Paris area), we focused on 3 tertiary centers (Cochin hospital, European Georges Pompidou hospital and Lariboisiere hospital) between May 2011 to November 2015 and assessed all patients successfully resuscitated after out-of-hospital cardiac arrest and discharged alive from hospital, whatever the etiology. The primary end-point was the incidence of post-resuscitation myocardial dysfunction (PRMD) at echocardiography defined as decreased LVEF below 40% during the post-resuscitation period. The secondary end-point was long-term major adverse cardiovascular events (MACE), including acute coronary events, cerebrovascular events, major vascular events in other localization, acute heart failure, and deaths. They were collected from the SNIIRAM database. We defined 2 groups according to the presence of a PRMD and compared them. We performed multivariate analysis to identify predictor factor of long-term cardiovascular prognosis.

**Results:** Among 763 patients discharged alive from the SDEC, 330 were managed in the 3 hospitals and 260 (mean age 57 (± 16), male *n* = 201, 77%) were included. 221 (85%) were CPC 1 or 2 at hospital discharge. Median of follow-up was 6 years for mortality and 3 years for MACE. The median LVEF at ICU admission was 40% [30–50] and 136 patients (52%) developed shock. 153 (59%) patients have PRMD at admission, with median LVEF of 30% [25–35]. MACE (including death) occurred in 84 patients (32%) with 39 deaths and hospital readmission for heart failure in 43 patients (17%). The incidence of MACE (including death) was not different in the group of patients with PRMD as compared with those without PRMD [Fig. 1]. The occurrence of PRMD was not associated with a worse cardiovascular prognosis whereas a previous history of heart failure or coronary diseases was associated with increased MACE (including death) occurrence during follow-up.

**Conclusion:** In survivors, the occurrence of a myocardial dysfunction after cardiac arrest was not associated with an increase in MACEs during long-term follow-up.

**References**

1. Lemiale V, Dumas F, Mongardon N, Giovanetti O, Charpentier J, Chiche J-D, et al. Intensive care unit mortality after cardiac arrest: the relative contribution of shock and brain injury in a large cohort. Intensive Care Med. 2013;39(11):1972–80.
2. Laurent I, Monchi M, Chiche J-D, Joly L-M, Spaulding C, Bourgeois B, et al. Reversible myocardial dysfunction in survivors of out-of-hospital cardiac arrest. J Am Coll Cardiol. 2002;40(12):2110–6.

**Compliance with ethics regulations:** N/A.
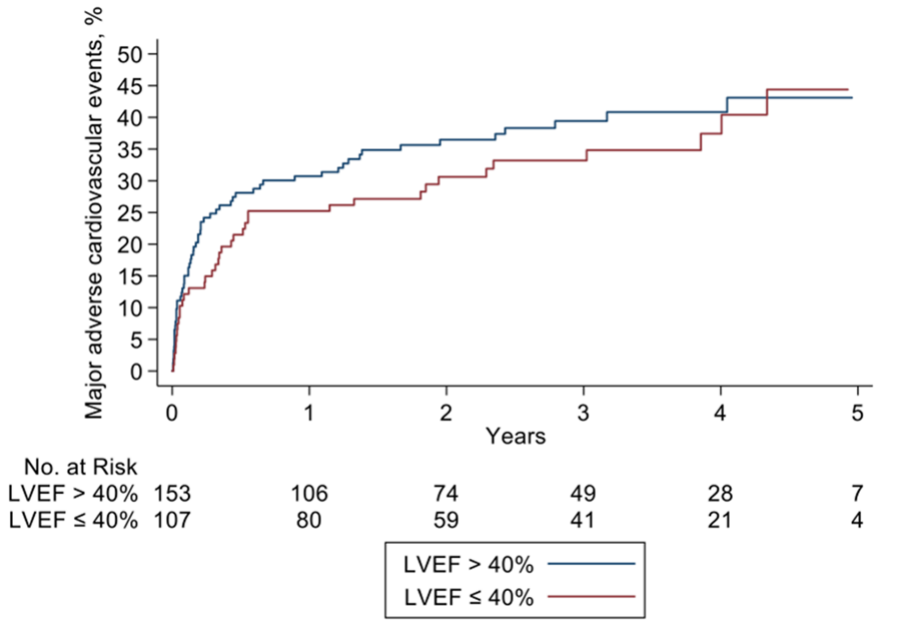


Long-term cardiovasculars events including death according to the occurrence of post-resuscitation myocardial dysfunction at the ICU admission

### CO-049 Severe cardiovascular features in SARS-CoV-2 related multisystem inflammatory syndrome in children: a single-center descriptive study of 58 critically ill children

#### Charles De Marcellus, Judith Chareyre, Marie Pouletty, Zahra Belhadjer, Lucile Houyel, Julie Toubiana, François Angoulvant, Marianne Leruez-Ville, Sylvain Renolleau, Mehdi Oualha, Florence Moulin, Marion Grimaud

##### ^1^Hôpital Necker, Paris, France

**Correspondence:** Charles De Marcellus - charles.de-marcellus@aphp.fr

*Annals of Intensive Care* 2021, **11(Suppl 1):**CO-049

**Rationale:** SARS CoV-2-related multisystem inflammatory syndrome in children (MIS-C) has been increasingly recognized. However, the clinical features of severe cardiovascular involvement merit further accurate description.

**Patients and methods/materials and methods:** This is an observational study conducted in a PICU in a tertiary hospital between April 1st and December 31st, 2020. Patients aged less than 18 years with SARS-CoV-2-related MIS-C and shock, according to the Centers for Disease Control and Prevention criteria were included. Demographics, clinical, biological data including inflammatory markers were collected.

**Results:** 58 critically ill children (median age of 9 years [interquartile range (IQR) 6.9–11.7]) admitted for shock had an acute myocarditis (left ventricular ejection fraction, 44% [IQR 35–50] troponin, 217 ng/mL [IQR 89–455]) and 15 patients exhibited a distributive component of shock. SARS-CoV-2 real-time polymerase chain reaction positive in 15 and antibody positive in 56 (96%) patients. Fever was present in all patients, as well as gastrointestinal symptoms (97%). Inflammatory markers C-reactive protein [257 mg/dL (IQR 209–339)], procalcitonin [24 ng/mL (IQR 9–55)], and serum interleukin-6 levels [131 pg/mL (IQR 28–351)] were uniformly elevated without documented bacteria infection. At least one feature of Kawasaki disease was found in all children, but none had the typical form. Only 11 patients (19%) required invasive mechanical ventilation. Renal involvement was present in 34 patients (59%). Diastolic dysfunction was noted in 18 patients (31%) with right ventricular dysfunction in 28 (48%). 50% of patients required resuscitative fluid infusion and 42 patients (72%) needed inotropic or vasoactive drug support therapy (epinephrine, *n* = 15 milrinone, *n* = 13; dobutamine, *n* = 26, norepinephrine, *n* = 9). None of the patients required extracorporeal membrane oxygenation. Almost all children (98%) received intravenous immunoglobulin (2 g per kilogram) and 37 patients received corticosteroids. IL 1 receptor antagonist was used in three patients. All children survived and were afebrile with a full left ventricular function recovery at PICU discharge. The median length of stay in PICU was 4 days [IQR 3–5].

**Conclusion:** Children with SARS-CoV-2-related MIS-C admitted at the PICU had circulatory shock including systolic and diastolic myocardial dysfunction and half of them had vasodilatory component. MIS-C with shock should be distinguished from Kawasaki disease shock syndrome, septic shock and viral myocarditis. Early recognition and aggressive management of shock and immune modulation with methyl-prednisolone and intravenous immunoglobulin are the keys of the treatment. Favourable short-term outcomes seems to be the rule.

**Compliance with ethics regulations:** Yes in clinical research.

### CO-050 COVID-19-related lockdown impacts incidence of infantile subdural hematoma: a single-center retrospective study

#### Alina Marilena Lazarescu, Sandro Benichi, Kevin Beccaria, Estelle Vergnaud, Juliette Montmayeur, Thomas Blauwblomme, Gilles Orliaguet

##### Hopital Necker Enfants Malades, Paris, France

**Correspondence:** Alina Marilena Lazarescu - alina.lazarescu@aphp.fr

*Annals of Intensive Care* 2021, **11(Suppl 1):**CO-050

**Rationale:** In case of infantile subdural hematomas (SDH), the French High Authority of Health provided in 2010 and 2017 evidence-based medicine guidelines to recognize “shaking” as the etiology. This ‘shaken baby syndrome’ (SBS) is the most common cause of trauma related mortality before 1 year of age. Risk factors for SDH may be linked to the infant (male, preterm, multiple pregnancies, difficulties with feeding and crying) or the parents (i.e. psychiatric disorders, social insecurity or isolation). In 2020, French authorities ordered a large lockdown to hinder the spread of COVID-19 pandemics in France from 17 March to 10 May, then from 30 October to 15 December, immediately followed by curfew that is still underway. As parents and children had to remain isolated in their households for prolonged period, we hypothesized that this unseen social fact may affect the incidence of SBS.

**Patients and methods/materials and methods:** We retrospectively reviewed the medical records of all patients admitted for SDH, between February 2017 and the end of January 2021, in our unit, a tertiary center where all SDH requiring Intensive Care Unit (ICU) admission and/or neurosurgical expertise are referred across the whole Ile-de-France region (12.2 million inhabitants). We compared the annual hospitalization rates before (2017/02/01–2020/01/31) and during the COVID19 crisis (2020/02/01–2021/31/01) using Fisher test for proportion and log-rank test for survival analysis. We analyzed annual incidence for SBS before and during the COVID-19 crisis as well as the effect of the first (March–April 2020) and second (November–December 2020 and January 2021) lockdown on SBS rates.

**Results:** Eighty-six patients fulfilled the criteria and were included in the analysis. From February 1st to January 31st, 24, 18 and 17 infants were, respectively, admitted in 2017-18-19, and 23 infants over 2020. There was no difference in the annual prevalence before and during the COVID-19 crisis (21 vs 23, *p* = 0.09). However, we observed a significant difference in the SDH distribution (*p* = 0.005) and in the age at diagnosis (176d vs 130d, *p* = 0.03). Indeed, SDH prevalence was null during the first lockdown (0 vs 5, *p* = 0.004), whereas it dramatically increased during the second lockdown and curfew (13 vs 5.3, *p* = 0.03).

**Conclusion:** COVID-19 related lockdown influenced the prevalence of SDH in the Ile-de-France region. This study provides insufficient data to precisely explain this phenomenon; however, our hypothesis is that reduced social interactions, a lack of social support, and newly arising stressors associated with the COVID-19 crisis increased the risk for SBS.

**References**

1. Laëtitia C. Haute Autorité de santé. 2017;46.
2. Dias MS, Rottmund CM, Cappos KM, Reed ME, Wang M, Stetter C, et al. Association of a Postnatal Parent Education Program for Abusive Head Trauma With Subsequent Pediatric Abusive Head Trauma Hospitalization Rates. JAMA Pediatr. 2017;171(3):223–9.

**Compliance with ethics regulations:** Yes in clinical research.
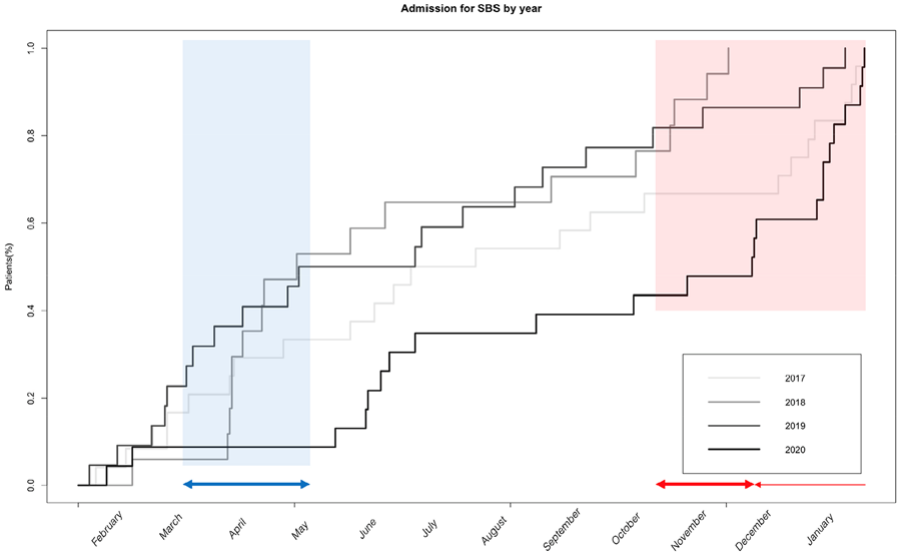


Prevalence of HSD before and durind Covid crisis

### CO-051 Transforming a paediatric ICU to an adult ICU for severe COVID-19: lessons learned

#### Maryline Chomton Cailliez, Lucile Marsac, Jerome Naudin, Anna Deho, Arielle Maroni, Guillaume Geslain, Geraldine Poncelet, Fleur Lebourgeois, Laure Maurice, Boris Lacarra, Karine Haverland, Armelle Nicolas Robin, Stéphane Dauger, Michael Levy

##### HOPITAL ROBERT DEBRE, Paris, France

**Correspondence:** Maryline Chomton Cailliez - maryline.chomton@aphp.fr

*Annals of Intensive Care* 2021, **11(Suppl 1):**CO-051

**Rationale:** During the first COVID-19 wave, our paediatric intensive care unit (PICU), like many others across the globe, was transformed to an adult ICU for patients with severe Covid-19 to overcome the shortage of ICU beds. We provide a detailed description of all the requirements we had to fulfil and share the lessons we learned when we set up an adult ICU within a paediatric hospital.

**Patients and methods/materials and methods:** Presentation of setting, equipment and supplies which had been required organizing this transformation. Description of human resources and organisation set up to assure an adult and paediatric intensive care activity in paediatric hospital, in France.

**Results:** Strong support from all hospital departments was crucial, as their activity was modified by the change. Healthcare workers from various units, notably the paediatric anaesthesiology department, worked in the adult ICU to ensure sufficient staffing. The adult bed capacity was increased from 8 to 18 within a single week. Fifteen PICU beds were set up in the emergency unit. We had enough ventilators in stock in the hospital to allow ventilation of all patients in both units. The number of physiotherapists and psychologists was increased. A support system for both healthcare workers and patients’ relatives was set up with the help of the mobile paediatric palliative care and support team. Supplies suitable for adults were ordered. Protocols for numerous procedures were written within a few days. Video tutorials, checklists, and simulation sessions realized by the PICU team were circulated to the entire staff. Head nurses guided and supported both new and usual PICU staff. The transformation was achieved within a week. The main difficulties were healthcare worker stress, changes in recommendations over time, absence of visits from relatives, and specific adult issues that paediatricians are unfamiliar with.

**Conclusion:** Despite a very short period of implementation, strong multidisciplinary cooperation helped to identify the main issues and to maximize the efficiency of the change from paediatric to adult ICU. This experience was challenging, but also rewarding as an opportunity for commitment, sharing, and supporting one another. For the staff, caring for adult patients was made easier by working in their familiar unit instead of being moved to an adult hospital with unfamiliar staff members and equipment.

**Compliance with ethics regulations:** Yes in clinical research.

### CO-052 The emotional state of healthcare professionals confronted to end-of-life signs in a PICU

#### Coralie Pereira Da Silva^3^, Charlotte Pierron^1^, Fleur Lebourgeois^2^

##### ^1^Centre hospitalier du Luxembourg, Luxembourg, Luxembourg; ^2^Hôpital Robert Debré, Paris, France; ^3^Université Paris-Est Créteil - Centre d’étude des discours, images, textes, écrits, communication, Créteil, France

**Correspondence:** Charlotte Pierron - charlottepierron@hotmail.com

*Annals of Intensive Care* 2021, **11(Suppl 1):**CO-052

**Rationale:** Few studies describe the end-of-life (EOL) signs in pediatric intensive care unit (PICU), but we know that some of these can be impressive or distressful for healthcare providers (HCPs) and parents.

**Patients and methods/materials and methods:** To evaluate the emotional state of HCPs confronted to EOL signs, we conducted a prospective study in a PICU where HCPs had an interview with a psychologist. These interviews were done following the predictable death of a child, allowing HCPs to inform parents about the upcoming death and likelihood of EOL signs. These deaths occured after a withdrawal of life-sustaining treatment or not. Following a sociological qualitative approach, their interviews were transcribed and encoded inductively to be analysed.

**Results:** Fiveteen HCPs participated to the study: 6 nurses, 4 physicians, 3 residents and 2 assistant nurses. The interviews took place 35.5 h (mean) after the child’s death, for a mean length of 15 min (9 to 27 min). The more stressful EOL signs mentioned were those which could be interpreted as a sign of the child’s presence (gasping or breathing, movements, etc.) and those which modify the child’s appearance and the last image given to its parents (changes of color, bleeding, etc.). More than the EOL signs, the parents’ reaction to them were feared by the HCPs. The distress and suffering of the parents constituted the major part of the violence felt by the HCPs facing EOL signs, especially gasping. Another parameter influencing the HCPs’ feeling was the duration of the signs. In contacts informing the parents before the onset of the signs is a way to reduce the HCPs distress. Questioned about the information to be given to the parents, most of the HCPs said that providing standard written information to the parents would be dehumanizing and violent. Finally, the HCPs declared to find support at their colleagues when the situation is unbearable.

**Conclusion:** HCPs confronted with EOL signs in PICU felt sometimes these situations violent, mainly following the parents’ reactions. These reactions could be relieved by anticipated information about EOL signs to the parents.

**Compliance with ethics regulations:** N/A.

### CO-053 Prevalence of pituitary dysfunction in children after traumatic brain injury: a prospective study in the acute phase and one year after trauma

#### Sonia Courtil-Teyssedre^1^, Aurelie Portefaix^2,3^, Benjamin Riche^4,5^, Berhouz Kassai-Koupai^2,3^, Fleur Cour-Andlauer^1^, Etienne Javouhey^1^

##### ^1^Hôpital femme Mère Enfant, Hospices Civils de Lyon, Bron, France; ^2^Centre d’Investigation Clinique, Hospices Civils de Lyon, Bron, France; ^3^Université Claude Bernard, CNRS, UMR 5558, Laboratoire de Biométrie et Biologie Evolutive, Villeurbanne, France; ^4^Pôle Santé Publique, Service de Biostatistique et Bioinformatique, Hospices Civils de Lyon, Lyon, France; ^5^CNRS, UMR 5558, Laboratoire de Biométrie et Biologie Evolutive, Équipe Biostatistique-Santé, Villeurbanne, France

**Correspondence:** Sonia Courtil-Teyssedre - sonia.teyssedre@chu-lyon.fr

*Annals of Intensive Care* 2021, **11(Suppl 1):**CO-053

**Rationale:** Traumatic brain injury (TBI) is a major cause of mortality and permanent disability in children. Almost 15% of them suffer from cognitive deficit and behavioural disorders, potentially related to pituitary dysfunction (PD). Aims of the study were to determine the prevalence of PD during the acute phase of TBI and 1 year later, and to investigate whether initial PD and severity of TBI may predict PD.

**Patients and methods/materials and methods:** Children hospitalized for moderate-to-severe TBI were included. Pituitary function was evaluated 1–2 days after trauma: serum and urine concentrations of electrolytes and osmolalities, cortisol and adrenocorticotropic hormone (ACTH) levels (from 8a.m to 8a.m), free urinary cortisol, free thyroxin and thyroid stimulating hormone (TSH), insulin-like growth factor, estradiol in girls and testosterone in boys (if pubertal development), follicle-stimulating hormone and luteinizing hormone urinary levels (if age > 10y). One year after TBI, the same tests were performed associated to dynamic tests to evaluate growth hormone (GH) secretion (betaxolol–glucagon ± clonidine test).

**Results:** One hundred-nine children (72 males, median age 7.69 years IQR [2.13–12.4]) were included. Thirty-four (31.2%) had at least one PD: 16 ACTH deficiency, 11 gonadotrophin deficiency and 9 TSH deficiency (2 combined deficiencies). Endocrine assessment was available in 94 children at 1 year. Twenty-three (24.4%) had at least one PD and 20 (21.2%) had GH deficiency. Others deficiencies were rare (2 ACTH deficiency, 3 TSH deficiency, 2 combined deficiencies). Among the 28 children with initial PD, 6 (21.4%) had PD at 1 year, compared to 17/66 (25.7%) of those without initial PD (NS). Children with GH deficiency at 1 year were significantly younger and had a lower weight than children without GH deficiency (3.95 years IQR [0.66–10.36] vs 8.57 [3.74–12.9], *p* = 0.045; 16 kg IQR [7.87–30] vs 27.8 [16–50], *p* = 0.031). We did not find any predictive trauma-related factors associated with existence of PD at 1 year (mechanism of TBI, polytrauma, Injury Severity Score, Glasgow Coma Scale, intracranial hypertension, neurosurgery).

**Conclusion:** Pituitary dysfunction occurs in 31% during the acute phase of TBI, particulary ACTH deficiency. At 1 year 24.4% have at least one PD, essentially GH deficiency. Deficiency during the acute phase and trauma-related factors are not predictive of PD at 1 year. A systematic endocrine assessment in acute phase and after 1 year after moderate or severe TBI in children is recommended.

**Compliance with ethics regulations:** Yes in clinical research.

### CO-054 Abuse head trauma in children: risk factors for poor neurological outcome in a multicenter cohort

#### Fanny Regeffe, Anne Millet, Alexandre Bellier, Guillaume Mortamet

##### CHU de Grenoble, Grenoble, France

**Correspondence:** Guillaume Mortamet - gmortamet@chu-grenoble.fr

*Annals of Intensive Care* 2021, **11(Suppl 1):**CO-054

**Rationale:** Infants with suspected or confirmed abuse head trauma (AHT) are commonly admitted in Pediatric Intensive Care Units (PICUs) at early stage of the disease and AHT remains the leading cause of brain injury in infants. However, the neurological outcome of these patients is scarcely described. This study aims to describe a cohort of patients with AHT and to identify early risk factors associated with a poor neurological outcome.

**Patients and methods/materials and methods:** A multi-center retrospective study was conducted over a 8-year period (from January 2012 to December 2020). Children under 1 year old admitted in PICU with a certain or probable diagnosis of AHT were included. The neurological outcome was assessed by Pediatric Overall Performance Category score (POPC) at discharge from hospital and at 2 years.

**Results:** A total of 117 patients (mean age 4.3 (± 2.5) months, 61% boys) from 3 PICUs were included in our cohort. The average Glasgow score at admission was 11. Cerebral imagery found a subdural hematoma in 112 (96%), a tadpole sign in 80 (68%), a diffuse hypoxic brain injury in 45 (38%) and a cerebral atrophy in 32 (27%). Retinal haemorrhages were found in 92 (79%) patients. At 2 years follow-up, 23 patients (20%) had multiple severe disabilities (20%). The main neurological sequelae were neurodevelopmental (*n* = 38, 35%) or hyperactivity disorder (*n* = 36, 33%), epilepsy (*n* = 34, 31%) and motor deficit (*n* = 28, 26%). A total of 40 patients (37%) suffered from ophthalmologic sequelae. At discharge, 77 patients (72%) were placed in care with an average duration of 11 (± 18) months. After multivariate analysis, only the presence of cardiorespiratory arrest and the low Glasgow score stand out as factors of poor outcome.

**Conclusion:** In children with AHT, the occurence of cardiorespiratory arrest before or during stay and a low Glasgow score at admission were found to be predictors of poor short and long-term neurological outcome.

**Compliance with ethics regulations:** Yes in clinical research.

### CO-055 Potential for lung recruitment in patients with SARS-CoV2-associated acute respiratory distress syndrome: a multicenter study

#### Tài Pham^1^, Clément Brault^2^, François Beloncle^3^, Margot Combet^1^, Anne-Fleur Haudebourg^4^, Maeva Rodriguez^5^, Martin Dres^6^, Lise Piquilloud^7^, Yoann Zerbib^2^, Bertrand Pavlosky^3^, Vincent Bony^6^, Rémi Coudroy^5^, Guillaume Carteaux^4^

##### ^1^Hôpital Bicêtre, Le Kremlin-Bicêtre, France; ^2^CHU Amiens-Picardie, Amiens, France; ^3^CHU d’Angers, Angers, France; ^4^CHU Henri Mondor, Créteil, France; ^5^CHU de Poitiers, Poitiers, France; ^6^GHU site Pitié Salpêtrière, Paris, France; ^7^CHU Vaudois, Lausanne, Suisse

**Correspondence:** Tài Pham - tai.pham@aphp.fr

*Annals of Intensive Care* 2021, **11(Suppl 1):**CO-055

**Rationale:** Respiratory mechanics and potential for lung recruitability of COVID-19-related acute respiratory distress syndrome (C-ARDS) have been variably reported in small series. As their knowledge may provide valuable information to guide ventilation personalization, we aimed at describing respiratory system characteristics in a larger cohort of intubated patients with C-ARDS and identifying factors associated with recruitability.

**Patients and methods/materials and methods:** This is a multicenter observational study performed in 6 ICUs in France. We included intubated C-ARDS patients. Within 72 h after intubation, respiratory mechanics, lung recruitability and airway opening pressure (AOP) were collected. Low respiratory system compliance was defined as < 40 ml/cmH2O. Lung recruitability was assessed using recruitment-to-inflation ratio (R/I), obtained with a drop in PEEP over a single breath maneuver, as previously described [1]. Patients were deemed high recruiters if R/I > 0.5. AOP was assessed with a low-flow inflation.

**Results:** A total of 190 patients were included, 78% were men, with a mean (SD) age of 61 (13), BMI of 29.7 (6.2) kg/m^2^, SAPS2 of 41 (15), and SOFA of 6 (3). Their median [IQR] duration of invasive ventilation was 14 [9;27] days and 57% were discharged alive from the ICU. A total of 103 patients (55%) exhibited a low respiratory system compliance and an AOP above 5 cmH2O was retrieved in 40 (23%) patients. A total of 102 patients (54%) were high recruiters with a R/I above 0.5. Comparisons between high recruiters and low recruiters are shown in the table. The mean expired volume in a single breath maneuver was 1061 (289) mL and did not differ between both groups (*p* = 0.103). There was no statistical difference except absolute lower tidal volume being and higher PEEP setting in the recruiters. When tidal volumes were standardized to predicted body weight, the difference was no longer significant.

**Conclusion:** In a cohort of 190 C-ARDS patients receiving invasive mechanical ventilation, approximately half were deemed highly recruitable and 23% presented an AOP > 5 cmH2O within 72 h after intubation. We could not identify specific parameters associated with recruitability. This emphasizes the importance of measuring respiratory mechanics and assessing recruitability to personalize mechanical ventilation in this population.

**Reference**

1. Chen L, Del Sorbo L, Grieco DL, Junhasavasdikul D, Rittayamai N, Soliman I, et al. Potential for Lung Recruitment Estimated by the Recruitment-to-Inflation Ratio in Acute Respiratory Distress Syndrome. A Clinical Trial. Am J Respir Crit Care Med. 2020;201.

**Compliance with ethics regulations:** Yes in clinical research.
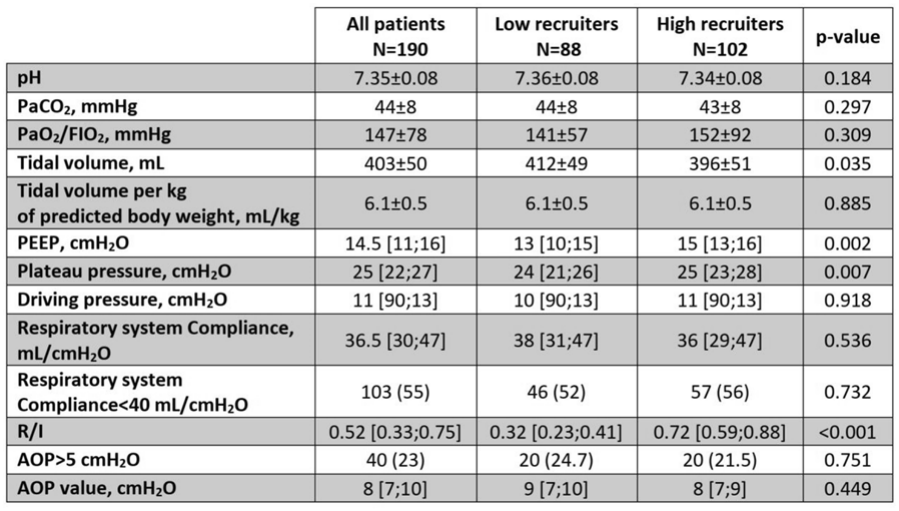


Characteristics of the patients according to recruitability. Data are shown as mean ± SD, median [IQR] or n (%).

### CO-056 Evolution of alveolar recruitability in acute respiratory distress syndrome related to COVID-19

#### Rémi Coudroy, Maeva Rodriguez, Florent Broca, Jean-Pierre Frat, Arnaud W. Thille

##### CHU de Poitiers, Poitiers, France

**Correspondence:** Rémi Coudroy - r.coudroy@yahoo.fr

*Annals of Intensive Care* 2021, **11(Suppl 1):**CO-056

**Rationale:** Acute respiratory distress syndrome (ARDS) related to COVID-19 pneumonia is associated with prolonged duration of mechanical ventilation. The ventilator management in this setting is debated. We report herein the evolution of respiratory system compliance and alveolar recruitability throughout ICU stay in patients admitted with ARDS related to COVID-19.

**Patients and methods/materials and methods:** From March 16th to April 10th 2020, all patients admitted to our ICU for ARDS related to COVID-19 infection according to the Berlin definition were included. Respiratory mechanics were collected daily during passive ventilation in supine position. Potential for lung recruitment during a single-breath maneuver and low-flow inflation pressure–time curve to detect airway closure were performed on a daily basis as previously described (1). Alveolar recruitability was defined by a recruitment-to-inflation ratio (R/I ratio) ≥ 0.5. The evolution of respiratory system compliance and R/I ratio over time was arbitrarily categorized in three periods of time according to the day of intubation: within the first 3 days, between day 4 and day 7, and after day 7

**Results:** Among the 32 patients included during the study period, PaO_2_/FiO_2_ after intubation was 133 ± 89 mmHg, positive end-expiratory pressure (PEEP) was 12 ± 3 cmH_2_O, median duration of mechanical ventilation was 18 days [11–25] and in-ICU mortality was 19%. Respiratory mechanics were assessed 222 times during their ICU stay. All in all, respiratory system compliance was 30 ± 8 mL/cm H_2_O, and decreased over time from 35 ± 6 mL/cmH_2_O in the first 3 days following intubation to 27 ± 8 mL/cmH_2_O after day 7. Potential for lung recruitment was assessed 67 times in 25 out of 32 patients (78%). All in all, R/I ratio was 0.55 (0.39–0.77) and remained stable over time.

**Conclusion:** According to our cohort of ARDS related to COVID-19, respiratory system compliance was low and decreased over time. However, most of patients had high potential for lung recruitment, which persisted after prolonged mechanical ventilation, despite the loss of compliance. Whether the adjustment of PEEP according to alveolar recruitability would improve outcomes remains to be tested.

**Reference**

1. Chen L et al. Potential for Lung Recruitment Estimated by the Recruitment-to-Inflation Ratio in Acute Respiratory Distress Syndrome. A Clinical Trial. American Journal of Respiratory and Critical Care Medicine. 2020;201(2):178–87.

**Compliance with ethics regulations:** Yes in clinical research.

### CO-057 Longitudinal changes in compliance, oxygenation and ventilatory ratio in COVID-19 vs. non-COVID-19 associated acute respiratory distress syndrome

#### François Beloncle^1^, Antoine Studer^2^, Valérie Seegers^3^, Jean-Christophe Richard^1^, Christophe Desprez^1^, Nicolas Fage^1^, Hamid Merdji^2^, Bertrand Pavlovsky^1^, Julie Helms^2^, Sybille Cunat^2^, Satar Mortaza^1^, Julien Demiselle^1,2^, Laurent Brochard^4^, Alain Mercat^1^, Ferhat Meziani^2^

##### ^1^Medical ICU, University Hospital of Angers, Vent’Lab, University of Angers, Angers, France; ^2^Medical ICU, University Hospital of Strasbourg, University of Strasbourg, INSERM, UMR 1260, RNM, FMTS, Strasbourg, France; ^3^Oncology Data Factory and Analytics, ICO Integrated Center for Oncology, Angers, France; ^4^Keenan Research Centre, Li Ka Shing Knowledge Institute, St. Michael’s Hospital, Interdepartmental Division of Critical Care Medicine, University of Toronto, Toronto, Canada

**Correspondence:** François Beloncle - francois.beloncle@univ-angers.fr

*Annals of Intensive Care* 2021, **11(Suppl 1):**CO-057

**Rationale:** Little is known about the specific pathophysiology of COVID-19 ARDS. It has been advocated that COVID-19 ARDS may be characterized by severe hypoxemia, relatively normal respiratory system compliance (C_RS_) and a high pulmonary dead space fraction compared to the “classical form” of ARDS. This study aimed at comparing initial values and longitudinal changes in C_RS_, oxygenation parameters and ventilatory ratio (VR) in COVID-19 and non-COVID-19-associated ARDS patients matched on oxygenation.

**Patients and methods/materials and methods:** One hundred and thirty-five patients with COVID-19 ARDS from two centers were prospectively included in a physiological study; 767 non-COVID-19 ARDS from a previously published large randomized controlled trial (*Express* study) were used for the purpose of at least 1:2 matching. A propensity-matching based on age, severity score, oxygenation and positive end-expiratory pressure was performed using 112 COVID-19 and 273 non-COVID-19 ARDS.

**Results:** Both groups were similar for initial oxygenation. 61.6% and 64.1% of COVID-19 and non-COVID-19 ARDS patients were still ventilated at day 7, respectively. COVID-19 patients had a higher body mass index. C_RS_ was higher in COVID-19 than in non-COVID-19 patients at days 1 (median [IQR], 35 [27–44] vs 31 [25–39] ml cmH_2_O^−1^, *p* = 0.014) and 3 but not at day 7. C_RS_ was correlated with PaO_2_/FiO_2_ only in non-COVID-19 patients. PaO_2_/FiO_2_ became lower in COVID-19 patients at days 3 and 7. VR was overall lower at day 1 in COVID-19 patients but became slightly higher at day 7. Among the 112 matched patients with COVID-19 ARDS, VR was higher in those ventilated using heat and moisture exchangers than in those using heated humidifiers. VR was higher in the 23 (21%) COVID-19 patients with a diagnosis of thromboembolic event than in those without. After adjustment on PaO_2_/FiO_2_, positive end-expiratory pressure and humidification device, C_RS_ and VR were found not different between COVID-19 and non-COVID-19 patients at day 7. Day-28 mortality did not differ in COVID-19 and non-COVID-19 ARDS (25.9% and 29.3%, respectively, *p* = 0.500).

**Conclusion:** COVID-19 ARDS initially differs from classical ARDS by a higher C_RS_ dissociated from oxygenation and a lower VR. Oxygenation becomes lower after the first days and VR increased from day 1 to day 7 in COVID-19 patients. Multivariate analyses revealed that differences in C_RS_ and VR were no longer existing at day 7, suggesting that initial specific features of COVID-19 ARDS disappeared during the first week of evolution due to the natural course of ARDS and the differences in ventilatory management.

**Compliance with ethics regulations:** Yes in clinical research.

### CO-058 TACOS study: therapeutic anticoagulation for the treatment of adult severe COVID-19 patients

#### Romy Younan^1^, Nicolas Peron^1^, Bertrand Hermann^1^, Emmanuel Guerot^1^, Ana Novara^1^, Jean-Loup Augy^1^, Amer Hamdan^1^, Clotilde Bailleul^2^, Francesca Santi^1^, Marine Rolland^1^, Nadia Aissaoui^1^, Caroline Hauw-Berlemont^1^, Jean-Luc Diehl^1^

##### ^1^Hopital Européen Georges Pompidou, Paris, France; ^2^Hopital Nord, Marseille, France

**Correspondence:** Romy Younan - romy_younan@hotmail.com

*Annals of Intensive Care* 2021, **11(Suppl 1):**CO-058

**Rationale:** The outbreak of SARS-CoV2 infection has affected nearly every country worldwide. Although the clinical spectrum of COVID-19 is variable, reports note increased thromboembolic events among hospitalized patients. However, the role of therapeutic anticoagulation (AC) remains unclear. We aimed at comparing prospectively the occurence of thrombotic/ischemic events in COVID-19 patients with acute respiratory distress syndrome (ARDS), in the medical intensive care unit (ICU) of a university hospital center, treated with either standard prophylactic low molecular weight heparin or curative unfractionned heparin (UFH), using a before/after design. We also evaluated the occurence of major bleeding events and the ICU mortality in each group.

**Patients and methods/materials and methods:** We analyzed 116 patients admitted for COVID-19 ARDS from March 17th 2020 to December 6th 2020; 56 patients in the prophylactic group (enoxaparin 4000 UI/day SC) and 60 in the therapeutic group (UFH continuous intravenous infusion with anti-factor Xa activity target between 0.3 and 0.6 UI/ml). The median age was 66 [58.8–73.3], 79% were male patients and the median SAPS II at the first day of admission was 48 [40–59]. The median body mass index (BMI) was 27.2 [24.0–30.6].

**Results:** The preliminary results show no significant difference between the two groups in terms of thromboembolic and major bleeding events (Table). Nineteen patients (16%) presented at least one deep vein thrombosis during the ICU stay, 10 patients (8%) presented at least one pulmonary embolism, 2 patients (2%) presented a confirmed ischemic stroke, and 4 patients (3%) a limb ischemia. A total of 10 patients (9%) presented at least one major bleeding event. Treatment was shifted from preventive to curative in 29 patients (51.8%); 18 of them for thrombotic and ischemic events, 6 because of a respiratory aggravation or significant increase in D-dimers, 3 for arrhythmias, and 2 for continuous renal replacement therapy. Sixty-six patients (57%) needed renal replacement therapy and ICU mortality rate was 50%, without significant difference between groups.

**Conclusion:** Therapeutic anticoagulation using UFH with careful anti-factor Xa activity monitoring was not associated with more severe bleeding. However, we did not observe less thrombotic events, and mortality was comparable with standard prophylactic regimen.

**Compliance with ethics regulations:** Yes in clinical research.
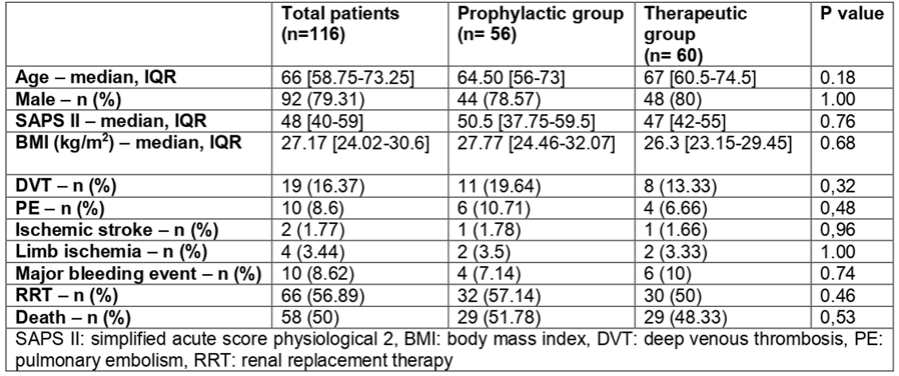


Demographic characteristics, thrombotic and ischemic complications, initiation of RRT, major bleeding events and death rate

### CO-059 Which CT scan score for Covid-19 pulmonary pneumonia: comparison between semi-quantitative and quantitative scores

#### Syrine Maatouk, Sourour Belhadj Youssef, Safa Fathallah, Salma Jerbi, Zeineb Hammouda, Manel Lahmar, Saouessen Ben Abdallah, Fehmi Dachraoui, Fekri Abroug, Lamia Ouanes Besbes

##### CHU F.Bourguiba, Monastir, Tunisie

**Correspondence:** Fekri Abroug - fekri.abroug@gmail.com

*Annals of Intensive Care* 2021, **11(Suppl 1):**CO-059

**Rationale:** CT scan findings are important for the diagnosis and prognosis of Covid-19. According to the literature, there are several scores based either on a semi-quantitative evaluation of the extent of lesions or on a quantitative evaluation taking into account the type and extension of associated lesions. We compared one of each category regarding their performance to estimate the disease severity.

**Patients and methods/materials and methods:** This is a prospective study performed during a 5-month period (September 2020–January 2021) in a 16-bed ICU of a University Hospital. All patients with ARDS secondary to COVID-19 confirmed with RT-PCR and a chest CT scan were included. Two CT scores were calculated: score 1 (based on the semi-quantitative estimation of the extent of pulmonary lesions) and score 2 based on the calculation of the disease extent between 6 lung areas weighed by the type of lesions (ground glass opacity, consolidation, air bronchogram and nodular opacities) (Ref 1). We assessed the discriminative power of the evaluated scores by measuring the area under the ROCurves (AUC) for the association with the need of tracheal intubation, and ICU mortality.

**Results:** During the study period 101 patients fulfilled the inclusion criteria and were included in the study. The median age was 64 (IQR: 55–69.5) years and 72.3% were male. The majority (83.2%) was assisted with high-flow nasal oxygen and 10 (9.9%) patients were intubated upon admission. In spontaneously breathing patients, failure of HFNO was observed in 24 (23.8%) patients and required secondary use of invasive mechanical ventilation. The AUC for score 1 and score 2 achieved were 0.66 (95% CI 0.556–0.782) and 0.74 (95% CI 0.636–0.849), respectively, indicating a good discrimination for the risk of intubation (Fig. 1). Regarding the performance of these scores in predicting ICU mortality, the AUC were 0.73 (95% CI 0.632–0.835) for score 1 and 0.79 (95% CI 0.700–0.889), for score2. Both scores were well correlated (Pearson correlation coefficient of 0.71).

**Conclusion:** Semi-quantitative and quantitative scores are well correlated and have acceptable performance in evaluating Covid-19 severity

**Reference**

1. Yuan M et al. PLoS ONE 2020.

**Compliance with ethics regulations:** Yes in clinical research.
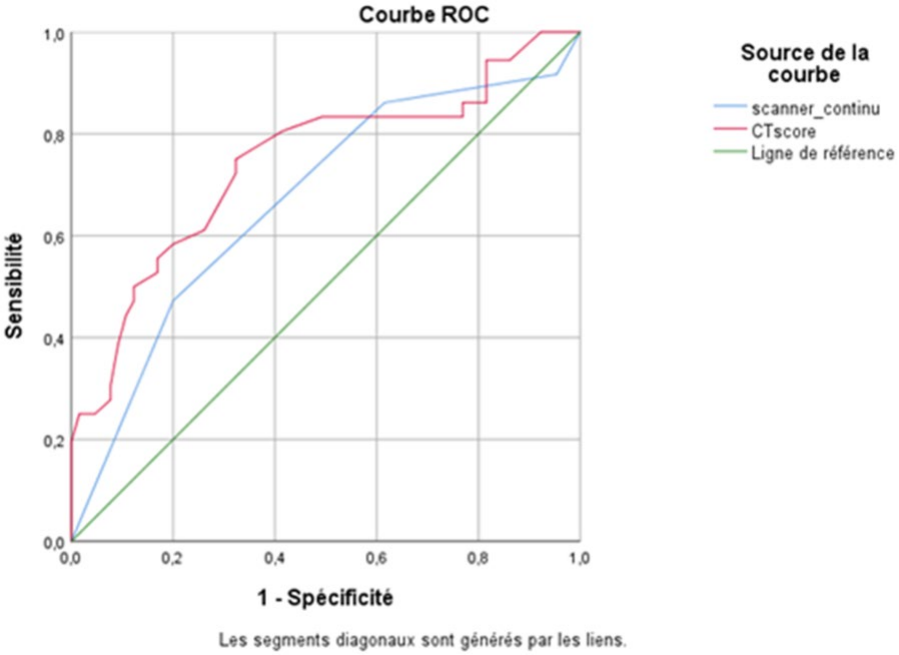


ROC analysis of the two scores for prediction of requiring invasive mechanical ventilation

### CO-060 Neutrophil extracellular traps in SARS-CoV2-related pneumonia in ICU patients: the NETCOV2 study

#### Mathieu Godement^1^, Jaja Zhu^1^, Charles Cerf^2^, Antoine Vieillard-Baron^1^, Agathe Maillon^1^, Benjamin Zuber^2^, Valérie Bardet^1^, Guillaume Geri^1^

##### ^1^Hôpital Ambroise Paré, Boulogne-Billancourt, France; ^2^Hôpital Foch, Suresnes, France

**Correspondence:** Mathieu Godement - mathieu.godement@aphp.fr

*Annals of Intensive Care* 2021, **11(Suppl 1):**CO-060

**Rationale:** Severe acute respiratory syndrome coronavirus 2 (SARS-CoV-2) is a poorly understood disease involving a high inflammatory status. Neutrophil extracellular traps (NETs) have been described as a new pathway to contain infectious diseases, but can also participate in the imbalance of the inflammatory and the coagulation systems. NETs could be a therapeutic target in COVID-19 patients.

**Patients and methods/materials and methods:** Consecutive patients with SARS-CoV2-related pneumonia admitted to the intensive care unit were included in a prospective bicentric study. Neutrophil extracellular trap concentrations were quantified in whole blood samples at day-1 and day-3 by flow cytometry. The primary outcome was the association between the blood NET quantification at ICU admission and the number of days with refractory hypoxemia defined by a PaO2/FIO2 ratio ≤ 100 mmHg.

**Results:** Among 181 patients admitted to the ICUs for acute respiratory failure related to SARS-CoV2 pneumonia, 58 were included in the analysis. Patients were 62 [54, 69] years old in median, mostly male (75.9%). The median number of days with severe hypoxemia was 4 [2, 6] days and day-28 mortality was 27.6% (*n* = 16). The blood level of NETs significantly decreased between day-1 and day-3 in patients who survived (59.5 [30.5,116.6] to 47 [33.2,62.4] *p* = 0.006; 8.6 [3.4,18.0] to 4 [1.4,10.7] *p* = 0.001 and 7.4 [4.0,16.7] to 2.6 [1.0,8.3] *p* = 0.001 for MPO+, Cit-H3+ and MPO+ Cit-H3 + NETs, respectively) while it remained stable in patients who died (38.4 [26.0,54.8] to 44.5 [36.4,77.7] *p* = 0.542; 4.9 [1.3,13.0] to 5.5 [2.8,6.9] *p* = 0.839 and 4 [1.3,13.6] to 2.7 [1.4,4.5] *p* = 0.421 for MPO+, Cit-H3+ and MPO + Cit-H3 + NETs, respectively). In multivariable negative binomial regression, the blood level of MPO + NETs was negatively associated with the number of days with severe hypoxemia within 7 days (0.84 [0.73, 0.97]), while neither Cit-H3 + NETs nor double-positive NETs were significantly associated with the primary outcome.

**Conclusion:** The whole blood level of NETs at day-1 was negatively associated with the number of days with severe hypoxemia in patients admitted to the intensive care unit for SARS-CoV2-related pneumonia. The lack of decrease in the blood level of NETs between day-1 and day-3 discriminated patients who died within day-28.

**Compliance with ethics regulations:** Yes in clinical research

### CO-061 Bacterial co-infection among intubated patients with SARS-CoV-2 or influenza pneumonia: a European multicenter comparative cohort study

#### Anahita Rouze^1^, Ignacio Martin-Loeches^2^, Pedro Povoa^3^, Demosthenes Makris^4^, Antonio Artigas^5^, Julien Labreuche^1^, Saad Nseir^1^

##### ^1^CHRU LILLE, Lille, France; ^2^Multidisciplinary Intensive Care Research Organization (MICRO), St. James’ Hospital, Dublin, Irland; ^3^Hospital de São Francisco Xavier, Lisbon, Portugal; ^4^University Hospital of Larissa, Larissa, Greece; ^5^Corporacion Sanitaria Universitaria Parc Tauli, CIBER Enfermedades Respiratorias, Sabadell, Spain

**Correspondence:** Anahita Rouze - anahitarouze@gmail.com

*Annals of Intensive Care* 2021, **11(Suppl 1):**CO-061

**Rationale:** Early empirical antimicrobial treatment is frequently prescribed to critically ill patients with COVID-19, based on Surviving Sepsis Campaign guidelines. We aimed to determine the prevalence of bacterial co-infection in intubated patients with SARS-CoV-2 pneumonia, as compared to influenza pneumonia, and to characterize its microbiology and impact on clinical outcomes.

**Patients and methods/materials and methods:** Multicenter retrospective European cohort performed in 36 ICUs. All adult patients receiving invasive mechanical ventilation > 48 h were eligible if they had SARS-CoV-2 or influenza pneumonia at ICU admission. Bacterial co-infection was defined by bacterial isolation, within 48 h after intubation, in endotracheal aspirates, bronchoalveolar lavage, or blood cultures, or a positive pneumococcal or legionella urinary antigen test.

**Results:** 1050 patients were included (568 in SARS-CoV-2 and 482 in influenza groups). The prevalence of bacterial co-infection was significantly lower in patients with SARS-CoV-2 pneumonia as compared to patients with influenza pneumonia (8.6 vs 32.7%, unadjusted odds ratio (OR) 0.20 (95% confidence interval (CI) 0.13 to 0.28), adjusted OR 0.21 (95% CI 0.14 to 0.30), *p* < 0.0001). Gram-positive cocci were responsible for 55% and 70% of co-infection in patients with SARS-CoV-2 and influenza pneumonia, respectively. Bacterial co-infection was associated with increased adjusted hazard ratio for 28-day mortality in patients with SARS-CoV-2 pneumonia (1.71 (95% CI 1.09 to 2.68), *p* = 0.019).

**Conclusion:** Bacterial co-infection within 48 h after intubation is significantly less frequent in patients with SARS-CoV-2 pneumonia as compared to patients with influenza pneumonia, and is associated with increased risk for 28-day mortality only in patients with SARS-CoV-2 pneumonia. (On behalf of the coVAPid study group)

**Compliance with ethics regulations:** Yes in clinical research.
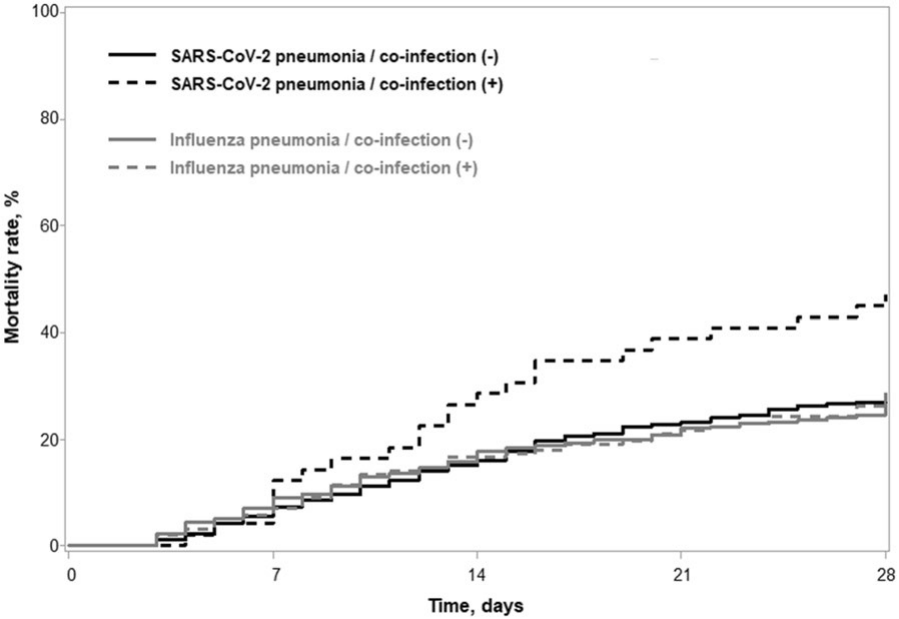


Cumulative incidence of 28-day mortality according to disease groups (SARS-CoV-2 pneumonia vs influenza pneumonia) and bacterial co-infection (presence vs absence)

### CO-062 Biomarkers of lung fibrosis in SARS-CoV2-related pneumonia patients: relationship with initial presentation and long-term pulmonary function

#### Pierre-Alexandre Haruel^1,4^, Ilan Obadia^1^, Dominique Cabral^1^, Anne-Laure Roux^1,4^, Martin Rottman^2,4^, Antoine Pilon^3^, Guillaume Geri^1,4^

##### ^1^Hôpital Ambroise Paré, Boulogne Billancourt, France; ^2^Hôpital Raymond Poincaré, Garches, France; ^3^Hôpital Saint Antoine, Paris, France; ^4^Université Paris-Saclay, Versailles, France

**Correspondence:** Guillaume Geri - guillaume.geri@aphp.fr

*Annals of Intensive Care* 2021, **11(Suppl 1):**CO-062

**Rationale:** SARS-CoV-2-related pneumonia may lead to different severity of acute lung injury and different CT scans patterns of the lung. Evolution to lung fibrosis has been suggested, but data are lacking on the usefulness of lung biomarkers in differentiating patients according to their clinical and radiological presentation.

**Patients and methods/materials and methods:** Consecutive patients admitted to a tertiary medical ICU for SARS-CoV-2-related pneumonia were included in the analysis. Influenza-related pneumonia ICU patients were included as controls. Fibrosis biomarkers were assessed on sera of these patients stored were analyzed using ELISA kits (TNF-alpha, RANTES, PDGF, MIP1-alpha, MIP1-beta, MCP1, IP10, IL13, IL9, IL8, IL6, IL4, IL1ra, IFN-gamma, G-CSF, eotaxin, FGF). Kazerroni’s radiological score has been evaluated by two radiologists, collecting spread of honeycomb and groun glass opacities (ranging from 0 to 5 for each). Descriptive statistics are provided.

**Results:** 81 patients were included in the analysis (70 SARS-CoV-2 and 11 influenza) (median age 64 [57,72], 80.2% male). No difference in terms of demographics was observed between influenza and SARS-CoV-2 patients. SAPS2 score was similar in both groups as well as respiratory SOFA score while total SOFA score was higher in influenza as compared to SARS-CoV-2 patients (10 [6,11] vs. 4 [3,6]). Overall day-28 mortality was 30.4% (20 vs. 32.2% in influenza and SARS-CoV-2 patients, *p* = 0.686). The total radiological score was 4.6 [3.2,5.8], higher in SARS-CoV-2 patients (3 [1, 3.8] vs. 5.2 [3.4, 6], *p* = 0.008). No correlation was observed between the level of fibrosis biomarkers and the total radiological score nor with the honeycomb or ground glass sub-scores. Biomarkers levels were not different in patients with or without restrictive lung-function at 6-months.

**Conclusion:** While the quantification of fibrosis on CT scan of the lung was higher in SARS-CoV-2 patients than in influenza patients admitted to the ICU, we did not observe any difference in the levels of fibrosis biomarkers allowing a better discrimination of patients.

**Reference**

1. Kazerooni EA, Martinez FJ, Flint A, Jamadar DA, Gross BH, Spizarny DL, et al. Thin-section CT obtained at 10-mm increments versus limited three-level thin-section CT for idiopathic pulmonary fibrosis: correlation with pathologic scoring. Am J Roentgenol.

**Compliance with ethics regulations:** Yes in clinical research.

### CO-063 Soluble VCAM1 could be a critical feature of the coagulopathy signature in severe COVID-19 pneumonia

#### Mathieu Blot^1,2^, Emmanuel De Maistre^3^, Abderrahmane Bourredjem^4^, Jean-Pierre Quenot^2,4,5^, Maxime Nguyen^2,6^, Belaid Bouhemad^2,6^, Pierre-Emmanuel Charles^5^, Christine Binquet^2,4^, Lionel Piroth^1,4^

##### ^1^Infectious Diseases Department, Dijon Bourgogne University Hospital, Dijon, France; ^2^Lipness team, INSERM Research Center LNC-UMR1231 and LabEx LipSTIC, University of Bourgogne, Dijon, France; ^3^Laboratory of Hemostasis, Dijon, Bourgogne University Hospital, Dijon, France; ^4^INSERM, CIC1432, Clinical Epidemiology unit, Dijon, France; Dijon Bourgogne University Hospital, Clinical Investigation Center, Clinical Epidemiology/Clinical trials unit, Dijon, France; ^5^Department of Intensive Care, Dijon Bourgogne University Hospital, Dijon, France; ^6^Anesthesiology and Critical Care Department, Dijon Bourgogne University Hospital, Dijon, France

**Correspondence:** Mathieu Blot - mathieu.blot@chu-dijon.fr

*Annals of Intensive Care* 2021, **11(Suppl 1):**CO-063

**Rationale:** COVID-19 displays distinct characteristics that suggest a unique pathogenesis. The objective of this study was to compare biomarkers of coagulopathy and outcomes in COVID-19 and non-COVID-19 patients with severe pneumonia.

**Patients and methods/materials and methods:** In a prospective study, were included non-immune-compromised patients with severe pneumonia (at least 2 criteria of the quick-SOFA score and/or need for mechanical ventilation or vasopressors). COVID-19 patients were all tested positive for SARS-CoV-2 by reverse transcriptase-polymerase chain reaction, and non-COVID-19 were enrolled before the pandemic started. Clinical and biological characteristics (including plasma biomarkers of coagulopathy measured with a Luminex assay) were compared.

**Results:** Thirty-six non-COVID-19 and 27 COVID-19 were included, most of them requiring ICU (respectively, 89% and 100%). At similar baseline severity (PaO2/FiO2 and SOFA score), COVID-19 patients required mechanical ventilation (MV) for significantly longer than non-COVID-19 patients (15 days, IQR = 7–22 vs. 4 days, IQR = 0–14.5; *p* = 0.0049) and more frequently developed venous thrombotic complications (7 (26%) vs. 2 (6%); *p* = 0.0312). Compared to non-COVID-19 patients, COVID-19 patients displayed a unique plasma signature of coagulopathy-associated biomarkers with significantly higher plasma concentrations of soluble vascular cell adhesion molecule 1 (sVCAM1) (5739 ± 3293 vs. 3700 ± 2124 ng/ml; *p* = 0.009), but lower levels of D-dimers, von Willebrand factor A2 (vWF-A2), soluble intercellular adhesion molecule 1 (sICAM1), soluble triggering receptor expressed on myeloid cells 1 (sTREM1), vascular endothelial growth factor (VEGF), and P-selectin (all with *p* < 0.05). In addition, we observed a non-significant difference for plasma levels of soluble thrombomodulin (sTM), tissue factor pathway inhibitor (TFPI), u-plasminogen activator (uPA), C-X-C motif chemokine ligand (CXCL)4, platelet derived growth factor (PDGF)-AA, PDGF-AB/BB. Principal component analysis identified two main patterns, with a clear distinction between non-COVID-19 and COVID-19 patients. In multivariable linear regression, sVCAM1 was independently associated with a longer duration of MV (*p* = 0.027), after adjustment for age, COVID-19 status, SOFA score and prehospital anticoagulation therapy. In addition, multivariable analysis showed that COVID-19 status was independently associated with sVCAM1. Finally, we identified close correlations between sVCAM1 and some features of COVID-19 immune dysregulation (i.e. CXCL10, GM-CSF, and IL-10).

**Conclusion:** Compared to non-COVID-19 patients, COVID-19 patients displayed a peculiar coagulopathy signature with significantly higher concentrations of sVCAM1, but lower concentrations of some classical biomarkers of coagulation or platelet activation. This could result into a higher level of immune-mediated endotheliopathy. sVCAM1 was independently associated with the duration of MV and could represent a major protagonist in driving coagulopathy and lung injury. VCAM-1, or the immune response driving its expression, could be considered as promising therapeutic pathway.

**Compliance with ethics regulations:** Yes in clinical research

### CO-064 Incidence and risk-factors of fungal infection in Covid-19 ICU patients

#### Sourour Belhajyoussef, Safa Fathallah, Syrine Maatouk, Zeineb Hammouda, Manel Lahmar, Saoussen Benabdallah, Fahmi Dachraoui, Fekri Abroug, Lamia Ouanes-Besbes

##### CHU F.Bourguiba, Monastir, Tunisie

**Correspondence:** Fekri Abroug - fekri.abroug@gmail.com

*Annals of Intensive Care* 2021, **11(Suppl 1):**CO-064

**Rationale:** Recent studies pointed out the frequency of the co- or upper-infection of Covid-19 patients under MV with fungi. This might be due to immunological disturbances caused by the virus, use of corticosteroids, or pre-existing comorbidities such as diabetes or obesity. We aimed at determining the frequency and risk-factors of fungal infections in ICU patients admitted with severe Covid-19 and requiring mechanical ventilation.

**Patients and methods/materials and methods:** This is a prospective study conducted between September 2020 to January 2021 in a 16-bed ICU of a University Hospital. All patients with ARDS secondary to COVID-19 confirmed with RT-PCR, an age older than 18 years, with an ICU stay longer than 24 h were included. They were divided into two groups according to the occurence of fungal subinfection (F (+) and F (−)). We recorded their baseline characteristics, clinical, radiologic and biological variables on ICU admission, and their treatment during the ICU stay. We recorded ICU occurence of fungal infection at admission or during the ICU stay, and the ICU outcome.

**Results:** During the study period, 137 patients with Covid-ARDS were admitted in the ICU. The median age was 64 years, 72.3% were males. The most frequent comorbidity was hypertension (48.9%), followed by diabetes (41.6%). For the ventilatory management, patients were assisted with both non-invasive ventilation (HFNC (79.6%) and NIV (4.4%)) and mechanical invasive ventilation (11.7%). Fungal subinfection and invasive candidiasis were found in 27.7% and 26.2% patients, respectively. Sites identified were mainly respiratory (65.8%) and urinary (57.9%). The most frequent species were C. albicans (68%) followed by C. tropicalis (42.1%). Most of patients received caspofungin (92.1%). The mortality rate was 35.8%. Comparison between infected and non-infected groups showed significant difference in their baseline characteristics in particular for diabetes (*p* = 0.045). The use of arterial catheter (*p* < 0.001), central venous catheter (*p* < 0.001), dialysis (*p* < 0.001), parenteral nutrition (*p* < 0.001), broad-spectrum antibiotics (*p* < 0.001), invasive mechanical ventilation (*p* < 0.001) were statistically different. The occurrence of septic shock (*p* < 0.001), ventilator-acquired pneumonia (*p* < 0.001), acute kidney injury (*p* < 0.001) were all significantly more frequent in patients who developed fungal infection.

**Conclusion:** Our study showed that critically ill patients with Covid-ARDS have poor prognosis and identified multiple factors related to the occurrence of fungal infection already present at ICU admission or acquired during ICU stay

**Compliance with ethics regulations:** Yes in clinical research.

### CO-065 Influenza and COVID-19-associated pulmonary aspergillosis: are the pictures different?

#### Florian Reizine, Kieran Pinceaux, Mathieu Lederlin, Brice Autier, Helene Guegan, Arnaud Gacouin, Yoann Launey, Mathieu Lesouhaitier, Benoit Painvin, Christophe Camus, Alexandre Mansour, Florence Robert-Gangneux, Yves Le Tulzo, Jean Marc Tadie, Adel Maamar, Jean-Pierre Gangneux

##### CHU rennes, Rennes, France

**Correspondence:** Florian Reizine - florian.reizine@chu-rennes.fr

*Annals of Intensive Care* 2021, **11(Suppl 1):**CO-065

**Rationale:** Invasive pulmonary aspergillosis (IPA) in intensive care unit patients is a major concern, in particular for those with acute respiratory distress syndrome (ARDS). As observed previously for influenza-associated ARDS, the SARS-CoV-2 pandemic has shown a high proportion of COVID-19 patients with ARDS to be at risk of developing invasive fungal diseases. The shared clinical, biological, and radiological characteristics of both COVID-19- and influenza-associated invasive pulmonary aspergillosis (IPA) are still little explored. We aimed at exploring similarities and differences between COVID-19-associated pulmonary aspergillosis (CAPA) and influenza-associated pulmonary aspergillosis (IAPA)

**Patients and methods/materials and methods:** In a monocentric cohort study, we included 120 patients, 71 with influenza and 49 with COVID-19 associated ARDS. IPA diagnosis was made in accordance with the newly published definitions of CAPA and IAPA (1–2).

**Results:** Among the 120 ARDS patients, we observed equivalent prevalence of IPA in influenza and COVID-19 populations: 17 IAPA (23.9%) and 10 CAPA (20.4%). There were no significant differences in demographic or admission characteristics between patients with and without IPA. Kaplan–Meier curves showed significantly higher 90-day mortality for overall IPA patients (*p* = 0.032), whereas mortality did not differ between CAPA and IAPA patients. The duration of mechanical ventilation was higher for patients with IPA (23 days [IQR 17–40] than those without (17 days [IQR 9–25], *p* = 0.038). Patients with COVID-19 and influenza associated ARDS treated with corticosteroids were more likely to develop IPA. Radiological findings of IPA in both populations using the new criteria increased sensitivity, but with still poor specificity. Nonetheless, we observed differences between IAPA and CAPA with a higher proportion of features suggestive of IPA in IAPA patients.

**Conclusion:** ICU patients presenting with ARDS during COVID-19 are very similar to those with severe influenza pneumonia in terms of the prevalence of IPA and outcome. The dramatic consequences on the patients’ prognosis emphasize the need for a better awareness in these particular populations. Future larger prospective studies may help in designing the most well-adapted personalized management to prevent IPA, which represents a high burden of death in severe COVID-19 and Influenza pneumonia.

**References**

1. Verweij PE, Rijnders BJA, Brüggemann RJM, Azoulay E, Bassetti M, Blot S, et al. Review of influenza-associated pulmonary aspergillosis in ICU patients and proposal for a case definition: an expert opinion. Intensive Care Med. 2020;46(8):1524–35.
2. Koehler P, Bassetti M, Chakrabarti A, Chen SCA, Colombo AL, Hoenigl M, Klimko N, Lass-Flörl C, Oladele RO, Vinh DC, Zhu LP, Böll B, Brüggemann R, Gangneux JP, Perfect JR, Patterson TF, Persigehl T, Meis JF, Ostrosky-Zeichner L, White PL, Verweij PE, Corne Lancet Infect Dis. 202014:S1473-3099(20)30847–1.

**Compliance with ethics regulations:** Yes in clinical research.
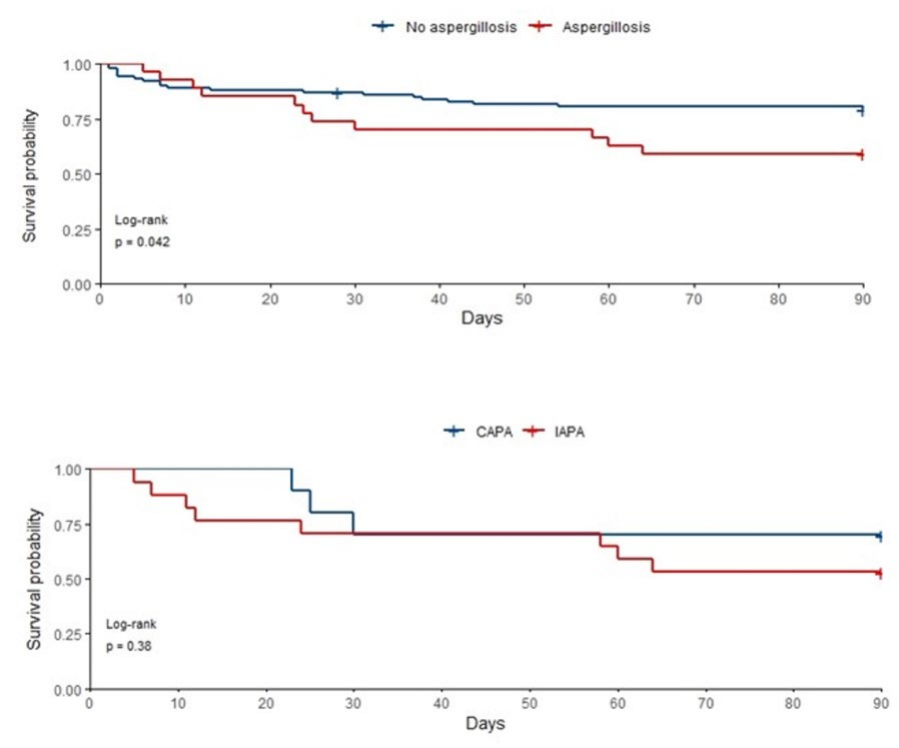


Cumulative 90-day mortality from admission to the intensive care unit in the whole population and among CAPA and IAPA patients

### CO-066 Does SARS-CoV-2 infection modify the relationship between ventilator-associated pneumonia and mortality? A planned ancillary analysis of the coVAPid cohort

#### Anahita Rouzé^1^, Ignacio Martin-Loeches^2^, Pedro Povoa^3^, Demosthenes Makris^4^, Antonio Artigas^5^, Saad Nseir^1^

##### ^1^CHU de Lille, Lille, France; ^2^Department of Intensive Care Medicine, Multidisciplinary Intensive Care Research Organization (MICRO), St. James’ Hospital, St. James Street, Dublin, Irland; ^3^Polyvalent Intensive Care Unit, São Francisco Xavier Hospital, Centro Hospitalar de Lisboa Ocidental, Lisbon, Portugal; ^4^Intensive Care Unit, University Hospital of Larissa, Larissa, Greece; ^5^Critical Care Center, Corporacion Sanitaria Universitaria Parc Tauli, Sabadell, Spain

**Correspondence:** Saad Nseir - s-nseir@chru-lille.fr

*Annals of Intensive Care* 2021, **11(Suppl 1):**CO-066

**Rationale:** Patients with SARS-CoV-2 infection are at higher risk for ventilator-associated pneumonia (VAP). No study has evaluated the relationship between VAP and mortality in this population, neither compared this relationship between SARS-CoV-2 patients and other populations. The aim of this study is to determine whether SARS-CoV-2 pneumonia, as compared to influenza pneumonia or no viral infection, modify the relationship between VAP and mortality.

**Patients and methods/materials and methods:** Planned ancillary analysis of a multicenter retrospective European cohort. VAP was diagnosed using clinical, radiological and quantitative microbiological criteria. Univariable and multivariable marginal Cox’s regression models, with cause-specific hazard for duration of mechanical ventilation and ICU stay, were used to compare outcomes between study groups. Extubation, and ICU discharge alive were considered as events of interest, and mortality as competing event.

**Results:** Of 1576 included patients, 568 were SARS-CoV-2 pneumonia, 482 influenza pneumonia, and 526 no evidence of viral infection at ICU admission. VAP was associated with significantly higher risk for 28-day mortality in SARS-CoV-2 (adjusted HR 1.70 (95% CI 1.16–2.47), *p* = 0.006), and influenza groups (1.75 (1.03–3.02), *p* = 0.045), but not in the no viral infection group (1.07 (0.64–1.78), *p* = 0.79). VAP was associated with significantly longer duration of mechanical ventilation in the SARS-CoV-2 group, but not in the influenza or no viral infection groups. VAP was associated with significantly longer duration of ICU stay in the 3 study groups. No significant difference was found in heterogeneity of outcomes related to VAP between the 3 groups.

**Conclusion:** VAP was associated with significantly increased 28-day mortality rate in SARS-CoV-2 patients. However, SARS-CoV-2 pneumonia, as compared to influenza pneumonia or no viral infection, did not significantly modify the relationship between VAP and 28-day mortality. On behalf of the coVAPid study group.

**Reference**

1. Relationship between SARS-CoV-2 infection and the incidence of ventilator-associated lower respiratory tract infections: a European multicenter cohort study. Rouzé A et al. Intensive Care Med 2021;47(2):188–98.

**Compliance with ethics regulations:** Yes in clinical research.
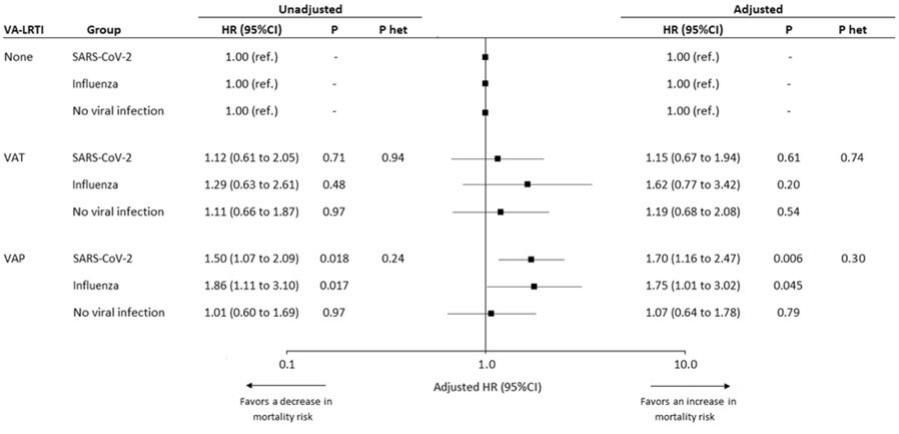


Association between ventilator-associated lower respiratory tract infections and 28-day mortality

### CO-067 Incidence, characteristics and outcome of patients eligible for VV-ECMO: ancillary study performed in a cohort of 752 moderate-to-severe ARDS

#### Matthieu Petit^1^, Armand Mekontso Dessap^2^, Paul Masi^2^, Bruno Evrard^3^, Annick Legras^4^, Guillaume Geri^1^, Philippe Vignon^1,3^, Antoine Vieillard-Baron^1^

##### ^1^Service de Médecine intensive et Réanimation, CHU Ambroise Paré, APHP, Boulogne-Billancourt, France; ^2^Service de Médecine intensive et Réanimation, CHU Henri Mondor, APHP, Créteil, France; ^3^Service de Réanimation polyvalente, CHU de Limoges, Limoges, France; ^4^Service de Médecine intensive et Réanimaiton, CHU de Tours, Tours, France

**Correspondence:** Matthieu Petit - matthieu.petit@aphp.fr

*Annals of Intensive Care* 2021, **11(Suppl 1):**CO-067

**Rationale:** Mortality in most severe form of ARDS is around 60%. EOLIA suggested that ECMO could be effective in some of these patients. Our objective was to assess the incidence of patients who reached EOLIA criteria, as well as their characteristics and outcome in a large cohort of moderate-to-severe ARDS patients.

**Patients and methods/materials and methods:** Ancillary study from a cohort including 11 French intensive care units (ICU) between 1994 and 2012 (ref). Patients with moderate or severe ARDS were included and routinely assessed using trans-esophageal echocardiography within the first 3 days following the diagnosis. All were ventilated with a lung-protective strategy. Patients with a PaO2/FiO2 < 80 mmHg with a PEEP of at least 6 cmH2O, or a pH < 7.25 and PaCO2 > 60 mmHg with a respiratory rate of at list 35 cycles/min, despite prone positionning or NO inhalation, were identified (group 1) and compared to the other patients (group 2).

**Results:** Among the 752 patients (age 58 ± 16 years, SAPS II 53 ± 21), 67 (9%) fullfilled the EOLIA criteria (group 1). They had lower PaO2/FiO2 (62 [55, 72] versus 114 [90, 152], *p* < 0.001), higher PEEP (10 [7, 12] versus 8 [5, 10] cmH2O, *p* < 0.001), and higher driving pressure (17 [14, 20] versus 15 [13, 19] cmH2O, *p* = 0.039). Prone position and NO were more frequently used (82% and 57%, respectively). Incidence of acute core pulmonale and severe right ventricule (RV) dilatation (RV size > LV size) was higher in group 1 (42% versus 20%, *p* < 0.001 and 24% versus 10%, *p* < 0.001, respectively). In-ICU mortality was higher in group 1, while not statistically different from group 2 (46% versus 35%, *p* = 0.11). Among the 31 deceased patients in group 1, 3 died from hypoxic cardiac arrest, 20 from a multi-organ failure, and 5 from ECMO complication. In multivariate analysis, factors associated with mortality in group 1 were severe RV dilatation and driving pressure.

**Conclusion:** In a non-selected and large cohort of moderate-to-severe ARDS patients, only 9% of patients could be considered for VV ECMO according to the Eolia criteria. They demonstrated higher driving pressure and more RV failure. In-ICU mortality was not significantly different from the rest of the cohort. Further studies are needed to determine among these patients those who will really benefit from VV ECMO.

**Reference**

1. Mekontso Dessap A, Boissier F, Charron C, Bégot E, Repessé X, Legras A, Brun-Buisson C, Vignon P, Vieillard-Baron A. Acute cor pulmonale during protective ventilation for acute respiratory distress syndrome: prevalence, predictors, and clinical impact. Intensive Care Med. 2016 May;42(5):862–70.

**Compliance with ethics regulations:** Yes in clinical research.

### CO-068 Prone positioning in severe acute respiratory distress syndrome requiring extracorporeal membrane oxygenation

#### Matthieu Petit^2,4^, Catalin Fetita^7^, Augustin Gaudemer^3,5^, Guillaume Lebreton^5,8^, Guillaume Franchineau^2,6^, Guillaume Hekimian^2,5^, Juliette Chommeloux^2,6^, Marc Pineton De Chambrun^2,5^, Cyrielle Desnos^2,5^, Nicolas Bréchot^2,5^, Charles-Edouard Luyt^2,5^, Alain Combes^2,6^, Matthieu Schmidt^2,5^

##### ^1^APHP/Hopital Ambroise Paré - Service de Médecine intensive et Réanimation, Boulogne-Billancourt, France; ^2^APHP/Groupe Hospitalier Pitié Salpêtrière - Charles Foix - Service de Médecine intensive et Réanimation., Paris, France; ^3^APHP/CHU Bichât - Claude Bernard- Service de radiologie, Paris, France; ^4^Université Paris-Saclay, Paris, France; ^5^Université de Paris, Paris, France; ^6^Université Paris-Sorbonne, Paris, France; ^7^Ecole Télécom-Sud Paris, Courcouronnes, France; ^8^APHP- Groupe Hospitalier Pitié Salpêtrière Charles Foix - Service de chirurgie cardiaque, Paris France

**Correspondence:** Matthieu Petit - matthieu.petit@aphp.fr

*Annals of Intensive Care* 2021, **11(Suppl 1):**CO-068

**Rationale:** The combination of veno-venous (VV)-extracorporeal membrane oxygenation (ECMO) and prone prositionning (PP) has not been fully evaluated and it is still unclear which patients are more likely to benefit from the combination of these two adjunct therapies. Our objective was to report the characteristics and outcomes of the patients proned during (ECMO) for severe acute respiratory distress syndrome (ARDS) and to identify a lung-computed tomography pattern associated with an improvement of the static compliance of the respiratory system after proning on ECMO.

**Patients and methods/materials and methods:** This is a retrospective, single-center study over 8 years in a 26-bed intensive care unit tertiary center. A propensity-score (PS)-matched analysis was performed to compare patients who had prone positioning (PP) during ECMO (“PP ECMO”) and those who did not (“No PP ECMO”). An increase in the static compliance by ≥ 3 mL/cmH2O after 16 h of PP defined a PP-responder. The primary outcome was the time to successful ECMO weaning within the 90 days following inclusion, with death as a competing risk. Assessment of lung aeration before PP with quantitative computed tomography was performed.

**Results:** Out of 298 adult patients with severe ARDS on VV-ECMO, 64 were proned during ECMO. While ECMO duration was similar between the two PS-matched groups, the 90-day probability of being weaned from ECMO and alive was higher in the patients from the PP-ECMO group (0.75 vs 0.54, *p* = 0.03; subdistribution hazard ratio [95% confidence interval] 1.54 [1.05–2.58]), with lower 90-day mortality (20% vs 42%, *p* < 0.01) (Fig. 1). As compared to those without PP during ECMO. ECMO-related complications were similar between the two groups. Patients who did not improve their static compliance had a higher proportion of non-aerated or poorly aerated lung in the ventral and medial-ventral regions (*p* = 0.047).

**Conclusion:** PP during VV-ECMO was a safe and effective procedure associated with a greater probability to be alive and weaned from ECMO at 90 days. Patients with massive dorsal lung lesions and a low proportion of non- or poorly aerated lung tissue in the ventral and medial-ventral regions would likely benefit from this management.

**Compliance with ethics regulations:** Yes in clinical research.
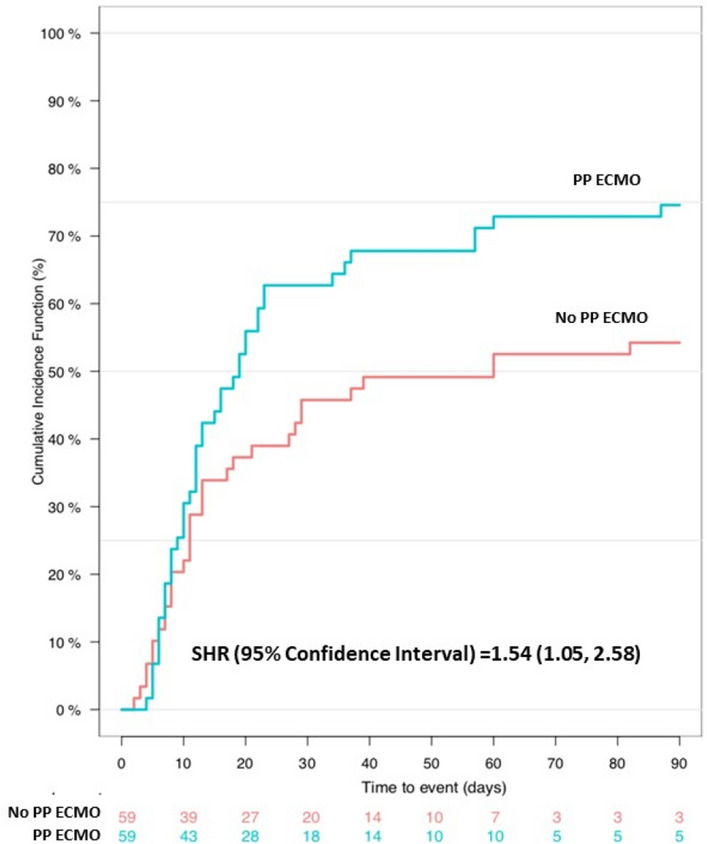


Fig. 1 Cumulative incidence probability of being weaned from ECMO and alive at day-90 in the propensity-matched score population. PP, prone positioning; SHR, subdistribution hazard ratio

### CO-069 Mechanisms associated with changes in respiratory mechanics after extracorporeal membrane oxygenation cannulation for acute respiratory distress syndrome

#### Sylvain Le Pape, Florent Joly, Jean-Pierre Frat, Arnaud W. Thille, Rémi Coudroy

##### CHU de Poitiers, Poitiers, France

**Correspondence:** Rémi Coudroy - r.coudroy@yahoo.fr

*Annals of Intensive Care* 2021, **11(Suppl 1):**CO-069

**Rationale:** A dramatic decrease in respiratory system compliance has been reported after ECMO cannulation in patients who were then transferred to referral center, that could be due to transportation or to changes in ventilator settings [1]. Our objective was to investigate the direct influence of ventilator settings on decreased respiratory system compliance after ECMO.

**Patients and methods/materials and methods:** All patients requiring ECMO for ARDS in our medical ICU between January 2013 and May 2020 were included. Those who had ECMO cannulation in another center and were then transferred to our center were excluded. To assess the influence of ventilator settings on decreased respiratory system compliance, we included only patients ventilated with pressure-mode immediately after ECMO cannulation. Ventilatory parameters were compared 3 h prior to and 3 h following ECMO cannulation

**Results:** Among the 48 patients requiring ECMO during the study period, 22 patients ventilated in pressure-mode after on-site ECMO cannulation were included. Prior to ECMO cannulation, all patients were treated in volume-control mode, received neuromuscular blockers, and all but one experienced prone position. Median [interquartile range] duration of ECMO was 12 days [7–17], 68% of patients (15/22) were successfully weaned from ECMO, and 59% (13/22) were discharged alive from ICU. After ECMO, patients were ventilated with a positive end-expiratory pressure of 15 cmH_2_O [13–15] and an inspiratory pressure of 25 cmH_2_O [25–25]. With respect to ECMO cannulation, mean driving pressure decreased from 16 cmH_2_O [13–24] to 10 [10–12] (*p* < 0.001), respiratory rate from 32 breaths/min [29–34] to 20 [16–20] (*p* < 0.001), and tidal volume from 6.4 mL/kg of predicted body weight [5.8–6.8] to 3.3 [1.9–3.9] (*p* < 0.001). Respiratory system compliance decreased by 27% [0–38] after ECMO (*p* < 0.001). Alveolar collapse after ECMO affected lung area with higher respiratory system compliance of 29 mL/cmH_2_O [17–52] than after ECMO cannulation (17 mL/cmH_2_O [8–23], *p* < 0.001). As a consequence, expiratory tidal volume after ECMO was markedly lower than tidal volume predicted according to respiratory system compliance before ECMO (189 mL [90–236] vs. 261 mL [156–306], *p* < 0.001), i.e. decreased by 27% [17–38] compared to the predicted tidal volume.

**Conclusion:** In our cohort of ECMO patients not transported and ventilated in pressure mode after ECMO, respiratory system compliance decreased by 27% after ECMO. Changes in ventilator settings may lead to deventilation of quite compliant lung. Further research is necessary to better understand the consequences of ultra-protective ventilation during ECMO.

**Reference**

1. Rozé H et al. Decrease of thoracopulmonary compliance with pressure assist controlled ventilation in ARDS patients under ECMO and transported to a referral centre. Intensive Care Med. 2017;43(1):148–9.

**Compliance with ethics regulations:** Yes in clinical research.

### CO-070 Effects of driving pressure-guided ventilation versus tidal volume-guided ventilation on mechanical power in acute respiratory distress syndrome

#### Anne-Fleur Haudebourg, Samuel Tuffet, François Perier, Keyvan Razazi, Nicolas De Prost, Armand Mekontso Dessap, Guillaume Carteaux

##### CHU HENRI MONDOR, Creteil, France

**Correspondence:** Anne-Fleur Haudebourg - annefleur.haudebourg@gmail.com

*Annals of Intensive Care* 2021, **11(Suppl 1):**CO-070

**Rationale:** Over the past decades, to limit the risk of ventilation-induced lung injury (VILI), tidal volume control has been the cornerstone of protective ventilation in acute respiratory distress syndrome (ARDS). However, recent evidence suggests that VILI may instead be related to inappropriate driving pressure. The aim of our study was to compare, in ARDS patients, the effect of driving pressure-guided ventilation versus tidal volume-guided ventilation on the mechanical power, a surrogate for the risk of VILI.

**Patients and methods/materials and methods:** We prospectively included adult patients with moderate-to-severe ARDS. PEEP was set by attending physician. Tidal volume was first adjusted to target 6 ml/kg of predicted body weight (PBW) and subsequently modified within a range from 4 to 10 ml/kg PBW to target a driving pressure between 12 and 14 cmH2O. The respiratory rate was then adjusted within a range from 12 to 40 breaths/min until EtCO2 returned to its baseline value. Respiratory mechanics was assessed during each step in order to compute mechanical power.

**Results:** 24 patients were included between December 2019 and January 2021. The basal driving pressure during tidal volume-guided ventilation was already within the target range of driving pressure-guided ventilation in 5 (21%) patients, above in 3 (12%) and below in 16 (67%). The change from tidal volume- to driving pressure-guided ventilation was accompanied by an overall increase in driving pressure from 9.5 cmH2O [8–12] to 13 cmH2O [12–14], with an increase in tidal volume from 6.0 mL/kg PBW [5.9–6.2] to 8.1 ml/kg PBW [6.4–9.1], while respiratory rate was decreased from 28 breaths/min [25–30] to 17 breaths/min [14–24] (*p* < 0.001 for all comparisons). Driving pressure-guided ventilation was accompanied by a decrease in mechanical power from 29.7 J/min [25.3–38.2] to 25.6 J/min [22.8–31] (*p* < 0.001, Fig. 1), representing a relative decrease of 16% [7–23].

**Conclusion:** A driving pressure-guided ventilation strategy in ARDS may reduce the mechanical power and thus the risk of VILI compared to conventional tidal volume-guided ventilation.

**Compliance with ethics regulations:** Yes in clinical research.
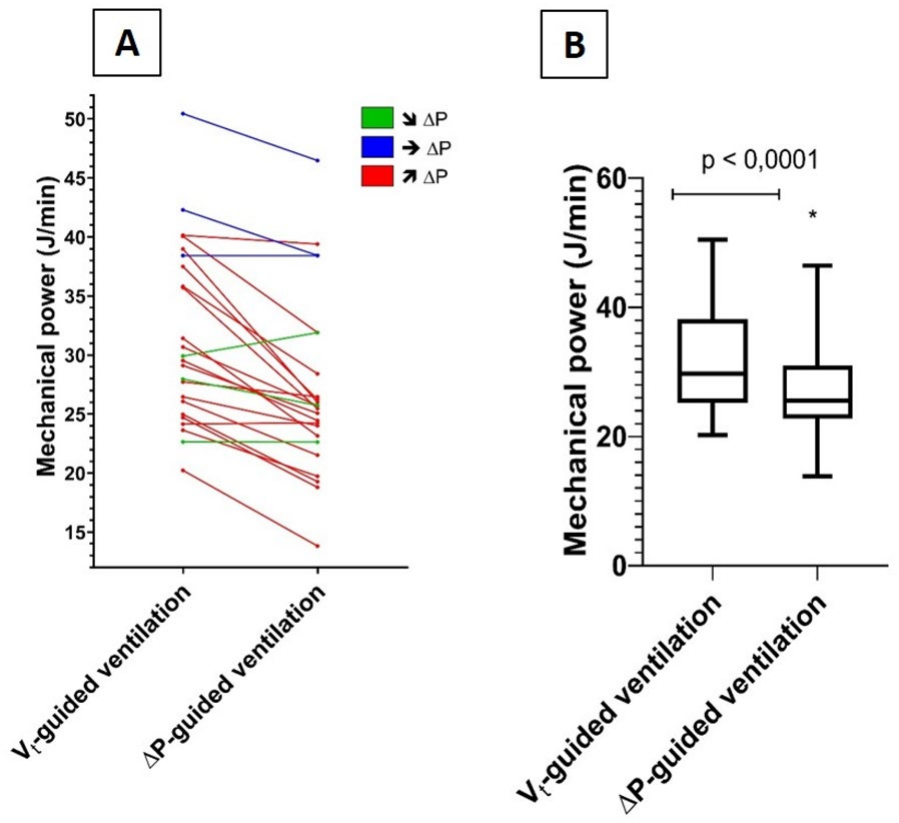


Fig. 1 Comparison of mechanical power between tidal volume-guided ventilation (Vt-guided ventilation) and driving pressure-guided ventilation (deltaP-guided ventilation). **A** Individual data. **B** Box-plots (median and IQR)

### CO-071 PEEP levels computed by titration strategies based on electrical impedance tomography or esophageal pressure measurement in acute respiratory distress syndrome and lung recruitability

#### Bertrand Pavlovsky, Christophe Desprez, Jean-Christophe Richard, Nicolas Fage, Dara Chean, Antonin Courtais, Alain Mercat, François Michel Beloncle

##### Médecine Intensive Réanimation, Vent’Lab, Centre Hospitalier Universitaire d’Angers, Angers, France

**Correspondence:** Bertrand Pavlovsky - bertrand.pavlovsky@gmail.com

*Annals of Intensive Care* 2021, **11(Suppl 1):**CO-071

**Rationale:** Various positive end-expiratory pressure (PEEP) titration strategies have been proposed to optimize ventilation in patients with acute respiratory distress syndrome (ARDS). This study aimed to compare four different strategies based on electrical impedance tomography (EIT) and respiratory mechanics with or without esophageal pressure measurement, and to assess the relationship between the determined PEEP levels and the potential for lung recruitment (PLR).

**Patients and methods/materials and methods:** 19 moderate-to-severe ARDS patients were enrolled in this monocentric study. Airway and esophageal pressure and EIT signal were continuously recorded during the study protocol. First, gas exchange and respiratory mechanics were assessed at PEEP 15 and 5 cmH2O. Then, a decremental PEEP trial from PEEP 20 to 5 cmH2O was performed. Optimal PEEP levels determined by the following strategies were determined as the one allowing to obtain: (1) plateau pressure < 28 cmH2O (Express); (2) tele-expiratory transpulmonary pressure > 0 cmH2O (PLe > 0); (3) center of ventilation closest to 0.5 (CoV) and (4) intersection of the overdistension and lung collapse curves (LC-OD) computed by EIT. PLR between PEEP 5 and 15 cmH2O was assessed using EIT-based recruitment-to-inflation ratio (R/IEIT), computed with the difference in end-expiratory lung volume (EELV) between the two PEEP levels estimated by EIT. Inspiratory transpulmonary plateau pressure (PLi) was calculated using the lung-to-respiratory system elastance ratio. The study population was divided in subgroups according to median values of PaO2/FiO2 and respiratory system compliance (CRS) at PEEP 5 cmH2O and R/IEIT.

**Results:** Median age was 64 [IQR 54–67] and SAPS II at admission was 45 [38–51]. At baseline, PaO2/FiO2 was 120 [91–177] mmHg and CRS was 50 [40–64] mL.cmH2O-1. R/IEIT was 0.67 [0.48–1.18]. The Express and CoV strategies led to higher PEEP levels than the PLe > 0 and LC-OD ones (17 [14–17], 20 [17–20], 5 [5–11], 10 [8–11] cmH2O, respectively, *p* < 0.001). Express and CoV strategies were associated with decreased CRS and increased overdistension, compared to the two other strategies. PLi was significantly higher with Express and CoV strategies (19 [16–22] and 23 [16–27] cmH2O, respectively) than with PLe > 0 and LC-OD (11 [8–15] and 14 [10–15] cmH2O, respectively, *p* < 0.001). There was no difference in optimal PEEP levels determined by each strategy between the groups defined according to PaO2/FiO2, CRS and PLR (Fig. 1).

**Conclusion:** PEEP titration strategies based on EIT, plateau pressure or expiratory trans-pulmonary pressure lead to different PEEP levels. PEEP levels determined by the different tested strategies are not related to PLR.

**Compliance with ethics regulations:** Yes in clinical research.
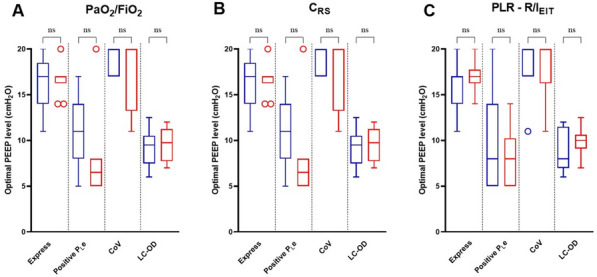


Fig. 1 Positive end-expiratory pressure (PEEP) determined according to the different titration strategies in patients with: **A** lower (red boxes) and higher PaO2/FiO2 ratio at baseline (blue boxes). **B** Lower (red boxes) and higher CRS (blue boxes) at ba

### CO-072 Reversible sepsis induced diaphragm dysfunction in critically ill patients

#### Marie Lecronier^1^, Boris Jung^2^, Thomas Similowski^1^, Jérôme Pinot^1^, Samir Jaber^2^, Alexandre Demoule^1^, Martin Dres^1^

##### ^1^CHU Pitié-Salpêtrière AP-HP, Paris, France; ^2^CHU de Montpellier, Montpellier, France

**Correspondence:** Marie Lecronier - lecronier.marie@gmail.com

*Annals of Intensive Care* 2021, **11(Suppl 1):**CO-072

**Rationale:** Whether sepsis-induced diaphragm dysfunction may improve despite the exposure of mechanical ventilation in critically ill patients is unclear. This study aims at describing the diaphragm function time course of mechanically ventilated patients depending on the presence of sepsis at the time of intubation.

**Patients and methods/materials and methods:** Bicentric retrospective observational study of mechanically ventilated patients in whom diaphragm function was assessed two times (within the 24 h after intubation and at recovery when patients could be switched to pressure support mode) by measuring the drop in endotracheal pressure induced by bilateral anterior phrenic nerve stimulation (Ptr, stim). In a subgroup of patients, diaphragm ultrasound was performed to measure the right hemidiaphragm thickness. Sepsis was defined according to the Sepsis-3 international guidelines.

**Results:** Ninety-two patients were enrolled in the study. Sepsis upon intubation was present in 51 (55%) patients, among them 40/51 (78%) had a microbiological evidence of infection. In septic patients, primary reason for ventilation was acute respiratory failure related to pneumonia (36/51; 71%). In non-septic patients, main reasons for ventilation were acute respiratory failure not related to pneumonia (16/41; 39%), coma (13/41; 32%) and cardiac arrest (6/41; 15%). Ptr, stim within 24 h after intubation was lower in septic patients as compared to non-septic patients: 6.3 (4.9–8.7) cmH2O vs. 9.8 (7.0–14.2) cmH2O (*p* = 0.004), respectively. The median (interquartile, IQR) duration of mechanical ventilation between first and second diaphragm evaluation was 4 (2–6) days in septic patients and 3 (2–4) days in non-septic patients (*p* = 0.07). Between two measurements, Ptr, stim significantly improved in septic patients, from 6.3 (4.9–8.7) to 7.9 (6.7–11.2) (*p* = 0.03), whereas it did not change in non-septic patients from 9.8 (7–14.2) to 7.3 (4.5–12.8) (*p* = 0.20). The change in Ptr, stim was +19% (−13–61) in septic patients and −7% (−40–12) in non-septic patients (*p* = 0.005). In a sub-group of patients with ultrasound measurements (58/92; 63%), end-expiratory diaphragm thickness significantly decreased in septic patients (232 (177–269) to 202 (155–231) (*p* = 0.04)), whereas it did not change in non-septic patients (213 (183–253) to 187 (160–224) (*p* = 0.16)).

**Conclusion:** Septic patients were associated with a significant increase in diaphragm function that was not observed in non-septic patients.

**Compliance with ethics regulations:** Yes in clinical research.

### CO-073 Reconnection to mechanical ventilation for 1 h after a successful spontaneous breathing trial in ICU: physiological effects on alveolar recruitment

#### Alice Lejars, Maeva Rodriguez, François Arrive, Faustine Reynaud, Florence Boissier, Anne Veinstein, Delphine Chatellier, René Robert, Jean-Pierre Frat, Arnaud W. Thille, Rémi Coudroy

##### CHU de Poitiers, Poitiers, France

**Correspondence:** Rémi Coudroy - r.coudroy@yahoo.fr

*Annals of Intensive Care* 2021, **11(Suppl 1):**CO-073

**Rationale:** As compared to direct extubation, reconnection to mechanical ventilation for 1 h after a successful spontaneous breathing trial (SBT) may be associated with lower reintubation rates (1). Whereas physiological explanation leading to these clinical effects remains unclear, we hypothesized that could be driven by alveolar recruitment induced by reconnection to mechanical ventilation. Our primary aim was to compare end-expiratory lung volume (EELV) measurements during the SBT and until 1 h after reconnection to mechanical ventilation.

**Patients and methods/materials and methods:** This is an ancillary study of a multicenter randomized controlled trial comparing T-piece versus pressure-support (pressure support of 8 cmH_2_O without positive end-expiratory pressure) for SBT before extubation. All patients included were at high-risk of extubation failure, i.e. intubated at least 24 h and older than 65 years or having underlying chronic cardiac or lung disease. This physiological study was performed in a single-center participating in the trial. EELV was measured using the nitrogen washin–washout technique before SBT under mechanical ventilation, at the end of the SBT, and then 10 min, and 1 h after reconnection to mechanical ventilation. SBT was performed for around 1 h using T-piece or low pressure-support levels according to the randomization.

**Results:** Sixteen SBTs performed in 13 patients were analyzed, including 9 (56%) SBTs performed using T-piece and 7 (44%) using pressure-support. SBT was successful in 75% of cases (12/16). Median [interquartile range] age was 73 years [69–75] and duration of mechanical ventilation was 12 days [7–15]. All in all, EELV was 1885 mL [1433–2088] before SBT and decreased by 36% [23–46] at the end of the SBT (*p* < 0.001). The decrease in EELV at the end of the SBT was greater using T-piece than using pressure-support (decrease by 46% [42–49] vs. 17% [13–29], *p* < 0.001). EELV significantly re-increased from 1236 mL [921–1531] at the end of the SBT to 1938 mL [1671–2604] after 1 h of reconnection to mechanical ventilation (*p* < 0.001). After only 10 min of reconnection to mechanical ventilation, 86% [71–101] of this increased EELV had already been recovered.

**Conclusion:** Performing a SBT induced a marked alveolar derecruitment which was significantly greater after SBT using T-piece than using pressure-support. This lung volume loss was completely recovered after 1-h reconnection to mechanical ventilation regardless the type of SBT, and was almost completely recovered after only 10 min of reconnection.

**Reference**

1. Fernandez MM et al. Reconnection to mechanical ventilation for 1 h after a successful spontaneous breathing trial reduces reintubation in critically ill patients: a multicenter randomized controlled trial. Intensive Care Medicine. 2017;43(11):1660–7.

**Compliance with ethics regulations:** Yes in clinical research.

### CO-074 Handgrip strength to predict extubation outcome: a prospective multicenter trial

#### Guillaume Cottereau^1^, Jonathan Messika^2^, Bruno Mégarbane^3^, Laurent Guérin^6^, Etienne De Montmollin^4^, Caroline Bornstain^5^, Benjamin Sztrymf^1^

##### ^1^Hôpital Antoine Béclère, Clamart, France; ^2^Hôpital Louis Mourier, Colombes, France; ^3^Hôpital Lariboisière, Paris, France; ^4^Hôpital Delafontaine, Saint Denis, France; ^5^Hôpital Intercommunal du Raincy Montfermeil, Le Raincy, France; ^6^Hôpital Bicêtre, Le Kremlin Bicêtre, France

**Correspondence:** Benjamin Sztrymf - benjamin.sztrymf@aphp.fr

*Annals of Intensive Care* 2021, **11(Suppl 1):**CO-074

**Rationale:** ICU-acquired weakness (ICUAW) has been shown to delay liberation from mechanical ventilation and to increase ICU length of stay. The Medical research Council (MRC) score is a bedside time-consuming test requiring experienced physiotherapists. Value of maximal handheld dynamometric strength measurement, handgrip strength (HG), has also been proven to be a reliable diagnostic tool of ICUAW. We aimed at testing the association between HG and extubation outcome.

**Patients and methods/materials and methods:** Prospective multicenter trial over an 18-month period in 6 ICUs. Adults receiving mechanical ventilation for at least 48 h were eligible. Just before weaning trial, HG, maximal inspiratory pressure (MIP), peak cough expiratory flow (PCEF) and MRC score were registered. The attending physicians were unaware of the tests results and weaning procedures were conducted according to guidelines. The main outcome was the link between handgrip strength and extubation outcome.

**Results:** 233 patients were included, median age 66 [53–75] years, 139 (59.6%) were men. Based on current consensus, weaning was defined as simple, difficult and prolonged in, respectively, 164 (70.4%), 49 (21%) and 18 (7.6%) patients. Extubation failure occurred in 51 (22.5%) patients, 39 (17.2%) required reintubation. Handgrip strength was 12 [6–20] kg and 12 [8–20] kg, respectively, in extubation success and failure (*p* = 0.85). There was no association between extubation outcome and MRC score, MRC score subgroup or dominant arm abduction angle. Handgrip strength was well correlated with MRC score (*r* = 0.718, *p* < 0.0001). Neither MIP, nor PCEF were associated with extubation outcome. Age was associated with extubation outcome (62.5 [53–73] years vs. 70 [58–78] years in extubation success and failure, respectively, *p* = 0.025). There was a non-significant trend toward a higher incidence of baseline respiratory comorbidities in the subgroup of patients experiencing extubation failure (41.2% vs. 29.6%, *p* = 0.12). ICU and hospital LOS were significantly higher in the subgoup of patients harboring muscular weakness as defined by handgrip strength performed at the first weaning trial (15 [10–25] days vs. 11 [7–17] days, *p* = 0.001 and 34 [19–66] days vs. 22 [15–43] days, respectively, *p* = 0.002).

**Conclusion:** No association was found between handgrip strength and extubation outcome. Whether this was explained by the appropriateness of the tool in this specific setting, or by the precise impact of ICUAW on extubation outcome deserves to be further evaluated.

**Compliance with ethics regulations:** Yes in clinical research.

### CO-075 Temporary transvenous diaphragm neurostimulation in mechanically ventilated patients: per protocol results from the RESCUE 2 randomized controlled trial

#### Dres Martin

##### Hôpital Pitié Salpêtrière, Paris, France

**Correspondence:** Martin Dres - martin.dres@aphp.fr

*Annals of Intensive Care* 2021, **11(Suppl 1):**CO-075

**Rationale:** Mechanical ventilation (MV), while lifesaving, can lead to ventilator-induced diaphragm dysfunction (VIDD) and be associated with difficulty in weaning and prolonged duration in the ICU. Rehabilitation of the diaphragm strength may thus improve the overall respiratory load/capacity balance and then reduce duration of MV. Temporary transvenous diaphragm neurostimulation (TTDN) using bilateral phrenic stimulation with a multi-electrode central venous catheter was used to assess clinical outcomes and respiratory function in a multicenter, open label, randomized controlled trial. In contrast to inspiratory muscle strength training, this therapy is standardized and does not depend on patients’ cooperation. Mechanical ventilation (MV), while lifesaving, can lead to ventilator-induced diaphragm dysfunction (VIDD) and be associated difficulty in weaning and prolonged duration in the ICU. Rehabilitation of the diaphragm strength may thus improve the overall respiratory load/capacity balance and then reduce duration of MV. Temporary transvenous diaphragm neurostimulation (TTDN) using bilateral phrenic stimulation with a multi-electrode central venous catheter was used to assess clinical outcomes and respiratory function in a multicenter, open-label, randomized controlled trial. In contrast to inspiratory muscle strength training, this therapy is standardized and does not depend on patients’ cooperation.

**Patients and methods/materials and methods:** Patients on invasive MV for > 96 h who failed at least two spontaneous breathing trials and satisfied readiness to wean criteria were randomized (1:1) to standard of care (SoC) or TTDN, consisting of up to 120 stimulations per day for up to 30 days. Clinical outcome measures included assessment of the proportion of successfully weaned patients, mean days on MV and 30-day survival. Respiratory function measures included maximal inspiratory pressure (MIP) and rapid shallow breathing index (RSBI).

**Results:** The study randomized 112 patients, 57 TTDN and 55 SoC. The stimulation catheter was successfully placed in 43 (75%) patients and at least 50% of the protocol-required stimulations were successfully delivered in 34 (60%) subjects. Table 1 shows the clinical and respiratory function outcomes (% or mean ± SD) of the per-protocol analysis subset. A stimulation dose–response relationship was shown for both MIP and RSBI in TTDN patients. There were no unanticipated adverse device effects noted in the study.

**Conclusion:** TTDN produced a significant diaphragm-related improvement in MIP and RSBI. While this study was not powered for the clinical outcomes, the study results contribute to defining the patient population and effect size to appropriately power future studies for clinical efficacy.

**Compliance with ethics regulations:** Yes in clinical research.
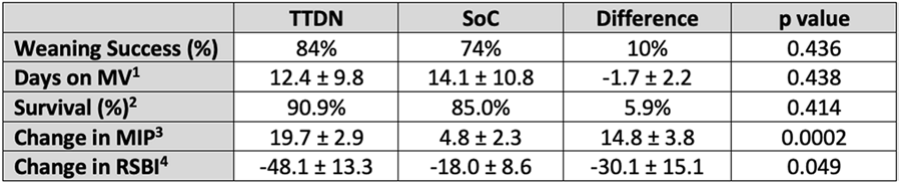


Table 1 Primary and secondary endpoints

MV: mechanical ventilation/MIP: maximal inspiratory pressure

RSBI: rapid shallow breathing index/TTDN: temporary transvenous diaphragm neurostimulation

SoC: standard of care

### CO-076 Non-invasive ventilation versus high-flow nasal oxygen after extubation in COPD patients: a post hoc analysis of a randomized controlled trial

#### Arnaud Thille^1^, Rémi Coudroy^1^, Mai-Anh Nay^2^, Arnaud Gacouin^3^, Maxens Decavèle^4^, Romain Sonneville^5^, François Beloncle^6^, Christophe Girault^7^, Laurence Dangers^8^, Alexandre Lautrette^9^, Quentin Levrat^10^, Anahita Rouzé^11^, Emmanuel Vivier^12^, Jean-Baptiste Lascarrou^13^, Jean-Damien Ricard^14^, Keyvan Razazi^15^, Guillaume Barberet^16^, Christine Lebert^17^, Stephan Ehrmann^18^, Alexandre Massri^19^, Jeremy Bourenne^20^, Gael Pradel^21^, Pierre Bailly^22^, Nicolas Terzi^23^, Jean Dellamonica^24^, Guillaume Lacave^25^, René Robert^1^, Stéphanie Ragot^1^, Jean-Pierre Frat^1^

##### ^1^CHU de Poitiers, Poitiers, France; ^2^CH d’Orléans, Orléans, France; ^3^CHU de Rennes, Rennes, France; ^4^Hôpital de La Pitié Salpétrière, Paris, France; ^5^Hôpital Bichat, Paris, France; ^6^CHU d’Angers, Angers, France; ^7^CHU de Rouen, Rouen, France; ^8^CHU Félix Guyon, Saint Denis De La Réunion, France; ^9^CHU de Clermont-Ferrand, Clermont-Ferrand, France; ^10^CH de La Rochelle, La Rochelle, France; ^11^CHU de Lille, Lille, France; ^12^Hôpital Saint Joseph - Saint Luc, Lyon, France; ^13^CHU de Nantes, Nantes, France; ^14^Hôpital Louis Mourier, Colombes, France; ^15^Hôpital Henri Mondor, Créteil, France; ^16^Groupe Hospitalier Régional Mulhouse Sud Alsace, Mulhouse, France; ^17^CH de Vendée, La Roche Sur Yon, France; ^18^CHRU de Tours, Tours, France; ^19^CH de Pau, Pau, France; ^20^CHU de Marseille, Marseille, France; ^21^CH d’Aurillac, Aurillac, France; ^22^CHU de Brest, Brest, France; ^23^CHU de Grenoble, Grenoble, France; ^24^CHU de Nice, Nice, France; ^25^CH de Versailles, Le Chesnay, France

**Correspondence:** Arnaud Thille - aw.thille@gmail.com

*Annals of Intensive Care* 2021, **11(Suppl 1):**CO-076

**Rationale:** Several randomized clinical trials have shown that non-invasive ventilation (NIV) applied immediately after extubation may prevent reintubation in patients at high risk of extubation failure. However, most of studies included patients with chronic respiratory disorders as well as patients without underlying respiratory disease. To date, no study has shown decreased risk of reintubation with prophylactic NIV after extubation among patients with chronic obstructive pulmonary disease (COPD). We hypothesized that prophylactic NIV after extubation may decrease the risk of réintubation in COPD patients as compared with high-flow nasal oxygen. We performed a post hoc subgroup analysis of COPD patients included in a multicenter, randomized, controlled trial comparing prophylactic use of NIV alternating with high-flow nasal oxygen versus high-flow nasal oxygen alone immediately after extubation.

**Patients and methods/materials and methods:** We performed a post hoc subgroup analysis of COPD patients included in a multicenter, randomized, controlled trial comparing prophylactic use of NIV alternating with high-flow nasal oxygen versus high-flow nasal oxygen alone immediately after extubation

**Results:** Among the 651 patients included in the original study, 150 (23%) had underlying COPD including 86 patients treated with NIV and 64 patients treated with high-flow nasal oxygen. The reintubation rate was 13% (11 out of 86 patients) with NIV and 27% (17 out of 64 patients) with high-flow nasal oxygen (difference, − 14% [95% CI − 27% to − 1%]; *p* = 0.03). Whereas reintubation rates were significantly lower with NIV than with high-flow nasal oxygen alone at 72 h and until ICU discharge, mortality in ICU did not differ between groups: 6% (5/86) with NIV vs. 9% (6/64) with high-flow nasal oxygen (difference − 4% [95% CI − 14% to 5%]; *p* = 0.40).

**Conclusion:** Compared with high-flow nasal oxygen alone, in COPD patients, prophylactic NIV significantly decreased the risk of reintubation.

**Compliance with ethics regulations:** Yes in clinical research.

### CO-077 Study of the dyspnea and the electromyographic activity of the diaphragm and extradiaphragmatic inspiratory muscles during weaning from mechanical ventilation

#### Côme Bureau, Martin Dres, Laurence Dangers, Julien Mayaux, Elise Morawiec, Thomas Similowski, Alexandre Demoule

##### ^1^Groupe Hospitalier Universitaire APHP - Sorbonne Université Site Pitié-Salpêtrière, Paris, France

**Correspondence:** Côme Bureau - come.bureau@gmail.com

*Annals of Intensive Care* 2021, **11(Suppl 1):**CO-077

**Rationale:** Success or failure of the spontaneous breathing trial (SBT) is defined by objective and subjective criteria of acute respiratory failure (ARF) (1). Although dyspnea is a key symptom of ARF, dyspnea is not considered as common criteria of SBT success or failure. Here, we assessed dyspnea during SBT. Our first objective was to determine whether dyspnea is a reliable criterion of weaning success or failure. Our second objective was to quantify during SBT the relationship between dyspnea and the respective electromyographic activity (EMG) of the diaphragm (EMGdi) and of the alae nasi (EMGan), an extradiaphragmatic inspiratory muscle.

**Patients and methods/materials and methods:** Patients hospitalized in an intensive care unit and in whom a SBT was initiated by the physician in charge were included in the study. Were continuously recorded during the 30-min SBT or until SBT failure: airway flow and pressure, EMGdi with nasogastric probe and EMGan with surface electrodes. Dyspnea intensity was assessed by the Dyspnea—Visual Analogic Scale (Dyspnea-VAS) at initiation and end of the SBT. At the end of the SBT, patients were classified as success or failure (1).

**Results:** Thirty-one patients were included, 71% male, aged (median [interquartile range]) 65 [61–71] years, SAPS2 53 [37–74]. They were invasively mechanically ventilated for 6 [3–10] days. Seventeen patients succeeded SVT. Baseline Dyspnea-VAS was similar in patients who succeeded (0 [0–2]) and in those who failed (2 [2–4] cm, *p* = 0.18) SBT. Dyspnea-VAS at the end of SBT was lower in patients who succeeded (0 [0–4] vs 10 [8–10] cm, *p* = 0.03). The increase in Dyspnea-VAS was lower in those who succeeded (0 [0–1] vs 6 [4–8] cm, *p* = 0.01). The area under the curve of Dyspnea-VAS was 0.909. During SBT, the EMGan increased was lower in patients who succeeded (0.93 [0.6–1.1] vs 1.43 [0.91–2.4], *p* = 0.01). The EMGdi increased was lower in patients who succeeded (0.87 [0.62–1.64] vs 1.87 [1.20–2.92], *p* = 0.02). The increase in Dyspnea-VAS was significantly correlated to the increase in EMGan (Rho = 0.43 [0.05–0.71], *p* < 0.05), but was not correlated to EMGdi (Rho = − 0.26 [− 0.68–0.28], *p* = 0.33).

**Conclusion:** During an SBT, dyspnea is a reliable marker of weaning success and failure, suggesting that Dyspnea-VAS could be used as a monitoring tool of SBT. In addition, dyspnea seems to be more closely related to the EMG activity of extradiaphragmatic inspiratory muscles than the diaphragm.

**Reference**

1. Boles J-M, Bion J, Connors A, Herridge M, Marsh B, Melot C, et al. Weaning from mechanical ventilation. Eur Respir J Off J Eur Soc Clin Respir Physiol. 2007;29(5):1033–56.

**Compliance with ethics regulations:** Yes in clinical research.

### CO-078 Non-invasive ventilation versus high-flow nasal oxygen for postextubation respiratory failure in ICU: a post hoc analysis of a randomized clinical trial

#### Arnaud Thille^1^, Grégoire Monseau^1^, Rémi Coudroy^1^, Mai-Anh Nay^2^, Arnaud Gacouin^3^, Maxens Decavèle^4^, Romain Sonneville^5^, François Beloncle^6^, Christophe Girault^7^, Laurence Dangers^8^, Alexandre Lautrette^9^, Quentin Levrat^10^, Anahita Rouzé^11^, Emmanuel Vivier^12^, Jean-Baptiste Lascarrou^14^, Jean-Damien Ricard^14^, Keyvan Razazi^15^, Guillaume Barberet^16^, Christine Lebert^17^, Stephan Ehrmann^18^, Alexandre Massri^19^, Jeremy Bourenne^20^, Gael Pradel^21^, Pierre Bailly^22^, Nicolas Terzi^23^, Jean Dellamonica^24^, Guillaume Lacave^25^, René Robert^1^, Stéphanie Ragot^1^, Jean-Pierre Frat^1^

##### ^1^CHU de Poitiers, Poitiers, France; ^2^CH d’Orléans, Orléans, France; ^3^CHU de Rennes, Rennes, France; ^4^Hôpital de La Pitié Salpétrière, Paris, France; ^5^Hôpital Bichat, Paris, France; ^6^CHU d’Angers, Angers, France; ^7^CHU de Rouen, Rouen, France; ^8^CHU Félix Guyon, Saint Denis De La Réunion, France; ^9^CHU de Clermont-Ferrand, Clermont-Ferrand, France; ^10^CH de La Rochelle, La Rochelle, France; ^11^CHU de Lille, Lille, France; ^12^Hôpital Saint Joseph - Saint Luc, Lyon, France; ^13^CHU de Nantes, Nantes, France; ^14^Hôpital Louis Mourier, Colombes, France; ^15^Hôpital Henri Mondor, Créteil, France; ^16^Centre hospitalier Mulhouse Sud Alsace, Mulhouse, France; ^17^CH de Vendée, La Roche Sur Yon, France; ^18^CHRU de Tours, Tours, France; ^19^CH de Pau, Pau, France; ^20^CHU de Marseille, Marseille, France; ^21^CH d’Aurillac, Aurillac, France; ^22^CHU de Brest, Brest, France; ^23^CHU de Grenoble, Grenoble, France; ^24^CHU de Nice, Nice, France; ^25^CH de Versailles, Le Chesnay, France

**Correspondence:** Arnaud Thille - aw.thille@gmail.com

*Annals of Intensive Care* 2021, **11(Suppl 1):**CO-078

**Rationale:** In intensive care units (ICUs), patients experiencing post-extubation respiratory failure have poor outcomes. In this setting, the use of noninvasive ventilation (NIV) to treat post-extubation respiratory failure may increase the risk of death. This study aims at comparing mortality between patients treated with NIV or high-flow nasal oxygen alone for post-extubation respiratory failure.

**Patients and methods/materials and methods:** Post hoc analysis of a multicenter, randomized, controlled trial comparing prophylactic use of NIV alternating with high-flow nasal oxygen versus high-flow nasal oxygen alone immediately after extubation in 641 patients at high risk of extubation failure in 30 French ICUs. The present study is a subgroup analysis focusing on patients who experienced post-extubation respiratory failure within the 7 days following extubation. The choice of oxygenation strategy to treat post-extubation respiratory failure was left to the physician’s decision. Patients were classified in the NIV group or the high-flow nasal oxygen group according to oxygenation strategy used after the onset of post-extubation respiratory failure. Patients reintubated within the first hour after extubation and those promptly reintubated without prior treatment were excluded. The primary outcome was mortality at day 28 after the onset of post-extubation respiratory failure.

**Results:** Among 651 extubated patients, 158 (25%) experienced respiratory failure and 146 were included in the analysis. Mortality at day 28 was 18% (15/84) with NIV and 29% (18/62) with high-flow nasal oxygen (difference, − 11% [95% CI, − 25 to 2]; *P* = 0.1214). Among the 46 patients with hypercapnia at the onset of respiratory failure, mortality at day 28 was 3% (1/33) with NIV and 31% (4/13) with high-flow nasal oxygen alone (difference, − 28% [95% CI, − 54 to − 6]; *P* = 0.0060). The proportion of patients reintubated 48 h after the onset of post-extubation respiratory failure was 44% (37/84) with NIV and 52% (32/62) with high-flow nasal oxygen (*P* = 0.2174).

**Conclusion:** In patients with post-extubation respiratory failure, NIV use did not increase the risk of death.

**Compliance with ethics regulations:** Yes in clinical research.

### CO-079 Contribution of imaging in the diagnostic of cerebral thrombosis related to snakebite envenoming by *Bothrops lanceolatus* in Martinique: about 16 cases

#### Dabor Resiere, Jules Rey, Laura Cerland, Jonathan Florentin, Cyrille Chabartier, Remi Neviere, Bruno Megarbane, Hatem Kallel, Hossein Mehdaoui

##### CHU de Martinique, Fort-De-France, Martinique

**Correspondence:** Dabor Resiere - dabor.resiere@chu-martinique.fr

*Annals of Intensive Care* 2021, **11(Suppl 1):**CO-079

**Rationale:** Snakebites due to *Bothrops lanceolatus* (Bl) represent frequent medical morbidity requiring admission to the emergency department and intensive care unit, with an average of 25–30 cases per year. Any bite by this unique snake in Martinique may result in severe thrombotic complications such as cerebral infarction, pulmonary or myocardial, involving life or functional prognosis. The composition of the venom and the lesion mechanisms are currently being assessed. Specific antivenom immunotherapy (AVI), Bothrofav^®^ 2, available since 2011, has resulted in a significant reduction in mortality and morbidity including ischemic strokes induced by envenoming. This study aimed to evaluate the contribution of imaging in the diagnosis of cerebral thrombosis in the envenomation by the bite of Bl.

**Patients and methods/materials and methods:** We conducted an observational, retrospective study over a period of 8 years between 2011 and 2019, including all successive patients admitted to the department of emergency medicine and intensive care at the University Hospital of Martinique for snakebites by Bl. The radiological data were completed and analyzed, via medical records using emergency software, Dx Care, X-plore, and Cyberlab.

**Results:** One hundred and eighty-nine patients were included in this study, 133 men and 56 women. Among these patients, 39 were grade III or IV only and 16 had an MRI. No thrombotic cerebrovascular accident was noticed.

**Discussion:** The indication to carry out a cerebral MRI systematically in the management of snakebites by Bl remains questionable.

**Conclusion:** This study showed that patients who received early Bothrofav2 antivenom do not develop thrombosis. Due to our underpowered study with methodological limitations, MRI should be limited to patients with grade 3 or 4 envenomation by *Bothrops lanceolatus*.

**Reference**

1. Resiere D, Thomas L and al. Bothrops lanceolatus bites: guidelines for severity assessment and emergent management. Toxins 2010, 2:163–73; 10.3390/toxins2010163.

**Compliance with ethics regulations:** Yes in clinical research.

### CO-080 Lung ultrasound as a triage tool in patients suspected of Covid-19

#### Taha Hounain, Khalid Kabba, Naila Boukoub, Anas Auhmani, Hamza Elhamzaoui, Taoufik Abouelhassan

##### CENTRE HOSPITALIER UNIVERSITAIRE MOHAMMED VI DE MARRAKECH, Marrakech, Maroc

**Correspondence:** Taha Hounain - taha.hounain.doc@gmail.com

*Annals of Intensive Care* 2021, **11(Suppl 1):**CO-080

**Rationale:** The pandemic of COVID-19 is seriously challenging the medical organization in many parts of the world. The rapid spread of the infection during this pandemic lead to to hospitalize a high number of patients. Lung ultrasound (LUS) may allow reliable characterization and helps in triaging and admitting patients. It can be carried out in 5 min, without radiation exposure, at the patient’s bedside, limiting the movement of patients and cross-contamination of both patients and nursing staff. The aim of this study is to identify the sonographic profile of patients suspected of covid-19 admitted to the emergency room (ER).

**Patients and methods/materials and methods:** This prospective study enrolled 88 patients with a suspected COVID-19 infection, admitted to the ER between March 15th and June 15th, 2020. Patients showed one or more symptoms of an acute respiratory infection, for which consequent COVID-19 testing was achieved using POCUS lung, chest CT, and RT-PCR. Radiologists blinded to RT-PCR results analyzed CT images. Two experienced intensivists performed LUS, and reports were analyzed by the researcher, blinded to clinical information, US imaging, CT, and RT-PCR test results. We excluded patients transferred to another hospital or lost to follow-up.

**Results:** Patients mean age was 45.14 years. 63.6% (56/88) of patients were male. At admission, the most common clinical symptoms were flu-like syndrome (47.7% (42/88)) and dyspnea (34% (30/88)). RT-PCR was positive in 69.3% (61/88) of all patients. Chest CT results were standardized using the CO-RADS classification and were as follows: Co-RADS I–III (69.3% (61/88)) and CO-RADS IV–V (30.6% (27/88)). The most common LUS findings were patchy confluent B-lines, found in half of the patients (44/88), and sub-pleural consolidations in 26% (23/88), findings were bilateral in 36.3% (32/88). LUS was normal in 22.7% (20/88). Of note, all patients who required immediate intensive care hospitalization, 13.63% (12/88) had positive LUS findings.

**Conclusion:** LUS may allow early triage in the ER with a low-cost, rapid, radiation-free and safe screening tool in the evaluation of a possible COVID-19 infection.

**References**

1. Narinx N., Smismans A., Symons R., Frans J., Demeyere A., Gillis M. Feasibility of using point-of-care lung ultrasound for early triage of COVID-19 patients in the emergency room. Emerg. Radiol. 2020.
2. Karagöz A, Sa?lam C, Demirba? HB, Korkut S, Ünlüer EE. Accuracy of Bedside Lung Ultrasound as a Rapid Triage Tool for Suspected Covid-19 Cases. Ultrasound Q. 2020.

**Compliance with ethics regulations:** Yes in clinical research.

### CO-081 Alcohol withdrawal syndrome in ICU patients: clinical features, management, and predictors of outcome

#### Aliénor Vigouroux, Charlotte Garret, Jean-Baptiste Lascarrou, Maelle Martin, Arnaud-Felix Miailhe, Jérémie Lemarié, Olivier Zambon, Amélie Seguin, Jean Reignier, Emmanuel Canet

##### CHU Nantes Hôtel-Dieu, Nantes, France

**Correspondence:** Aliénor Vigouroux - alienor.vigouroux@gmail.com

*Annals of Intensive Care* 2021, **11(Suppl 1):**CO-081

**Rationale:** Alcohol withdrawal syndrome (AWS) is common in hospitalized patients, yet its epidemiology in the ICU remains poorly characterized.

**Patients and methods/materials and methods:** Retrospective cohort of patients admitted to the ICU of a French University Hospital between January 1, 2017 and December 31, 2019 and coded for AWS at discharge using the ICD-10 criteria. The primary objective of the study was to identify factors associated with complicated AWS. Complicated AWS was defined as: ICU length of stay or mechanical ventilation (MV) ≥ 7 days, or hospital mortality.

**Results:** Among 5641 patients admitted to ICU during the study period, 246 (4.4%) were coded as having AWS. After detailed analysis of the medical notes, 42 patients had exclusion criteria and 204 patients were included in the study. One-third of patients had pre-existing psychiatric disease and one-fifth reported a past history of AWS. Median daily alcohol intake was 129 (72–216) grams. The three primary reasons for ICU admission were sepsis (29.9%), awareness disorders (29.4%), and seizures (24%). At ICU admission, median Cushman score was 6 (4–9), median SOFA score was 3 (2–6), and median Glasgow Coma Scale was 14 (12–15). Delirium tremens occurred in just over half the patients and one-fifth developed seizures. One in every three patients had pneumonia. During the ICU stay, 42.2% of patients required MV. Overall, 48% of patients developed complicated AWS, of whom 92.8% stayed in the ICU than 7 days or more, 36.7% received MV for 7 days or more, and 16.3% died during hospital stay. On multivariable analysis, only two factors were independently associated with complicated AWS. The number of organ dysfunctions at ICU admission increased the risk of complicated AWS (OR 1.18; 95 CI 1.05–1.32, *p* = 0.005), while ICU admission for seizures was associated with a lower risk of complicated AWS (OR 0.14; 95% CI 0.026–0.80, *p* = 0.026).

**Conclusion:** AWS in ICU patients is a syndrome affecting young patients with few co-morbidities, frequently triggered by a precipitating factor such as sepsis, trauma or surgery. Despite having low severity scores at ICU admission, half the patients experienced extended ICU stay, prolonged mechanical ventilation, or died during hospital stay. The likelihood of developing complicated AWS relied on the reason for ICU admission and the number of organ dysfunctions at ICU admission, while AWS history, the Cushman score, and delirium tremens had no impact.

**Compliance with ethics regulations:** Yes in clinical research.

### CO-082 Extracorporeal cardiopulmonary resuscitation candidates at the time of emergency call: characteristics of patients finally not supported

#### Marion Cholley, Gilles Capellier, Hadrien Winiszewski, Lydie Bretillot, Marc Puyraveau, Thibaut Desmettre

##### CHRU Besancon, Besancon, France

**Correspondence:** Marion CHolley - mcholley@chu-besancon.fr

*Annals of Intensive Care* 2021, **11(Suppl 1):**CO-082

**Rationale:** Improving early identification of extracorporeal cardiopulmonary resuscitation (eCPR) candidates is a key issue to decrease mortality of patients with out of hospital refractory cardiac arrest (OHCA). Objective of the study was to describe the characteristics of patients eligible to eCPR at the time of emergency call and finally not supported.

**Patients and methods/materials and methods:** In this retrospective observational study, we enrolled patients with OHCA eligible to eCPR at the time of emergency call and finally not supported. Eligibility to eCPR at the time of emergency call was defined by age between 18 and 65, and witnessed refractory OHCA, and suspected cardiac origin or hypothermia or drowning. Refractory OHCA was defined as the absence of a return of spontaneous circulation after at least 15 min of conventional cardiopulmonary resuscitation (CPR). Patients with severe comorbidities, traumatic, neurologic, or toxic etiology were not eligible. At the time of operating room admission, only patients with low flow duration less than 150 min were still eligible.

**Results:** From January 2018 to December 2019, 50 patients with OHCA were eligible to eCPR at the time of emergency call, and 19 were finally supported by eCPR. Among the 31 patients finally not supported by eCPR, median age was 52 years [min–max 35–64], 22 (77%) were males and 13 (42%) had initial shockable rhythm. Suspected origin of arrest was cardiac in 17 patients (55%), drowning in 3 (10%), hypothermia in 1 (3%), and was undetermined in 10 patients (32%). Median no flow duration was 3 min [min max 00; 27:00]. Median time from collapse and starting CPR by emergency medical unit was 27 min [min max 09:00;50:00]. Median time from collapse to decision to stop resuscitation was 67 min [min max 24:13;136:57]. Reasons for not attempting eCPR were low-flow duration, low end tidal CO2 and persistence of a non-shockable rhythm per CPR.

**Conclusion:** Low-flow duration was the main reason for not attempting eCPR in patients with OHCA eligible at the time of emergency call. Improving the process (on site, multiple phone calls, secondary transport team mobilization) will increase the number of eligible OHCA to eCPR.

**Compliance with ethics regulations:** Yes in clinical research.

### CO-083 Prognostic factors in calcium-channel blocker poisonings managed medically or with veno-arterial extracorporeal membrane oxygenation: an observational cohort study

#### Sebastian Voicu, Isabelle Malissin, Nicolas Deye, Laetitia Sutterlin, Aymen Mrad, Naim Giulia, Adrien Pepin-Lehalleur, Thomas Lacoste-Palasset, Bruno Megarbane

##### Lariboisière, APHP, Paris, France

**Correspondence:** Sebastian Voicu - sebastoso@yahoo.com

*Annals of Intensive Care* 2021, **11(Suppl 1):**CO-083

**Rationale:** Calcium-channel blocker (CCB) poisoning is the main cause of cardiotoxicant-attributed death. However, prognostic factors have been poorly studied while essential to the decision of veno-arterial extracorporeal membrane oxygenation (VA-ECMO) implementation. Here, we aimed to evaluate mortality in CCB-poisoned patients and determine predictors of death due to CCB-attributed shock.

**Patients and methods/materials and methods:** We performed a single-centre observational study including all CCB-poisoned patients admitted to our ICU between January 2000 and December 2020. We retrospectively recorded clinical, laboratory, electrocardiographic and echocardiographic data. Catecholamine dose was expressed as the sum of epinephrine + norepinephrine + isoprenaline infusion rate (in mg/h). CCB-attributed death (CCB-death) was defined as mortality resulting from persistent shock due to CCB despite adequate fluid, catecholamine and antidotal treatment (1). The study was approved by our institutional ethics committee. Continuous variables were compared using Mann–Whitney and categorical variables using Fisher’s exact tests. Based on logistic univariable and multivariable analyses, we identified factors associated with CCB-death. Data are expressed as median [IQR] and numbers (%).

**Results:** Two hundred and sixty-three patients (age, 51 years [40–64]; 147 female) were included. Ingested CCB doses were 11-fold [5–23] the recommended daily doses. Additional cardiotoxicants were co-ingested in 103 patients (39%). Initial blood lactate concentration was 3.6 mmol/L [1.9–6.6], longest QRS duration 0.08 s [0.08–0.11] and lowest left ventricular ejection fraction (LVEF) 60% [55–65]. We observed no death in the 53 patients without shock during the ICU stay; 11 deaths [7%, from CCB-attributed shock (*N* = 5) and other causes (*N* = 6)] in the 169 patients with shock without VA-ECMO; and 21 deaths (51%) in the 41 VA-ECMO-treated patients. All nine patients admitted with refractory cardiac arrest died. In VA-ECMO-free patients with shock, survivors had lower peak catecholamine doses (7 mg/h [3–15] vs 32 mg/h [30–60], *p* = 0.0007), higher LVEF (60% [60–70] vs 50% [50–50], *p* = 0.0016) and less frequently cardiac arrest (3/158 (1.9%) vs 2/5 (40%), *p* < 0.0001) than non-survivors due to CCB-death. In multivariable analysis, peak catecholamine dose (OR, 1.06; 95% CI, 1.01–1.11) and LVEF (0.81; 95% CI, 0.68–1.07) were the only predictors significantly associated with CCB-death (area under the curve of the model, 0.97; 95% CI, 0.91–0.99, *p* = 0.0001). According to the model, patients with 40% LVEF and 15 mg/h catecholamines and patients with 50% LVEF and 45 mg/h catecholamines have a 50%-risk of CCB-death.

**Conclusion:** Overall mortality in CCB-poisoned patients managed in the ICU is 7% in patients with shock not requiring VA-ECMO and 51% in patients requiring VA-ECMO. Predictors of CCB-death are decreased LVEF and peak catecholamine dose, which should be taken into account in the decision to implement VA-ECMO.

**Reference**

1. Mégarbane B, Oberlin M, Alvarez JC, et al. Management of pharmaceutical and recreational drug poisoning. Ann Intensive Care. 2020;10(1):157.

**Compliance with ethics regulations:** Yes in clinical research.

### CO-084 Feasibility of prone position inter-hospital transportation for severe acute respiratory distress syndrome by a dedicated emergency medical team

#### Clément Brault^1^, Pierre Gosset^1^, Emilien Arnaud^1^, Yoann Zerbib^1^, Loay Kontar^1^, Olivier Bonef^2^, Antoine Riviere^3^, Jérome Lepretre^4^, Michel Slama^1^, Carole Amsallem^5^, Christophe Boyer^1^, Julien Maizel^1^

##### ^1^CHU Amiens, Amiens, France; ^2^CH Saint Quentin, Saint Quentin, France; ^3^CH Abbeville, Abbeville, France; ^4^CH Montreuil, Rang Du Fliers, France; ^5^Simusante, Amiens, France

**Correspondence:** Julien Maizel - maizel.julien@chu-amiens.fr

*Annals of Intensive Care* 2021, **11(Suppl 1):**CO-084

**Rationale:** During the COVID-19 pandemia, many patients in severe ARDS required transport to a tertiary medical center due to capacity issues or lack of advanced critical care therapies. Usually patients are transported only in supine position by emergency medical services and may require the implantation of ECMO before transportation. However, many patients improving their pO2/FiO2 in prone position would not require the implantation of ECMO if we could transport the patient in prone position.

**Patients and methods/materials and methods:** Patients with a pO2/FiO2 in supine position between 60 and 80 due to severe ARDS with appropriate ventilatory settings and requiring transport to our medical ICU (Amiens university hospital) were prospectively screened. Patients with a pO2/FiO2 > 70 mmHg in prone position and without high posology of norepinephrine and not indicated for immediate ECMO implantation were included. Transports were performed by a dedicated team (a nurse, an emergency and a critical care physicians) previously trained to a specific procedure for prone position transport in ambulance and/or helicopter.

**Results:** We report here the first four patients transported in prone position. Two were transported by helicopter and 2 by ambulance. They were all male with a median age of 56 (45–71) y/o admitted for an ARDS due to SARS-Cov2. Before transportation the median PO2/FiO2 was 70 (63–77) in supine and 102 (95–134) in prone position. All patients received NO, were sedated and received neuromuscular blockers. The median ventilatory settings during transportation were tidal volume of 6 ml/kg (5.5–6.3), a PEEP of 12 cmH2O (12–13), driving pressure 14 cmH2O (13–19). The median time to prepare the patient was 72 min (63–79) and the transport lasted for 60 min (38–77). During the transport only one patient experienced a desaturation below 85%, no other adverse event was reported (hypotension, unintended dislodgement of device or cardiac arrest). Later one patient received ECMO, and one patient extracorporeal CO2 removal. Two patients died before ICU discharge.

**Conclusion:** Air and ground transport of severe ARDS patients in prone position appear feasible when performed by a dedicated team trained to an established protocol.

**Compliance with ethics regulations:** Yes in clinical research.

### CO-085 Patterns of ICU admissions and outcomes in patients with solid tumours: a 12-year retrospective study

#### Clara Vigneron^1^, Julien Charpentier^1^, Sandrine Valade^3^, Jean-Paul Mira^1^, Jérôme Alexandre^1^, Valérie Laurence^4^, François Goldwasser^1^, Matthieu Jamme^2^, Frédéric Pène^1^

##### ^1^Hôpital Cochin, Paris, France; ^2^Centre Hospitalier Poissy Saint Germain en Laye, Poissy, France; ^3^Hôpital Saint-Louis, Paris, France; ^4^Institut Curie, Paris, France

**Correspondence:** Clara Vigneron - claravigneron@hotmail.fr

*Annals of Intensive Care* 2021, **11(Suppl 1):**CO-085

**Rationale:** The epidemiology and outcome of cancer have changed over the past two decades with increased incidence but improved survival due to recent therapeutic advances. Immunotherapy and targeted therapy led to a better prognosis for several solid tumours, but can also cause new life-threatening toxicities. The objective of this study was to address the trends in admission patterns and outcomes in patients with solid tumours admitted to a medical ICU.

**Patients and methods/materials and methods:** We conducted a retrospective monocenter study including patients with solid tumors requiring unplanned admission to a medical ICU over a 12-year period (2007–2018). Main causes of admission patterns were distributed in three different patterns: specific complications (directly linked to underlying malignant disease), iatrogenic complications (side effects of antitumoral drugs or of diagnostic or therapeutic procedures) and non-specific complications. Independent predictors of short-term (in-ICU) and long-term (1-year) outcome were addressed by a cause-specific multivariate Cox regression analysis.

**Results:** 1525 patients (median age 67 years) were analysed. The leading malignancy sites were the lung, the gastrointestinal tract and the urinary tract. The number and the proportion of patients with solid tumors in the ICU increased over the 12-year period, with increasing proportion of patients with metastatic disease (60.2% in 2017–2018 vs 48.6% in 2007–2008, *p* = 0.004) and progression under treatment (53.3% vs 38.5%, *p* = 0.001). Admissions related to iatrogenic complications increased from 8.8% in 2007–2008 to 16.0% in 2017–2018 (*p* = 0.01). ICU, hospital and one-year mortality rates were 22.6%, 39.6% and 55.9% without any significant changes over time. In multivariate analysis, factors associated with ICU-mortality were metastatic disease, cancer in progression under treatment, need for invasive or non-invasive ventilation or inotropes/vasopressors and admission for specific complications. Patients with urologic cancer displayed better in-ICU survival. One-year mortality in ICU survivors is displayed by type of cancer in Fig. 1 and remained stable over time. It was independently associated with the type of cancer, lung cancer harbouring the worst prognosis, metastatic disease, cancer in progression under treatment, admission for specific complications and decision to forgo life-sustaining therapies at the time of ICU discharge.

**Conclusion:** Patients with solid tumors account for a growing proportion of unplanned ICU admissions. Advances in the management of cancer have modified the characteristics of ICU-admitted patients, with more advanced and often metastatic diseases albeit without impairments in the short-term nor in long-term survival. Thus, about three out of four patients survive the ICU stay and almost half of them are still alive at 1 year.

**Compliance with ethics regulations:** Yes in clinical research.
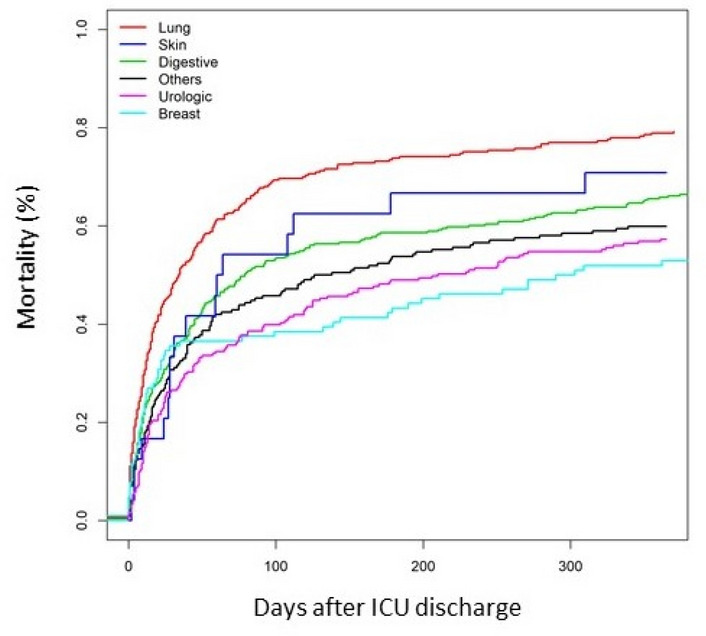


One-year mortality in ICU-survivors according to the type of cancer

### CO-086 Diagnostic yield, therapeutic impact and safety of bronchoalveolar lavage performed at ICU admission for acute respiratory failure in patients with haematological malignancies

#### Vincent Bonny^1^, Jacques Tankovic^1^, Jean Luc Baudel^1^, Guillaume Dumas^2^, Paul Gabarre^1^, Geoffroy Hariri^1^, Jean-Rémi Lavillegrand^1^, Sandie Mazerand^1^, Tomas Urbina^1^, Hafid Ait-Oufella^1^, Bertrand Guidet^1^, Eric Maury^1^, Naïke Bigé^1^

##### ^1^Hôpital Saint Antoine, Paris, France; ^2^Hôpital Saint-Louis, Paris, France

**Correspondence:** Vincent Bonny - vincent.bonny@aphp.fr

*Annals of Intensive Care* 2021, **11(Suppl 1):**CO-086

**Rationale:** Acute respiratory failure (ARF) is the leading cause of intensive care unit (ICU) admission in patients with haematological malignancies (HM). Fungal infections and undetermined etiology are associated with increased mortality. Therefore, diagnostic strategy of ARF in HM patients represents a major issue. Broncho-alveolar lavage (BAL) might be useful, but may worsen respiratory status. The present study aimed at describing diagnostic yield, therapeutic impact and safety of BAL performed at ICU admission for ARF in HM patients.

**Patients and methods/materials and methods:** Monocentric retrospective cohort study including consecutive HM patients who underwent BAL within the first 24 h of ICU admission for ARF between January 2015 and March 2019.

**Results:** One hundred and three patients—median age 62 [55–75] years—were included. Median time from first symptoms to ICU admission was 4 [1–7] days. Median SOFA score at ICU admission was 9 [6–13]. Forty-nine (48%) patients were receiving standard oxygen, five (5%) high-flow nasal oxygen (HFNO), one (1%) non-invasive ventilation (NIV), two (2%) NIV and HFNO, and 46 (45%) patients were intubated. BAL led to 84 diagnoses in 61 (59%) patients. Infectious pneumonia constituted the main diagnosis: bacterial (*n* = 46), viral (*n* = 31), fungal (*n* = 8), tuberculosis (*n* = 1). Other etiologies were the following: disease-related infiltration (*n* = 3), diffuse alveolar hemorrhage (*n* = 2) and drug-induced pulmonary toxicity (*n* = 1). Ninety (87%) patients received antibiotics with a median duration of 6 [2–13] days before BAL. Bacteriologic yield was significantly higher when patients did not receive antibiotics before BAL or when antibiotics duration was less than 2 days (60% vs 34%, *P* = 0.009). BAL led to a modification of treatment in 60 (58%) patients: broadening of antimicrobial spectrum (16%), narrowing of antimicrobial spectrum (41%), toxic drug withdrawal (2%). Deterioration of respiratory status occurred after BAL in 28 patients (51%) spontaneously breathing patients and in 4 (9%) intubated patients (*P* < 0.001). Intubation was required after BAL in 14 (25%) patients. Worsening of respiratory status was also more frequent in patients admitted more than 2 days after first symptoms’ onset than in those admitted earlier (40% vs 20%, *P* = 0.029).

**Conclusion:** BAL represents an important tool in the diagnostic strategy of ARF in HM patients. However, safety issues should be taken into account in the evaluation of benefit-to-risk balance in spontaneously breathing patients. Early realization of BAL after respiratory symptoms onset and antibiotics initiation seems to be associated with better diagnostic yield and safety.

**Compliance with ethics regulations:** Yes in clinical research.

### CO-087 Clinical features and outcome of patients with primary central nervous system lymphoma admitted to the intensive care unit: a French national expert center experience

#### Maxens Decavèle^1^, Aliénor Dreyfus^1^, Nicolas Gatulle^1^, Nicolas Gatulle^1^, Caroline Houillier^1^, Sophie Demeret^1^, Julien Mayaux^1^, Isabelle Rivals^2^, Martin Dres^1^, Julie Delemazure^1^, Elise Morawiec^1^, Charles-Edouard Luyt^1^, Khe Hoang-Xuan^1^, Sylvain Choquet^1^, Thomas Similowski^1^, Alexandre Demoule^1^

##### ^1^APHP - Hôpital Pitié-Salpêtrière, Paris 13, France; ^2^ESPCI Paris-PSL, Paris, France

**Correspondence:** Maxens Decavèle - maxencesar@hotmail.fr

*Annals of Intensive Care* 2021, **11(Suppl 1):**CO-087

**Rationale:** There is no data on critically ill patients with primary central nervous system lymphoma (PCNSL) requiring intensive care unit (ICU) admission. We sought to describe the reasons for ICU admission and to evaluate the outcomes and prognostic factors of patients with PCNSL admitted to the ICU.

**Patients and methods/materials and methods:** Retrospective observational cohort study of consecutive PCNSL patients admitted to three ICUs over a two-decade period. Patients with previous lymphoma or concurrent lymphoma systemic involvement were excluded. Multivariate analysis was performed to predict 6-month mortality after ICU admission. Kaplan–Meier survival curves according to disease status and reason for ICU admission were computed for 6-month mortality. The study was approved by the French Intensive Care Society Institutional Review Board (CE SRLF 20–15)

**Results:** Acute respiratory failure, mainly secondary to aspiration pneumonia and *Pneumocystis jirovecii* pneumonia, was the leading reason for ICU admission (33%). The Glasgow Coma Scale score on admission was significantly lower in patients with aspiration pneumonia than in patients with other causes of ARF (7 [6–11] vs. 14 [13–15], *p* = 0.016). Aspiration pneumonia was more common in patients with brainstem tumor (67% vs. 0%, *p* < 0.001), whereas patients with intracranial hypertension were more frequently admitted for coma without seizures (61% vs. 9%, *p* = 0.004). Hospital and 6-month mortality rates were 47% and 53%, respectively. In multivariate analysis, admission for coma without seizures (OR 7.28), cancer progression (OR 3.47), mechanical ventilation (OR 6.58) and vasopressors (OR 4.07) were associated with higher 6-month mortality. Karnofsky performance status and partial or complete response prior to ICU admission were independently associated with lower 6-month mortality (Fig. 1).

**Conclusion:** Six-month survival of PCNSL patients admitted to the ICU appears to be relatively favorable (around 50%) and the presence of PCNSL alone is not a relevant criterion for ICU refusal. Predictive factors of mortality may help clinicians to make optimal triage decisions.

**Compliance with ethics regulations:** Yes in clinical research.
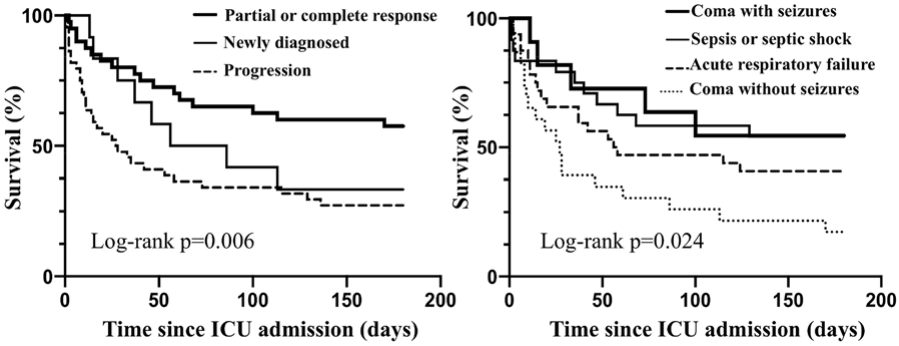


Kaplan–Meier survival curves (6-month mortality) according to the reason for intensive care unit (ICU) admission (right panel) and disease status (left panel)

### CO-088 CAR-T cells in the ICU: a 2-year experience

#### Sandrine Valade, Eric Mariotte, Virginie Lemiale, Nicolas Boissel, Catherine Thieblemont, Bertrand Arnulf, Asma Mabrouki, Lara Zafrani, Elie Azoulay, Michael Darmon

##### APHP Hôpital Saint Louis, Paris, France

**Correspondence:** Sandrine Valade - sandrine.valade@aphp.fr

*Annals of Intensive Care* 2021, **11(Suppl 1):**CO-088

**Rationale:** CAR-T cell (chimeric antigen receptor T) therapy is an emerging treatment in refractory haematological malignancies (especially acute lymphoid leukemia, ALL and diffuse large B cell lymphoma, DLBCL). Several CAR-T cells-related complications can lead the patients to the ICU. We aim to describe critically ill patients who received CAR-T cells and to assess risk factors of mortality.

**Patients and methods/materials and methods:** Single-center prospective study. Consecutive CAR-T cell recipients requiring ICU admission from July 2017 and December 2020 were included. Patients were classified into 4 groups: “sepsis” (documented infection), “CRS” (without documented or suspected infection), “sepsis or CRS” (suspected infection while fulfilling CRS criteria) and “disease progression”. Only the first ICU admission was considered for each patient.

**Results:** 71 patients (42 men, 59%), median age 60 years [37–68], were admitted in the ICU, 6 days [4–7] after CAR-T cells infusion. The underlying haematological malignancy was mostly DLBCL (*n* = 53, 75%), whereas 17 patients had ALL (24%) or multiple myeloma (*n* = 1, 1.45%). Median performance status (PS) was 1 [1–2]. Hemodynamic failure was the main reason for ICU admission (*n* = 40, 48%) and SOFA at day 1 was 4 [2–6]. Isolated CRS was the most common complication (*n* = 33, 46%), 21 patients (30%) had documented sepsis (catheter-related infection in 71% of the cases). Thirteen patients (18%) had suspected “sepsis or CRS” and the remaining 4 patients (5.6%) presented disease progression. Neurological toxicity was found in 26 (37%) and was systematically associated with features of CRS. At ICU admission, vasopressors were required in 18 patients (25%), antibiotics in 70 (98%), and invasive mechanical ventilation in two. Overall, 49 patients (69%) and 40 patients (56%) received tocilizumab and steroids, respectively. Hospital mortality was 11% (*n* = 8). The median follow-up was 6 months [2–15]. At the last follow-up, 25 patients had a complete response (35%) and 6 (8%) had a partial response; 26 patients (37%) were deceased and 11 (15%) experienced disease progression. Cox model identified reason for ICU admission (HR 4.02 disease progression vs. sepsis or CRS [95% CI 1.10–14.65]), PS (HR 1.97 per point [95% CI 1.14–3.41]) and SOFA score (HR 1.16 per point [95% CI 1.01–1.33]) associated with mortality (Fig. 1).

**Conclusion:** CAR-T cells therapy is a promising treatment in refractory haematological malignancies. Microbiologically documented infections are frequent and usually related to catheter infection. Performance status strongly influences the outcome, survival being constant in patients with very good performance status.

**Compliance with ethics regulations:** Yes in clinical research.
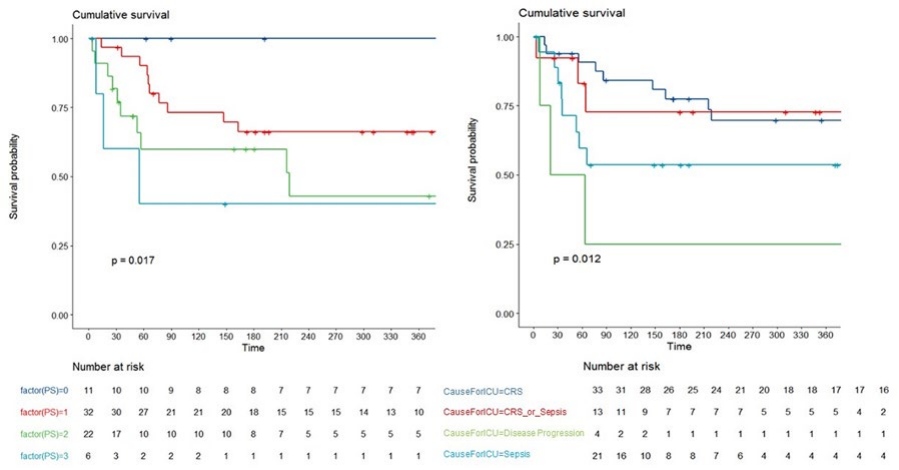


Fig. 1 Survival according to performance status (PS) and reason for ICU admission

### CO-089 Clinical significance of thrombocytopenia in patients with septic shock

#### Edwige Péju, Gaëlle Fouqué, Julien Charpentier, Jean-François Llitjos, Alain Cariou, Jean-Paul Mira, Mathieu Jozwiak, Matthieu Jamme, Frédéric Pène

##### Hôpital Cochin, Paris, France

**Correspondence:** Edwige Péju - edwigepeju@gmail.com

*Annals of Intensive Care* 2021, **11(Suppl 1):**CO-089

**Rationale:** Thrombocytopenia is a common albeit multifactorial disorder in critically ill patients and is known to be associated with poor prognosis. Whether thrombocytopenia accounts for a bystander of severity or may drive specific complications is unclear. With respect to the various immune and procoagulant functions of platelets, the aim of this study is to address the impact of thrombocytopenia on the development of ICU-acquired infections, bleeding and ischemic events in the high-risk patients with septic shock.

**Patients and methods/materials and methods:** This single-center retrospective study was conducted in the medical ICU over a 12-year period (2008–2019). Patients diagnosed for septic shock (Sepsis-2 definition) within the first 48 h of ICU admission were included. Patients who were discharged or died within the first 48 h were excluded to retain patients at risk of further ICU-acquired complications. Time course of platelet counts and transfusion of blood products including platelet concentrates were collected daily. Thrombocytopenia was defined as absolute (platelet count < 100 G/L) or relative (decrease in platelet count > 30% regardless of nadir count). Outcomes were in-ICU mortality and the development of ICU-acquired infections, severe bleeding events (WHO grade 3–4) and acute ischemic events.

**Results:** Among the 1310 patients admitted for septic shock, 1024 remained alive in the ICU after 48 h. Median age of 48-h survivors was 68 (57–77) years, and 39% of them were immunocompromised, including one-third of hematological malignancies. Upon admission, 284 patients (28%) had absolute thrombocytopenia. Over the first week in the ICU, 259 (25%) and 169 (17%) patients exhibited absolute and relative thrombocytopenia, respectively. ICU mortality rates of 48-h survivors with absolute or relative thrombocytopenia or without thrombocytopenia were 35%, 18% and 15%, respectively (*p* < 0.001). Patients with absolute or relative thrombocytopenia had increased frequencies of ICU-acquired infections (31% and 24%, vs. 16%, *p* < 0.001), severe bleeding (22% and 14%, vs. 4%, *p* < 0.001) and ischemic events (12% and 7%, vs. 5%, *p* = 0.009).

**Discussion:** Platelet disorders may plausibly contribute to the development of secondary complications in septic shock. The predictive value of thrombocytopenia will be investigated using multivariate time-dependent Cox cause-specific proportional hazard model.

**Conclusion:** Thrombocytopenia in critically ill patients with septic shock is associated with increased risks of ICU-acquired infections, bleeding and ischemic events and eventually with increased mortality.

**Compliance with ethics regulations:** Yes in clinical research.

### CO-090 Microvascular barrier alteration is associated with major bleeding in thrombocytopenic patients admitted in intensive care unit

#### Vincent Belossi, Geoffroy Hariri, Louis Perol, Jean-Rémi Lavillegrand, Tomas Urbina, Sandie Mazerand, Jean-Luc Baudel, Naïke Bigé, Bertrand Guidet, Eric Maury, Hafid Aït-Oufella

##### Hôpital Saint Antoine - GH Sorbonne Université, Paris, France

**Correspondence:** Geoffroy Hariri - geoffroyhariri@hotmail.com

*Annals of Intensive Care* 2021, **11(Suppl 1):**CO-090

**Rationale:** Thrombocytopenia, a frequent condition in intensive care unit (ICU) is associated with increased risk for bleeding. However, in critically ill patients with thrombocytopenia, risk factors for major bleeding remain unknown. We hypothesized that skin or mucosa purpura which reflects microvascular barrier alteration, may aggravate risk for major bleeding in critically ill patients with thrombocytopenia.

**Patients and methods/materials and methods:** During a 1-year period, we prospectively collected data from adult patients admitted with thrombocytopenia (< 150 G/L) in our department. In the absence of bleeding, platelet threshold for transfusion was 20 G/L in our ICU. Clinical and biological parameters were collected at admission. Microvascular barrier alteration was defined as the presence of purpura (on the skin, the buccal mucosa or the conjunctiva) or hematuria. Patients with bleeding at admission were excluded. During the ICU stay, we prospectively recorded major bleedings defined as bleeding requiring RBC transfusion or bleeding leading to life-threatening organ failure (Brain, lung…).

**Results:** Ninety-two patients were included during a 1-year period, median age was 61 [46–68] years and median SOFA was 6 [3–8]. Among them, 22 patients (24%) had major bleeding complication during their ICU stay. Median time between ICU admission and major bleeding was 8 [2–19] days. Demographic characteristics were not different between Bleeding and No-bleeding groups as well as the cause of thrombocytopenia. Median platelet count was not different between groups (57 [24–94] vs 90 [31–127] G/L; *p* = 0.16), as well as hemoglobin level and coagulation parameters. Patients in the bleeding group are characterized by higher urea level (13 [9–31] vs 9 [5–13] mmol/L; *p* < 0.01), higher creatinine level (148 [84:282] vs 91 [60;136] µmol/L; *p* = 0.06) and more frequent need of renal replacement therapy (29 vs 7%; *p* < 0.05). Clinical signs of microvascular barrier alteration are more frequent in the bleeding group (55 vs 27%; *p* = 0.01). Finally, thrombocytopenic patients with clinical signs of microvascular barrier alteration had a threefold higher risk of developing major bleeding complication during their ICU stay (HR: 3.4 [1.3–8.3]; *p* < 0.01) (Fig. 1)

**Conclusion:** In critically ill patients with thrombocytopenia, we identified several factors associated with major bleeding complications: increased urea and creatinine levels and clinical manifestations of microvascular barrier alteration. These parameters may be included in platelet transfusion strategy to limit major bleeding in thrombocytopenic patients.

**Compliance with ethics regulations:** Yes in clinical research.
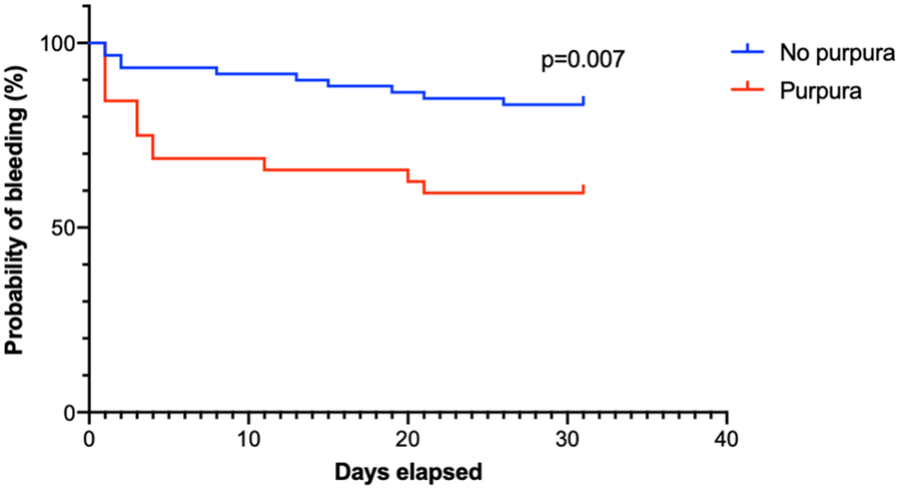


Survival curves of risk of major bleeding in ICU in patients with purpura (red) and without purpura (blue)

### CO-091 High-flow nasal cannula decreases respiratory effort and drive in patients with sepsis and septic shock

#### Bertrand Pavlovsky^2^, Tommaso Mauri^2^, Elena Spinelli^2^, Domenico Grieco^3^, Irene Ottaviani^4^, Maria Cristina Basile^2^, Francesca Dalla Corte^4^, Gabriele Pintaudi^3^, Eugenio Garofalo^5^, Annalisa Rundo^6^, Carlo Alberto Volta^4^, Antonio Pesenti^2^, Savino Spadaro^4^

##### ^1^IRCCS (Institute for Treatment and Research) Ca’ Granda Maggiore Policlinico Hospital Foundation, Milano, Italie; ^2^IRCCS (Institute for Treatment and Research) Ca’ Granda Maggiore Policlinico Hospital Foundation, Milano, Italie; ^3^Fondazione Policlinico Universitario A.Gemelli IRCCS (Institute for Treatment and Research), Roma, Italie; ^4^Sant’Anna University Hospital, Ferrara, Italie; ^5^Università Magna Greacia, Catanzaro, Italie; ^6^Polo ospedaliero Belcolle ASL, Viterbo, Italie

**Correspondence:** Bertrand Pavlovsky - bertrand.pavlovsky@gmail.com

*Annals of Intensive Care* 2021, **11(Suppl 1):**CO-091

**Rationale:** High-flow nasal cannula (HFNC) is a non-invasive respiratory support mainly indicated in hypoxemic patients. HFNC reduces respiratory effort in this population. Experimental data suggest an increase in respiratory drive in patients with sepsis and septic shock. We aimed to assess the effect of HFNC on respiratory drive and effort in this population.

**Patients and methods/materials and methods:** 25 non-intubated patients admitted to 3 intensive care units with a diagnosis of sepsis or septic shock were enrolled. Patients’ characteristics (age, BMI) and sepsis severity (SOFA score and lactate plasmatic concentration at enrollment) were assessed. Study protocol was composed of 3 consecutive steps of 30 min: (1) low-flow oxygen (LFO) device at baseline, (2) HFNC, (3) LFO post-HFNC. Gas exchange, esophageal pressure (Pes) and electrical impedance tomography (EIT) were recorded toward the end of each step. Respiratory effort was measured as the esophageal negative pressure swing (ΔPes). Respiratory drive was measured as the inspiratory esophageal pressure change during the first 500 ms from start of inspiration (P0.5). Finally, dynamic respiratory system compliance was calculated as the tidal volume (measured by EIT) divided by ΔPes (VT/ΔPes). To assess predictors of effort improvement associated with HFNC, correlations between ΔPes improvement between steps (1) and (2) and physiological variables at baseline were performed.

**Results:** Median age was 69 [interquartile range 54–79], BMI was 24.2 [21.6–27.1] kg m^−2^. At enrollment, 13 patients (52%) were in septic shock, with a SOFA score of 5 [4–9] and a lactate plasmatic concentration of 2.9 [2.4–4.7] mEq L^−1^. PaO2/FiO2 ratio did not improve significantly between the 3 steps (257 [228–331] vs.329 [280–367] vs.308 [246, 356], *p* = 0.160), nor PaCO2 (33.8 [30.9–41.3] vs. 33.9 [29.9–39.1] vs. 34.1 [32.1–41.2], *p* = 0.151). HFNC induced a significant decrease in ΔPes, P0.5 and a significant increase in VT/ΔPes (Fig. 1). Respiratory effort improvement with HFNC was correlated with ΔPes (r2 = 0.461, *p* < 0.001) and respiratory rate (r2 = 0.235, *p* = 0.030) at baseline, but not with PaO2 (r2 = 0.002, *p* = 0.836) nor PaCO2 (r2 = 0.026, *p* = 0.498).

**Conclusion:** HFNC decreases respiratory effort and drive at baseline, and improves respiratory mechanics in patients with sepsis and septic shock. These changes are not related to an improvement of PaO2/FiO2 or a decrease in PaCO2. Higher improvement of respiratory effort and drive occurs in patients with higher ΔPes and respiratory rate.

**Compliance with ethics regulations:** Yes in clinical research.
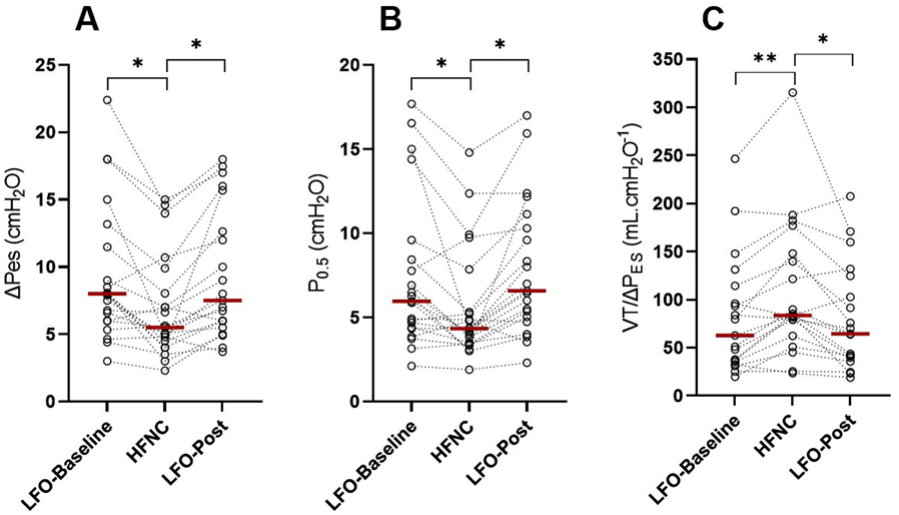


Fig. 1 Individual effect high-flow nasal cannula on negative esophageal pressure swing (ΔPes, **A**), ΔPes at 500 ms of the effort start (P0.5, **B**) and estimated dynamic compliance (VT/ΔPes, **C**). Median values are expressed in red bars. An ANOVA testing was *p*

### CO-092 Physiological effects of high-flow nasal cannula versus continuous positive airway pressure in acute hypoxemic respiratory failure

#### Samuel Tuffet^1^, François Perier^1^, Tommaso Maraffi^2^, Caio Morais^3^, Anne-Fleur Haudebourg^1^, Nicolas De Prost^1^, Keyvan Razazi^1^, Armand Mekontso-Dessap^1^, Guillaume Carteaux^1^

##### ^1^Hôpital Henri Mondor, Créteil Cédex, France; ^2^Centre Hospitalier Intercommunal de Créteil, Créteil, France; ^3^TIMPEL, Sao Paulo, Bresil

**Correspondence:** Samuel Tuffet - samuel.tuffet@aphp.fr

*Annals of Intensive Care* 2021, **11(Suppl 1):**CO-092

**Rationale:** The respective physiological effects of high-flow nasal cannula (HFNC) and continuous positive airway pressure (CPAP) in patients suffering from acute hypoxemic respiratory failure (AHRF) have been poorly assessed. Our aim was to compare the effects of HFNC and CPAP on tidal volume, end-expiratory lung volume (EELV) and ventilation distribution in patients with AHRF.

**Patients and methods/materials and methods:** Prospective single-center physiological study. Adult patients with AHRF were included after written informed consent. Chest electrical impedance tomography (EIT) (Enlight 1800, Timpel^®^) was recorded during three 10-min periods: (1) HFNC set at 50–60 L/min (HFNC1); (2) CPAP of 4 cm H2O; (3) resume HFNC (HFNC2). Flow signal was also recorded during CPAP, allowing to measure the expired tidal volume. Tidal volume under HFNC was computed by multiplying tidal impedance variation during HFNC by the ratio between tidal volume and tidal impedance variation under CPAP. Tidal volume, EELV, and ventilation distribution were compared between each period.

**Results:** In these preliminary results, we report the first 15 included patients. Ten men and five women, aged 63 (57–69), were included. Etiology for AHRF was pneumonia in 13 (87%) patients. HFNC had been introduced since 12 (10–16) h before inclusion, with a median FiO2 of 70% (50–100). Tidal volume during CPAP was significantly higher than during both HFNC1 and HNFC2 periods: 602 (553–647) mL (9.3 [8.5–10.7] mL/kg predicted body weight [PBW]), 443 (328–484) mL (6.5 [5.5–7.4] mL/kg PBW) and 440 (320–461) mL (6.5 [5–7.8] mL/kg IBW), respectively (*p* < 0.01 for each comparison between CPAP and HFNC, Fig.). No correlation was found between tidal volume recorded during CPAP and HFNC1 period (Pearson correlation coefficient = 0.49; *p* = 0.06). EELV increased by 173 (110–421) mL when CPAP was applied (Fig. 1). Ventilation distribution did not significantly differ between periods and was slightly predominant in posterior areas (median anterior distribution: 44% for HFNC1, 42% for CPAP and 42% for HFNC2; *p* = 0.28).

**Conclusion:** As compared to HFNC set at 50–60 L/min, a CPAP level of 4 cmH2O resulted in significantly higher tidal volume and EELV, and comparable ventilation distribution.

**Compliance with ethics regulations:** Yes in clinical research.
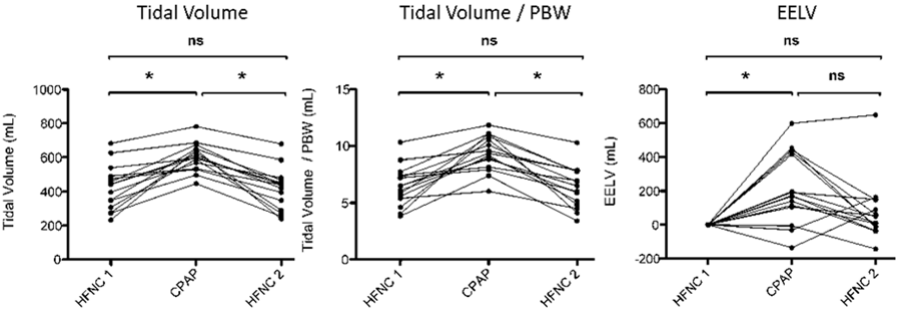


Evolution of tidal volume (left), tidal volume/PBW (middle) and EELV (right). EELV during HFNC 1 is the reference and plotted at 0.* denotes *p* < 0.05

### CO-093 Escalation of oxygenation therapy and mortality in critically ill immunocompromised patient with acute hypoxemic respiratory failure: a clustering analysis

#### Elise Yvin^1^, Michael Darmon^1^, Achille Kouatchet^2^, Fabio Silvio Taccone^3^, Omar Ben Hadj Salem^4^, Philippe Bauer^5^, Amélie Seguin^6^, Andry Van De Louw^7^, Victoria Metaxa^8^, Kada Klouche^9^, Ignacio Martin Loeches^10^, Luca Montini^11^, Sangeeta Mehta^12^, Fabrice Bruneel^13^, Thiago Lisboa^14^, William Nascimento^15^, Peter Pickkers^16^, Lene Russell^17^, Katerina Rusinova^18^, Jordi Rello^21^, Francois Barbier^19,22^, Djamel Mokart^20^, Elie Azoulay^1^

##### ^1^APHP - Hopital Saint Louis, Paris, France; ^2^Service de médecine intensive et réanimation, Angers, France; ^3^Médecine intensive et réanimation, Bruxelles, Belgique; ^4^Service de médecine intensive réanimation, Hôpital Cochin, Paris, France; ^5^Service de réanimation, Mayo Clinic, Rochester, Etats-Unis; ^6^Service de réanimation, Nantes, France; ^7^Service de réanimation, Penn state University College of Medicine, Hershey, Etats-Unis; ^8^Service de réanimation, King’s college Hospital, Londres, Royaume-Uni; ^9^Service de réanimation, Montpellier, France; ^10^Service de réanimation, Trinity College, St James Hospital, Dublin, Irlande; ^11^Hopital universitaire Agostino Gemelli, Rome, Italie; ^12^Service de réanimation, Sinai Health System, Toronto, Canada; ^13^Service de réanimation, Hôpital André Mignot, Versailles, France; ^14^Service de réanimation, Hopital Santa Rita, Rio De Janeiro, Bresil; ^15^Service de réanimation, Hopital Copa d’Or, Rio De Janeiro, Bresil; ^16^Département de réanimation, Nijmegen, Pays-Bas; ^17^Service de réanimation, Hopital universitaire, Copenhague, Pays-Bas; ^18^Service d’anesthésie et réanimation, Prague, Republique Tcheque; ^19^Service d’anesthésiologie, CHU Nimes, Nimes, France; ^20^Réanimation polyvalente et département d’anesthésie réanimation, Institut Paoli Calmettes, Marseille, France; ^21^Centro de Investigación Biomédica en Red en enfermedades respiratorias, Barcelone, Espagne; ^22^Médecine intensive et réanimation, Orléans, France

**Correspondence:** Elise Yvin - yvin.elise@gmail.com

*Annals of Intensive Care* 2021, **11(Suppl 1):**CO-093

**Rationale:** Acute respiratory failure (ARF) in immunocompromised patients remains the leading cause of admission in Intensive Care Unit (ICU), with high mortality. Among strategies that were believed to influence outcome of critically ill immunocompromised patients, choice of the initial oxygenation modality remains debated. Despite promising results in physiological studies, a recent RCT failed to demonstrate any benefit of high-flow nasal oxygen (HFNP) in this setting (1). The study aims to assess evolutionary profiles of oxygenation strategy and their influence on outcome.

**Patients and methods/materials and methods:** This study is a post hoc analysis of a multicenter multinational dataset (2) using Group-Based Trajectory Modeling, a non-parametric longitudinal clustering technique derived from *k*-mean.

**Results:** A total of 1547 patients were included. Rate of mechanical ventilation (MV) at day 1 was 24.3% (*n* = 376) while, respectively, 50.3% (*n* = 778), 11.4% (*n* = 177) and 14% (*n* = 216) of patients received standard oxygen therapy, high-flow nasal oxygen and non-invasive mechanical ventilation. Three clusters of oxygenation profiles were identified. Cluster A (limited need for MV and a good outcome) included 717 patients (46.3%). Cluster B (unfavorable outcome and 32.9% need for MV) consisted of 499 patients (32.3%). Cluster C (low mortality and 37.5% need for MV) included 331 patients (21.4%). After adjustment for confounders, Cluster B and C were independently associated with mortality (respectively, OR 142 [73.4–313] and OR 0.465 [0.295–0.718]) and need for MV (OR 9.87; 95% CI [7.26–13.5] and OR 19.8; 95% CI [13.7–29.1]). Exploratory analysis confirmed that variables at ICU admission (namely respiratory rate for Cluster B and number of abnormal chest X-ray quadrant for Cluster C) may predict oxygenation strategy evolutionary clusters.

**Conclusion:** This study identified three distinct highly performing clusters of response to initial oxygenation strategy, reliably predicting mechanical ventilation requirement and mortality. Studies to assess clinical outcomes from implementing a cluster-based ICU organization are warranted.

**References**

1. Azoulay E, Lemiale V, Mokart D, Nseir S, Argaud L, Pène F, et al. Effect of High-Flow Nasal Oxygen vs Standard Oxygen on 28-Day Mortality in Immunocompromised Patients With Acute Respiratory Failure. JAMA. 2018;320(20):2099–107.
2. Azoulay E, Pickkers P, Soares M, Perner A, Rello J, Bauer PR, et al. Acute hypoxemic respiratory failure in immunocompromised patients: the Efraim multinational prospective cohort study. Intensive Care Med. 2017;43(12):1808–19.

**Compliance with ethics regulations:** Yes in clinical research.
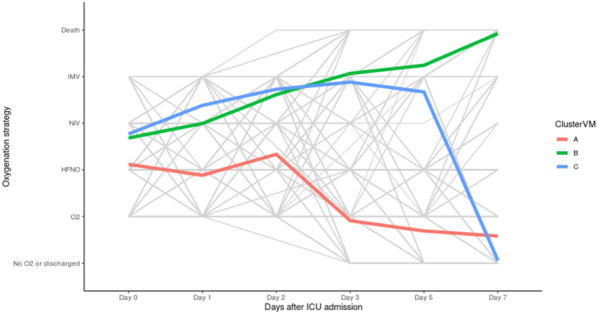


Cluster identification according to oxygenation modalities

### CO-094 High-flow nasal oxygen therapy to avoid invasive mechanical ventilation in SARS-CoV-2 pneumonia: a retrospective study

#### Nicolas Bonnet^1^, Olivier Martin^1^, Marouane Boubaya^1^, Vincent Levy^1^, Nathan Ebstein^1^, Philippe Karoubi^2^, Yacine Tandjaoui-Lambiotte^1^, GuillaumeVan Der Meersch^1^, Johanna Oziel^1^, Marie Soulié^1^, Mohamed Ghalayini^1^, Anais Winchenne^1^, Jean Ralph Zahar^1^, Passem Ahmed^2^, Stephane Gaudry^1^, Yves Cohen^1^

##### ^1^CHU Avicenne, Bobigny, France; ^2^CH Rambouillet, Rambouillet, France

**Correspondence:** Nicolas Bonnet - nicolas.bonnet@aphp.fr

*Annals of Intensive Care* 2021, **11(Suppl 1):**CO-094

**Rationale:** The efficacy of high-flow nasal canula oxygen therapy (HFNO) to prevent invasive mechanical ventilation (IMV) is not well established in severe coronavirus disease 2019 (COVID-19). The aim of this study was to compare the risk of IMV between two strategies of oxygenation (conventional oxygenation and HFNO) in critically ill COVID 19 patients

**Patients and methods/materials and methods:** This was a bicenter retrospective study which took place in two intensive care units (ICU) of tertiary hospitals in the Paris region from March 11, to May 3, 2020. We enrolled consecutive patients hospitalized for COVID-19 and acute respiratory failure (ARF) who did not receive IMV at ICU admission. The primary outcome was the rate of IMV after ICU admission. Secondary outcomes were death at day 28 and day 60, length of ICU stay and ventilator-free days at day 28. Data from the HFNO group were compared with those from the standard oxygen therapy (SOT) group using weighted propensity score.

**Results:** Among 138 patients who met the inclusion criteria, 62 (45%) were treated with SOT alone, and 76 (55%) with HFNO. In HFNO group, 39/76 (51%) patients received IMV and 46/62 (74%) in SOT group (OR 0.37 [95% CI, 0.18–0.76] *p* = 0.007). After weighted propensity score, HFNO was still associated with a lower rate of IMV (OR 0.31 [95% CI, 0.14–0.66] *p* = 0.002). Length of ICU stay and mortality at day 28 and day 60 did not significantly differ between HFNO and SOT groups after weighted propensity score. Ventilator-free days at days 28 was higher in HNFO group (21 days vs 10 days, *p* = 0.005). In the HFNO group, predictive factors associated with IMV were SAPS2 score (OR 1.13 [95%CI, 1.06–1.20] *p* = 0.0002) and ROX index > 4.88 (OR 0.23 [95% CI, 0.008–0.64] *p* = 0.006).

**Conclusion:** High-flow nasal canula oxygen for ARF due to COVID-19 is associated with a lower rate of invasive mechanical ventilation

**Compliance with ethics regulations:** Yes in clinical research.

### CO-095 Evaluation of the mortality of COVID-19 patients treated with non-invasive ventilatory support as ceiling treatment compared to patients eligible for invasive mechanical ventilation

#### Clement Medrinal^1^, Alexis Gillet^2^, Fairuz Boujibar^4^, Jonathan Dugernier^3^, Marcel Zwahlen^7^, Bouchra Lamia^1^, Christophe Girault^4^, Jacques Créteur^2^, Jean-Marc Fellrath^3^, Laurence Haesler^3^, Laurie Lagache^1^, Laure Goubert^1^, Elise Artaud Macari^4^, Olivier Taton^2^, Dimitri Leduc^2^, Olivier Van Hove^2^, Michèle Norrenberg^2^, Guillaume Prieur^1^, Yann Combret^1^, Nils Correvon^3^, Roger Hilfiker^5^, Olivier Contal^6^

##### ^1^Groupe Hospitalier du Havre, Le Havre, France; ^2^Erasme University Hospital, Brussels, Belgium, Brussels, Belgique; ^3^Réseau Hospitalier Neuchâtelois, Neuchâtel, Suisse; ^4^CHU Rouen, Rouen, France; ^5^School of Health Sciences, University of Applied Sciences and Arts Western Switzerland Valais (HES-SO Valais-Wallis), Leukerbad, Suisse; ^6^University of Applied Sciences and Arts, Western Switzerland (HES-SO), Lausanne, Suisse; ^7^Institute of Social & Preventive Medicine, University of Bern, Bern, Suisse

**Correspondence:** Clement Medrinal - medrinal.clement.mk@gmail.com

*Annals of Intensive Care* 2021, **11(Suppl 1):**CO-095

**Rationale:** Early use of mechanical ventilation is the gold standard approach for COVID-19 patients with hypoxemic acute respiratory distress. Massive influx of patients did not allow a maximalist approach for all patients. Due to their co-morbidities, advanced age or lack of space in intensive care units, many patients received a “Do not intubate” order (DNI). Few data were available concerning management of these patients. The objective of this study was to compare the mortality of patients with severe Covid-19 managed with high-flow nasal cannula (HFNC) and CPAP as ceiling therapy and patients scheduled to receive invasive mechanical ventilation if previous support failure.

**Patients and methods/materials and methods:** Data from 4 centers across 3 European countries were collected. All consecutive patients with a severe COVID-19 with high oxygeno-dependance (HFNC or PaO2/FiO2 < 300 or SpO2 < 94% with O2 flow 10L/min) were included. We emulated a hypothetical target trial where patients with an only non-invasive ventilation strategy were compared to patients who were treated with a strategy including intubation if necessary. We set up a propensity score for the probability of belonging to the “DNI” and an inverse probability of treatment weighting to remove the confounding by indication. A logistic regression model was fitted with the variables that informed to decision to classify patients in the “able to intubate” or “DNI” group the variables that are potentially associated with mortality.

**Results:** 400 patients were included in our analysis. Briefly, 270 (67.5%) were classified as eligible to intubation with invasive mechanical ventilation and 130 (32.5%) were classified as “DNI”. In the unadjusted analyses, 72 (27%) patients eligible to intubation died compared to 77 (59%). The adjusted risk ratio to die for patients eligible to intubation was 0.803 (95% CI 0.444 to 1.453) compared to the do not intubate patients. The effect sizes for the differences between the two groups in the important prognostic factors were strongly reduced by inverse probability of treatment weighting. However, there was still a small difference for age. The median length of stay in ICU was similar between the groups for survivors (18 (10–31) vs. (19 (13–23.5; *p* = 0.76).

**Conclusion:** For patients with DNI, a complete ventilatory strategy using HFNC as first line and CPAP therapy in case of degradation do not show a significant difference in the adjusted mortality rate compared to invasive mechanical ventilation.

**Compliance with ethics regulations:** Yes in clinical research.

### CO-096 Prediction of outcome of nasal high-flow use during COVID-19-related acute hypoxemic respiratory failure

#### Noémie Zucman^1^, Jimmy Mullaert^2^, Damien Roux^1^, Oriol Roca^3^, Jean-Damien Ricard^1^

##### ^1^Hôpital Louis Mourier, APHP, Colombes, France; ^2^Hôpital Bichat, APHP, Paris, France; ^3^Vall d´Hebron University Hospital, Barcelone, Espagne

**Correspondence:** Noémie Zucman - noemie.zucman@gmail.com

*Annals of Intensive Care* 2021, **11(Suppl 1):**CO-096

**Rationale:** In the course of COVID-19-related acute hypoxemic respiratory failure (AHRF), nasal high-flow (NHF) has been broadly used as first-line ventilatory support. We evaluated the ROX index (1), defined as the ratio of SpO2/FiO2 to respiratory rate (RR), as an early marker of NHF response and a potential predictor of its failure in the ICU setting.

**Patients and methods/materials and methods:** In this single-center retrospective study, all adults admitted to the ICU during the peak of the COVID-19 outbreak were screened for eligibility. Participants presenting with AHRF related to COVID-19 infection and treated with NHF as first-line ventilatory support were included. Main variables were collected from admission until NHF weaning or intubation which defined NHF failure. The ROX index was recorded several times daily. Statistical analysis included association between early response to NHF (i.e. the latest value of the ROX index within the first 4 h after NHF initiation (ROX-H0H4)) and risk for intubation (Cox’s model for patients still at risk at H4). Maximization of the Youden’s index led to an optimal cut-off of the ROX index to predict NHF outcome.

**Results:** Sixty-two patients were included. Median age was 55 (IQR 48–63). Participants presented with profound hypoxemia at NHF initiation (median FiO2 and SpO2 were 0.8 (IQR 0.6–1.0) and 96% (IQR 94–98), respectively) with median RR of 25 breaths per minute (IQR 21–32). Twenty-one patients (34%) succeeded on NHF and were discharged from ICU, whereas 39 (63%) required MV and 2 (3%) died while under NHF because of do-not-intubate order. Overall ICU mortality was 17%. Median time to intubation was 10 h (95% CI 7–57). Kaplan–Meier estimates of risk for intubation (*N* = 60) is illustrated in Fig. Median ROX-H0H4 was 5.4 (IQR 3.9–7.1). In Cox’s model, ROX-H0H4 ≥ 5.37 was significantly associated with a lower risk for intubation after H4 (HR 0.59, 95% CI 0.41–0.84; *P* = 0.0037) for patients still at risk (*N* = 45). ROX-H0H4 demonstrated a good discrimination (area under the ROC curve 0.75, 95% CI 0.60–0.90; sensitivity 0.66, specificity 0.83).

**Conclusion:** In COVID-19-related AHRF, use of NHF as first-line ventilatory support may have obviated the need for intubation in up to a third of cases. The ROX index could be used to identify patients who will fail on NHF, in order not to further delay intubation.

**Reference**

1. Roca O, Caralt B, Messika J, Samper M, Sztrymf B, Hernández G, et al. An Index Combining Respiratory Rate and Oxygenation to Predict Outcome of Nasal High-Flow Therapy. Am J Respir Crit Care Med. 2019;199(11):1368–76.

**Compliance with ethics regulations:** Yes in clinical research.
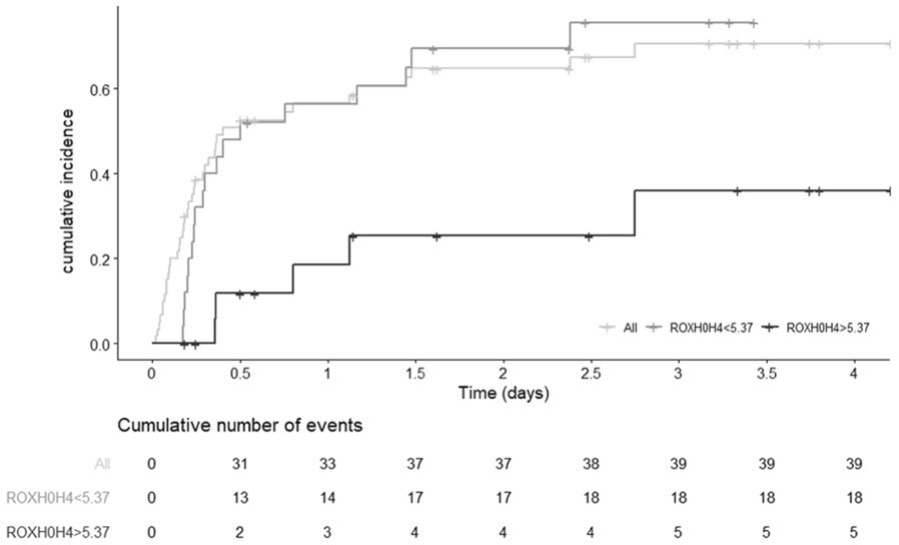


Cumulative incidence of orotracheal intubation for all at-risk patients (*N* = 60) and after 4 h of NHF (*N* = 45) stratified on ROX-H0H4 (landmark at H4). NHF, nasal high-flow. ROX-H0H4, latest value of the ROX index within the first 4 h after NHF initia

### CO-097 The composition of the gut microbiota differs between ESBL-producing E. coli and ESBL-K. pneumoniae faecal carriers

#### Renaud Prével, Raphaël Enaud, Arthur Orieux, Adrian Camino, Patrick Berger, Alexandre Boyer, Laurence Delhaes, Didier Gruson

##### CHU Bordeaux, Bordeaux, France

**Correspondence:** Renaud Prével - renaud.prevel@hotmail.fr

*Annals of Intensive Care* 2021, **11(Suppl 1):**CO-097

**Rationale:** Extended spectrum beta-lactamase (ESBL) producing Enterobacteriales are the main source of both antimicrobial resistance and empiric antimicrobial therapy failure in Europe. Microbiota is now suggested to be a key player in host resistance to colonization with multi-drug-resistant organisms. However, its role and the underlying mechanisms involved in the resistance to colonization with ESBL-producing Enterobacteriales (ESBL-E) remain unknown. The impact of the bacterial specie carrying the ESBL enzyme, *Escherichia coli* or *Klebsiella pneumoniae* being the most frequently isolated species on clinical samples, is also unknown. The aim of this work is thus to compare the gut microbiota composition between patients colonized with ESBL-producing *E. coli* and *K. pneumoniae*.

**Patients and methods/materials and methods:** The rectal swab performed for faecal ESBL-E carriage screening at admission of every consecutive patient admitted to ICU between January and March 2019 was collected and frozen at − 80 °C. DNA extraction was performed thanks to QIAamp^®^ PowerFecal^®^ Pro DNA kit (QIAgen^®^). V3–V4 regions of 16SRNA coding gene and ITS2 were amplified by PCR. Sequencing (2250 bp) was performed on MiSeq sequencer (Illumina^®^) and DADA2 pipeline on R software was used for bioinformatics analyses.

**Results:** Among the 255 patients admitted to ICU, 23 (9%) had a faecal carriage with an ESBL-E: 11/23 (48%) *E. coli*, 6/23 (26%) *K. pneumoniae* and 6/25 (8%) *Enterobacter aerogenes*, *Citrobacter freundii* or *Serratia marcescens* (2 patients for each). Only patients (*n* = 17) colonized with ESBL-producing *E. coli* and *K. pneumoniae* were further compared for statistical issues. Patients colonized with ESBL-producing *K. pneumonia* had higher SAPS2 score (80, IQR: 68–96 vs 55, IQR: 31–68, *p* = 0.007) and a higher rate of acute kidney injury (6/6 (100%) vs 2/11 (18%), *p* = 0.002) but similar age, sex ratio, haemodynamic or respiratory failure rates at admission. Gut microbiota α-diversity was lower for patients colonized with ESBL-producing *K. pneumoniae* than ESBL-producing *E. coli* both regarding Shannon and Simpson index (*p* = 0.02 and *p* = 0.007, respectively), but β-diversity was not different (*p* = 0.44). We did not find any difference in gut mycobiota α- or β-diversity between patients colonized with ESBL-producing *K. pneumoniae* and ESBL-producing *E. coli*.

**Conclusion:** The composition of the gut microbiota differs between ESBL-producing *E. coli* and ESBL-producing *K. pneumoniae* faecal carriers. These data suggest that bacterial specie, in addition to the mechanism of resistance, should probably be taken into account when investigating the role of gut microbiota in resistance to gut colonization with ESBL-E.

**Compliance with ethics regulations:** Yes in clinical research.

### CO-098 Microvascular hyperreactivity in cirrhotic patients with septic shock

#### Geoffroy Hariri, Tomas Urbina, Jean-Rémi Lavillegrand, Maxime Gasperment, Sandie Mazerand, Abdelkrim Abdelmalek, Naïke Bigé, Jean-Luc Baudel, Bertrand Guidet, Eric Maury, Hafid Ait-Oufella

##### Hôpital Saint Antoine - GH Sorbonne Université, Paris, France

**Correspondence:** Geoffroy Hariri - geoffroyhariri@hotmail.com

*Annals of Intensive Care* 2021, **11(Suppl 1):**CO-098

**Rationale:** Cirrhosis is a chronic liver disease responsible for high mortality and morbidity worldwide. Liver cirrhosis induces a large range of hemodynamic disorders including peripheral vasodilatation, decreased blood pressure and increased cardiac output. Physiological studies using forearm vessel occlusion plethysmography on medium-sized arteries have reported increased vascular reactivity in cirrhotic patients when compared to non-cirrhotic patients, vascular hyperactivity being more pronounced at the advanced stages of the disease. However, microvascular reactivity of cirrhotic patients in the context of sepsis has poorly been investigated. In this study, we aimed to compare skin microvascular function in cirrhotic and non-cirrhotic patients in the context of septic shock.

**Patients and methods/materials and methods:** During an 18-month period, we prospectively included adult patients admitted in our ICU for septic shock with and without cirrhosis. After initial resuscitation, global hemodynamic parameters were recorded and skin microvascular reactivity to local acetylcholine iontophoresis was measured. Acetylcholine acts as an endothelium-dependent vasodilator which induces the production of nitric oxide (NO) after stimulation of the endothelial NO-Synthase.

**Results:** Thirty patients with septic shock were included (60% male), 10 with cirrhosis and 20 without, with a median age of 61 [54–74] years. Cirrhotic patients were mainly classed Child–Pugh C (80%) and all of them had ascites. SOFA score and ICU mortality of cirrhotic patients was higher compared to non-cirrhotic patients, respectively (6.5 [5.0–8.3] vs 11.5 [9.0–14.0]; *p* < 0.01; 15 vs 70%; *p* < 0.01). Peripheral tissue perfusion and global hemodynamic parameters were not different between cirrhotic and non-cirrhotic patients, but arterial lactate level was threefold higher in patients with cirrhosis (*p* < 0.01). Basal skin microvascular blood flow was not statistically different between groups (4.94 [3.45–8.73] vs 6.95 [5.24–8.38] PU; *p* = 0.29). After acetylcholine simulation, skin microvascular blood flow increased more in cirrhotic patients than in non-cirrhotic patients (644 [217–966] vs 169 [73–505]%, *p* = 0.03). Global microvascular reactivity was sevenfold higher in cirrhotic patients (AUC: 16412 [13898–19041] vs 2664 [969–4604] PU; *p* < 0.001) (Fig. 1).

**Conclusion:** Our study investigated skin microvascular reactivity in cirrhotic patients in the context of septic shock. We found that baseline microvascular blood flow was not different between groups, but skin microvascular reactivity was strongly increased in cirrhotic patients when compared to non-cirrhotic patients. This exaggerated skin microvascular reactivity in cirrhotic patients may be related to NO endothelial overproduction.

**Compliance with ethics regulations:** Yes in clinical research.
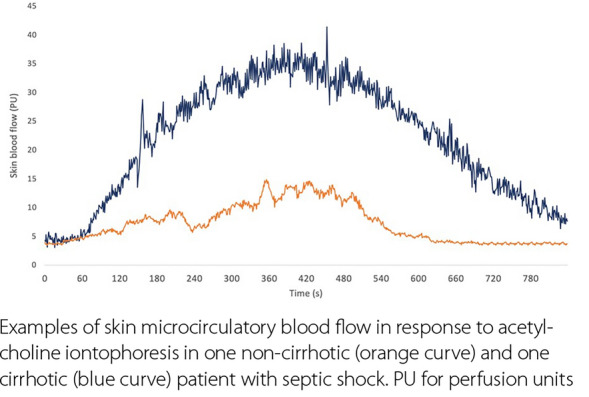


Examples of skin microcirculatory blood flow in response to acetylcholine iontophoresis in one non-cirrhotic (orange curve) and one cirrhotic (blue curve) patient with septic shock. PU for perfusion units

### CO-099 Standard versus high dose of tigecycline: efficiency and safety in nosocomial pneumonia

#### Wala Lazrag, Oussama Jaoued, Hajer Nouira, Wael Chamli, Rim Gharbi, Habiba Ben Sik Ali, Mohamed Fekih Hassen, Souheil Atrous

##### service de réanimation médicale hôpital Taher Sfar, Mahdia, Tunisie

**Correspondence:** Oussama Jaoued - oussamajaoued@gmail.com

*Annals of Intensive Care* 2021, **11(Suppl 1):**CO-099

**Rationale:** Nosocomial pneumonia is one of the leading causes of healthcare consumption and mortality in critically ill patients. The prevalence of multidrug resistance infection is increasing in intensive care unit. Classic antimicrobial agents used to treat these infections are becoming ineffective. Tigecycline has a bacteriostatic activity against multidrug-resistant germs. It is a glycylcycline antibiotic with a broad-spectrum activity against nearly all Gram-positive and Gram-negative germs. It is indicated for treatment of skin infection, complicated intra-abdominal infections and community-acquired bacterial pneumonia. We aimed to compare the efficiency of high dose and standard dose of tigecycline in the outcomes of patients with nosocomial pneumonia

**Patients and methods/materials and methods:** We conducted a retrospective study between 2018 and 2020 in the intensive care of a teaching hospital. Patients with nosocomial pneumonia and treated with tigecycline at standard dose (100 mg per day) or at a high dose (200 mg per day) were included in the analysis. Biological and clinical outcomes were investigated. The primary outcome was mortality. The secondary outcomes were clinical response rate, duration of mechanical ventilation, length of stay and adverse events.

**Results:** A total of 73 patients were included. *Acinetobacter baumannii* (64%), *Klebsiella pneumonia* carbapenem-resistant (22%) and *Escherichia coli* (3%) were the main isolated pathogens. Thirty-one patients received standard dose. The duration of tigecycline treatment was 8 ± 3 days in standard-dose group and 9 ± 4 days in the high-dose group (*p* = 0.08). The evolution of C-reactive protein and white blood cells was similar in both groups. The standard-dose group had a clinical cure rate of 61% versus 50% in the other group (*p* = 0.238). Superinfection rate was 19% in the standard-dose group versus 18% in high-dose group (*p* = 0.98). The length of stay was significantly higher in standard-dose group (34 ± 29 days versus 24 ± 12 days *p* = 0.02). Although not statistically significant, mortality was higher in the standard-dose group than the high-dose one (77% vs 55%, *p* = 0.08). ln the standard-dose group, four patients were dead due to another episode of infection. Side effects were similar in both groups.

**Conclusion:** When treating patients with nosocomial pneumonia, high dose of tigecycline had similar biological and clinical efficiency.

**Compliance with ethics regulations:** Yes in clinical research.

### CO-100 Adverse events in critically ills patients treated by plasma exchange therapy in intensive care unit: a 5-year retrospective multicenter cohort study

#### Mickaël François^1^, Antoine Gaillet^3^, Judith Provoost^4^, Rémi Trusson^2^, Romain Arrestier^3^, Laura Platon^1^, Racim Benomar^1^, Philippe Corne^1^, Olivier Hequet^5^, Jean-Christophe Richard^4^, Olivier Moranne^2^, Boris Jung^1^, Delphine Daubin^1^, Romaric Larcher^1^, Kada Klouche^1^

##### ^1^CHU Lapeyronie, Montpellier, France; ^2^CHU Carémeau, Nîmes, France; ^3^Hôpital Henri Mondor APHP, Créteil, France; ^4^Hôpital de la Croix Rousse HCL, Lyon, France; ^5^Centre Hospitalier Lyon sud Pavillon Marcel Berard HCL, Lyon, France

**Correspondence:** Delphine Daubin - d-daubin@chu-montpellier.fr

*Annals of Intensive Care* 2021, **11(Suppl 1):**CO-100

**Rationale:** Plasma exchange therapy (PET) is used in a variety of severe conditions including autoimmune diseases. The aim of the present study was to assess the incidence of PET-related adverse events in patients treated in the intensive care unit (ICU).

**Patients and methods/materials and methods:** All data of adults admitted to ICU of 4 French university hospitals between January 2015 and December 2019 who received PET (at least one session) were retrospectively collected from medical and procedure records. Groups with and without PET-related complications were compared using univariate and multivariate analyses.

**Results:** During the study period, 711 PET sessions were performed on 124 patients. Most common diseases treated by PET were: thrombotic thrombocytopenic purpura (*n* = 32, 26%), myasthenia gravis (*n* = 25, 20%), acute polyradiculoneuropathy (*n* = 12, 10%), ANCA-associated vasculitis (*n* = 8, 6%), catastrophic antiphospholipid syndrome (*n* = 9, 7%), acute disseminated encephalomyelitis (*n* = 4, 3%), humoral graft rejection (*n* = 4, 3%), and other (*n* = 25, 20%). At least one infectious complication occurred in 61/124 patients (49%) which were: ventilator-associated pneumonia (VAP): 42, septic shock: 19, viral reactivations: 14, pneumonia: 13, bacteremia: 12. Other reported adverse events included catheter-related infections: 6, bleeding complications: 18 (15%), and acute pulmonary oedema: 16 (13%). By multivariate analysis, PET-related infectious adverse events were significantly associated with the length of stay in the ICU (24 days versus 7 days (OR: 1.09 [1.04–1.15], *p* < 0.001)) and the requirement of an invasive mechanical ventilation (92% versus 35% (OR: 15.12 [4.70–62.29], *p* < 0.001)).

**Conclusion:** Infectious complications, including VAP were the most frequent PET-related adverse events observed in this cohort of critically ill patients. The incidence reported herein did not differ from that of ICU-acquired infections as well as associated factors. PET may not be considered as an additional infectious risk in critically ills patients.

**Compliance with ethics regulations:** Yes in clinical research

### CO-101 Relationship between obesity and ventilator-associated pneumonia: a post hoc analysis of the NUTRIREA2 trial

#### Olivier Pouly^1,4^, Saad Nseir^1^, Amélie Le Gouge^2^, Jean Reignier^3^

##### ^1^CHU LILLE, Lille, France; ^2^CHU TOURS, Tours, France; ^3^CHU NANTES, Nantes, France; ^4^HOPITAL SAINT PHILIBERT, Lomme, France

**Correspondence:** Olivier Pouly - olivier.pouly@hotmail.fr

*Annals of Intensive Care* 2021, **11(Suppl 1):**CO-101

**Rationale:** Patients with obesity are at higher risk for community-acquired and nosocomial infections. However, no study has specifically evaluated the relationship between obesity and ventilator-associated pneumonia (VAP).

**Patients and methods/materials and methods:** Post hoc analysis of the NUTRIREA-2 open-label RCT, performed in 44 French ICUs. Adults receiving invasive mechanical ventilation and vasopressor support for shock, and parenteral nutrition or enteral nutrition were included. Obesity was defined as body mass index (BMI) ≥ 30 kg/m^2^ at ICU admission. VAP diagnosis was adjudicated by an independent blinded committee, based on all available clinical, radiological, and microbiological data. Only first VAP episodes were taken into account. Incidence of VAP was analyzed using Fine and Gray model, with extubation, and death as competing risks.

**Results:** 699 (30%) of the 2325 included patients had obesity. 224 first VAP episodes were diagnosed (60, and 164 in obese, and non-obese groups, respectively). The incidence of VAP at day 28 was 8.6% vs 10.1% in the two groups (HR: 0.85 (95% CI 0.63–1.14), *p* = 0.26). After adjustment on gender, McCabe score, age, anti-ulcer treatment, and SOFA at randomization, the incidence of VAP remained non-significant between obese and non-obese patients (HR 0.893 (95% CI 0.66–1.2), *p* = 0.46). Although no significant difference was found in duration of mechanical ventilation, and ICU length of stay; 90-day mortality was significantly lower in obese than in non-obese patients (272 of 692 (39.3%) patients vs 718 of 1605 (44.7%), *p* = 0.02). In a sub-group of patients (*n* = 123) with available pepsin, and alpha-amylase measurements, no significant difference was found in rate of abundant microaspiration of gastric contents, or oropharyngeal secretions between obese and non-obese patients.

**Conclusion:** Our results suggest that obesity has no significant impact on the incidence of VAP. On behalf of the NUTRIREA-2 study group.

**Reference**

1. Reignier J et al. Enteral versus parenteral early nutrition in ventilated adults with shock: a randomised, controlled, multicentre, open-label, parallel-group study (NUTRIREA-2). Lancet 2017;391:133–43.

**Compliance with ethics regulations:** Yes in clinical research.

### CO-102 Gut bacteriobiota and mycobiota can both predict day 28 mortality among critically ill patients

#### Renaud Prével, Raphaël Enaud, Arthur Orieux, Adrian Camino, Patrick Berger, Alexandre Boyer, Laurence Delhaes, Didier Gruson

##### CHU Bordeaux, Bordeaux, France

**Correspondence:** Renaud Prével - renaud.prevel@hotmail.fr

*Annals of Intensive Care* 2021, **11(Suppl 1):**CO-102

**Rationale:** The role of gut microbiota (including both the bacterial kingdom: bacteriobiota and the fungal one: mycobiota) in human health and diseases has been investigated in the last decade thanks to the development of next-generation sequencing. Evidence is increasing that it can be a key player in numerous chronic diseases. Nevertheless, its impact on the prognosis of critically ill patients has been poorly investigated so far. The aim of this study is to assess the predictive value of gut microbiota (both bacteriobiota and mycobiota) for day 28 mortality in critically ill patients.

**Patients and methods/materials and methods:** The rectal swab performed for ESBL-E carriage screening at admission of every patient admitted to ICU in March 2019 was collected and frozen at − 80 °C. DNA extraction was performed thanks to QIAamp^®^ PowerFecal^®^ Pro DNA kit (QIAgen^®^). V3–V4 regions of 16SRNA coding gene and ITS2 were amplified by PCR. Sequencing (2*250 bp) was performed on MiSeq sequencer (Illumina^®^). DADA2 pipeline on R software was used for bioinformatics analyses. Predictive value for Day28 mortality was assessed by determining the area under the curve (AUC) of receiver operating characteristic (ROC) curves and by positive and negative predictive values (respectively, PPV and NPV).

**Results:** Fifty-seven patients were consecutively admitted to ICU of whom 13 (23%) deceased and 44/57 (77%) survived. Bacteriobiota α-diversity was significantly lower among non-survivors than survivors when expressed by the mean of Shannon index or Simpson index (respectively, *p* = 0.0008 and *p* = 0.001) as was mycobiota α-diversity (respectively, *p* = 0.03 and *p* = 0.03). AUC to predict day-28 mortality, regarding bacteriobiota, were 0.80, 95CI [0.65; 0.94] for Shannon index with PPV: 46%, PNV: 94% and 0.79, 95CI [0.66; 0.92] for Simpson index with PPV: 57%, NPV: 90% and regarding mycobiota, 0.70, 95CI [0.52; 0.87] for Shannon index with PPV: 39%, NPV: 90%, and 0.70, 95CI [0.53; 0.87] for Simpson index with PPV: 39%, NPV: 88%. Bacteriobiota β-diversity analysis also demonstrated significant differences between survivors and non-survivors (*p* = 0.05) but not mycobiota β-diversity (*p* = 0.57). Non-survivors had a higher abundance of *Staphylococcus haemolyticus*, *Clostridiales* sp., *Campylobacter ureolyticus* and *Akkermansia* sp. whereas survivors had a higher abundance of *Blautia* sp., *Streptococcus* sp., *Faecalibacterium prausnitzii* and *Bifidobacterium* sp. Non-survivors had a higher abundance of *Malassezia sympodialis*, *Malassezia dermatis* and *Saccharomyces cerevisiae*.

**Conclusion:** The gut bacteriobiota and mycobiota compositions of critically ill patients can predict day-28 mortality with interesting negative predictive values. The impact of gut microbiota modulation in ICU should be investigated.

**Compliance with ethics regulations:** Yes in clinical research.

### CO-103 Paracetamol use and lowered risk of acute kidney injury in patients with rhabdomyolysis

#### Maxime Desgrouas, Thierry Boulain

##### Centre Hospitalier Régional d’Orléans, Orléans, France

**Correspondence:** Maxime Desgrouas - maxime.desgrouas@orange.fr

*Annals of Intensive Care* 2021, **11(Suppl 1):**CO-103

**Rationale:** Mortality with rhabdomyolysis-associated acute kidney injury can be up to 80%. Experimental data from mouse models of rhabdomyolysis showed that paracetamol reduces the expected increase in serum creatinine level [1]. We aimed to assess the association between paracetamol use and the need for starting renal replacement therapy (RRT).

**Patients and methods/materials and methods:** We conducted a propensity score-matched cohort study in a 1136-bed, public, university affiliated and teaching hospital. All patients with serum creatine phosphokinase (CK) level > 5000 IU/L between January 1, 2008 and December 31, 2017 were included. A propensity score was calculated for each included patient by using multivariable logistic regression and all available baseline characteristics. The main outcome was the incidence of RRT initiation from day 1 to day 28 in the propensity score-matched cohort between patients exposed and unexposed to paracetamol.

**Results:** Over the study period, 1065 patients with at least one CK level > 5000 IU/L were included; 40 (3.8%) had at least one RRT session. Among the 343 matched pairs, 10 (2.9%) exposed and 24 (7.0%) unexposed patients underwent RRT before day 28 (*P* = 0.021). Primary time-to-event analysis showed that the exposure to paracetamol was significantly associated with reduced absolute risk of RRT: absolute risk difference = − 3.18% (95% CI: − 5.23 to − 1.20, *P* = 0.001) (Fig. 1). All secondary analyses showed a significantly reduced absolute risk of RRT in patients exposed to paracetamol.

**Conclusion:** Our study showed a significant association between paracetamol exposure and reduced incidence of RRT among patients with rhabdomyolysis.

**Reference**

1. Boutaud O, Moore KP, Reeder BJ, et al. (2010) Acetaminophen inhibits hemoprotein-catalyzed lipid peroxidation and attenuates rhabdomyolysis-induced renal failure. Proceedings of the National Academy of Sciences 107:2699–704. 10.1073/pnas.0.

**Compliance with ethics regulations:** Yes in clinical research.
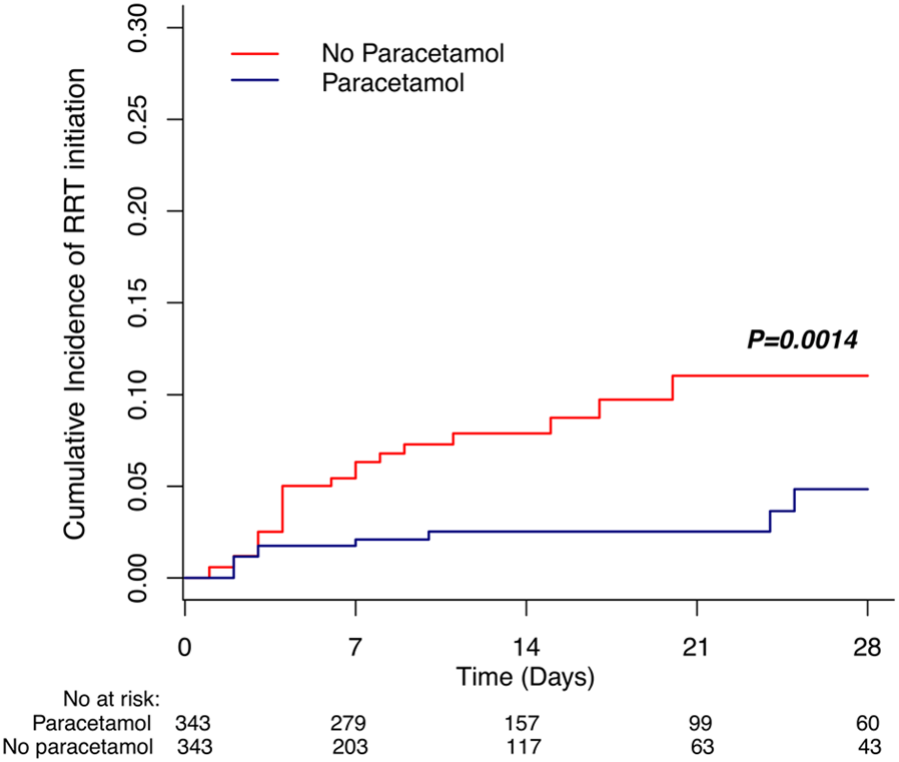


Cumulative incidence of RRT initiation in the 343 * 2 matched population by exposure. Incidence of RRT initiation was significantly lower for patients exposed than not exposed to paracetamol. ARD = − 3.18% (95%CI: − 5.23 to − 1.20), *P* = 0.001.

### CO-104 Clinical trajectories and impact of acute kidney disease after acute kidney injury in intensive care unit: a 5-year single-center cohort study

#### Arthur Orieux^1^, Mathilde Prezelin-Reydit^2^, Renaud Prevel^1^, Christian Combe^1^, Didier Gruson^1^, Sébastien Rubin^1^, Alexandre Boyer^1^

##### ^1^CHU Bordeaux, Bordeaux, France; ^2^AURAD AQUITAINE, Gradignan, France

**Correspondence:** Arthur Orieux - arthur.orieux@chu-bordeaux.fr

*Annals of Intensive Care* 2021, **11(Suppl 1):**CO-104

**Rationale:** Patients suffering from acute kidney injury (AKI) in intensive care unit (ICU) could have various renal trajectories and outcomes. Aims were to assess the various clinical trajectories after AKI in ICU and to determine risk factors for developing chronic kidney disease (CKD) taking into account the new concept of acute kidney disease (AKD).

**Patients and methods/materials and methods:** We conducted a prospective 5-year follow-up study in a medical ICU in a teaching hospital (France). The patients who received invasive mechanical ventilation, catecholamine infusion or both and developed an AKI (defined by KDIGO criteria) from September 2013 to May 2015 were included. AKD was defined as a condition wherein the criteria for AKI persists ≥ 7 days. Renal recovery was defined by creatinine ≤ 125% of basal creatinine. CKD was defined by an estimated glomerular filtration rate (eGFR) of < 60 ml/min/1.73 m^2^ at least 90 days after the AKI.

**Results:** 232 patients were enrolled. Age was 62 ± 16 years. Median follow-up was 52 [6–1553] days. At day 7, 109/232 (47%) had been progressing to AKD and 65/232 (28%) had recovered (Fig. 1). Among the AKD patients, 21/109 (19%) recovered before day 90, 41/109 (38%) died and 47/109 (43%) progressed to CKD to which are added 16 patients who developed CKD during follow-up without having had AKD. The cumulative incidence of CKD was 30 [24−36]% at 5-year follow-up. This incidence was higher in AKD patients (48 [39−58]%) than in non-AKD patients (22 [10−34]%) after 5 years of follow-up (*p* < 0.0001). In multivariate analysis, the hazard of developing CKD in AKD patients was increased up to 6 months compared to those without AKD (HR (hazard radio) 42.6 [9.7−186.1]) but was not different between AKD patients and non-AKD patients (HR 2.2 [0.6−7.9]) after 6 months. In patients alive without CKD at day 90, AKD was not associated with CKD (HR 0.7 [0.1−4.2]).

**Conclusion:** There were many clinical trajectories after AKI in ICU. AKD was the main risk factors for developing CKD at short-term and appeared to be no longer at risk in patients who had not developed CKD after 6 months.

**Compliance with ethics regulations:** Yes in clinical research.
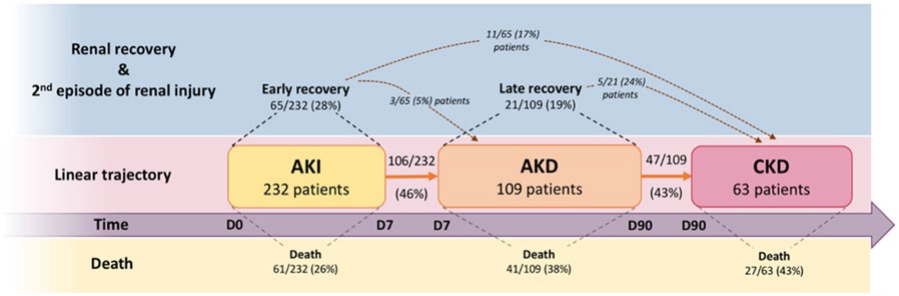


Fig. 1 Clinical trajectories after acute kidney injury

### CO-105 Five years outcome of critically ill patients with severe metabolic acidemia: a post hoc analysis of a multicentric trial

#### Eddine Bendiab^1^, Fanny Garnier^1^, Vincent Brunot^1^, Claudine Gniadek^1^, Kada Klouche^1^, Samir Jaber^2^, Marion Soler^1^, Nicolas Molinari^1^, Boris Jung^1^

##### ^1^Centre Hospitalier Universitaire Lapeyronie, Montpellier, France; ^2^Centre Hospitalier Universitaire de Saint-Eloi, Montpellier, France

**Correspondence:** Eddine Bendiab - eddine.bendiab@gmail.com

*Annals of Intensive Care* 2021, **11(Suppl 1):**CO-105

**Rationale:** Although there are many studies on acid–base abnormalities in ICU patients, specific studies focusing on severe acidemia are rare and long-term outcome of this population are unknown. The aim of this study was to assess the long-term survival and quality of life of critically ill patients with severe metabolic acidemia.

**Patients and methods/materials and methods:** We performed a post hoc analysis of the BICARICU trial1 that suggested that sodium bicarbonate infusion is associated with a decrease of mortality in patients with severe metabolic acidemia (pH < 7.20) and moderate-to-severe acute kidney injury (AKI). Herein, we assessed the 5-year survival and quality of life of these patients through a telephone interview using the Short-Form 36 (SF-36) and the EuroQol health related quality of life questionnaires (EQ-5D). The SF-36 consists of eight scaled scores into a 0–100 scale (the lower the score the more disability). The EQ-5D measures health status in terms of five dimensions ranging from 1 to 5, where 1 represents the best score possible. Parametric and non-parametric tests were used and a *p* value less than 0.05 was considered significant.

**Results:** Of the 389 patients included, 273 died (70%), 42 were lost to follow-up and 12 were unable to answer a questionnaire. Sixty-two patients were eventually included in the present study. Overall 5-year survival was 30% with no significant difference between the bicarbonate and the control groups. For the control and bicarbonate groups, respectively, SF-36 associated social functioning domain was conserved (scores: 82/100 and 75/100) with a low limitation due to emotional problem (scores: 96/100 and 85/100), whereas physical health was decreased (score: 64/100 and 50/100) as well as fatigue (score: 48/100 and 53/100) and patients’ feelings about their general state of health (score: 59/100 and 55/100) (Fig. 1). Quality of life reported using EQ-5D showed 9/62 (14%) patients with severe-to-extreme difficulty to perform usual activities and a relatively well-conserved self-care capacity 49/62 (79%) patients being able to wash and dress themselves without difficulty. There was no significant difference between the bicarbonate and the control groups on each domains of SF-36 and EQ-5D scores.

**Conclusion:** Five-year survival of patients with severe metabolic acidemia is 30%. In survivors, the quality of life is lower than what is expected, with a focus in the physical domains, but with reasonable scores in emotional and social functioning. The impact of rehabilitation in this high-risk population is unknown.

**Reference**

1. Jaber et al. Lancet 2018; 392: 31–40. 10.1016/s0140-6736(18)31080-8.

**Compliance with ethics regulations:** Yes in clinical research.
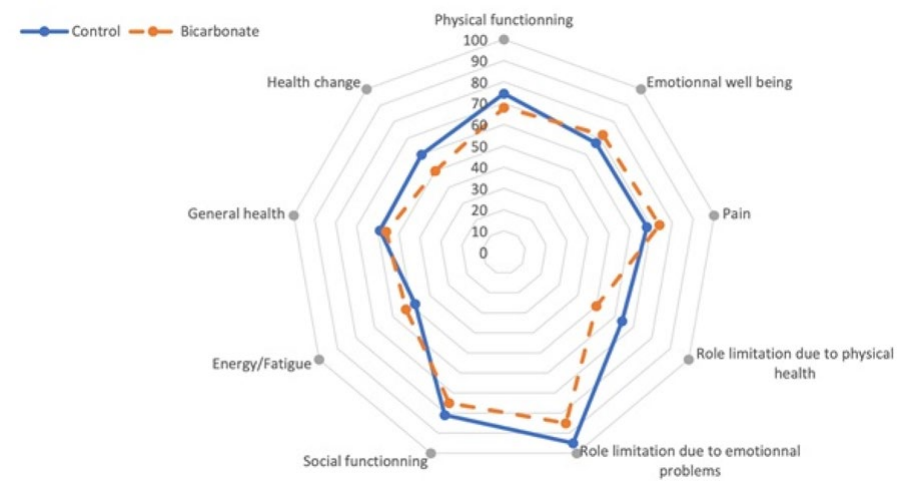


Fig. 1 Radar chart with Short-Form (SF)-36 quality of life score for the 62 5-year survivors and eligible patients (16%) who received sodium bicarbonate therapy or control patients who did not receive sodium bicarbonate therapy. For each component

### CO-106 Continuous renal replacement therapy versus intermittent haemodialysis as first modality for renal replacement therapy in severe acute kidney injury

#### Stéphane Gaudry^1^, François-Camille Grolleau^2^, Saberv Barbar^3^, Laurent Martin-Lefevre^4^, Bertrand Pons^5^, Eric Boulet^6^, Alexandre Boyer^7^, Guillaume Chevrel^8^, Florent Montini^12^, Julien Bohe^13^, Julio Badie^14^, Jean-Philippe Rigaud^15^, Christophe Vinsonneau^9^, Raphael Porcher^2^, Jean-Pierre Quenot^10^, Didier Dreyfuss^11^

##### ^1^Hôpital Avicenne, Bobigny, France; ^2^Hôtel Dieu, Paris, France; ^3^CHU Nimes, Nimes, France; ^4^CHD La Roche sur Yon, La Roche Sur Yon, France; ^5^CHU les Abymes, Pointe À Pitre, France; ^6^CH René Dubos, Pontoise, France; ^7^CHU Bordeaux, Bordeaux, France; ^8^CH Sud Francilien, Corbeil Essone, France; ^9^CH Béthune, Béthune, France; ^10^CHU Dijon, Dijon, France; ^11^Hôpital Louis Mourier, Colombes, France; ^12^CH d’Avignon, Avignon, France; ^13^ Hospices Civils de Lyon, Lyon, France; ^14^Hôpital Nord Franche Comté, Trévenans, France; ^15^CH Dieppe, Dieppe, France

**Correspondence:** Stéphane Gaudry - stephanegaudry@gmail.com

*Annals of Intensive Care* 2021, **11(Suppl 1):**CO-106

**Rationale:** Intermittent haemodialysis (IHD) and continuous renal replacement therapy (RRT) with haemofiltration (CRRT) are the two main RRT modalities used in the intensive care unit for patients with severe acute kidney injury (AKI). Meta-analyses conducted more than 10 years ago did not show survival differences between these two modalities, but indication for RRT was not clear. We aimed to reassess whether the choice of IHD or CRRT as first modality affects survival of critically ill patients with severe AKI.

**Patients and methods/materials and methods:** Secondary analysis of two trials (AKIKI and IDEAL-ICU) that compared an early initiation strategy with a delayed one. We merged the two datasets and included patients allocated to the early strategy in order to emulate a trial where patients would have been randomized to receive either IHD or CRRT within 12 h after the documentation of severe AKI. We determined each patient’s modality group as the first RRT modality they received. The primary outcome was 60-day overall survival. The analysis relied on inverse probability of treatment weighting (IPTW) to balance the differences in baseline characteristics between groups. A propensity score model was estimated using logistic regression with the first RRT modality as dependent variable.

**Results:** A total of 543 patients were included. Continuous RRT was the first modality in 269 patients (50%) and IHD in 274 (50%). Patients receiving CRRT had higher haemodynamic and total-SOFA scores. Inverse probability weighting allowed to balance the groups on all predefined confounders. Through the 60-day follow-up period, 146 patients in the CRRT group and 127 in the IHD group died. The weighted Kaplan–Meier death rate at day 60 was 54.4% in the CRRT group and 46.5% in the IHD group (weighted HR 1·26, 95% CI 1·01 to 1·60) (Fig. 1). In further IPTW analyses, ICU mortality, hospital mortality, renal function recovery at day-28 did not differ between groups.

**Conclusion:** Compared to IHD, CRRT as first RRT modality was associated with reduced survival within 60 days after adjustment for confounders through propensity scores methods. Given the provocative results of the study, we believe a prospective randomized non-inferiority trial should be implemented.

**Compliance with ethics regulations:** Yes in clinical research.
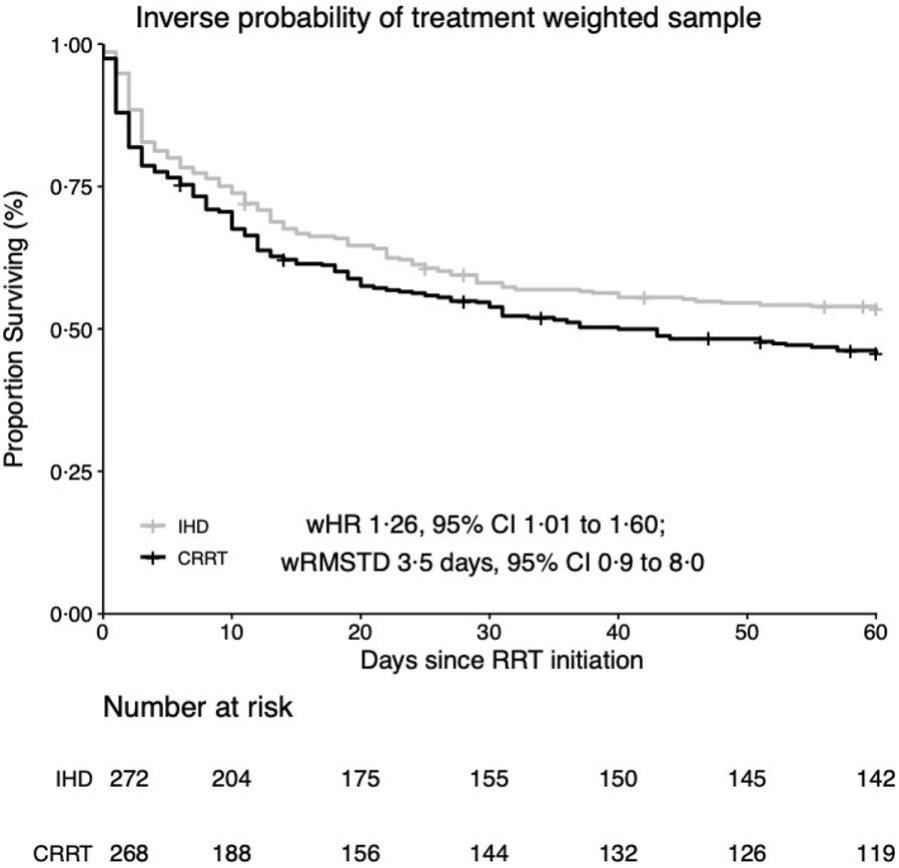


Inverse probability of treatment-weighted sample

### CO-107 Acute kidney injury in SARS-CoV2-related pneumonia ICU patients: a retrospective multicenter study

#### Guillaume Geri^1,2,4^, Michael Darmon^3,10,12^, Lara Zafrani^3,11,12^, Muriel Fartoukh^5,7^, Guillaume Voiriot^5,7,8^, Julien Le Marec^6,7,9^, Saafa Nemlaghi^6,7,9^, Antoine Vieillard-Baron^1,2,4^, Elie Azoulay^3,10,12^

##### ^1^Hôpital Ambroise Paré, Boulogne Billancourt, France; ^2^Université Paris Saclay, Versailles, France; ^3^Hôpital Saint Louis, Paris, France; ^4^INSERM UMR 1018, Villejuif, France; ^5^Hôpital Tenon, Paris, France; ^6^Groupe Hospitalier Pitié Salpétrière, Paris, France; ^7^Université Paris Sorbonne, Paris, France; ^8^INSERM U955, Créteil, France; ^9^INSERM UMRS1158, Paris, France; ^10^INSERM U1153, Paris, France; ^11^INSERM U976, Paris, France; ^12^Université de Paris, Paris, France

**Correspondence:** Guillaume Geri - guillaume.geri@aphp.fr

*Annals of Intensive Care* 2021, **11(Suppl 1):**CO-107

**Rationale:** While acute kidney injury (AKI) is frequent in severe SARS-CoV2-related pneumonia ICU patients, few data are still available about its risk factors.

**Patients and methods/materials and methods:** Retrospective observational study performed in four university-affiliated hospitals in Paris. AKI was defined according to the KIDGO guidelines. Factors associated with AKI were picked up using multivariable mixed-effects logistic regression. Independent risk factors of day-28 mortality were assessed using Cox model.

**Results:** 379 patients (median age 62 [53,69], 77% of male) were included. Half of the patients had AKI (*n* = 195, 52%) including 58 patients (15%) with AKI stage 1, 44 patients (12%) with AKI stage 2, and 93 patients (25%) with AKI stage 3. Chronic kidney disease (OR 7.41; 95%CI 2.98–18.4), need for invasive mechanical ventilation at day 1 (OR 4.83; 95%CI 2.26–10.3), need for vasopressors at day 1 (OR 2.1; 95%CI 1.05–4.21) were associated with increased risk of AKI. Day 28 mortality in the cohort was 26.4% and was higher in patients with AKI (37.4 vs. 14.7%, *P* < 0.001). Neither AKI (HR 1.35; 95%CI 0.78–2.32) nor AKI stage were associated with mortality (HR [95%CI] for stage 1, 2 and 3 when compared to no AKI of, respectively, 1.02 [0.49–2.10], 1.73 [0.81–3.68] and 1.42 [0.78–2.58]).

**Conclusion:** In this large cohort of SARS-CoV2-related pneumonia patients admitted to the ICU, AKI was frequent, mostly driven by preexisting chronic kidney disease and life-sustaining therapies, with unclear adjusted relationship with day-28 outcome.

**Compliance with ethics regulations:** Yes in clinical research

### CO-108 The clinico-pathological spectrum of renal lesions in the course of COVID-19

#### Matthieu Jamme^1^, Sophie Ferlicot^2^, François Gaillard^3^, Julie Oniszczuk^4^, Aymeric Couturier^5^, Olivia May^6^, Anne Grünenwald^2^, Aurélie Sannier^3^, Anissa Moktefi^4^, Ophélie Le Monnier^7^, Camille Petit Hoang^8^, Nadine Maroun^1^, Albane Brodin Sartorius^9^, Arthur Michon^2^, Hélène Dobosziewicz^2^, Fabrizio Andreelli^7^, Matthieu Guillet^2^, Hassan Izzedine^10^, Christian Richard^2^, Manon Dekeyser^2^, Romain Arrestier^4^, Thomas Sthelé^4^, Edouard Lefèvre^2^, Alexis Mathian^7^, Christophe Legendre^12^, Charlotte Mussini^2^, Marie Christine Verpont^8^, Nicolas Pallet^11^, Zahir Amoura^7^, Marie Essig^5^, Renaud Sanoudj^9^, Isabelle Brocheriou-Spelle^7^, Hélène François^8^, Xavier Belenfant^6^, Eric Daugas^3^, Vincent Audard^4^, David Buob^8^, Ziad Massy^5^, Mohamad Zaidan^2^, Guillaume Geri^5^

##### ^1^Centre Hospitalier Poissy Saint Germain en Laye, Poissy, France; ^2^Hopital Bicetre - APHP, Le Kremlin Bicetre, France; ^3^Hopital Bichat - APHP, Paris, France; ^4^Hopital Mondor - APHP, Créteil, France; ^5^Hopital Ambroise Paré - APHP, Boulogne Billancourt, France; ^6^Hôpital André Grégoire, Montreuil, France; ^7^Hopital Pitié Salpétrière - APHP, Paris, France; ^8^Hopital Tenon - APHP, Paris, France; ^9^Hopital Foch, Suresnes, France; ^10^Hopital Privé des Peupliers, Paris, France; ^11^Hopital Européen Georges Pompidou - APHP, Paris, France; ^12^Hopital Necker - APHP, Paris, France

**Correspondence:** Matthieu Jamme - matthieu.jamme@ght-yvelinesnord.fr

*Annals of Intensive Care* 2021, **11(Suppl 1):**CO-108

**Rationale:** The spectrum of kidney damage in COVID-19 has been mostly limited to autopsies and case reports. Here, we report the first case series of patients with COVID-19 who developed kidney injury and underwent a kidney biopsy in the Paris and its metropolitan area.

**Patients and methods/materials and methods:** We included all patients with COVID-19 who underwent a kidney biopsy between March 08 and May 19, 2020. Data were obtained through the review of the medical records.

**Results:** Forty-seven patients (80.9% men) with COVID-19 were included in the present study. Median age was 63 years IQR [52–69]. Comorbidities included hypertension (66.0%), diabetes mellitus (27.7%), previous history of chronic kidney (25.5%), cardiac (38.6%) and respiratory (27.3%) diseases. Initial symptoms were fever (85.1%), cough (63.8%), shortness of breath (55.3%), and diarrhea (23.4%). Almost all patients developed acute kidney injury (97.9%) and 63.8% required renal replacement therapy. Kidney biopsy analysis showed two main histopathological patterns, including isolated acute tubular necrosis in 22 (46.8%) patients, and dominant glomerular injury consisting of collapsing glomerulopathy or focal segmental glomerulosclerosis in 17 (36.2%) patients. Eight (17%) patients had alternative diagnosis, which were most likely unrelated to COVID-19. Acute tubular necrosis occurred almost invariably in the setting of severe forms of COVID-19, whereas patients with glomerular injury had various profiles of COVID-19 severity and collapsing glomerulopathy was only observed in patients harboring a combination of APOL1 risk variants. At last follow-up, 16 of the 30 patients who initially required dialysis were still on dialysis, and 9 died from COVID-19 or related-adverse events.

**Conclusion:** The present study describes clinico-pathological spectrum of kidney lesions in yet-alive patients with COVID-19. While acute tubular necrosis is correlated with COVID-19 severity, the pattern of glomerular injury is intimately associated with the expression of APOL1 risk variants. Whether SARS-CoV-2 directly underlies renal damage remains a major but unsolved issue.

**Compliance with ethics regulations:** Yes in clinical research.

### CO-109 Four-month clinical assessment of a cohort of patients following hospitalization for COVID-19

#### Tài Pham^1^, Luc Morin^1^, Laurent Savale^1^, Romain Colle^1^, Samy Figueiredo^1^, Anatole Harrois^1^, Matthieu Gasnier^1^, Anne-Lise Lecoq^1^, Olivier Meyrignac^1^, Nicolas Noël^1^, Elodie Baudry^1^, Marie-France Bellin^1^, Antoine Beurnier^1^, Walid Choucha^1^, Emmanuelle Corruble^1^, Laurent Dortet^1^, Isabelle Hardy-Léger^1^, François Radiguer^1^, Sabine Sportouch^1^, Christiane Verny^1^, Benjamin Wyplosz^1^, Mohamad Zaidan^1^, Laurent Bequemont^1^, David Montani^1^, Xavier Monnet^1^

##### ^1^Hôpital Bicêtre, Le Kremlin-Bicêtre, France

**Correspondence:** Tài Pham - tai.pham@aphp.fr

*Annals of Intensive Care* 2021, **11(Suppl 1):**CO-109

**Rationale:** Healthcare systems worldwide faced a surge of patients with COVID-19 showing a broad spectrum of clinical presentation from pauci-symptomatic to severely critically ill. Little is known, but much attention is paid on potential long-term COVID [1]. We aimed to describe the symptoms and complaints of COVID-19 survivors 4 months after hospital discharge.

**Patients and methods/materials and methods:** This is a prospective single-center study (NCT04704388). COVID-19 survivors admitted in the participating hospital between March and May 2020, were invited to a teleconsultation investigating respiratory, cognitive, and functional symptoms 3 months after discharge. Patients with relevant symptoms and all intensive care unit (ICU) survivors were invited to an outpatient clinic 4 months after hospital discharge. At the outpatient clinic, patients underwent pulmonary function tests, lung CT-scan, psychometric and cognitive tests and, in ICU or symptomatic patients, echocardiography

**Results:** Among 834 eligible patients, 478 were evaluated by telephone consultation and 177 consulted at the outpatient clinic, including 97 ICU patients (Table 1). Half of these 97 ICU patients had received invasive mechanical ventilation (75%, *N* = 51). Lung CT-scan showed abnormalities in 108/170 (63%) cases, mainly subtle ground-glass opacities. Fibrotic lesions were observed in 33/170 (19%) patients, mostly ICU patients, involving < 25% of parenchyma in all but one case. Fibrotic lesions were observed in 19 (39%) patients with acute respiratory distress syndrome (ARDS), a prevalence similar as to previous cohorts of patients with non-COVID-19 ARDS. New-onset dyspnea was found in 78 (16%) of the 478 patients, mainly in ICU survivors (31% vs 10%, *p* < 0.001). Dyspnea was related to lung sequelae in 44 (56%) cases and dysfunctional breathing in 14 (18%) cases. Cognitive complaint was reported by 86/416 (21%) patients at teleconsultation, and cognitive impairment was diagnosed in 61/159 (38%) outpatients. Anxiety, depression and post-traumatic symptoms were observed in 22 (24%), 17 (18%) and 7 (7%) ICU patients, respectively. The left ventricular ejection fraction was < 50% in 10 (12%) ICU patients. Two new-onset chronic kidney diseases were observed, both in ICU patients.

**Conclusion:** Four months after hospitalization, severe lung sequelae were rare, concerning mainly ICU patients. In COVID-19 ARDS patients, lung sequelae were similar to those reported in non-COVID-19 ARDS. These findings are limited by the absence pre-COVID assessments and control group; further research is needed to understand longer-term outcomes.

**Reference**

1. Yelin D, Wirtheim E, Vetter P, Kalil AC, Bruchfeld J, Runold M, et al. Long-term consequences of COVID-19: research needs. Lancet Infect Dis. 2020;20:1115–7

**Compliance with ethics regulations:** Yes in clinical research.
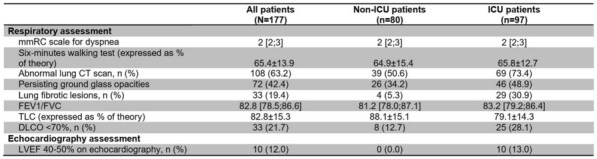


Respiratory and cardiac assessments of 177 patients who attended the outpatient clinic

### CO-110 Characteristics, management, and prognosis of elderly patients with COVID-19 admitted in the ICU during the first wave Insights from the COVID-ICU study

#### Martin Dres^1^, David Hajage^1^, Said Lebbah^1^, Antoine Kimmoun^2^, Tai Pham^3^, Gaetan Beduneau^4^, Alain Combes^1^, Alain Mercat^5^, Bertrand Guidet^6^, Alexandre Demoule^1^

##### ^1^Hôpital Pitié Salpêtrière, Paris, France; ^2^CHRU de Nancy, Nancy, France; ^3^Hôpital Bicêtre, Le Kremlin Bicêtre, France; ^4^Rouen University Hospital, Rouen, France; ^5^CHU d’Angers, Angers, France; ^6^Hôpital Saint Antoine, Paris, France

**Correspondence:** Martin Dres - martin.dres@aphp.fr

*Annals of Intensive Care* 2021, **11(Suppl 1):**CO-110

**Rationale:** The COVID-19 pandemic is a heavy burden in terms of health care resources. Future decision-making policies require consistent data on the management and prognosis of the older patients with COVID-19 admitted in the intensive care unit (ICU). The present study aimed at describing the characteristics, management, and prognosis of critically ill old patients (> 70 years)

**Patients and methods/materials and methods:** Data were extracted from the international prospective COVID-ICU database. A propensity score weighted-comparison evaluated the impact of intubation upon admission on Day-90 mortality.

**Results:** The analysis included 1199 (28% of the COVID-ICU cohort) patients (median [interquartile] age 74 [72–78] years). Fifty-three percent, 31%, and 16% were 70–74, 75–79, and over 80 years old, respectively. The most frequent comorbidities were chronic hypertension (62%), diabetes (30%), and chronic respiratory disease (25%). Median Clinical Frailty Scale was 3 (2–3). Upon admission, the PaO2/FiO2 ratio was 154 (105–222). 740 (62%) patients were intubated on Day-1 and eventually 938 (78%) during their ICU stay. Overall Day-90 mortality was 46% and reached 67% among the 193 patients over 80 years old. Mortality was higher in older patients, diabetics, and those with a lower PaO2/FiO2 ratio upon admission, cardiovascular dysfunction, and a shorter time between first symptoms and ICU admission. In propensity analysis, early intubation at ICU admission was associated with a significantly higher Day-90 mortality (42% vs 28%; hazard ratio 1.68; 95% CI, 1.24–2.27; *p* < 0.001).

**Conclusion:** Patients over 70 years old represented more than a quarter of the COVID-19 population admitted in the participating ICUs during the first wave. Day-90 mortality was 46%, with dismal outcomes reported for patients older than 80 years or those intubated upon ICU admission.

**Compliance with ethics regulations:** Yes in clinical research.

### CO-111 Inter-hospital transport of critically ill patients to manage intensive care unit surge during COVID-19 pandemic in France

#### Benoît Painvin^1^, Helene Messet^3^, Maeva Rodriguez^2^, Thomas Lebouvier^1^, Delphine Chatellier^2^, Louis Soulat^1^, Stephane Ehrmann^3^, Arnaud Thille^2^, Arnaud Gacouin^1^, Jean-Marc Tadie^1^

##### ^1^CHU RENNES, Rennes, France; ^2^CHU POITIERS, Poitiers, France; ^3^CHU TOURS, Tours, France

**Correspondence:** Benoît Painvin - benoit.painvin@chu-rennes.fr

*Annals of Intensive Care* 2021, **11(Suppl 1):**CO-111

**Rationale:** COVID-19 pandemic led authorities to evacuate via various modes critically ill ventilated patients in less crowded units. However, it is not known if interhospital transport impacts COVID-19 patient’s mortality in intensive care unit (ICU).

**Patients and methods/materials and methods:** Data were retrospectively analyzed from medical records in 3 teaching hospitals in West of France (Poitiers, Rennes, and Tours). All patients above 18 years old admitted to these 3 ICUs from the 15th of March until the 15th of April and requiring invasive mechanical ventilation for COVID-19 confirmed by reverse transcriptase-polymerase chain reaction test were included. The main objective was to compare ICU mortality between patients who underwent inter-hospital transfer during their ICU stay and patients who did not. Secondary objectives were the comparisons of ICU-acquired infections, length of mechanical ventilation, length of sedation and use of neuromuscular blocking agents, length of vasopressor use, ICU and hospital length of stay between the two groups.

**Results:** Among the 133 patients included in the study, 95 (71%) were male patients and median age was 63 years old (interquartile range: 54–71). Overall ICU mortality was 11%. Mode of transport included train (48 patients), ambulance (6 patients), and plane plus helicopter (14 patients). During ICU stay, 7 (10%) transferred patients and 8 (12%) non-transferred patients died (*p* = 0.71). Median SAPS II score at admission was 33 (interquartile range: 25–46) for the transferred group and 35 (27–42) for non-transferred patients (*p* = 0.53). SOFA score at admission was 4 (3–6) for the transferred group versus 3 (2–5) for the non-transferred group (*p* = 0.25). In the transferred group, median PaO2/FiO2 ratio (P/F) value within 24 h before departure was 197 mmHg (160–250) and remained − 166 mmHg (125–222) in the first 24 h post-arrival (*p* = 0.13). During the evacuation 46 (68%) and 21 (31%) of the patients, respectively, beneficiated from neuromuscular blocking agents and from vasopressors. Transferred and non-transferred patients had similar rate of nosocomial infections, respectively, 37/68 (54%) versus 34/65 (52%) (*p* = 0.80). Median length of mechanical ventilation was significantly increased in the transferred group compared to the non-transferred group, 18 days (11–24) and 14 days (8–20) (*p* = 0.007), respectively. Finally, ICU and hospital length of stay did not differ between both groups.

**Conclusion:** In France, inter-hospital evacuation of COVID-19 ventilated ICU patients did not appear to increase mortality and therefore could be proposed to manage ICU surge.

**Compliance with ethics regulations:** Yes in clinical research.
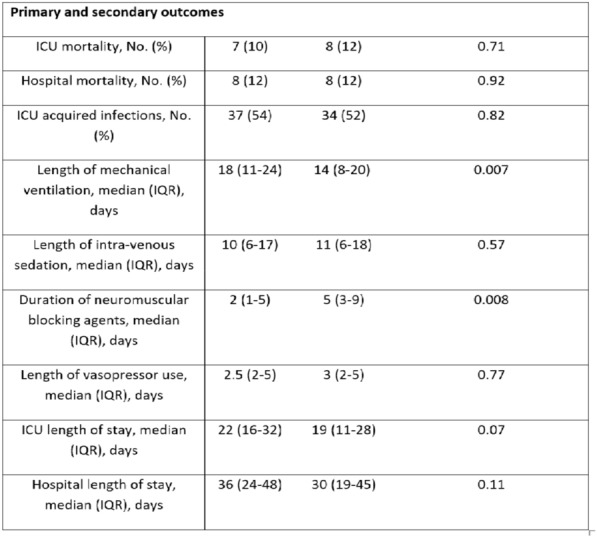


Primary and secondary outcomes

### CO-112 Outcomes of tracheotomized COVID-19 patients transferred to a weaning center

#### Morgane Faure^1^, Maxens Decavele^1^, Elise Morawiec^1^, Martin Dres^1^, Nicolas Gatulle^1^, Julien Mayaux^1^, Thomas Similowski^1^, Julie Delemazure^1^, Alexandre Demoule^1^

##### ^1^Hôpital Pitié-Salpétriêre, Paris, France

**Correspondence:** Morgane Faure - morgane.faure@aphp.fr

*Annals of Intensive Care* 2021, **11(Suppl 1):**CO-112

**Rationale:** Patients with acute respiratory distress syndrome due to COVID-19 may require tracheostomy and transfer to a weaning center. To date, data on the outcome of these patients are scarce. The objective of the study was (1) to determine the factors associated with time to decannulation and MRC gain; (2) to describe the outcome of these patients 3 months after admission to the weaning center.

**Patients and methods/materials and methods:** Observational retrospective study of COVID-19 patients requiring tracheostomy after prolonged ventilation and transferred in a weaning center from 2020 April the 4th to May the 30th.

**Results:** Forty-three patients were included. Median age (interquartile range) was 61 (48–66) years, 81% were men and body mass index (BMI) was 30 (26–35) kg m^2^. Tracheostomy was performed after 19 days of mechanical ventilation and the median length of stay in the intensive care unit prior to transfer to the weaning center was 27 days (22–40). On admission to the weaning center, the Medical Research Council (MRC) score was 36 (27–44) and 70% of patients had delirium. Time to decannulation was 9 (30–61) days after admission to the weaning center. The only factor independently associated with time to decannulation was the MRC score at admission to the weaning center (odds ratio [OR] 1.16, 95% confidence interval [CI] 1.06–1.31, *P* = 0.005). During weaning center stay, MRC gain was 10 (4–16). Two factors were independently associated with MRC gain ≥ 10: BMI (OR 0.88, 95% CI 0.76–0.99, *p* = 0.045) and MRC on admission (OR 0.91, 95% CI 0.82–0.98, *p* = 0.025). Three months after admission to the weaning center, 40 patients were weaned from mechanical ventilation. In addition, 36 patients (84%) were back home, 6 (14%) were still at hospital and 1 had died.

**Conclusion:** Three months after weaning center admission, a large majority of COVID-19 patients were weaned of mechanical ventilation and back home.

**Compliance with ethics regulations:** Yes in clinical research

### CO-113 Impact of pulmonary embolism on the progress of pneumonia related to COVID-19

#### Ahlem Trifi^1^, Nabil Bouguezzi^1^, Mohamed Abbessi^2^, Mouna Hachani^1^, Hichem Cherif^2^, Eya Seghir^1^, Yosri Masseoudi^2^, Oussema Benjima^2^, Yosr Touil^1^, Sami Abdellatif^1^, Adel Ammous^2^, Salah Ben Lakhal^1^

##### ^1^Medical ICU, la Rabta hospital, Faculty of Medicine of Tunis, Tunis, Tunisie; ^2^Surgical ICU, la Rabta hospital, Faculty of Medicine of Tunis, Tunis, Tunisie

**Correspondence:** Ahlem Trifi - trifiahlem2@gmail.com

*Annals of Intensive Care* 2021, **11(Suppl 1):**CO-113

**Rationale:** Coagulation disturbances are frequently observed in patients with COVID-19 as well as clinical thrombotic events. The most serious complication is pulmonary embolism (PE). Activation of inflammation and coagulation in addition to hypoxic vasoconstriction are the major phenomena in cause of occlusion of the pulmonary vessels during the COVID-19. Objective: to study the impact of PE on the progress of infection with SARS-CoV 2 in ICU patients: clinical presentation, biology, oxygenation and ventilatory mechanics, hemodynamic and survival.

**Patients and methods/materials and methods:** Prospective double-cohort study of ICU patients hospitalized for COVID-19: those who developed PE versus those who not developed PE. The diagnostic of PE was either confirmed by CT or strongly suspected in front of a bundle of arguments (electrocardiographic, blood gas and echocardiography). All the illness process elements were followed and compared between the 2 cohorts.

**Results:** A confirmed or strongly suspected PE was diagnosed in 29 patients among 143 hospitalized in ICU for acute respiratory failure related to COVID-19, i.e. an incidence of 20.3%. No difference was shown in regard to demographic characteristics between COVID-19 patients with PE vs COVID-19 patients without PE (age: 63 [53–69] vs 64 [57–71], *p* = 0.42, sex ratio: 22/7 vs 80/34, *p* = 0.8 and BMI: 27.5 [26–31] vs 27 [24–31], *p* = 0.72, respectively). Exposure to tobacco, comorbidities, severity scores and the clinical features (dyspnea, cough, chest pain and hemodynamic) were present in the 2 groups similarly. For the laboratory findings: 1st point of troponin, lactate and D-dimer were higher in PE group. Alanine aminotransferase (ALT) was also higher in this group with p near to significance. CT scan extension and initial blood gas were comparable. For cardiac ultrasound, in PE group: SPAP was significantly increased (43 mmHg [38–49] vs 31 [26–33], *p* < 10-3) and TAPSE was close to be lower (13.5 [11–17] vs 16 [13–19], *p* = 0.07). Regarding the disease progress: PE was a significant impact on P/F ratio at day 10 (reduced, *p* = 0.05), on respiratory compliance (elevated, *p* = 0.033) and plateau pressure (reduced, 0.029). PE-COVID-19 patients required more mechanical ventilation (MV) (65.5% vs 40%, *p* = 0.035). No impact of PE was observed on MV duration (*p* = 0.5), ICU length of stay (*p* = 0.77) and mortality (0.29). The compared values are displayed in the attached table.

**Conclusion:** Once PE occurred, it influenced the progress of a pneumonia related to COVID-19 mainly in the ventilatory behavior: increased compliance, decreased pressure plateau and P/F and required more invasive ventilation.

**Compliance with ethics regulations:** Yes in clinical research.
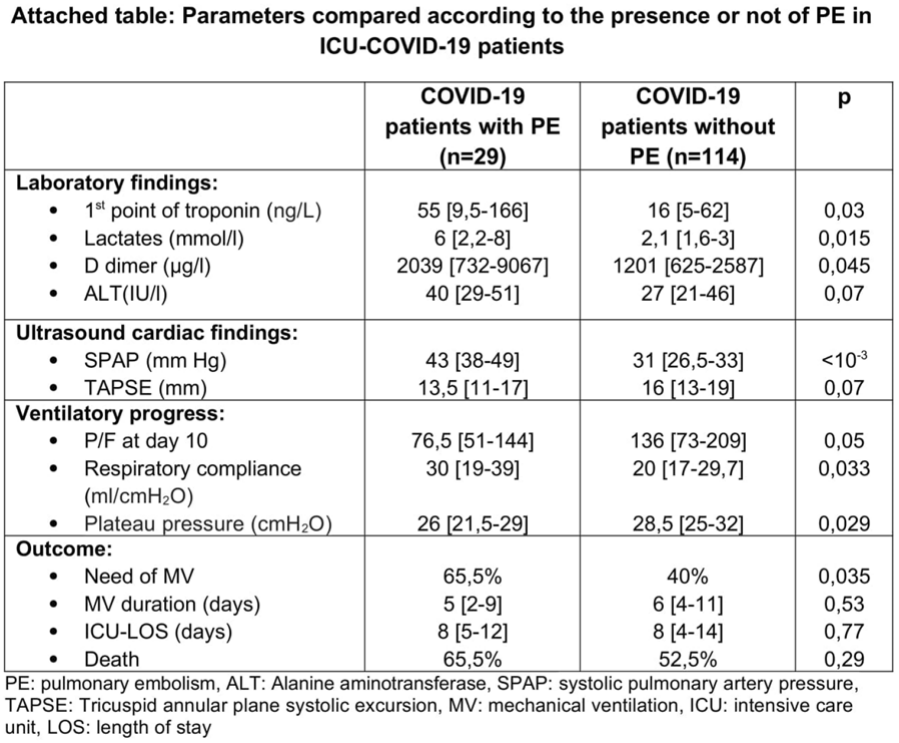


Attached table: Parameters according to the presence or not of PE in ICU-COVID-19 patients

### CO-114 Prognosis factors influencing 3-month respiratory outcomes in intensive care unit survivors after COVID-19 pneumonia

#### Sinan Karaer^1^, Fréderic Claudé^1^, Guillaume Eberst^1^, Lucie Laurent^1^, Aurélia Meurisse^1^, Pauline Roux-Claudé^1^, Cindy Barnig^1^, Kevin Bouiller^1^, Catherine Chirouze^1^, Julien Behr^1^, Franck Grillet^1^, Ophélie Ritter^1^, Sébastien Pili-Floury^1^, Hadrien Winiszewski^1^, Emmanuel Samain^1^, Gilles Capellier^1^, Virginie Westeel^1^

##### ^1^CHRU Jean Minjoz, Besançon, France

**Correspondence:** Sinan Karaer - karaer.sinan@yahoo.fr

*Annals of Intensive Care* 2021, **11(Suppl 1):**CO-114

**Rationale:** Outcomes of the most severe patients who have survived COVID-19 are abundantly described, but few data are currently available on the factors influencing these possible sequelae. We aimed to evaluate prognosis factors influencing outcomes in patients who survived severe COVID-19 pneumonia.

**Patients and methods/materials and methods:** All patients admitted to intensive care unit (ICU) were screened. Eligible patients were assessed 3 months after onset of SARS-CoV-2 symptoms. Patients underwent a physical examination, pulmonary function testing, chest CT and 6-min walk test (6MWT). The cohort was divided into patients with and without DLCO Impairment (DI) defining pulmonary sequalae, then we assessed risk factors for pulmonary sequalae.

**Results:** 84 patients (median age 68.6 years (IQR: 60.4–73), 78.6% male, 33.3% obese) participated. The median length of ICU stay was 17 days (IQR: 1–49). 85.7% required intubation and 84.3% were treated with curare, 73.5% with prone position, 69.1% with catecholamine, 4.8% with dialysis. Lowest PaO2/Fio2 ratio median was 125 (IQR: 56.2–318.8) and median value of ventilatory ratio (VR) was 1.74 (IQR: 1.43–2.09). 35 patients (41.7%) were diagnosed with DI (median DLCO of 61.8%). There was no difference between groups regarding age, gender, comorbidities, intubation, severity and VR. Groups differed in obesity, median IGS2 score and use of dialysis. At 3 months, patients with DI had higher prevalence of velcro crackles (34.3% vs 10.2%), irregular interface (62.9% vs 36.6%), traction bronchiectasis (85.7% vs 53.7%), a lower percentage of predicted TLC, KCO, SNIP, walk distance in 6MWT and more oxygen desaturation. In multivariate analysis, length of stay (LOS) in ICU ≥ 27 days (OR = 7.40) and serum creatinine level (SCL) ≥ 92.8 mmol/l (OR = 4.23) were risk factors for DI while obesity (OR = 0.069) was protective factor (*p* < 0.01).

**Conclusion:** Our findings suggest a residual pulmonary fibrosis at 3 months after onset disease. LOS in ICU and high SCL may be useful for prediction for DI while obesity can be protective factor. Follow-up of Covid-19 patients to manage any emerging or persistent long-term sequelae is needed.

**Compliance with ethics regulations:** Yes in clinical research.

## Flash communications

### FC-001 Prognostic value of lactate in predicting intensive care unit mortality

#### Chiheb Eddine Ben Maaouia^1^, Faten Haddad^1^, Cherifa Ben Miled^1^, Hajer Arfaoui^1^, Skander Naimi^1^, Asma Ben Souissi^1^, M’hamed Sami Mebazaa^1^

##### ^1^Hôpital Mongi Slim La Marsa, La Marsa, Tunisie

**Correspondence:** Chiheb Eddine Ben Maaouia - benmaaouia.chiheb@hotmail.fr

*Annals of Intensive Care* 2021, **11(Suppl 1):**FC-001

**Rationale:** Many prognostic indices have been developed to assess clinical status and predict the probability of death in the intensive care unit (ICU), but none have perfect sensitivity or specificity. Lactate which has been always seen as a metabolic waste and therapeutic dead end, is now considered as a powerful prognostic tool in ICU. The aim of our study was to evaluate the prognostic value of admission lactate in patients admitted to ICU.

**Patients and methods/materials and methods:** An analytic, prospective study was carried out in the ICU of Mongi Slim Hospital, la Marsa, over a 12-month period. Arterial bood lactate was measured at ICU admission (H0), then 6 h (H6), 12 h (H12), 24 h (H24) and 48 h (H48) after admission. Lactate superior to 2 mmol/l defined hyperlactatemia. Prognostic scores were calculated 24 h since the admission. We also recorded biological data, hemodynamic parameters and the evolution during the stay in intensive care. Primary endpoint was ICU mortality. The final step in the statistical study was to perform a multivariate analysis

**Results:** We included 135 patients. The average age was 47.22 ± 16.88 years. ICU mortaily was 48%. The mean serum lactate on admission was 3.05 ± 2.63 mmol/l, higher in the dead group with a statistically significant difference (*p* < 10-3). Patients with hyperlactatemia on arrival were clinically more serious with significantly higher prognostic scores. Prognostic value of lactate on admission was less powerful than severity indices in this study, but remain excellent with an AUC > 0.7 defining “cut-off” values with a good sensitivty and specificty. In multivaritate analysis, initial lactate > 2 mmol/l was found to be an independent predictive factor of ICU mortality with an odds ratio [IC 95%] = 1.16 [1.07–3.6]; *p* = 0.04. A derived score including lactate > 3.4 showed the best sensitivity and specificity to predict ICU mortality (84.6% and 75.5%, respectively).

**Conclusion:** Monitoring lactatemia in ICU could allow better identification of patients at high risk of death and the reassessment of therapeutic efficacy.

**Compliance with ethics regulations:** Yes in clinical research.
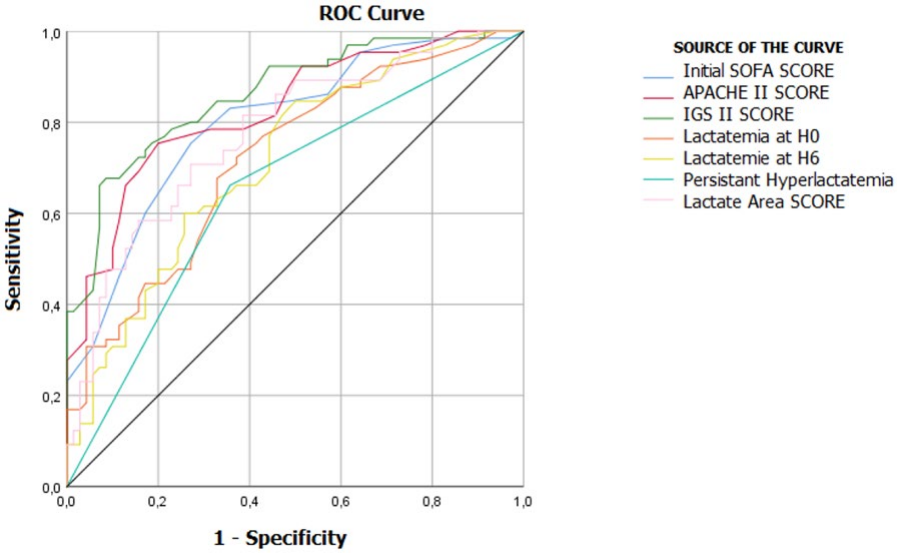


Superimposed ROC curves of the various predictives parameters of mortality in general ICU

### FC-002 Impact of insulin resistance on ICU death: a post hoc analysis of the CONTROLLING randomized control trial

#### François Thouy^1^, Claire Dupuis^1^, Hassane Abidi^1^, Vincent Brunot^1^, Nicholas Sedillot^1^, Jean-Pierre Quenot^1^, Hervé Hyvernat^1^, Florent Wallet^1^, Pierre Eric Danin^1^, Julio Badie^1^, Jérôme Morel^1^, Ali Mofredj^1^, Anne Mialon^1^, Abdelhamid Fatah^1^, Kada Klouche^1^, Xavier Tchenio^1^, Jean-Baptiste Roudaut^1^, Fabrice Thiollière^1^, Jean Dellamonica^1^, Bertrand Souweine^1^, Julien Bohé^1^

##### ^1^CHU Clermont Ferrand, Clermont Ferrand, France

**Correspondence:** Claire Dupuis - cdupuis1@chu-clermontferrand.fr

*Annals of Intensive Care* 2021, **11(Suppl 1):**FC-002

**Rationale:** The evolution of insulin resistance (IR) during intensive care unit (ICU) stay and the impact of IR on mortality remain to be determined. The objective of this study was to assess a non-biased effect of IR at day 3, 5 and 7 on Day-90 death

**Patients and methods/materials and methods:** It is a post hoc analysis of the CONTROLLING study, a multicenter double-blind randomized trial comparing ICU patients who underwent either individualized glucose control (GC) to target the patient’s pre-admission glycemia, or conventional GC to maintain glycemia below 180 mg/dl. From May 2015 to July 2016, all patients of the individualized GC group, with an ICU stay > 72 h were included. Patients treated for diabetes mellitus were excluded. Case mix, severity on admission and outcomes were collected. At Day 3, 5 and 7, insulin administered and caloric intakes, and GC were recorded. IR was defined as insulin requirement to control glycemia. The primary outcome was Day-90 mortality. The covariates assessed were IR, IR with and without GC, no-IR, insulin/caloric intakes and an increase in insulin intake between landmark day and landmark day-2. We used Fine and Gray sub-distribution survival models with ponderation with inverse probability of treatment weighting (IPTW) to model the risk of death (IPTWSubHR > 1 in favor of death) with ICU discharge as competing event, at landmark Day 3, 5 and 7.

**Results:** The study population included 572 patients, with a sex ratio of 1.5, a median age of 66 (53–76) years; body mass index of 26.2(23–30.2), HbA1c level of 5.7(5.3–6.1) and SAPS II of 49(37–63). A total of 572 patients were analysed at day 3, 476 at day 5, and 374 at day 7. On day 3, IR was observed in 354 (61.9%) patients (IR-group), including 51 (8%) without GC. In the study population, the median ICU length of stay was 8 days (5–15.5), and Day-90 mortality was 20.6%, with no-significant difference between the IR and the non-IR groups: 22.9% vs 17%, (*p* = 0.09), respectively. The covariates associated with an increased Day-90 mortality were the increase in insulin intake at day 3; and IR, IR with and without GC, and an increased in insulin/caloric intake at day 5. At day 7, none of these covariates were associated with Day-90 mortality (Table 1).

**Conclusion:** IR at Day-5 was associated with a poor outcome.

**Compliance with ethics regulations:** Yes in clinical research.
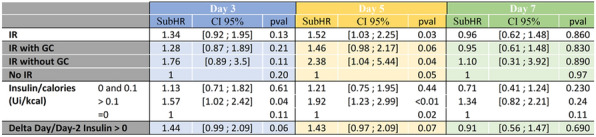


Associations between insulin resistance and other markers of insulin intakes and death by Day 90, using competing risk sub-distribution survival analyses with ponderation on IPTW

### FC-003 Prognostic value of C-reactive protein in Covid-19 patients

#### Emna Abid^1^, Yosr Touil^1^, Sawsen Ben Nessira^1^, Amal Mefteh^1^, Foued Daly^1^, Cyrine Abdennebi^1^, Ahlem Trifi^1^, Sami Abdellatif^1^, Salah Ben Lakhal^1^

##### ^1^Hôpital la RABTA, Tunis, Tunisi﻿e

**Correspondence:** Yosr Touil - touilyosr@hotmail.fr

*Annals of Intensive Care* 2021, **11(Suppl 1):**FC-003

**Rationale:** Covid-19 patients can develop a hyper-inflammatory syndrome which can impact on mortality. C-reactive protein (CRP), a routinely measured inflammatory marker, was increased in most patients with COVID-19. It can be used as an indicator of the severity of this disease. Objective: To evaluate the prognostic value of the CRP.

**Patients and methods/materials and methods:** This retrospective study was conducted in an ICU at a university hospital in Tunis (Tunisia) between September 2020 and January 2021. Covid-19 patients (determined by a positive PCR result for SARS-cov-2) in respiratory distress with a ratio PaO2/FiO2 less of than 300 were enrolled. All patients received dexamethasone and curative anticoagulation. They were divided into two groups: survivors and non-survivors. Data of demographic parameters, clinical characteristics, laboratory tests and outcomes were collected.

**Results:** Fourty-nine patients were included. There were 32 patients (65.3%) in the non-survivor group, whereas the survivor-group comprises 17 patients (34.7%). Non-survivors were older than the survivors (66 [57–72] versus 58 [52–64.5]), but the difference was not significant. Non-survivors had a significantly higher SOFA score at admission (4 [4–5] versus 3 [2–4], *p* < 0.001), a higher troponin level (292.53 versus 31.6; *p* = 0.002) and a lower PaO2/FiO2 (68 [60–91] versus 108 [90–189], *p* = 0.0001). There was no significant difference in white blood cell count (*p* = 0.081) nor in the lymphocyte count (*p* = 0.09). The percentage of patients requiring endo-tracheal intubation was significantly higher in the non-survivor group. (93.8% versus 2%, *p* = 0.0001). The area under the curve (AUC) of CRP used to assess Covid-19 mortality was 0.8 and the optimal cut-off for predicting mortality was a CRP value of 138 (specificity: 76% and sensitivity: 81%, PPV: 86% and NPV: 68%). In univariate analysis: a value of CRP greater than 138 was associated with mortality (*p* = 0.0001). In multivariate analysis: a value of CRP greater than 138 was the independent predictor of mortality (*p* = 0.013, OR: 7.59, CI95% [1.53–37.65]) while the elevation of PaO2/FiO2 was a protective factor (*p* = 0.023, OR:0.97, CI95% [0.94–0.99]).

**Conclusion:** The CRP level is correlated with morbidity and mortality. Thus, it can be used to identify the severe forms of the coronavirus disease.

**Compliance with ethics regulations:** Yes in clinical research.

### FC-004 Admission high-sensitive cardiac Troponin T levels are predictive of mortality in critically ill patients with COVID-19: a multicenter study

#### Romaric Larcher^1,4^, Noémie Besnard^1^, Aziz Akouz^2^, Emmanuelle Rabier^3^, Lauranne Teule^2^, Thomas Vandercamere^3^, Samuel Zozor^1^, Matthieu Amalric^1^, Racim Benomar^1^, Vincent Brunot^1^, Philippe Corne^1^, Olivier Barbot^2^, Anne-Marie Dupuy^1^, Jean-Paul Cristol^1,4^, Kada Klouche^1,4^

##### ^1^CHU Lapeyronie, Montpellier, France; ^2^CH Perpignan, Perpignan, France; ^3^CH Narbonne, Narbonne, France; ^4^PhyMedExp, Université de Montpellier, INSERM, CNRS, Montpellier, France

**Correspondence:** Romaric Larcher - r-larcher@outlook.fr

*Annals of Intensive Care* 2021, **11(Suppl 1):**FC-004

**Rationale:** In COVID-19 patients, increase in high-sensitive cardiac troponin T (hs-cTnT) has been reported to be associated with worse outcomes. Early identification of critically ill COVID-19 patients with the worst prognosis could be mandatory to provide better care to the greatest number of individuals. However, in critically ill COVID-19 patients, the prognostic value of hs-cTnT remains to be assessed given that most of previous studies have involved case mix of non- severely ill patients, and few ICU patients.

**Patients and methods/materials and methods:** This observational retrospective cohort study was carried out from March 9, 2020 to May 3, 2020 in three French ICUs. Investigators on each site collected prospectively clinical and biological data for all critically ill patients diagnosed with a SARS-CoV-2 infection confirmed by RT-PCR. Patients were followed up until hospital discharge or death and 3 months later by phone call. ICU, in-hospital, and 90-day mortalities were recorded. Predictive factors of in-hospital mortality were then identified.

**Results:** One hundredand eleven critically ill patients (68% of male), with median age of 67 [58–75] years, median Charlson index and Simplified Acute Physiology Score II of 3 [2–5] and 37 [27–48], respectively, were included. At ICU admission, median PaO2/FiO2 ratio was 140 [98–154]) and hs-cTnT serum level was 16.00 [10.15–31.85] ng/L. Seventy-five patients (68%) were mechanically ventilated, 41 (37%) were treated by norepinephrine (> 1 µg/kg/min), and 17 (15%) underwent renal replacement therapy. In-hospital mortality was 29% (32/111). In-hospital mortality was independently associated with higher age, lower PaO2/FiO2 ratio and higher hs-cTnT serum level. Age ≥ 74 years old, PaO2/FiO2 ≤ 100 and hs-cTnT ≥ 22 ng/L had a negative predictive value of 84%, 80% and 86%, respectively, but a low positive predictive value for in-hospital mortality.

**Conclusion:** At admission, age, low PaO2/FiO2 and elevated hs-cTnT serum level were independently associated with in-hospital mortality in critically ill COVID-19 patients. Besides age and PaO2/FiO2 ratio, hs-cTnT levels may allow early risk stratification and triage in such patients.

**Compliance with ethics regulations:** Yes in clinical research.
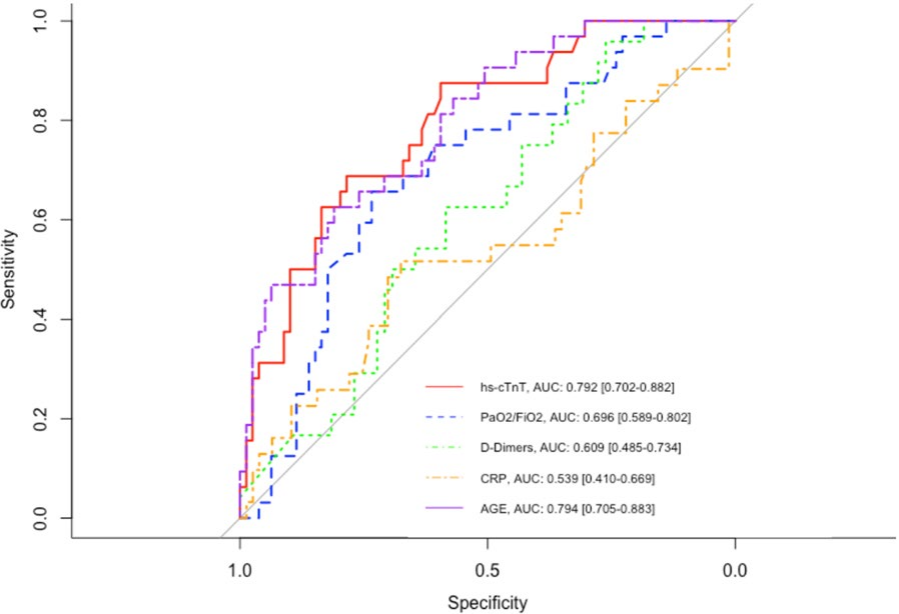


ROC curves of age (purple semi-dashed line), initial PaO2/FiO 2ratio (blue dashed line), initial high-sensitive cardiac troponin T (hs-cTnT, red line), initial D-dimers (green dotted line), and initial C-reactive protein (CRP, orange dashed and dotted lin

### FC-005 Hematological and biochemical abnormalities associated with severe forms of COVID-19

#### Ines Sdiri^1,3^, Fatma Essafi^1,3^, Imen Talik^1,3^, Sana Dhorgham^1,3^, Aida Bayyoudh^2^, Ilhem Fessi^2^, Moez Kaddour^1,3^, Takoua Merhabene^1,3^

##### ^1^Intensive care unit, Regional Hospital, Zaghouan, Tunisie; ^2^sevice de pneumologie, Hôpital réginal de Zaghouan, Tunis, Tunisie; ^3^Faculty of Medicine of Tunis, University Tunis El Manar, Tunis, Tunisie

**Correspondence:** Fatma Essafi - fatma.essafi@fmt.utm.tn

*Annals of Intensive Care* 2021, **11(Suppl 1):**FC-005

**Rationale:** Several studies have been conducted to identify the clinical, biological, and radiological risk factors associated with severe forms of COVID-19. This study aimed to analyze possible link between biological characteristics and stage of respiratory failure.

**Patients and methods/materials and methods:** A retrospective, observational cohort study of inpatients with COVID-19 pneumonia confirmed by swab test from 1st March 2020 to 31st January 2021 was performed. Patients were categorized according to their degree of acute respiratory failure: mild group (patients who required < 6 l/mn of oxygen) and severe group (≥ 6 l/mn). Data were pooled to compare the hematologic and biochemical findings between the two groups. Twenty parameters were analyzed.

**Results:** Our study involved 195 patients, 108 were assigned to the severe group (GI) and 87 in the mild one (G II). There were no significant differences between the 2 groups in gender (sex ratio 0.5 vs 0.9; *p* = 0.068) and age (63.5 ± 11.8 vs 62.5 ± 16; *p* = 0.63) whereas GI showed more frequently diabetes (41.7% vs 27.6%; *p* = 0.05), obesity (66.3% vs 44.2%; *p* = 0.02), and chronic obstructive pulmonary disease. The median time of symptoms at the first consultation was 9 ± 4.3 [1–30] days in GI vs 7.4 ± 5.4 [1–30]. Dry cough (81.7% vs 68.3; *p* = 0.03) fever (58.7% vs 41.3%; *p* = 0.15) and dyspnea (86.8% vs 74%; *p* = 0.02) were the most frequent clinical features. Time from illness onset to dyspnea and admission was significantly higher in GI (6 ± 2.7 [1–12] day vs 3.4 ± 3 [1–15]). Comparing biological parameters, we found significant differences in median levels of white blood cell count (WBC): 10279 elt/mm^3^ [3500–22300] vs 9072 elt/mm^3^ [2600–35000], Lymphocyte count (LYM) 1057 elt/mm^3^ [400–3100] vs 1515 elt/mm^3^ [420–2700], aspartate aminotransferase (AST) 52UI/L [9–477] vs 35UI/L [4–214], alanine aminotransferase (ALT) 43 UI/L [5–648] vs 28 UI/L [4–113], C-reactive protein (CRP) 121 mg/L [6–372] vs 88 mg/L [2–418] and glucose (GLU) level 11.5 mmol/L [4.5–41] vs 9.4 mmol/L [4.6–45.5]. At admission, median levels of urea, serum creatinine and lactate dehydrogenase, were higher in severe patients, but were not significantly different between the two groups. The cut-off critical point was WBC ≥ 10500 elt/mm^3^, LYM < 950 elt/mm^3^, AST ≥ 30 UI/L, ALT ≥ 35 UI/L, GLU ≥ 8.5 mmol/L and CRP ≥ 80 mg/L.

**Conclusion:** This study highlights the association of some biochemical parameters with the severity of COVID-19 pneumonia and might assist clinicians to closely monitor patients in order to predict prognosis.

**Compliance with ethics regulations:** N/A.

### FC-006 Outcomes of patients with thyroid disorders related to the coronavirus disease 2019

#### Wael Chamli^1^, Hajer Nouira^1^, Oussama Jaoued^1^, Mayssa Jrad^1^, Rim Gharbi^1^, Habiba Ben Sik Ali^1^, Mohamed Fekih Hassen^1^, Souheil Atrous^1^

##### ^1^Service de réanimation médicale hôpital Taher Sfar, Mahdia, Tunisie

**Correspondence:** Oussama Jaoued - oussamajaoued@gmail.com

*Annals of Intensive Care* 2021, **11(Suppl 1):**FC-006

**Rationale:** The coronavirus disease 2019 (COVID-19) has spread rapidly across the world and has caused an important public health burden. It is associated with high rates of morbidity and mortality. Therefore, identification of factors contributing to worse outcomes is crucial. Thyroid disorders seem to be associated with an increased risk of severity. However, the impact of thyroid dysfunction on the prognosis of patients remains not yet elucidated. This study aimed to identify the effect of thyroid disorders on outcomes related to COVID-19.

**Patients and methods/materials and methods:** This analytic prospective study was conducted between September 2020 and January 2021. Patients admitted to the ICU with confirmed SARS-CoV-2 pneumonia who underwent thyroid function test on admission were included. The diagnosis of Covid-19 was performed with reverse-transcription polymerase chain reaction (RT-PCR). Patients with pre-existing thyroid problems were excluded. Two groups were defined: group 1: euthyroid patients, group 2: those with thyroid dysfunction. Thyroid disorders involve hypothyroidism and hyperthyroidism (peripheral and central). Primary outcome was ICU mortality. Secondary outcomes were duration of invasive mechanical ventilation, ICU length of stay, ventilation requirement and nosocomial infection. A logistic regression analysis was used to identify if thyroid dysfunction was associated with mortality.

**Results:** A total of 68 patients were included. The most common co-morbidities were hypertension in 48.5% and diabetes mellitus in 36.8% of cases. Mean SOFA score and APACHEII score were, respectively, 4.3 ± 2.5 and 11.8 ± 5.7. Thyroid dysfunction was found on 27.9% of included patients. Out of these, 23.5% had peripheral hyperthyroidism, 2.9% had peripheral hypothyroidism, and 1.5% had central hyperthyroidism. Overall ICU mortality was 45.6%. The characteristics of both groups were comparable (age, sex, and comorbidities). Patients having thyroid dysfunction were more likely to require invasive mechanical ventilation: 73% vs. 45.8%, *p* = 0.03 and to present nosocomial infection: 73.6% vs. 38.7%, *p* = 0.01. There were no significant differences in duration of mechanical ventilation [16 ± 11 vs 10 ± 7 days, *p* = 0.23] and ICU length of stay [19 ± 13 vs 15 ± 9 days, *p* = 0.1]. In the regression multivariate analysis, thyroid dysfunction was not associated with mortality (OR = 1.9; 95% IC [0.3–10], *p* = 0.43). Mechanical ventilation (OR = 13; 95% IC [4.2–23.1], *p* < 0.001) and septic shock (OR = 9.2; 95% IC [6.8–21.2], *p* = 0.003) were found to be associated with mortality.

**Conclusion:** In the current study, we found that patients with thyroid dysfunction were more likely to require invasive ventilation and to present nosocomial infection.

**Compliance with ethics regulations:** Yes in clinical research.

### FC-007 Obesity’s influence on mortality in patients with COVID-19 pneumonia: myth or reality?

#### Rihab Rajah^1^, Fatma Essafi^1,2^, Imen Talik^1,2^, Khaoula Ben Ismail^1,2^, Moez Kaddour^1,2^, Takoua Merhabene^1,2^

##### ^1^Intensive care unit, Regional Hospital, Zaghouan, Tunisie; ^2^Faculty of Medicine of Tunis, University Tunis El Manar, Tunis, Tunisie

**Correspondence:** Fatma Essafi - fatma.essafi@fmt.utm.tn

*Annals of Intensive Care* 2021, **11(Suppl 1):**FC-007

**Rationale:** Many observational studies have suggested that obesity confers an increased risk of morbidity–mortality in the general population and may compromise prognosis in critically ill patients. The aim of our study was to determine whether obesity is a risk factor for poor outcome in patients affected by COVID-19 admitted to ICU.

**Patients and methods/materials and methods:** This study was performed at the Zaghouan’s hospital ICU, a 10-bed secondary ICU in Tunisia. It was a retrospective study enrolled between 1st March 2020 and 31st January 2021 in patients with confirmed RT-PCR Covid-19. Body mass index (BMI) was used as indicator for obesity status. Simplified Acute Physiology Score (SAPS II), Acute Physiology and Chronic Health Evaluation II (APACHE II) scores, comorbidities, invasive mechanical ventilation requirement, ICU length of stay and mortality were evaluated. Patients were divided into two groups: G1: obese, BMI ≥ 30 kg/m^2^ and G2: non-obese, BMI < 30 kg/m^2^.

**Results:** During the study period, 121 patients with COVID-19 pneumonia were admitted. A total of 79 patients (65.3%) with BMI-defined obesity were included in G1, and 42 (34.7%) were assigned to G2. Median age was 62.3 [34–92] years in G1, and 63 [37–92] in G2. Demographic data and comorbidities (sex ratio, SAPS II, APACHE II, hypertension, diabetes, chronic respiratory failure) were similar between the 2 groups. There was no significant difference between PaO2/FiO2 ratio at admission between group 1: 155 ± 107.4 mmHg [47–328] and group 2: 155.7 ± 73 [64–360], *p* = 0.97. At admission, non-invasive mechanical ventilation was prescribed in 70 patients (88.6%) in group 1, and in 33 (78.6%) in G2 with *p* = 0.14. Obesity does not represent limiting factor for awake prone position: instead the obese patients were more adherent to the prone position in spontaneous ventilation (68.4% vs 50%; *p* = 0.048). No adverse effects as pressure ulcer were observed. Length of ICU stay was similar in two groups (9.8 ± 5.7 day [2–30] vs 9.5 ± 5.7 day [2–23]). No significant differences were observed in need to invasive mechanical ventilation (39.2% vs 45.2%; *p* = 0.5) and mortality (39.2% vs 52.4%; *p* = 0.16).

**Conclusion:** Obesity conferred an increased risk for intensive care unit admission, but it did not seem to be associated with increased risk for need for invasive mechanical ventilation, or influence ICU length of stay and/or mortality.

**Compliance with ethics regulations:** N/A.

### FC-008 Evolution of the nutritional status of COVID-19 critically ill patients: a prospective monocentric observational study from ICU admission to 3 months after ICU discharge

#### Claire Rives-Lange^1^, Aymie Zimmer^1^, Amel Merazka^1^, Claire Carette^1^, Anissa Martins-Bexinga^1^, Caroline Hauw-Berlemont^1^, Anne-Sophie Jannot^1^, Jean-Luc Diehl^1^, Sébastien Czernichow^1,2^, Bertrand Hermann^1,3^

##### ^1^Hôpital Européen Georges Pompidou, APHP, Paris, France; ^2^Innovative Therapies in Haemostasis, INSERM, F-75006 Paris, France and Biosurgical Research Lab (Carpentier Foundation), Paris, France; ^3^Institut du Cerveau et de la Moelle épinière - ICM, Inserm U1127, CNRS UMR 7225, F-75013, Paris, France

**Correspondence:** Bertrand Hermann - bertrand.hermann@aphp.fr

*Annals of Intensive Care* 2021, **11(Suppl 1):**FC-008

**Rationale:** Malnutrition following intensive care unit (ICU) stay is frequent and could be especially prominent in critically ill Coronavirus Disease 19 (COVID-19) patients as they present prolonged inflammatory state and long length stay. We aimed to determine the prevalence of malnutrition in critically ill COVID-19 patients both at the acute and recovery phases of infection.

**Patients and methods/materials and methods:** We conducted a prospective observational study including critically ill COVID-19 patients requiring invasive mechanical ventilation discharged alive from a medical ICU of a university hospital. We collected demographic, anthropometric and ICU stay data (SAPS2, recourse to organ support and daily energy intake). Nutritional status and nutritional support were collected at 1 month after ICU discharge (M1) by phone interview and at 3 months after ICU discharge (M3) during a specialized and dedicated consultation conducted by a dietitian. Malnutrition diagnosis was based on weight loss and body mass index (BMI) criteria following the Global Leadership Initiative on Malnutrition. Primary outcome was the prevalence of malnutrition at M3 and secondary outcomes were the evolution of nutritional status from ICU admission to M3 and factors associated with malnutrition at M3.

**Results:** From March 13th to May 15th, 2020, 38 patients were discharged alive from the ICU, median [IQR] age 66 [59–72] years, BMI 27.8 [25.5–30.7] kg/m^2^ and SAPS2 47 [35–55]. Thirty-three (86%) patients were followed up to M3. Prevalence of malnutrition increased during the ICU stay, from 18% at ICU admission to 79% at ICU discharge and then decreased to 71% at M1 and 53% at M3. Severe malnutrition prevailed at ICU discharge with a prevalence of 55% decreasing to 45% at M1 and 32% at M3 at the benefit of moderate malnutrition (Fig.). At M3, the only factors associated with malnutrition in univariate analysis were the length of invasive mechanical ventilation and length of ICU stay (25 [16, 39] vs. 11 [9, 12] days, *P* = 0.006 and 28 [21, 42] vs. 15 [11, 17] days, *P* = 0.010, respectively), while no ICU preadmission and admission factors, nor energy and protein intakes distinguished the two groups. Only 32% of undernourished patients at M3 had benefited from a nutritional support.

**Conclusion:** Malnutrition is frequent, protracted and probably under-recognized among critically ill Covid-19 patients requiring invasive mechanical ventilation with more than half patients still being undernourished 3 months after ICU discharge. A particular attention should be payed to the nutritional status of critically ill Covid-19 patients not only during their ICU stay, but also following ICU discharge.

**Compliance with ethics regulations:** Yes in clinical research.
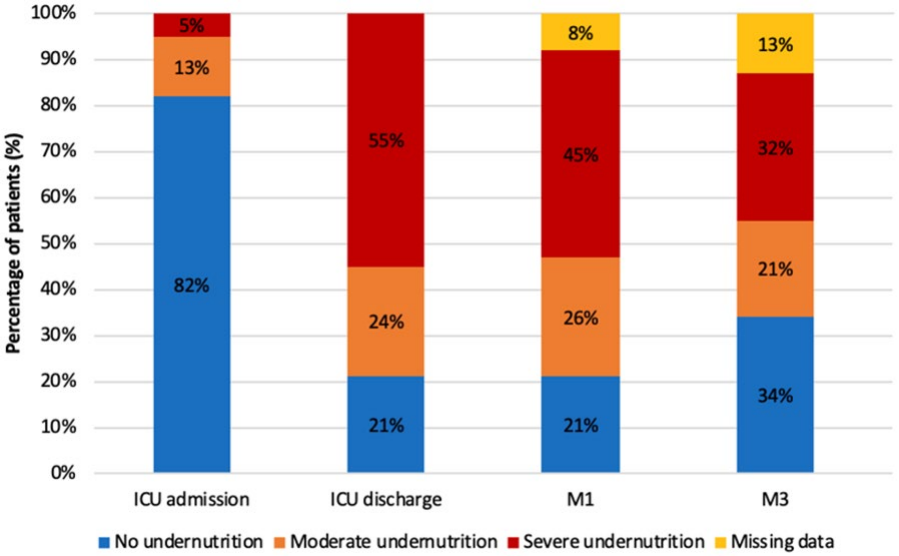


Nutritional status of patients from ICU admission to 3 months post-ICU discharge nutritional status at ICU admission, ICU discharge, 1 month (M1) and 3 months (M3) following ICU discharge was determined by body mass index (kg/m2) and weight loss

### FC-009 Impact of delayed transit in severe COVID 19 patients: a retrospective analysis

#### Dominique Prat^1^, Frédéric Jacobs^1^, Olfa Hamzaoui^1^, Marta Luperto^1^, Karim Toumert^1^, Benjamin Sztrymf^1^

##### ^1^Hôpital Antoine Béclère, Clamart, France

**Correspondence:** Benjamin Sztrymf - benjamin.sztrymf@aphp.fr

*Annals of Intensive Care* 2021, **11(Suppl 1):**FC-009

**Rationale:** Lower gastro-intestinal (GI) tract dysfunction has been proven to be associated with relevant outcomes such as mortality or time spent on mechanical ventilation (MV) in intensive care unit (ICU) patients. A significant subset of COVID patients harbor GI symptoms, whose prognostic significance remain elusive. No studies have characterized the transit of severe COVID ICU patients, and its prognostic value is unknown.

**Patients and methods/materials and methods:** Single-centre, retrospective analysis of all COVID patients admitted in a 12-bed ICU, between February and December 2020. Demographic data, time spent under MV, treatments, transit and survival were compared according to occurrence of delayed transit, as defined by a delay of 6 days or more without stool passage [1]. Early and late delayed transit were defined as occurring, respectively, in the days following ICU admission or later during the ICU stay.

**Results:** 51 patients were included, age 67.6 [56.3–73] years, 40 men, SAPS II 34 [24–42]. At least one baseline comorbidity was found in 21 patients, 15 had diabetes, 27 had systemic hypertension and 20 were obese. Seven patients exhibited diarrhea before admission, without outcome impact. Overall, 36 patients were intubated and received sedation and norepinephrine. Neuromuscular blocking agents (NMBAs) were given to 34 patients. Enteral nutrition was provided to 34 patients, subsequently stopped for 20 of them, mainly for vomiting (*n* = 15). Delayed transit was evidenced in 35 (68.6%) patients (30 of whom were under MV) with a significant association with survival (21/35 vs.4/16; *p* = 0.033), time spent under MV (20[16–32] vs. 6.5[6–16] days; *p* = 0.004) and LOS (20[14–32.8] vs. 9.5[6.5–15.5] days; *p* = 0.002). In univariate analysis (logistic regression) renal replacement therapy, minimal PaO2/FiO2, time spent under NMBAs, parenteral nutrition and percentage of days with stool passage were associated with outcome in MV patients. In multivariate analysis, only minimal PaO2/FiO2 was associated with survival. In ventilated patients with delayed transit (*n* = 30), LOS was longer in patients in late vs. early subgroup (31.5[20–43] vs. 19.5[15–32] days, *p* = 0.04). A trend in a higher time spent under MV was found as well (28.5[19–43] vs. 19[14–32] days, *p* = 0.09). No such difference was found in patients without delayed transit.

**Conclusion:** A high incidence of delayed transit was found in COVID patients. Delayed transit was associated with invasive MV, catecholamine and NMBAs. Delayed transit did not appear to be an independent risk factor for survival. Late, rather than early delayed transit was associated with worse outcomes, raising the question of prophylactic management in this subset of patients.

**Reference**

1. Prat D, Messika J, Millereux M, Gouezel C, Hamzaoui O, Demars N, Jacobs F, Trouiller P, Ricard JD, Sztrymf B. Constipation in critical care patients: both timing and duration matter. Eur J Gastroenterol Hepatol 2018;30:1003–8.

**Compliance with ethics regulations:** Yes in clinical research.

### FC-010 Before–after study: evaluation of the implementation of a nutrition protocol in the intensive care unit

#### Fanny Le Gall^1^, Marjolaine Vallee^1^, Michel Ramakers^1^, Damien Du Cheyron^2^

##### ^1^CH SAINT LO, Saint Lo, France; ^2^CHU CAEN, Caen, France

**Correspondence:** Fanny Le Gall - fanny.legall@ch-stlo.fr

*Annals of Intensive Care* 2021, **11(Suppl 1):**FC-010

**Rationale:** Nutrition has become an essential part of the standard care of patients admitted to the Intensive Care Units (ICU). The main goal of this adjunctive therapy is to reduce impairment of nutrition status induced by “aggression” and ICU stay. The objective of the study is to assess the effect of the implementation of a nutrition protocol on the caloric and protein intakes received by critically ill patients, according to recent expert French guidelines.

**Patients and methods/materials and methods:** In a monocentric before–after study, we compared the implementation of a nutrition protocol in a general intensive care unit. The “before” phase was performed during 3 months at the beginning of 2018 and the “after” phase during a period of a same duration in 2019. Nutrition protocol was introduced to the medical staff at the end of 2018. All adult patients admitted for 3 days or more in the ICU were included. One hundred and seven patients and 72 patients were included in the “before” and “after” groups, respectively. The calorie targets were based on the recommendations of the Société de Réanimation de Langue Française published in 2014. The nutritional support was mixed, oral and/or enteral and/or parenteral. Calorie and protein intakes among both periods were compared at day 0, day 3 and day 7. The efficiency and safety of the protocol was assessed indirectly by insulin therapy-induced glycemic control and phosphoremia repeated measurements.

**Results:** Following the protocol implementation, calorie intakes increased from 159 to 525 kCal/day (*p* < 0.0001) at day 0 and 584 to 853 kCal/day (*p* = 0.007) at day 3. Similarly, protein intakes were improved among periods at day 0 and day 3, respectively. This difference between groups was no more observed at day 7. At day 7, only 33.4% of the patients had a delivered calories/maximum expected calories ratio greater than 75%. No adverse events were observed during the “after” period. There was no effect of the protocol on the main outcome of the patients.

**Conclusion:** Our study confirms that implementation of a nutrition protocol according to recent guidelines allows early improvement of the caloric and protidic intakes in critically ill patients with safety. Better adhesion to protocol from the team is, however, necessary to target maximum expected calorie intake in all patients.

**References**

1. Hurel H, Lefranc J-Y, Cano NJ. Guidelines for nutrition support in critically ill patients. Réanimation. 2014; 23:332–50
2. McClave SA, Taylor BE Guidelines for the provision and assessment of nutrition support therapy in the adult critically ill patient. A.S.P.E.N. 2016; 2: 159–211

**Compliance with ethics regulations:** Yes in clinical research.
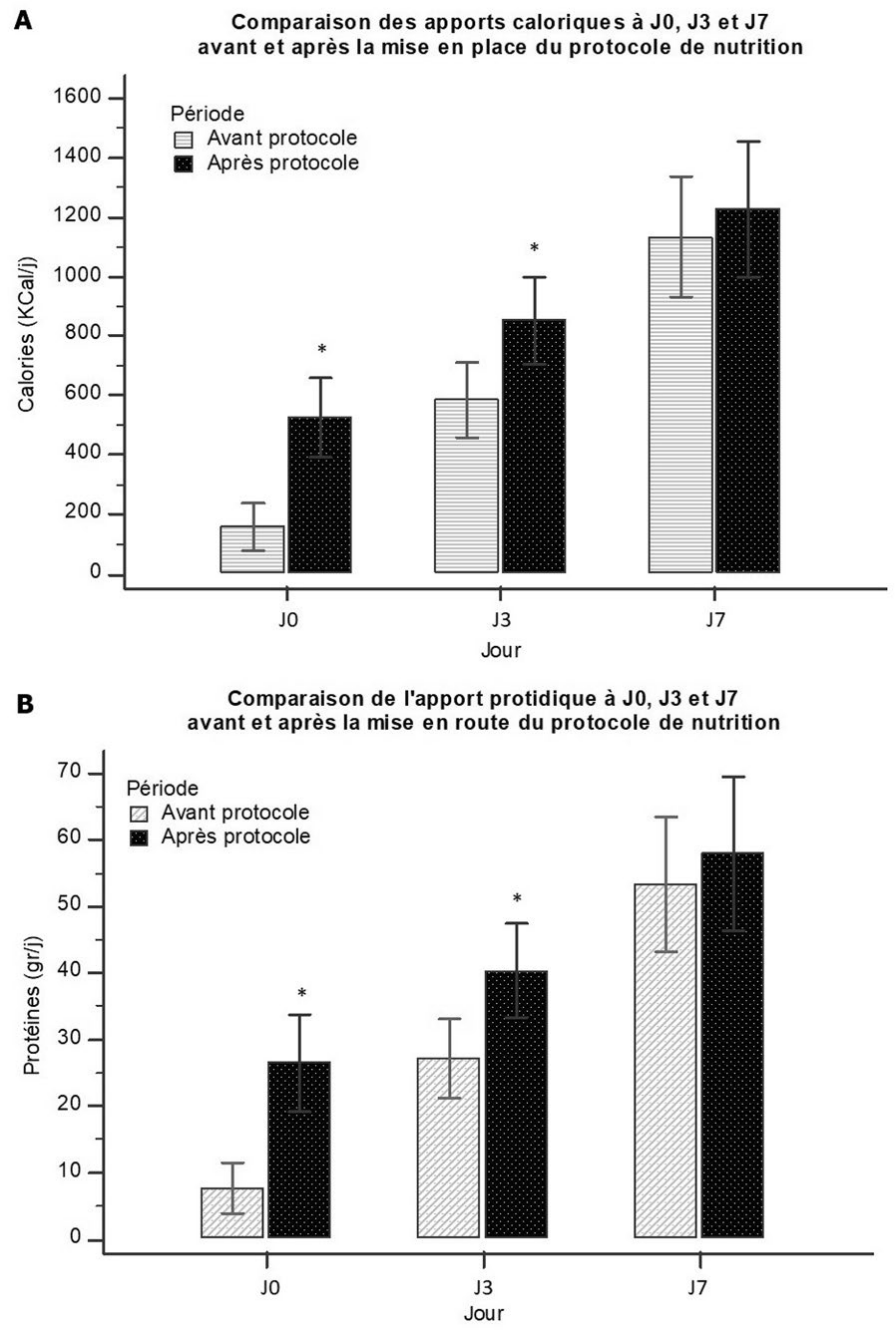


Calorie and protein intake on Day 0, 3 and 7 before and after implementation of the nutrition protocol

### FC-011 Prealbumin is a predictor of mortality at D30 in intensive care patients 75 years and older: a 4-year retrospective study

#### Elodie Edwige^1^, Gilles Albrand^1^, Thomas Tannou^2^, Régis Aubry^2^, Thomas Gilbert^1^, Claire Falandry^1^, Marc Bonnefoy^1^, Pierre Krolak-Salmon^1^, Vincent Piriou^1^

##### ^1^Hospices Civils de Lyon, Pierre-Benite, France; ^2^CHU de Besançon, Besançon, France

**Correspondence:** Elodie Edwige - elodie.edwige@chu-lyon.fr

*Annals of Intensive Care* 2021, **11(Suppl 1):**FC-011

**Rationale:** Prealbumin is an early marker of malnutrition. ICU and post-ICU mortality rates are higher in elderly patients than in younger populations. Age itself explains only a small part of the increased hospital mortality, suggesting that specific information such as nutritional status should be collected to predict mortality in elderly ICU patients. The main objective of this study was to determine if prealbumin is a predictor of 30-day mortality of patients aged 75 years and older admitted in intensive care for at least 24 h, regardless of the reason for admission.

**Patients and methods/materials and methods:** In this monocentric retrospective cohort study, we reviewed the medical charts of patients aged 75 years and over, hospitalized in intensive care (Hospices Civils de Lyon, Groupement Sud, France) for at least 24 h, from 1st November 2015 to 31 December 2019. The data collected were: age, sex, prealbumin, prothrombin, diagnosis of infection, initial IGSII severity score, length of stay (LOS), and 30-day mortality. Exclusion criteria were unmeasured prealbumin, missing initial severity score, re-hospitalization or ICU < 24 h. Mortality at D30 was defined as death occurring within 30 days of admission to ICU.

**Results:** From a total of 1306 admissions, 253 patients (19%) fulfilled the study criteria. The rate of 30-day mortality was 23.3%, 59 patients. Mortality at D30 within the intensive care unit was significantly associated with prealbumin (*p* = 0.009). In the multivariate model, the different risk factors for death were: infection (OR = 2.81 [1.19;7.53] *p* = 0.025) and IGS2 (OR = 1.35 [1.02;1.05] *p* < 0.001). A prealbumin level between 0.1 and 0.2 g/L was a protective factor (OR = 0.39 [0.19;0.80]) *p* = 0.010]. We observed a difference in survival according to the rate of prealbumin: prealbumin levels > 0.20 g/L increased probability of survival at D30: 3 days (logrank *p* value = 0.0032). Comparison of ROC curves shows that IGS II has better diagnostic performance than prealbumin (Fig. 1).

**Conclusion:** Our study showed that hypoprealbuminemia is a risk factor for death. Nevertheless, the hypoprealbuminemia is not the best predictor of mortality at D30. Hypoprealbuminemia is possibly a marker of frailty in patients aged 75 years and older.

**Compliance with ethics regulations:** Yes in clinical research.
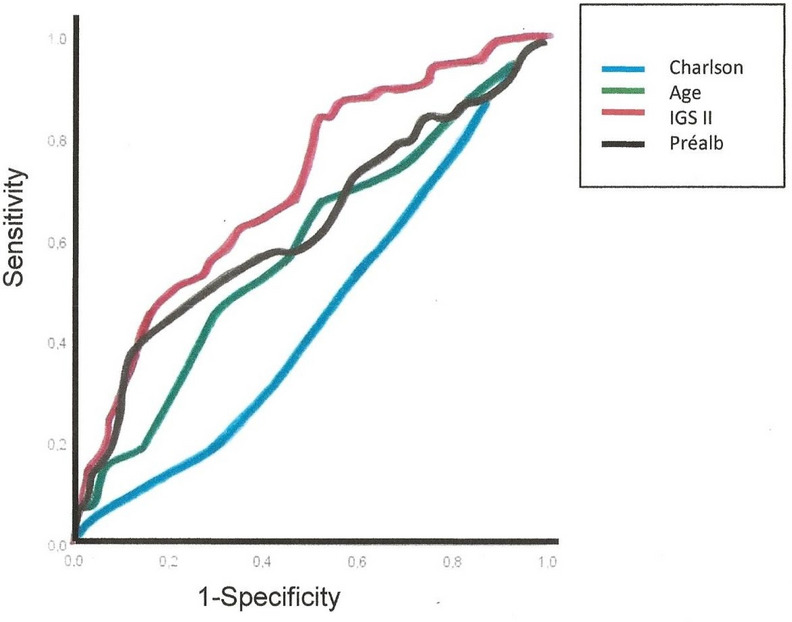


Prognostic accuracy of prealbumin to predict 30-day mortality in comparison to IGS II, age, Charlson

### FC-012 Therapeutic adequacy in abdominal sepsis (about 134 cases)

#### Khalid Khaleq, Wadii Khaya, Aziz Bouhouri, Driss Hamoudi, Rachid Harrar, Khalid Hattabi

##### CHUIbn Rochd Casablanca, Casablanca, Maroc

**Correspondence:** Khalid Khaleq - khaleq 20@gmx.fr

*Annals of Intensive Care* 2021, **11(Suppl 1):**FC-012

**Rationale:** Abdominal sepsis is one of the most frequent digestive emergencies and one of the leading causes of septic shock, involving the life-threatening prognosis of patients who are often elderly or who have underlying pathologies. The management of abdominal sepsis is multidisciplinary. The objective of our study is to describe the epidemiological, clinical, bacteriological and evolutionary data of abdominal sepsis, and to assess the predictive factors of mortality as well as the role of therapeutic de-escalation in the improvement of the vital prognosis.

**Patients and methods/materials and methods:** We carried out a retrospective descriptive and analytical study spanning 3 years (between January 2017 and December 2019) on 134 cases of abdominal sepsis, hospitalized in the intensive care unit of the P33 surgical emergencies of CHU Ibn Rochd Casablanca. Adult patients with community-based or postoperative abdominal sepsis who received medico-surgical treatment were included in our study. The parameters studied are demographic, clinical, radiological, perioperative, bacteriological and evolutionary data. Statistical analysis was performed using SPSS software.

**Results:** The incidence of abdominal sepsis in our work during the study period was 22%, it was divided into community sepsis (69%) and postoperative sepsis (31%). The average age was 52.9 years, with a sex ratio of 1.5. Clinical signs were dominated by abdominal pain (71%), vomiting (51%), extra-abdominal signs (55%), hemodynamic failure (71%), renal failure (63%), and respiratory disorders (41%). Therapeutic management was based on perioperative resuscitation, treatment of organ failure, probabilistic antibiotic therapy and surgery by median laparotomy. The main etiologies of abdominal sepsis were: purulent effusion (36%), serous effusion (17%) and small bowel perforation (15%). Bacteriological samples taken during the operation allowed to have the profile bacteriological: predominance of BGNs (87%) dominated by *E. coli* (29%) followed by *Klebsiella pneumonia* (25%) and *Acinetobacter baumannii* (16.5%). The mean hospital stay was 10.67 days. The death rate was 59%. The main prognostic factors that emerged in our univariate analysis study were: advanced age, diabetes, prior antibiotic therapy, organ failure and development of septic shock. Therapeutic de-escalation was a protective factor

**Conclusion:** Abdominal sepsis is a serious condition with high mortality. The improvement of its prognosis is based on a screening of risk factors, an updating of medico-surgical protocols, a management guided by operators and senior resuscitators and an adapted antibiotic therapy which will depend on the direct examination of the samples, and also on the bacterial ecology of the service.

**Compliance with ethics regulations:** N/A.

### FC-013 Upper gastrointestinal bleeding in adults under veno-arterial extracorporeal membrane oxygenation: a single-center retrospective study

#### Jules Stern, Claire Dupuis, Jang Reuter, Camille Vinclair, Marylou Para, Patrick Nataf, Anne Laure Pelletier, Etienne De Montmollin, Lila Bouadma, Jean Francois Timsit, Romain Sonneville

##### CHU Bichat, Paris, France

**Correspondence:** Jules Stern - julesfresson@gmail.com

*Annals of Intensive Care* 2021, **11(Suppl 1):**FC-013

**Rationale:** Upper gastrointestinal bleeding is a common complication in adults treated with veno-arterial extracorporeal membrane oxygenation (VA-ECMO) for refractory cardiogenic shock or cardiac arrest. We aimed to determine risk factors, prevalence and outcomes associated with upper gastrointestinal bleeding (UGIB) in adult patients under VA-ECMO.

**Patients and methods/materials and methods:** We conducted a retrospective cohort study (2014–2017) on consecutive VA-ECMO patients admitted to the department of intensive care medicine of a university hospital, in Paris, France. UGIB was defined as (1) an overt bleeding (hematemesis, melena, hematochezia), or (2) acute anemia associated with a lesion diagnosed on upper gastrointestinal endoscopy. Cause-specific models were used to identify factors associated with UGIB and death, respectively.

**Results:** 257 patients were included, of whom 48 (19%) patients were diagnosed with UGIB after a median of 18 [7; 43] days following cannulation, median SAPS II was 59 [43; 76]. 100 (39%) patients were implanted after cardiac surgery. Mortality occurred in 121 (58%) patients without UGIB and 31 (65%) patients with. UGIB patients had longer ICU stays (41 [19; 82] vs. 15 [6; 26]; *p* < .01), longer ECMO (10.5 [7; 15] vs 6 [3; 10]; *p* < .01) and mechanical ventilation durations (31 [18; 45] vs. 9 [5; 18]; *p* < .01) in days, as compared to non-UGIB patients. Ninety-nine upper gastrointestinal endoscopy (UGE) were performed and the most frequent endoscopic lesions were gastro-duodenal ulcers (*n* = 28, 28%), leading to 12/99 therapeutic procedures. Antiplatelet therapy prior ICU admission and history of peptic ulcer were not associated with UGIB in univariate analysis. By multivariate analysis (table), a BMI (body mass index) > 30 kg/m^2^ (cause-specific hazard ratio (CSHR) [95% CI]): 3.06 [1.56; 5.98]), and extracorporeal cardiopulmonary resuscitation (ECPR) (CSHR 2.34 [1.03; 5.35]) were independently associated with an increased risk of UGIB.

**Conclusion:** In adult patients under VA-ECMO, obesity and ECPR were independently associated with UGIB. This study highlights the potential role of obesity and acute ischemia reperfusion injury in the pathophysiology of VA-ECMO-associated UGIB.

**Compliance with ethics regulations:** N/A.
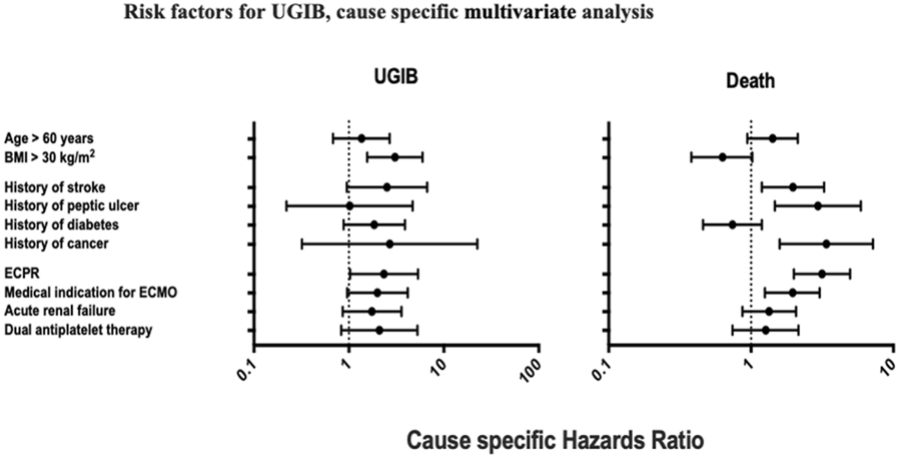


Results are presented as hazard ratio [95% confidence interval] Acute renal failure was defined as a renal SOFA score > 2 UGIB: upper gastrointestinal bleeding; BMI: body mass index, ECMO: extracorporeal membrane oxygenation, SOFA: Sequential Organ Failure

### FC-014 Evaluation of hypercoagulability using viscoelastic testing in critically ill patients with COVID-19

#### Laure Calvet, François Thouy, Anne-Françoise Sapin, Jean-Mathias Liteaudon, Kevin Grapin, Mireille Adda, Claire Dupuis, Bertrand Souweine

##### CHU CLERMONT FERRAND, Clermont Ferrand, France

**Correspondence:** Laure Calvet - lcalvet@chu-clermontferrand.fr

*Annals of Intensive Care* 2021, **11(Suppl 1):**FC-014

**Rationale:** Patients with severe forms of COVID-19 have hypercoagulability, which is associated with arterial or venous thrombotic events. Rotational thromboelastometry (ROTEM) is a laboratory test that analyzes changes in the viscoelastic properties of clot initiation (CT), propagation (CFT), and strength (MCF) in whole blood samples. These parameters may be used to indicate hypercoagulability. The main objective of this observational study is to determine the incidence of hypercoagulability at intensive care unit (ICU) admission using ROTEM results.

**Patients and methods/materials and methods:** This single-center, prospective observational study was carried out in the medical ICU of the university hospital in Clermont-Ferrand, France. All consecutive adult patients with a laboratory confirmed COVID-19 admitted between April 2020 and January 2021 and who did not decline to participate were included. We evaluated coagulation using ROTEM and standard hemostatic tests. ROTEM values suggesting hypercoagulability are CFT below the normal range (NR) in EXTEM (NR = 46 to 149 s) and INTEM (52 to 130 s), MCF above the NR in EXTEM (NR = 55 to 72 mm), INTEM (NR = 51 to 69 mm) and FIBTEM (NR = 6 to 21 mm). Hypercoagulability was ultimately defined by a G-score ((5000 × MCF)/(100-MCF) in EXTEM ≥ 11 dyne/cm^2^ (Taura).

**Results:** Fifty-three patients, with a median (IQR) age of 72.2 [65.8–76.6] years and SAPS2 of 36 [30.6–42] were enrolled in the study; 12 (23%) were administered antiaggregant treatments and 47 (89%) preventive anticoagulant treatments, including reinforced preventive anticoagulation in 34 cases. The median hospital mortality was 30%. The median platelet count, prothrombin level, activated partial thromboplastin time ratio, and serum fibrinogen and D-dimer values were, respectively, 243 G/L [189–346], 83% [76–91], 1.2 [1.2–1.4], 7.4 g/dL [6.8–7.8] and 1355 ng/mL [840–2426]. The median values of EXTEM and INTEM CFT were 42 [36–55], 47 [40–58], respectively, and of EXTEM, INTEM and FIBTEM MCF 73 [69–75], 70 [66–73] and 31 [28–36], respectively; suggesting overally hypercoagulability. Hypercoagulability as defined by a G-score > 11 dyne/cm^2^ was observed in 42 patients; 2 of them presented a pulmonary embolism upon admission.

**Conclusion:** ROTEM results confirm that patients with severe forms of COVID-19 have hypercoagulability on admission. Further investigations are still running to analyze hypercoagulability according to patients’ characteristics, COVID -19 pneumonia specificities, anticoagulant treatments and thromboembolic events.

**Reference**

1. Taura P, Rivas E, Martinez-Palli G, Blasi A, Holguera JC, Balust J, et al. Clinical markers of the hypercoagulable state by rotational thrombelastometry in obese patients submitted to bariatric surgery. Surg Endosc. 2014;28(2):543–51.

**Compliance with ethics regulations:** Yes in clinical research.

### FC-015 Venous and arterial thromboembolic complications in COVID-19 critically ill patients

#### Thomas Janson^1^, Sondes Mhamdi^1^, Florent Bavozet^1^, Kais Mhamdi^1^

##### ^1^Centre Hospitalier Victor Jousselin, Dreux, France

**Correspondence:** Kais Mhamdi - drkaismhamdi@gmail.com

*Annals of Intensive Care* 2021, **11(Suppl 1):**FC-015

**Rationale:** Coronavirus disease 2019 (COVID-19) has been associated with cardiovascular complications and coagulation disorders. Few data are available on the rate and characteristics of thromboembolic complications in ICU patients with COVID-19. The aim of this study was to estimate the prevalence of thromboembolic complication in critically ill patients with COVID-19 and to identify the clinical, radiological and biological characteristics associated with these complications.

**Patients and methods/materials and methods:** We conducted a retrospective study of symptomatic patients with laboratory-proven COVID-19 admitted to ICU (Mars 2020–January 2021). We searched thromboembolic complication including venous thromboembolism (VTE), ischemic stroke, acute coronary syndrome (ACS) or myocardial infarction (MI) and catheter-related thrombosis (CRT). Characteristics of patients, radiological data and anticoagulant treatment were recorded.

**Results:** A total of 82 critically ill patients were studied (median age: 63 years, 58.5% men). The median intensive care unit stay was 13 days (2–80 days). 8 patients have a prior arterial or venous thrombosis event, 16 patients have a history of cancer. Thromboprophylaxis was used in 100% of ICU patients. Therapeutic doses were used in 26 patients. Thromboembolic events occurred in 18 patients. Three patients presented more than one event. The incidence of thromboembolic complication was: distal VTE: 4 cases, proximal VTE: 1 case, pulmonary embolism: 10 cases, CRT: 6 cases, ACS: 2 cases. Parenchymal involvement was noted in 17 patients. Median level of fibrinogen and D-dimers in patients with thromboembolic complication were, respectively, 6 g/L (5.0 e 7.5) and 4 μg/mL (3.2 e 5.6).

**Conclusion:** The high number of arterial and, in particular, venous thromboembolic events diagnosed among the few COVID-19 patients suggest that there is a need to improve specific VTE diagnostic strategies and investigate the efficacy and safety of thromboprophylaxis in critically ill COVID-19 patients. Strict clinical surveillance is needed to detect new events that can occur despite anticoagulation. The prophylactic anticoagulant dose may need to be increased in patients with a low risk of bleeding.

**Compliance with ethics regulations:** N/A.

### FC-016 Effective doses of low-molecular-weight heparin and anti-Xa activity in COVID 19 critically ill patients

#### Feriel Ben Aba, Hamdi Hemdene Doghri, Nadia Zarouane, Ines Sdiri, Imen Zaghdoudi, Youssef Zied El Hechmi, Nebiha BorsalI-Falfoul

##### Université de Tunis El Manar, Faculté de Médecine de Tunis. Hôpital Habib Thameur. Service des urgences et de réanimation médicale, Tunis, Tunisie

**Correspondence:** Hamdi Hemdene Doghri - doghri.hamdi87@gmail.com

*Annals of Intensive Care* 2021, **11(Suppl 1):**FC-016

**Rationale:** High Incidence of thrombo-embolic events is reported in COVID 19 especially in intensive care population. The management of such a prognosis compromising complication is challenging. The objective of our study was to assess the effective dose of LWHM in COVID 19 critically ill patients and identify correlated factors.

**Patients and methods/materials and methods:** Observational, prospective and monocentric study including patients with SARS COV 2 infection admitted in an ICU department between 09/07/2020 and 12/31/2020. Clinical and biological features including anti-Xa (aFXa) assay were reported.

**Results:** 67 patients infected by the SARS COV 2 were included. Sex ratio was 1.57. Age and SAPS II score had a median value, respectively, 64 and 35. Among this population, 50 (74.6%) were treated with therapeutic doses of enoxaparin and 36 had at least one aFXa assay with a median of 2 assays per patient [1–3], and extremes of 1 and 5. Target was reached in 43 aFXa tests and a mean LWHM dose used was about 1.1 mg/kg/dose ± 0.17 with a minimum of 0.74 and a maximum of 1.43. In 29 assay, patients needed higher then 1 mg/kg/dose of enoxaparin to reach target aFXa with a mean dose of 1.21 mg/kg/dose ± 0.12 with a minimum of 1.02 and a maximum of 1.43. The effective dose of LWHM had a positive correlation with the concomitant D-dimer rate and a negative correlation with the patient’s weight. The correlation coefficients are detailed in the table below.

**Conclusion:** Guidelines are recommending to consider higher doses of anticoagulation therapy in patients in ICU. Our study demonstrated two factors that are correlated to the effective dose of LWHM, which are D-dimer rate and patient’s body weight.

**Compliance with ethics regulations:** Yes in clinical research.
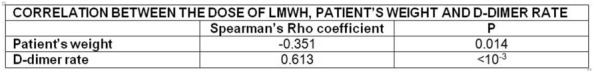


Correlation between the dose of LMWH, patient’s weight and D-dimer rate

### FC-017 Relationship between ABO blood groups and the COVID-19 susceptibility

#### Khaoula Ben Ismail^1^, Boudour Ben Dhia^1^, Najla Ben Slimene^1^, Fatma Essafi^1^, Moez Gaddour^1^, Takoua Merhabene^1^

##### ^1^Hopital regional de Zaghouan, Zaghouan, Tunisie

**Correspondence:** Khaoula Ben Ismail - khaoula87@hotmail.fr

*Annals of Intensive Care* 2021, **11(Suppl 1):**FC-017

**Rationale:** There is currently no biological marker known to predict the susceptibility to COVID-19. ABO blood groups have been implicated as potential risk factors for various diseases. Here, we investigated the relationship between the ABO blood type and the susceptibility to COVID-19.

**Patients and methods/materials and methods:** It was a retrospective study performed at the zaghouan’s hospital ICU, a 10-bed tertiary ICU in Tunisia between 1st September 2020 and 31st January 2021. All adult patients who had ABO blood grouping and had been tested positive to COVID-19 by viral RNA polymerase chain reaction testing were enrolled. Patients were subdivided into four groups based on ABO blood grouping: O, A, B, and AB. The association between blood types and outcome were evaluated using multivariate logistic regression analysis.

**Results:** A total of 126 patients were hospitalized during the study period. Mean age was 62 ± 12 years [34–92] with a sex ratio 1.5. Seventy-nine patients (65.3%) were obese (IMC ≥ 30) and 30.6% were smokers. Mean SAPSII, Charlson and APACHE II scores were 25.9 ± 8 [6–50]; 2.8 ± 1 [0–7]; and 7.7 ± 3 [1–27], respectively. We collected ABO-typed blood samples from 83 patients. Based on ABO blood grouping, 43 patients had blood type A (35.5%), 22 had blood type O (18.2%), 14 patients had blood type B (11.6%) and 4 had blood type AB (3.3%). Our results showed that blood group A was associated with a higher risk for acquiring severe COVID-19 compared with non-A blood groups (odds ratio = 3.26, *p* = 0.009) and higher mortality (OR = 2.26; *p* = 0.005) versus all others, whereas blood group O was associated with a lower risk of mechanical ventilation (OR = 0.81) and acquiring nosocomial infections (OR = 0.64) compared with non-O blood groups.

**Conclusion:** We report an association between the ABO blood group and COVID-19 susceptibility, demonstrating the latter to be a biomarker differentiating the former. Our findings require to be confirmed by further studies.

**Compliance with ethics regulations:** Yes in clinical research.

### FC-018 A descriptive Blood sample Volume Analysis in a Multidisciplinary Pediatric Intensive caRE unit (B-VAMPIRE)

#### Tine François^1^, Michael Sauthier^1^, Julien Charlier^1^, Baruch Toledano^1^, Jessica Dessureault^1^, Sally Al Omar^1^, Jacques Lacroix^1^, Geneviève Du Pont-Thibodeau^1^

##### ^1^CHU Sainte-Justine, Montreal, Canada

**Correspondence:** Tine François - tine.francois1@gmail.com

*Annals of Intensive Care* 2021, **11(Suppl 1):**FC-018

**Rationale:** Anemia is a significant complication in pediatric intensive care units (PICUs). Diagnostic blood testing is a recognized contributor. Yet, little is known about current blood testing practices in PICUs. We aimed to characterize the blood collected for laboratory testing and the transfusion practice in our PICU, and its impact on the incidence of anemia.

**Patients and methods/materials and methods:** We conducted a prospective observational single-center cohort study in children admitted to the PICU of CHU Sainte-Justine between September 2019 and January 2020. Diagnostic blood volumes and volumes discarded were collected throughout the PICU stay. Data collection further included admission diagnoses, length of stay (LOS) in PICU, ventilatory support, vascular access, first and last hemoglobin during PICU stay, and transfusion. Anemia was defined according to the Canadian Blood Services diagnostic criteria.

**Results:** 423 children (mean age 4.9 ± 5.6 years) were enrolled. The most common medical diagnoses were respiratory illness (36.9%), neurological disease (8.3%), intoxication (4.5%), septic state (3.3%), and non-septic shock (4.3%). 33.1% were admitted postoperatively, including 50 (35.2%) following cardiac surgery. The mean LOS was 4.5 days with a mean worst PELOD2 of 4.9 ± 3.9. Invasive and non-invasive ventilation was used in 29.8% and 46.8% of patients, respectively. A total of 31.7% had a central venous line, 28.4% an arterial line. The mean blood volume drawn was 5.3 ± 6.3 mL/patient/day, with a mean volume discarded of 1.1 ± 1.8 mL/patient/day. The total blood loss was 3.9 ± 0.9 mL/kg/stay, corresponding to 5% of total blood volume. Patients with blood-drawing access had more blood sampled compared to patients without (8.2 ± 6.4 vs. 3.1 ± 5.3 mL/patient/day; *p* < 0.0001). Cardiac surgery patients and septic patients were sampled the most (14.3 and 11.0 mL/patient/day, respectively), while the least sampling occurred following otorhinolaryngologic procedures (1.8 mL/patient/day). Mean hemoglobin values were 117 ± 23 g/L and 110 ± 21 g/L at PICU admission and discharge, respectively (*p* < 0.05). Over half of PICU survivors (56.2%) were anemic at discharge, including 16.25% with a hemoglobin < 90 g/L; 10% received ≥ 1 red blood cell transfusion during PICU stay. Multivariate analysis revealed no significant risk factors for a hemoglobin decline during PICU stay, except for a correlation with sampling volumes > 5 ml/kg/stay.

**Conclusion:** Blood sampling is significant in PICU patients, accounting for 5% of children’s total blood volume. Indwelling catheters, sepsis, and cardiac surgery are associated with higher blood loss. Improved blood testing strategies are needed to decrease the incidence of iatrogenic anemia.

**Compliance with ethics regulations:** Yes in clinical research.

### FC-019 Therapeutic plasma exchange in critically ill patients, results of monocentric observational study

#### Claire Bachelier, Laure Calvet, François Thouy, Elisabeth Coupez, Kévin Grapin, Claire Dupuis, Bertrand Souweine

##### CHU Clermont Ferrand, Clermont Ferrand, France

**Correspondence:** Claire Dupuis - cdupuis1@chu-clermontferrand.fr

*Annals of Intensive Care* 2021, **11(Suppl 1):**FC-019

**Rationale:** Data on therapeutic plasma exchange (TPE) in the intensive care unit (ICU) setting are scarce. We aimed to describe indications, practices and outcome of TPE in critically ill patients.

**Patients and methods/materials and methods:** This retrospective study included consecutive patients who underwent TPE between January 2011 and December 2017 in the medical ICU of the university Hospital in Clermont Ferrand France.

**Results:** The study population included 58 patients, who received 314 TPE sessions (median number of 4 [3–5] TPE per patients), with a sex ratio of 1, a median age of 60 years [49; 71], a median SAPS II of 32.5 [22; 45]. On ICU admission a respiratory and renal SOFA subscore ≥ 2 was present in 20 and 29 patients, respectively, and the disease requiring TPE previously diagnosed in 16. The main indications for TPE were: thrombotic microangiopathy (21 patients/149 sessions), including thrombotic thrombocytopenic purpura (5 patients) and hemolytic uremic syndrome (13 patients); neurologic disorders (13 patients/56 sessions), including Guillain–Barré syndrome (9 patients); vasculitis (10 patients/67 sessions), including anti-neutrophil cytoplasmic antibodies-associated vasculitis (9 patients); and hyperviscosity syndromes (10 patients/13 sessions), including hypertriglyceridemia (N patients). TPE was started within the first 24 h of ICU admission in 50 patients. TPE was performed using a dialysis catheter in 54 patients, regional citrate anticoagulation was systematically used, and the TPE technique was centrifugation in 279 (89%) sessions and double filtration plasmapheresis in others. The type of replacement fluid was plasma frozen with or without albumin 5% in 271 sessions and albumin 5% in the others. Twenty-eight (9%) of TPE sessions were reported with at least one adverse event, including mostly allergic reactions (*n* = 8) and bleeding (*n* = 7); hypotension, hypocalcemia and transfusion-related acute lung injury were observed for each one, in one case. The median ICU and hospital length of stays were 6 days [3; 14] and 22 [14; 32], respectively. Forty-eight patients were discharged alive from hospital, including 8 with dialysis dependent renal failure. Among patients with fatal outcome 3 received TPE for vasculitis, 3 for neurologic disorders, 1 for hyperviscosity and 1 for thrombotic microangiopathy.

**Conclusion:** In patients admitted to ICU, TPE can be performed in emergency, is relatively safe and generally well-tolerated. In most cases, the disease indicating TPE was not diagnosed prior to ICU admission. The outcome seems to depend on the etiology of the underlying disease requiring TPE.

**Compliance with ethics regulations:** Yes in clinical research.

### FC-020 PLASMIC and French scores have decreased performance for thrombotic thrombocytopenic purpura prediction when applied to an unbiased cohort of thrombotic microangiopathy patients

#### Nicolas Fage^1^, Nicolas Henry^1,2^, Chloé Mellaza^1,2^, Franck Genevieve^5^, Marie Tuffigo^3^, Corentin Orvain^4^, Jean François Augusto^1^, Benoit Brilland^1^

##### ^1^Service de Néphrologie-Dialyse-Transplantation CHU Angers, Angers, France; ^2^Service de Néphrologie et hémodialyse, CH Laval, Laval, France; ^3^Laboratoire d’Hémostase CHU Angers, Angers, France; ^4^Service des maladies du sang CHU Angers, Angers, France; ^5^Laboratoire d’Hématologie CHU Angers, Angers, France

**Correspondence:** Nicolas Fage - fage.nicolas@gmail.com

*Annals of Intensive Care* 2021, **11(Suppl 1):**FC-020

**Rationale:** The PLASMIC (*1*) and French scores (*2*) have been developed to help clinicians in the early identification of patients with thrombotic thrombocytopenic purpura. However, the validity of these scores in thrombotic microangiopathy cohorts with low thrombotic thrombocytopenic purpura prevalence remains uncertain. Thus, we aimed to evaluate the diagnostic performance of these scores in routine clinical practice using an unbiased cohort of thrombotic microangiopathy patients. We also analyzed the value of proteinuria when added to PLASMIC and French scores for thrombotic thrombocytopenic purpura prediction.

**Patients and methods/materials and methods:** We retrospectively included all consecutive patients between 01/01/2010 and 12/31/2019 with a biological thrombotic microangiopathy syndrome at admission in a tertiary hospital. Thrombotic microangiopathy etiology was retrieved and scores were assessed. Modified scores including proteinuria were compared to original scores for thrombotic thrombocytopenic purpura prediction.

**Results:** Among 273 patients presenting with a full biological TMA syndrome, 238 were classified as having TMA diagnosis after chart review. Data for PLASMIC and French scores were available in 225 patients (94%) with a TTP prevalence of 6.2% (scores cohort). In this population, the area under the curve (AUC) of PLASMIC and French scores for TTP diagnosis were 0.67 (*p* = 0.02) and 0.65 (*p* = 0.06), respectively. Proteinuria at admission was assessed in 134 patients. In this population (modified scores cohort), the AUC of modified PLASMIC score for TTP diagnosis was 0.76 (*p* = 0.006), significatively higher than the standard score (*p* = 0.003) and the AUC of modified French score was 0.81 (*p* = 0.001), tending to be higher than the standard score (*p* = 0.07) (Fig. 1). Specificity and predictive positive value of high-risk modified scores were significantly improved by adding proteinuria.

**Conclusion:** PLASMIC and French scores have low predictive values when applied to an unbiased TMA cohort. Addition of proteinuria to original scores improves their predictive performance for TTP diagnosis.

**References**

1. Bendapudi PK, Hurwitz S, Fry A, Marques MB, Waldo SW, Li A et al. Derivation and external validation of the PLASMIC score for rapid assessment of adults with thrombotic microangiopathies: a cohort study. Lancet Haematol. 2017;4(4):e157–64
2. Coppo P, Schwarzinger M, Buffet M, Wynckel A, Clabault K, Presne C et al. Predictive Features of Severe Acquired ADAMTS13 Deficiency in Idiopathic Thrombotic Microangiopathies: The French TMA Reference Center Experience. PLOS ONE. 2010;5(4):e10208

**Compliance with ethics regulations:** Yes in clinical research.
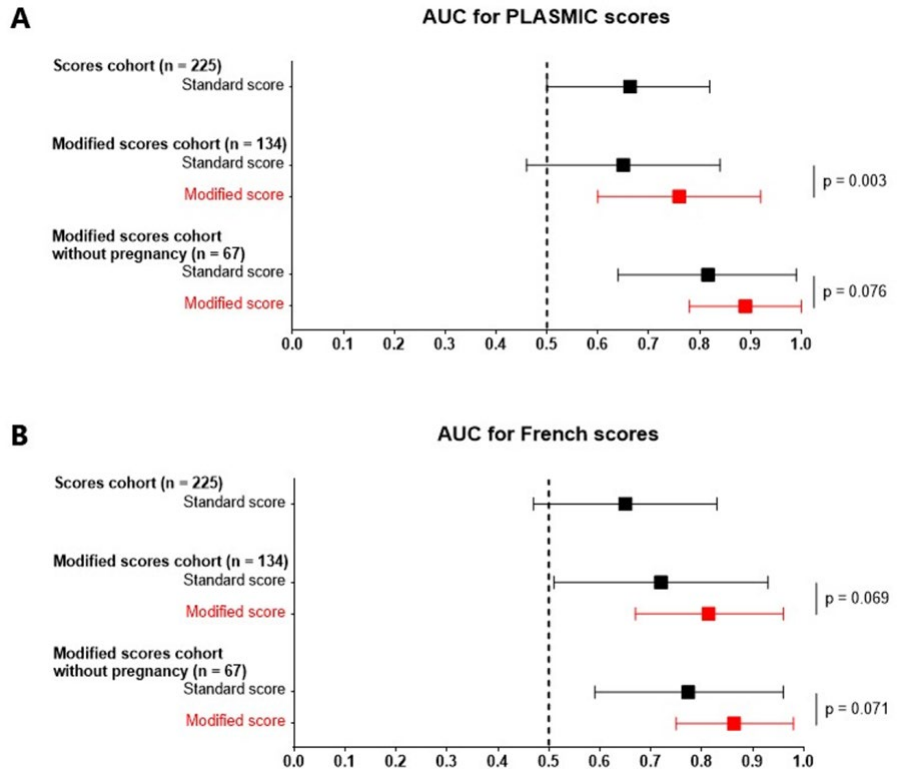


Fig. 1 Contribution of the proteinuria to PLASMIC (**A**) and French (**B**) score to predict the diagnosis of TTP

### FC-021 Evaluation of the performance of the Arctic Sun^®^ external cooling device for controlling fever in septic shock patients

#### Marie-Claire Diemoz, Tin-Hinan Mezdad, Pierre Chaffiotte, Emmanuelle Mercier, Marie Lecronier, Jerome Cecchini, Tommaso Maraffi, Russel Leon, Frédérique Schortgen

##### Hopital Inter Communal de Créteil, Creteil, France

**Correspondence:** Tin-Hinan Mezdad - thmezdad@gmail.com

*Annals of Intensive Care* 2021, **11(Suppl 1):**FC-021

**Rationale:** Fever control by external cooling is proposed to help in controlling shock in septic patients. However, little is known about the optimal method. The aim of this study was to assess the performance of a water-circulating cooling device (Arctic Sun^®^-BD) for controlling fever in patients with septic shock.

**Patients and methods/materials and methods:** We conducted an observational, retrospective, monocentic study, in 16 febrile septic shock patients. Inclusion criteria were fever > 38 °C in patients requiring norepinephrine, mechanical ventilation and sedation. External cooling was applied to achieve fever control with a targeted core temperature at either 36.7 or 38 °C according to physician choice. The oesophageal core temperature recorded every minute was extracted from the Arctic Sun^®^ system. To evaluate the performance of the system, we recorded the time to reach target expressed by cooling rate (°C/h) and the percentage of time at the target ± 0.5 °C calculated from the time of target reached to H24 after cooling start or to the end of therapy, whenever which came first. We assessed whether patient’s characteristics influenced the efficacy of cooling and the association between cooling efficacy and evolution of shock at 24 h. Data are presented as median and 25th–75th interquartile range. Funding of the study: none.

**Results:** Median age was 56 [41–63] years, SAPS II score 41 [26–53], weight 78 [66–98] kg, BMI 28 [25–31]. Ten patients had ARDS. At the time of cooling start the temperature was 38.7° [38.5 °C–39.3 °C], dose of norepinephrine 0.34 [0.2–0.5] µg/kg/min, serum lactate 1.4 [1.2–1.7] mmol/l and RASS score − 5 [− 5–− 4]. During cooling, 12/16 patients received curare and 7 corticoids. One patient never reached the temperature target. Time to reach temperature target was 115 [62–229] min leading for a cooling rate of 1.03 [1.12–0.56] °C/h. The percentage of time at the targeted temperature was 74%. We found no correlation between cooling efficacy and patients characteristics in terms of severity, level of sedation, age, BMI. Cooling rate and time at temperature target were not statistically different between patients with or without curare or corticoids. At H24 norepinephrine could has been stopped in 8/16 patients. We found no correlation between cooling efficacy and shock evolution.

**Conclusion:** We report the first evaluation of the performance of water-circulating external cooling system for controlling fever in septic shock patients. In this limited population, the Arctic Sun^®^ device appears suitable independently from patients characteristics.

**Compliance with ethics regulations:** Yes in clinical research.
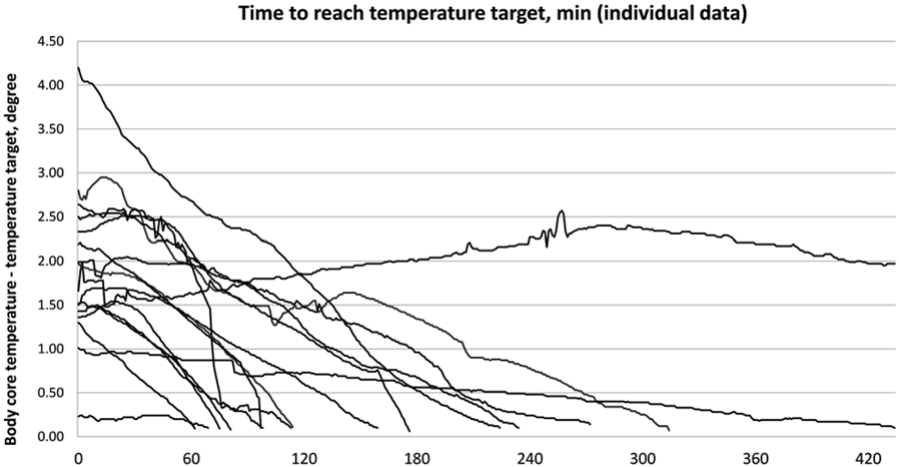


Time to reach temperature target (min)

### FC-022 Identification of potential probiotics candidates for decontamination against ESBL-producing Enterobacteriales faecal carriage by gut microbiota analysis

#### Renaud Prével, Raphaël Enaud, Arthur Orieux, Adrian Camino, Patrick Berger, Alexandre Boyer, Laurence Delhaes, Didier Gruson

##### CHU Bordeaux, Bordeaux, France

**Correspondence:** Renaud Prével - renaud.prevel@hotmail.fr

*Annals of Intensive Care* 2021, **11(Suppl 1):**FC-022

**Rationale:** Extended spectrum beta-lactamase producing Enterobacteriales (ESBL-E) are the main source of empiric antimicrobial therapy failure in Europe. Patients colonized with ESBL-E are at high risk of subsequent ESBL-E infection. Decolonization has been proposed for these patients, but concerns remain about the selection of antimicrobial resistance under the pressure of antimicrobial compounds. Another approach would be to administrate probiotics for decolonization, but it has been disappointing so far. A major pitfall is that the given micro-organisms were selected based on a poor rationale. We thus aimed to identify potential probiotics candidates comparing the gut microbiota between ESBL-E faecal carriers and non-carriers.

**Patients and methods/materials and methods:** The rectal swab performed for ESBL-E carriage screening at admission of every consecutive patient admitted to ICU between January and March 2019 was collected and frozen at − 80 °C. DNA extraction was performed thanks to QIAamp^®^ PowerFecal^®^ Pro DNA kit (QIAgen^®^). V3–V4 regions of 16SRNA coding gene and ITS2 were amplified by PCR. Sequencing was performed on MiSeq sequencer (Illumina^®^). ESBL-E fecal carriers were matched with the first two consecutive controls of similar age and sex. DADA2 pipeline on R software was used for bioinformatics analyses. Identification of potential probiotics candidates was assessed by LefSe analysis.

**Results:** Among the 255 patients admitted to ICU, 23 (9%) were colonized with an ESBL-E: 11/23 (48%) carried ESBL-producing *E. coli*, and 6/23 (26%) ESBL-producing *K. pneumoniae*. ESBL-producing *E. coli* faecal carriers (*n* = 11) were similar in terms of SAPS2, proportion of patients presenting with septic shock and ARDS, requiring norepinephrine support or invasive mechanical ventilation, ICU- and Day28-mortality rates with the 22 age- and sex-matched controls as were ESBL-producing *K. pneumoniae* faecal carriers (*n* = 6) compared with the 12 age- and sex-matched controls. Microbiota α- and β-diversities were not different between ESBL-producing *E. coli* faecal carriers and non-carriers (*p* = 0.09 and *p* = 0.69, respectively). Interestingly, the presence of *Sellimonas intestinalis* (*Clostridium* cluster XIVa) was found to be associated with the absence of colonization with ESBL-producing *E. coli* (LDA score > 3). Microbiota α-diversity was significantly decreased in ESBL-producing *K. pneumoniae* faecal carriers compared with non-carriers (*p* = 0.05), but β-diversity was not different (*p* = 0.8). Interestingly, *Campylobacter ureolyticus*, *Campylobacter hominis* and bacteria belonging to *Clostridium* cluster XI were associated with the absence of colonization with ESBL-producing *K. pneumoniae* (LDA score > 3).

**Conclusion:** Specific and different bacterial species are associated with the absence of gut colonization with ESBL-producing *E. coli* or *K. pneumoniae*. These species could be potential probiotics candidates for “tailored decontamination”.

**Compliance with ethics regulations:** Yes in clinical research.

### FC-023 Strategy towards antibiotic stewardship: ICU cultures in 2017

#### Alexander Verheggen, Marc Bourgeois, Eric Nulens

##### AZ Sint-Jan Brugge, Bruges, Belgique

**Correspondence:** Alexander Verheggen - alexander_verheggen@hotmail.com

*Annals of Intensive Care* 2021, **11(Suppl 1):**FC-023

**Rationale:** In current times of the increasing multidrug-resistant (MDR) organisms in intensive care units and the rising awareness of cost-effectiveness, implementing strategies towards antibiotic stewardship is imperative. Therefore, current habits should be evaluated prior to initiating new protocols.

**Patients and methods/materials and methods:** This study was retrospective and was conducted in the intensive care unit of a non-university hospital, AZ Sint-Jan, in Bruges, Belgium. We evaluated all cultures taken from patients in 2017. A total of 3529 samples of 340 different patients were investigated: 1789 endotracheal aspirates (50.69%), 773 urines (21.90%), 469 bronchial aspirates (13.29%), 183 hemocultures (5.19%), 180 sputums (5.10%), 101 bronchoalveolar lavages (2.86%) and 34 central venous catheters (0.96%).

**Results:** Patients ranged in age from 12 to 92 years; 62% were male and 38% female. 91.8% of the hemocultures were positive, mainly *Staphylococcus spp.* (24.0%), followed by *Pseudomonas aeruginosa* (18.6%), *Enterococcus spp.* (14.21%), *Escherichia coli* (7.65%) and *Klebsiella spp*. (6.56%). 1349 superficial respiratory tract samples were positive (68.51%): 1/3 *Pseudomonas aeruginosa* (31.13%), 13.57% *Klebsiella spp.* and 10.90% *Enterobacter spp.* and 10.67% *Staphylococcus aureus*. 404 deep respiratory samples were positive (70.88%): 1/3 *Pseudomonas aeruginosa* (28.71%) 13.86% *Klebsiella spp.* and 12.62% *Staphylococcus aureus*. Urine was positive in 41.79%, mainly *Enterococcus spp.* (29.10%). 50% of central venous catheters grew bacteria: 20.58% *Staphylococcus epidermidis*, 17.65% *Pseudomonas aeruginosa*. Incidence of MDR was low in all.

**Conclusion:** Retrospective evaluation of cultures is the first step towards implementing strategies for antibiotic stewardship. It is necessary to obtain knowledge of the intrahospital ‘microbioma’ to adapt empiric therapy as well as to have a clear sight of patterns of MDR organisms.

**Reference**

1. Pickens CI, Wunderink RG. Principles and Practice of Antibiotic Stewardship in the ICU. Chest 2019; 156(1): 163–71.

**Compliance with ethics regulations:** Yes in clinical research.

### FC-024 Effect of sepsis on platelet function analyzer (PFA)-100 closure time: a prospective observational study

#### Anis Chaari, Ghada Al Harbi, Dermot Cox, Kamel Bousselmi

##### King Hamad University Hospital, Muharaq, Bahrein

**Correspondence:** Anis Chaari - anischaari2004@yahoo.fr

*Annals of Intensive Care* 2021, **11(Suppl 1):**FC-024

**Rationale:** Platelet function analyser (PFA)-100 closure time is a useful tool to assess both primary and acquired platelet dysfunction. The effect of sepsis on this parameter has not been assessed previously.

**Patients and methods/materials and methods:** Prospective study conducted in the critical care department of King Hamad University hospital (Bahrain). All patients admitted with sepsis or septic shock were included. Patients with known hematologic malignancies, known platelet disease or taking any anti-platelet medication were excluded. A group of 20 healthy volunteers were also included. Closure time after platelet exposure to collagen/epinephrine (CEPI) and collagen/adenosine diphosphate (CADP) was measured on admission.

**Results:** Fifty-seven patients were included. Median[Quartile] age was 63[54–76] years. Median APACHE-II score was 20 [14–27]. Sex ratio was 0.8. Platelet count was similar between septic and control groups (respectively, 169.8 [93.4–258.7] vs. 181.5 [148.8–225] G/L; *p* = 0.45). Both groups had similar closure time after exposure to CEPI (respectively, 137 [111.5–207.3] and 151 [118.3–182] s; *p* = 0.996), whereas septic patients had more prolonged CT after exposure to CADP (125 [98–189.5] vs. 106.5 [88.3–132] s; *p* < 0.001). In septic patients, CT was comparable between patients with sepsis and those with septic shock (respectively, 125.5 [99.8–185.8] vs. 167[117.5–221.8] s; *p *= 0.173 for CEPI and 106.5 [96–182.8] vs. 147[102.5–208] s for CADP; *p *= 0.169). Similarly, CT was comparable between survivors and non-survivors in septic patients (respectively, 130[103.8–197.8] vs. 182[134.3–239] s for CEPI; *p *= 0.153 and 120[99.5–182.8] vs. 138[92–214] s for CADP; *p *= 0.861).

**Conclusion:** Sepsis is associated with prolonged CT following exposure to CADP suggesting platelets dysfunction and decreased platelet reactivity in comparison to healthy volunteers. However, sepsis severity and the outcome were not associated to any significant changes in closure time.

**Compliance with ethics regulations:** Yes in clinical research

### FC-025 Immune suppression is associated with enhanced systemic inflammatory, endothelial and procoagulant responses in critically ill patients

#### Fabrice Uhel^1,2^, Xanthe Brands^2^, Lonneke Van Vught^2^, Maryse Wiewel^2^, Arie Hoogendijk^2^, René Lutter^2^, Marcus Schultz^2,3,4^, Brendon Scicluna^2^, Tom Van Der Poll^2^

##### ^1^Hôpital Louis Mourier, Colombes, France; ^2^Academic Medical Center, Amsterdam, Pays-Bas; ^3^Mahidol Oxford Tropical Medicine Research Unit, Bangkok, Thailande; ^4^University of Oxford, Oxford, Royaume-Uni

**Correspondence:** Fabrice Uhel - fabrice.uhel@aphp.fr

*Annals of Intensive Care* 2021, **11(Suppl 1):**FC-025

**Rationale:** Critical illness is associated with immune suppression and administration of immune stimulatory agents has been suggested as a novel therapeutic approach in patients showing immunological evidence of this host response aberration. We here tested the hypothesis that the extent of immune suppression is proportional to the degree of systemic hyperinflammation in critically ill patients.

**Patients and methods/materials and methods:** 77 consecutive critically ill patients admitted to the intensive care unit (ICU) in a tertiary hospital in the Netherlands. Blood was collected on the first morning after ICU admission. Leukocytes were stimulated with lipopolysaccharide (LPS) and cytokines were measured in supernatants.

**Results:** Patients were stratified into four groups of increasing immune suppression based on quartiles of LPS-induced tumor necrosis factor (TNF)-α production (from normal to severely reduced). Reduction in TNF-α production capacity was associated with a proportionally reduced release of IL-1β and IL-6 upon stimulation with LPS. Measurements of 15 plasma biomarkers reflecting major pathways involved in the pathogenesis of critical illness showed that the extent of impairment of TNF-α production by blood leukocytes was associated with enhanced systemic inflammatory responses, enhanced endothelial cell activation, loss of vascular integrity and increased procoagulant responses.

**Conclusion:** Critically ill patients with the strongest immunosuppression (lowest blood leukocyte TNF-α production capacity) concurrently show stronger signs of systemic inflammatory responses. These results are relevant for the development of precision medicine in critical care and selection of patients for treatment with immune stimulatory agents.

**Compliance with ethics regulations:** Yes in clinical research

### FC-026 The contribution of (PvCO2-PaCO2)/(CaO2-CvO2) ratio level as an indicator of anaerobic metabolism variation in patients with septic shock

#### Walid Sellami, Ines Ben Mrad, Iheb Labbene, Mustapha Ferjani

##### Department of critical care medicine and anesthesiology, Military Hospital of Tunis, Tunisia, Tunis, Tunisie

**Correspondence:** Walid Sellami - drsellamiwalid@yahoo.fr

*Annals of Intensive Care* 2021, **11(Suppl 1):**FC-026

**Rationale:** Blood lactate level in patient with septic shock is dependent on various factors and can be difficult to interpret. A ratio (PvCO2-PaCO2)/(CaO2-CvO2) (hereafter termed “Ratio”) superior to 1.4 was described as parameter of anaerobic metabolism’s evaluation. The aim of this study was to describe the correlation between fast hemodynamic status’ variation and the Ratio and if this parameter is related to blood lactate level in patients with septic shock.

**Patients and methods/materials and methods:** We included all patients with septic shock who are intubated, ventilated, sedated, with stable hemodynamic status requiring noradrenalin and with cardiac catheterization. Norepinephrine infusion was reduced in patients who had PAM greater than 85 mmHg under norepinephrine, targeting a PAM at 65 mmHg. Cardiac output was measured with heart ultrasound. Hemoglobin, arterial, venous blood gases were measured before and after norepinephrine modifications.

**Results:** Thirty patients with documented septic shock were included. The mean age of patients was 60 [20–82] years. Blood lactate levels was 3.5 [2–8.5] mmol/L. Twenty-three patients had an elevated Ratio at 1.4. The falling of PAM resulted in the rise of the Ratio from 6% to 130% in 20 cases and the falling of the Ratio between 40% and 10% in 10 cases. In 26 patients, blood lactate level remained unchanged while 23 patients had a Ratio variation from 6% to 130%. The absence of Ratio variation was always associated with an absence of lactate level variation. There was no correlation between variation of the Ratio and variation of cardiac output.

**Conclusion:** Hemodynamic status’ modification in patient with septic shock under norepinephrine can result in modification of anaerobic metabolism detected with a ratio (PvCO2-PaCO2)/(CaO2-CvO2) variation and still is detected with lactate level variations.

**Compliance with ethics regulations:** Yes in clinical research.

### FC-027 Bacterial coinfection in critically ill COVID-19 patients with severe pneumonia

#### Alexandre Elabbadi^1^, Matthieu Turpin^1^, Grigoris Gerotziafas^2^, Marion Teulier^1^, Guillaume Voiriot^1^, Muriel Fartoukh^1^

##### ^1^Assistance Publique des Hôpitaux de Paris, Médecine Intensive Réanimation, Hôpital Tenon, Paris, France; ^2^Assistance Publique des Hôpitaux de Paris, Thrombosis Center, Service d’Hématologie Biologique, Hôpital Tenon, Paris, France

**Correspondence:** Alexandre Elabbadi - alexandre.elabbadi@aphp.fr

*Annals of Intensive Care* 2021, **11(Suppl 1):**FC-027

**Rationale:** Severe 2019 novel coronavirus infectious disease (COVID-19) with pneumonia is associated with high rates of admission to the intensive care unit (ICU). Bacterial coinfection has been initially reported to be rare. We aimed at describing the rate of bacterial coinfection in critically ill adult patients with severe COVID-19 pneumonia.

**Patients and methods/materials and methods:** All the patients with laboratory-confirmed severe COVID-19 pneumonia admitted to the ICU in a university-teaching hospital, from February 22 to May 7th, 2020 were included. Respiratory tract specimens were obtained within the first 48 h of ICU admission.

**Results:** During the study period, 101 patients were referred to the ICU for COVID-19 with severe pneumonia. Most patients (*n* = 83; 82.2%) were intubated and mechanically ventilated on ICU admission. Overall, 20 (19.8%) respiratory tract specimens obtained within the first 48 h. *Staphylococcus aureus* was the main pathogen identified, accounting for almost half of the early-onset bacterial etiologies.

**Conclusion:** We found a high prevalence of early-onset bacterial coinfection during severe COVID-19 pneumonia, with a high proportion of *S. aureus*. Our data support the current WHO guidelines for the management of severe COVID-19 patients, in whom antibiotic therapy directed to respiratory pathogens is recommended.

**Compliance with ethics regulations:** Yes in clinical research

### FC-028 Bacterial co-infections during severe COVID-19 pneumonia: prognosis consequences

#### Frédéric Martino, Laure Flurin, Jean-David Pommier, Laurent Camous, Michel Carles

##### CHU DE LA GUADELOUPE, Les Abymes, Guadeloupe

**Correspondence:** Frédéric Martino - frederic.martino@chu-guadeloupe.fr

*Annals of Intensive Care* 2021, **11(Suppl 1):**FC-028

**Rationale:** COVID-19, through lung damages, need of mechanical ventilation, and viral infection-related immunosuppression, increases the risk of co-infection with other pathogens.

**Patients and methods/materials and methods:** We included all consecutive patients hospitalized for a severe COVID-19 pneumonia between March 15th and April 22nd 2020 in the Intensive Care Unit (ICU). Steroids and high-dose anticoagulation were not in the standard of care at this time. A systematic initial antibiotic therapy by macrolide for 7 days was done. Nosocomial bacterial co-infections (Coinf + group) diagnosis included at least a microbiological confirmation of an evocative clinical picture, enforced by a CT-scan criteria each time it was appropriated or possible, during ICU stay.

**Results:** Thirty-two patients were included, among them 23 required mechanical ventilation (MV). Most were Afro-Caribbean male patients (73%), mean age of 67 ± 11.7 years old, having a treated hypertension in 41% and diabetes in 31% of cases. The mean body mass index was 27 (kg/m^2^), with no difference between CoInf + (*n* = 20) and CoInf− (*n* = 12). The Sequential Organ Failure Assessment (SOFA) at ICU admission was 7.1 ± 4.4 in Coinf + vs. 3.2 ± 2.5 in the CoInf−, *p *= 0.007. MV during ICU was 100% in Coinf + vs. 25% in Coinf−, *p* < 0.001 A steroid treatment was initiated in 10 patients, including *n* = 6 CoInf + vs *n* = 4 CoInf−, *p *= 1.0. In Coinf + group, 30 bacterial co-infections occurred including 16 blood stream infection and 14 ventilator-acquired pneumonia (with seven associated bacteremia). Most involved pathogens were enterobacteracae (*Klebsiella*, *Enterobacter* *n* = 12), *Staphylococcus aureus* *n* = 5 or non-fermentative Gram-negative bacilli as *Pseudomonas aeruginosa* *n* = 6 and others *n* = 7. Bacterial co-infections occurred only in MV patients. A clinical improvement (microbiological and/or radiological success) was achieved for 19 patients (59%). Thirteen patients over 32 (40%) died during hospitalization, most of them (11) in Coinf + group. Bacterial co-infection was identified as the main cause of death in 7/11 (64%) (medical staff reassessment). At ICU day 7, biological mean data Coinf + vs. Coinf− were: lymphocytes 0.9 vs. 2.07 G/L, *p *< 0.05, platelets 247 vs. 405 G/L, *p *< 0.05 and C-reactive protein 275 vs. 100 mmol/L, *p *< 0.01.

**Conclusion:** Bacterial co-infection is frequent in severe COVID-19 pneumonia requiring ICU and strongly associated to MV. This complication is associated with an increased risk of death. An immunosuppression, COVID-19-related or due to anti-inflammatory therapeutics could be the main trigger of this harmful complication.

**Compliance with ethics regulations:** Yes in clinical research.

### FC-029 Dexamethasone in covid-19 infected patients: 12 mg versus 24 mg

#### Saba Makni, Sana Kharrat, Kamilia Chtara, Olfa Turki, Rania Ammar, Salma Jerbi, Hedi Chelly, Chokri Ben Hamida, Mabrouk Bahloul, Mounir Bouaziz

##### CHU Habib Bourguiba, Sfax, Tunisie

**Correspondence:** Sana Kharrat - sanakharrat15@hotmail.com

*Annals of Intensive Care* 2021, **11(Suppl 1):**FC-029

**Rationale:** Coronavirus disease 2019 (COVID-19) remains an increasing global pandemic, with significant morbidity and mortality. SARS coronavirus infections are known to induce a storm of pro-inflammatory cytokines and subsequent tissue damage in the lungs in moderate-to-severe cases. The treatment of choice for this new disease remains unknown. Recent reports indicated that the corticosteroid dexamethasone may modulate inflammation-mediated lung injury and may reduce mortality of severe COVID-19 patients. In our study, we aimed to compare clinical outcomes of hospitalized COVID-19 patients receiving different doses of corticosteroids therapy.

**Patients and methods/materials and methods:** We conducted a retrospective study in an intensive care unit including critically ill patients with confirmed SARS-COV2 infection between September 2020 and January 2021. We compared two different doses of dexamethasone (24 mg per day for group 1 versus 12 mg per day in group 2) given intravenously over 10 days.

**Results:** We evaluated 119 patients with COVID-19 admitted to our institution. The median [IQR] age was 65 [56–72] years and 90 patients (75.6%) were male. Among these patients, 56 (47.1%) had hypertension, 54 (45.4%) had diabetes and 13 (10.9%) had chronic lung disease. Mean SAPSII score on ICU admission was 34.3 ± 15.5 and mean SOFA score was 5 ± 2.9. On admission, all inpatients presented a respiratory distress and received dexamethasone. 43 patients (36.1%) were included in group 1 and 76 (63.9) patients in group 2. There was no difference in mortality rate (44% vs. 46%) or the need of mechanical ventilation (31% vs. 39%) between the two groups. However, in the higher dose dexamethasone group, we noticed a higher incidence of hyperglycemia (53% vs. 34%; *p *= 0.04) and higher rate of hypokalemia (12% vs. 6%; *p *= 0.039).

**Conclusion:** It seems that there is similar efficacy between the dexamethasone treatment groups at the doses of 12 mg and 24 mg. However, the high-dosage group patients exposed patients with severe COVID-19 to more metabolic complications.

**Compliance with ethics regulations:** N/A

### FC-030 Does the use of iv corticosteroids increase the risk of infection in critical patients admitted to the ICU for severe forms of SARS-CoV-2 Pneumonia?

#### Maria Aroca, Luis Ensenat, Danielle Reuter, Sophie Marque^1^, Pierrick Cronier^1^, Karim Chergui^1^, Cecilia Billou^1^, Celine Clergue^1^, Marie Baron^1^, Nicola Maziers^1^, François-Xavier Laborne^1^, Besma Zbidi^1^, Delphine Ceraudo^1^, Richard Boiteau^1^, Mathieu Desmard^1^, Guillaume Chevrel^1^

##### ^1^Centre Hospitalier Sud Francilien, Corbeil-Essonnes, France

**Correspondence:** Maria Aroca - mariaarocaruiz@gmail.com

*Annals of Intensive Care* 2021, **11(Suppl 1):**FC-030

**Rationale:** COVID-19 severe pneumonia has brought national and international health systems to limits never explored before in our century. No other infectious disease has become explosively epidemic within our days, considering treatment options and support care available nowadays. Thus, the application of modern methodologies has allowed clinicians to change protocols in accordance with available data. We aim to compare infection incidence between first and second waves in patients admitted to the critical care unit of our hospital, considering as main changes the different profiles as well as the addition of intra-venous corticoids to the standard care.

**Patients and methods/materials and methods:** In collaboration with the research unit, a registry of all patients who had suffered a severe form of SARS-CoV-2 pneumonia and admitted to the critical care unit from 06/03/2020 to 28/12/2020 was created. Data regarding demographic characteristics, past medical history, presenting symptoms, labs, ventilation strategies, ECMO support, renal replacement therapy, drugs used, complications and status at 28 days of admissions were collected and analyzed. The statistical analysis performed included a univariate analysis; the median of continuous variables were compared by Wilcoxon test; while for categorical variables a comparison of percentages with their 95% confidence interval was performed with a Fisher test. When data were unavailable, and in order to decrease bias and increase precision, a multi-variate analysis MICE (multivariate imputation by chained equations) was used. In order to compare periods, the second wave was defined as any patient with severe COVID19-form arriving after August 2020 and the first wave, as any patient arriving earlier than August.

**Results:** Two hundred and twelve patients were admitted to our ICU with a positive RT-PCR SARS-CoV-2 test from 06/03/2020 to 28/12/2020, having a global mortality of 33.7%. As defined before, 124 patients were admitted during the first wave period, and 88 during the second wave. Globally, more patients received intravenous corticoids during the second wave when compared to the first (88 out of 88, compared with 12 out of 124, *p *< 0.0001). No statistically significant difference was found in the incidence of associated ventilator pneumonia (57 vs. 37 patients, *p *= 0.48); bacteremia (12 vs. 2; *p *= 0.63); or catheter infection (9 vs. 6, *p *> 0.99).

**Conclusion:** No statistically significant differences were found in the incidence of infectious complications between the two periods analyzed, which markedly differ in the use of intravenous corticosteroids as standard care.

**Compliance with ethics regulations:** Yes in clinical research.

### FC-031 Comparison of two different corticosteroid doses in critically ill patients admitted for SARS-COV2 pneumonia

#### Hedia Ben Ahmed, Oussama Jaoued, Sabrina Chaouch, Wael Chamli, Hajer Nouira, Habiba Ben Sik Ali, Mohamed Fekih Hassen, Souheil Atrous

##### service de réanimation médicale hôpital Taher Sfar, Mahdia, Tunisie

**Correspondence:** Oussama Jaoued - oussamajaoued@gmail.com

*Annals of Intensive Care* 2021, **11(Suppl 1):**FC-031

**Rationale:** Systemic corticosteroid treatment is commonly used among critically ill patients with viral pneumonia, including severe acute respiratory syndrome. The use of corticosteroids is now recommended in critically ill patients with hypoxemic respiratory failure caused by SARS-Cov-2 pneumonia. The aim of our study is to determine the effect of different doses of corticosteroid on critically ill patients’ mortality with severe SARS-Cov-2 pneumonia.

**Patients and methods/materials and methods:** Inclusion criteria were adult older than 18 years with confirmed SARS-CoV-2 pneumonia, having symptoms evolving for more than 10 days and admitted to the intensive care unit (ICU) between October and December 2020. Patients dead within 24 h after admission were excluded. All patients received corticosteroid on admission. The choice of the dose was left to the attending physician. Patients were assigned to 2 groups. Group 1: low-to-moderate dose (LMD) of methylprednisolone defined as ≤ 80 mg per day of methylprednisolone (≤ 1 mg/kg per day) or equivalent. Group 2: high dose (HD) of corticosteroids defined as > 80 mg per day of methylprednisolone (> 1 mg/kg per day) or equivalent. Primary outcome was ICU mortality. Secondary outcomes were duration of invasive mechanical ventilation (IMV), ICU length of stay and the occurrence of corticosteroids adverse effects.

**Results:** A total of 85 patients with a mean age of 62 ± 12 years and a mean APACHE-II of 11 ± 4 were included. Mean SOFA score was 4.6 ± 2.5. At randomization, 18(21%) patients were on IMV and 53 patients (62%) were receiving high-flow nasal oxygen therapy (HFNO). Twenty-eight patients were intubated during hospitalization, so a total of 55% of patients received IMV. ICU length of stay was 15 ± 11 days. ICU mortality was 41%. Thirty-eight patients (44.7%) received HD of corticosteroid. HFNO duration was similar among groups (5.3 vs. 4.9 days, *p *= 0.7). Secondary intubation’s rate was comparable between LMD and HD corticosteroids (44% vs. 32%, *p *= 0.3). Duration of IMV and length of stay were similar among groups. There was no difference in mortality rate: 44% in group 2 vs. 32% in group 1. Although not statistically significant, nosocomial infection rate was higher in HD group (59% vs. 39%, *p *= 0.06). The other adverse effects were similar among groups. In multivariate regression analysis, corticosteroids dose was not associated with mortality. Factors associated with mortality were SOFA score (OR = 1.493; 95% IC: 1.089–2.046, *p *= 0.01) and nosocomial infection (OR = 9.948; 95% IC: 2.730–36.255, *p *= 0.001).

**Conclusion:** High dose of corticosteroids seems to increase the rate of nosocomial infection. Different corticosteroids doses do not affect mortality.

**Compliance with ethics regulations:** Yes in clinical research.

### FC-032 Hydrocortisone among critically ill patients with COVID-19: a post hoc analysis of the CAPE-COVID trial

#### Pierre-François Dequin^1,2,3,21^, Nicholas Heming^4,5,21^, Ferhat Meziani^6,7,21^, Gaëtan Plantefève^8,21^, Guillaume Voiriot^9^, Julio Badié^10,21^, Bruno François^11,12,13,21^, Cécile Aubron^14^, Jean-Damien Ricard^15^, Stephan Ehrmann^1,2,3,21^, Youenn Jouan^1,2,3,21^, Antoine Guillon^1,2,3,21^, Marie Leclerc^16^, Carine Coffre^16^, Hélène Bourgoin^17^, Céline Lengellé^18^, Caroline Caille-Fénérol^13,21^, Elsa Tavernier^3,21^, Sarah Zohar^19^, Bruno Giraudeau^3,20,21^, Djillali Annane^4,5,21^, Amélie Le Gouge^3,21^

##### ^1^Hôpital Bretonneau, CHU et Université de Tours, Tours, France; ^2^INSERM UMR1100 Centre d’Etude des Pathologies Respiratoires, Tours, France; ^3^INSERM CIC 1415, Tours, France; ^4^Hôpital Raymond Poincaré, AP-HP et Université Paris Saclay, Garches, France; ^5^INSERM UMR 1173, Garches, France; ^6^Nouvel Hôpital Civil, CHU et Université de Strasbourg, Strasbourg, France; ^7^INSERM UMR 1260, Strasbourg, France; ^8^Hôpital Victor Dupouy, Argenteuil, France; ^9^Hôpital Tenon, AP-HP et Université Paris Sorbonne, Paris, France; ^10^Hôpital Nord Franche-Comté, Trevenans, France; ^11^Hôpital Dupuytren, CHU et Université de Limoges, Limoges, France; ^12^INSERM UMR 1092, Limoges, France; ^13^INSERM CIC 1435, Limoges, France; ^14^Hôpital de la Cavale Blanche, CHU et Université de Bretagne Occidentale, Brest, France; ^15^Hôpital Louis Mourier, AP-HP et Université de Paris, Colombe, France; ^16^Délégation à la Recherche Clinique et à l’Innovation, CHU de Tours, Tours, France; ^17^Pharmacie, CHU de Tours, Tours, France; ^18^Pharmacovigilance des Essais Cliniques, CHU de Tours, Tours, France; ^19^INSERM, Centre de Recherche des Cordeliers, Université de Paris Sorbonne, Paris, France; ^20^INSERM UMR 1246, Universités de Nantes et Tours, Tours, France; ^21^CRICS - TriGGERSep Network, Strasbourg, France

**Correspondence:** Pierre-François Dequin - dequin@univ-tours.fr

*Annals of Intensive Care* 2021, **11(Suppl 1):**FC-032

**Rationale:** Dexamethasone decreases mortality in severe forms of COVID-19. The release of this result resulted in the premature termination of trials evaluating hydrocortisone for ethical reasons. Their lack of power has left a doubt about its effectiveness. In the CAPE-COVID trial [1], low-dose hydrocortisone, compared with placebo, did not significantly reduce treatment failure at day 21, nor did it significantly reduce death at day 21 (14.7 vs. 27.4%, *p *= 0.057). We report here a post hoc analysis of the longer-term effects of hydrocortisone in this trial.

**Patients and methods/materials and methods:** ICU patients with a severe form of COVID-19 (mechanically ventilated and/or PaO_2_/FiO_2_ < 300) were randomized to receive hydrocortisone 200 mg/d initially or placebo, in a double-blind fashion. Mortality at day 28 and 90 were analyzed using two-proportion z-tests based on normal approximation. Differences of proportions were estimated with their confidence interval. Lengths of ICU stay were compared in the framework of a competing risk model, with death considered as a competing event of end of stay.

**Results:** 150 patients were included (45 women, mean age 62.3 years, 122 mechanically ventilated at inclusion, mean PaO_2_/FiO_2_ 145.7, mean SOFA 6.0). At day 28, mortality was 12/76 in the hydrocortisone group and 22/74 in the placebo group, difference of proportions − 13.9% [95%CI: − 27.2%; − 0.7%], *p *= 0.04. There was 14.0 [IQR 0.0; 21.0] ventilator-free days in the hydrocortisone group vs. 6.5 [0.0; 18.0] in the placebo group, *p *= 0.08. At day 90, mortality was 17/75 in the hydrocortisone group and 26/73 in the placebo group (2 missing data), difference of proportions − 12.9% [95%CI: −27.5%; 1.6%], *p *= 0.08. Figure 1 shows ICU mortality and ICU discharge as a function of time.

**Conclusion:** In this double-blind, placebo-controlled, multicentric trial, post hoc analysis suggests a decrease in mortality at day 28 in patients with severe forms of COVID-19 receiving hydrocortisone. Early termination of the trial made it underpowered to show a significant difference on other endpoints.

**Reference**

1. JAMA 2020;324:1298–306.

**Compliance with ethics regulations:** Yes in clinical research.
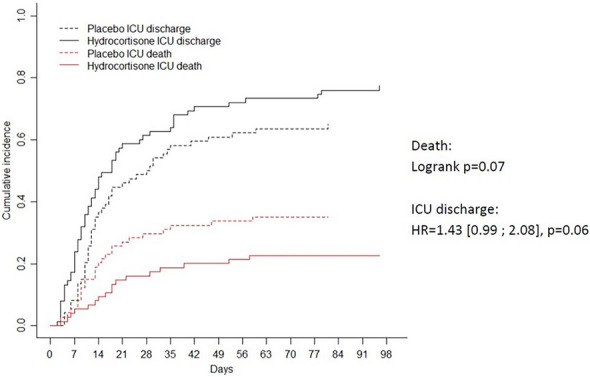


Fig. 1 Mortality (in red) and discharge from ICU (in black) for both groups

### FC-033 Presentation and outcome of tuberculous meningoencephalitis: a retrospective cohort study in immunocompetent adult patients

#### Ahlem Trifi, Asma Mehdi, Eya Seghir, Cyrine Abdennebi, Yosr Touil, Foued Daly, Sami Abdellatif, Salah Ben Lakhal

##### medical ICU la Rabta hospital, Faculty of Medicine of Tunis, Tunis, Tunisie

**Correspondence:** Ahlem Trifi - trifiahlem2@gmail.com

*Annals of Intensive Care* 2021, **11(Suppl 1):**FC-033

**Rationale:** Tuberculous meningoencephalitis (TME) is the most severe form of tuberculosis disease. The clinical features are non-specific and the diagnosis is based on brain imaging and cerebrospinal fluid (CSF) analysis. We aimed to study the diagnostic, therapeutic and outcome characteristics of TME in immunocompetent patients.

**Patients and methods/materials and methods:** Retrospective and descriptive study including immunocompetent patients hospitalized in ICU from January 2016 to June 2020 for TME. All clinical, paraclinical and outcome data were collected.

**Results:** During the study period, there were 758 admissions including 36 for tuberculosis among them 18 TME, resulting to an annual incidence of 23.3/1000 admissions and 50% of cerebral location. Gender ratio = 1.57, mean age = 48 years, median SOFA = 4, and previous tuberculosis was reported in 3 patients. The time from initial signs to consultation and admission was 14 and 21 days, respectively. Fever was present in all patients and seizures in 13 cases. On admission, 2/3 had a median GCS at 8.5. All patients had lymphopenia, mean CRP = 117 mg/l and procalcitonin = 0.23. Severe hyponatremia was observed in three patients. In 11/18, the cerebral CT revealed hydrocephalus and hypodensities. 14 had additional MRI which was contributory in seven cases, particularly in terms of tuberculomas and vasculitis. For CSF analysis, the lymphocyte formula was predominant (14 cases), the mean glucorrachia/blood glucose ratio = 0.36, the median proteinorrachia = 1.6 g/l and median lactatorrachia = 5 mmol/l and PCR was positive in two cases. Isoniazid/rifampicin/pyrazinamide/ethambutol quadruple therapy in addition to dexamethasone was prescribed for all patients and two had an external ventricular bypass. Invasive ventilation was indicated in 13 patients including one for ARDS related to miliary tuberculosis. Outcome: 13 patients (72%) had favourable outcome and the others: brain death (2), vegetative state (2) and major deficit (1). Mortality (all cause combined) was 39% (7/18). For the current state: three of the nine contacted have fully recovered and six have no serious sequelae.

**Conclusion:** TME is a non-rare affection with severe morbidity and mortality, particularly motor and cognitive sequelae. The clinical signs lack specificity and brain imaging, especially angio MRI, in addition to CSF analysis, are the key elements of diagnosis. However, the delay in their reports leads to therapeutic delay, which is a determining factor in the prognosis. The establishment and generalization of the PCR technique using the MTB/RIF (Xpert) Ultra gene is an absolute priority, the only guarantee of a rapid diagnosis and thus an early treatment.

**Compliance with ethics regulations:** Yes in clinical research.

### FC-034 Systematic brain CT-scan for neuroprognostication of critically ill adult patients with infective endocarditis: a single-center retrospective study

#### Thomas Rambaud^1,2,4^, Etienne De Montmollin^2^, Pierre Jaquet^2^, Sonia Abid^2,5^, Michel Wolff^2,3^, Lila Bouadma^2^, Jean-François Timsit^2^, Romain Sonneville^2,4^

##### ^1^Hôpital Avicenne AP-HP, Réanimation médico-chirurgicale, Bobigny, France; ^2^Hôpital Bichat AP-HP, Réanimation médicale et infectieuse, Paris, France; ^3^Hôpital Sainte-Anne, réanimation, Paris, France; ^4^INSERM U1148 Laboratoire de Recherche Vasculaire Translationelle, Paris, France; ^5^Hopital Saint Louis AP-HP, Anesthésie-réanimation chirurgicale, Paris, France

**Correspondence:** Thomas Rambaud - rambaud.t@gmail.com

*Annals of Intensive Care* 2021, **11(Suppl 1):**FC-034

**Rationale:** Neurological failure is a strong determinant of outcome in critically ill patients with infective endocarditis (IE). Current guidelines (European Society of Cardiology (ESC) 2015) recommend systematic brain imaging in pre-operative evaluation. However, the impact of such imaging has never been evaluated in critically ill patients. Our aim was to assess the impact of CT-defined NC on functional outcome of critically ill IE patients.

**Patients and methods/materials and methods:** This retrospective cohort study included all consecutive patients aged ≥ 18 years with a severe (SOFA ≥ 2) left-sided definite IE (diagnosed according to Duke criteria) hospitalized ≤ 30 days after initiation of anti-microbial treatment in a tertiary care hospital’s ICU. Patients with no baseline brain CT, transferred to ICU after cardiac surgery for IE or with ICU-acquired IE were excluded. Baseline CT-scans were classified in five mutually exclusive categories (normal CT, moderate-to-severe acute ischemic stroke (AIS), minor AIS, intracranial hemorrhage (ICH), other abnormal CT) using a double reading, blinded to patients’ characteristics and outcomes. Main outcome was the proportion of patients with 1-year favorable outcome, defined as a modified Rankin Scale score between 0 (asymptomatic) and 3 (moderate disability). Factors associated with favorable outcome were identified by multivariable logistic regression. Secondary outcome was the rate of post-operative severe NC. Neurological surgical contraindication was defined according to ESC 2015 guidelines as hemorrhagic (any ICH) or ischemic (AIS with Glasgow Coma Scale < 10).

**Results:** Between 06/01/2011 and 07/31/2018, 156 patients (male 71%, 63 years old [54–70]) were included in the analysis (284 included, 128 excluded due to pre-specified criteria). 87/156 (56%) had a CT-defined NC: moderate-to-severe AIS *n* = 33/156 (21%), ICH *n* = 24/156 (15%), minor AIS *n* = 29/156 (19%), other *n* = 3/156 (2%). 69 patients (45%) had a 1-year favorable outcome. Factors associated with favorable outcome are detailed in Table 1. Compared to normal CT, only moderate-to-severe AIS was associated with a higher rate of post-operative severe NC (*n* = 8/23 (35%) vs. *n* = 1/46 (2%), *p *< 0.01). Urgent surgery was performed in 28 patients despite neurological contraindication. One-year favorable outcome was more frequent in patients operated despite hemorrhagic contraindication than in patients operated despite ischemic contraindication (*n* = 12/18 (67%) vs. *n* = 2/10 (20%) *p *= 0.046).

**Conclusion:** Moderate-to-severe AIS had an independent negative impact on 1-year functional outcome in critically ill IE patients, whereas other complications, including ICH, had no such impact. Sensitivity analyses conducted in operated patients revealed similar findings. Systematic use of brain imaging might improve management of critically ill patients with left-sided IE.

**Compliance with ethics regulations:** Yes in clinical research.
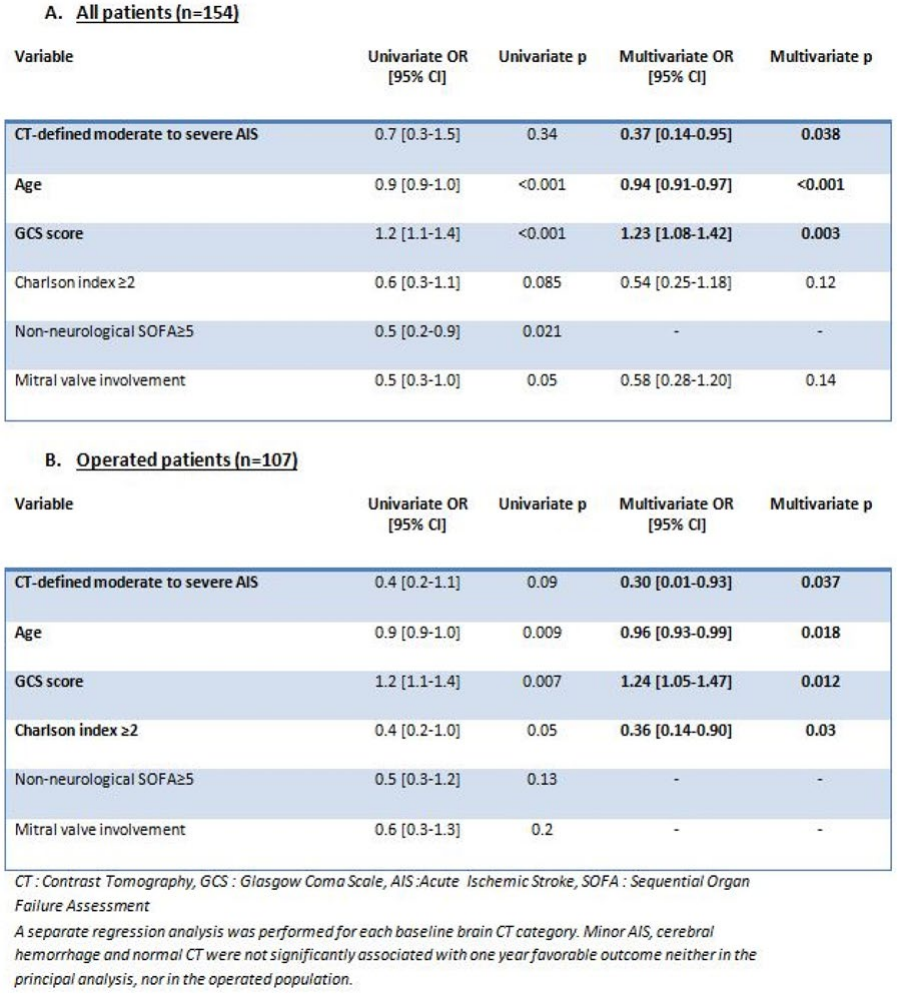


Table 1 multivariable analysis of factors associated with favorable functional outcome (mRS 0–3) at 1 year

### FC-035 Prevalence, associated factors and impact of a first false-negative PCR in critically ill Herpes simplex virus encephalitis patients

#### Etienne De Montmollin^1,4^, Claire Dupuis^2^, Pierre Jaquet^1^, Benjamine Sarton^3,6^, Michel Wolff^1^, Jean-François Timsit^1,4^, Romain Sonneville^1,5^

##### ^1^APHP, Hôpital Bichat - Claude Bernard, Paris, France; ^2^Hôpital Gabriel Montpied, Clermont-Ferrand, France; ^3^Hôpital Purpan, Toulouse, France; ^4^INSERM UMR 1137, Université de Paris, Paris, France; ^5^INSERM UMR 1148, Université de Paris, Paris, France; ^6^INSERM UPS, Université de Toulouse, Toulouse, France

**Correspondence:** Etienne De Montmollin - etienne.demontmollin@aphp.fr

*Annals of Intensive Care* 2021, **11(Suppl 1):**FC-035

**Rationale:** In Herpes simplex virus (HSV) encephalitis (HSE), among patients who require intensive care admission, 70% have a poor neurological outcome (modified Rankin score (mRS) 3–6) at 90 days [1]. HSE remains a diagnostic challenge, notably because of possible initial normal cerebrospinal fluid (CSF) content. Although the sensitivity of HSV PCR is around 95% [2], there are case reports/series reporting false-negative results if PCR is performed within 4 days of symptoms onset. We aimed to describe the prevalence, associated factors and impact of a first false-negative HSV PCR in a large database of critically ill CSF PCR-proven HSE patients

**Patients and methods/materials and methods:** We conducted a re-analysis of retrospective multicenter database of PCR-proven HSE patients from 47 ICUs in France between 2007 and 2017 [1]. Logistic regression was used to identify the specific effect off an initial false-negative HSV PCR on poor neurological outcome, defined as an mRS ≥ 4 (indicating severe disability or death) at hospital discharge

**Results:** A total of 273 patients, age: 64.6 [55.5; 73.7] years, Glasgow Coma Score: 9 [6; 12] at ICU admission, were included. Time from first symptoms to first lumbar puncture (LP) and ICU admission were 1 [0; 3] and 2 [1; 4] days, respectively. Initial HSV PCR was negative in 12 (4.4%) cases. Poor outcome at hospital discharge occurred in 101 (37%) patients. In univariate analysis, the only clinical feature positively associated with a first negative PCR was the presence of focal neurological signs (6/12 (50%) vs. 62/259 (23.9%), *p *= 0.04). Compared to patients with a first positive PCR, patients with a first negative PCR had lower CSF pleocytosis (4 [3; 62] vs. 51 [12; 160] cells/mm^3^, *p *= 0.02) and lower CSF protein levels (0.5 [0.3; 0.6] vs. 0.7 [0.5; 1.2] g/L, *p *= 0.01). There were no false-negative HSV PCR when LP was performed ≥ 4 days after the onset of symptoms. Having a first false-negative HSV PCR was associated with an increased delay between LP and acyclovir treatment (2.5 [1.5; 7] vs. 0 [0; 0] days, *p *< 0.01). Multivariable analysis revealed that an initial false-negative HSV PCR was independently associated with a poor neurological outcome (Table 1).

**Conclusion:** In critically ill HSE patients, a first false-negative HSV PCR occurred in 4.4%, only in LPs performed less than 4 days from symptom onset. False-negative HSV PCR were associated with lower CSF inflammation, increased delay in acyclovir treatment, and were independently associated with worse neurological outcome at hospital discharge

**References**

1. Jaquet P, de Montmollin E, Dupuis C, Sazio C, Conrad M, Susset V, et al. Functional outcomes in adult patients with herpes simplex encephalitis admitted to the ICU: a multicenter cohort study. Intensive Care Med. 2019;45:1103–11.
2. Tansarli GS, Chapin KC. Diagnostic test accuracy of the BioFire^®^ FilmArray^®^ meningitis/encephalitis panel: a systematic review and meta-analysis. Clin Microbiol Infect. 2020;26:281–90.

**Compliance with ethics regulations:** Yes in clinical research.
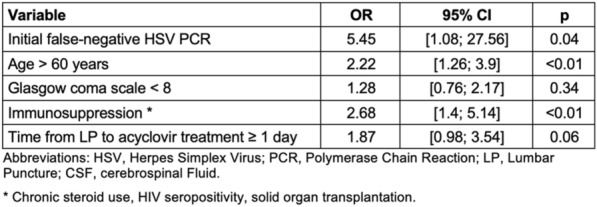


Table 1 Multivariable logistic regression of factors associated with a modified Rankin score 4–6 at hospital discharge

### FC-036 ICU admission plasma fibrinogen can predict cognitive impairment in mechanically ventilated COVID-19 patients

#### Sarah Saxena, Alexandre-Raphael Wery, Adrian Khelif, Christophe Lelubre, Patrick Biston, Michael Piagnerelli

##### CHU de Charleroi, Charleroi, Belgique

**Correspondence:** Sarah Saxena - sarahksaxena@gmail.com

*Annals of Intensive Care* 2021, **11(Suppl 1):**FC-036

**Rationale:** COVID-19 ARDS is associated with long periods of mechanical ventilation and sedation. Alterations of the cognitive state were reported in 69% of patients suffering from COVID-19 ARDS. Not only do the exact pathophysiological mechanisms behind neurocognitive dysfunction in COVID-19 patients remain unknown, but it is also difficult to predict which patients are likely to suffer from this phenomenon. In this retrospective study in all patients admitted for hypoxia due to COVID-19, the impact of demographic factors as well inflammation-related factors at ICU admission on the development of delirium during ICU stay were evaluated.

**Patients and methods/materials and methods:** After approval of the ethical committee, we included patients admitted to the ICU of the CHU de Charleroi, Belgium, between March to December 2020 for invasive mechanical ventilation with sedation due to COVID-19 ARDS. Pregnant patients and patients requiring ECMO support were excluded. A standard case report form was used for de-identified data collection. Data including delirium status, were collected from the patients’ medical records by three independent researchers. Statistical methods included classical parametrical and non-parametrical tests for qualitative and quantitative variables, as well as calculations of areas under the ROC curve (AUC ROC) for delirium binary prediction.

**Results:** 78 patients were included in this study. Demographic factors and comorbidities were not different between patients with or without ICU delirium. Out of several ICU admission inflammation markers (ferritin, CRP, white blood cells, lymphocytes, D-dimers, fibrinogen), higher fibrinogen levels were associated with altered cognitive state during ICU stay (7.19 [6.58–8.30] in patients with delirium vs. 6.63 [5.41–7.77] mg/dL in patients without delirium, *p *= 0.025; AUC ROC: 0.65 [95% CI: 0.53–0.75], *p *= 0.02) (Table 1).

**Discussion:** Recently, fibrinogen has been used as a marker of blood brain barrier disruption in neurodegenerative diseases, such as Alzheimer’s disease and multiple sclerosis. Increased fibrinogen levels can indeed lead to microglial activation through the CD11b/CD18 pathway and have been associated with cognitive decline (1). Interestingly, some authors have suggested that neurological symptoms in COVID-19 patients might be due to blood brain barrier disruption and subsequent microglial activation (2).

**Conclusion:** This retrospective study highlights the role of ICU admission plasma fibrinogen as an easy-to-dose marker of cognitive impairment in mechanically ventilated COVID-19 patients. Large prospective clinical trials are needed to confirm this finding.

**References**

1. Davalos D et al. Fibrinogen-induced perivascular microglial clustering is required for the development of axonal damage in neuroinflammation. Nat Commun; 2012.
2. Achar A et al. COVID-19-associated neurological disorders: the potential route of cns invasion and blood–brain barrier relevance. Cells; 2020.

**Compliance with ethics regulations:** Yes in clinical research.
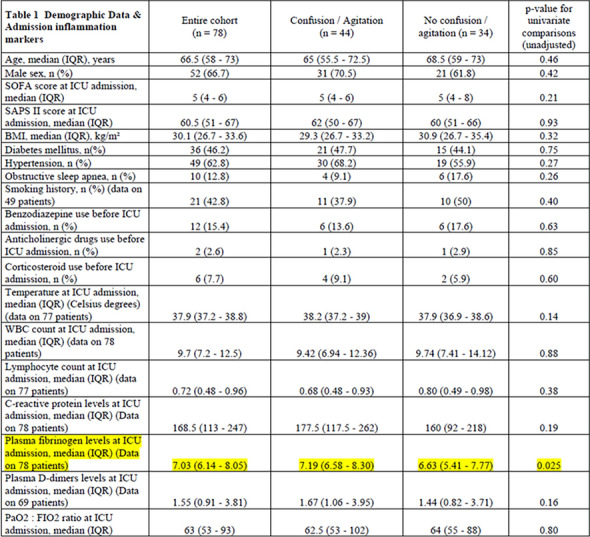


Table 1 Demographic data and admission inflammation markers

### FC-037 Brainstem dysfunction in COVID-19 critically ill patients with ARDS: a prospective multicentric observational study

#### Sarah Benghanem^2,3^, Alain Cariou^2,3,4^, Jean-Luc Diehl^1,3,7,8^, Julien Charpentier^2^, Jean-Loup Augy^1,3^, Caroline Hauw-Berlemont^1^, Frédéric Pène^2,3^, Jean-Paul Mira^2,3^, Tarek Sharshar^5^, Bertrand Hermann^1,3,6^

##### ^1^Médecine Intensive Réanimation, Hôpital Européen Georges Pompidou, Assistance Publique - Hôpitaux de Paris-Centre (APHP-CUP), Paris, France; ^2^Médecine Intensive Réanimation, Hôpital Cochin, Assistance Publique - Hôpitaux de Paris-Centre (APHP-CUP), Paris, France; ^3^Faculté de Médecine, Université de Paris, Paris, France; ^4^Paris-Cardiovascular-Research-Center, INSERM U970, Paris, France; ^5^Neuroréanimation, GHU Sainte Anne, Paris, France; ^6^Institut du Cerveau et de la Moelle épinière - ICM, Inserm U1127, CNRS UMR 7225, F-75013, Paris, France; ^7^Innovative Therapies in Haemostasis, INSERM, F-75006, Paris, France; ^8^Biosurgical Research Lab (Carpentier Foundation), Paris, France

**Correspondence:** Bertrand Hermann - bertrand.hermann@aphp.fr

*Annals of Intensive Care* 2021, **11(Suppl 1):**FC-037

**Rationale:** Acute brain dysfunction with protracted disorders of consciousness has been described in severe Coronavirus Disease 2019 (COVID-19), with histological evidence of brainstem insults. We aimed to describe the prevalence prognostic value of clinical brainstem dysfunction (BS-dys) in critically ill COVID-19 patients.

**Patients and methods/materials and methods:** We conducted a prospective observational study in two French ICUs including mechanically ventilated patients with acute respiratory distress syndrome due to laboratory confirmed SARS-CoV-2 infection between April and December 2020. Brainstem reflexes were assessed in deeply sedated (RASS < −2, > 12 h) patients at least 12 h after neuromuscular blockade cessation to compute the Brainstem Responses Assessment Sedation Score, with BS-dys defined as a score ≥ 1. Primary outcome was the prevalence of BS-dys. Secondary outcomes were mortality, coma-free days, delirium free-days and ventilator free-days at day-28 of admission and day-14 of neurological assessment.

**Results:** Fifty-two patients were included, mostly male (81%), median age of 68 [56–74] years, median SAPS2 score 48 [35–69]. Patients were mainly sedated with midazolam (98%) and sufentanil (100%). Neurological assessment was performed 4 [3–7] days after admission, in patients with a median RASS of − 4 [− 4–5]). BS-dys was present in 26 (50%) patients, with abolition of grimacing to pain in 42%, cough reflex in 23% and corneal reflex in 8%. Clinical dysautonomia, defined by bradycardia and/or hypotension during suctioning maneuver was present in 19% and double-triggering in 17%. BS-dys was associated with higher SOFA scores (12 [10–14] vs. 8 [6–11], *p *= 0.003) and higher infusion rate of opioids (0.21 [0.14–0.25] vs. 0.12 [0.07–0.21], *p *= 0.030), with no differences in sedative infusion rate, PaO_2_/FiO_2_, PaCO_2_, blood glucose and urea, liver enzymes and ammonemia. Double-triggering asynchrony was more frequent in the BS-dys group (8 (31%) vs. 1 (3.8%), *p *= 0.024). BS-dys was associated with poor day-28 outcomes, namely lower coma-free days (3 [1–11] vs. 16 [3–22], *p *= 0.001), delirium-free days (2 [0–9] vs. 13 [2–20], *p *= 0.006) and ventilator-free days at day-28 (0 [0–0] vs. 12 [0–18], *p *= 0.01). After adjustment on SOFA, midazolam and opioid infusion rates, BS-dys remained associated with a lower cumulative incidence of coma recovery (adjusted HR 0.34 [0.12–0.95], *p *= 0.036) and delirium recovery (adjusted HR 0.30 [0.10–0.91], *p *= 0.027) at day-14 of assessment (Fig. 1).

**Conclusion:** The association of BS-dys with poor short-term outcome suggests that the brainstem, a structure playing a central role in arousal and vital functions, is particularly susceptible to pathophysiological mechanisms triggered by SARS-CoV-2, potentially contributing to the severity of COVID-19 in critically ill patients.

**Compliance with ethics regulations:** Yes in clinical research.
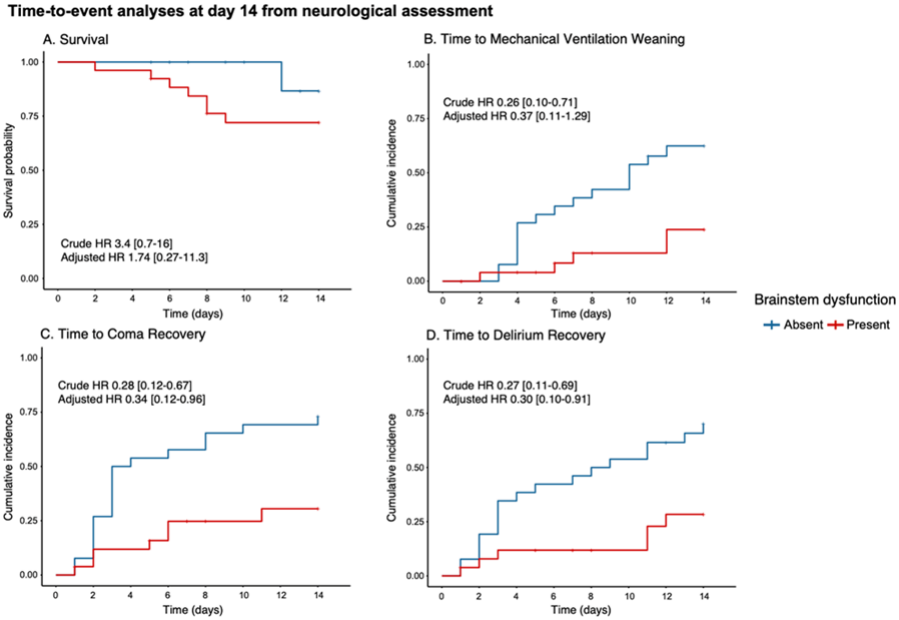


Fig. 1 Time-to-event analyses at day 14 from neurological assessment according to brainstem dysfunction

### FC-038 Neuro-psychiatric manifestations in severe SARS-CoV-2 infection: clinical features and impact on mortality

#### Fatma Essafi^1,2^, Amina Haddad^1,2^, Malek Kharrat^1,2^, Khaoula Ben Ismail^1,2^, Moez Kaddour^1,2^, Takoua Merhabene^1,2^

##### ^1^Intensive care unit, Regional Hospital, Zaghouan, Tunisie; ^2^Faculty of Medicine of Tunis, University Tunis El Manar, Tunis, Tunisie

**Correspondence:** Fatma Essafi - fatma.essafi@fmt.utm.tn

*Annals of Intensive Care* 2021, **11(Suppl 1):**FC-038

**Rationale:** Described initially as a respiratory tract disease, the 2019 coronavirus infectious disease (COVID-19) has been found to have causal association with a plethora of neurological, neuropsychiatric and psychological effects. We aimed to assess neuropsychiatric manifestations of severe COVID-19 pneumonia and determine its impact on patients’ management and outcome.

**Patients and methods/materials and methods:** We reviewed retrospectively all data of confirmed COVID-19 patients hospitalized in Zaghouan’s Hospital ICU between March 2020 and January 2021. Were collected epidemiological, clinical and paraclinical data, prescribed medications and outcomes. Factors associated with delirium and their impact on mortality were evaluated through binary logistic regression models.

**Results:** Among 121 COVID-19 patients, 79 patients have developed neurologic and/or psychiatric manifestations during their hospital stay (65%). First neurological examination was normal in all patients. Mean age was 63.5 ± 12.6 with sex ratio = 1.1. Mean SAPS II and APPACHE II scores were 26.7 ± 7.8 and 8 ± 3.8, respectively. Psychiatric manifestations were seen in 48 patients. Four of them had underlying psychiatric states. One patient had history of ischemic stroke with no sequelae. The most common psychiatric disorders included an unexpected agitation in 24 patients (50%), depressed mood in 12 patients (25%) and sleep disturbances in 8 patients (16.7%). Depression was manifested by slowed thinking and speaking, reduced appetite and anorexia, tiredness and lack of energy and hallucination. Delirium and confusion were reported in one case each. Neurologic features were reported in 45 patients. It consisted of 42 episodes of headache, 19 anosmia and/or ageusia, one ischemic stroke; no peripheral nervous system involvement or status epilepticus was reported. Management of patients consisted in majority of cases in benzodiazepine prescription alone or associated to neuroleptic agents. No differences were noted in demographic characteristics and history of neuropsychiatric pathology between patients who developed neuropsychiatric signs and the others. At admission, the two groups had also comparable respiratory exchange and laboratory findings, whereas those with psychiatric features were less adherent to awake prone position or non-invasive ventilation. In consequence, they required more invasive mechanical ventilation (54.2% vs. 32.9%, *p *= 0.02), had longer length of ICU stay (12 ± 2 vs. 8 ± 4.6, *p *< 0.05) and higher ICU mortality (56.3% vs. 35.6%, *p *= 0.025). Neurologic manifestations alone were not associated with worse prognosis.

**Conclusion:** Psychiatric manifestations are commonly seen in COVID-19 patients and are associated with poor impact on mortality. Long-term effects of these manifestations remain unknown. Early recognition, prompt management, and long-term follow-up are recommended to provide better care.

**Compliance with ethics regulations:** N/A.

### FC-039 Neuro-psychological outcome of ICU-admitted COVID-19 patients presenting with central nervous system complications

#### Juliette Pelle^1^, Thomas Nedelec^2^, Loïc Le Guennec^1^, Benjamin Rohaut^1,2^, Nicolas Weiss^1,2^, Albert Cao^1^, Nadya Pyatigorskaya^1,2^, Stéphanie Bombois^1^, Sophie Demeret^1^, Jean-Christophe Corvol^1,2^, Cécile Delorme^1,2^, Clémence Marois^1^

##### ^1^APHP, Hôpital de Pitié-Salpêtrière, Paris, France; ^2^Institut de Neurosciences Translationnelles IHU-A-ICM, Paris, France

**Correspondence:** Juliette Pelle - juliette.pelle@outlook.fr

*Annals of Intensive Care* 2021, **11(Suppl 1):**FC-039

**Rationale:** More than one-third of COVID-19 hospitalized patients present with neurological manifestations [1]. This prevalence is even higher in the most severe forms, i.e. requiring intensive care. In particular, ICU-associated encephalopathy has been commonly found in these patients, evaluated in up to 84% of ICU-admitted patients in a French monocentric observational study [2]. Thus, the aim of this study was to identify the central neurological symptoms in ICU-admitted COVID-19 patients and their long-term prognosis.

**Patients and methods/materials and methods:** A retrospective multicentric observational study was conducted between March 1st and April 30th 2020. Consecutive patients admitted to the ICU for a COVID-19 disease and for whom clinicians described central neurological manifestations such as encephalopathy, encephalitis, stroke, or epilepsy were included, according to a predefined protocol. Clinical characteristics such as preexisting comorbidities, cognitive and functional status, COVID-19 disease severity, organ failure occurrence, neurological symptoms were collected, as well as biological, electrophysiological and radiological parameters when available. Nine-month outcome was collected by a standardized telephone interview.

**Results:** Sixty-six patients were included, 79% were male; they were 60 [IQR = 14–8] years old, 58 (88%) patients had coexisting comorbidities, mainly cardiac diseases (67%), diabetes (41%), obesity (24%) and smoking (22%). Fifty-seven patients (86%) presented with encephalopathy, five (8%) with encephalitis, seven (11%) with stroke, four (6%) with epilepsy. At 9 months, seven patients (11%) had died, three patients (4.5%) were lost to follow-up. Among survivors, four (7%) remained in hospital (including two with persistent disorders of consciousness), 11 (20%) reported persistent central neurological symptoms. Patients are planned to be evaluated at 1 year.

**Conclusion:** Encephalopathy is the main neurological manifestation presented in our cohort while encephalitis appears to be rare. This result suggests that central neurological complications could be mainly caused by organ failure and intensive care therapies, known contributing factors to ICU-associated encephalopathy, rather than neurotropic invasion by SARS-CoV-2. An overall low mortality rate was observed, and 20% of patients reported persistent neurological complaint at 9 months. The 1-year neurological and neuropsychological assessment will enable the evaluation of the frequency and severity of long-term neurological sequelae through a standardized cognitive assessment, as well as quality of life and the presence of post-traumatic stress disorder.

**References**

1. Mao L et al. Neurologic Manifestations of Hospitalized Patients With Coronavirus Disease 2019 in Wuhan, China. JAMA Neurol. 2020; 77(6):683. 10.1001/jamaneurol.2020.1127.
2. Helms J et al. Delirium and encephalopathy in severe COVID-19: a cohort analysis of ICU patients. Crit Care. 2020; 24. 10.1186/s13054-020-03200-1.

**Compliance with ethics regulations:** Yes in clinical research

### FC-040 Post-traumatic hypoxemia in intensive care unit: incidence, causes and impact on outcome

#### Feki Alia, Mabrouk Bahloul, Rezk Ghorbel, Karama Mnif, Sana Kharrat, Hedi Chelly, Chokri Benhmida, Mounir Bouaziz

##### Centre Hospitalo-universitaire Habib Bourguiba sfax, Sfax, Tunisie

**Correspondence:** Rezk Ghorbel - rezkghorbel3@gmail.com

*Annals of Intensive Care* 2021, **11(Suppl 1):**FC-040

**Rationale:** Polytrauma is one of the major reasons for patients to be admitted in intensive care unit (ICU). Hypoxemia is one of the major complications observed in this group of patients. However, there is little data about this subject. This is why we conducted this study to evaluate the incidence of hypoxemia to determine each cause and to study its impact on outcome.

**Patients and methods/materials and methods:** We conducted a prospective study during the period between March the 1st 2019 and December 31st 2019 on 110 patients admitted to the ICU of the academic medical center Habib Bourguiba of Sfax to understand the causality of the hypoxemia in polytrauma patients and to analyze its prognosis.

**Results:** Out of 110 polytrauma patients, 83 showed hypoxemia manifestations. The mean age of polytrauma patients with hypoxemia was 35 years and the sex ratio was 5.4 (M/F). The average coma Glasgow score was 8 ± 4. The SOFA score, the SAPS II and the APACHE II score were assessed on the day of admission and the day hypoxemia first appeared and they showed averages of 8, 32 and 14, respectively. The blood glucose levels ranged from 0.5 to 4 g/dL with a mean of 1.34. The blood gas analysis showed that 53 patients developed a hypoxemia on day of admission with a PaO_2_/FiO_2_ ratio under 300 mmHg. Ninety-five percent of the patient underwent a body scan revealing brain contusion and cerebral hemorrhage in around half the cases and pulmonary contusion in 41% of the cases. Catecholamines were used in 59% of the cases while artificial respiration aid was needed in 94% of the patients. One-third of patients underwent a surgical intervention while 41% of the cases needed a blood transfusion. Factors associated with a poor prognosis in univariate analysis were lower PAO_2_/FiO_2_ ratio (*p *= 0.01), lower blood pH (*p *= 0.01), elevated blood sugar level on admission (*p *= 0.007), the need for blood transfusion (*p *= 0.022), surgical interventions (*p *= 0.037), elevated SOFA (*p *= 0.002), SAPS II (*p *= 0.028) and APACHE II (*p *= 0.023) scores. Multivariate analysis showed that advanced age (OR = 1.08), higher SOFA score (OR = 1.86) and sugar blood rates (OR = 1.64) were associated with a poor prognosis.

**Conclusion:** Hypoxemia is frequently observed in polytrauma patients and it is associated with a poor outcome. The causes are multiple. Further studies are needed on this subject.

**Compliance with ethics regulations:** Yes in clinical research.

### FC-041 Factors affecting mortality of ICU hospitalized chest trauma patients

#### Olfa Turki, Rezk Ghorbel, Najeh Baccouch, Dorsaf Dlensi, Rania Ammar, Kamila Chtara, Chokri Benhmida, Mabrouk Bahloul, Mounir Bouaziz

##### Centre Hospitalo-universitaire Habib Bourguiba sfax, Sfax, Tunisie

**Correspondence:** Rezk Ghorbel - rezkghorbel3@gmail.com

*Annals of Intensive Care* 2021, **11(Suppl 1):**FC-041

**Rationale:** Blunt chest-wall trauma accounted for over 15% of all trauma admissions to emergency departments (EDs) and intensive care unit (ICU) worldwide. Blunt chest trauma is the second leading cause of death among trauma patients (4 to 60%). However, no current guidelines exist to assist in the management of this patients group. Early identification and aggressive management of blunt thoracic trauma is essential to reduce the significant rates of morbidity and mortality. The risk factors for mortality following blunt chest wall trauma have neither been well established nor summarized. We aimed to identify the risk factors of mortality in blunt chest wall trauma patients based on a prospective study in ICU

**Patients and methods/materials and methods:** We assessed clinical and para-clinical parameters predicting mortality in patients hospitalized in our ICU. We collected prospectively the demographic, clinical, laboratory, and echocardiography data of patients hospitalized for blunt chest trauma in a period of 6 months in the intensive care unit of our university hospital between June 2019 and December 2019.

**Results:** During the study period 54 patients were hospitalized for blunt chest trauma. The mean age was 32 ± 20 years with a sex ratio of 4.4. Blunt chest trauma was associated with brain trauma in 89% of cases. For the traumatic scores, the means of GCS, ISS and TSS were 9.4 ± 4.1; 31.6 ± 18 and 17.1 ± 3.9, respectively. Significant predictors of the development of complications and mortality on admission were TTS and ISS scores, sternal fracture, myocardial contusion (lower FEVG and positive troponin level). During the hospitalization, developing renal failure, hyperglycemia, acidosis and shock were associated with a poor outcome.

**Conclusion:** Blunt chest-wall trauma patients are often difficult to manage in ICUs, due to the frequent onset of delayed complications associated with a poor prognosis. Early identification of factors associated with a poor prognosis is essential to manage and reduce the significant rate of mortality.

**Compliance with ethics regulations:** Yes in clinical research.

### FC-042 Prognostication of chest trauma: validation of the Chest Trauma Scoring (CTS) system

#### Malek Khemili, Mourad Gahbiche

##### Surgical intensive care unit of Fattouma Bourguiba Hospital, Monastir, Tunisie

**Correspondence:** Malek Khemili - malek.khemili@yahoo.com

*Annals of Intensive Care* 2021, **11(Suppl 1):**FC-042

**Rationale:** Prediction of outcome in chest trauma patients by scoring system is of vital importance to predict morbidity, and therefore institute early intensive focussed care.

**Patients and methods/materials and methods:** This was a retrospective observational study done in our intensive care unit. We noted demographic parameters, history, vital parameters and necessary investigations including chest X-ray and chest CT-scan when patient was admitted in trauma unit. The CTS was composed of four different components with a point system assigned: age (< 45 years = 1, 45–65 = 2, > 65 = 3); pulmonary contusion (none = 0, unilateral minor = 1, bilateral minor = 2, unilateral major = 3, bilateral major = 4); number of rib fractures (< 3 = 1, 3–5 = 2, > 5 = 3); and the presence of bilateral rib fracture = 2. Number of rib fractures and pulmonary contusion were noted from chest X-ray and chest CT-scan. Each parameter has been assigned specific score and final score was calculated by adding scores of each parameter. Final CTS was then calculated which ranges from 2 to 12.

**Results:** Out of 32 patients, admitted from January 2016 to December 2019, 14 (43.8%) patients were younger than 45 years, and nine (28.2%) were older than 65 years. The mean ± SD age of the patients admitted with isolated chest trauma was 48.9 ± 22.4 years. An epidural analgesia have been place in 12 cases (37.5%) out of 32. Seven patients (21.9%) required mechanical ventilation. Association between high CTS (≥ 5) and mortality was not found to be statistically significant (Chi-square coefficient of 1.027 and *p*-value of 0.311). However, an area under the receiver operating characteristic curve (ROC) for mortality shows an acceptable value of 0.766 (Fig. 1). At CTS score 9 maximum sensitivity was 66.7% and specificity 84.6%.

**Conclusion:** CTS of at least 9 is associated with worse patient outcomes in our population. Increased vigilance is needed with trauma patients who present with a CTS > 9 at initial presentation. This simple scoring system may improve early identification of vulnerable patients and expedite therapeutic interventions.

**Compliance with ethics regulations:** N/A.
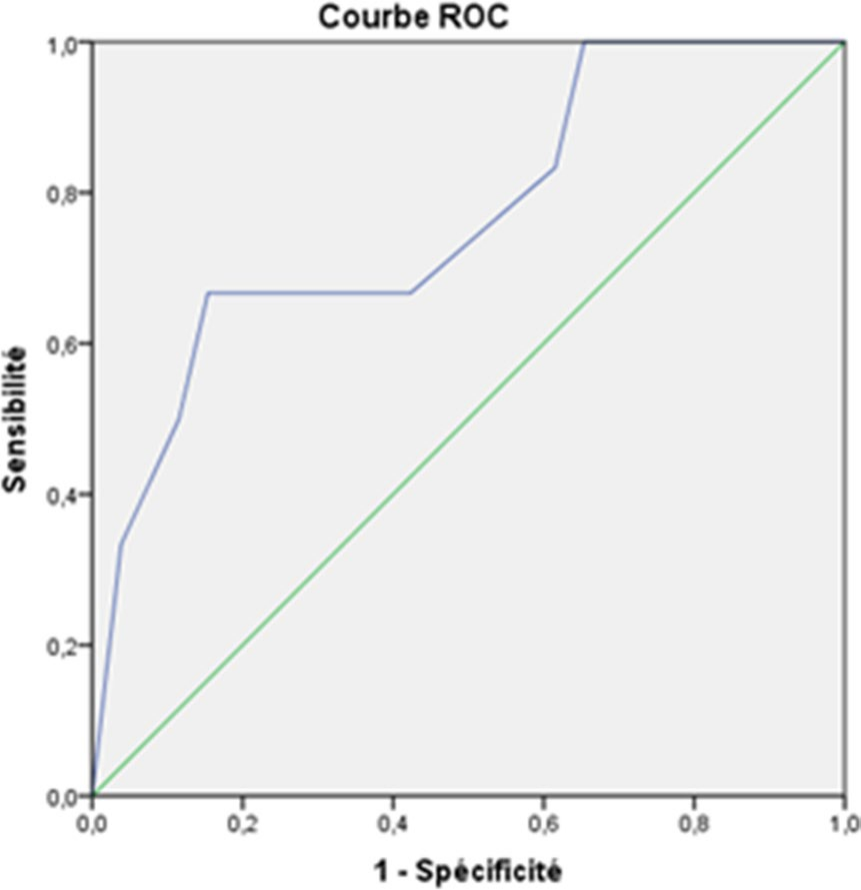


Fig. 1 Area under the receiver operating characteristic curve (ROC) for mortality

### FC-043 Prognosis of trauma patients over 65 years of age admitted to intensive care

#### Maha Ben Mansour, Ines Koubaa, Elhem Kaabia, Rafif Zoubeidi, Marwen Baccar, Rabeb Miled, Sawsen Chakroun, Faouzi Ben Salem, Mourad Gahbiche

##### CHU FATTOUMA BOURGUIBA MONASTIR, Monastir, Tunisie

**Correspondence:** Maha BEN Mansour - benmansour.maha@yahoo.fr

*Annals of Intensive Care* 2021, **11(Suppl 1):**FC-043

**Rationale:** Injuries are a major cause of death in all ages. They are accompanied by major morbidity with sometimes serious consequences both on a personal, family and social level, particularly for elderly patients whose number continues to increase with the increase in life expectancy. The aim of this study was to assess the prognosis and quality of life after multiple trauma in patients over 65 years.

**Patients and methods/materials and methods:** This is a retrospective study including all patients over 65 years of age admitted to surgical intensive care for multiple trauma between 2017 and 2020. Epidemiological data, the circumstances of the accident, the lesion assessment and morbi-mortality in intensive care and in long term were collected. The quality of life of survivors was determined using the qualitative VAS (0–100) before and at a distance from the trauma and the SF36 questionnaire collected by phone.

**Results:** During the study period, 72 patients were included. The median age was 73 (65–87) years, 86.2% lived at home, 70% of them were independent before the trauma. The most frequent injury mechanisms were a motorcyclist hit by a car in 43% of cases, and accidental falls in 28% of cases. Severe lesions involved 72% of patients with preferentially thoracic trauma in 67% of cases, cranial trauma in 52% of cases, trauma to the pelvis in 19% of cases and trauma to the spine in 17% of cases. Sixty-eight percent of patients presented at least one complication during their stay in intensive care, 38% had ventilator-acquired pneumonia. The mortality in our series was 39.5%. The factors influencing the prognosis and predictive of mortality were: age, cardiovascular and respiratory history, necessity of pre-hospital intubation, initial Glasgow score, and IGS and APACH scores in intensive care. Forty-three patients left the intensive care unit alive, 28 completely independent, 15 dependent on a third party, one of whom was in a vegetative state.

**Conclusion:** Multiple traumas in elderly patients are frequent in clinical practice, they are responsible for multiple multiple lesions and the occurrence of visceral failures endangering the patient’s vital and functional prognosis, especially in the presence of increasing morbidity with age. Multidisciplinary care and monitoring of patients even after their discharge improve their quality of life.

**Compliance with ethics regulations:** N/A.

### FC-044 Association between prehospital shock index variation and 28-day mortality among patients with septic shock

#### Romain Jouffroy^1,2^, Basile Gilbert^3^, Jean-Pierre Tourtier^4^, Emmanuel Bloch-Laine^5^, Patrick Ecollan^6^, Josiane Boularan^7^, Vincent Bounes^3^, Benoit Vivien^2^, Papa Gueye^8^

##### ^1^APHP - Hôpital Ambroise Paré - Service de Médecine intensive et réanimation, Boulogne Billancourt, France; ^2^APHP - Hôpital Necker enfants malades - SAMU, Paris, France; ^3^Department of Emergency Medicine, SAMU 31, University Hospital of Toulouse, Toulouse, France; ^4^Paris Fire Brigade, Paris, France; ^5^Emergency Department, Cochin Hospital, Paris, France & Emergency Department, SMUR, Hôtel Dieu Hospital, Paris, France; ^6^Intensive Care Unit, SMUR, Pitié Salpêtrière Hospital, Paris, France; ^7^SAMU 31, Castres Hospital, Castres, France; ^8^SAMU 972 CHU de Martinique Pierre Zobda Quitman Hospital, Fort De France - Martinique, France

**Correspondence:** Romain Jouffroy - romain.jouffroy@aphp.fr

*Annals of Intensive Care* 2021, **11(Suppl 1):**FC-044

**Rationale:** Septic shock (SS) hyperdynamic phase is characterized by tachycardia and low-blood pressure reflecting the relative hypovolemia. Shock index (SI), a simple objective tool, is usable for hypovolemia severity and SS prognosis assessment. The aim of this study was to evaluate the relationship between prehospital SI variation and 28-day mortality of SS initially cared for in prehospital setting by a mobile intensive care unit (mICU).

**Patients and methods/materials and methods:** From April 6th, 2016 to December 31st, 2020, 406 patients with SS requiring prehospital mICU were retrospectively analysed. Initial SI, i.e. first measurement after mICU arrival to the scene, and final SI, i.e. last measurement of the prehospital stage, were used to calculate delta SI (initial SI − final SI) defining positive and negative delta SI. A survival analysis after propensity score matching was used to compare the 28-day mortality of SS with positive/negative delta SI.

**Results:** Pulmonary, digestive and urinary infections were the suspected cause of the SS in 42%, 25% and 17%, respectively. The 28-day overall mortality was 29%. Cox regression analysis revealed a significant association between 28-day mortality and negative delta SI with an adjusted hazard ratio (HRa) of 1.88 [1.07–3.31] (*p *= 0.03) and positive delta SI: HRa = 0.53 [0.30–0.94] (*p *< 10^−3^).

**Conclusion:** In this study, we report that prehospital hemodynamic delta SI among SS cared for by a mICU is associated with 28-day mortality. A negative prehospital delta SI helps to identify SS with higher risk of 28-day mortality rate.

**Compliance with ethics regulations:** Yes in clinical research.
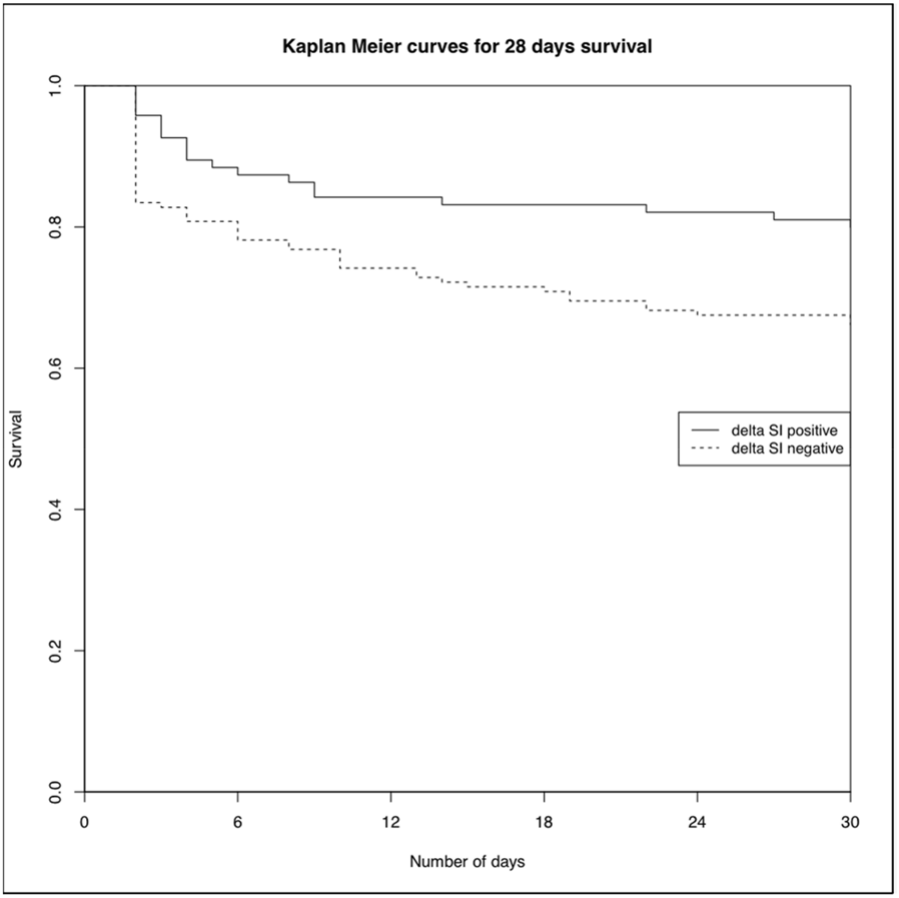


Fig. 1 Kaplan–Meier curves of 28-day survival between patients with negative delta SI and those with positive delta SI

### FC-045 Increase in the diameter of the subclavian vein according to the arm’s position: a preliminary ultrasound study

#### Walid Sellami, Ines Ben Mrad, Iheb Labbene, Mustapha Ferjani

##### Department of critical care medicine and anesthesiology, Military Hospital of Tunis, Tunisia, Tunis, Tunisie

**Correspondence:** Walid Sellami - drsellamiwalid@yahoo.fr

*Annals of Intensive Care* 2021, **11(Suppl 1):**FC-045

**Rationale:** The cannulation of the subclavian vein may seem difficult due to the proximity of the clavicle and the pleura. Changing the arm position amends the anatomical relationship between the clavicle and the vein and could amend its access. To confirm this hypothesis, we conducted a preliminary anatomical study on the hospitalized patients. The aim of this study was to compare the subclavian vein diameter in two arm positions: neutral position (PN) and 90° abduction and 90° external rotation position (ARE).

**Patients and methods/materials and methods:** This prospective study was conducted during 3 months. Patients older than 18 years, hospitalized consecutively in the ICU were included. A unique expert operator performed the ultrasound exams with a vascular probe. The probe was placed perpendicularly to the skin, parallel to the clavicle in its median third portion. The measures were realized for the right side exclusively. At each time, after obtaining a cross section of the vein, the depth and the diameter of the vein were measured, as well as the distances vein–pleura and vein–artery.

**Results:** Thirty-five patients were included. The median age of patients was 62 [18–72] years. The subclavian vein diameter increased significantly after the arm mobilization (11 mm vs. 14 mm, *p *= 0.03). The artery, the pleura and the brachial plexus were viewed as frequently in both positions without a significant difference in the distance between these structures and the subclavian vein (*p *= 0.06).

**Conclusion:** Changing the position of the arm in 90° abduction and 90° external rotation enhances significantly the cross section area of the vein which can make the ultrasound-guided cannulation easier and safer.

**Compliance with ethics regulations:** Yes in clinical research.

### FC-046 History of hypertension but not diabetes is associated with intrahospital mortality in patient presenting to the emergency department for dyspnea

#### Manel Kallel, Khadija Zaouche, Dhouha Nsib, Radhia Boubaker, Hamida Maghraoui

##### RABTA HOSPITAL, Tunis, Tunisie

**Correspondence:** Manel Kallel - DR.MANEL.KALLEL@GMAIL.COM

*Annals of Intensive Care* 2021, **11(Suppl 1):**FC-046

**Rationale:** Mortality in emergency departments (ED) is still very high in developing countries. Identifying factors associated with mortality allows defining areas of prevention and establishing protocols of management. The objective of this study was to report some of the factors influencing mortality in critically ill patients presenting to the ED.

**Patients and methods/materials and methods:** We conducted a 1-year retrospective study including adult patients visiting ED for acute dyspnea. Logistic regression was developed to study factors predictive of mortality in these patients.

**Results:** We included 1389 patients. Their mean age was 60 ± 17 years, their sex ratio was 1.4 and 41% of them were active smokers. Most frequent comorbidities were hypertension (37%), diabetes, (30%), COPD (20%), dyslipidemia (14%), history of stroke (6%), atrial fibrillation (12.5%) and renal failure (9.5%). We recorded 202 deaths, i.e. an intra-hospital mortality rate of 14.5%. Septic shock (48%) was the most common cause of death followed by acute cardiac failure with cardiogenic shock (30%). Death occurred after an average stay of 75 ± 18 h. In univariate analysis, renal failure (OR = 0.55 [0.35–0.85], *p *= 0.034) and hypertension (OR = 0.6 [0.4–0.8], *p *= 0.004) were significantly associated with intra-hospital mortality. In contrast, diabetes was not associated with death (*p *= 0.058), nor was COPD (*p *= 0.54).

**Conclusion:** Hypertension and renal failure are significantly associated with mortality in patient visiting emergency department for dyspnea

**Compliance with ethics regulations:** Yes in clinical research.

### FC-047 Acetate-free biofiltration dramatically improves the metabolic and hemodynamic tolerance of intermittent hemodialysis in critically ill patients

#### Anna Gouin, Pierre Tailpied, Laurence Lavayssiere, Chloé Medrano, Marie-Béatrice Nogier, Olivier Cointault, Stanislas Faguer

##### Centre Hospitalier Universitaire de Toulouse, Toulouse, France

**Correspondence:** Stanislas Faguer - stanislas.faguer@inserm.fr

*Annals of Intensive Care* 2021, **11(Suppl 1):**FC-047

**Rationale:** There is an unmet need to develop easy-to-use techniques of renal replacement therapy with better metabolic and hemodynamic tolerance, especially in critically ill patients. Acetate-free biofiltration (AFB) was developed in patients receiving chronic intermittent hemodialysis (IHD). AFB is characterized by bicarbonate-free (and thereafter acid buffer-free) dialysate with post-filter bicarbonate reinjection. The lack of acid buffer in the dialysate may improve the hemodynamic tolerance compared to bicarbonate-IHD (among other, owing to the lack of acid buffer-induced CO_2_ increase in the blood returning to patients), but data in critically ill patients are scarce.

**Patients and methods/materials and methods:** Collection of hemodynamic parameters (blood pressure, heart rate) every 30 min of the dialysis sessions (AFB or B-IHD). Blood gauzes collected before and after dialysis filter, and before and after sessions. Study was approved by national Ethical Committee.

**Results:** We prospectively included 41 mechanically ventilated patients who received 4-h sessions of AFB (*n* = 61) or B-IHD (*n* = 50). Characteristics at baseline were similar between the two techniques. Blood flow and bicarbonate reinjection were settled at 250–300 mL/min and bicarbonate reinjection 2–2.3 L/h, respectively. The number of episodes of intra-dialytic hypotension was significantly lower in AFB compared to B-IHD (number of mean BP decrease > 10 mmHg: 1.84 ± 2.2 vs. 2.9 ± 2.6, *p *= 0.02; systolic BP decrease > 20 mmHg, 1.34 ± 2.2 vs. 2.2 ± 2.6, *p* = 0.02) (Fig. 1). Mean dose of norepinephrine was stable in B-IHD and slightly decreased in AFB. Mean PvCO_2_, bicarbonate concentration and pH after the dialysis filter (i.e. in blood returning to the patients) were 60 ± 4.4, 32 ± 2.3 and 7.34 ± 0.04 in B-IHD compared to 45 ± 9.6, 24 ± 4.9 and 7.35 ± 0.07 in AFB (*p *< 0.001, *p *< 0.001 and *p *= 0.79, respectively). The mean CO_2_ removal with bicarbonate zero dialysate was 120 mL/min. The final CO_2_ load (i.e. [total CO_2_ content “after” minus “before” filter]*blood flow) induced by AFB was 70% lower than with B-IHD.

**Conclusion:** AFB is safe in critically ill patients and is better tolerated than usual bicarbonate-IHD, mainly due to increased diastolic BP suggesting optimization of arterial tone. Euhydric hypercapnia in blood returning from B-IHD probably accounts for hemodynamic instability that is significantly fewer observed with AFB. In-depth characterization of systemic and pulmonary hemodynamic changes (especially, pulmonary arterial resistances) during AFB and B-IHD sessions is warranted.

**Compliance with ethics regulations:** Yes in clinical research.
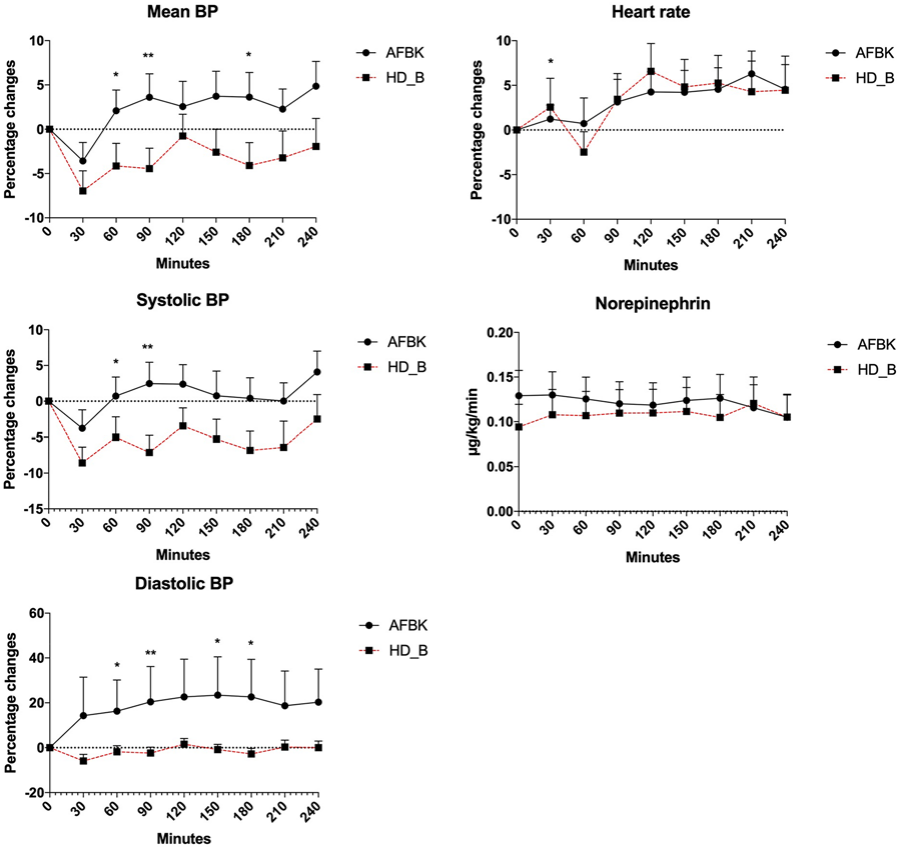


Fig. 1 Hemodynamic parameters during dialysis sessions with acetate-free biofiltration (AFBK) or hemodialysis with bicarbonate buffer (HD-B) in critically ill patients receiving mechanical ventilation. BP, blood pressure.

### FC-048 “Intermittent veno-venous hemodia-dialysis”: a new approach for renal replacement therapy in the ICU setting

#### Noémie Zucman, Charles Verney, Fabrice Uhel, Jean-Damien Ricard, Damien Roux

##### Hôpital Louis Mourier, APHP, Colombes, France

**Correspondence:** Noémie Zucman - noemie.zucman@gmail.com

*Annals of Intensive Care* 2021, **11(Suppl 1):**FC-048

**Rationale:** In critically ill patients with acute kidney injury (AKI), optimal renal replacement therapy (RRT) modality remains debated. Continuous and intermittent techniques can be used equally, based on availability and medical experience (1). Intermittent techniques require a water treatment system that limits its use and increases the cost. Here, we describe a new intermittent approach we called “intermittent veno-venous hemodia-dialysis” (IVVHDD) which prevents these limitations.

**Patients and methods/materials and methods:** We used a hemodiafiltration machine (MultiFiltrate Pro^®^, Fresenius Medical Care) with “Post-dilution CVVHDF” set to deliver intermittent RRT. We connected the replacement pump with the dialysis pump using a T-union to double the dialysate flow. This allows to increase diffusive transfers and suppress filtration. Schematic representations of extracorporeal circuits of IVVHDD (compared with conventional post-dilution CVVHDF) are shown in Fig. 1. Standard initial settings were dialysate flow of 9000 ml/h (4500 ml/h per pump), blood flow of 250 ml/min, and ultrafiltration as required, up to 1000 ml/h.

**Results:** We report preliminary results of six patients who underwent a total of 18 IVVHDD sessions in our ICU. Median age was 67, two patients had end-stage kidney disease requiring chronic dialysis. Four patients presented with AKI on ICU admission. Median [IQR] duration of IVVHDD sessions was 3.9 [2;4] h. At time of initiation, median plasma urea and creatinine levels were 40.6 [30.2; 53] mmol/L and 478 [348; 781] µmol/L, respectively. Median plasma urea reduction ratio was 38 [29.1; 48.6] %. Hemodynamic impairment requiring fluid infusion or catecholamine increase occurred in 11% of sessions. No dysnatremia, hypokalemia or hypophosphatemia was observed. Other adverse events were scarce but emphasize the need for staff training and standardized procedures.

**Conclusion:** In conclusion, our technique is a proof-of-concept of intermittent RRT using the MultiFiltrate Pro^®^ machine, requiring no water treatment system. IVVHDD is an interesting alternative to traditional RRT modalities, especially as logistic and financial considerations are at stake.

**Reference**

1. Vinsonneau C et al.Renal replacement therapy in adult and pediatric intensive care: Recommendations by an expert panel from the French Intensive Care Society (SRLF) with the French Society of Anesthesia Intensive Care (SFAR) French Group for Pediatric Int.

**Compliance with ethics regulations:** Yes in clinical research.
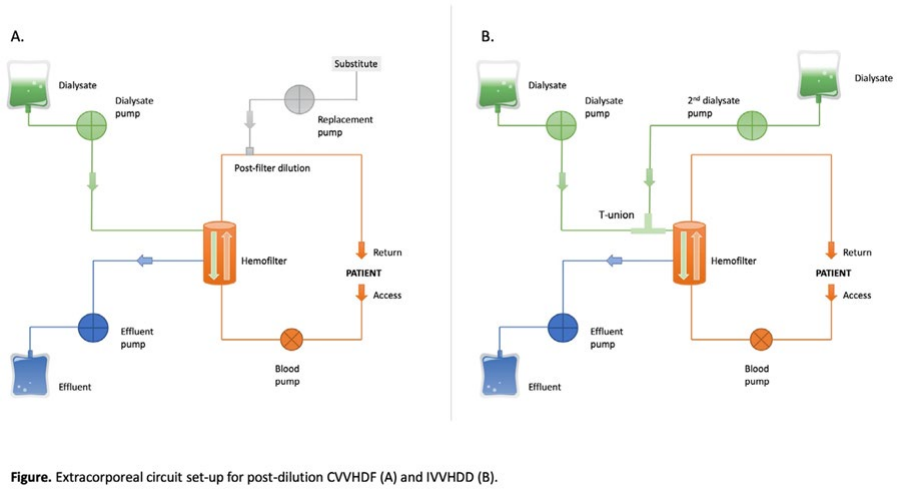


Fig. 1 CVVHDF: continuous veno-venous hemodiafiltration; IVVHDD: intermittent veno-venous hemodia-dialysis

### FC-049 Renal replacement therapy for acute kidney injury in the French intensive care unit: a nationwide survey of practices

#### Jean-Pierre Quenot^1^, Idris Amrouche^1^, Jean-Yves Lefrant^2^, Kada Klouche^3^, Samir Jaber^4^, Damien Du Cheyron^5^, Jacques Duranteau^6^, Julien Maizel^7^, Eric Rondeau^8^, Etienne Javouhey^9^, Theophile Gaillot^10^, René Robert^11^, Jean Dellamonica^12^, Bertrand Souweine^13^, Julien Bohé^14^, Saber Davide Barbar^15^, Caroline Sejourné^16^, Christophe Vinsonneau^16^

##### ^1^CHU Dijon, Dijon, France; ^2^CHU, Pôle Anesthésie Réanimation Douleur Urgence, Nîmes, France; ^3^Lapeyronie Hospital University Hospital and INM University Montpellier, INSERM, Montpellier, France; ^4^University of Montpellier Saint Eloi Hospital, and PhyMedExp, University of Montpellier, INSERM, CNRS, Montpellier, France; ^5^CHU de Caen Normandie, Service de Réanimation Médicale, Caen, France; ^6^Hôpitaux Universitaires Paris Sud, Université Paris-Sud, Université Paris-Saclay, Hôpital de Bicêtre, Assistance Publique Hôpitaux de Paris, Kremlin Bicêtre, France; ^7^Amiens University Hospital, Amiens, France; ^8^AP-HP, Hôpital Tenon, Department of Nephrology and Transplantation, INSERM UMR-S 1155 et Urgences Néphrologiques et Transplantation rénale, Sorbonne Université, Paris, France; ^9^Hospices Civils de Lyon, et University Claude Bernard Lyon 1, Lyon, France; ^10^CHU de Rennes, et CIC-P Inserm 0203 Université Rennes, Rennes, France; ^11^CHU La Milétrie, Poitiers, France; ^12^l’Archet Hospital, University Hospital of Nice, Nice, France; ^13^CHU de Clermont-Ferrand, Clermont-Ferrand, France; ^14^Centre Hospitalier Lyon-Sud, Hospices Civils de Lyon, Pierre Bénite, France; ^15^Centre Hospitalier Universitaire, Nîmes, France; ^16^Hôpital de Bethune, Bethune, France

**Correspondence:** Jean-Pierre Quenot - jean-pierre.quenot@chu-dijon.fr

*Annals of Intensive Care* 2021, **11(Suppl 1):**FC-049

**Rationale:** The frequency of acute kidney injury (AKI) can be as high as 50% in the intensive care unit (ICU). Despite the publication of national guidelines in France in 2015 for the use of RRT, there are no data describing the implementation of these recommendations in real-life.

**Patients and methods/materials and methods:** We performed a nationwide survey of practices from 15/11/2019 to 24/01/2020 in France. An electronic questionnaire based on the items recommended in the national guidelines was sent using an online survey platform, to the chiefs of all ICUs in France.

**Results:** We identified 356 eligible ICUs, from which 88 (24.7%) Department Chiefs and 232/285 physicians (82%) completed the questionnaire. Intermittent RRT was first-line choice in > 75% in a patient with single organ (kidney) failure at the acute phase, whereas continuous RRT was predominant (> 75%) in patients with septic shock or multi-organ failure. Blood and dialysate flow for intermittent RRT were 200–300 ml/min and 400–600 ml/min, respectively. The dose of dialysis for continuous RRT was 25 to 35 ml/kg/h (65%). Insertion of the dialysis catheter was mainly performed by the resident under echographic guidance, in the right internal jugular vein. The most commonly used catheter lock was citrate (53%). The most frequently cited criterion for weaning from RRT was diuresis, followed by a drop in urinary markers (urea, creatinine).

**Conclusion:** This study shows a satisfactory level of compliance with French guidelines and recent scientific evidence regarding initiation of RRT for AKI in the ICU.

**Compliance with ethics regulations:** Yes in clinical research.

### FC-050 Comparison between regional citrate anticoagulation and heparin for intermittent hemodialysis in ICU patients: a propensity score-matched cohort study

#### Christophe Leroy, Bruno Pereira, Edouard Soum, Claire Bachelier, Elisabeth Coupez, Laure Calvet, Konstantinos Bachoumas, Claire Dupuis, Bertrand Souweine, Alexandre Lautrette

##### CHU Montpied, Clermont-Ferrand, France

**Correspondence:** Alexandre LAUTRETTE (alautrette@chu-clermontferrand.fr)

*Annals of Intensive Care* 2021, **11(Suppl 1):**FC-050

**Rationale:** Regional citrate anticoagulation (RCA) is the gold standard of anticoagulation for continuous renal replacement therapy, but is rarely used for intermittent hemodialysis (IHD) in ICU. Few studies assessed the safety and efficacy of RCA during IHD in ICU, however, no data is available comparing RCA to heparin anticoagulation, which is commonly used for IHD. The aim of this study was to assess the efficacy and safety of RCA compared to heparin anticoagulation during IHD.

**Patients and methods/materials and methods:** This retrospective single-center cohort study included consecutive ICU patients treated with either heparin anticoagulation (unfractionated or low-molecular-weight heparin) or RCA for IHD from July to September in 2015 and 2017. RCA was performed with citrate infusion according to blood flow and calcium infusion by diffusive influx from dialysate. Using a propensity score analysis, as the primary endpoint we assessed whether RCA improved efficacy, quantified with Kt/V from the ionic dialysance, compared to heparin anticoagulation. The secondary endpoint was safety. Exploratory analyses were performed on the changes in efficacy and safety between the implementation period (2015) and at long-term (2017).

**Results:** In total, 208 IHD sessions were performed in 56 patients and were compared (124 RCA and 84 heparin coagulation). There was no difference in Kt/V between RCA and heparin (0.95 ± 0.38 vs. 0.89 ± 0.32; *p* = 0.98). A higher number of circuit clotting (12.9% vs. 2.4%; *p* = 0.02) and premature interruption resulting from acute high transmembrane pressure (21% vs. 7%; *p* = 0.02) occurred in the RCA sessions compared to the heparin sessions. In the propensity score matching analysis, RCA was associated with an increased risk of circuit clotting (absolute differences = 0.10, 95%CI [0.03–0.18]; *p* = 0.008). There was no difference in efficacy and safety between the two time periods (2015 and 2017).

**Conclusion:** RCA with calcium infusion by diffusive influx from dialysate for IHD was easy-to-implement with stable long-term efficacy and safety, but did not improve efficacy and could be associated with an increased risk of circuit clotting compared to heparin anticoagulation in non-selected ICU patients. Randomized trials to determine the best anticoagulation for IHD in ICU patients should be conducted in a variety of settings.

**Reference**

1. Accepted in Annals of Intensive Care (January 2021).

**Compliance with ethics regulations:** Yes in clinical research.

### FC-051 Early hypertension after withdrawal of norepinephrine in critically ill septic patients may have an impact on the occurrence of acute kidney disease

#### Antoine Dewitte, Pierre Khan, Younès El Boustani, Arthur Orieux, Renaud Prevel, Didier Gruson, Sébastien Rubin, Alexandre Boyer

##### CHU de Bordeaux, Bordeaux, France

**Correspondence:** Antoine DEWITTE (antoine.dewitte@chu-bordeaux.fr)

*Annals of Intensive Care* 2021, **11(Suppl 1):**FC-051

**Rationale:** The occurence of hypertension after withdrawal of vasopressor is poorly described in intensive care units (ICU), but appears to be frequent (1). Hypertension could result in a second injury to the kidney following septic shock (2). The objectives of this study were to describe the incidence of early hypertension after norepinephrine withdrawal and investigate its association with acute kidney disease (AKD).

**Patients and methods/materials and methods:** Retrospective cohort including 368 patients admitted for septic shock in two separate intensive care units of a university hospital. Hypertension was defined by the introduction of an intravenous antihypertensive maintained at least 24 h or by a 24-h average blood pressure > 140/90 mmHg occurring within 7 days of norepinephrine withdrawal. Acute kidney injury (AKI) was defined according to KDIGO criteria. AKD was defined by recovery within 7 to 90 days. Secondary outcomes were Major Adverse Kidney Events within 30 days (MAKE30), death, use of RRT and renal recovery at ICU discharge. An adjusted Cox proportional-hazards regression model was applied to determine hazard ratios (HR).

**Results:** Hypertension occurred in 234 (64%) patients. Among them, 160 (68%) patients were treated, in 87% of cases with α1-adrenoceptor antagonist (urapidil) and/or calcium channel blockers (nicardipine) by continuous intravenous administration. Only 46 (28%) of the patients had their blood pressure controlled on the third day after hypertension. Early hypertension was associated with AKD (HR (95% CI): 1.5 (1.1–2.1); *P* = 0.005), RRT (HR: 1.4 (1.1–1.9); *P* = 0.01) and MAKE30 (HR: 1.4 (1–1.9); *P* = 0.03) in a fully adjusted model (Table 1). Hypertensive patients with a coefficient of systolic blood pressure variation > 20% presented a significantly higher risk of AKD (HR: 1.7 (1.2–2.5); *P* = 0.002).

**Conclusion:** Early hypertension is a common phenomenon after withdrawal from norepinephrine, with rare blood pressure control. Its independent association with AKD raises the question of its significance as second renal injury after septic shock. The interest of optimizing antihypertensive treatment in a bundle of nephroprotective measures in ICU should be confirmed in future studies.

**References**

1. Gaci R et al. Early hypertension after vasopressor weaning during septic shock: associated factors and prognostic significance. Minerva Anestesiol. 2018; 84:196–203.
2. Vanmassenhove J et al. Management of patients at risk of acute kidney injury. Lancet. 2017; 389:2139–51.

**Compliance with ethics regulations:** Yes in clinical research.

Table 1. Hazard Ratios for hypertensive patients after norepinephrine withdrawal and Acute Kidney Disease, MAKE^30^, Death, Renal Replacement Therapy and Renal Recovery.
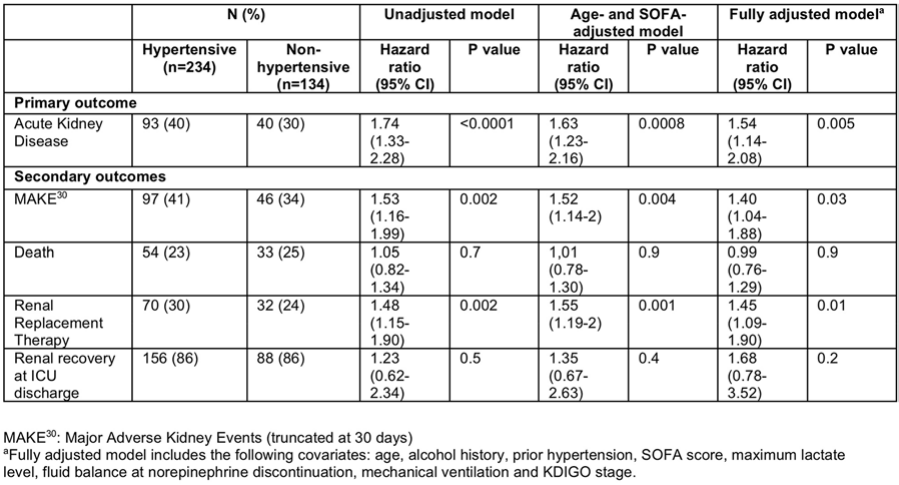


### FC-052 Severe COVID-19 induced AKI: a 3-month follow-up

#### Arthur Orieux, Sébastien Rubin, Renaud Prevel, Antoine Garric, Didier Gruson, Alexandre Boyer

##### CHU de Bordeaux, Bordeaux, France

**Correspondence:** Arthur Orieux - arthur.orieux@chu-bordeaux.fr

*Annals of Intensive Care* 2021, **11(Suppl 1):**FC-052

**Rationale:** COVID-19 is mainly responsible for pneumonia with severe respiratory damages like acute respiratory distress syndrome (ARDS) and could require prolonged invasive mechanical ventilation in the intensive care unit (ICU). Other organ injuries, probably underestimated, were reported like acute kidney injury (AKI) with an incidence upper than 50%. To date, no data are reported concerning renal outcome after COVID-19-induced AKI.

**Patients and methods/materials and methods:** We carried out a 3-month follow-up study of patients admitted to a medical ICU. A standardized blood and urine laboratory test to be performed at 3 months were provided to the patient upon discharge from ICU.

**Results:** From 5th Mars to 2nd May 2020, 57 patients were admitted. Among them, 8 died during the hospitalization, 7 patients were lost to follow-up and 2 patients with previous chronic kidney disease (CKD) (eGFR < 60 mL/min/1.73 m^2^) before admission were excluded. Among the 40 patients analyzed, 27 (68%) suffered from AKI during their stay in ICU. The mean age was 61 ± 11, the mean SAPSII score was 37 ± 17. 19/27 (70%) and 20/27 (74%) required, respectively, vasopressor and mechanical ventilation. Basal serum creatinine (SCr) was 66 ± 14 μmol/L; basal eGFR was 98 ± 12 mL/min/1.73m2 (Table 1). 9/27 patients (33%) had an AKI KDIGO stage I; 9/27 (33%) an AKI stage II and 9/27 patients (33%) AKI stage 3. Among them, 4/9 (44%) required renal replacement therapy (RRT). At 3 months of follow-up, SCr was 80 ± 28 μmol/L and eGFR was 84 ± 21 ml/min/1.73 m^2^. Urine protein/creatinine ratio was 9 [5–34] mg/mmol without hematuria or glycosuria. 20/27 (74%) AKI patients had an early recovery in the first 7 days (SCr < 125% of baseline) and 7/27 (26%) had acute kidney disease (AKD). 3/7 (43%) AKD patients recovered within the 3 months follow-up. Renal recovery (SCr < 125% baseline SCr) at D90 was presented by 23/27 (85%) patients. 4/27 (15%) patients suffered from new onset CKD (3 patients required RRT during ICU hospitalization). All these new CKD patients suffered from AKD after AKI and prior to CKD. No patient needed RRT at 90 days of follow-up. MAKE criteria at 90 days was found in 4/27 (15%) patients, due exclusively to the need of RRT during ICU hospitalization.

**Conclusion:** Because of the specificity of COVID-19-induced AKI associated with a highly inflammatory state, a higher incidence of CKD at 3 months could be expected. However, this report seems to be reassuring and the low proteinuria supported this hypothesis. Nevertheless, prolonged and careful follow-up is necessary with larger multicenter studies.

**Compliance with ethics regulations:** Yes in clinical research.
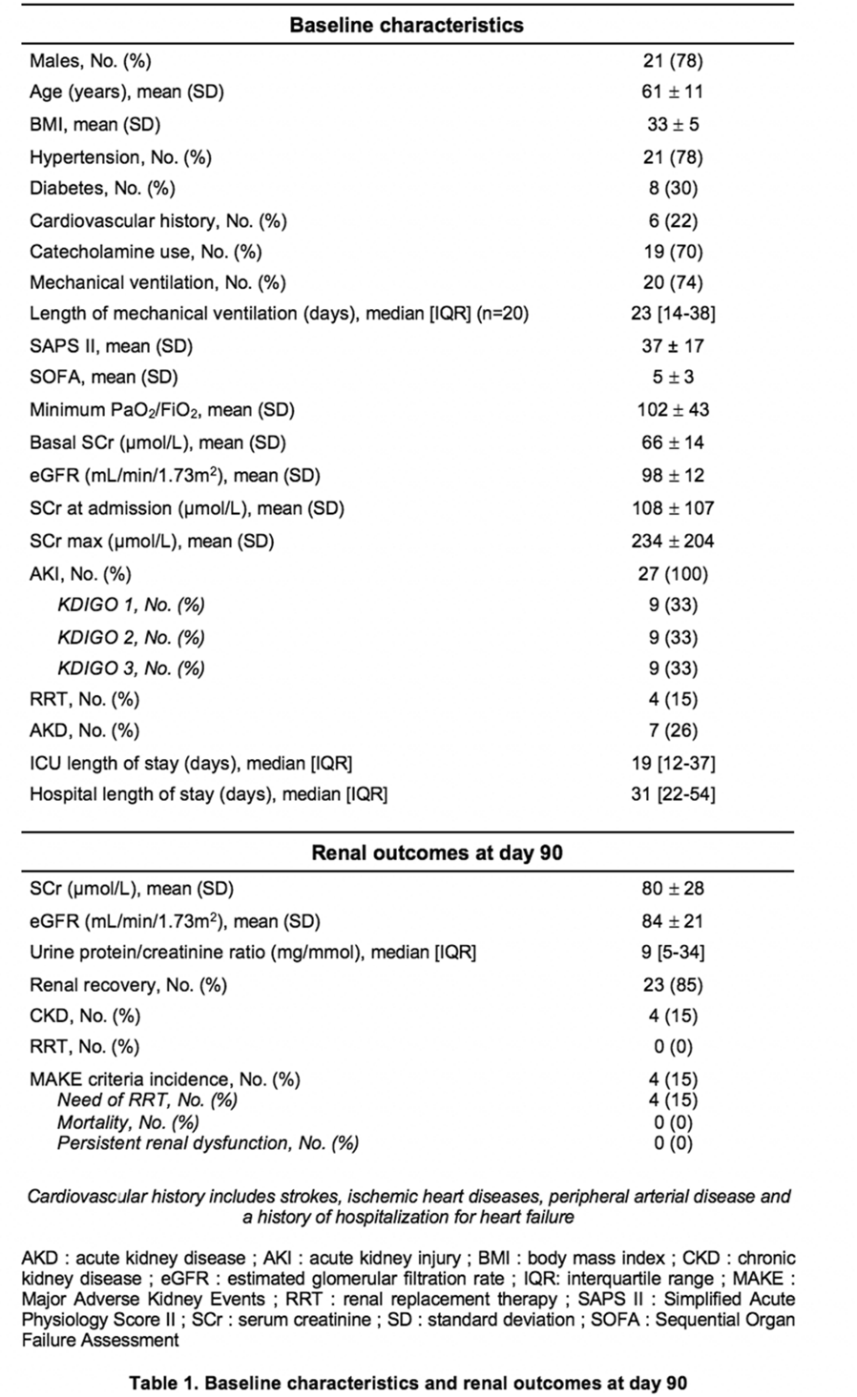


Table 1 Baseline characteristics and renal outcomes at day 90

### FC-053 Comparison of four-channeled videolaryngoscopes to Macintosh laryngoscopy for simulated intubation of critical ill patients: a randomized study: the MACMAN2 Study

#### Paul Decamps, Nicolas Grillot, Aurélie Le Thuauta, Noelle Brule, Corinne Lejus, Jean Reignier, Jen-Baptiste Lascarrou

##### CHU de Nantes, Nantes, France

**Correspondence:** Paul Decamps - paul.decamps@hotmail.fr

*Annals of Intensive Care* 2021, **11(Suppl 1):**FC-053

**Rationale:** Videolaryngoscopes with an operating channel could improve the success rate of intubation for critical ill patients. We aim to compare 4-channeled videolaryngoscope to Macintosh standard laryngoscopy during intubation on high-fidelity simulation mannikin.

**Patients and methods/materials and methods:** The MACMAN 2 study was a randomized, open-label, single-center trial on a high-fidelity simulator. Recruitment was carried out among anesthesia and intensive care residents in their first and second year, emergency medicine residents in their first to third year and medical specialty residents who had completed at least one rotation in ICU.

**Results:** In this study with high-fidelity simulation mannikin, the success rate at first laryngoscopy was 97.5% [95% CI 91.1–99.7] for Airtraq™, KingVision™ and Pentax AWS200™, 92.4% [95% CI 84.2–97.2] for the VividTrac VT-A100™ and 70.9% [95% CI 59.6–80.6] for the Macintosh laryngoscope. The success rate for the Macintosh blade was significantly lower compared to the other devices with for Airtraq™, KingVision™ and Pentax AWS200™ (70.9% vs 97.5%; *p* < 0.0001) and to the VividTrac VT-A100™ (70.9% vs 92.4%; *p* < 0.0001).

**Conclusion:** Airtraq™, KingVision™ and Pentax AWS200™ channeled videolaryngoscope expected high rate of success for simulated intubated of critical ill patients. A multicentric, controlled, randomized clinical study comparing channeled videolaryngoscope to direct laryngoscopy can include one of those videolaryngoscope studied with lower boundary of first pass success above 90%.

**Compliance with ethics regulations:** Yes in clinical research.

### FC-054 Accuracy of tidal volume delivery in volume control ventilation from open source and intermediate ventilators in the COVID-19 pandemic

#### Nicolas Terzi^3^, Adrien Farrugia^4^, François Charbon^1^, Florian Degivry^1^, Fabrice Rastello^5^, Christophe Déhan^6^, Cyril Fromentin^7^, Martin Cour^1^, Laurent Argaud^1^, Bruno Louis^2^, Claude Guérin^1,2^

##### ^1^Hospices Civils de Lyon, Lyon, France; ^2^Institut Mondor De Recherches Biomédicales, Créteil, France; ^3^CHUGA, Grenoble, France; ^4^SteadXP, Grenoble, France; ^5^INRIA, Grenoble, France; ^6^MinMaxMedical, Saint Martin D’hères, France; ^7^FineHeart, Pessac, France

**Correspondence:** Claude Guérin - claude.guerin@chu-lyon.fr

*Annals of Intensive Care* 2021, **11(Suppl 1):**FC-054

**Rationale:** The quick release of cheap open source ventilators and the use of intermediate ventilators dedicated to step-down or emergency units were two examples among the strategies to overcome the anticipated shortage in Intensive Care Unit (ICU) ventilators during the current COVID-19 pandemic. Present bench study aims at testing the accuracy of tidal volume (V_T_) delivery by one open source, five intermediate and one ICU ventilators. Our hypothesis was that the open source ventilator performed as good as the ICU ventilator and the intermediate ventilators in-between them.

**Patients and methods/materials and methods:** Seven ventilators were investigated: the open source (e-SPIRO), five intermediate (T60, T75, EOV, SV300, Osiris), and one ICU (SV600). The e-SPIRO operated through the mechanical compression of an Ambu bag by two horizontally pivoting arms equipped with 3D-printed jaws moved by a stepper motor. The expiratory valve uses a low pressure electromagnetic control valve generated by the bag pressure itself during insufflation. Positive end-expiratory pressure (PEEP) is generated by a tube dipping in water at the set level controlled by the stepper motor. The overall system is hosted in two plastic boxes (total 48 × 40 × 60cm weighting 27 kg). Alarms, touch-screen interface and manual end-expiratory and end-inspiratory airway occlusions complete the set-up. A robust control is achieved by independent software and state machine running on a STM-32 controller. Each ventilator was set in volume control, respiratory rate 20 breaths/min, inspiratory time 0.8 s, FIO2 21%. We tested three VT (300, 400 and 500 ml), each at 5, 10 and 15 cmH2O PEEP. Each ventilator was connected to an ASL5000 lung model set in passive condition with three combinations of resistance (R in cmH2O/L/s) and compliance (C in ml/cmH2O) of 10/50, 20/60, 10/40 aimed at mimicking normal, chronic obstructive pulmonary disease and acute respiratory distress syndrome clinical conditions, respectively. Same circuit was used, including High Efficiency Particulate Air (Iso-Gard HEPA light) at both inspiratory and expiratory valve inlets and one Humid-Vent HEPA at the ASL5000 inlet. V_T_ error was computed as ((set *V*_*T*_ − measured *V*_*T*_)/set *V*_*T*_)*100 and was assessed across the 10% accuracy boundaries.

**Results:** The median VT error was within the 10% accuracy window in all of the ventilators, including the Open Source (e-SPIRO), but the Osiris (Fig. 1). This latter under-delivered V_T_ at each RC condition (Fig. 1).

**Conclusion:** In present bench conditions the open source and the intermediate ventilators, except for the Osiris3 ventilator, can deliver VT as accurately as the ICU ventilator.

**Compliance with ethics regulations:** N/A.
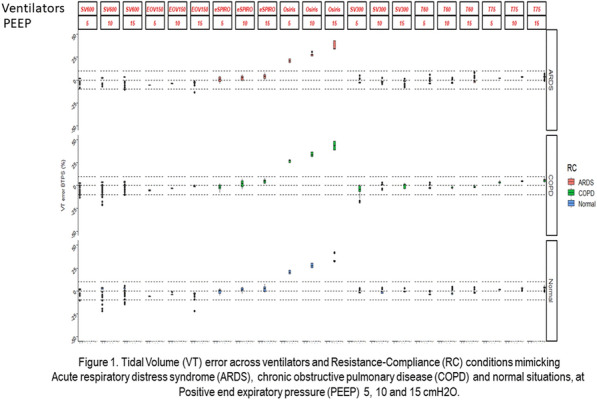


Fig. 1 Tidal volume (VT) error across ventilators and resistance–compliance (RC) conditions mimicking acute respiratory distress syndrome (ARDS), chronic obstructive pulmonary disease (COPD) and normal situations, at positive end-expiratory pressure (PEEP) 5, 10 and 15 cmH2O

### FC-055 Reliability and limits of transport ventilators to safely ventilate severe patients in special surge situations

#### Dominique Savary^1^, Arnaud Lesimple^3^, Nathan Prouvez^3^, François Beloncle^2^, François Morin^1^, François Templier^1^, Christophe Desprez^2^, Alexandre Broc^3^, Laurent Brochard^5^, Jean-Christophe Richard^2,3^, Alain Mercat^2^

##### ^1^Département de Médecine d’Urgence, Centre Hospitalier Universitaire d’Angers, Vent’Lab, Univ Angers, Angers, France; ^2^Département de Médecine Intensive-Réanimation et Médecine Hyperbare, Centre Hospitalier Universitaire d’Angers, Vent’Lab, Univ Angers, Angers, France; ^3^Laboratoire Med2Lab, Air Liquid Medical Systems (ALMS), Antony, France; ^4^Keenan Research Centre, Li Ka Shing Knowledge Institute, St. Michael’s Hospital, Toronto, Canada; ^5^Interdepartmental Division of Critical Care Medicine, University of Toronto, Toronto, Canada

**Correspondence:** François Morin - francois.morin@chu-angers.fr

*Annals of Intensive Care* 2021, **11(Suppl 1):**FC-055

**Rationale:** Intensive care units (ICU) have sometimes been overwhelmed by the surge of COVID-19 patients with acute respiratory distress syndrome (ARDS). Extending ICU capacity can be limited by the lack of air and oxygen (O2) pressure sources available. Transport ventilators, usually used by Emergency Medical Services (EMS), requiring a single O2 source, can be used as an alternative of ICU ventilators in such places. The aim of this study is to compare the performances of four transport ventilators and one ICU ventilator in simulated severe respiratory conditions.

**Patients and methods/materials and methods:** Two pneumatic transport ventilators (Oxylog 3000, Draeger; Osiris 3, Air Liquide Medical Systems (ALMS)), two turbine transport ventilators (Elisee 350, ResMed; Monnal T60, ALMS) and one ICU ventilator (Engström Carestation, GE Healthcare) were evaluated on a Michigan test lung. We tested each ventilator with different tidal set volumes (Vtset = 350, 450, 550 mL), two levels of compliance (20 or 50 mL/cmH2O) and a resistance of 15 cmH2O/L/s based on values described in COVID-19 ARDS. Volume error (%Vtset) with P0.1 of 4 cmH2O and trigger delay during assist-control ventilation simulating spontaneous breathing activity with P0.1 of 4 cmH2O and 8 cmH2O were measured.

**Results:** Grouping all conditions, the %Vtset was 2.9 ± 2.2%; 3.6 ± 3.9%; 2.5 ± 2.1%; 5.4 ± 2.7% and 8.8 ± 4.8% for Engström Carestation; Osiris 3; Oxylog 3000; Monnal T60 and Elisee 350, respectively. Grouping all conditions (P0.1 of 4 cmH2O and 8 cmH2O), trigger delay was 50 ± 11 ms, 71 ± 8 ms, 132 ± 22 ms, 60 ± 12 and 67 ± 6 ms for Engström Carestation, Osiris 3, Oxylog 3000, Monnal T60 and Elisee 350, respectively.

**Discussion:** Transport ventilator performances are close to that of the tested ICU ventilator. In all tested conditions, %Vtset is < 0.5 mL/kg predicted body weight (PBW), except for Elisee 350 and Osiris 3. Trigger delay is acceptable (< 100 ms) except for Oxylog 3000.

**Conclusion:** In overcrowding ICU context caused by COVID-19 pandemia, transport ventilators may be used to accurately control delivered volumes in locations, where only oxygen pressure supply is available. Performances regarding triggering function are acceptable for three out of the four transport ventilators tested.

**Compliance with ethics regulations:** N/A.

### FC-056 B-lines on chest ultrasound predicts elevated left ventricular diastolic pressures

#### Walid Sellami, Ines Ben Mrad, Iheb Labbene, Mustapha Ferjani

##### Department of critical care medicine and anesthesiology, Military Hospital of Tunis, Tunisia, Tunis, Tunisie

**Correspondence:** Walid Sellami - drsellamiwalid@yahoo.fr

*Annals of Intensive Care* 2021, **11(Suppl 1):**FC-056

**Rationale:** The use of ultrasound in the ICU is currently developing as a result of its employment in many indications. The aim of this study was to investigate the relationship between the ultrasonic B-profiles and spectral tissue Doppler echocardiography (E/Ea ratio), a non-invasive surrogate for left ventricular diastolic pressures, in patients presenting with suspicion of acute pulmonary edema

**Patients and methods/materials and methods:** This is a prospective observational study of patients presenting with acute pulmonary edema and B-profile detected by echocardiography with a 5-MHz curvilinear probe. The Filling pressure of the left ventricle considered high when E/Ea is equal or > 15 or when value between 9 and 14 with ultrasound chest B pattern. The filling pressure is considered normal if E/Ea is equal or below 8 or the value between 9 and 14 with A-line pattern.

**Results:** Seventy-five patients were included (45% male, with a mean age 56.3). The mean E/Ea level in the patients with B-profile was (22.1), compared with the mean level in the patients with an A-profile of (8.2) (*p* = 0.002). Based on the value of E/Ea, the sensitivity and specificity (including the 95% confidence interval) were determined and are shown in Table 1. The systolic function in the subjects with a B-profile was below 52% in 70.5% of the subjects. All the subjects with B-profile and systolic function > 50% had elevated NT-proBNP and E/Ea > 15

**Conclusion:** Detecting the B-profile in lung ultrasound is highly sensitive and specific for elevated left ventricular diastolic pressures in patients with acute pulmonary oedema.

**Compliance with ethics regulations:** Yes in clinical research.
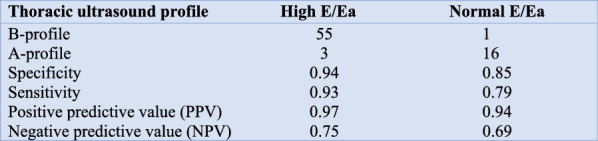


Table 1 Chest ultrasound profiles-based spectral tissue Doppler echocardiography E/Ea

### FC-057 Sampling of heat and moisture exchange filters for SARS-Cov-2 detection by real-time reverse-transcriptase polymerase chain reaction (rRT-PCR) in mechanically ventilated COVID-19 patients

#### Toufik Kamel, Grégoire Muller, Lekbir Baala, Mai-Anh Nay, Fabien Lesne, Armelle Mathonnet, Clémence Guillaume, Marie Skarzynski, Anne Bretagnol, Maxime Desgrouas, Isabelle Runge, Sophie Jacquier, Jérôme Guinard, Thierry Boulain

##### ^1^CHR Orléans, Orléans, France

**Correspondence:** Thierry Boulain - thierry.boulain@chr-orleans.fr

*Annals of Intensive Care* 2021, **11(Suppl 1):**FC-057

**Rationale:** Frequent respiratory samples, usually tracheal aspirates, are recommended in COVID-19 patients treated by mechanical ventilation (MV) to monitor the evolution of the respiratory SARS-Cov-2 load by rRT-PCR and to detect at which time the patient could be de-isolated. However, open tracheal suction is an aerosol-generating procedure that exposes care providers to contamination. The use of heat and moisture exchange filters (HMEF) is recommended for all patients under MV unless the small dead-space they add is seen detrimental. We investigated whether sampling HMEFs for SARS-Cov-2 detection by rRT-PCR could replace tracheal aspirates.

**Patients and methods/materials and methods:** We included patients with laboratory-confirmed COVID-19, treated by MV and who had a HMEF inserted in their ventilator circuit. HMEFs were changed at 72-h intervals according to the manufacturer’s instructions. At each HMEF change, a tracheal aspirate and the used HMEF were sent to the hospital laboratory for SARS-Cov-2 rRT-PCR. The patient’s side of the HMEF was rubbed with a swab according to a standardized procedure; the swab was placed in viral transportation liquid awaiting analysis. With the equipment used (TaqPathTM kit and QuantStudioTM thermocycler [Thermo Fisher]) that targets 3 genes (ORF1ab, S and N), a Cycle threshold (Ct) ≤ 28 or < 32 indicates a significant or a moderate-to-very low viral excretion (1). The Ct values obtained with the HMEF and the tracheal sample were compared by the Bland- Altmann method (for which undetermined Cts were assigned the value of 40). The HMEF/Tracheal ratio of gene expression was calculated by the 2^−ΔΔCt^ method (2). Sensitivity, specificity and area under the ROC curve (AUC) of the HMEF sample Ct value to detect a tracheal sample Ct < 32 were calculated.

**Results:** 129 HMEF-tracheal aspirates pairs of rRT-PCR were obtained in 26 patients. The agreement interval between tracheal and HMEF Cts for the N-gene extended from − 16 to 8. The mean (SD) tracheal/HMEF ratio of gene expression was 1.9 (2.1) log. The AUC of the HMEF N-gene Ct for detecting a tracheal Ct < 32 was 0.86 (95%CI: 0.80–0.92). A HMEF N-gene Ct < 32 had a sensitivity of 50% (95%CI: 38–62) and a specificity of 98% (95%CI: 90–100) for detecting a tracheal Ct < 32.

**Conclusion:** Ct values obtained from HMEF and tracheal aspirates are not interchangeable as tracheal aspirates contain much more viral material. However, due to its high specificity, a HMEF rRT-PCR-positive result obviates the need for a tracheal test to affirm that a patient is not ready to be de-isolated.

**References**

1. Avis du 25 septembre 2020 de la Société Française de Microbiologie (SFM) relatif à l’interprétation de la valeur de Ct (estimation de la charge virale) obtenue en cas de RT-PCR SARS-CoV-2 positive sur les prélèvements cliniques réalisés à des fins diagnos
2. Livak KJ, Schmittgen TD. Analysis of relative gene expression data using real-time quantitative PCR and the 2???Ct method. Methods. 2001; 25: 402–8.

**Compliance with ethics regulations:** Yes in clinical research.
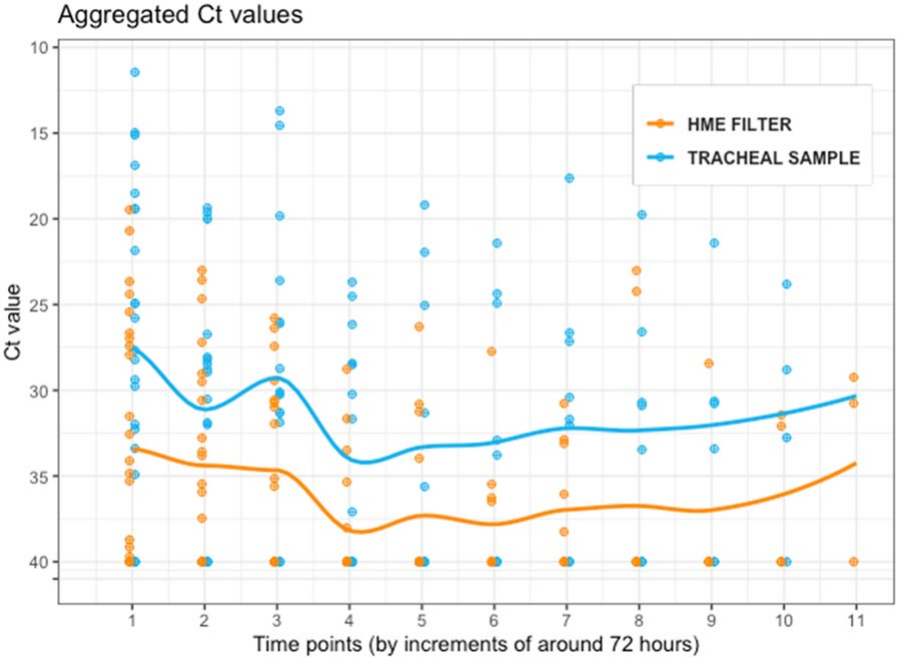


Ct values for the N-gene detection on HME filters and in tracheal samples. The lines represent a smoothed moving average of Ct value in each group.

### FC-058 Does adding a filter to minimize aerosol generation in COVID-19 context impact CPAP performances? A bench study

#### François Morin^1^, Arnaud Lesimple^3^, Nathan Prouvez^3^, Bilal Badat^3^, François Beloncle^2^, Alexandre Broc^3^, Guillaume Carteaux^4^, François Templier^1^, Laurent Brochard^5,6^, Alain Mercat^2^, Dominique Savary^1^, Jean-Christophe Richard^2,3^

##### ^1^Département de Médecine d’Urgence, Centre Hospitalier Universitaire d’Angers, Vent’ Lab, Univ Angers, Angers, France; ^2^Département de Médecine Intensive-Réanimation et Médecine Hyperbare, Centre Hospitalier Universitaire d’Angers, Vent’ Lab, Univ Angers, Angers, France; ^3^Laboratoire Med2lab, Air Liquid Medical Systems (ALMS), Antony, France; ^4^Assistance Publique-Hôpitaux de Paris, CHU Henri Mondor, Service de Médecine Intensive Réanimation, Créteil, France; ^5^Keenan Research Centre, Li Ka Shing Knowledge Institute, St. Michael’s Hospital, Toronto, Canada; ^6^Interdepartmental Division of Critical Care Medicine, University of Toronto, Toronto, Canada

**Correspondence:** François Morin - francois.morin@chu-angers.fr

*Annals of Intensive Care* 2021, **11(Suppl 1):**FC-058

**Rationale:** Patients presenting with acute hypoxemic respiratory failure could need a continuous positive airway pressure (CPAP) ventilation device. Boussignac CPAP (B-CPAP) (Vygon, Ecouen, France) is a cheap, easy-to-use, non-electrical device that works with no ventilator. In COVID-19 context, a bacteriological filter has to be inserted between the oro-nasal mask and the CPAP valve to avoid SARS-CoV-2 aerosolization. We studied the effect of adding a filter on B-CPAP performances.

**Patients and methods/materials and methods:** This bench study has compared, with or without two electrostatic filters (Clear-Guard filter [CG-F] or DAR-Filter [DAR-F]): (1) static measurements of airway pressure effectively delivered depending of the oxygen flow. (2) In dynamic conditions, the work of breathing (WOBimposed) induced by the filter and the consequent tidal volume (VT) reduction at low (− 5 cmH2O) and high (− 10 cmH2O) effort were assessed on a lung simulator. Two levels of CPAP (6 and 10 cmH2O) were tested at a resistance of 5 cmH2O/L/s and 15 cmH2O/L/s and a compliance set at 50 mL/cmH2O. The theoretical increase in patient WOB (WOBpatient) required to maintain the same VT after the addition of the filter was also assessed. We finally evaluated the risk of aerosolization by observing smoke exhalation dispersion.

**Results:** The static airway CPAP pressure effectively delivered was not affected by adding a filter. Dynamic measurements showed that adding a filter variably increased the WOBimposed by the CPAP from 61% to 78% (mostly with a higher filter resistance [DAR-F]) and decreased VT from 236 ± 104 ml to 203 ± 85 ml for a given effort (mostly with a lower filter resistance [CG-F]). The estimated increase of the simulated patient’s effort to sustain initial VT without filter varied from 12 to 31% of its total effort, especially with a higher filter resistance [DAR-F] (Fig.). Smoke exhalation was visible through the conventional B-CPAP, but not through the filter-B-CPAP.

**Conclusion:** Adding a filter to limit contamination doesn’t affect B-CPAP performances on static CPAP level delivered while ensuring low viral aerosolization. It slightly increases the WOBimposed and reduces the VT, requiring increasing the initial WOBpatient, that must be taken into account in a clinical situation.

**Compliance with ethics regulations:** N/A.
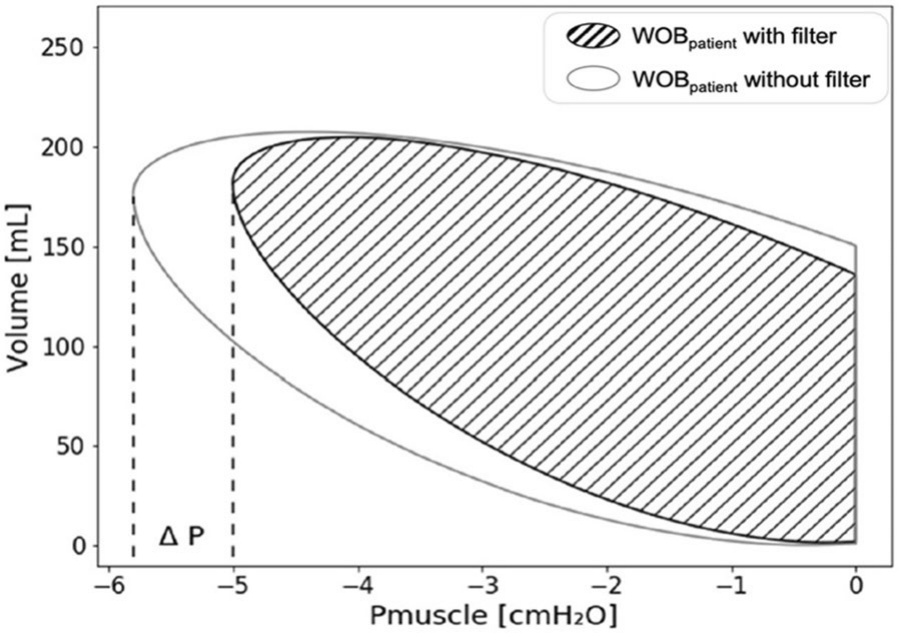


Fig. 1 Dynamic pressure (Pmuscle) volume loops recorded with and without an additional Clear-Guard™ filter (Intersurgical^®^, Fontenay Sous Bois, France)

### FC-059 The use of filters in intensive care units in COVID-19 period, in context of supply tensions

#### Justine Lemtiri, Céline Cafede, Emilie Herbez, Constance Merlin, Jérôme Aubert, Julien Boyer^1^, Nabil Elbeki^1^, Fabien Lambiotte^1^, Perrine Drancourt^1^

##### CH Valenciennes, Valenciennes, France

**Correspondence:** Justine Lemtiri - lemtiri-j@ch-valenciennes.fr

*Annals of Intensive Care* 2021, **11(Suppl 1):**FC-059

**Rationale:** In March 2020, expert agreements preconised the use of the hydrophobic filter in ICU, in order to protect the ventilation devices (optiflow, respirator) in the COVID-19 patient in addition to those usually used in ICU: the electrostatic filter and the hydrophilic-patient filter. Their use was explained in internal procedure. However, in view of the numerous stock shortages, the sources of supply were multiple, leading to confusion between the different filters (color/form/filter link made in the absence of standardization). The purpose of this work is to make an inventory of the good use of filters and to set up improvement actions;

**Patients and methods/materials and methods:** In September 2020, a multidisciplinary group was formed, composed of intensivists, health managers, the critical care pharmacist (CP) and the pharmacist specialized in medical devices (PMD). An audit grid has been drafted to enable the CP and PMD to perform an EPP (Evaluation des Pratiques Professionnelles) in ICU. Areas for improvement were established and implemented (good use sheet, training).

**Results:** Four audits were conducted between November 2020 and January 2021. At the beginning of November, 67% of the filters usage were in conformity. Work was then carried out jointly by the pharmacy and the ICU (verification of the storage areas, discussions with the nurses/health managers), and a poster was produced and put in place in the storage areas and in units (see appendix). Despite these measures, compliance remained stable at 67% and dropped 2 weeks later to 29%. Following these results, during 1 month, 8 short training sessions for caregivers (70% of the personnel trained [65 caregivers]) were conducted, resulting in a compliance rate of 73% during the 4th audit (the main “non-compliance” being the presence of a hydrophobic filter machine in the non-COVID patients, i.e. an overprotection of the machine).

**Conclusion:** The COVID-19 period is marked in ICU by an increased activity, medical and paramedical staff coming in for support and practices evolving every day. In this context, the link between ICU and pharmacy, facilitated by the presence of a CP, which was put to the test during this period, was strengthened and made it possible to carry out projects with good use. The main difficulty was communication with all filter users the time needed for training was unfortunately not found in the middle of the COVID-19 wave. A monthly audit of filter use will be carried out if there are COVID-19 patients in ICU.

**Compliance with ethics regulations:** N/A.

### FC-060 Diaphragm function in patients with sepsis and septic shock: a longitudinal ultrasound study

#### Aymeric Le Neindre^1^, Johan Wormser^2^, Marta Luperto^2^, Cédric Bruel^2^, Benoit Misset^4^, Belaid Bouhemad^3^, Francois Philippart^2^

##### ^1^Hôpital Forcilles, Férolles-Attilly, France; ^2^Groupe Hospitalier Paris Saint-Joseph, Paris, France; ^3^CHU de Dijon, Dijon, France; ^4^Centre Hopsitalier Universitaire de Liège, Liège, Belgique

**Correspondence:** Aymeric Le Neindre - aymeric.leneindre@gmail.com

*Annals of Intensive Care* 2021, **11(Suppl 1):**FC-060

**Rationale:** Sepsis is very common in the Intensive Care Unit (ICU) and is a major risk factor of muscle dysfunction. Limited previous literature studied determinants of diaphragm dysfunction in septic ICU patients. The goal of the study was to evidence the proper effect of sepsis on the diaphragm in ICU patients.

**Patients and methods/materials and methods:** This prospective and observational study was conducted between June 2015 and July 2019. Ultrasound measures of diaphragm thickness were daily performed in septic patients. The primary outcome was the prevalence of diaphragm dysfunction at baseline and during ICU stay. Secondary outcomes were the diaphragm thickness and the diaphragm daily atrophy rate. Potential associated factors were prospectively recorded.

**Results:** Fifty patients were enrolled in the study. The prevalence of diaphragm dysfunction was 58% and was significantly higher in ventilated patients than in non-ventilated patients (71 vs. 37%, *p* = .04). The incidence of diaphragm dysfunction during ICU stay was 22%. No diaphragm atrophy during ICU stay was found. Diaphragm dysfunction was associated with the alteration of consciousness, intra-abdominal sepsis, hypnotics and opioids, and mechanical ventilation. Administration of hypnotics, opioids, and steroids was associated with a lower diaphragm thickening fraction. Diaphragm dysfunction had no impact on patients’ outcomes. Forty-five percent of patients with diaphragm dysfunction recovered from during ICU stay.

**Conclusion:** Our data show a high prevalence of diaphragm dysfunction in septic patients occurring at the onset of sepsis. Administration of hypnotics, opioids, and steroids was associated with alteration of diaphragm function as well as intra-abdominal sepsis.

**Compliance with ethics regulations:** Yes in clinical research.

### FC-061 Prognosis and risk factors of mechanical ventilation of ICU patients with Guillain–Barré syndrome

#### Rezk Ghorbel, Olfa Turki, Houda Mayoufi, Kamilia Chtara, Rania Ammar, Chokri Ben Hamida, Mabrouk Bahloul, Mounir Bouaziz

##### centre Hospitalo-universitaire Habib Bourguiba Sfax, Sfax, Tunisie

**Correspondence:** Rezk Ghorbel - rezkghorbel3@gmail.com

*Annals of Intensive Care* 2021, **11(Suppl 1):**FC-061

**Rationale:** Patients suffering from Guillain–Barré syndrome (GBS) may develop an acute respiratory failure, which need frequently ventilatory support. We assessed clinical and para-clinical predictors of mechanical ventilation (MV) in patients hospitalized in the ICU for a GBS.

**Patients and methods/materials and methods:** We retrospectively collected the demographic, clinical, laboratory, and electrophysiological data of patients hospitalized for GBS decompensation in the intensive care unit of our university hospital between 2010 and 2020. Patients were categorized into two groups based on the requirement for MV. Factors associated with the need for MV were determined using both univariate and multiple analyses.

**Results:** Forty-three patients with severe GBS were studied. The mean age of our population was at 41.4 ± 15 years with a sex ratio at 1.4. Twenty-two patients (51.1%) required MV. The history of high blood pressure (*p* = 0.04), the severity of the risk score at admission (SOFA score and SAPS II score with *p* < 0.001) and lower limbs or bulbar weakness (*p* = 0.037), were associated with the use of MV.

**Conclusion:** Understanding the risk factors of severe GBS and requiring mechanical ventilation may provide better treatment strategies and improve the outcomes.

**Compliance with ethics regulations:** Yes in clinical research.

### FC-062 Hypertension is an independent risk factor associated with mortality in ARDS treated by veno-venous extracorporeal membrane oxygenation: a multicentric retrospective observational study

#### Elise Godeau^1^, Thomas Clavier^2^, Emmanuel Besnier^2^, Christophe Girault^2^, Fabienne Tamion^2^, Gaetan Beduneau^2^, Nicolas Cousin^3^, Cédric Daubin^4^, Elie Zogheib^5^, Steven Grange^2^

##### ^1^CHi Elbeuf, Saint Aubin Les Elbeuf, France; ^2^CHU ROUEN, Rouen, France; ^3^CHU LILLE, Lille, France; ^4^CHU CAEN, Caen, France; ^5^CHU AMIENS, Amiens, France

**Correspondence:** Elise Godeau - elisegodeau@gmail.com

*Annals of Intensive Care* 2021, **11(Suppl 1):**FC-062

**Rationale:** Acute respiratory distress syndrome (ARDS) is a severe condition associated with a high mortality rate. Veno-venous extracorporeal membrane oxygenation (VV-ECMO) is used when hypoxemia is refractory to conventional therapies, but its large-scale indication remains currently controversial. Mortality risk factors identification could help clinicians to select patients who could most benefit from VV-ECMO.

**Patients and methods/materials and methods:** Patients with severe ARDS treated by VV-ECMO between January 2015 and December 2019. The main criterion was the association of major chronic comorbidites with ICU mortality.

**Results:** 192 patients with severe ARDS treated by VV-ECMO were included. The mortality rate in ICU was 50.5%. The median RESP score was 1 [−1–3], significantly lower in deceased patients. We identified hypertension, low BMI (body mass index), and high SAPS II as independent risk factors associated with mortality. Progressive neoplastic pathology, male sex, age, cardiopathy, diabetes, chronic respiratory disease were not identified as independent risk factors for ICU mortality in multivariate analysis. The mortality rates at 90 days and 6 months were 53% and 54.6%, respectively

**Conclusion:** Hypertension appears to be an independent risk factor for ICU mortality in patients with very severe ARDS treated with VV-ECMO.

**Compliance with ethics regulations:** N/A.
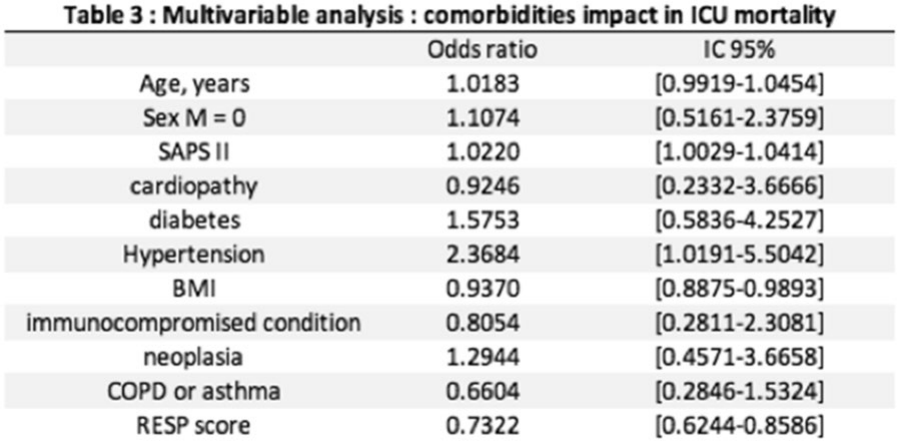


Table 1 Values are odds ratio Abbreviations: BMI = body mass index; COPD = chronic obstructive pulmonary disease; ICU: intensive care unit; RESP Score: Respiratory Extracorporeal membrane oxygenation Survival Prediction; Score SAPS II = Simplified Acute Physiology Score

### FC-063 Incidence of pulmonary embolism in hypoxic patients in intensive care

#### Najeh Baccouch, Houda Myoufi, Sana Kharrat, Rania Ammar, Mabrouk Bahloul, Mounir Bouaziz

##### Faculty of medecine of Sfax university of Sfax, Sfax, Tunisie

**Correspondence:** Najeh Baccouch - baccouch.najeh@gmail.com

*Annals of Intensive Care* 2021, **11(Suppl 1):**FC-063

**Rationale:** Hypoxemia affected more than half of ICU patients The pulmonary embolism (PE) remains a serious condition that requires intensive care unit (ICU) admission

**Patients and methods/materials and methods:** We aimed to determine the incidence of pulmonary embolism in hypoxic patients in intensive care. This is a prospective study, conducted in the department of emergencies and ICU of Habib Bourguiba Hospital during a 6-month period carried out from 1 January 2020 to 30 June 2020. All patients who presented with hypoxia are included in this study. Hypoxemia was defined as a PaO2/FiO2 ratio ≤ 300 mmHg

**Results:** During this period three hundred and fifty patients were admitted to the ICU. One hundred of them developed hypoxia. The mean age was 54.74 ± 17.54 years with extremes ranging from one year at 88. Sex ratio was 2.44. The SAPS II score was 45.6 [14; 76] points and the SOFA score was 6.8 ± 2.7 points [4; 14]. The reason of ICU admission were acute respiratory failure (34%), shock (20%) polytrauma (20%), post-operative resuscitation (21%) and poisoning (5%). The mean time to onset of hypoxia was 0.9 day with extremes ranging from 0 to 10 days. Sixty patients were ventilated on admission. Non-invasive ventilation was required for 18% of patients and invasive mechanical ventilation was necessary in 65% of cases. The median duration of mechanical ventilation was 12 days [10–23]. Pulmonary embolism was the cause of hypoxia in 10% of cases. The other etiologies of hypoxia were: pneumonia, acute pulmonary edema, pneumothorax, atelectasis, Pulmonary embolism was more frequent in polytrauma patient (75%). The death rate was 53%. Pulmonary embolism was not a risk factor for death in the multivariate analysis

**Conclusion:** Pulmonary embolism is a frequent complication in ICU. But it was not the main cause of hypoxemia.

**Compliance with ethics regulations:** Yes in clinical research.

### FC-064 Clinical characteristics and outcomes of patients with severe viral community-acquired pneumonia

#### Fatma Essafi^1,2^, Meriam Ksouri^1,2^, Boudour Ben Dhia^1,2^, Imen Talik^1,2^, Moez Kaddour^1,2^, Takoua Merhabene^1,2^

##### ^1^faculté de médecine de Tunis, Tunis, Tunisie; ^2^Réanimation médicale hôpital régional Zaghouan, Zaghouan, Tunisie

**Correspondence:** Meriam Ksouri - meriamksouri18@gmail.com

*Annals of Intensive Care* 2021, **11(Suppl 1):**FC-064

**Rationale:** Viral community-acquired pneumonia (VCAP) requiring intensive care units (ICU) admission is still poorly documented and characterized. Here, we analyzed epidemiology, clinical features and management of all patients admitted in ICU for severe VCAP.

**Patients and methods/materials and methods:** It was a retrospective descriptive study conducted over 3 years from September 2018 to December 2020. Patients admitted to Zaghouan’s ICU for confirmed VCAP were enrolled. We collected baseline characteristics, management and outcomes.

**Results:** During the study period, 120 patients were enrolled (incidence = 183/1000 admissions). Mean age was 61.14 ± 14.4 years [13–92]. Sex ratio was 1.5. Mean APACHE II and SAPS II scores were, respectively, 10 ± 5 and 27.9 ± 10.6. Chronic heart disease, obesity, diabetes mellitus, and chronic obstructive pulmonary disease were the most common co morbidities (52.5%; 32.5%. 26.7% and 25.8%, respectively). Acute respiratory failure was the main cause of ICU admission (100%). Initial symptoms are essentially fever (81.8%), nonproductive cough (85.8%), dyspnea (95%) and gastrointestinal symptoms (23.3%). Acute respiratory distress syndrome was present in 71% of cases. It was mild in 8 patients (9.4%), moderate in 33 patients (39%) and severe in 44 patients (52%). Biological associated disorders were frequent: lymphopenia (73.3%), hyponatremia (29.2%), hepatic cytolysis (16.7%) and rhabdomyolysis (10%). Virus was detected by PCR on nasopharyngeal swab in most patients (99.1%). Isolated pathogens were, respectively, SARS COV-2 (*n* = 88), haemophilus influenza (*n* = 20), measles (*n* = 1) and rubella virus (*n* = 1). Mechanical ventilation was indicated in 70.8%, it was invasive in 61%. Mean duration of mechanical ventilation was 6.6 ± 4 days. At admission, septic shock was present in 4 patients and acute renal failure in 30 patients (25%). Antiviral treatment was prescribed in 20 patients. Prone positioning was prescribed in 54.5%, it was essentially applied in awake COVID-19 patients. Most of patients had received corticosteroids (83.5%) and antibiotics (95.5%). Care-related complications were frequents: ventilator? associated pneumonia in 32 patients, pulmonary embolism in 2 patients, acute renal failure in 32 patients, pneumomediastinum in one patient. Median ICU length of stay was 9.2 ± 5.5 days [2–26]. Hospital mortality was 47. 5%. The outcome was favorable in 58 patients (48%). Multivariate analysis showed that age ≥ 65 years, chronic heart disease and mechanical ventilation were independent factors for ICU mortality.

**Conclusion:** VCAP admitted in ICU are mostly presented as acute respiratory distress requiring ventilation. It was associated with a high ICU mortality. Age ≥ 65 year, chronic heart disease and mechanical ventilation were independent risk factors for ICU mortality.

**Compliance with ethics regulations:** N/A.

### FC-065 Ecology of nosocomial infections in the intensive care unit setting of surgical emergencies

#### Mehdi Soussane El

##### CHU Casablanca, Casablanca, Maroc

**Correspondence:** El Mehdi SOUSSANE (Soussaneelmehdi@gmail.com)

*Annals of Intensive Care* 2021, **11(Suppl 1):**FC-065

**Rationale:** Analyze the epidemiological and bacteriological profile of nosocomial infections in the intensive care unit of surgical emergencies.

**Patients and methods/materials and methods:** This work consists of carrying out a retrospective study of 332 cases of nosocomial infections in the intensive care unit of surgical emergencies of the Ibn Rochd CHU in Casablanca carried out between January 2007 and November 2011. Have been included: the data were entered and analyzed with the EPI INFOS version 6 software. The statistical analysis is performed using the Student’s test and the CHI 2 test. A *P* value < 0.05 was considered significant.

**Results:** Of the 332 cases collected, there is a clear male predominance over the entire study period with a sex ratio of 4.9. The average age is 43 ± 17 years. To better assess the severity of infected patients, we mainly used the following scores: the GCS was on average 10 ± 3; the IGS II was on average 28 ± 9. In terms of history, 28.2% of infected patients have a pathological history of which diabetes is the most common. Among 1932 patients hospitalized during the study period, 1725 were hospitalized for more than 48 h, of which 332 were infected, for an incidence of 19.25%. The overall incidence density was 36.6 per 1000 hospital days and that per infectious site shows a high rate of pneumonia. In terms of infectious sites, pulmonary infection ranked first throughout the study period. Of our infected patients, 34.3% received prior antibiotic therapy.

**Conclusion:** Nosocomial infection is associated with heavy mortality and morbidity. Not to mention the high cost which represents an economic burden adding to the budgetary constraints of hospitals. In order to limit this damage, a preventive strategy must be organized which will be based on general measures, mainly washing hands by friction with a hydroalcoholic product, the proper use of gloves, isolation of patients at risk, and the geographical organization of services.

**Compliance with ethics regulations:** Yes in clinical research.

### FC-066 Is Cirrhosis associated with increased risk for abundant microaspiration in critically ill mechanically ventilated patients?

#### Clémentine Levy^1^, Emmanuelle Jaillette^1^, Jean Reignier^2^, Malika Balduyck^1^, Saadalla Nseir^1^

##### ^1^CHRU de Lille, Lille, France; ^2^CHRU de Nantes, Nantes, France

**Correspondence:** Clémentine Levy - levy_clem@msn.com

*Annals of Intensive Care* 2021, **11(Suppl 1):**FC-066

**Rationale:** Intrabdominal hypertension and cirrhosis were identified as risk factors for ventilator-associated pneumonia (VAP). However, no study to date has evaluated the impact of cirrhosis on microaspiration. The aim of our study was to determine the relationship between abundant microaspiration of gastric content and cirrhosis in intubated critically ill patients.

**Patients and methods/materials and methods:** We performed a case–control study using data from three randomized controlled trials on abundant microaspiration in patients receiving invasive mechanical ventilation for more than 48 h. Each cirrhotic patient was matched with 2 controls, based on sex, age, SAPS-II and ICU admission category. Cirrhosis was defined by histology or the association of clinical, laboratory and radiological evidence. Abundant microaspiration was defined by the presence of pepsin and alpha amylase in tracheal aspirates at significant levels. All tracheal aspirates were collected for the first 48 h of study period. Abundant gastric content microaspiration was the primary outcome. Secondary outcomes included abundant microaspiration of oropharyngeal secretions, VAP incidence, duration of mechanical ventilation, ICU length of stay and mortality.

**Results:** Of all 599 patients, 38 (6.3%) had cirrhosis, and were successfully matched with 71 controls. Shock at admission was significatively more frequent in cases than in controls (84.2% vs 59.2% *p* = 0.014 (0.07–0.63)). Abundant microaspiration of gastric contents did not differ between the two groups (55.6 vs 59.4% (95% CI (0.85–1.35), *p* = 0.59)). There was no significant difference in duration of mechanical ventilation, ICU length of stay, or ICU mortality (HR = 1.44; 95% CI 0.72–3.02, *p* = 0.28). Cirrhotic patients had significantly higher risk to develop VAP (HR = 2.0; (95% CI 1.02–3.93), *p* = 0.04) (Fig. 1).

**Conclusion:** Our results suggest that cirrhosis is not associated with abundant gastric-content microaspiration in mechanically ventilated patient. VAP could be favored by other mechanisms, such as bacterial translocation, and immunological alterations in cirrhotic patients.

**Compliance with ethics regulations:** Yes in clinical research.
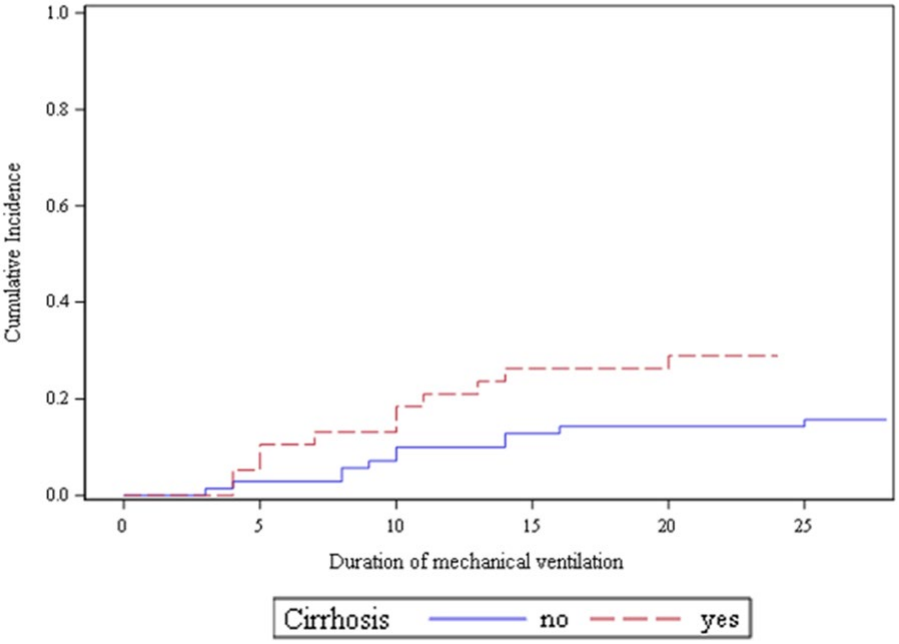


Fig. 1 Comparison of ventilator-associated pneumonia incidence between cases and controls

### FC-067 Clinical characteristics, treatment and outcomes of critically ill patients with COVID 19

#### Khaoula Ben Ismail, Amenne Alouini, Boudour Ben Dhia, Fatma Essafi, Moez Gaddour, Takoua Merhabene

##### Hopital regional de Zaghouan, Zaghouan, Tunisie

**Correspondence:** Khaoula Ben Ismail - khaoula87@hotmail.fr

*Annals of Intensive Care* 2021, **11(Suppl 1):**FC-067

**Rationale:** The new coronavirus, severe acute respiratory syndrome coronavirus 2 (SARS-CoV-2) has globally spread since December 2019. To this date 220.000 cases were reported in Tunisia. Purpose: To describe epidemiologic, clinical and paraclinical features management and outcomes of critically ill COVID 19 patients.

**Patients and methods/materials and methods:** It was a retrospective, descriptive and cross-sectional study, including all critically ill COVID-19 confirmed patients hospitalized in the medical intensive care unit of Zaghouan Hospital (Tunisia) from March 2020 to 31st January 2020. We collected demographic, clinical, paraclinical, evolving therapeutic and outcomes.

**Results:** 126 patients were enrolled. Mean age was 62 ± 12 years [34–92] with a sex ratio 1.5. 47% of patients were over 65 years. Seventy-nine patients (65.3%)were obese(IMC ≥ 30)and 30.6% were smokers. Mean SAPSII, Charlson and APACHE II scores were 25.9 ± 8 [6–50]; 2.8 ± 1 [0–7]; 7.7 ± 3 [1–27], respectively. Comorbidities were present in 66.9% of cases; it was mainly cadio-vascular (60.3%) and diabetes (35.5%). Dyspnea was reported in 84.3% cases, fever in 84.3%, cough in 81.4% and asthenia in 73.6%. Digestive signs and headaches were, respectively, reported in 21.5% and 34.7% of cases. Less than 20% of patients reported oto-rhino-laryngology symptoms. At admission, 4 patients had a septic shock (3.3%) and 24 patients (19%) had acute renal failure. Acute respiratory distress syndrome was present in 78.4% of cases, median P/F = 155 mmHg [47–583]. Eight patients (6.6%) had rhabdomyolysis and 28 (23.1%) had hypophosphatemia. On the chest tomography, 112 patients (92.6%) had ground-glass bilateral opacities, 57 (58%) had pulmonary condensation and 70 (47%) had crazy paving aspect. All patients received anticoagulation therapeutic-dose, vitamin therapy, β-lactamines and corticosteroids. Mechanical ventilation was necessary in 92.5% of patients, it was invasive in 44.6% of cases. Awake prone position was applied in 65.5% of cases. ICU stay was characterized by the occurrence of acute renal failure in 30.6% and nosocomial infection in 39.7% with a predominance of ventilator-associated pneumonia (87.5%). Means duration of invasive mechanical ventilation was 7.5 days [1–19] and length of ICU stay was days 9.7 [2–30]. Overall mortality rate was 43%. Multivariate analysis showed that independent predictor factors of mortality were age > 65 year, nosocomial infection, acute renal failure and invasive mechanical ventilation.

**Conclusion:** Extreme age, acute renal failure, use of invasive mechanical ventilation and nosocomial infection were key determinants of worse prognosis in our study. These findings should be taken into consideration during future healthcare planning in our country.

**Compliance with ethics regulations:** Yes in clinical research.

### FC-068 Comparison of the characteristics, morbidity, and mortality of COVID-19 and seasonal influenza in a Tunisian intensive Care Unit

#### Meriam Ksouri^1,2^, Fatma Essafi^1,2^, Imen Talik^1,2^, Khaoula Ben Ismail^1,2^, Moez Kaddour^1,2^, Takoua Merhabene^1,2^

##### ^1^faculté de médecine de Tunis, Tunis, Tunisie; ^2^Réanimation médicale hôpital régional Zaghouan, Zaghouan, Tunisie

**Correspondence:** Meriam Ksouri - meriamksouri18@gmail.com

*Annals of Intensive Care* 2021, **11(Suppl 1):**FC-068

**Rationale:** Since December 2019, a new disease known as COVID-19, caused by severe acute respiratory syndrome coronavirus 2 (SARS-COV2) emerged. Considering its ability to spread, it was thought to be similar to influenza giving that they are two viral respiratory diseases with the same mode of transmission. The aim was to compare risk factors, clinical features, and outcomes between patients hospitalised for COVID-19 and influenza.

**Patients and methods/materials and methods:** It was a retrospective comparative study conducted in the ICU of Zaghouan’s hospital, Tunisia, between September 2018 and December 2020. 2 groups were individualized: G1 = patients hospitalized for influenza; G2 = patients hospitalized for COVID-19. Clinical data and outcomes were compared between the two groups.

**Results:** During the study period, 118 patients were enrolled, 88 were assigned to G1 and 30 in G2. Median ages were, respectively, 63 ± 13 and 57 ± 17 years. A clear male predominance was noted in the 2 groups. Mean APACHE II score were, respectively, 15 ± 7.6 and 8.5 ± 3.7. Patients in G1 were more frequently obese or overweight, had more often diabetes, heart failure and hypertension, whereas those with influenza had more frequently history of chronic respiratory disease (43.3% vs. 20.5%; *p* = 0.014). Patients hospitalized for influenza have significantly a higher tobacco consumption (50.0% vs 19.3%; *p* = 0.001). At admission, the same most frequent respiratory signs were reported in the two groups. Patients with COVID-19 exhibited higher proportions of gastrointestinal symptoms (28.4% vs 10%; *p* < 0.05). In comparison, patients with COVID-19 had more often lymphopenia (80.7% vs 50%; *p* = 0.001), but less acute renal failure (15.9% vs 53.3%, *p* < 0.05) and rhabdomyolysis (6.8% vs 20%; *p* = 0.039). Patients with COVID-19 had significantly higher incidence of acute respiratory distress syndrome, it was classified more frequently moderate and/or severe (70% vs 36%; *p* = 0.011). Need to invasive mechanical ventilation occurred more likely in G1 (48.86% vs 30%; *p* < 0.05) with longer mean duration (7 ± 4 vs. 4.3 ± 3 days; *p* = 0.007). Prone position was performed in 67% of patients with COVID-19 vs. 3.3% in G2 (*p* < 0.05). Mean length of ICU stay was significantly longer in G1 (10.2 ± 5.6 vs. 6.3 ± 3.75 days, *p* = 0.005). Hospital mortality was higher for patients with COVID-19 (51% vs. 40%) (OR = 1.6, 95% IC 0.67–3.64, *p* = 0.2)

**Conclusion:** COVID 19 and influenza are two serious respiratory diseases which differ considerably. Patients with COVID 19 have more comorbidities, leading to more risk of developing acute respiratory failure and higher mortality.

**Compliance with ethics regulations:** N/A.

### FC-069 Comparison of Covid-19 patient management and outcome in a tertiary care center during the first and second waves of the pandemic

#### Bernard Lambermont^1^, Laurence Seidel^1^, Marie Thys^1^, Jonathan Cavalleri^1^, Pierre Delanaye^1^, Jean-Luc Canivet^1^, Grâce Kisoka^1^, Nathalie Layios^1^, Didier Ledoux^1^, Paul Massion^1^, Philippe Morimont^1^, Gilles Parzibut^1^, Sonia Piret^1^, Sébastien Robinet^1^, Anne-Françoise Rousseau^1^, Patricia Wiesen^1^, Geoffrey Chase^2^, Christelle Meuris^1^, Michel Moutschen^1^, Benoît Misset^1^

##### ^1^University Hospital of Liege, Liege, Belgique; ^2^Department of Mechanical Engineering, Centre for Bio-Engineering, University of Canterbury, Christchurch, Nouvelle-Zelande

**Correspondence:** Bernard Lambermont - b.lambermont@chuliege.be

*Annals of Intensive Care* 2021, **11(Suppl 1):**FC-069

**Rationale:** This retrospective single-center study assesses differences in patient management and outcome during the first (W1) and second (W2) waves of the Covid-19 pandemic.

**Patients and methods/materials and methods:** All adult patients hospitalized at the University Hospital of Liege for Covid-19 from March 15 to May 15, 2020 (W1) and from October 1 to November 30, 2020 (W2) were included. Primary outcome was 30-day mortality. Secondary outcomes were: 30-day ICU mortality; transfer to ICU from the ward; ICU length of stay (LOS); use and duration of mechanical ventilation (MV); use of high-flow nasal oxygen (HFNO); renal replacement therapy (RRT); and vasopressor support.

**Results:** During W1 and W2, 341 and 695 patients were hospitalized for Covid-19. The severity on hospital admission was similar in the two cohorts. During W2, 33 patients (4.8%) were secondarily transferred to other hospitals due to local overcrowding and were excluded from this analysis. Median age of W1 and W2 patients was 68 (57–80) and 71 (60–80) (*p* = 0.15), respectively. 30-day mortality was 74/341 (22%) W1 and 98/662 (15%) W2, respectively (*p* = 0.0074). In the ward, 11/341 (3.2%) W1 and 404/662 (61%) W2 patients received dexamethasone (*p* < 0.001); 6/341 (2%) and 79/662 (12%) patients received HFNO (*p* < 0.0001); 87/341 (26%) and 128/662 (19%) (*p* = 0.02) patients were transferred to ICU. On ICU admission, median SOFA was 6 (3–7) in W1 and 4 (3–6) (*p* = 0.02) in W2 patients. HFNO was given to 16/87 (18%) W1 and 102/128 (80%) W2 ICU patients (*p* < 0.0001). A total of 69/87 (79%) and 56/128 (44%) patients were mechanically ventilated (*p* < 0.001) with durations 17 (10–26) and 10 (5–17) days (*p* = 0.01). Median ICU LOS was 14 (5–27) and 6 (3–11) days (*p* < 0.001); 16/87 (18%) and 8/128 (6%) patients received RRT (*p* = 0.0055); and 64/87 (74%) and 51/128 (40%) needed vasopressor support (*p* < 0.001). 30-day ICU mortality was 25/87 (29%) and 31/128 (24%) during W1 and W2, respectively (*p* = 0.61).

**Conclusion:** In our hospital, the main therapeutic changes between W1 and W2 were use of steroids for hypoxemic patients and HFNO, while fewer patients received MV fort shorter durations and 4.7% of W2 patients were transferred to other hospitals in case of overcrowding. These changes were associated with a decrease in 30-day mortality, ICU admission and organ support, suggesting a better selection of patients requiring ICU, alleviating local overcrowding and likely improving quality of care and long-term recovery for survivors.

**Compliance with ethics regulations:** Yes in clinical research0

### FC-070 Prognosis of COVID-19 patients in the intensive care unit: impact of Emergency Mobile Unit

#### Sarra Akkari, Emna Rachdi, Rim Jameli, Samia Ayed, Fatma Jarraya, Amira Jamoussi, Jalila Ben Khelil^1^

##### Hopital Abderrahman Mami, Ariana, Tunisie

**Correspondence:** Emna Rachdi - e.rachdi@yahoo.fr

*Annals of Intensive Care* 2021, **11(Suppl 1):**FC-070

**Rationale:** COVID-19 is a pandemic affection that continues to spread. Each country has planned a consensus for patient management according to its health system. In Tunisia, prehospital emergency care is working in regulating and transporting COVID-19 patients. Some other patients have chosen to consult the emergency room themselves. Does the way to get to medical care have an impact on the prognosis?

**Patients and methods/materials and methods:** It was a prospective descriptive and comparative study in the intensive care unit of: Abderrahmane Mami’s Hospital during 9 months (from March to November 2020). Patients included were those how were hospitalized for SARS-COV2 infection by Emergency Mobile Unit (Group1(G1)) or via the hospital’s emergency room (Group2(G2)).

**Results:** We included 60 patients: 30 for each group. The average age was 62 years for G1 and 63 years for G2 (*p* = 0.18) with a male predominance for both groups (*p* = 0.4). The most common histories were hypertension (14 cases in G1 and G2, *p* = 1), diabetes (10 cases in G1 and 8 cases in G2, *p* = 0.75) and obesity (14 cases in G1 and 12 cases in G2, *p* = 0.55). Patients in G2 consulted the emergency department later (*p* = 0.02) with a mean time from symptom onset to hospitalization of 9.6 days (vs. 6.5 days for G1). On admission, they were more asthenic (*p* = 0.024), with a mean spo2 of 89% (vs 95% in G1, *p* = 0.008). Patients returned by the Emergency Mobile Unit had less recourse to orotracheal intubation (11 cases vs 21 in G2, *p* = 0.01). Mortality was significantly higher in G2 patients (21 cases vs 10 in G1, *p* = 0.04).

**Conclusion:** COVID-19 patients who were hospitalized in intensive care in coordination with the prehospital emergency care and transported via the intensive care unit, have had a better prognosis compared to patients who were hospitalized via the emergency room.

**Compliance with ethics regulations:** Yes in clinical research.

### FC-071 Comparison between two waves of patients hospitalized into ICU for Covid 19 infection

#### Giulia Moratelli, Kais Regaieg, Takoua Khzouri, Nesrine Fraj, Laure Gazaigne, Dany Goldran-Toledano

##### GHT GRAND-PARIS NORD-EST, Paris, France

**Correspondence:** Giulia Moratelli - giulia.moratelli@gmail.com

*Annals of Intensive Care* 2021, **11(Suppl 1):**FC-071

**Rationale:** We analyzed the epidemiological, clinical and prognostic differences between the two waves of Covid.

**Patients and methods/materials and methods:** This was an observational retrospective study, we collected data of Covid patients admitted into ICU during 2020. We have considered as first wave the period between from March 2020 to July 2020 and the second from August 2020 to December 2020.

**Results:** In the first wave, 75 patients were included in the study. Median age was 58 (28–81); mortality rate was 29% (*n* = 22). Median SAPS was 35.9 (12–60), male sex patients were 55 (73%), median BMI was 30 (16–68), diabetic patients were 21 (28%). The patients presenting AKI were 19 (26%), the chronic kidney diseases were 7 (9.5%), 10 of them (13%) have needed RRT; 50 (66.7%) patients were treated with NIV and 43 (57%) have received mechanical ventilation for a median duration of 11.5 (1–59) days with a mortality of 42% (18), 8 (11%) have needed ECMO. 31 (42%) patients presented a shock. In the univariate analysis the variables associated with mortality were age, SAPS and mechanical ventilation. In the second wave 83 patients were included. Median age was 61 (25–86) and the mortality rate was 24% (*n* = 20). Median SAPS was 33.8 (18–89), shocked patients were 30 (37%) patients. Male sex patients were 55 (66%), median BMI 30 (16–64), diabetic patients were 37 (45%). Patients with chronic kidney disease were 12 (14%) and 23 (28%) patients presented AKI at the admission, 7 (8.4%) of them have needed RRT. NIV was performed in 71 (86%) cases and 34 (41%) patients have needed mechanical ventilation for a median duration of 6.5 (1–38) days with a mortality rate of 47% (*n* = 16); no one needed ECMO. In the univariate analysis the mortality was associated with age, SAPS, BMI, AKI, shock and mechanical ventilation. The multivariate analysis has not demonstrated statistically significative differences of both general mortality in the two waves (*p* = 0.46) and ventilated patient’s mortality (*p* = 1)

**Conclusion:** The difference between the general mortality rate of the two waves is not statistically significant even if it is inferior in the second wave; may be the mortality is influenced by the use of dexamethasone and better knowledge of the disease.

**Compliance with ethics regulations:** Yes in clinical research.
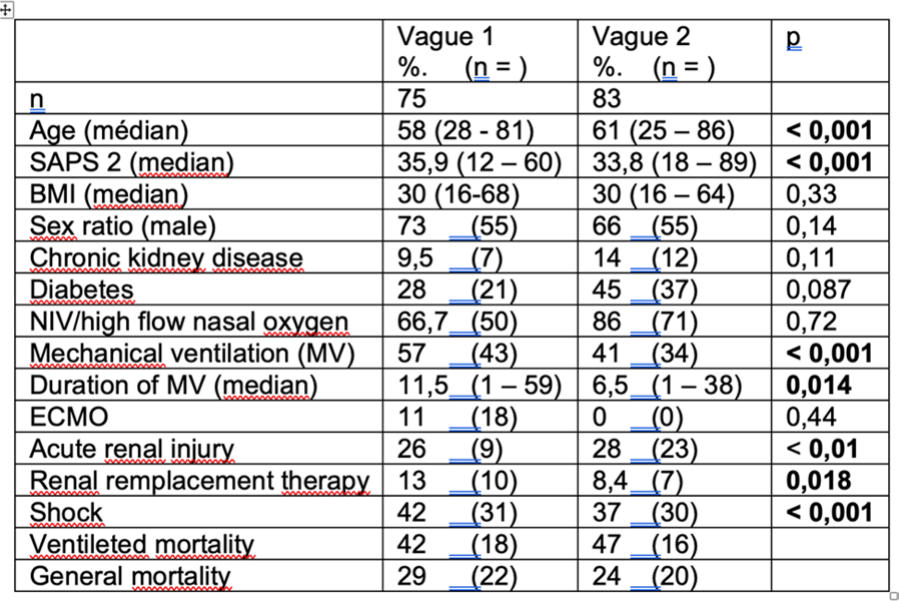


Table 1 Epidemiological characteristics and univariate analysis as a function of mortality

### FC-072 Respiratory status and quality of life 6 months after severe COVID 19: an intensive care unit (ICU) cohort

#### Nicolas Viault^1^, Mouloud Bellahoues^1^, Celine Piedvache^3^, Hervé Clavier^1^, Laurent Tric^1^, Marlene Cherruault^1^, Jean Luc Le Guillou^1^, Elodie Teil^1^, Lamiae Grimaldi Bensouda^2^, Chrisitan Lamer^1^

##### ^1^Institut Mutualiste Montsouris, Paris, France; ^2^Department of Pharmacology, Clinical Research Unit, Hospital Group Paris-Saclay, Assistance Publique- Hôpitaux de Paris; UFR des Sciences de la Santé, University Paris-Saclay, Paris., Paris, France; ^3^Clinical Research Unit, Hospital Group Paris-Saclay, Assistance Publique- Hôpitaux de Paris, Paris, France

**Correspondence:** Nicolas Viault - n.viault@live.fr

*Annals of Intensive Care* 2021, **11(Suppl 1):**FC-072

**Rationale:** Many patients with COVID 19 require extended stay in ICU with heavy therapeutics and may have long-term persistent symptoms. Our objective is to evaluate functional respiratory outcome and quality of life (QoL) 6 months after ICU discharge for severe COVID 19.

**Patients and methods/materials and methods:** This is an observational, single-center and prospective, cohort study. All surviving patients admitted for COVID 19 in our ICU between March and September 2020, were contacted by telephone 6 months after ICU discharge to provide their respiratory status using the Medical Research Council (MRC) dyspnea score (a 1–5 stage scale used to determine clinical grades of breathlessness) and QoL using the EQ 5D scale (a health-related QoL in five dimensions: mobility, self-care, usual activities, pain/discomfort, and anxiety/depression). Each EQ 5D health state was converted into a single summary index (EQ 5D Index) using country-specific value sets. Patients were also asked to record their overall health status using a visual analogue scale (EQ-VAS, 0 to 100).

**Results:** Results are expressed as mean ± SD. Over the 7-month period, 67 patients (16 females/51 males, mean age 61 ± 12 years) were admitted in our ICU. SAPS 2 at admission was 33.4 ± 1.7. Their length of ICU stay was 20.2 ± 17 days. In-hospital mortality was 25% (18 patients). Among the discharged patients, 44 could be evaluated at 6 months; their SAPS 2 at admission was 30.1 ± 12 and length of ICU stay 20.1 ± 15 days. Out of these 44 patients, 29 (66%) were mechanically ventilated over 15.6 ± 16.6 days. At 6 months, 11 patients (25%) had a MRC dyspnea score ≥ 3 and, compared with the 33 patients with a dyspnea score < 3, showed: a lower EQ 5D Index (0.73 vs 0.95; *p* = 0.0024); a lower EQ-VAS (54.6 ± 21.9 vs 83.8 ± 13; *p* = 0.0002); and an impaired in all but one (anxiety/depression) QoL dimensions.

**Conclusion:** In our cohort, 25% of patients admitted for COVID 19 have disabling dyspnea 6 months after ICU discharge. Severity of dyspnea is associated with impaired quality of life. Follow-up of these patients is required to set up appropriate measures and assess their impact on respiratory status and quality of life.

**Compliance with ethics regulations:** Yes in clinical research.

### FC-073 Long-term respiratory sequelae of COVID-19

#### Camille Guizard^1^, Laurent Robriquet^1^, Ahmed Elkalioubie^1^, Sophie Six^1^, Arthur Simonnet^3^, Anne Sophie Moreau^1^, Patrick Girardie^1^, Saad Nseir^1^, Raphael Favory^1^, Sebastien Preau^1^, Julien Poissy^1^, Michele Catto^2^, Regis Matran^2^, Merce Jourdain^1^

##### ^1^CHU Lille, Pôle de Médecine Intensive et Réanimation, Hôpital Roger Salengro, Lille, France; ^2^CHU Lille, Service d’Exploration fonctionnelles respiratoires, Hôpital Albert Calmette, Lille, France; ^3^Centre hospitalier de Roubaix, Service de réanimation-unité de surveillance continue de l’adulte, Roubaix, France

**Correspondence:** Camille Guizard - guizard.camille@gmail.com

*Annals of Intensive Care* 2021, **11(Suppl 1):**FC-073

**Rationale:** Most critically ill patients with severe acute respiratory distress syndrome (ARDS) related to coronavirus disease 2019 (COVID-19) require invasive mechanical ventilation (MV). However, limited follow-up data are available on respiratory functional disability after Intensive Care unit (ICU) discharge. The study aimed to investigate respiratory functional disability at 6 months after ICU discharge among survivors of the COVID-19 ARDS requiring MV.

**Patients and methods/materials and methods:** We evaluated survivors of COVID-19 ARDS at 6 months after discharge from the ICU. Patients were interviewed and examined between September 2020 and January 2021; underwent pulmonary-function tests, the 6-min walk and cardiopulmonary exercise testing (CPET) with peak exercise blood gas.

**Results:** 38 mechanically ventilated patients with COVID-19 ARDS were included. Median age was 60 [26–76] years and 28 (74%) were male. The comorbidities included hypertension (39%), obesity (52%) and diabetes (26%). Median Simplified Acute Physiology Score II at ICU admission was 43 [12–76]. Median duration of MV was 12 [3–47] days and length of stay in ICU was 16 [5–64] days. 18 patients (47.6%) received early rehabilitation after ICU discharge. Pulmonary function tests were normal at 6 months. Median forced expiratory volume in 1 s was 99% [90–113], median forced vital capacity was 101% [88–119], median total lung capacity was 89% [84–104] and median carbon monoxide diffusion capacity was 75% [67–91]. Median distance walked in 6 min was 441 m [392–520]. Among CPET variables, median maximum oxygen consumption (VO2 peak) was decreased (< 84%) in 47% patients, 18.4 ml/min/kg [15.97–20.55]. Using multiple regression analysis, early rehabilitation was a significant predictor of VO2 peak improvement, (odds ratio [OR], 11.8; 95% confidence interval [CI], 1.2–115) and oxygen pulse was a significant predictor of VO2 peak impairment, (OR, 24; 95% CI, 1.5–58–387). VO2 peak alteration was not correlated with pulmonary-function tests.

**Conclusion:** In surviving patients requiring MV for COVID-19 ARDS, pulmonary-function tests were normal to near-normal at 6 months after ICU discharge. Exercise limitation was frequent with VO2 peak alteration in 47% patients. Our data also suggested that early rehabilitation after ICU discharge had protective effect on the VO2 decrease.

**Compliance with ethics regulations:** Yes in clinical research.

### FC-074 Tocilizumab for severe COVID-19 patients admitted in intensive care units: a retrospective multicentric analyze

#### Loay Kontar^1^, Antoine Riviere^2^, Eloi Goullieux^3^, Olivier Leleu^2^, Guillaume Flahaut^2^, Clément Brault^1^, Yoann Zerbib^1^, Mathieu Carpentier^1^, Ugo Fouquet^1^, Matthieu Metzelard^1^, Michel Slama^1^, Serge Redeker^2^, Julien Maizel^1^

##### ^1^CHU Amiens, Amiens, France; ^2^CH Abbeville, Abbeville, France; ^3^CH Laon, Laon, France

**Correspondence:** Julien Maizel - maizel.julien@chu-amiens.fr

*Annals of Intensive Care* 2021, **11(Suppl 1):**FC-074

**Rationale:** The SARS-CoV-2 induce both systemic and localized immune response leading to increased inflammation. Severe pulmonary lesions in SARS-Cov-2 ARDS has been found to be associated with severe inflammatory cell infiltration and elevated pro-inflammatory cytokines. In this context, the use of tocilizumab (a recombinant humanized monoclonal antibody against IL6 receptor) may reduce the consequences of SARS-Cov-2 on the different organs. Currently results from large multicentric studies reported some conflicting results. Here, we report our experience of tocilizumab started in severe Covid-19 hospitalized in ICU.

**Patients and methods/materials and methods:** We conducted a multicenter retrospective observational study between February 2020 and April 2020 in 3 French ICU. All patients admitted for a respiratory distress related to a COVID-19 were eligible and classified in two groups: group tocilizumab and group standard. According to a local procedure established in their institution by the intensivist, internist and pneumologist patients were eligible to receive tocilizumab if they were admitted in ICU for a severe respiratory distress due to COVID-19 with at least 6 l/min of oxygen, symptom of COVID-19 > 7 days, the absence of therapeutic limitations, a high concentration of CRP and a low procalcitonin concentration in plasma. Patients received 400 mg of tocilizumab intravenously and a second injection of 400 mg was possible 2 days later in case of persistent fever, high CRP and low PCT.

**Results:** Sixty patients were eligible: 14 patients group tocilizumab and 46 group standard of care with a similar delay since start of symptom (8 (6–10) and 9 (8–11) days; *p* = 0.0.4). At admission the severity scores (SAPS2 or SOFA score) were not different between both groups. Respectively 71% and 82% (*p* = 0.4) were mechanically ventilated between tocilizumab and standard groups with similar pO2/FiO2 ratio (102 (66–138) and 115 (60–170) *p* = 0.1, respectively). The group of patients who received the tocilizumab received less lopinavir/rotinavir (0% vs 37%, *p* = 0.007) or dexamethasone (9% vs 46%, *p* = 0.03) than the standard group. Between Tocilizumab and standard groups, the length of stay in ICU (11 vs 18 days, *p* = 0.4), duration of mechanical ventilation (13 vs 15 days, *p* = 0.4) or mortality at D28 (36% vs 26%, *p* = 0.5) or D90 (29% vs 30%, *p* = 0.9) were not different.

**Conclusion:** Our data do not show any improvement of outcome when tocilizumab was started at the admission in the ICU in patients with symptoms of COVID 19 lasting for more than 7 days.

**Compliance with ethics regulations:** Yes in clinical research.

### FC-075 High-dose corticosteroid therapy for severe COVID-19 in patients with overweight or obesity

#### Bruno Garcia, Mikael Chetboun, Julien Labreuche, Arthur Simonnet, Violeta Raverdy, Enagnon Alidjinou, Laurent Robriquet, Sophie Six, Ahmed El Kalioubie, Alain Duhamel, François Pattou, Mercé Jourdain

##### CHU LILLE, Lille, France

**Correspondence:** Bruno Garcia - br.garcia@icloud.com

*Annals of Intensive Care* 2021, **11(Suppl 1):**FC-075

**Rationale:** Severity of COVID-19 increases with body mass index. We analyzed the impact of high-dose corticosteroid therapy on clinical outcomes of SARS-CoV-2 in patients with overweight or obesity under invasive mechanical ventilation (IMV). Severity of COVID-19 increases with body mass index.

**Patients and methods/materials and methods:** In this retrospective cohort study, we analyzed the relation between high-dose corticosteroid therapy and invasive mechanical ventilation (IMV) withdrawal (primary end point) and death (secondary end point), in 150 consecutive patients with overweight or obesity (body mass index above 25 kg/m^2^), admitted in intensive care for SARS-CoV-2. Cumulative incidences were estimated with competing risk survival analysis. The impact of high-dose corticosteroid therapy was evaluated using cause-specific Cox’s regression models, by treating corticosteroid therapy as a time-varying covariable.

**Results:** The estimate of the 28-day mortality was 21.3% (95% CI, 15.2 to 28.2). A total of 109 patients (72.7%) required IMV, and among them, 58 received high-dose corticosteroid therapy after a median delay of 5 days (IQR, 2 to 8), for 8 days (IQR, 5 to 10). When considering the time of exposure, and following adjustment to age, body mass index and severity score at admission, high-dose corticosteroid therapy was associated with an increase in IMV withdrawal, with an adjusted HR of borderline of significance (HR, 1.62; 95%CI, 0.97 to 2.72), in reduction of IMV duration, and a non-significant decrease in mortality rate, with a HR of 0.60 (95% CI, 0.24 to 1.52).

**Conclusion:** In patients with overweight or obesity admitted in intensive care for SARS-CoV-2, high-dose corticosteroid therapy was associated with a reduction of IMV duration, and a non-significant reduction in point estimates of 28-day mortality. Overall, these results support a net benefit of corticosteroid therapy in severe COVID-19

**Compliance with ethics regulations:** Yes in clinical research.

### FC-076 Effect of late administration of high doses of corticosteroids in critically ill patients with Coronavirus Disease 2019 (COVID-19)

#### Bibi Yashirah Deebeely, Laurent Robriquet, Ahmed El Kalioubie, Sophie Six, Mercedes Jourdain, Julien Poissy, Raphael Favory, Sebastien Preau, Saadalla Nseir

##### CHU de Lille, Lille, France

**Correspondence:** Bibi Yashirah Deebeely - yashirah30@hotmail.com

*Annals of Intensive Care* 2021, **11(Suppl 1):**FC-076

**Rationale:** Acute respiratory distress syndrome (ARDS) related to COVID-19 is challenging. Early administration of low-dose corticosteroids (CTC) has been proved to be effective in severe COVID-19. However, no data are available on the impact of late administration of high doses of CTC to treat lung fibrosis in persistent ARDS. The study aimed to analyze the link between late high doses of CTC and clinical outcomes for COVID-19 ARDS patients.

**Patients and methods/materials and methods:** We conducted a retrospective study in the University Hospital Intensive Care Unit (ICU). All patients with COVID-19 ARDS receiving late high-doses of CTC between March 2020 and January 2021 were included. Fibrotic lung changes were assessed on medical history, respiratory mechanics evaluation and chest CT scans. Patients received a dose of 1 g per day of methylprednisolone equivalent during 3 days following by 1 mg/kg per day until ICU discharge.

**Results:** 22 patients were included. Median age was 66 [44–73] years. 19 patients (86%) were male. Comorbidities included hypertension (45.5%), obesity (27.3%) and diabetes (40.9%). Median Simplified Acute Physiology Score II at ICU admission was 47 [16–83]. 21 patients (95%) received invasive mechanical ventilation. 12 patients (55%) required extracorporeal life support, 21 (95.5%) received early low-doses of CTC. Median delay between tracheal intubation and late initiation of CTC was 13 [1–26] days. 15 patients (68%) presented a documented infection following high-doses of CTC (18% septicemia and 86% ventilation-associated pneumonia). On the day of high doses of CTC, median ratio of the PaO2/FiO2 was 111 mmHg [57–174], respiratory system compliance was 16.4 ml/cmH2O [2.9–41], plateau pressure was 29 cm H2O [24–31], driving pressure was 20 cm H2O [10–38], ventilator ratio was 1.48 [0.21–4.2] and C-reactive protein was 65 mg/l [14–160]. Patients ICU mortality was 50%. Using multiple regression analysis, no independent predictors of mortality were identified between survivors and nonsurvivors. Median delay between high doses of CTC administration and ICU discharge or death was 13 [3–53] days and was significantly increase in survivors vs nonsurvivors (21 [1–26] days vs 7 [1–24] days, *p* < 0.05).

**Conclusion:** This study of a cohort of persistent ARDS found no significant differences between survivors and nonsurvivors ICU patients after late administration of high doses of CTC. Longer delay in survivors between CTC administration and ICU discharge may suggest a necessary sufficient delay for the efficacy of the treatment. Furthers trials are needed to better define the use of late high doses of CTC as rescue therapy in profibrotic evolution of COVID-19 ARDS.

**Compliance with ethics regulations:** N/A.

### FC-077 Impact of prone position on prognosis of COVID-19-infected patients

#### Sana Kharrat, Rezk Ghorbel, Najeh Baccouch, Salma Jerbi, Kamilia Chtara, Ilef Alila, Ahmad Izidbih Yahya, Chokri Ben Hamida, Mabrouk Bahloul, Mounir Bouaziz

##### CHU Habib Bourguiba, Sfax, Tunisie

**Correspondence:** Sana Kharrat - sanakharrat15@hotmail.com

*Annals of Intensive Care* 2021, **11(Suppl 1):**FC-077

**Rationale:** Coronavirus disease (COVID-19) has become a global public health crisis. One of the most feared complications is acute respiratory distress syndrome (ARDS) due to its high mortality. The global experience of managing patients with COVID-19-associated ARDS is rapidly expanding. Several reports have assessed the feasibility and effectiveness of prone positioning in awake, non-intubated patients with COVID-19, but benefits on mortality and the ulterior need of mechanical ventilation remain controversial. We conducted this study to evaluate the effectiveness of prone position in the management of critically ill patients.

**Patients and methods/materials and methods:** We report the experience of an intensive care unit between September 2020 and January 2021. We included all patients with confirmed SARS-COV2 infection developing severe ARDS without ventilatory assistance. Respiratory parameters were measured at 2 time points: on supine position and during proning. Primary outcome measures were the change in oxygen saturation (SpO2), respiratory rate and the presence of signs of respiratory struggle. The secondary outcome was the incidence of intubation and mortality rate.

**Results:** A total of 79 patients were included. 64 (81%) experienced prone position during spontaneous breathing with high-flow nasal oxygen or on non-re-breather mask. A significant improvement in oxygenation was found from supine to prone positioning in 58 (90.6%) patients (responders). Respiratory rate decreased from 30.4 ± 12.4 to 21.3 ± 4.7 (*p* < 0.001). Oxygen saturation increased from 88.1 ± 9.7 to 95.7 ± 3.7 (*p* < 0.001). Signs of respiratory struggle disappeared in the 78.1% of cases. Non-responders to proning evidenced a higher mortality (83.3% versus 34.5%; *p* = 0.02; OR = 9.5). No differences in mortality or in rates of intubation were seen in those who experienced proning compared with those who refused it.

**Conclusion:** While our results may not show statistically significant effects on mortality, we clinically observed improvement in respiration status of patients by applying prone positioning which makes this strategy feasible and a useful option in the management of acute respiratory failure due to this disease. Other studies are needed on this subject.

**Compliance with ethics regulations:** N/A.

### FC-078 Response to prone positioning in SARS-CoV2 mechanically ventilated patients

#### Margot Combet, Arthur Pavot, Christopher Lai, Quentin Fossé, Soufia Ayed, Ludvik Jelinski, Florian Lardet, Nicolas Fage, Laurent Guérin, Nadia Anguel, David Osman, Jean-Louis Teboul, Xavier Monnet, Tài Pham

##### Hôpital Bicêtre, Le Kremlin-Bicêtre, Paris, France

**Correspondence:** Margot Combet - margotcombet@gmail.com

*Annals of Intensive Care* 2021, **11(Suppl 1):**FC-078

**Rationale:** Intensive care units worldwide face an overwhelming number of patients with SARS-CoV2-associated ARDS. Some authors [1] have hypothesized the existence of two phenotypes in intubated patients with SARS-CoV2: type L for low elastance, low shunt and low lung weight and type H for high elastance, high shunt and high lung weight. It has been suggested that these phenotypes may benefit from different management in ICU in terms of tidal volume, PEEP and prone positioning [1, 2]. We aimed to assess response to prone positioning in SARS-CoV2-patients and according to ARDS phenotype.

**Patients and methods/materials and methods:** This is a single-center study in a French medical ICU. We included patients receiving mechanical ventilation for SARS-CoV2-ARDS and undergoing at least one proning session. We categorized patients in the L or H groups according to their respiratory system compliance before the first proning session: patients with a respiratory compliance ≥ 40 mL/cmH2O were classified in the L group. We considered patients to be prone responders if they fulfilled at least one of the three following criteria: an increase in PaO2 to FiO2 (PF) ratio ≥ 20 mmHg, a PF ratio increase ≥ 20% or a decrease in PaCO2 of at least 5 mmHg.

**Results:** Seventy patients were included in our study. Median age was 61 ± 12 years and median SOFA on admission was 6 ± 4. Overall mortality was 65%. Most patients (85%) had low compliance < 40 mL/cmH2O before prone positioning. Respiratory characteristics before prone positioning and response to proning are shown in Table 1. Type L patients were taller, and consequently received higher tidal volume. Tidal volume standardized with predicted body weight was similar in both groups. Most patients (74%) met at least one criterion for response to proning. Patients with high compliance had higher initial PaCO2 and a non-statistically significant rate of patients having a PaCO2 decrease of 5 mmHg or more.

**Discussion:** Difference in height and tidal volume may impact respiratory system compliance calculation. Though non-significant, decrease in PaCO2 with proning in the group of patients with lower elastance suggests benefits of the procedure by limiting overdistension and dead-space.

**Conclusion:** Most patients with SARS-COV2-associated ARDS presented altered compliance and responded to proning, regardless of their initial respiratory system compliance. Further research is needed to better understand the determinants of L and H phenotypes and their responses to prone position in terms of PCO2 reduction.

**References**

1. Gattinoni L, Chiumello D, Rossi S. Covid 19 pneumonia: ARDS or not? Critical Care; 2020.
2. Fan E, Beitler J, Brochard L et al. COVID-19-associated acute respiratory distress syndrome: is a different approach to management warranted? Lancet Respir Med; 2020.

**Compliance with ethics regulations:** Yes in clinical research.
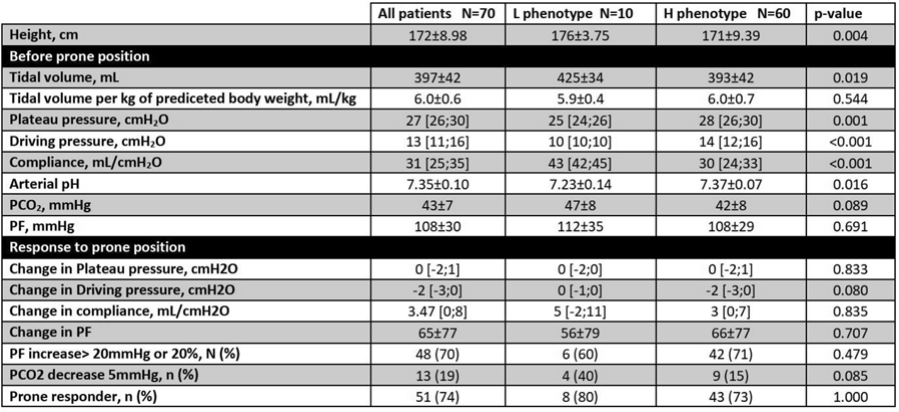


Respiratory parameters before and during prone positioning session. Data are presented as mean ± standard deviation, median [interquartile range] or number (percentage)

### FC-079 Evolution of the response to prone position in patients with SARS-CoV-2 infection-related ARDS

#### Ludwik Jelinski, Margot Combet, Florian Lardet, Christopher Lai, Shi Rui, Francesca Moretto, Arthur Pavot, Quentin Fossé, Guillaume Roger, Thibaut Gobé, Nicolas Fage, Imane Adda, Laurent Guérin, Jean-Louis Teboul, Xavier Monnet, Tài Pham

##### Hôpital Bicêtre, Kremlin-Bicêtre, France

**Correspondence:** Ludwik Jelinski - jelinski.l@hotmail.fr

*Annals of Intensive Care* 2021, **11(Suppl 1):**FC-079

**Rationale:** Prone position was shown to decrease mortality in patients with the most hypoxemic forms of the acute respiratory distress syndrome (ARDS) [1]. The factors associated with gas exchange improvement during prone position as well as the evolution of the response to prone over successive sessions are currently unknown.

**Patients and methods/materials and methods:** This single-center observational study was performed in a medical ICU between March 2020 and February 2021. Patients receiving mechanical ventilation for SARS-CoV2-ARDS who had at least 2 proning sessions with respiratory mechanics measurement and arterial blood gas drawn before and at the end of both sessions were included. We considered patients to be prone responders if they fulfilled at least one of the following criteria: a PaO2-to-FiO2 (PF) ratio increase ≥ 20 mmHg; a PF ratio increase ≥ 20%; and/or a decrease in PaCO2 ≥ 5 mmHg. This study aimed at determining the characteristics of the patients benefiting from prone position and the evolution of this response between the first two sessions of prone position.

**Results:** A total of 46 patients were included in this analysis, aged [mean (SD)] 62 (11), with a BMI of 29 (6) kg/m^2^, admission SAPS2 of 41 (14) and SOFA of 6 (4). Half of them had hypertension, 37% diabetes and 17% chronic kidney disease (comprising 7% with chronic dialysis). Most patients were responders (70%, *n* = 32) to the first proning session, including 8 (17%) not responding to the second session; 33 patients were responders to the second session (72%), including 9 (20%) who were not responders to the first one. Finally, 5 patients (10%) were not responders to any of the proning sessions. Responders to both sessions had lower PF and pH before the 1st session (Table). At the moment of the analysis, 7 patients were still intubated in the ICU and ICU survival (excluding these patients) was low: 20% in patients who did not respond to at least one of the proning session and 38% in patients who responded to both sessions (*p* = 0.35)

**Conclusion:** Patients with COVID-19-related ARDS requiring several proning sessions have a high mortality. Gas exchange improvement with prone position is highly variable between patients and within patients from one session to another. Patients with most severe initial arterial blood gas seem to show greater benefit in terms of gas exchange.

**Reference**

1. Guérin C, Reignier J, Richard J-C, Beuret P, Gacouin A, Boulain T, et al. Prone positioning in severe acute respiratory distress syndrome. N Engl J Med. 2013;368:2159–68.

**Compliance with ethics regulations:** Yes in clinical research.
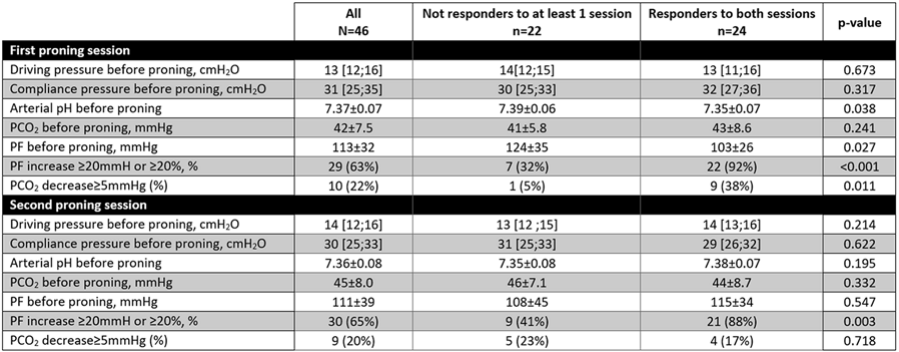


Table Ventilatory parameters in responders and non-responders to prone sessions

### FC-079bis Knowledge of paramedical professionals about tracheostomy in respiratory weaning unit and intensive care unit

#### Jean-Baptiste Peretout^1^, Laura Federici^2^, Noémie Zucman^2^, Laurent Serpin^3^, Jean-Damien Ricard^2^, Gérald Choukroun^1^

##### ^1^Service de Réadaptation Post-Réanimation respiratoire, Hôpital Forcilles, Ferolles-Attilly, France; ^2^Service de Médecine Intensive Réanimation, Hôpital Louis-Mourier, Colombes, France; ^3^Service de Réanimation polyvalente, Hôpital Ajaccio, Ajaccio, France

**Correspondence:** Jean-Baptiste Peretout - jperetout@cognacq-jay.fr

*Annals of Intensive Care* 2021, **11(Suppl 1):**FC-079bis

**Rationale:** The proportion of patients with tracheostomy in intensive care units (ICU) remains unclear (5–54%) (1) and the paramedical experience is unknown. The use and management of tracheostomy cannulas require specific knowledge to prevent misuse and complications. The objective of this study was to compare the knowledge of paramedical professionals about tracheostomy in a respiratory weaning unit and in intensive care units (ICU).

**Patients and methods/materials and methods:** This prospective, observational, multicentric study was conducted in one weaning unit and two ICU in France. A 10-question survey was distributed to paramedical professionals (nurses and care assistants [CA]) to assess their basic knowledge about cannulas choice and management of breathing in tracheostomy patients. The percentage of correct answers was analyzed.

**Results:** In the 3 units, 88 surveys were completed: 34 in the weaning unit (19 nurses, 15 CA), 32 in ICU1 (18 nurses, 14 CA) and 22 in ICU2 (16 nurses, 6 CA). The percentage of correct answers was higher in the respiratory weaning unit compare to the ICUs: 60% (67.7; 52.3) vs 41% (47.6; 34.2) for ICU 1 and 36% (47.3; 24.5) for ICU 2, respectively (*p* < 0.001) [Fig. 1]. No difference between nurses and care assistants was found in the weaning unit (*p* = 0.09), ICU1 (*p* = 0.16) or ICU2 (*p* = 0.15).

**Conclusion:** Knowledge of paramedical professionals about tracheostomy was better among paramedics working in a weaning unit compared to those in intensive care units without difference between professionals in the three units. This suggests that ventilator weaning of patients with tracheostomy, when recovered from other organ failures, should be conducted in specialized respiratory units.

**Reference 1**

1. Blot F, Melot C. Commission d’Epidémiologie et de Recherche Clinique. Indications, timing, and techniques of tracheostomy in 152 French ICUs. Chest. 2005;127(4):1347–52.

**Compliance with ethics regulations:** N/A.
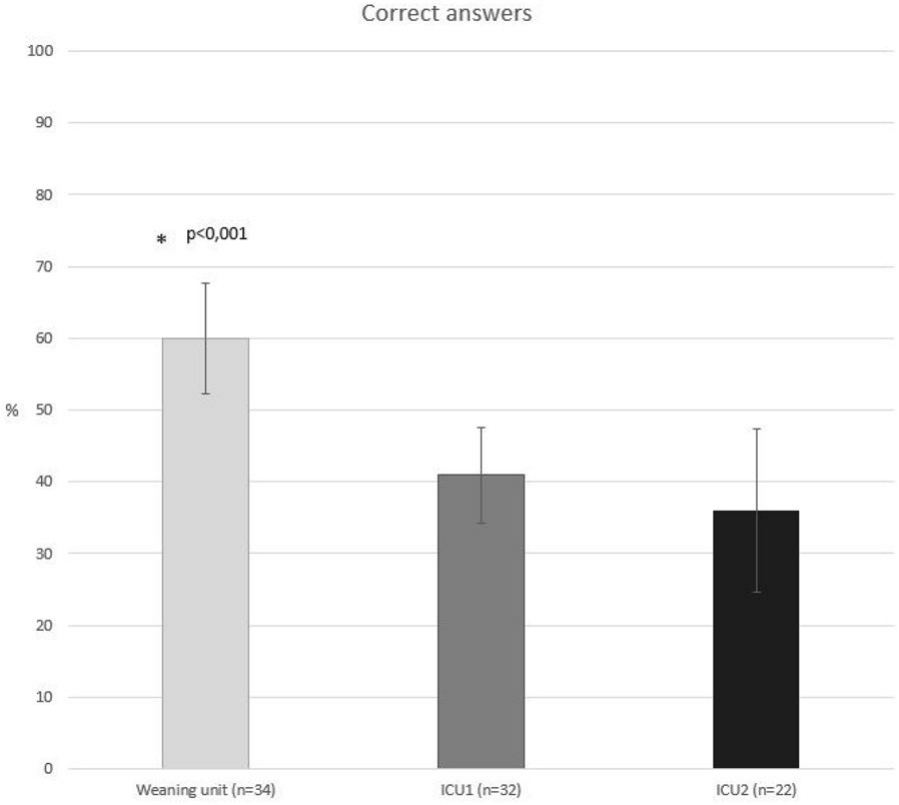


Fig. 1 Correct answers by unit

### FC-080 Prone position during ECMO in COVID hypoxaemic patient

#### Younes Oujidi, Houssam Bkiyar, Brahim Housni

##### Centre Hospitalier universitaire Mohammed VI, Oujda, Oujda, Maroc

**Correspondence:** Younes Oujidi - younesoujidi@gmail.com

*Annals of Intensive Care* 2021, **11(Suppl 1):**FC-080

**Rationale:** The main aim of this study was to investigate the modification of the PaO2/FiO2 ratio, the compliance of the respiratory system in patients under VV-ECMO with refractory hypoxemia. The secondary objective was to evaluate the safety and feasibility of the inclined position for ECMO.

**Patients and methods/materials and methods:** We retrospectively reviewed the electronic records and lists of all 23 COVID-19 patients who were placed for the first time in prone position (PP) with an average duration of 16 h (Fig. 1). Patient characteristics, pre-ECMO characteristics, ventilator/ECMO settings, and changes in ventilator/ECMO settings and blood gas analysis before and after PP were recorded.

**Results:** A total of 23 eligible PP were performed, the PaO2/FiO2 ratio and respiratory compliance improved 16-h PP without accidents during PP. PaO2/FiO2 increased from 64.57 mmHg [48–69] to 134.71 mmHg [110–200] and respiratory system compliance from 12.86 mL/cmH2O [6.66–19] to 17.07 [12.94–21.11].

**Conclusion:** The use of PP during VV-ECMO demonstrated an improvement in oxygenation (PaO2/FiO2) as well as of lung compliance, to be placed in perspective with the safety of the procedure, which was agreed to be a safe and reliable technique.

**Compliance with ethics regulations:** Yes in clinical research.
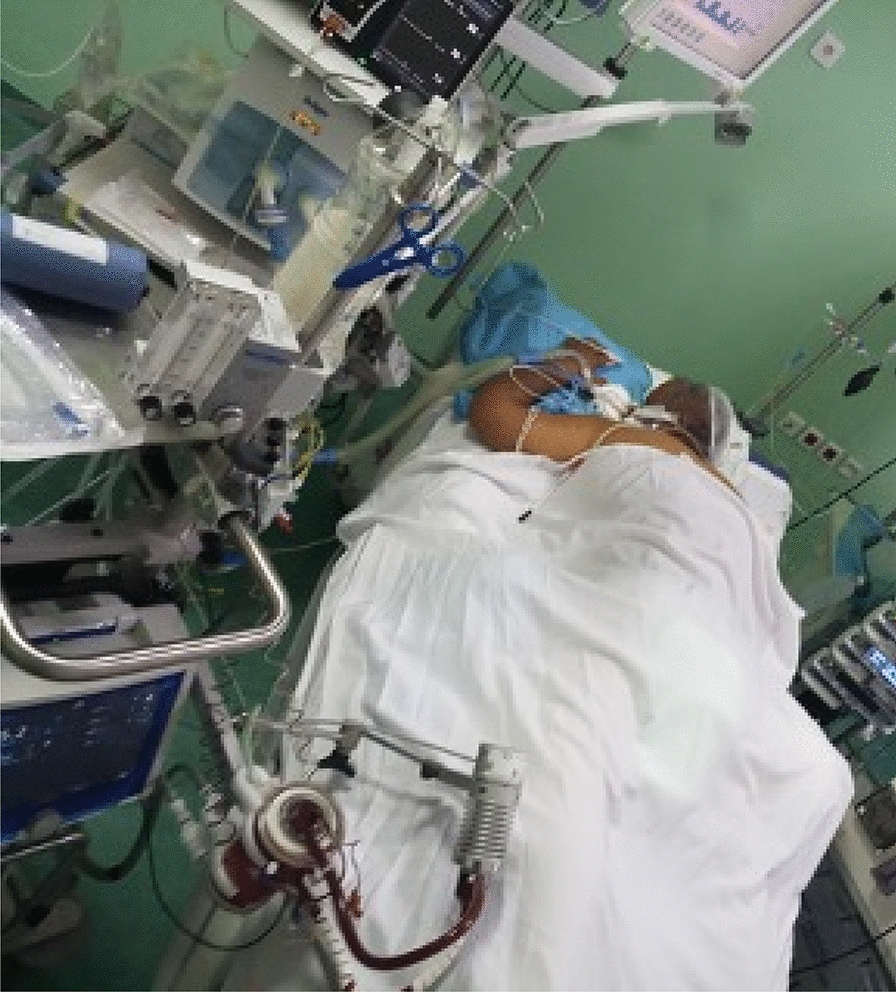


Fig. 1 Patient in prone position with oral intubation

### FC-081 Low-PEEP mechanical ventilation strategy: is it a new approach for COVID-19 patients management?

#### Samuele Ceruti^1^, Andrea Saporito^3^, Maira Biggiogero^1^, Andrea Glotta^1^, Patrizia Urso^1^, Alain Borgeat^2^, Christian Garzoni^1^

##### ^1^Clinica Luganese Moncucco, Lugano, Suisse; ^2^Balgrist University Hospital, Zürich, Suisse; ^3^Ospedale Regionale di Bellinzona e Valli, Bellinzona, Suisse

**Correspondence:** Ceruti Samuele - samuele.ceruti@moncucco.ch

*Annals of Intensive Care* 2021, **11(Suppl 1):**FC-081

**Rationale:** Critically ill COVID-19 patients were worldwide burdened by high mortality rate. In our Center, the assessment to improve patients’ management was performed developing standard criteria for ICU admission, a low-PEEP strategy as standard intensive care treatment regarding invasive mechanical ventilation (IMV) in agreement with the ARDSnet table and other associated features, like thromboembolism. We reported as primary outcome the effects of low-PEEP strategy on P/F ratio evolution during IMV; moreover, ICU length-of-stay (LOS), length of the IMV and ICU mortality rate was reported as secondary outcomes.

**Patients and methods/materials and methods:** A retrospective analysis was conducted on consecutive patients with COVID-19 with acute respiratory distress syndrome admitted to ICU from March 2nd to April 10th, 2020. Patients were treated with a low-PEEP strategy according to BMI (PEEP 10 cmH_2_O if BMI < 30 kg/m^2^, PEEP 12 cmH_2_O if BMI 30–50 kg/m^2^, PEEP 15 cmH_2_O if BMI > 50 kg/m^2^).

**Results:** Thirty-four patients were on IMV. The mean PEEP applied was 11 ± 3.8 cmH_2_O for BMI < 30 kg/m^2^, 15 ± 3.26 cmH_2_O for BMI > 30 kg/m^2^. No patients presented BMI > 50 kg/m^2^. After low-PEEP application, patients’ P/F ratio showed a daily improvement from ICU admission over the next 48 (*p* < 0.001; 95% CI) and 72 h (*p* < 0.001; 95% CI). This improvement was significant when comparing the P/F ratio before and after orotracheal intubation (OTI). The mean ICU LOS was 11 days (6–18); the median IMV duration was 8 days (5–13); the ICU mortality rate was 24.3%.

**Conclusion:** Low-PEEP strategy resulted in steady improved P/F ratio, shorter IMV length, ICU LOS, and lower mortality rate compared to high-PEEP standard treatment.

**References**

1. Gattinoni L, Coppola S, Cressoni M, et al. COVID-19 does not lead to a “typical” acute respiratory distress syndrome. American Journal of Respiratory and Critical Care Medicine 2020; 201: 1299–300.
2. Bendjelid K, Raphaël G. Treating hypoxemic patients with SARS-COV-2 pneumonia: Back to applied physiology. Anaesthesia Critical Care and Pain Medicine 2020; 39: 389–90.

**Compliance with ethics regulations:** Yes in clinical research.
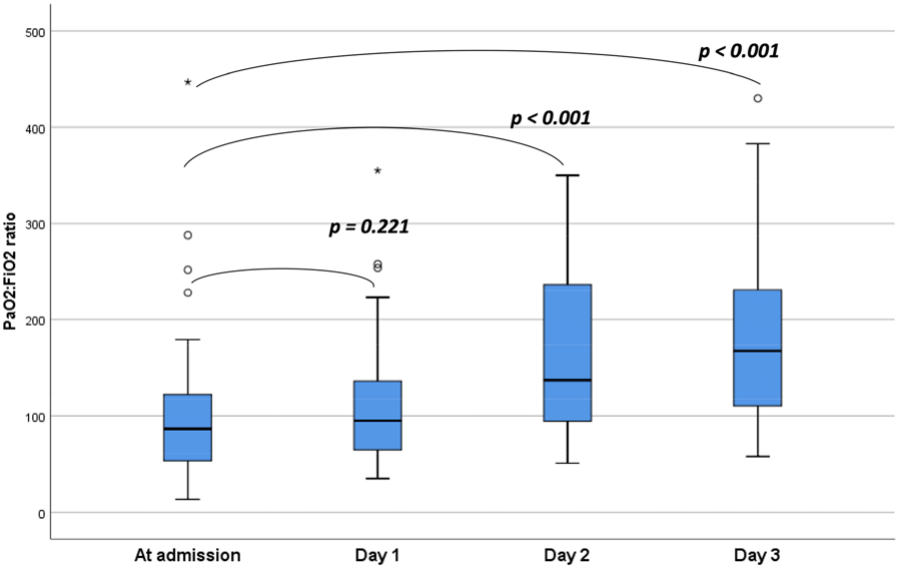


P/F ratio variation at ICU admission compared to the first, second and third day of MV. All daily median PF-valued resulted significantly different compared to admission, even with the use of low-PEEP setting on MV

### FC-082 The clinical, laboratory profile and outcome of covid-19 patients hospitalized in an ICU in Tunisia

#### Sawsen Ben Nessira, Yosr Touil, Emna Abid, Foued Daly, Ahlem Trifi, Cyrine Abdennebi, Sami Abdellatif, Salah Ben Lakhal

##### hôpital la RABTA (tunis), Tunis, Tunisie

**Correspondence:** Yosr Touil - touilyosr@hotmail.fr

*Annals of Intensive Care* 2021, **11(Suppl 1):**FC-082

**Rationale:** COVID-19 disease caused by SARS-Cov-2 coronavirus began in Wuhan, China, in December 2019. Patients can develop acute respiratory distress syndrome, which require admission in the intensive care unit. Objective: To determine the clinical, biological characteristics and the outcome of COVID-19 patients admitted in the ICU.

**Patients and methods/materials and methods:** A retrospective observational study was conducted in the medical ICU of the tertiary university hospital la Rabta in Tunis (Tunisia) between September 2020 to January 2021. Patients with laboratory confirmed SARS-CoV-2 infection (RT PCR) and a PaO2/FiO2 ratio under 300 were enrolled. This data includes gender, age, co-morbidities, clinical symptoms and different ventilatory support.

**Results:** 49 patients were included with a PaO2/FiO2 of 87 [63–108.5], and a SOFA score of 4 [3–4]. Median age was 63 years [56–71.5] and 75.5% were men. The main co-morbidities were arterial hypertension (46.9%) and diabetes (34.7%). Median BMI was 27 [25–30.7]. The most common symptoms reported were dyspnea (85.7%), fever (73.5%), cough (69.4%) and myalgia (34.7%). Concerning the biological tests, 41 patients (83.7%) had lymphopenia, 35 (71.4%) had hyperleucocytosis, 40 (81.6%) had elevated CRP levels, 28 (57.1%) had elevated serum LDH levels and 19 (38.8%) had cytolysis. Twenty-nine (59.2%) patients had moderate-to-severe degree of pulmonary involvement on chest CT scan. Regarding the ventilatory support, 20 (40.8%) patients were treated with high-flow nasal cannula, 19 (38.8%) with conventional oxygen therapy. Among these cases, 34 (69.4%) received non-invasive ventilation. Finally, 10 patients (20.4%) needed immediate endo-tracheal intubation. As for the evolution, 25 patients (51%) required endo-tracheal intubation, 4 patients (8.2%) developed myocarditis, and only one patient had a pneumothorax. The mortality rate was 65.3%.

**Conclusion:** Covid-19 patients in our unit had a high mortality rate. More studies should be conducted to help provide guidance in the clinical management of this pandemic.

**Compliance with ethics regulations:** Yes in clinical research.

### FC-083 Predictive factors of non-invasive ventilation failure in COVID 19

#### Feriel Ben Aba, Hamdi Hemden Doghri, Nadia Zarouane, Ines Sdiri, Imen Zaghdoudi, Nabiha Borsali Falfoul

##### Université de Tunis El Manar, Faculté de Médecine de Tunis. Hôpital Habib Thameur. Service des urgences et de réanimation médicale, Tunis, Tunisie

**Correspondence:** Feriel Ben Aba - benaba.feriel@gmail.com

*Annals of Intensive Care* 2021, **11(Suppl 1):**FC-083

**Rationale:** The SARS COV 2 infection was responsible of numerous cases of acute respiratory failure that needed intensive care. The management of these severe forms relies on ventilatory support. Even though non-invasive ventilation (NIV) was initially controversial, it remains highly used. Our objective was to assess predictive factors of NIV failure in critically ill COVID 19 patients.

**Patients and methods/materials and methods:** Descriptive, prospective and monocentric study including all patients admitted in our department between 09/07/2020 and 12/31/2020. All data including ventilation parameters and outcomes were taken from patient’s medical files.

**Results:** 67 patients infected by the SARS COV 2 were admitted in our department. Sex ratio was 1.57 and median age was 64 years. 37 patients (55.2%) had arterial hypertension, 24 (35.8%) diabetes mellitus and 31 (46%) obesity.15 patients (22.4%) had smoking habits. Time period between symptoms onset and admission had a median value of 7 days [6,10]. Median SAPS II was 35. Thirty-one patients had a severe form of acute respiratory distress syndrome (ARDS), 32 had a moderate form, and 4 had a mild form. NIV was applied in 47 cases (70%). The number of ventilation sessions and their duration in hours were, respectively, three [2–3] and four [4–12] as median values. Median PaO2/FiO2 ratio and FiO2 were, respectively, 124 [94,175] and 0.7 [0.5–1]. The average positive end-expiratory pressure (PEEP) and pressure support ventilation were, respectively, 8 cm H2O ± 1.16 and 10.8 cm H2O ± 1.42. The need of invasive ventilation (IV) was inevitable in 19 cases. 13 patients died. In univariate analysis, NIV failure was associated significantly to an age above 68 years (*p* < 10–3), a severe form of ARDS (*p* < 10-3) and SAPS II score above 29 (*p* < 10–3). It showed that C-reactive protein > 150 mg/l and fibrinogen > 6.5 g/l (*p* < 10–3) were also associated to NIV failure. Complications such as acute kidney failure and nosocomial infections were associated significantly to NIV failure. In a multivariate analysis, 2 independent factors predictive of NIV failure were found: Severe form of ARDS at admission (OR:18.1; 95% CI: 1.01–321; *p* = 0.049), and acute kidney failure (OR: 21.8; 95% CI: 1.70–281; *p* = 0.018). NIV failure was associated to an extended ICU stay (*p* < 10-3) and a higher mortality (*p* < 10-3).

**Conclusion:** A severe form of ARDS at admission and acute kidney failure were reported in our study as independent risk factors predicting NIV failure. This therapeutic complication is associated to an extended ICU stay and a higher mortality.

**Compliance with ethics regulations:** Yes in clinical research.

### FC-084 Efficiency and safety of non-invasive mechanical ventilation in critically ill COVID-19 patients

#### Khaoula Ben Ismail, Boudour Ben Dhia, Najla Ben Slimene, Fatma Essafi, Moez Kaddour, Takoua Marhabene

##### Hopital regional de Zaghouan, Zaghouan, Tunisie

**Correspondence:** Khaoula Ben Ismail - khaoula87@hotmail.fr

*Annals of Intensive Care* 2021, **11(Suppl 1):**FC-084

**Rationale:** Use of non-invasive mechanical ventilation (NIMV) has become very popular in our units even in patients with acute hypoxemic respiratory failure (AHRF). However, its high infectiousness and particle dispersion capabilities generate controversy regarding its use as a respiratory support to patients with active COVID-19 infections. Our aim was to explore the feasibility, safety and efficacy of NIMV in the treatment of AHRF and acute respiratory distress syndrome (ARDS) due to COVID19.

**Patients and methods/materials and methods:** We performed this retrospective cohort study in COVID-19 patients hospitalized in Zaghouan’s Hospital ICU between 24th March 2020 and 31st January 2021. Need for invasive mechanical ventilation (IMV) and mortality rate in patients treated with NIMV were assessed according to different ventilatory support modalities. It includes continuous positive airway pressure (CPAP), non-invasive pressure support (NIPSV) and high-flow nasal cannula oxygen therapy (HFNC).The later was applied alone in the ARDS mild group.

**Results:** One hundred twenty-one critically ill COVID-19 patients were hospitalized. All patients met the Berlin criteria for ARDS. At admission, NIMV was directly applied coupled with awake prone position in 83% patients. It consisted on NIPSV in 32% of cases, CPAP in 29%, HFNC with NIPSV in 22%, HFNC with CPAP in 12%, HFNC alone in 0.5%. Secondary need for invasive mechanical ventilation (IMV) was required in 50 patients (41.3%), it was within the first 48 h in 23 cases. Rates of intubation in each group were: 72% in NIPSV, 83% in HFNC with CPAP, 41% in CPAP, 21% in HFNC in NIPSV and no need to IMV in HFNC. Median static respiratory compliance in intubated patients was 28 mL/cm H2O[12–54]. All cases were treated with daily prone positioning, profound sedation and neuromuscular blockade. Medians ventilatory parameters were: PEEP = 12 cm H2O [6–14], driving pressure = 14 cm H2O [4–24] and tidal volumes = 375 ml [300–450]. Most reported pulmonary complications associated with IMV were reventilation collapse in 44%, healthcare associated pneumonia in 21.2%, and barotrauma in 2% of cases. Median duration of NIMV in the success group was 9[2–30] days whereas that of IMV was 6 [1–19]. We reported no incident of viral contamination in our staff. Global mortality rate was 43.8%; it was 90% in the IMV group vs 13.8% in NIMV group (OR: 6.4; *P* < 0.0005).

**Conclusion:** Our study confirmed that NIV was an appropriate bridging adjunct in the early of the COVID-19 disease. Success seems to depend on choice of ventilator modality.

**Compliance with ethics regulations:** Yes in clinical research

### FC-085 Predictors of failure of high-flow nasal oxygen therapy in critically ill COVID-19 patients with acute respiratory failure

#### Wael Chamli, Oussama Jaoued, Hajer Nouira, Mayssem Abdelkarim, Rim Gharbi, Habiba Ben Sik Ali, Mohamed Fekih Hassen, Souheil Atrous

##### service de réanimation médicale hôpital Taher Sfar, Mahdia, Tunisie

**Correspondence:** Oussama Jaoued - oussamajaoued@gmail.com

*Annals of Intensive Care* 2021, **11(Suppl 1):**FC-085

**Rationale:** High-flow nasal oxygen (HFNO) which reduces the need for endotracheal intubation in patients with acute respiratory failure, is now widely used as first line oxygen delivery means in patient with acute respiratory failure (ARF) caused by COVID-19 pneumonia. The aim of the study is to identify the predictive factors of HFNO failure (shift to invasive mechanical ventilation (IMV)) in patient with ARF.

**Patients and methods/materials and methods:** This was a single-center, observational study performed between October and December 2020. We included patients aged more than 18 years with confirmed SARS-CoV-2 pneumonia and receiving HFNO at admission. Patients intubated within 24 h were excluded. Two groups were identified: HFNO success group (patients discharged from ICU without requiring IMV) and HFNO failure group (requirement of IMV). Collected data: demographic characteristics, time from illness onset to ICU admission, clinical parameters, SOFA score and ROX-index at admission, thoracic CT results, length of stay on ICU and ICU mortality.

**Results:** A total of 70 patients were included. The common comorbidities were diabetes (43%) and hypertension (54%). Mean SOFA and APACHEII were, respectively, 3.9 ± 1.6 and 10 ± 3.5. Time from illness onset to ICU admission was 8 ± 4 days. Mean Rox-index was 3.9 ± 1.1. Prone position (PP) was used on 49% of patients. The mean duration of HFNO was 5.5 ± 2 days and the mean duration of ICU stay was 15 ± 10 days. HFNO failure rate was 43% and mortality rate was 33%. APACHEII score and SOFA scores were higher in the HFNO failure group (12 ± 4vs 8 ± 2, *p* < 10-3 and 4.8 ± 2 vs 3.2 ± 0.5, *p* < 0.001). Time from illness onset to ICU admission was shorter in the HFNO success group (6.5 ± 3 vs 9.5 ± 5 days, *p* = 0.008). PP was more commonly used in the HFNO failure group (88% vs 54%, *p* = 0.005). Respiratory rate (RR) was higher in the failure group (33 ± 4 vs 28 ± 4, *p* = 0.008) and PaO2 to FiO2 ratio was lower in the same group (77 ± 25 vs 108 ± 39, *p* < 0.001). ROX-index was higher in the HFNO success group (4.5 ± 1 vs 3 ± 07, *p* < 0.001). The accuracy of the ROX-index to predict intubation was good [AUC = 0.844 (0.754–0.689), *p* < 0.001]. The cutoff of Rox-index > 3.42 had the best sensibility (84%) and specificity (70%). Lung CT damage > 75% was more frequently observed in the failure group. In multivariate regression analysis, SOFA score (OR = 5.9, 95%IC[3.1–19.7], *p* = 0.001) and ROX-index > 3.42 at admission (OR = 0.22, 95%IC[0.05–0.89], *p* = 0.034) were independently associated with HFNO failure.

**Conclusion:** A ROX-index less than 3.42 appears to have a good ability to predict intubation in SARS-COV2 patient with acute respiratory failure.

**Compliance with ethics regulations:** Yes in clinical research

### FC-086 Inappropriate heart rate response to hypotension in COVID-19 intensive care patients

#### Charles Verney^2^, David Legouis^3^, Guillaume Voiriot^1^, Muriel Fartoukh^1^, Vincent Labbe^1^

##### ^1^Hôpital Tenon, Paris 20, France; ^2^Hôpital Louis Mourier, Colombes, France; ^3^Hôpital universitaire de Genève, Genève, Suisse

**Correspondence:** Charles Verney - charlesverney@gmail.com

*Annals of Intensive Care* 2021, **11(Suppl 1):**FC-086

**Rationale:** Human cell infection by SARS-CoV-2 leads to ACE2 intracellular pathway downregulation [1]. ACE2 is involved in the baroreflex pathway. Therefore, SARS-CoV-2 could alter responses to hypotension in COVID-19 patients. To assess baroreflex function in COVID-19 intensive care patient, we aimed to compare heart rate (HR) variations in response to dialysis-induced hypotension between COVID-19 and non-COVID-19 septic patient.

**Patients and methods/materials and methods:** We screened all consecutive patients admitted to the Intensive Care Unit (ICU) of the Tenon university hospital (Paris, France) who underwent CVVHDF with ongoing net ultrafiltration and stable catecholamine infusion level from February 2018 to April 2020. Patients who experienced DIH defined as a systolic BP < 90 mmHg with a fall > 30 mmHg (or mean BP < 65 mmHg with a fall > 10 mmHg) were enrolled. Exclusion criteria were non-sinus rhythm, administration of medications inducing bradycardia at the day of DIH onset (cardio-vascular medication including betablockers, neuroleptic, dexmedetomidine), therapeutic modification 2 h before DIH (fluid loading, ultrafiltration, sedative drug), and neurologic disease. Endpoint was the HR variation defined as the HR at DIH onset minus last HR before DIH.

**Results:** Overall, 6 COVID-19 patients (66.7% men; age 58[53–64] years) and 12 non-COVID-19 septic patients (58.3% men; age 67[51–71] years) were included. Baseline characteristics, laboratory finding, and hemodynamic parameters before DIH were similar between the two groups. Catecholamine, dialysis settings and sedative drugs infusion before DIH were similar between two groups except for propofol and midazolam infusion rates that were significantly higher in COVID-19 group (200[200–200] mg/h vs. 160[75–200] mg/h, *p* = 0.02; 4.0[0.8–5.0] mg/h vs. 0 [0–0] mg/h, *p* = 0.02; respectively). DIH occurred with a similar time from CVVHDF initiation between COVID-19 group and non-COVID-19 group (4[1–7] vs. 5[2–7], *p* = 0.70). DIH was similar between the two groups regarding absolute values of and variation of BP. The HR variation in COVID-19 group was significantly lower than those in non-COVID-19 group (− 7[− 9–− 2] vs. 2[2–5] bpm, *p* = 0.003, Fig. 1). After adjustment on absolute HR at DIH onset and infusion rates of propofol, midazolam, and sufentanil, COVID-19 status remains independently associated with a lower HR variation (− 10[− 16 to − 4] bpm; *p* = 0.004).

**Discussion:** While numerous COVID-19 cardiovascular involvements have been reported including myocarditis, arrhythmias, and bradycardia, ANS dysfunction has not been yet described [2].

**Conclusion:** In conclusion, we report inappropriate heart rate response to hypotension in COVID-19 intensive care patients, probably related to ANS dysfunction. Intensivists should be aware of this cardiovascular involvement for hemodynamic management of these patients.

**References**

1. Xia H, Lazartigues E. Angiotensin-converting enzyme 2: central regulator for cardiovascular function. Curr Hypertens Rep. 2010; 12:170–5. 10.1007/s11906-010-0105-7
2. Amaratunga EA, Corwin DS, Moran L, Snyder R. Bradycardia in patients with COVID-19: a calm before the storm? Cureus. 2020; 12:e8599. 10.7759/cureus.8599

**Compliance with ethics regulations:** Yes in clinical research.
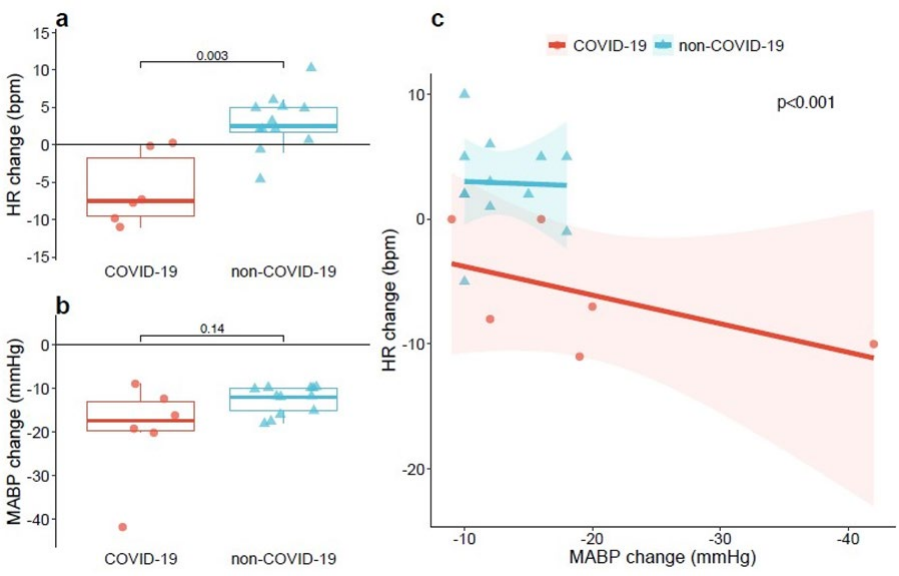


Variation of the heart rate (**a**) and blood pressure (**b**) during dialysis-induced hypotension in COVID-19 patients and non-COVID-19 septic patients as a control group, 2 h before and during dialysis induced hypotension. Linear regression (**c**) showing a non-phy

### FC-087 Characterization and outcomes of acute myocardial injury in COVID-19 intensive care patients

#### Vincent Labbe^1,6^, Stephane Ederhy^2,7^, Nathanael Lapidus^3^, Joe-Elie Salem^4^, Antoine Trinh-Duc^1^, Ariel Cohen^2,5,7^, Muriel Fartoukh^1,6^, Guillaume Voiriot^1,6^

##### ^1^Sorbonne Université, Assistance Publique-Hôpitaux de Paris (AP-HP), Hôpital Tenon, Service de Médecine Intensive Réanimation, Département Médico-Universitaire APPROCHES, Paris, France; ^2^Department of Cardiology, UNICO-GRECO cardio-oncology program, Hôpital Saint-Antoine, Assistance Publique-Hôpitaux de Paris (AP-HP), Paris, France; ^3^Sorbonne Université, INSERM, Institut Pierre Louis d’Epidémiologie et de Santé Publique IPLESP, Assistance Publique-Hôpitaux de Paris (AP-HP).Sorbonne Université, Public Health Department, Saint-Antoine Hospital, F75012, Paris, France; ^4^Assistance Publique-Hôpitaux de Paris (AP-HP), Pitié-Salpêtrière Hospital, Departments of Pharmacology and Cardiology, APHP.Sorbonne UNICO-GRECO Cardio-oncology Program, CIC-1901, INSERM, Sorbonne Université, F-75013, Paris, France; ^5^Sorbonne Université, UMR-S ICAN 1166, Paris, France; ^6^Groupe de Recherche Clinique CARMAS, Université Paris Est Créteil, Creteil, France; ^7^INSERM U 856, 75013, Paris, France

**Correspondence:** Vincent Labbe - vincent.labbe@aphp.fr

*Annals of Intensive Care* 2021, **11(Suppl 1):**FC-087

**Rationale:** The prevalence of myocardial injury (MI) in severe COVID-19 patients evaluated by systematic serial cardiac troponin assessments is unknown. In addition, information for comprehension of potential underlying mechanism leading to myocardial injury is still lacking. We aim to address the question of prevalence, characterization, and prognostic value of myocardial injury in severe COVID-19 patients.

**Patients and methods/materials and methods:** All consecutive patients with laboratory-confirmed SARS-CoV-2 infection admitted to our dedicated COVID-19 ICU between February 22 and April 31, 2020, were analysed. Cardiac investigations were systematically collected including daily dosage of High sensitivity cardiac troponin I (Hs-cTnI), and B-type natriuretic peptide, electrocardiography, and two-dimensional transthoracic echocardiography on ICU admission. The presence of MI was defined by the highest Hs-cTnI value above the 99th percentile upper reference limit and a change in values of ≥ 20% within the first 48 h. Studied outcomes were the overall mortality and the cardiovascular events (a composite of death, cardiac-arrest, cardiogenic shock and arterial thrombotic event) at day 28.

**Results:** Overall, 92 patients (78.3% men; age 62 [53–69] years) were analysed. MI occured in 53 patients (57.6%; 95% confidence interval[CI], 46.8%–67.9%) with a Hs-cTnI initial value of 112 (54–260) pg/ml. Among patient with MI, 13 (24.5%) presented ECG abnormalities including 1 (1.9%) ST segment elevation, 2 (3.8%) ST segment depression and 13 (24.5%) T wave inversion. Patients with MI had higher BNP levels (78 [20–188] vs. 20 [13–59], *p* < 0.001) and a lower left ventricular ejection fraction (55 [50–60] vs. 60 [55–60], *p* = 0.02) than patients without MI. Cardiovascular events occurred in 23 (25%) patients, including cardiac arrest (*n* = 1, 1.1%), cardiogenic shock (*n* = 4, 4.3%), arterial thrombotic event (*n* = 4, 4.3%), and death (*n* = 18; 19.6%). Figure 1 illustrates the association of mortality with MI (Kaplan–Meier survival curves, log-rank test *p* = 0.05). The OR for death and cardiovascular events in patients with versus without MI were 3.14 (95% CI 1.02–11.89) and 4.22 (95%CI 1.43–12.40), respectively. When adjusting on Sepsis-related Organ Failure Assessment, these associations were not significant. The magnitude of the Hs-cTnI initial values was associated with overall mortality (crude OR 2.42; 95%CI 1.25–4.94 per tenfold increase). Median daily Hs-cTnI values during the first week of ICU admission remained higher in non-survivors, as compared with survivors.

**Conclusion:** Acute myocardial injury is very frequent in critically ill COVID-19 patients, and is associated with severity

**Compliance with ethics regulations:** Yes in clinical research.
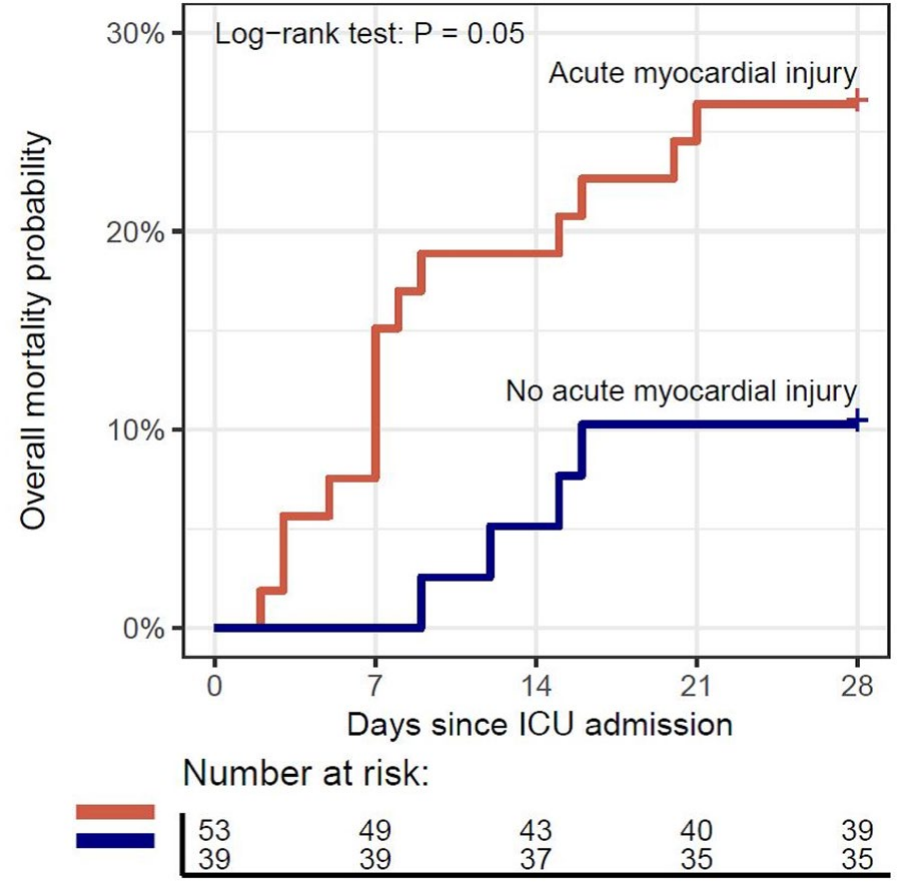


Kaplan–Meier curves for overall mortality at day 28 as a function of the presence of an acute myocardial injury (log-rank test: *p* = 0.05)

### FC-088 Sodium bicarbonate for out-of-hospital cardiac arrest: insights from French and North-American datasets

#### Maxime Touron^1^, François Javaudin^1^, Quentin Lebastard^1^, Valentine Baert^2^, Hervé Hubert^2^, Brice Leclere^1^, Jean Baptiste Lascarrou^1^

##### ^1^CHU Nantes, Nantes, France; ^2^Université de Lille, Lille, France

**Correspondence:** Maxime Touron - touronmaxime@yahoo.fr

*Annals of Intensive Care* 2021, **11(Suppl 1):**FC-088

**Rationale:** No large randomized controlled trial has assessed the potential benefits of sodium bicarbonate administration in patients with out-of-hospital cardiac arrest (OHCA). Current guidelines on this point are based on old and discordant data. Our objective here was to evaluate the potential benefits of sodium bicarbonate infusion during prolonged OHCA.

**Patients and methods/materials and methods:** We used two large datasets, one from France (RéAC Registry) and the other one from North America (ROC-CCC trial). We adjusted for potential confounders by performing a propensity score analysis with inverse probability-of-treatment weighting.

**Results:** Of the 54,807 patients in the French dataset, 1234 (2.2%) received sodium bicarbonate. After propensity score matching, sodium bicarbonate infusion was not associated with better neurological outcomes on day 30 (adjusted odds ratio [aOR], 0.912; 95% confidence interval [95%CI], 0.501–1.655). In the North-American dataset, of the 23,711 patients included, 4902 (20.6%) received SB. In the propensity-score matched analysis, SB infusion was associated with poorer neurological outcomes at hospital discharge (aOR, 0.45; 95%CI, 0.34–0.58).

**Conclusion:** In patients with OHCA, pre-hospital SB administration was not associated with neurological outcomes in the French dataset and was associated with worse neurological outcomes in the North-American dataset. The use of SB in patients with OHCA must be reserved, in accordance with the latest international guidelines, for patients with absolute indications such as hyperkaliemia and/or ventricular arrhythmias with QRS widening.

**Compliance with ethics regulations:** Yes in clinical research.

### FC-089 Extracorporeal cardiopulmonary resuscitation: effect of body position on carotid blood flow and intracranial pressure

#### Yael Levy^1,2^, Rocio Fernandez^2^, Fanny Lidouren^2^, Matthias Kohlhauer^2^, Lionel Lamhaut^2^, Alice Hutin^2^, Pierre-Louis Léger^1,2^, Guillaume Debaty^2^, Keith Lurie^2^, Bijan Ghaleh^2^, Renaud Tissier^2^

##### ^1^CHU Armand Trousseau, Paris, France; ^2^INSERM U955- PHYDES-IMRB Equipe 3- Stratégies pharmacologiques et thérapeutiques de l’ischémie myocardique et l’insuffisance coronaire- Ecole Vétérinaire de Maisons-Alfort (ENVA), Maisons-Alfort, France

**Correspondence:** Yael Levy - l.yael@hotmail.fr

*Annals of Intensive Care* 2021, **11(Suppl 1):**FC-089

**Rationale:** Extracorporeal cardiopulmonary resuscitation (E-CPR) could be proposed for refractory cardiac arrest. During conventional CPR, body position was shown to mitigate the cerebral alterations following resuscitation regarding carotid blood flow (CBF) and oxygenation. Here, we hypothesized that body position may also modify cerebral hemodynamics and metabolism during E-CPR. Our goal was to determine whether a whole-body tilt-up challenge (TU) could improve intracranial pressure (ICP), without deteriorating CBF during E-CPR in a swine model of cardiac arrest.

**Patients and methods/materials and methods:** Pigs were anesthetized and instrumented for the continuous evaluation of CBF, ICP and systemic hemodynamics. They were submitted to 15 min of untreated ventricular fibrillation, followed by 30 min of E-CPR. Electric attempts of defibrillation were then delivered until resumption of spontaneous circulation (ROSC). Extracorporeal circulation was continued after ROSC to target a mean arterial pressure above 60 mmHg. Animals were maintained in flat position (FP) throughout protocol, except during a TU challenge of the whole body (+ 30°) during 2 min at baseline, during E-CPR and after-ROSC.

**Results:** Four animals were submitted to the entire procedure and 3 of them elicited ROSC. After cardiac arrest, E-CPR was set to an average flow of 29 ± 2 ml/kg/min in order to obtain a mean blood pressure of 57 ± 8 mmHg in FP. Then, CBF achieved 28% of its baseline value and ICP remain stable (12 ± 1 vs 13 ± 1 mmHg during E-CPR vs baseline, respectively). Regarding TU effect, it significantly decreased ICP (− 63 ± 7%) in baseline conditions, as compared to FP, with a non-significant reduction in CBF (− 21 ± 3%) or systemic hemodynamics. During E-CPR and after ROSC, TU dramatically reduced ICP without any changes in CBF vs FP (Fig.).

**Conclusion:** Whole body TU reduce ICP without further deteriorating CBF during E-CPR. This deserves additional investigation with prolonged TU or head-up position during E-CPR.

**Compliance with ethics regulations:** Yes in animal testing.
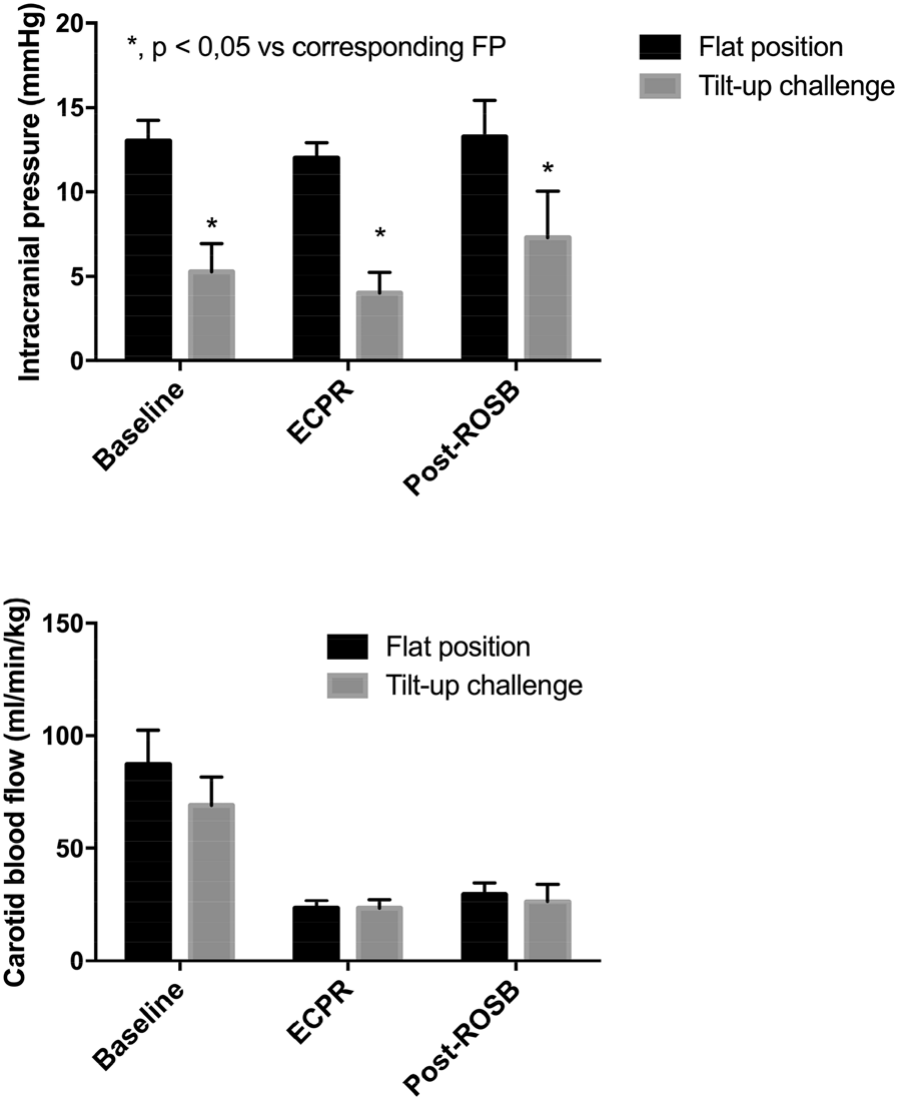


Intracranial pressure and carotid blood flow during baseline, E-CPR and ROSC

### FC-090 Predictors of death due to post-resuscitation shock after out-of hospital cardiac arrest: a multi-center study

#### Sebastian Voicu^1^, Wulfran Bougouin^3^, Georgios Sideris^1^, Emmanuel Gall^1^, Sofia Ortuno^3^, Marine Paul^2^, Nicolas Deye^1^, Isabelle Malissin^1^, Florence Dumas^2^, Eloi Marijon^3^, Xavier Jouven^3^, Bruno Megarbane^1^, Nadia Aissaoui^3^, Alain Cariou^2^

##### ^1^Lariboisière, APHP, Paris, France; ^2^Cochin, APHP, Paris, France; ^3^HEGP, APHP, Paris, France

**Correspondence:** Sebastian Voicu - sebastoso@yahoo.com

*Annals of Intensive Care* 2021, **11(Suppl 1):**FC-090

**Rationale:** Even after successful resuscitation, out-of-hospital cardiac arrest (OHCA) is a high-mortality condition due to death related to anoxic brain injury and/or post-resuscitation shock (PRS). Predictors of death due to PRS have been poorly investigated (1,2), despite direct therapeutic implications since PRS may be successfully managed with mechanical circulatory support. We performed a retrospective multi-center study in three French university hospitals, in order to determine predictors of death due to PRS.

**Patients and methods/materials and methods:** We included patients successfully resuscitated from non-traumatic OHCA between May 2011 and March 2020, and we assessed baseline and OHCA characteristics, laboratory, hemodynamic parameters on admission and mode of death. Death due to PRS was defined as death from persistent shock despite adequate fluid repletion and catecholamine treatment or due to recurrent cardiac arrest. We performed logistic univariable and multivariable analyses to determine factors associated with death due to PRS and validated the results in a replication cohort. Data are expressed as mean ± standard deviation or median (interquartile range IQR) as appropriate and numbers (percentages).

**Results:** 1002 patients were included, 822 in the derivation cohort, age 61 ± 16, 73% men, 54% with shockable initial rhythm. Total ischemic time from collapse to return of spontaneous circulation was 25 (15–35) min, left ventricular ejection fraction on admission (LVEF) was 40% (30–50). 516/822 (63%) of the patients died in-hospital, and among them 150/822 (18%) died of PRS. At the time of admission, predictors of death due to PRS were non-shockable initial rhythm, odds ratio (OR) 2.28, CI95 (1.34–3.87), *p* = 0.002, LVEF < 40%, OR 2.26, CI95 (1.26–4.07), *p* = 0.007, continuous vasopressor infusion OR 3.25, CI95 (1.53–6.89) *p* = 0.002, arterial lactate > 5 mmol/L, OR 4.33, CI95 (2.15–8.73) *p* < 0.0001 and arterial pH < 7.24, OR 0.47, CI95 (0.25–0.86) *p* = 0.01. In the replication cohort of 180 patients, the area under the curve of the model was 0.78 (IC95 0.67–0.89) and the Hosmer–Lemeshow goodness of fit test showed good performance of the model, *p* = 0.39.

**Conclusion:** On admission, 5 easy to identify risk factors (non-shockable initial rhythm, vasopressor treatment, LVEF < 40%, arterial pH < 7.24 and lactate concentration > 5 mmol/L) are independent predictors of death due to post-resuscitation shock after OHCA. These variables can be used to select patients who may benefit from mechanical circulatory support.

**References**

1. Voicu S, Baud FJ, Malissin I, Deye N, Bihry N, Vivien B, et al. Can mortality due to circulatory failure in comatose out-of-hospital cardiac arrest patients be predicted on admission? A study in a retrospective derivation cohort validated in a prospect.
2. Bascom KE, Dziodzio J, Vasaiwala S, Mooney M, Patel N, McPherson J, et al. Derivation and Validation of the CREST Model for Very Early Prediction of Circulatory Etiology Death in Patients Without ST-Segment-Elevation Myocardial Infarction After Cardiac.

**Compliance with ethics regulations:** Yes in clinical research.
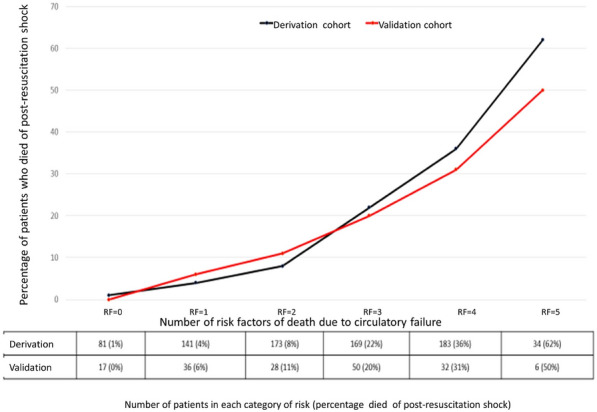


Probability of death due to circulatory failure according to the number of risk factors present on admission in the derivation and validation cohorts (RF-risk factors)

### FC-091 Incidence, clinical characteristics and outcome after unexpected cardiac arrest among critically ill adults with Covid-19: a prospective cohort study

#### Jonathan Chelly^1^, Gaetan Plantefeve^2^, Toufik Kamel^3^, Cedric Bruel^4^, Saad Nseir^5^, Christopher Lai^6^, Giulia Cirillo^7^, Elena Skripkina^8^, Sebastien Ehrminger^9^, Julio Badie^10^, Julien Le Marec^11^, Marine Paul^12^, Marie Labruyere^14^, Nicolas Pichon^15^, Romaric Larcher^16^, Mathieu Metzelard^17^, Nicolas Deye^13^, Laurent Ducros^1^

##### ^1^Centre Hospitalier Intercommunal Toulon La Seyne sur Mer, Toulon, France; ^2^Centre Hospitalier Victor Dupouy, Argenteuil, France; ^3^Centre Hospitalier Régional d’Orléans, Orléans, France; ^4^Groupe Hospitalier Paris Saint Joseph, Paris, France; ^5^Centre Hospitalier Universitaire Lille, Lille, France; ^6^Hôpital de Bicêtre, Le Kremlin-Bicêtre, France; ^7^Groupe Hospitalier Sud Ile de France, Melun, France; ^8^Hôpitaux Universitaires Henri Mondor, Créteil, France; ^9^Grand Hôpital de l’Est Francilien, Jossigny, France; ^10^Hôpital Nord Franche-Comté, Trevenans, France; ^11^AP-HP 6 Sorbonne Université, site Pitié-Salpêtrière, Paris, France; ^12^Centre Hospitalier de Versailles, Le Chesnay, France; ^13^Centre Hospitalier Universitaire Lariboisière, Paris, France; ^14^Hospitalier Universitaire François Mitterrand, Dijon, France; ^15^Centre Hospitalier Dubois, Brive La Gaillarde, France; ^16^Hôpital Lapeyronie, Montpellier, France; ^17^Centre Hospitalier Universitaire d’Amiens-Picardie, Amiens, France

**Correspondence:** Jonathan Chelly - jonathanchelly1@gmail.com

*Annals of Intensive Care* 2021, **11(Suppl 1):**FC-091

**Rationale:** Several reports have described the dramatical poor outcome of unexpected cardiac arrest in patients hospitalized in intensive care unit (ICU-CA) for Covid-19 pneumonia in China and USA. However, there are limited data on the characteristics and outcomes of ICU-CA in patients with Covid-19 in Europe.

**Patients and methods/materials and methods:** Multicenter prospective observational cohort in 35 French ICUs including all consecutive ICU patients admitted for Covid-19 pneumonia with occurrence of an ICU-CA with cardiopulmonary resuscitation (CPR) and sustained return of spontaneous circulation (ROSC). Favorable and unfavorable outcome were defined as modified Rankin scale from 0 to 3 and from 4 to 6, respectively.

**Results:** During the study period, 2425 patients were admitted in ICU for Covid-19 pneumonia of whom 186 (8%) ICU-CA occurred and 146/186 (78%) had CPR. Among the 117 ICU-CA patients with sustained ROSC, 74 (63%) died in the ICU, including 40 (34%) within the first day after ICU-CA occurrence and 18 (15%) from withdrawal of life sustained therapy. Forty-three patients (37%) survived at ICU discharge and 30 patients (26%) had favorable outcome at day 90 after ICU-CA. Most of ICU-CA occurred with non-shockable rhythms (89%). At the time of ICU-CA, SOFA score was 10 [7–14], 105 patients (90%) were under mechanical ventilation, 61 (52%) were under vasopressors and 56 (48%) suffered from almost 3 organ failures. After adjustment on confounding factors, the presence of 3 or more organ failures before ICU-CA occurrence was the only factor significantly associated with unfavorable outcome at day 90 after ICU-CA (OR 5.05; 95%CI [1.61–15.80] with *p* = 0.005).

**Conclusion:** Among this French cohort of patients admitted for Covid-19 pneumonia, ICU-CA incidence remained high. However, one in four patients with sustained ROSC was alive at 3 months with good neurological status which contrast with recent reports with dramatical poor outcome in such patients.

**Compliance with ethics regulations:** Yes in clinical research.

### FC-092 Health-related quality of life in critically ill survivors: specific impact of cardiac arrest in non-shockable rhythm. Insights from two randomized trials

#### Guillaume Geri^1,2,3,4^, Nadia Aissaoui^4,5,6^, Colin Gwenhael^4,7^, Alain Cariou^4,6,8,9^, Jean-Baptiste Lascarrou^4,9,10^

##### ^1^Hôpital Ambroise Paré, Boulogne Billancourt, France; ^2^Université Paris Saclay, Versailles, France; ^3^INSERM UMR 1018, Villejuif, France; ^4^AfterROSC Network, Paris, France; ^5^Hôpital Européen Georges Pompidou, Paris, France; ^6^Université de Paris, Paris, France; ^7^CHD Les Oudairies, La Roche Sur Yon, France; ^8^Hôpital Cochin, Paris, France; ^9^INSERM, U970, Paris Cardiovascular Research Center (PARCC), Team 4 Cardiovascular Epidemiology & Sudden Death, Paris, France; ^10^Hotel Dieu, Nantes, France

**Correspondence:** Guillaume Geri - guillaume.geri@aphp.fr

*Annals of Intensive Care* 2021, **11(Suppl 1):**FC-092

**Rationale:** Intensive care has a strong impact on health-related quality of life (HRQOL). The specific impact of cardiac arrest in non-shockable rhythm is poorly known.

**Patients and methods/materials and methods:** We gathered patients included in two randomized controlled trials (AWARE and HYPERION). We compared the 3-month HRQOL of these patients to those of a large sample of the French general population. Physical and mental dimension were compared. Multivariable linear regression was used to pick up factors associated with HRQOL.

**Results:** 72 and 307 patients of the HYPERION and the AWARE studies were compared to 20,574 French controls. Patients included in the HYPERION trial evidenced lower scores in all the SF-36 dimensions compared to those included in the AWARE trial and the controls except in the mental health dimension. The physical component score was lower in patients from the HYPERION trial compared to those from the AWARE trials and to controls (39.1 [28.6–45.9], 41.6 [35.0–48.2] vs. 53.9 [48.1–57.3], *p* < 0.001) (Fig.). After adjustment for age and gender, HYPERION and AWARE trial status were associated with lower physical but higher mental component score.

**Conclusion:** Health-related quality of life of unshockable cardiac arrest survivors evaluated at 3 months was similar to ICU survivors and significantly lower than in individuals from the general population, especially in the physical dimensions.

**Compliance with ethics regulations:** Yes in clinical research.
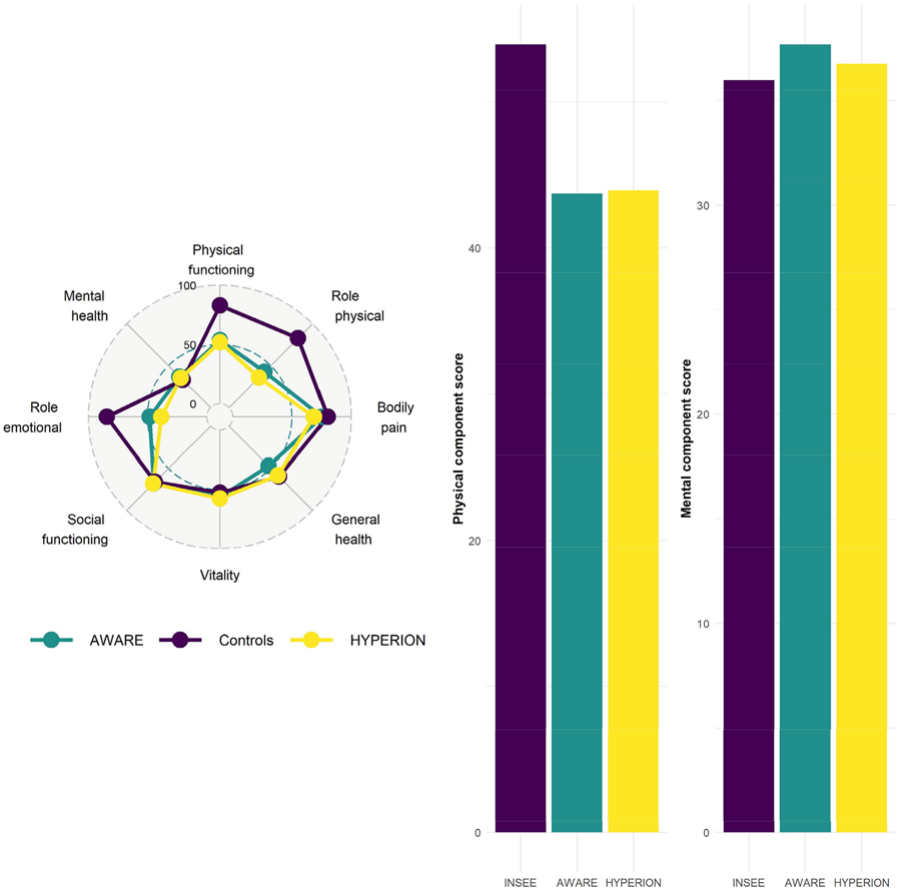


Fig. Health-related quality of life assessed by the SF-376 questionnaire of both patients included in the AWARE and HYPERION studies and French controls

### FC-093 Factors associated with anxiety and depression among healthcare workers in intensive care units during COVID-19 pandemic in Tunisia

#### Iyed Maatouk, Manel Lahmar, Rihab Boubtane, Abir Chihaoui, Oussama Saadaoui, Zeineb Hammouda, Saoussen Ben Abdallah, Fahmi Dachraoui, Fekri Abroug, Lamia Ouanes Besbes

##### Intensive Care Department, Teaching Hospital, Fattouma Bourguiba,University of Monastir, Monastir, Tunisia, Monastir, Tunisie

**Correspondence:** Iyed Maatouk - maatouk.yed@gmail.com

*Annals of Intensive Care* 2021, **11(Suppl 1):**FC-093

**Rationale:** Recent studies have shown that Covid-19 has a substantial impact on mental health of healthcare workers (Azoulay et al. AJRCCM 2020) [1]. We aimed at evaluating the prevalence of anxiety and depression symptoms among healthcare workers in intensive care units during the COVID-19 pandemic in Tunisia.

**Patients and methods/materials and methods:** We conducted a cross-sectional study using an online French language questionnaire created with Google Forms and submitted through social media. The participation was entirely voluntary. We used the 7-item Generalized Anxiety Disorder scale (GAD-7) and the 9-item Patient Health Questionnaire (PHQ-9) to evaluate, respectively, the level of symptoms of anxiety and depression.

**Results:** In total, 80 healthcare workers participated in the study. Among the participants, 53.8% were physicians and 46.3% were nurses. The majority of respondents were female (57.5%). The median age was 29 years (interquartile range: 27–30). Before the COVID-19 pandemic, only 16.3% reported having poor or fair mental health, while during the COVID-19 pandemic, poor or fair mental health was reported by most of respondents (71.3%). The mean GAD-7 score was 7.39 ± 4.4. Among the participants, 28.8% reported having symptoms of moderate-to-severe anxiety. Participants with moderate-to-severe levels of anxiety were more frequently nurses than physicians (65.6% vs 34.8%; *p* = 0.031), were living more frequently in urban areas than rural ones (78.3% vs 21.7%; *p* = 0.04), and belonged mores frequently to healthcare workers caring for COVID-19 patients (91.3% vs 8.7%; *p* = 0.012). The mean PHQ-9 score was 9 ± 5.3. Almost half of the respondents (45%) reported having symptoms of moderate-to-severe depression. No significant differences were disclosed between having moderate-to-severe level symptoms of depression and occupation (50% for both physicians and paramedical personnel; *p* = 0.54), place of residence (urban areas vs rural areas: 88.9% vs 11.1%; *p* = 1) and management of COVID-19 patients (those caring for COVID-19 patients vs those who did not: 75% vs 25%; *p* = 0.5).

**Conclusion:** COVID-19 pandemic can affect the mental health of healthcare workers especially those in intensive care units. Interventional studies should be conducted to show the impact of group therapy on the mental health of these healthcare professionals.

**Reference**

1. Azoulay E, Cariou A, Bruneel F, Demoule A, Kouatchet A, Reuter D, et al. Symptoms of Anxiety, Depression, and Peritraumatic Dissociation in Critical Care Clinicians Managing Patients with COVID-19. A Cross-Sectional Study. Am J Respir Crit Care Med. 31 ao.

**Compliance with ethics regulations:** N/A.

### FC-094 Description of mental health support services made available to caregivers in 40 French hospitals during the COVID-19 epidemic

#### Mélanie Loiseau^1^, Fiona Ecarnot^2,3^, Alexandra Laurent^4^, Irene François-Purssell^1^, Christine Binquet^1^, Jean-Pierre Quenot^1^

##### ^1^CHU Dijon, Dijon, France; ^2^CHU Besançon, Besançon, France; ^3^EA3920, University of Franche-Comté, Besançon, France; ^4^Laboratoire de Psychologie, University of Burgundy, Dijon, France

**Correspondence:** Jean-Pierre Quenot - jean-pierre.quenot@chu-dijon.fr

*Annals of Intensive Care* 2021, **11(Suppl 1):**FC-094

**Rationale:** The COVID-19 pandemic has far-reaching repercussions in terms of mental health, especially for intensive care unit workers, prompting the creation of innovative support services. Few, if any data are available describing mental health support services for caregivers (in terms of organisation, services provided, etc.). We sought to describe the mental health support services implemented during the pandemic, and to describe how they functioned and who their intended users were.

**Patients and methods/materials and methods:** Prospective study during the first national lockdown in France from 17 March to 18 May 2020. An online questionnaire was sent to the 77 hospitals participating in the PsyCovid study to identify and describe any mental health support services made available for caregivers. Centres were invited to participate in a follow-up phone call to provide further details.

**Results:** 40/77 centres returned the completed questionnaire (52% response rate). A mental health support service was available in 36/40 (90%) centres; 28 of these (78%) were created specifically for the COVID-19 epidemic. Median availability was 8 h per day (range 2–24 h/day). Almost all (92%) provided support services via a telephone call centre. The majority (34/36, 94%) were intended for caregivers, and 35/36 (97%) were staffed by psychologists, and/or psychiatrists (18/36, 50%). The professionals who manned these services mainly worked in the same hospital where the mental health support service was set up. Five centres participated in the follow-up phone call to provide further details, and all noted that the rate of use of their mental health support service was low.

**Conclusion:** Mental health support services in the form of telephone helplines were implemented in numerous hospitals in France during the first COVID wave. Most of these were new initiatives, and were staffed by psychologists and psychiatrists from within the hospital. These projects underline the awareness of the need to provide caregivers with support, but the low rate of use of these services raises the question of whether this form of support is the most appropriate way to address caregivers’ mental health needs.

**Compliance with ethics regulations:** Yes in clinical research.

### FC-095 PsyCovid mental health support: experiences of professionals working in call centres providing mental health support for caregivers during the COVID-19 pandemic

#### Mélanie Loiseau^1^, Fiona Ecarnot^2,3^, Alexandra Laurent^4^, Irene François-Purssell^1^, Christine Binquet^1^, Jean-Pierre Quenot^1^

##### ^1^CHU Dijon, Dijon, France; ^2^CHU Besançon, Besançon, France; ^3^EA3920, University of Franche-Comté, Besançon, France; ^4^Laboratoire de Psychologie, University of Burgundy, Dijon, France

**Correspondence:** Jean-Pierre Quenot - jean-pierre.quenot@chu-dijon.fr

*Annals of Intensive Care* 2021, **11(Suppl 1):**FC-095

**Rationale:** The PsyCovid study revealed the significant mental health impact of the COVID-19 pandemic among caregivers in intensive care in France. Awareness of the mental health repercussions of the pandemic prompted innovation in support services for care workers, e.g., mental health support services. We sought to describe the population of healthcare professionals (HCPs) providing mental health support via call centres for caregivers, to understand their experience of this innovative service.

**Patients and methods/materials and methods:** Prospective study in France involving HCPs working in a mental health call centre for caregivers during the first national lockdown (17 March–18 May 2020). An online questionnaire was distributed to 40 mental health support centres across France identified through a questionnaire sent to all 77 participating centers in the PsyCOVID study. The questionnaire recorded the HCPs’ perceptions about how comfortable they felt providing this service (ranked on a scale of 0 (very uncomfortable) to 10 (perfectly comfortable), any difficult situations they encountered, and what improvements they would suggest. Data were described using standard statistics and by thematic analysis of raw data.

**Results:** A total of 36 HCPs completed the questionnaire. Most were women (77.8%), aged 35–49 years (41.7%), and psychologists (69.4%). Less than one-third were physicians. More than half (58.3%) worked in the East of France. The majority of respondents felt comfortable providing mental health support (score of 8/10) and almost all said they would do it again if needed. Reported difficulties concerned the following aspects: (i) organisation of the services; (ii) certain specific clinical situations; (iii) managing their own emotions; (iv) recognition of their contribution to this activity; (v) training; (vi) decision-making. Many respondents indicated that additional training would be useful, both in terms of organisation and for professional competence. Suggested areas for improvement include: ( i) practical organisation; (ii) recognition of the support service; (iii) training of HCPs providing support; (iv) auditing performance of the service.

**Conclusion:** This study shows that HCPs providing mental support for care workers during the COVID-19 epidemic in France were comfortable with their role, but encountered a range of organisational, hierarchical and emotional difficulties. This extraordinary type of service disrupts the usual professional paradigm, and calls into question the role and recognition of each HCP.

**Compliance with ethics regulations:** Yes in clinical research.

### FC-096 Experience of ICU healthcare workers during the COVID-19 epidemic in France: the PsyCovid Qualitative Study

#### Fiona Ecarnot^1,2^, Nicolas Meunier-Beillard^3^, Aurélie Pourrez^3^, Sandrine Lombion^4^, Christine Binquet^3^, Jean-Pierre Quenot^3^

##### ^1^CHU Besancon, Besançon, France; ^2^EA3920, Université de Franche-Comté, Besançon, France; ^3^CHU Dijon, Dijon, France; ^4^SLC Expertise, Chamesey, France

**Correspondence:** Fiona Ecarnot - fiona.ecarnot@univ-fcomte.fr

*Annals of Intensive Care* 2021, **11(Suppl 1):**FC-096

**Rationale:** The repercussions of the COVID-19 pandemic on the personal and professional lives of intensive care unit (ICU) healthcare workers (HCWs) remain unclear.

**Patients and methods/materials and methods:** We performed a qualitative study using semi-structured interviews with nurses and nurses’ aides working in the ICU during the first wave of the epidemic in 5 French centers. Interviews were performed from 15 July to 15 September 2020. Interviews were transcribed in full and analysed using thematic analysis.

**Results:** A total of 18 interviews were performed. The main issues raised concerned several distinct domains: (1) personal and family life: HCWs encountered logistic difficulties in the home (schooling, minding children, housework, separation from families, burden of hygiene, fielding questions from relatives/children about the epidemic). On a personal level, they experienced physical and psychological repercussions, fatigue and burn-out. (2) In the workplace: practical organisation was challenging, with a high workload, and low availability of resources. HCWs found it hard to “switch off” from their work and get quality rest; the uncertainty and anxiety of the situation was ever present, and perpetuated by constant media attention. (3) At the level of their profession: HCWs reported alternately stigmatisation due to their profession, or gratitude and recognition by the public of their contribution (via gifts and public solidarity). They hoped that the media attention would raise the profile of ICU HCWs in the public opinion, and perhaps lead to increased recognition (financial and/or social/symbolic). Some of the behaviours they encountered during the epidemic, either on the part of others (the public, colleagues, hierarchy, patients’ families) or actions they were themselves obliged to carry out, may have been in opposition with the values they ascribe to the profession of ICU HCW. (4) At local and national level: the epidemic raised the awareness of the importance of short supply circuits, and strong interactions at local level. In parallel, their personal experience was played out against the background of the governmental measures to contain the epidemic, which constituted exterior factors to be reckoned with but that could not be influenced.

**Conclusion:** The COVID-19 epidemic had profound repercussions on HCWs in their personal and family life, in the workplace, and within their profession. It remains to be determined whether the effects are transitory, or whether any of these aspects may be durably changed.

**Compliance with ethics regulations:** Yes in clinical research.

### FC-097 Assessment of psychosocial risks among health workers in a Tunisian COVID-19 ICU: state of the art

#### Amira Jamoussi^1^, Nacef Ben Mrad^1^, Soumaya Ben Dhaou^1^, Omar Belhadj Mohamed^1^, Aymen Ouertani^1^, Houda Douss^1^, Samia Ayed^1^, Fatma Jarraya^1^, Emna Rachdi^1^, Hager Ghannouchi^2^, Houda Gharbi^1^, Jalila Ben Khelil^1^, Mohamed Besbes^1^

##### ^1^CHU Abderrahmen Mami, Tunis, Tunisie; ^2^Instance Nationale de l’Évaluation et de l’Accréditation en Santé-INEAS, Tunis, Tunisie

**Correspondence:** Amira Jamoussi - dr.amira.jamoussi@gmail.com

*Annals of Intensive Care* 2021, **11(Suppl 1):**FC-097

**Rationale:** During the actual COVID pandemic, the health sector is in a worldwide crisis. Due to the complexity of their tasks, ICU healthcare providers are especially affected by this situation, in several aspects of their lives. We aimed to assess psychosocial risks among members of a COVID-19 ICU team.

**Patients and methods/materials and methods:** This study was carried in the 18-beds’ COVID-19 ICU of Abderrahmen Mami hospital, within a team compounded of 20 doctors (6 seniors and 14 juniors), 42 nurses, 17 orderlies and 17 workers. A 41-question questionnaire (tool validated by the national institute for research and security entitled ‘Outil Faire le point des risques psychosociaux’) was used. Answers were given collectively by a representative sample of all trades. Questions with disagreement among participants were voted on. Final results of the questionnaire were automatically generated by the application and a level of risk (from low to high) was given according to the 8 themes of the questions.

**Results:** On the day of study (February 2021, the 13th), 15 members of the ICU team, in addition to the head of ICU, Quality Manager and Responsible of Authority Accreditation participated. The total duration of the session was 2 h and 30 min; mean time spent per question was 2’:17’’ [0’:10”–9’:30”]. Five questions were voted on after conflicted answers. After completion of the questionnaire, obtained risks assessments are listed in Table 1.

**Conclusion:** Psycho-affective burden in COVID-19 ICU is high. Through this assessment tool, we identified several psychosocial risk factors that can have an impact on the emotional and physical workload as well as on the quality of care. Several actions need to be taken according to the level of risk for each item, the results of which will be disclosed in future studies.

**Compliance with ethics regulations:** Yes in clinical research.
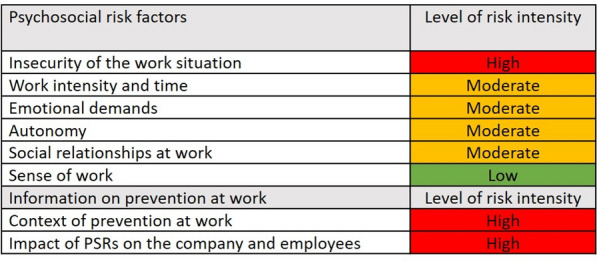


Psychosocial risk factors assessment

### FC-098 Factors associated with post-traumatic stress disorder among healthcare professionals in intensive care units during COVID-19 pandemic in Tunisia

#### Iyed Maatouk^1^, Amani Maatouk^2^, Haifa Safr^1^, Oussama Jaoued^1^, Habiba Ben Sik Ali^1^, Hassen Mohamed Fekih^1^, Souheil Elatrous^1^

##### ^1^Intensive Care Department, University Hospital Tahar Sfar of Mahdia, Tunisia, Mahdia, Tunisie; ^2^Department of community and preventive medicine, Tunisia, Monastir, Tunisie

**Correspondence:** Iyed Maatouk - maatouk.yed@gmail.com

*Annals of Intensive Care* 2021, **11(Suppl 1):**FC-098

**Rationale:** Intensive care professionals are among the workers the most involved in the management of COVID-19 patients and are therefore more likely to develop mental health disorders such as post-traumatic stress disorder (PTSD) after exposure to threatening events. Our study aimed to determine factors associated with PTSD symptoms among healthcare professionals in intensive care units during the COVID-19 pandemic in Tunisia.

**Patients and methods/materials and methods:** We carried out a cross-sectional study using an online French language questionnaire created with Google Forms and submitted through social media. The participation was voluntary. We used the Posttraumatic Stress Disorder Checklist for DSM 5 (PCL 5) to assess the presence and the severity of PTSD symptoms. This score ranges from 0 to 80. A linear regression analysis was used to evaluate the association between factors associated with PTSD. Data analysis was performed using the Statistical Package for Social Sciences (SPSS) version 21.0.

**Results:** Our study included 58 healthcare professionals. They were similarly distributed according to sex (55.2% males vs 44.8% females). The median age was 28 years (interquartile range: 26–30). Only 12.1% of the study population had psychological comorbidities. Among the participants, 17.2% were infected by coronavirus. The mean PCL 5 score was 33.05 ± 17.45. Among the respondents, 55.2% had symptoms of PTSD (*n* = 32). In univariate analysis, participants who were infected by coronavirus developed a significantly higher level of PTSD symptoms as compared to those who were infected by this virus (42.9 ± 15.9 versus 31 ± 17.2; *p* = 0.04). The level of PTSD symptoms was significantly higher among respondents aged more than 30 years (42 ± 12.2 years; *p* = 0.047). The mean PCL 5 score was 40.13 ± 16.25 among nurses and 30.58 ± 17.35 among doctors (*p* = 0.06). Linear regression analysis showed that being infected by coronavirus was an independent factor associated with PTSD (*p* = 0.04).

**Conclusion:** Intensive care professionals are experiencing high levels of PTSD symptoms during the COVID-19 pandemic. Therefore, useful coping strategies should be adopted to control this disorder.

**Compliance with ethics regulations:** N/A.

### FC-099 Does hypnosis have a place in the critical care unit? Health care professionals are open to be trained

#### Nicolas Bèle^1^, Andres Jurado^2^, Alain Besancon^1^, Riyad Farhat^1^, Vincent Grégoire^1^, Eric Harb^2^, Santiago Picos^2^, Christophe Roussel^1^, Michel Kaidomar^1^

##### ^1^Centre Hospitalier intercommunal de Fréjus-Saint Raphaël, Fréjus, France; ^2^Centre Hospitalier de la Dracénie, Draguignan, France

**Correspondence:** Nicolas Bèle - nicolasbele@free.fr

*Annals of Intensive Care* 2021, **11(Suppl 1):**FC-099

**Rationale:** Medical hypnosis is a tool increasingly used by health care teams as a complementary technique to the usual medical techniques. Numerous studies have shown beneficial effects in situations that cause stress or pain. Critical care units are high-risk areas of physical and psychological trauma for patients, relatives and health care workers. However, medical hypnosis appears to be little used despite multiple potential indications for patients, relatives or health care workers.

**Patients and methods/materials and methods:** The objective was to assess the knowledge of critical care providers of the general principles of medical hypnosis, the willingness of these caregivers to learn them and the potential obstacles to its implementation in their units. The study was carried out by the distribution of an anonymous questionnaire devised by 2 doctors both with a medical hypnosis university diploma in the 2 critical care units of the hospitals of Frejus-Saint Raphael and Draguignan (Fédération de Réanimation Var Est) with a total of 26 critical care beds.

**Results:** A total of 70 questionnaires were returned out of a potential 100 health care workers (70% response rate). The average age was 38.2 years (23–59). The professions of the responding health care workers were 43 nurses (61.4%), 20 health care support workers (28.6%), 6 doctors (8.5%) and 1 physiotherapist (1.4%). Forty-three caregivers (61.4%) had more than 5 years of critical care experience. Sixty-seven (95.7%) had already heard about medical hypnosis through discussions with colleagues (51.4%), articles (20.5%) or in other circumstances (28.1%). Fifty-one (72.8%) did not feel capable of giving a clear definition of medical hypnosis. Respectively, 16 (22.8%) and 49 (70%) did not completely or partially know the indications of medical hypnosis. The main situations proposed by caregivers regarding the use of medical hypnosis are related to the management of anxiety and invasive procedures. The numerical scale assessment of the value of using hypnosis in critical care was measured on average at 8 (CI; 3–10). The interest in training staff in hypnosis specific to critical care was measured on average at 8.9 (CI; 4–10). The constraints identified for the development of medical hypnosis are successively the low proportion of trained personnel (42.1%), the time available (19.7%) and skepticism about the usefulness of hypnosis (15.7%).

**Conclusion:** Health care professionals in critical care units appear to be largely in agreement with the potential value of medical hypnosis and appear to be motivated for training tailored to the practice of critical care.

**Compliance with ethics regulations:** Yes in clinical research.

### FC-100 Epidemiology of sepsis in Mali: a prospective multicenter study

#### Seydina Alioune Beye^1^, Hammadoun Dicko^1^, Boubacar Diallo^1^, Adriel Yekpogni Dihete^1^, Madame Thierno Diop^2^, Mahamedoun Coulibaly^3^, Abdoulaye Traoré^4^, Modibo Diakité^5^, Bagouma Traoré^4^, Mahamane Djibo Diango^2^, Youssouf Coulibaly^1^, Armand Mekentso Dessap^6^

##### ^1^Centre Hospitalier universitaire Point G, Bamako, Mali; ^2^Centre Hospitalier universitaire Gabriel Touré, Bamako, Mali; ^3^Centre Hospitalier universitaire “Le Luxembourg”, Bamako, Mali; ^4^Centre Hospitalier Nianankoro Fomba, Ségou, Mali; ^5^Centre hospitalier régional, Gao, Mali; ^6^Hôpitaux universitaires Henri Mondor, Créteil, France

**Correspondence:** Seydina Alioune Beye - beyealioune@gmail.com

*Annals of Intensive Care* 2021, **11(Suppl 1):**FC-100

**Rationale:** Sepsis is an organ dysfunction due to an inappropriate response of the body to an infection. This study aimed at describing the epidemiology of sepsis in Mali (excluding COVID-19).

**Patients and methods/materials and methods:** This was an observational, descriptive and prospective study over a 1-year period from May 2019 to April 2020 in five hospitals in Mali, before the outbreak of the COVID-19 pandemic. Data entry and analysis were done with Windows 10 and SPSS version 22.0 softwares.

**Results:** During the study period, 257 sepsis cases were recorded. The mean age was 42 ± 19 years. Male patients were predominant (65%). The main co-morbidities were high blood pressure (28%) and diabetes (23%). The low availability of biological data did not allow us to make a systematic evaluation of SAPS II or SOFA scores. Fifty (20%) patients presented with shock (defined as the need for catecholamine infusion). Overall hospital mortality rate was 42%; it increased when catecholamines (80%) or invasive mechanical ventilation was required (80% and 82%, respectively); conversely, the malarial etiology of sepsis and the performance of interventional treatment for source control were associated with lower mortality.

**Conclusion:** Sepsis is a major cause of mortality in Mali, particularly when circulatory or respiratory organ support is required. A special effort is needed to improve the availability and quality of organ support in limited resources countries

**Compliance with ethics regulations:** Yes in clinical research.

### FC-101 Epidemiological and bacteriological profile of post-operative pulmonary resection surgery pneumonia (PPO)

#### Fatma Guelmami, Maha Touaibia, Amira Dridi, Mohamed Sofien Mtir, Mehdi Abdennadher, Sonia Ouerghi, Taher Mestiri

##### Hôpital abderrahmen Mami Ariana, Tunisie, Manar Ii, Tunis, Tunisie

**Correspondence:** Fatma Guelmami - fatma.guelmami@gmail.com

*Annals of Intensive Care* 2021, **11(Suppl 1):**FC-101

**Rationale:** PPO in pulmonary resection surgery is a serious complication that can engage the prognosis. However, the bacterial epidemiology of PPO remains poorly known and variable according to the various series published in the literature.

**Patients and methods/materials and methods:** We conducted a retrospective study at the department of intensive care at the hospital Abderrahmen Mami Ariana from September 2017 to September 2019. We included patients aged more than 18 years, who underwent pneumonectomy, lobectomy or bi-lobectomy and who developed a PPO. The exclusion criteria were a negative bacteriological test, a non-significant rate according to validated methods or the necessary data set was not found in the medical records. Pneumonia is confirmed if a bacterium is identified according to a validated technique. We selected: sputum culture, protected distal sampling and broncho-alveolar washing. The statistical study was carried out by the SPSS23 software. A p0.05 is considered to be significant.

**Results:** We reviewed 150 files. 110 were excluded for missing data. A total of 40 files were analyzed. The average age of patients was 56.70 ± 11.66, a sex ratio of 2.1. Factors contributing to the acquisition of multi-resistant bacteria (MRB) in the univariate study were: invasive post-operative procedure, mechanical ventilation, and hospitalization time in an intensive care setting. They were significantly associated with the acquisition of MRB (*P*: 0.002). Radiological impairment was homolateral to the intervention in 53.8% of and contralateral in 46.2% of cases. The diagnosis of pneumonia was microbiologically documented on the sputum culture in 55.6% of cases, protected distal sampling in 37.8% of cases and mini broncho-alveolar washing in 6.6% of cases. Culture was poly-microbial in 64% 41, 2% of bacterial strains were MRB. Empirical antibiotic therapy was adequate in 40% of cases. 30.6% of patients had non-invasive ventilation and 16.7% were under invasive ventilation with an average hospitalization lengths of stay of 14 days. 11.9% of patients presented with ARDS. Overall mortality in ICU was 11.9% or 5 patients with MRB. The susceptibility of the bacteria to ceftazidime and imipenem is shown in Table 1

**Conclusion:** Our study showed that 41.2% of patients had multi-resistant bacteria. Given the limited number of new anti-infectives, the absence of effective antibiotics against multi-resistant bacteria, one of the urgent solutions to be applied remains a rationalization of the use of antibiotics and antifungals by taking example on the concept Antimicrobial Stewardship

**Compliance with ethics regulations:** N/A.
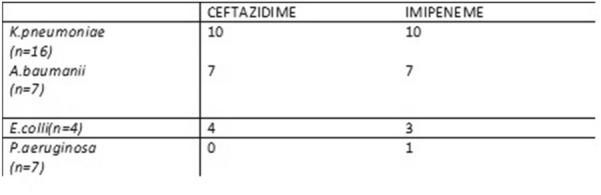


Table 1

### FC-102 Clinical and bacteriological particularities of nosocomial meningitis in intensive care: prospective study of 19 cases

#### Najeh Baccouch, Dorsaf Dlensi, Rezk Ghorbel, Rania Ammar, Mabrouk Bahloul, Mounir Bouaziz

##### faculty of medecine of sfax university of sfax, Sfax, Tunisie

**Correspondence:** Najeh Baccouch - baccouch.najeh@gmail.com

*Annals of Intensive Care* 2021, **11(Suppl 1):**FC-102

**Rationale:** Meningitis is said to be nosocomial if it occurs in or if it follows a procedure potentially contaminating. They pose problems in terms of diagnosis and treatment

**Patients and methods/materials and methods:** This is a prospective study, conducted in the department of emergencies and ICU carried out from January 2019 to December 2019. We included 19 patients

**Results:** The mean age was 45.58 ± 17.28 years with extremes ranging from 11 years to 68. Sex ratio was 1.11. Most of them were without comorbidity (11 patients 57.89%). 4 patients presented arterial hypertension (20.05%), and 5 patients presented diabetes (26.31%). The reason for admission to intensive care was: severe head trauma in 9 cases (47.4%), postoperative resuscitation of a brain tumor in 7 cases (36.8%), one case (5.3%) of neuromeningeal tuberculosis, one case (5.3%) of respiratory distress and one case (5.3%) of hemorrhagic stroke. The period of development of meningitis were 11.68 ± 10.62 days with extremes ranging from 1 day at 40 days. The mechanism of nosocomial meningitis was—by inoculation: excision of a brain tumor, placement of an external ventricular bypass, hematoma evacuation decompression flap in 13 cases (68.42%),—an osteodurmeric breach in two cases (10.52%),—sepsis in 3 cases (15.78%) and by contiguity in a single case. Nosocomial meningitis in our study was revealed by: convulsion, neurological degradation, agitation, delayed awakening, pus at the operative site. The bacteriological CSF sample was negative in 8 cases (42.1%) and positive in 11 patients. The germs found are: *Acinetobacter*, 5 cases or 26.31%; *Klebsiella pneumoniae* 5 cases or 26.31%; *Pseudomonas aeruginosa* 1 case or 5.2%. These isolated germs were multidrug resistant. The antibiotic therapy used was based on the combination intravenous of gentamicin + imipenem intravenous in 14 cases (73.6%), colistin + imipenem in 2 cases (10.5%) and colistin + tigecycline in 3 cases (15.7%).Colimycin was used intrathecally in 2 patients as well as gentamicin in 1 patient. The main neurological complications were cerebral abscess (20.52%) and hydrocephaly (42.1%), cerebral thrombophlebitis (10.52%), empyema (15.78%). The recurrence was observed in 3 cases. The outcome was favorable in only 6 cases. The cause of death was: septic shock in 57% of cases, neurological worsening of the initial lesions in 28% of cases and cardiogenic shock in 14% of cases.

**Conclusion:** Nosocomial meningitis and its management constitute a real challenge, both diagnostic and therapeutic. An osteodumeric breach, neurosurgical procedures are considered as risk factors for the occurrence of this complication.

**Compliance with ethics regulations:** Yes in clinical research

### FC-103 Severe acute respiratory infections: epidemiology, clinical outcomes and evolving features

#### Selma Feki, Lilya Debbiche, Emna Rachdi, Nacef Ben Mrad, Fatma Jarraya, Amira Jamoussi, Samia Ayed, Mohamed Besbes, Jalila Ben Khelil

##### Hôpital Abderrahman Mami Ariana, Tunis, Tunisie

**Correspondence:** Lilya Debbiche - debbichelilya@gmail.com

*Annals of Intensive Care* 2021, **11(Suppl 1):**FC-103

**Rationale:** Severe acute respiratory infections (SARI) are defined as infections associated with a fever and cough evolving for 10 days and requiring hospitalization. The aim of this study was to describe epidemiological, clinical and outcome of SARI.

**Patients and methods/materials and methods:** It was a descriptive, retrospective, longitudinal and monocenter study, including all hospitalized patients between January 1st, 2015 and December 31st, 2018, with SARI. We recorded demographic, clinical, and biological data and evolving features in all patients. A comparison between viral SARI and bacterial SARI was carried out.

**Results:** SARI had an incidence of 18.5%. They affected adults of all ages, male and having comorbidities (85%). Vaccination against influenza was very low (4.6%). On admission, patients had signs of respiratory severity in 351 cases, hemodynamics in 80 cases and neurological (Glasgow Coma Score < 8) in 50 cases. A bacterial origin was identified in 38.6% against 14.6% for the viral origin. The etiology was unknown in 41.6% of the cases and viral and bacterial co-infection was recorded in 19 cases. The use of mechanical ventilation and catecholamines was frequent (respectively, in 267 and 118 patients). Antiviral treatment was prescribed in 76 patients, 22 of whom had confirmed influenza. Antibiotics were administered to 278 patients (mostly combination of amoxicillin–clavulanic acid). At least one complication was noted in 153 patients. The most frequent was acute respiratory distress syndrome in 124 patients. Mean length of stay was 11.22 + − 12.20 days and mortality was 38.4%. Comparison of viral SARIs with bactrial SARIs showed that bacterial SARIs were significantly more frequent (143 vs 54; *p* < 0.05). Pregnant women and patients not vaccinated against influenza had more viral infection (7.4 vs 1.4%; *p* = 0.034 and 3.7 vs 3.5% *p* = 0.022). The SARIs of viral origin consulted later (delay = 8.04 ± 4.86 vs 6.5 ± 4.45 days; *p* = 0.042); received more antibiotics before admission (74 vs 47.6%; *p* < 0.001), were more frequently feverish (46.3 vs 25.2%; *p* = 0.004) and had significantly more frequent interstitial opacity in radiography.

**Conclusion:** SARI are frequent pathologies with important morbidity and mortality. A multicenter and large-scale study seems necessary to validate these findings.

**Compliance with ethics regulations:** N/A.

### FC-104 Empirical antibiotic therapy for early ventilator-induced pneumonia in severe brain injury

#### Antoine Premachandra^1^, Aurelien Mazeraud^1^, Caroline Schimpf^1^, Camille Legouy^1^, Matthieu Daniel^1^, Alain Sermet^1^, Xavier Sauvageon^1^, Alexis Amine Benmostefa^1^, Tarek Sharshar^1^, Michel Wolff^1^

##### ^1^GHU Sainte Anne, Paris, France

**Correspondence:** Antoine Premachandra - antoine.prema@gmail.com

*Annals of Intensive Care* 2021, **11(Suppl 1):**FC-104

**Rationale:** Pneumonia are common after severe brain injury. They are either inhalation or ventilator-associated pneumonia (VAP). The combination of amoxicillin and clavulanic acid (ACA) or a 3rd generation cephalosporin is recommended by the SRLF and SFAR (2017) for the probabilistic treatment of early VAP (< 5 days) without risk factors for multi-resistant bacteria. The aim of this study was to study microbial epidemiology and to evaluate the frequency of bacteria resistant to the amoxicillin–clavulanic acid combination during early VAP in severely brain injured patients.

**Patients and methods/materials and methods:** Retrospective study in a ten-bed neurosurgical intensive care unit between 01/08/2018 and 30/04/2020. VAPs were considered early if they occurred after 48 h and before the 6th day of mechanical ventilation (MV). VAPs were defined by the association of clinical and radiological signs with a positive culture of a protected distal sampling (> 10^3^ cfu/mL) or bronchoalveolar lavage (> 10^4^ cfu/mL).

**Results:** Six hundred and two patients were admitted, of which 198 (33%) with MV > 24 h. Their characteristics were as follows: median age 58 years [49;69], 65.2% male and mean (± SD) IGS2 of 48.5 ± 14.5. The main pathologies were: cerebral hemorrhage (23%) or meningitis (22%), head trauma (17%), malignant ischemia (11%). Eighty-four (42%) patients developed at least one VAP, including 56 early VAPs. A total of 116 bacteria were isolated from early VAPs (majority of which were plurimicrobial). No initial characteristics were able to differentiate patients with early VAP with or without Gram-negative bacteria (GNB), except for a higher IGS2 in the ones with GNB (53 vs 42, *p* = 0.02). Among the 56 early VAPs, 21 (37.5%) were associated with GNB insensitive to ACA. Patients with early VAP who had received antibiotic therapy (*n* = 28) before the onset of pneumonia (including intraoperative antibiotic prophylaxis) were more likely to have GNB insensitive to ACA (*p* = 0.02). No other variable were associated with pneumonia untreatable by ACA. Bacterial epidemiology of early VAP according to the antibiotic therapy statute is described in the table.

**Conclusion:** In severely brain injured patients admitted to our institution, the amoxicillin–clavulanic acid association is not appropriate in more than a third of early VAP. It should not be used as a probabilistic treatment, particularly in the case of pre-admission antibiotic therapy, including surgical antibiotic prophylaxis

**References**

1. Léone et al. RFE SFAR—SRLF Pneumonies associées aux soins de réanimation; 2017.
2. E. De Montmollin et al. Pneumonia in acute ischemic stroke patients requiring invasive ventilation. J Infect. 2019;79(3):220–7.PMID: 31238051

**Compliance with ethics regulations:** Yes in clinical research.
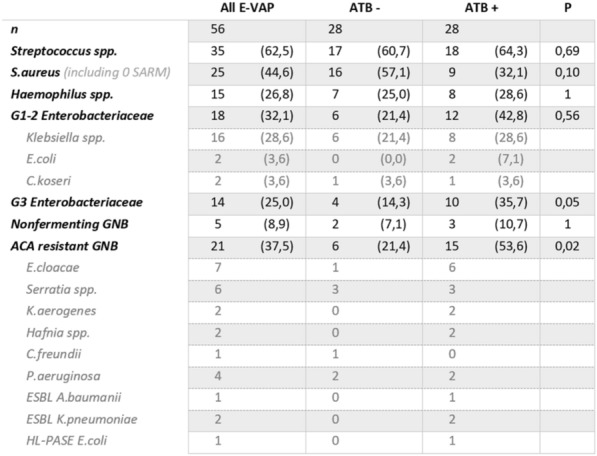


Bacterial epidemiology of early ventilator-acquired pneumonia (E-VAP) according of the previous antibiotic treatment (ATB+) or not (ATB−).

### FC-105 Usefulness of Sepsis-3 in diagnosing and predicting mortality of ventilator-associated lower respiratory tract infections

#### Alexandre Gaudet, Matthieu Devos, Sylvain Keignart, Olivier Pouly, Sylvain Lecailtel, Frederic Wallet, Saad Nseir

##### CHU de Lille, Lille, France

**Correspondence:** Alexandre Gaudet - alexandre.gaudet@chru-lille.fr

*Annals of Intensive Care* 2021, **11(Suppl 1):**FC-105

**Rationale:** Early distinguishing ventilator-associated tracheobronchitis (VAT) and ventilator-associated pneumonia (VAP) remains difficult in the daily practice. However, this question appears clinically relevant, as treatments of VAT and VAP currently differ (1). In this study, we assessed the accuracy of sepsis criteria according to the Sepsis-3 definition in the early distinction between VAT and VAP.

**Patients and methods/materials and methods:** Retrospective single-center cohort, including all consecutive patients with a diagnosis of VAT (*n* = 70) or VAP (*n* = 136), during a 2-year period. Accuracy of sepsis criteria according to Sepsis-3, total SOFA and respiratory SOFA, calculated at time of microbiological sampling were assessed in differentiating VAT from VAP, and in predicting mortality on ICU discharge.

**Results:** Sensitivity and specificity of sepsis criteria were found, respectively, at 0.4 and 0.91 to distinguish VAT from VAP, and at 0.38 and 0.75 for the prediction of mortality in VA-LRTI. A total SOFA ≥ 6 and a respiratory SOFA ≥ 3 were identified as the best cut-offs for these criteria in differentiating VAT from VAP, with sensitivity and specificity, respectively, found at 0.63 and 0.69 for total SOFA, and at 0.49 and 0.7 for respiratory SOFA. Additionally, for prediction of mortality, a total SOFA ≥ 7 and a respiratory SOFA = 4 were identified as the best cut-offs, respectively, yielding sensitivity and specificity at 0.56 and 0.61 for total SOFA, and at 0.22 and 0.95 for respiratory SOFA.

**Conclusion:** Sepsis criteria according to the Sepsis-3 definition show a high specificity but a low sensitivity for the diagnosis of VAP. Our results do not support the use of these criteria for the early diagnosis of VAP in patients with VA-LRTI.

**Reference**

1. Kalil AC, Metersky ML, Klompas M, Muscedere J, Sweeney DA, Palmer LB, et al. Management of adults with hospital-acquired and ventilator-associated pneumonia: 2016 clinical practice guidelines by the infectious Diseases Society of America and the American.

**Compliance with ethics regulations:** Yes in clinical research.

### FC-106 Empiric antibiotic therapy in severe SARS COV 2 infection

#### Feriel Ben Aba, Hamdi Hemden Doghri, Sajida Sboui, Badra Bahri, Ines Sedghiani, Nabiha Borsali Falfoul

##### Université de Tunis El Manar, Faculté de Médecine de Tunis. Hôpital Habib Thameur. Service des urgences et de réanimation médicale, Tunis, Tunisia

**Correspondence:** Feriel Ben Aba - benaba.feriel@gmail.com

*Annals of Intensive Care* 2021, **11(Suppl 1):**FC-106

**Rationale:** SARS COV 2 pandemic is distinguished by a raised antibiotic prescription. This is justified by bacterial co-infection and superinfection risk and antiviral and immunomodulator effect of macrolide antibiotics. Actually, this prescription is increasingly controversial. Our objective was to assess the impact of empiric antibiotic therapy in COVID 19 critically ill patients and to determine its supporting arguments.

**Patients and methods/materials and methods:** Descriptive, prospective and monocentric study including all patients admitted in an intensive care units (ICU) department between 09/07/2020 and 12/31/2020. Demographics, clinical, biological, radiological, bacteriological, therapeutical data and outcomes were collected.

**Results:** Among the 67 patients included and infected by the SARS-COV-2, sex ratio was 1.6 and median age was 64 year. Median SAPS II was 35. Severe acute respiratory distress syndrome (ARDS) was present in 31 patients, moderate ARDS in 32, and mild ARDS in 4. Fever was present in 14 patients (21%) at admission and mean value of C-reactive protein (CRP) was 162 mg/l. All patients received empiric antibiotics associated to a parenteral corticosteroid therapy, including a combination of antibiotics in 64 cases. The remaining three patients received a monotherapy with a macrolide before admission. Antibiotic therapy prescribed was a third-generation cephalosporin in 63 cases, a macrolide in 62 cases, ureidopenicillins in four cases, quinolones in two cases and metronidazole in one case. The mean duration of antibiotic therapy was 7.6 days and global mortality was 49% (*n* = 33). The empiric antibiotic prescription was justified by high procalcitonin value in 19 cases and by parenchymal infiltrates in chest CT-scan in 38 cases. No microbiological evidence of a community bacterial superinfection was found.

**Conclusion:** COVID-19 pandemic is associated to an over-consumption of antibiotics, especially in ICU. This systematic prescription is not justified by a bacterial superinfection rarely established. Measures of rationalization by more microbiological investigations are needed.

**Compliance with ethics regulations:** Yes in clinical research.

### FC-107 Incidence and impact prognosis of pulmonary embolism in COVID-19 infected patients

#### Sana Kharrat, Malek Hafdhi, Najeh Baccouch, Amal Triki, Mohamad Ali Hadj Mosbeh, Rania Ammar, Rezk Ghorbel, Ilef Alila, Mabrouk Bahloul, Mounir Bouaziz

##### CHU Habib Bourguiba, Sfax, Tunisia

**Correspondence:** Sana Kharrat - sanakharrat15@hotmail.com

*Annals of Intensive Care* 2021, **11(Suppl 1):**FC-107

**Rationale:** Coronavirus disease 2019 (COVID-19) remains an increasing global pandemic with significant morbidity and mortality. COVID-19 has been accurately described as the cause for a pro-inflammatory and hypercoagulable status which increases risk of pulmonary embolism (PE) in critically ill patients. The incidence of PE is reported to be up to one-third of those requiring intensive care unit (ICU) admission. In this study, we aimed to assess the prevalence of PE and its impact prognosis in our cohort.

**Patients and methods/materials and methods:** We conducted a retrospective study in an intensive care unit including critically ill patients with confirmed SARS-COV2 infection who underwent Computed Tomography Pulmonary Angiography (CT-PA) between September 2020 and January 2021.

**Results:** We evaluated 122 patients with COVID-19 admitted to our institution. The mean age was 62 ± 13 years and 95 patients (78%) were male. Among these patients, 57(47%) had hypertension, 52 (43%) had diabetes and 11(9%) had chronic lung disease. 43% of patients were obese (BMI > 30 kg/m^2^). Mean SAPSII score on ICU admission was 34 ± 16 and mean SOFA score was 5 ± 3. On admission, most of patients were haemodynamically stable (84%). The mean rate of troponin was 0.048 ng/ml. All inpatients received anticoagulation (enoxaparin 40 mg twice daily in ward patients, or enoxaparin 60 twice daily in obese according to local practice). They were referred for CT-PA at initial presentation or in patients who had presented clinical deterioration during hospitalization such as an increased need for oxygen. PE was diagnosed in 5 patients with an incidence of 4%. In patients with PE, emboli were located in segmental arteries in 60% of cases and in proximal arteries in 40% of cases. PE was mainly unilateral (80%). No statistical difference between PE-positive and PE-negative groups was noted regarding age, sex, co-morbidities and in outcomes including mortality, the need of mechanical ventilation and length of stay. Disease extent on CT-PA was not significantly different between patients with or without PE.

**Conclusion:** Our data suggest a low incidence of PE in our population when compared to the literature. However, this low incidence of PE may be attributed to the optimal prescription of therapeutic anticoagulation by front-line physicians in our country even for mild-to-moderate COVID-19 infection.

**Compliance with ethics regulations:** N/A.

### FC-108 Obesity in COVID-19 infected patients: incidence and impact outcome

#### Ilef Alila, Sana Kharrat, Rania Ammar, Farah Zouari, Kamilia Chtara, Asma Koubaa, Saba Makni, Najeh Baccouche, Mabrouk Bahloul, Mounir Bouaziz

##### CHU Habib Bourguiba, Sfax, Tunisia

**Correspondence:** Sana Kharrat - sanakharrat15@hotmail.com

*Annals of Intensive Care* 2021, **11(Suppl 1):**FC-108

**Rationale:** Coronavirus disease 2019 (COVID-19) remains an increasing global pandemic, with significant morbidity and mortality. Obesity is common in patients with (COVID-19). However, the association of obesity with the severity of this disease remains unclear. We aimed to assess the effects of obesity on clinical outcomes of COVID-19 including requirement of mechanical ventilation (MV) and mortality.

**Patients and methods/materials and methods:** We conducted a retrospective monocenter study including critically ill patients with confirmed SARS-COV2 infection between September 2020 and January 2021. Underweight was defined as a body mass index (BMI) < 18.5 kg/m^2^, normal weight as 18.5–24.9 kg/m^2^, overweight as 25.0–29.9 kg/m^2^, and obesity as ≥ 30 kg/m^2^.

**Results:** A total of 155 patients were admitted during the study period. The mean age was 62 ± 13 years, and 118 (76%) of patients were male. Among these patients, 73 (47%) had hypertension, 68 (44%) had diabetes and 14 (10%) had chronic lung disease, 42% of patients were obese. Mean SAPSII score on ICU admission was 34 ± 16 and mean SOFA score was 5 ± 3. Among the included patients, 32% were normal weight, 2% were underweight, 24% were overweight and 42% were obese. Obesity was significantly associated with higher cardiovascular comorbidities (*p* < 0.001) including hypertension (*p* = 0.004). Moreover, women had a significantly higher prevalence of obesity than men (65% versus 35%; *p* = 0.002). Obese patients seem to have more digestive symptoms (*p* = 0.05) and neurological symptoms (*p* = 0.006) as initial presentation of the disease, compared with non-obese patients. Patients with obesity more frequently needed mechanical ventilation (76% vs. 60%;  *p* = 0.04;OR = 2.11 IC [1.1–4.3]). No statistical difference between obese and non-obese patients was noted regarding mortality and length of stay.

**Conclusion:** Obesity is a well-recognised cause of respiratory function compromise which might make this group of patients at risk of poor outcomes in COVID-19, with a higher probability of mechanical ventilation.

**Compliance with ethics regulations:** N/A.

### FC-109 Organ dysfunctions in COVID-19 patients requiring oxygen in Mali: a multicenter study

#### Seydina Alioune Beye^1^, Boubacar Diallo^1^, Nouhoun Diani^2^, Amadou Sidibé^2^, Hammadoun Dicko^1^, Mamadou Karim Touré^3^, Charles Dara^4^, Abdoulaye Mamadou Traoré^3^, Abdoulaye Traoré^5^, Moussa Kanté^6^, Abdoulaye Traoré^7^, Modibo Diakité^8^, Youssouf Coulibaly^1^, Armand Mekentso Dessap^9^

##### ^1^Centre Hospitalier universitaire Point G, Bamako, Mali; ^2^Centre Hospitalier universitaire Hôpital du Mali, Bamako, Mali; ^3^Centre Hospitalier universitaire Dermatologique, Bamako, Mali; ^4^Centre hospitalier régional, Tombouctou, Mali; ^5^centre hospitalier Nianankoro Fomba, Ségou, Mali; ^6^Centre Hospitalier régional, Sikasso, Mali; ^7^Centre hospitalier Somino Dolo, Mopti, Mali; ^8^centre Hospitalier, Gao, Mali; ^9^Hôpitaux universitaire Henri Mondor, Créteil, France

**Correspondence:** Seydina Alioune Beye - beyealioune@gmail.com

*Annals of Intensive Care* 2021, **11(Suppl 1):**FC-109

**Rationale:** Coronavirus 2019 disease (COVID-19) is a respiratory tract infection caused by severe acute respiratory syndrome coronavirus-2 (SARS-COV-2). Approximately, 14% of patients develop a severe form that requires hospitalization for oxygen treatment. This study aimed to determine the prevalence and factors associated with organ dysfunction in patients with COVID-19 in Mali.

**Patients and methods/materials and methods:** This was an observational multicenter study, with retrospective data collection. Patients were 18 years of age or older, hospitalized for suspected or confirmed infection with COVID-19. The SAPS II or SOFA scores could not be systematically evaluated due to the incomplete availability of biological data. The Quick-SOFA (Sepsis 3 definition) was used to evaluate respiratory, circulatory and neurological dysfunctions.

**Results:** Among the 2646 patients admitted for COVID-19 infection, 220 (8%) needed oxygen treatment and were included in the study. The mean age was 58 ± 16 years (range from 18 to 92 years). There were more men (68%) than women. The main co-morbidities were high blood pressure (32%) and diabetes (17%). The QuickSOFA at entry was positive in the majority of patients for respiratory function (75%), in a minority for neurological function (18%), and exceptionally for circulatory function (2%). During hospital stay, only a minority of patients received organ support, either non-invasive respiratory support (12%), invasive respiratory support (8%) or circulatory support (5% received catecholamine infusion). Vital status was collected in 207 patients, with an overall hospital mortality rate of 25%. This mortality was significantly higher in patients with neurological dysfunction at admission (63%) and in those who required intubation (72%) or catecholamines (80%) during the hospital stay.

**Conclusion:** Respiratory and neurological dysfunctions predominate in COVID-19 patients requiring oxygen treatment in this multicenter study in sub-Saharan Africa. These dysfunctions are associated with a significantly higher mortality in this context. Vital organ support should probably be considered early on admission to improve the prognosis of these patients.

**Compliance with ethics regulations:** Yes in clinical research.

### FC-110 SARS-CoV2 surfaces and air contamination in ICU: comparison of HFNC vs MV

#### Vincent Greux, Anne-Laure Lebreil, Odile Bajolet, Antoine Huguenin, Laurent Andreoletti, Bruno Mourvillier

##### CHU Reims, Hopital Robert Debré, Reims, France

**Correspondence:** Bruno Mourvillier - bmourvillier@chu-reims.fr

*Annals of Intensive Care* 2021, **11(Suppl 1):**FC-110

**Rationale:** Since the end of 2019, the human coronavirus SARS-CoV-2 (severe acute respiratory syndrome coronavirus 2) has been responsible for a worldwide pandemic and France on May the 16th counted nearly 100,000 hospitalizations and 28,000 deaths. Its inexorable advance across different countries had raised the question of its spread. At this time, some former studies about SARS-CoV-1 and a recent experimental study (1) highlight the viability for hours of SARS-CoV-2 in aerosols and on various surfaces that could expose healthcare workers to inhaled droplets from surfaces and aerosols particles in enclosed spaces such as Intensive Care Unit’s room. Medical expert groups have warned caregivers against the risk of aerosolization procedures as orotracheal intubation, tracheal suction and the use of high-flow nasal canula (HFNC). Some countries recommend N95 masks at all time when taking care of COVID-19 patients. The aim of this study was to compare the spreading by aerosolization of SARS-CoV-2 in ICU rooms between patients under HFNC and under controlled mechanical ventilation.

**Patients and methods/materials and methods:** From April 2020 to May 2020, 7 samples were taken from 20 patients’ individual rooms in Reims medical ICU, 10 from rooms with patients treated with HFNC and 10 from rooms with patients under controlled mechanical ventilation. These rooms were regulated by a negative pressure system and we sampled rooms with patients tested positive for SARS-CoV2 within the last 72 h. The last cleansing of the patient’s room had to be done at least 15 h before the sampling and the oxygenation support had to be the same. Samples, taken from each room, included: 2 air samples, one close (< 50 cm) and far (> 130 cm) from the patient’s head, and 5 surface samples at previously defined places: headboard wall, foot barrier, nursing bench, contralateral wall, and ventilator screen. Surface stability was evaluated on ICU surfaces. Inocula were measured using quantitative-PCR. Three replicate experiments were performed for each sample. Samples were sent directly to the virology laboratory. Moreover, we also performed viral culturing of each sample. Results are shown as graphics with copies per cm^2^ of surface and copies per liter of air sampled. We used the nonparametric Mann–Whitney U test to compare the differences between these samples.

**Conclusion:** Viral load in the air and on various surfaces was similar between ICU rooms, whatever the ventilatory support used (CMV or HFNC). None of the samples were positive for viral culture.

**Compliance with ethics regulations:** N/A.
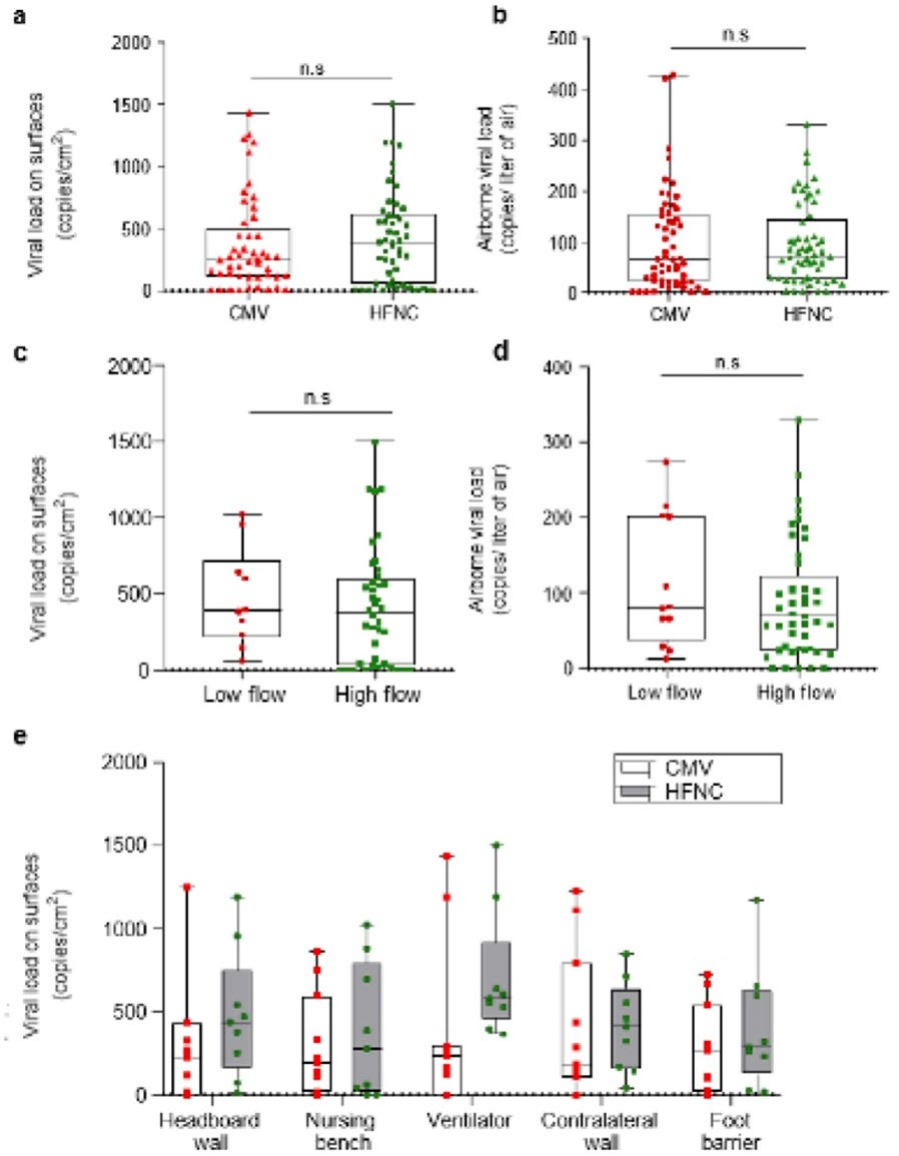


Comparison between CMV and HFNC ventilation in the air and into various surfaces

### FC-111 Risk factors of mortality in critically ill patients affected with COVID-19

#### Ahlem Trifi^1^, Bedis Tlili^1^, Mohamed Abessi^2^, Asma Ouhibi^1^, Dorra Nouri^2^, Nabil Bouguezzi^1^, Bedis Jeribi^2^, Chokri Omri^2^, Foued Daly^1^, Cyrine Abdennebi^1^, Sami Abdellatif^1^, Adel Ammous^2^, Salah Ben Lakhal^1^

##### ^1^Medical ICU, la Rabta hospital, Faculty of Medicine of Tunis, Tunis, Tunisie; ^2^Surgical ICU, la Rabta hospital, Faculty of Medicine of Tunis, Tunis, Tunisia

**Correspondence:** Ahlem Trifi - trifiahlem2@gmail.com

*Annals of Intensive Care* 2021, **11(Suppl 1):**FC-111

**Rationale:** From the abundant literature on Covid-19, the common data emerged is the high rate of mortality among Covid-19 patients that required ICU admission. Prior studies have identified older age, metabolic co-morbidities and obesity such as risk factors for mortality. Our objective was to assess the relationship between a priori-defined factors of interest and mortality in COVID-19 patients admitted in ICU.

**Patients and methods/materials and methods:** A retrospective analytical cohort study of critical Covid-19 patients. The primary outcome was in ICU-mortality and a priori-defined characteristics of interest were the following: sex, obesity, clinical signs, co-morbidities, time-to-ICU admission from signs onset, laboratory findings, acute respiratory distress syndrome (ARDS) and other complications. A multivariable model was used to determine the independents factors.

**Results:** Of 143 patients, the median age was 64 years [57–71] and 102 (71%) were men and 78 (55%) died. From the results of the univariate analysis, the following factors were introduced to the multivariable model (entered method): diabetes, BMI, CURB-65, fever, respiratory rate (RR), heart rate (HR), arthromyalgia, mottling, pH, PaO_2_/FiO_2_ ratio at admission, WBC, Lymphocytes, Neutrophiles, CRP, D-dimer, prothrombin time (PT), troponin at admission, CPK, LDH, AST, ALT, CT-chest extension > 50%, ARDS, septic shock, acute kidney injury (AKI) and mechanical ventilation (MV). Independent factors of mortality were the following: HR > 90b/min [OR = 6.15 (*p* = 0.013)], lymphocytes count < 700 el/mm^3^ [OR = 5.26 (*p* = 0.022)], CRP > 150 mg/l [OR = 4.36 (*p* = 0.037)], D-dimer > 1300 µg/l [OR = 9.9 (*p* = 0.002)], septic shock [OR = 12 (*p* = 0.001)], AKI [OR = 12.5 (*p* < 10-3)} and MV [OR = 9.19 (*p* = 0.002)].

**Conclusion:** While our mortality rate was considerable, as commonly reported, we did not find the mostly cited risk factors (i.e. older age, obesity and metabolic co-morbidities), it was rather the emphasis of inflammatory reaction and coagulation dysfunction. The secondary complications (such as septic shock and AKI) and the use of MV were the factors independently associated to the death of critical patients hospitalized for COVID-19.

**Compliance with ethics regulations:** Yes in clinical research.

### FC-112 Role of chest CT in the diagnosis and prognostication of COVID-19 in intensive care units

#### Feriel Ben Aba, Hamdi Hemdene Doghri, Emna Abid, Amal Oussaifi, Imen Zaghdoudi, Chiraz Chammakhi, Nebiha Borsali-Falfoul

^1^Faculté de médecine de Tunis. Hôpital Habib ThameurUniversité de Tunis El Manar, Faculté de Médecine de Tunis. Hôpital Habib Thameur. Service des urgences et de réanimation médicale, Tunis, Tunisia.

##### **Correspondence:** Hamdi Hemdene Doghri - doghri.hamdi87@gmail.com

*Annals of Intensive Care* 2021, **11(Suppl 1):**FC-112

**Rationale:** In the COVID-19 pandemic, the RT-PCR tests are not available in all hospitals and their results need several hours. Therefore, it was crucial to find a performing and rapid diagnosis tool of SARS-CoV 2 infection. Chest computed tomography (CT) scan revealed a typical aspect of the COVID-19 and had a crucial contribution in this pandemic. The aim of our study was to describe the role of the chest CT in diagnosis and prognosis in critically ill COVID-19 patients.

**Patients and methods/materials and methods:** Descriptive and prospective study including patients admitted in an ICU department between 09/07/2020 and 12/31/2020. Demographics, clinical, biological, radiographic data and outcomes were collected.

**Results:** During the study period, 67 patients infected by the SARS-CoV 2 were included with a sex ratio of 1.6, median age of 64 years, and median IGSII of 35 points. The RT-PCR (reverse transcriptase-polymerase chain reaction) performed in 61 patients detected 49 positive cases (80%). Antigenic rapid test was performed in 26 patients and was positive in 8 cases (30%). At admission, 66 patients had a chest CT-scan including 44 a CT pulmonary angiography. Time period between symptoms onset and the imaging had a median value of 7 days. The diagnosis of COVD-19 pneumonia was established on chest CT-scan in 25 patients, with a pattern of ground-glass in all cases. Lesions distribution was peripheral in 24 cases, central and peripheral in 42 cases. Quantification of lung lesions was moderate, important, severe, and critical in, respectively, 6, 17, 20, and 23 patients. Other patterns were reported in 51 cases (76%) including consolidation (37 cases), emphysema (8 cases), reticulations (4 cases), bronchiectasis (3 cases) and fibrosis in one case. The chest CT revealed complications in 15 cases (13 pulmonary embolism and 2 pulmonary barotrauma). PaO_2_/FiO_2_ ratio < 150 was identified as an independent risk factor predictive of a severe to critical pulmonary damage (superior to 50%) on the chest CT (OR: 4.77; 95% CI: 1.55–14.66; *p* = 0.006). A critical pulmonary damage (superior to 75%) on the imaging was associated to a higher mortality (*p* = 0.047).

**Conclusion:** The chest CT was identified as a swift, accessible, and reliable diagnostic modality. It allowed an alternative tool to the RT-PCR for Covid-19 diagnosis and helped in assessing prognosis by quantifying lung lesions and revealing complications in critical ill patients.

**Compliance with ethics regulations:** Yes in clinical research.

### FC-113 Have we improved the management of COVID-19 patients admitted in intensive care between the two waves?

#### Lauréline De Visscher, David Fagnoul, Eric Carlier, Patrick Biston, Karim Zouaoui Boudjeltia, Michael Piagnerelli

##### CHU-Charleroi Marie Curie, Charleroi, Belgium

**Correspondence:** Lauréline De Visscher - laureline.de.visscher@ulb.be

*Annals of Intensive Care* 2021, **11(Suppl 1):**FC-113

**Rationale:** As with other countries in Europe, we experienced two distinct waves (from March to August and September to December 2020) of patients with COVID-19 admitted in our 40-bed ICU. During the first wave, we adapt our local practices in the management of these patients, by applying the results of the major studies, sometimes endorsed by the WHO [1]. We treat hypoxic patients with dexamethasone in place of hydroxychloroquine, despite few data on the severity of the patients enrolled. We also increased dose of thromboprophylaxis due to higher risk of thrombotic events [2], we specially applied ultra-protective mechanical ventilation and have reserved vvECMO only in selected patients [3]. We, therefore, aimed to evaluate the impact of our adaptive practice on ICU mortality between both waves.

**Patients and methods/materials and methods:** We collected and compared demographics, biological and pulmonary data of all adult patients admitted in our ICU for hypoxia due to COVID-19.

**Results:** Higher number of patients was admitted during the second wave (40 versus 134 patients). ICU mortality was similar in the second compared to the first wave for all patients (39 versus 30%; *p* = 0.3). Approximatively 70% of the patients required mechanical ventilation in both waves. In these more severe patients, more co-infections were diagnosed at ICU admission but no differences on demographic, duration of symptomatology before ICU admission, times for ICU admission and intubation were observed between patients (Table 1). Mortality in patients on mechanical ventilation was higher during the second wave (48 versus 36%; *p* = 0.28). In a multiple logistic regression, no variables were associated with mortality in ventilated patients during the first wave. In contrast, age (odds ratio: 1.08 [1.03–1.13]; *p* = 0.001); lactate concentrations at ICU admission (odds ratio: 1.64 [1.09–2.47]; *p* = 0.01); thromboembolic complications (odds ratio: 4.04 [1.41–11.5]; *p* = 0.009) and use of vasopressors (odds ratio: 5.53 [1.98–15.4]; *p* = 0.001) were associated with ICU mortality in the second wave. Despite modifications of treatments, thromboembolic complications and length of mechanical ventilation were the same between both waves (Table 1).

**Conclusion:** In summary, while management and therapies performed on ICU patients with COVID-19 were modified, ICU morbidities and mortality did not change. This teaches us that the results obtained on large cohorts of patients are difficult to translate into current practice. We need rapidly multicentric comparisons between the two waves to adapt our management.

**References**

1. Tsang JLY et al. Intens Care Med. 2021. 1–5.
2. Schmidt M et al. Lancet Respir Med. 2020; 8 (11): 1121–31.

**Compliance with ethics regulations:** Yes in clinical research.
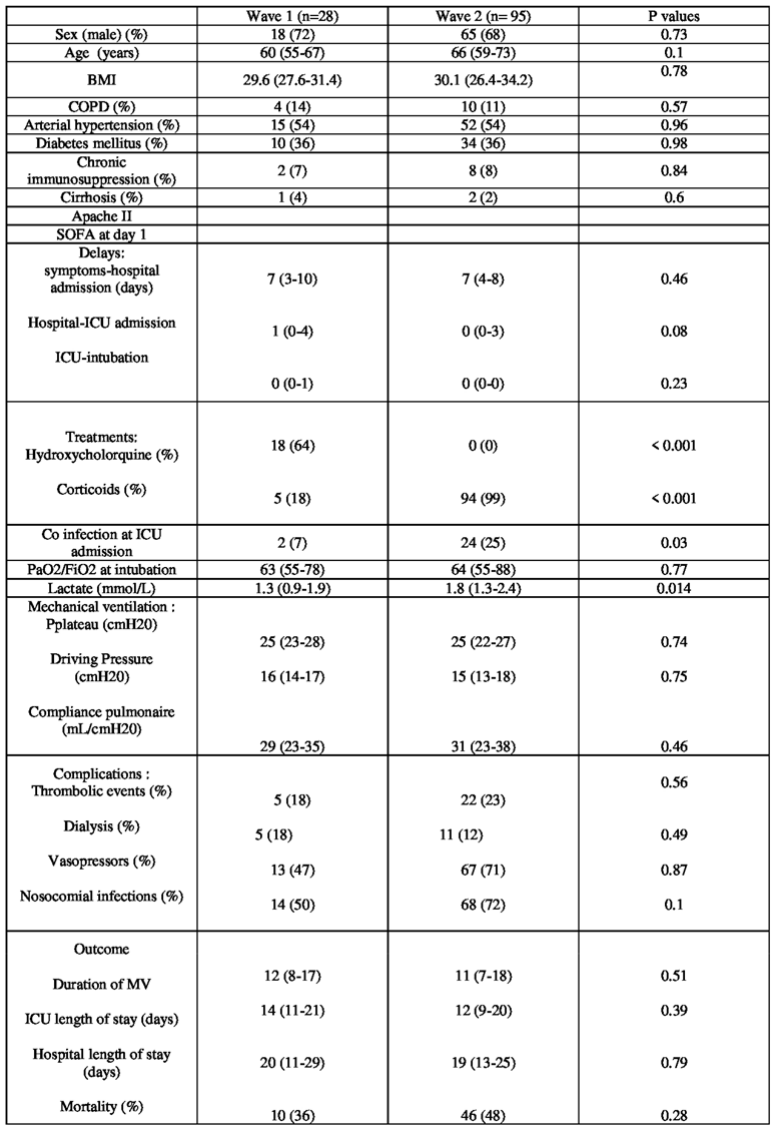


Characteristics of the ICU hypoxic patients admitted on mechanical ventilation

### FC-114 Acute community-acquired pneumonia due to COVID-19 in Moroccan medical ICU: predictive and prognostic factors

#### Samah El Mhadi, Tarek Dendane, Abbas Haroun, Khalid Abidi, Amine Ali Zeggwagh

##### Hopital universitaire Ibn Sina, Rabat, Morocco

**Correspondence:** Tarek Dendane - tdendane@hotmail.com

*Annals of Intensive Care* 2021, **11(Suppl 1):**FC-114

**Rationale:** The 2019 coronavirus disease (COVID-19) outbreak, caused by infection with severe acute respiratory syndrome coronavirus 2 (SARS-COV2) emerged in China and quickly spread throughout the whole world. We were interested in all the acute community pneumonia admitted during this period of the COVID 19 pandemic and which requires admission to intensive care. The aims of this study were to describe the epidemiological characteristics, to identify the predictive factors as well as the prognostic factors of COVID 19 pneumonia.

**Patients and methods/materials and methods:** This is a prospective cohort study carried out in the Medical ICU at the Ibn Sina University Hospital in Rabat, from March to August 2020. All adults patients admitted for suspected or confirmed SARS-COV2 community-acquired pneumonia, requiring hospitalization in intensive care and having undergone a PCR test for SARS-COV2 were included in the study. Factors associated with mortality in confirmed SARS-COV2 infection were determined.

**Results:** Of the 100 patients admitted to ICU during the study period 40 patients had positive SARS-CoV-2 (mean age of 65 ± 13 years, 63 males, 37 females). The mean Fine score was 112 ± 36. Five % of patients had septic shock at admission. Invasive mechanical ventilation was needed in 53% of the cases. The CORADS score was 5 for 90% of patients. The mortality of patients with COVID-19 pneumonia was 33.5%. Obesity (OR = 4.6, 95% CI 1.46–14.8), hyperglycemia (OR = 2.4, 95% CI 1.1–5.7), peripheral damage to the CT scan (OR = 8.4, 95% CI 1.6–45), the use of prone position (OR = 4.2, 95% CI 1.3–13), and use of NIV (OR = 5.6, 95% CI 2.2–14) were independently associated with mortality.

**Conclusion:** COVID-19-related acute community-acquired pneumonia mortality was high. This mortality appears to be associated with obesity, hyperglycemia on admission, peripheral damage to the CT scan, use of prone position and non-invasive ventilation.

**Compliance with ethics regulations:** Yes in clinical research.

### FC-115 Fatality rate and risk factors of mortality in severe COVID-19-infected patients

#### Ilef Alila, Sana Kharrat, Kamilia Chtara, Rania Ammar, Farah Zouari, Najeh Baccouch, Olfa Turki, Hedi Chelly, Mabrouk Bahloul, Mounir Bouaziz

##### CHU Habib Bourguiba, Sfax, Tunisia

**Correspondence:** Sana Kharrat - sanakharrat15@hotmail.com

*Annals of Intensive Care* 2021, **11(Suppl 1):**FC-115

**Rationale:** The world is witnessing a global pandemic of coronavirus disease 2019 (COVID-19), which is considered to be related to infection by severe acute respiratory syndrome coronavirus 2 (SARS-CoV-2). Much interest has been devoted to report data on clinical characteristics and prognostic factors. However, to the best of our knowledge, few analyses were published from our country. In this study, we aimed to assess characteristics and potential predictors of hospital mortality of COVID-19 patients.

**Patients and methods/materials and methods:** We conducted a retrospective study including critically ill patients with confirmed SARS-COV2 infection in an intensive care unit between September 2020 and January 2021. Clinical characteristics, laboratory findings, treatment and outcomes were collected. The prognostic effects of variables were analyzed using logistic regression model.

**Results:** A total of 155 patients were admitted during the study period. The mean age was 62 ± 13 years, and 118 (76%) of patients were male. Among these patients, 73 (47%) had hypertension, 68 (44%) had diabetes and 14 (10%) had chronic lung disease. 42% of patients suffered from obesity. Mean SAPSII score on ICU admission was 34 ± 16 and mean SOFA score was 5 ± 3. The median PaO_2_/FiO_2_ ratio was at 105 mm Hg IQR [66–120]. Oxygen support was required for all patients, in particular, 63 (41%) needed invasive mechanical ventilation. The chest computed tomography performed in 118 patients (76%), showed 50% or greater of lung involvement in 88 patients (57%), 79 patients (51%) died. Multivariate logistic regression analysis showed that severity at admission (SAPSII > 35) (OR: 4.9 [1.7–14.5] and the use of mechanical ventilation (invasive OR: 40[11–143] or non-invasive OR: 8.5 [2.6–27.5]) were independent predictors of mortality in patients with severe COVID-19.

**Conclusion:** The COVID-19 outbreak determined high in-hospital mortality in severe patients, the main clinical predictors of which were severity at admission and the need of mechanical ventilation.

**Compliance with ethics regulations:** N/A.

### FC-116 Do we need a specific score to predict mortality in Covid-19?

#### Syrine Maatouk, Sourour Belhajyoussef, Manel Lahmar, Zeineb Hammouda, Safa Fathallah, Saoussen Benabdallah, Fahmi Dachraoui, Fekri Abroug, Lamia Ouanes-Besbes

##### CHU F.Bourguiba, Monastir, Tunisia

**Correspondence:** Fekri Abroug - fekri.abroug@gmail.com

*Annals of Intensive Care* 2021, **11(Suppl 1):**FC-116

**Rationale:** In addition to standard ICU scores, several specific prognostic scores have been developed to evaluate the risk of death in patients with confirmed SARS-CoV-2 infection requiring ICU admission. Determining which patients are at high risk of mortality is essential for appropriate clinical decision-making. In this study, we aimed to evaluate the prognostic prediction value of severity scores to help predict mortality.

**Patients and methods/materials and methods:** This is a prospective study performed during a 5-month period (from September 2020 to January 2021) in a 16 bed-ICU of a teaching Hospital. All patients with ARDS secondary to COVID-19 confirmed with RT-PCR, and aged 18 years and more, expected to stay more than 24 h in the ICU were enrolled in this study. They were evaluated according to their baseline characteristics, clinical, radiological and biological variables on ICU admission, as well as the therapeutic characteristics and the evolution during hospitalization. Three scores were calculated to identify high-risk patients for in-hospital mortality: SAPSII, SOFA, and a new COVID-19 severity score [1]﻿, ranging from 0 to 10, based on age, oxygen saturation, mean arterial pressure, blood urea nitrogen, C-reactive protein, and the international normalized ratio was calculated on admission. The performance of these scores in mortality predication was assessed by calculating the area under the receiver operating characteristic curves (AUC).

**Results:** During the study-period 109 patients fulfilled the inclusion criteria and were included in the study. The median age was 64 (IQR: 55–70) years, and 72% were male with a BMI of 28 (27–31). The most frequent comorbidity was hypertension (51%). For the therapeutic approach, the majority (83%) was assisted with high-flow nasal oxygen on admission, and 41 (37%) required invasive ventilation during their ICU stay. The median SAPS II and SOFA score were 29 (24–34.5) and 4 (3–5), respectively. Mortality occurred in 37 patients (34%). The ROC curve is displayed in Fig. 1. Among all three scores, SAPS II and SOFA score had the highest discrimination ((AUC 0.902; 95% CI 0.83–0.97) and (AUC 0.902; 95% CI 0.82–0.98)), respectively, whereas the novel COVID-19 severity score had an AUC = 0.84; 95% CI 0.76–0.93).

**Conclusion:** SAPSII and SOFA have a higher discrimination power than a specifically developed score in the prediction of Covid-19 mortality.

**Reference**

1. Altschul DJ.A novel severity score to predict inpatient mortality in COVID-19 patients. Nature (Scientific Reports 2020).

**Compliance with ethics regulations:** Yes in clinical research.
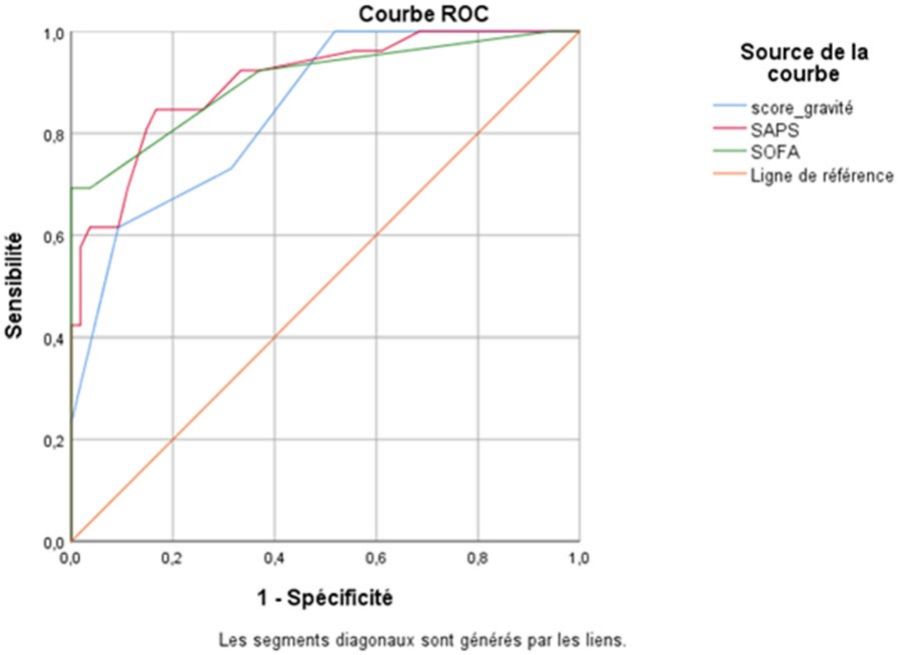


ROCurves and AUC of the three scores for prediction of ICU mortality

### FC-117 Decline in mortality of critically ill patients with COVID-19 during the second wave: the factors behind this improvement

#### Fanny Garnier, Philippe Corne, Racim Benomar, Matthieu Amalric, Noémie Besnard, Vincent Brunot, Delphine Daubin, Liliane Landreau, Sonia Machado, Valérie Moulaire, Corinne Pelle, Laura Platon, Boris Jung, Kada Klouche

##### CHU Lapeyronie, Montpellier, France

**Correspondence:** Fanny Garnier - garnier.fanny26@gmail.com

*Annals of Intensive Care* 2021, **11(Suppl 1):**FC-117

**Rationale:** After the first wave coronavirus disease 2019 (COVID-19) pandemic that lasted in May 2020, we faced a second wave from the end of August to December 2020. The progress made worldwide in both understanding and treating the disease could lead to improved prognosis. Indeed, we observed a significant decrease in mortality during the second wave. Our aim in this study was therefore to identify factors associated with this improvement including early use of dexamethasone and curative anticoagulation.

**Patients and methods/materials and methods:** We retrospectively collected clinical and biological features of all consecutive patients with a SARS-CoV-2 infection confirmed by RT-PCR who were admitted to our 20-bed ICU. Characteristics and outcome of patients admitted during the first (March 10, 2020 to May 15, 2020) and the second wave (August 16, 2020 to December 15, 2020) were compared. Univariate analysis and multivariate regression were then performed to identify factors associated with outcome.

**Results:** Among 183 critically ill COVID-19 patients, 51 patients were admitted during the first wave and 132 during the second wave. In-ICU and in-hospital mortality significantly decreased during the second wave, 25.5% vs 10%, *p* = 0.01 and 33% vs 12%, *p* < 0.01, respectively. Age, sex, and SAPSII score did not differ significantly between the 2 groups. Patients admitted during the second wave were less likely to require invasive mechanical ventilation (46, 90% vs 87, 66%; *p* < 0.01). However, median SOFA score at admission was significantly lower in second wave patients (7 vs 5, *p* < 0.01), and they underwent less renal replacement therapy (31% vs 7%, *p* < 0.01). A higher proportion of patients was treated by early glucocorticoid therapy (20% vs 98%, *p* < 0.01), and by curative anticoagulation (80% vs 100%, *p* < 0.01) during the second wave, but less were treated by antiviral treatment (82% vs 9%, *p* < 0.01). Multivariate analysis showed that only age (odds ratio (OR), 1.19; 95% confidence interval (CI), 1.08–1.32; *p* < 0.01), and SOFA score (OR, 1.38; 95% CI, 1.08–1.78; *p* = 0.01) were independently associated with ICU mortality. By contrast, early glucocorticoid therapy, antiviral therapy, curative anticoagulation in ICU were not significantly associated with a better outcome.

**Conclusion:** During the second wave COVID-19 pandemic, we observed a significant decrease in ICU-mortality critically ill patients. Factors associated with this improvement were age and admission SOFA score. We failed, however, to demonstrate any effect of an early corticoid use.

**Compliance with ethics regulations:** Yes in clinical research.

### FC-118 Blood pressure in ischemic vascular accidents. What modality of care at EHU Oran?

#### Setti Aouicha Zelmat, Djamila Bouabida, Fatema Mazour

##### Faculté de medecine, Oran, Algeria

**Correspondence:** Setti Aouicha Zelmat - Settiaouichazelmat@yahoo.fr

*Annals of Intensive Care* 2021, **11(Suppl 1):**FC-118

**Rationale:** The objective of the early management of these patients is to preserve a maximum of brain cells and not to aggravate a hemorrhagic process. Currently, the management of blood pressure in patients with ischemic stroke is consensual while the attitude is much less clear in the context of hemorrhagic stroke. We aimed to assess the control of the blood pressure of patients eligible for intravenous thrombolysis.

**Patients and methods/materials and methods:** Prospective study over a period of 12 months (April 2019 to February 2020). All patients with acute localized neurological deficit presenting to the emergency department eligible for intravenous thrombolysis were included. The data collection was based on an evaluation of the NIHSS score (National Institutes of Health Stroke Score), biological assessment, a brain scanner. The admitted patient was monitored (PNI, FC, FR, SCOPE). The parameters thus studied were epidemiological, clinical in particular the blood pressure profile, paraclinical, therapeutic and progressive controlling hypertension by administering a calcium channel blocker (nicardipine) to achieve blood pressure targets of 180 mmHg/100 mmHg or less. During episodes of hypotension, treatment with vasopressor or volume expansion was initiated cautiously with a blood pressure target of PAS > 150 mmHg.

**Results:** Ninety-three ischemic stroke patients eligible for IV thrombolysis, only 20 patients (21.5%) benefited from IV thrombolysis. We note a slight male predominance 62%, with an average age of 55 years (21–82 years), 6/20 patients required the administration of an antihypertensive treatment to control hypertension and only one case required a vasopressor treatment to control low blood pressure. We noted two thrombolysis failures, no hemorrhagic transformation, and no deaths.

**Conclusion:** A poorly managed hypertension treatment is very harmful because it decreases the cerebral perfusion therefore the perfusion of the penumbra zone, leading to an extension of the ischemic process. Relative hypertension in the early phase of ischemic stroke is beneficial especially when the patient has carotid stenosis. Apart from specific clinical situations, antihypertensive treatment should not be undertaken. One of the objectives of the initiation of antihypertensive treatment is the absolute necessity of varying the blood pressure figures slowly and gradually. All jolts are very harmful.

**Compliance with ethics regulations:** Yes in clinical research.

### FC-119 Malignant middle cerebral artery infarction: comparison between surgical and conservative treatment

#### Rania Ammar, Saba Makni, Najeh Baccouch, Sana Kharrat, Chokri Ben Hamida, Mabrouk Bahloul, Mounir Bouaziz

##### faculty of medecine of Sfax,university of Sfax, Sfax, Tunisia

**Correspondence:** Rania Ammar - rania.ammarzayani@gmail.com

*Annals of Intensive Care* 2021, **11(Suppl 1):**FC-119

**Rationale:** Malignant middle cerebral artery infarction is a devastating condition, with up to 80% mortality in conservatively treated patients. Treatment options include general measures to limit the extent of space-occupying edema, but these therapies have not been efficient. Surgical interventions with decompressive hemicraniectomy (DHC) have proven the efficacy with a significant decrease in mortality but with severe disability. To compare effect of chirurgical treatment versus conservative treatment on mortality and functional outcome.

**Patients and methods/materials and methods:** A retrospective study including patient with malignant middle cerebral artery infarction over 10 years (1st January 2010 to 31 December 2020).

**Results:** We included 55 patients. The mean (SD) age was 60 ± 14 years (31–90). The range age between 50 and 60 represented 30.9%. Mean GCS (SD) was 8.4 ± , 3.2. SAPS II ≥ 30 was found in 45 patients (82%). SOFA > 5 was found in 45 patients (82%). Patients in the DHC + group were younger than patients in the DHC- group with a mean age of 57 years ± 12 vs 64 years ± 15 with no statistically significant difference (*p* = 0.057). Patients in the DHC + group received more mannitol (*p* = 0.001). Patients who underwent DHC had more pulmonary embolism (*p* = 0.012) and delayed awakening (*p* = 0.002). Mortality was higher in non-operated patients 64% vs 56%. Operated patient had poor prognosis mRs (4–5) than non-operated 44% vs 29%.

**Conclusion:** Decompressive hemicraniectomy in malignant middle cerebral artery infarction may reduce mortality, but with more poor functional outcome.

**Compliance with ethics regulations:** N/A.

### FC-120 Ability of a quantitative analysis of EEG reactivity to predict awakening of severe brain-vascular injured patients

#### Eléonore Bouchereau, Guillaume Turc, Angela Marchi, Martine Gavaret^1^, Tarek Sharshar

##### GHU Paris Psychiatrie et Neurosciences, Paris, France

**Correspondence:** Eléonore Bouchereau - eleonorebouchereau@gmail.com

*Annals of Intensive Care* 2021, **11(Suppl 1):**FC-120

**Rationale:** Predicting awakening in severe brain-injured patients is challenging and usually relies on neurophysiological tests, especially Mismatch Negativity (MMN) with Event-Related Evoked Potentials (ERPs) and EEG reactivity (EEG-R) to auditory and painful stimulation. However, visual assessment of EEG-R is liable to inter-rater variability, prompting us to develop predictive models taking into account connectivity analysis with two quantitative (qEEG) measures of EEG-R, namely spectral analysis (FFT: Fast Fourier Transform) and phase synchronization analysis (PLI: Phase Lag Index) following auditory and nociceptive stimulations. Our aim is to assess whether FFT and PLI better predicts awakening within 28 days after non-traumatic brain injury than visual EEG-R and MMN.

**Patients and methods/materials and methods:** We prospectively enrolled comatose patients admitted in neuro-intensive care (monocentric study) for acute brain-vascular injury (poor grade subarachnoid hemorrhage, malignant cerebral infarction and intra-parenchymal hematomas) and who required invasive mechanical ventilation for more than 48 h. Neurophysiological tests included ERPs and EEG to assess MMN and reactivity to auditory (clapping, patients’ own name and first name) and nociceptive stimulations (nail bed pressure), respectively. Visual assessment of EEG-R was performed by two experienced neurophysiologists. The predictive models took into account the best variations of qEEG across channels and frequencies, expressed as relative differences (RD) before and after stimulations. The ability of each RD to discriminate reactive and non-reactive EEGs was assessed using the area under the receiver operating characteristic curves (AUC-ROC). Subsequently, an automated stepwise approach was used to select qEEG features for inclusion in a multivariable logistic regression model, with awakening at day 28 as the dependent variable.

**Results:** We have currently analyzed 59 out of 100 enrolled patients. Neurophysiological tests were performed at a median of 3 (interquartile range, IQR: 3;5) days after brain injury. Median (IQR) GCS and RASS were 3 (3;4) and − 5 (− 5; − 2), respectively. 38 (74.5%) patients had EEG-R, 25 (49%) had positive MMN and 37 (77%) awoke before 28 days. The ability of the final model to predict awakening within 28 days after brain injury was significantly better than that of the EEG-R visual analysis (AUC 0.93 versus 0.55, *p* = 0.007) but not significantly higher than that of the MMN (AUC 0.93 versus 0.73, *p* = 0.26).

**Conclusion:** Our preliminary results suggest that assessment of quantitative EEG connectivity with spatial and frequency resolution improves prediction of awakening in comatose brain-vascular injured patients at 28 days.

**Compliance with ethics regulations:** Yes in clinical research.

### FC-121 Prognosis of subarachnoid aneurysmal hemorrhage requiring transatlantic air transport

#### Frédéric Martino^1^, Antoine Fleuri^1^, Laure Flurin^1^, Michel Piotin^2^, Nicolas Engrand^2^, Michel Carles^1^

##### ^1^CHU DE LA GUADELOUPE, Les Abymes, Guadeloupe; ^2^FONDATION OPHTALMOLOGIQUE ADOLPHE DE ROTHSCHILD, Paris, France

**Correspondence:** Frédéric Martino - frederic.martino@chu-guadeloupe.fr

*Annals of Intensive Care* 2021, **11(Suppl 1):**FC-121

**Rationale:** Subarachnoid aneurysmal hemorrhage (SAH) is responsible for significant morbidity and mortality. Because no interventional neuroradiology platform is available in the West Indies, all patients with SAH requiring a neuroradiological intervention are transported by transatlantic flight to a reference center in France (RCF). We aimed to evaluate the prognosis of SAH patients transported across the Atlantic for neuroradiological intervention.

**Patients and methods/materials and methods:** We conducted a retrospective observational study carried out at our University Hospital from January 2010 to December 2019. The primary endpoint of the study was the 1-year survival of patients with SHA sent by transatlantic flight to the reference center. Secondary outcomes were mean of Glasgow Outcome Scale (GOS) and modified Rankin score at the exit of RCF, as well as time to transfer.

**Results:** A total of 151 patients were included. The mean age was 54 years, and 60% were female. The average Glasgow score at admission was 13. The most common symptom was headache (87%). Almost 47% of patients were intubated at hospital admission. The mean World Federation of Neurosurgical Societies score was 2. The mean FISHER Score was 3.2. Sixty-five patients benefited from the placement of a temporary Cerebral Spinal Fluid drainage. Among the 151 patients analyzed, 45 patients (30%) died at 1 year follow-up. In multivariate analysis, several independent criteria at admission were associated with the risk of death at 1 year: the motor deficit, a Fisher score equals to 4 on the initial CT scanner, intracranious hypertension or Glasgow score < 7 before take-off. In this study, the mean GOS score was 3.6 and the mean Rankin score was 2.5 at the follow-up. Median transfer time to RCF is 1 day after hospital admission.

**Conclusion:** Compared to literature data on SAH, patients requiring an early transatlantic transportation demonstrated no impairment of their 1-year clinical prognosis. We identified four prognostic criteria able to help clinicians in the decision to send the patient to the remote neuroradiological center (Fig. 1). These results deserve a comparative study between patients with initial air transportation and those treated without air transportation.

**Compliance with ethics regulations:** Yes in clinical research.
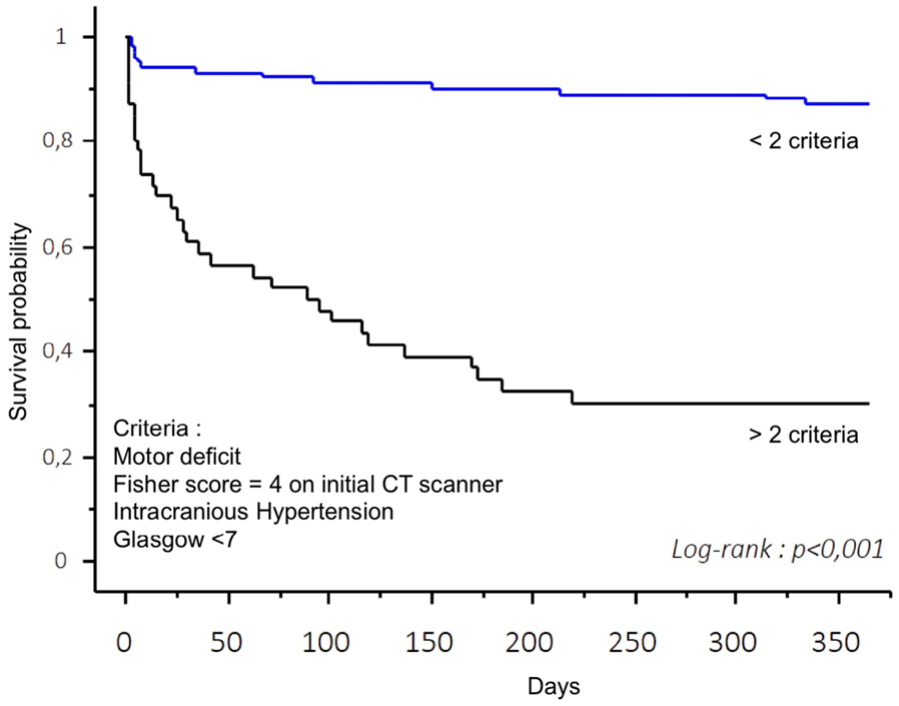


Survival Curve after SAH

### FC-122 Intravenous milrinone for treatment of delayed cerebral ischemia associated with subarachnoid hemorrhage: friend or foe?

#### Selim Aboulethar, David Cortier, Arturo Consoli, Catherine Horodyckid, Anthony Lanceleur, Julia Egbeola, Mathilde Neuville, Guillaume Tachon, Charles Cerf

##### Fondation Hopital Foch, Suresnes, France

**Correspondence:** Guillaume Tachon - g.tachon@hopital-foch.com

*Annals of Intensive Care* 2021, **11(Suppl 1):**FC-122

**Rationale:** Delayed cerebral ischemia (DCI) after subarachnoid hemorrhage (SAH) is a main cause of morbidity. Treatment is based on the induced hypertension (IH) with an objective of mean arterial pressure (MAP) above 90 mmHg. Use of Intravenous milrinone is increasing because of its vasodilatory effect and to increase cardiac output. Nevertheless, it could lead difficulties in maintaining target blood pressure.

**Patients and methods/materials and methods:** Retrospective, observational, single center study included patients suffering from DCI after SAH treated by intravenous milrinone and IH (MIH) or IH (IH) only. Invasive blood pressure was prospectively recorded every 60 s. The primary study endpoint was the hypotension dose (area under the curve) measure under the target MAP (90, 100, 120 mmHg) between both groups parametric test (Mann–Whitney, Chi^2^) were used. Multivariate analysis and propensity score with inverse probability treatment weighting methodology was performed integrating the variables associated with the allocation of milrinone treatment and those corresponding to the initial characteristics of the patients. Secondary endpoints were: need of rescue endovascular procedure, cerebral infarctus constitution, Rankin scale (mRs) at 6 months.

**Results:** Between 2011 and 2019, a total of 105 patients suffering from DCI were included: 71 in MIH group, and 34 in IH. Hypotension dose was lower in MIH than in IH group for all predetermined blood pressure level 90, 100 and 120 mmHg: 21 vs 66 mmHg.h (*p* = 0.01), 122 vs 266 mmHg.h (*p* = 0.006) et 1146 vs 1805 mmHg.h (*p* = 0.03), respectively. The times ratio of hypotension reported to treatment duration for average thresholds 90, 100, 120 mmHg was lower in MIH than IH group: 2.9% vs 9% (*p* = 0.001), 12% vs 26.2% (*p* = 0.001) and 70% vs 85% (*p* = 0.042), respectively. The median norepinephrin dose (mg/h) was not different between both groups. MIH patients were more severe (WFNS grade) than IH patients, needed more rescue procedures (61% vs 35% (*p* = 0.021)). Cerebral infarction was more frequent and proportion of good outcome (mRs < 3) was lower in MIH group: 61% vs 32% and 61% vs 85% (*p* = 0.03), respectively. After propensity score weighting, milrinone remained associated with lower hypotension dose for all average BP thresholds but proportion of good outcome was not different between two groups.

**Conclusion:** In our study, intravenous milrinone does not complicate the maintenance of blood pressure goals as part of induced hypertension therapy. Nevertheless, its potentially beneficial effect in treatment of delayed cerebral ischemia remained unclear and needs further evaluation.

**Compliance with ethics regulations:** Yes in clinical research.

### FC-123 Comparison of the non-specific gravity scoring systems in severe traumatic brain injury for prediction of mortality

#### Rania Ammar, Dorsaf Dlensi, Houda Mayoufi, Baccouch Najeh, Chokri Ben Hamida, Mabrouk Bahloul, Mounir Bouaziz

##### faculty of medecine of Sfax,university of Sfax, Sfax, Tunisia

**Correspondence:** Rania Ammar - rania.ammarzayani@gmail.com

*Annals of Intensive Care* 2021, **11(Suppl 1):**FC-123

**Rationale:** This study assessed the efficacy of the predicting power for in-ICU mortality of non-specific gravity scoring systems for severe traumatic brain injury in intensive care unit (ICU).

**Patients and methods/materials and methods:** A retrospective study was conducted over a period of 10 months in an intensive care unit including severe traumatic brain injury with age > 18 years and ICU stay > 48 h. APACHEII, IGSII scores at admission and SOFA, MODS and LODS scores were calculated at day 1, 3 and 5.

**Results:** Of the 37 included patients, mean age (SD) was 42 ± 15 years with a sex ratio of 6. Mean (SD) GCS was 6.8 ± 1.6. The mortality rate was 16% (6 patients). IGSII and APACHEII in deceased patients were higher than in survivors: 57 ± 21 vs. 38 ± 14 (*p* = 0.004) and 25 ± 7 vs 17 ± 4 (*p* = 0.001), respectively. ROC curve showed that IGSII > 53.5 had AUC = 0.82, Se 83% and Sp 93% (*p* = 0.013), and APACHE II > 20.5 has AUC = 0.84, Se 83% and Sp 87% (*p* = 0.01). LODS day1 and MODS day1 were higher in deceased patients than in survivors: 11 ± 5 vs 5 ± 2 (*p* = 0.034) and 10 ± 4 vs 5 ± 2 (*p* = 0.034), respectively. ROC curve showed that LODS day 1 > 6.5 has AUC = 0.847, Se 83% and Sp 87% (*p* = 0.008) and MODS day 1 > 5.5 has AUC = 0.815, Se 83% and Sp 67% (*p* = 0.016). LODS day 3 and MODS day 3 were higher in death patients than survivors: 9 ± 4 vs 4 ± 2 (*p* = 0.037) and 10 ± 5 vs 4 ± 2 (*p* = 0.05), respectively. ROC curve showed a MODS day 3 > 6.5 has AUC = 0.86, Se8 3% and Sp 90% (*p* = 0.005) and LODS day 3 > 6.5 has AUC = 0.88, Se 83% and Sp 93% (*p* = 0.003). The SOFA day 5, LODS day 5 and MODS day 5 were higher in deceased patients than in survivors: 11 ± 6 vs 5 ± 3 (*p* = 0.048), 9 ± 4 vs 4 ± 2 (*p* = 0.02) and 10 ± 5 vs 4 ± 3 (*p* = 0.032), respectively. ROC curve showed that a SOFA day 5 > 6.5 has AUC = 0.819, Se 83% and Sp 79% (*p* = 0.015), that LODS day 5 > 6.5 has AUC = 0.911, Se 83% and Sp 93% (*p* = 0.002) and MODS day 5 > 6.5 has AUC = 0.855, Se 83% and Sp 89% (*p* = 0.003).

**Conclusion:** The IGS II at admission was better to predict ICU-mortality than APACHE II, LODS d1 and MODS d1 with better sensitivity and specificity. The GCS did not demonstrate its validity as a predictive tool in severe traumatic brain injury. LODS d3 was superior to MODS d3, and LODS d5 was superior to MODS d5 and SOFA d5 with better sensitivity and specificity.

**Compliance with ethics regulations:** N/A.

### FC-124 Evaluation of the practice of transcranial Doppler in severe head trauma

#### Hamza Mimouni

##### CHU MOHAMMED VI OUJDA MAROC, Oujda, Morocco

**Correspondence:** Hamza Mimouni - hamzamimouni10@gmail.com

*Annals of Intensive Care* 2021, **11(Suppl 1):**FC-124

**Rationale:** The prevention of cerebral ischemia is the main objective of initial resuscitation of brain-injured patients. The first hours are crucial for the prognosis. The evaluation of cerebral perfusion is essential and feasible from the pre-hospital phase by transcranial Doppler (DTC), which helps to evaluate intracerebral hemodynamic rapidly and non-invasively at patient’s bedside, thus it facilitates the adaptation of immediate therapeutics.

**Patients and methods/materials and methods:** This is a descriptive prospective study of 71 cases conducted in the anesthesia and resuscitation department of the Mohammed VI Oujda University Hospital, over a period of 1 year between August 2017 and July 2018, including all traumatized serious skulls having benefited from a DTC, performed by the team of anesthesia–resuscitation department of the University Hospital Mohammed VI Oujda

**Results:** The mean age of patients was 39 years [8–83], with a male predominance. Reasons for admission were severe trauma (73%) followed by isolated head injury (27%), 17 patients (57%) had a GCS at admission less than 8 points. In 5 patients, DTC was in favor of a cerebral haemodynamic emergency with pulsatility index > 1.4 associated with a diastolic velocity < 20 cm/s. 39 measurements were supported, based on DTC. Evolution was marked by a total improvement in 33% of patients, 25% had a partial improvement due to diffuse axonal lesions in the majority of cases, 11 patients had a secondary neurological aggravation. The mortality rate was 15%. The mean duration of DTC was 7 min with extremes ranging from 16 min and 1 min. A marked improvement in the duration of the examinations was noted between the beginning and the end of the study.

**Conclusion:** DTC is the tool of choice to detect patients at risk of cerebral ischemia for optimizing immediate and individualized therapy and reducing the duration of cerebral ischemia that improves the prognosis. The use of DTC is rapid, non-invasive, feasible at the patient’s bedside, easy learning and reproducible, but requires regular practice. DTC has proved its place in the management of traumatic brain injury in the acute phase in detecting hemodynamic cerebral emergencies, and screening patients at risk of secondary neurological aggravation, and to guide the patient. Current efforts by the industry to miniaturize the components allow this technique to be considered in the resuscitation ambulance.

**Compliance with ethics regulations:** Yes in clinical research.

### FC-125 The prescription of antibiotics in pediatric intensive care units

#### Khadija Essafi, Elmehdi Soussane, Mohamed Anass Fehdi, Kaoutar Elfakhr, Samira Kalouch, Khalid Yaqini, Abdelaziz Chlilek

##### CHU Ibn Rochd, Réanimation pédiatrique, Casablanca, Morocco

**Correspondence:** Mohamed Anass Fehdi - mohamedanassf@gmail.com

*Annals of Intensive Care* 2021, **11(Suppl 1):**FC-125

**Rationale:** Bacterial resistance to antibiotics has increased dramatically over the last years, especially in the intensive care unit. We decided to conduct a study to evaluate antibiotics consumption and prescribing practices.

**Patients and methods/materials and methods:** We conducted a descriptive analytical, retrospective study of patients admitted to the pediatric intensive care unit at Ibn Rochd hospital, in year 2014. Data were collected from a standardized survey form. The adequacy of the antibiotic prescribed initially was analyzed and compared to clinical guidelines and bacteriological data.

**Results:** In this study, 314 patients were treated with antibiotics. The incidence of prescribing was 72%. The mean hospital stay was 7.9 days. While 47% of patients received antibiotic treatment prior to hospital admission, the empirical antibiotic therapy concerned 90% of the cases (data available in 86% of patients). The mean duration of antibiotic therapy prior hospital admission was 4.3 days. After admission, 88% of patients received curative antibiotic therapy, while only 16% of patients received a surgical antibiotic prophylaxis. The curative antibiotic therapy was empirical in 94% of cases based on clinical, biological, radiological and on the ecology of the hospital units. Community-acquired pneumonia (72%) was the main infection. The most frequent prescribed molecules were third-generation cephalosporins (61% of cases) followed by aminoglycosides and carbapenems. Only 16% of patients received monotherapy, while 84% of patients received a combination antibiotic therapy. The initial antibiotic therapy was modified in 72 patients or 26% of the cases. In 39% of cases, modification of initial antibiotic therapy was required to avoid worsening symptoms in 43% of the cases. The average duration of curative antibiotic therapy was 8.1 days. The results of the present study showed that the majority of antibiotics prescriptions in the intensive care unit were justified (98%) and consistent with bacteriology results in 87% of cases. However, only 75% of prescriptions were in accordance with clinical guidelines. The curative antibiotic therapy was excessive in 12% of cases, insufficient in 10% of cases, and inadequate in 1% of cases. The evolution antibiotic was associated with favorable response in 52% of patients. The prognosis was influenced by the age (*p* < 0.008), the severity of initial symptoms (*p* < 0.001) and accordance with clinical guidelines (*p* = 0.009).

**Conclusion:** All these findings showed that adequate antibiotic therapy prior to hospital admission had an impact on patient outcome suggesting that evaluation of prescribing practices can improve the antibiotics use.

**Compliance with ethics regulations:** N/A.

### FC-126 Carbapenems in pediatrics: compliance with guidelines

#### Clara Cebron, Guillaume Mortamet, Julie Arata-Bardet, Marie Martinod

##### CHU GRENOBLE ALPES, Grenoble, France

**Correspondence:** Clara Cebron - cebronc@gmail.com

*Annals of Intensive Care* 2021, **11(Suppl 1):**FC-126

**Rationale:** The increase of carbapenem resistance is a major health problem and improving the use of carbapenem is now crucial. Although the pediatric population is very specific, there still be a lack of data in children. Our aim was to assess the compliance of carbapenem prescriptions with guidelines in pediatrics.

**Patients and methods/materials and methods:** We carried out a retrospective study over a 1-year period (2019) in our university hospital. All children admitted who received at least one dose of carbapenem were included. The use of carbapenem was considered as appropriate when 1/compliant with the most recent local/national/international guidelines and/or 2/approved by an pediatric infectious disease consultant at initiation and/or 3/approved by two infectious disease consultants when reviewed retrospectively. As a secondary outcome, we analysed the review of carbapenem at 72 h after initiation.

**Results:** We collected a total of 96 carbapenem prescriptions (80 (83%), 10 (10%) and 6 (6%) for meropenem, imipenem and ertapenem, respectively) in 75 patients (median age 46 months [IQR 4–115], 34 males). In 61 cases, a bacteria was isolated including 19 (29%) extended spectrum β-lactamases (ESBL)-producing Enterobacteriaceae, mostly *Escherichia coli* (*n* = 12, 63%) and *Klebsiella pneumoniae* (*n* = 6, 32%). Overall, most of carbapenem prescriptions were considered as appropriate at initiation (*n* = 89, 93%) whether there were empirical (*n* = 71, 92%) or documented (*n* = 18, 95%). The main reasons for carbapenem prescription according to the physician in charge were the unfavorable evolution while treated by another antibiotic (*n* = 33, 34%), the presence of ESBLs-producing bacteria risk factors (*n* = 21, 21%), the degree of criticalness (*n* = 22, 23%) or the presence of neutropenia (*n* = 19, 20%). The treatments were stepped down at 72 h in 30 (31%) of prescriptions. For 38 (39%) of prescriptions, there was no alternative to carbapenem given the bacteriological documentation.

**Conclusion:** We found that most of carbapenem prescriptions were considered as appropriate at initiation in a retrospective pediatric cohort. At 72 h after initiation of carbapenem, around two thirds of prescriptions should have been improved with a step down strategy.

**Compliance with ethics regulations:** Yes in clinical research.

### FC-127 Contribution of a weekly interdisciplinary rounds with a pediatric infectious disease spescialist in a pediatric intensive care unit

#### Marion De Gregorio, Julie Arata Bardet, Cécile Bost Bru, Guillaume Mortamet

##### CHU Grenoble Alpes, Grenoble, France

**Correspondence:** Marion De Gregorio - mdegregorio@chu-grenoble.fr

*Annals of Intensive Care* 2021, **11(Suppl 1):**FC-127

**Rationale:** Primary or secondary infectious disease are common in Pediatric Intensive Care Units (PICUs). The main objectives of this study were 1/to describe the clinical recommendations given on a weekly basis by an infectious disease specialist into a PICU and 2/to assess its compliance.

**Patients and methods/materials and methods:** A observational prospective single-center study conducted over a 5-month period in a PICU of a tertiary university hospital. All clinical recommendations provided by an infectious disease specialist during a weekly round were collected.

**Results:** We reported a total of 92 clinical recommendations interesting 66 patients (median age 34 months [IQR 4 month–10 years]). Infections occurred in patients with comorbidity in 32 (48%) of them. Most of them had pulmonary infections (*n* = 46, 50%) and patients presented with septic shock in 15 cases (18%). Most of infections were related with a multiresistant bacteria (*n* = 7, 8%). Clinical recommendations were considered as preventive, diagnosis, monitoring and therapeutic in 9 (10%), 35 (8%), 18 (20%) and 80 (89%), respectively. Treatment was optimized in 55 (60%) of cases, including treatment discontinuation (*n* = 27, 30%), spectrum reduction (*n* = 7, 8%) and dosage adjustment (*n* = 8, 9%). The duration of treatment was also optimized in 57 cases (63%).Overall, we found a full compliance in most of cases (*n* = 85, 92%).

**Conclusion:** We found a very good compliance of clinical recommendations provided on a weekly basis by an infectious disease specialist in a PICU. Larger pediatric studies are warranted to assess a potential benefit on patients’ outcome.

**Compliance with ethics regulations:** Yes in clinical research.

### FC-128 Descriptive study of therapeutic drug monitoring of antibiotics in critically ill children

#### Noémie De Cacqueray^1^, Sana Boujaafar^2^, Emmanuelle Bille^1^, Florence Moulin^1^, Inès Gana^2^, Sihem Benaboud^2^, Déborah Hirt^2^, Agathe Béranger^1^, Julie Toubiana^1^, Sylvain Renolleau^1^, Jean Marc Tréluyer^1,2^, Mehdi Oualha^1^

##### ^1^Necker, Paris, France; ^2^Cochin, Paris, France

**Correspondence:** Noémie De Cacqueray - n_a_cacqueray@hotmail.fr

*Annals of Intensive Care* 2021, **11(Suppl 1):**FC-128

**Rationale:** Septic critically ill children are subject to changes in pharmacokinetics (PK) parameters leading to high risk of inadequate antibiotics exposure. Therapeutic drug monitoring (TDM) is a growing strategy that improves the likelihood to reach the PK/PD targets. It is commonplace for very few antibiotics but occasionally used for any antibiotic. We aimed to depict the use and related impact of TDM in critically ill children.

**Patients and methods/materials and methods:** We conducted a 6-month prospective single center study to review how TDM was performed and applied by clinicians in critically ill children receiving antibiotics. Blood samples were collected during routine laboratory tests and antibiotics were quantified using LC–MS/MS or HPLC–UV.

**Results:** From June to December 2019, 209 children with a median [IQR] age of 2.4 [0.5–9] years received antibiotics. TDM was performed in 58 (28%) patients with a total of 208 samples. Children undergoing TDM had more organ dysfunctions (3 vs 1, *p* < 0.05), were more often immunocompromised (64% vs 29%, *p* < 0.05) and received longer antibiotic therapies (96% vs 89% long treatments, *p* = 0.012). Median [IQR] time to results was three [1–5] days and majority of the patients did not reach the PK/PD targets; 40 (53%) of the 75 off-target concentrations available in due time led to dosing adjustments. After adjustment, concentrations better achieved PK/PD targets (70% vs 20%, *p* 0.013). Initial pharmacological evaluation using surrogates from the European Committee on Antimicrobial Susceptibility Testing (EUCAST) data were not consistent with remote analysis based on measured minimum inhibitory concentration (MIC) in ten (20%) cases.

**Discussion:** This study demonstrates, once again, that antibiotic dosing in PICU is not adequate, with frequent low concentrations. Facing this, TDM in PICU led to changes in practices with dosing adjustments that improved achievement of the PK/PD targets. A few weaknesses limited the value of TDM such as long time to results and the use of surrogates for MIC. Moreover, other PK tools are being developed to guide dosing adjustment at the patient’s bedside.

**Conclusion:** TDM may be effective to improve antibiotic exposure in critically ill children. It requires timely available antibiotic plasma concentration and measured MIC. It is mandatory to define which patients should benefit from TDM and how TDM should be managed.

**Compliance with ethics regulations:** Yes in clinical research.

### FC-129 Management of subarachnoid hemorrhage and cerebral vasospasm in children

#### Clément Isola, Anne Millet, Amélie Desrumaux, Isabelle Wroblewski, Jean-Francois Payen, Guillaume Mortamet

##### CHU de Grenoble, Grenoble, France

**Correspondence:** Guillaume Mortamet - gmortamet@chu-grenoble.fr

*Annals of Intensive Care* 2021, **11(Suppl 1):**FC-129

**Rationale:** The present study aims 1/to report the incidence of cerebral arterial vasospasm in a cohort of children with subarachnoid hemorrhage (SAH), 2/to describe how transcranial Doppler can be useful in this clinical application and 3/to describe the management of patients with cerebral arterial vasospasm.

**Patients and methods/materials and methods:** A single-center retrospective study performed over a 10-year period, from January 2010 to December 2019. All patients from one month to 18-years old admitted to the pediatric or adult intensive care unit (ICU) with a diagnosis of SAH were eligible.

**Results:** Data from 80 patients (65% male, median age 8.6 [3.3–14.8] years) were available. SAH was trauma-related in most of patients (*n* = 59, 74%) and a total of 14 patients (18%) developed a cerebral vasospasm. The non-trauma cause of SAH was the only independent factor associated with the occurrence of vasospasm (*p* < 0.001). The vasospasm was suspected by TCD before being confirmed by CT or MR angiography in eight patients out of 14 (57%). Increased velocities and decreased pulsatility index were found in these patients. All 14 patients who developed vasospasm were treated by curative intravenous milrinone without side-effect. Finally, death occurred in 12 (15%) patients with a similar incidence between patients who developed vasospasm and those who did not.

**Conclusion:** A significant proportion of pediatric patients who have suffered SAH developed cerebral arterial vasospasm during the course of their treatment. Children in whom vasospasm developed were more likely to have a non-trauma-related SAH.

**Compliance with ethics regulations:** Yes in clinical research

### FC-130 Diabetic ketoacidosis in pediatric intensive care: prognostic factors

#### Samira Kalouch, Khalid Yaqini, Aziz Chlilek

##### chu ibn rochd, Casablanca, Morocco

**Correspondence:** Samira Kalouch - dr.kalouch@gmail.com

*Annals of Intensive Care* 2021, **11(Suppl 1):**FC-130

**Rationale:** Diabetic ketoacidosis (DKA) is a metabolic emergency and one of the main causes of morbidity and mortality in diabetic children through its complications.

**Patients and methods/materials and methods:** We conducted a retrospective study, including 122 cases admitted to the pediatric intensive care unit, between January 2010 and January 2020. The different variables collected on admission were analyzed and then compared between two groups: the deceased patients d and the survivors. Statistical analysis was carried out with SPSS 21 software.

**Results:** 122 cases of diabetic ketoacidosis were identified. The average age was 7.6 years old. Consanguinity was found in 13% of our patients. Ketoacidosis is inaugural in 62% of cases. The polyuro–polydipsia syndrome was found in all patients, followed by digestive disorders in 56% of cases. The mean GCS on admission was 12. A state of shock was found in 17% of cases and polypnea with Kussmaul-type breathing in 71% of cases. 10% of patients had severe dehydration. The mean capillary blood glucose on admission was 4.9 g/l. Arterial gas analysis was performed in 60 patients, 36 patients had profound metabolic acidosis. The procalcitonin was requested in 47 patients (33%), positive in 15% of patients. ECBU was requested in 84 patients (59%), it revealed a urinary tract infection in 9 (6%) of them. Brain CT was performed in 77 patients (54%). It was normal in 53% of cases, on the other hand it objectified a cerebral edema in 33 patients or 43%. The mortality rate was 15%. The treatment consisted in an adequate rehydration associated with insulin therapy, according to the protocol for the service. In analyzed united varied, factors significantly associated with mortality are the altered consciousness, shock, fever, use of antibiotics on admission, brain edema, nosocomial infection, use of mechanical ventilation, use of vasopressor and setting up a catheter exchange. In multivariate analysis, factors associated with mortality were the alteration of consciousness, brain edema, nosocomial infection, use of mechanical ventilation, administration of vasopressors.

**Conclusion:** The diagnosis of DKA must be made early and requires a well-codified management. Difficult access to care, poor hospital equipment and the lack of sufficient pediatric intensive care and resuscitation units remains the main challenge responsible for an increasing incidence of DKA and a non-negligible mortality rate.

**Compliance with ethics regulations:** Yes in clinical research

### FC-131 Acute renal failure in intensive care complicating rhabdomyolysis: a complex situation

#### Setti Aouicha Zelmat, Djamila Bouabida, El Hassan Boucherit, Fatema Mazour

##### Faculté de médecine, Oran, Algerie

**Correspondence:** Setti Aouicha Zelmat - Settiaouichazelmat@yahoo.fr

*Annals of Intensive Care* 2021, **11(Suppl 1):**FC-131

**Rationale:** The occurrence of acute renal failure (AKI) complicating rhabdomyolysis is often multifactorial, however more common in adults in the intensive care unit of medico-surgical emergencies in a hospital establishment. Our objective is to determine the risk factors for the occurrence of this AKI sequel to rhabdomyolysis and to define the prognosis.

**Patients and methods/materials and methods:** Retrospective study on the charts of patients admitted for medical and surgical emergencies over a period of 2 years (2017–2019). We identified all the complicated rhabdomyolysis from an AKI. AKI was defined according to the RIFLE criteria, the parameters studied: demographic variables, time to onset of AKI, causes of rhabdomyolysis, biological variables (myoglobinemia assay) and clinical variables. 234 patients were included among the 856 patients treated for medical and surgical emergencies.

**Results:** Two hundred and thirty-four patients presented with rhabdomyolysis, the etiological aspects of which on admission were represented by: medical cause (59/234), surgical cause (98/234), traumatic cause (77/234). During the 2-year time interval, AKI occurred in (146/234) 62.4% of patients with rhabdomyolysis; (31/146) 21% of AKI patients were on hemodialysis. The length of stay was (5–60 days). The course was known by complete recovery in 112 patients (76.7%), death occurred in 15 patients (10.3%), 19 patients (13%) progressed to chronic renal failure. The etiopathogenic factors of AKI complicating rhabdomyolysis were: uncorrected hypovolemia (82/146), sepsis (27/146), nephrotoxicity (10/146) and bed rest (27/146). Twenty-six patients (17.8%) required mechanical ventilation, 25.3% required hemodynamic support.

**Discussion:** Multivariable analysis showed that the severity of uncorrected hypovolemia and bed rest remain major risk factors for the onset of post-rhabdomyolysis ARI in intensive care. Due to the shortage of resuscitation nurses, nursing care cannot be done properly to avoid complications from agitation. AKI occurrence in the context of rhabdomyolysis is frequent in intensive care units and is fraught with significant morbidity and mortality.

**Conclusion:** Post-rhabdomyolysis AKI is common in intensive care. Its treatment is long and complex, however its prevention is simple by identifying risk situations including rhabdomyolysis, its treatment consists of the rapid correction of hypovolemia in time.

**Reference**

1. Patrick A. Torres, John A. Helmstetter, Adam M. Kaye and Alan David Kaye Ochsner Journal March 2015, 15 (1) 58–69.
2. Huerta-Alardín AL, Varon J, Marik PE. Bench-to-bedside review: Rhabdomyolysis—an overview for clinicians. Crit Care. 2005;9(2):158–69. [PMC free article] [PubMed] [Google Scholar]

**Compliance with ethics regulations:** Yes in clinical research.

### FC-132 Effectiveness of antivenom in patients consulting lately after snake bite

#### Stéphanie Houcke^1^, Guy Roger Lontsi Ngoula^1^, Didier Hommel^1^, Patrick Djahi^1^, Dabor Resiere^2^, Hatem Kallel^1^

##### ^1^Centre Hospitalier de Cayenne, Cayenne, Guyane Francaise; ^2^CHU Martinique, Fort De France, Martinique

**Correspondence:** Hatem Kallel - hatem.kallel@ch-cayenne.fr

*Annals of Intensive Care* 2021, **11(Suppl 1):**FC-132

**Rationale:** Snakebite envenoming is an acute emergency requiring an early care delivery. However, in some cases, patients can take several hours before consulting or attending the hospital and receive antivenom (AV). We conducted this study to search for the effectiveness of antivenom in patients consulting tardily after snake bite in French Guiana.

**Patients and methods:** Our study is a prospective observational work. It was conducted in the Intensive Care Unit (ICU) of Cayenne General Hospital between January 1st, 2016 and, December 31, 2019. We included all patients hospitalized for snakebite envenoming. We excluded all patients who received AV in less than 6 h from SB. Our study contained two groups (without AV, AV > 6 h).

**Results:** During the study period, 106 patients were included. The mean age of patients was 40 ± 17 years and 74.5% were male. The meantime from SB to the first medical consultation was 9:27 ± 16:51. The mean time from SB to antivenom therapy was 20:28 ± 14:41. The main clinical symptoms were edema (98.1%) and pain (96.2%). Biological parameters at admission showed defibrinogenation in 96 cases (91.4%), thrombocytopenia in 38 cases (36.5%), hemolysis in 28 cases (26.7% %), and rhabdomyolysis in 38 cases (36.2%). International normalized ratio was > 2 in 79 cases (75.2%), and partial thromboplastin time was > 1.5 in 49 cases (46.7%). Time from SB to normal clotting analyses was shorter in patients receiving AV.

**Conclusion:** Patients receiving AV > 6 h showed a reduction in the time to return to normal clotting tests, as compared to those managed without AV. We suggest that AV is effective in patients consulting lately after snake bite and receiving AV > 6 h.

**Compliance with ethics regulations:** Yes in clinical research.
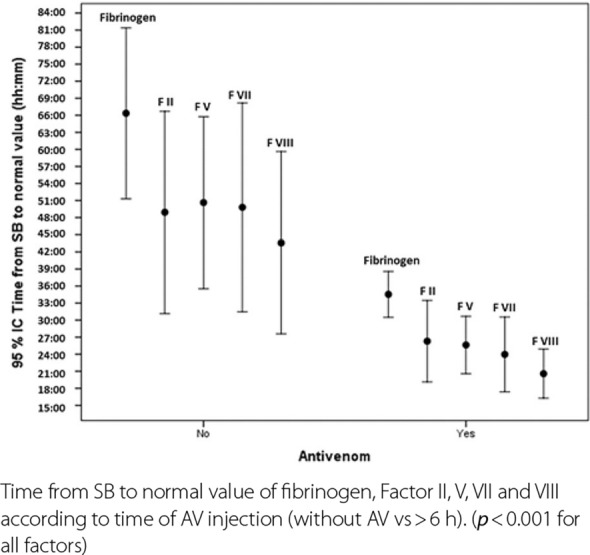


Time from SB to normal value of fibrinogen, Factor II, V, VII and VIII according to time of AV injection (without AV vs > 6 h). (*p* < 0.001 for all factors)

### FC-133 Ecstasy recreational drug abuse: an emergency overview

#### Ameni Khaled, Ameni Abidi, Hatem El Ghord, Ammar Mhamdi, Hafedh Thabet

##### Service des urgences CAMU Tunis, Tunis, Tunisie

**Correspondence:** Ameni Khaled - ameni.khaled1988@gmail.com

*Annals of Intensive Care* 2021, **11(Suppl 1):**FC-133

**Rationale:** The 3.4 methylenedioxymethamphetamine, known as ecstasy, is one among many methylated amphetamine derivatives. It has become the recreational drug of choice for adolescents. Ecstasy poisoning can cause sever life-threatening pathologies which remain poorly codified. The aim of this study was to identify the initial gravity of ecstasy absue.

**Patients and methods/materials and methods:** This was a retrospective study from February 2019 to November 2020 including patients with acute ecstasy poisoning managed in the emergency department (ED). We collected clinical and therapeutic findings and early outcome

**Results:** 80 cases were enrolled. The gender ratio was 4.3. The median age of patients was 24 ± 7 years. Most patients were attended on summer months (August and September). A history of drug abuse was recorded in 45%. Forty-one percent of patients had consumed additional drugs (alcohol 23%; cannabis 21% and cocaine 6%). The main reasons for attendance at the ED were cognitive disturbances (35%); agitation (20%) and chest pain (7%). The initial neurological examination noticed agitation (20%), hemiparesis in 4 cases. The electrocardiograph showed morphological abnormalities in 8 cases. Rhythm disturbances were found in 10 cases (sinus tachycardia *n* = 8 and atrial fibrillation *n* = 2). Biological findings showed rhabdomyolysis in 7 cases and high troponin I concentration in 2 cases. The short-term outcome was favorable in 88% of cases. Six patients were admitted to the ICU. A number of pills > 2 (*p* = 0.05; OR = 2.8; IC95% = 1.521–15.678) and heart rate > 100 beats per minute (*p* = 10^−3^; OR = 8.9; IC95% = 1.474–54.024) were significantly associated with admission ICU.

**Discussion:** Studies have shown the severity of NMDA abuse were correlated with rhabdomyolysis and male gender. This difference could be explained by the heterogeneity of drug composition.

**Conclusion:** Ecstasy overdose is a serious problem. The occurence of sever forms is related to quantity consumed > 2 pills and heart rate > 100 bpm.

**Reference**

1. Richards JR, Wang CG, Fontenette FW. Rhabdomyolysis, methamphetamine, amphetamine and mdma use: associated factors and risks. JDD. 2020. 429–37.

**Compliance with ethics regulations:** Yes in clinical research.

### FC-134 Pregabalin poisoning in Tunisia: a rising phenomenon

#### Ameni Abidi, Ameni Khaled, Hatem El Ghord, Hafedh Thabet

##### Centre d’assistance médicale urgente et de réanimation, Tunis, Tunisie

**Correspondence:** Ameni Abidi - drabidiameni@gmail.com

*Annals of Intensive Care* 2021, **11(Suppl 1):**FC-134

**Rationale:** Pregabalin use have been recently associated to recreational drug abuse, outshining other substances as we have witnessed a recent increase in the number of patients presenting to emergency. Therefore, it would be interesting to acknowledge the particularities of this rising intoxication.

**Patients and methods/materials and methods:** We conducted a review of all patients presenting with pregabalin usage during 25 months, starting from January 2019 to January 2021. Cases were identified and noted.

**Results:** Twenty-six cases were collected during the study period. Median age was 20 [21; 53] with extremes ranging from 15 to 53 years. Gender ratio was of 1, 13 males and 13 females. Globally, intoxications became more frequent in August (11%), September (15%), October (15%) and mainly in January (19%). Only three patients (11%) had a history of drug abuse. Five (19%) had a follow-up in psychiatry for a depressive disorder, including a patient who attempted suicide with a rodenticide identified as chloralose that required invasive mechanical ventilation. Two (8%) had diabetes mellitus, one (4%) was dyslipidemic and had chronic obstructive pulmonary disease. The mean delay to presenting to the emergency department was of 6 (± 3.2), [1; 12] h. Pregabalin use was unanimously voluntary. It was associated to other substances in seventeen cases (65%) largely benzodiazepines in nine cases (35%) led by prazepam (11%) and alprazolam (8%).In addition, two patients (8%) smoked cannabis and another used 3,4-methylenedioxymethamphetamine and ethyl alcohol. No opioids, nor cocaine were taken. Twenty-five (96%) patients ingested pregabalin and one (4%) sniffed it. Median pregabalin supposed ingested dose was of 750 [431; 1687] with extremes from 150 to 4500 mg. At first consultation, two (8%) were comatose, two (8%) had seizures, both (8%) showed dysarthria and the rest of sixteen (61%) did not show any sign of poisoning. Pupils examination showed mydriasis in three (11%) cases and miosis in one (4%) case. Osteotendinous reflexes were sharp in only one of the poisoned patients. In total, seven (27%) were admitted to the intensive care unit. Among them four (15%) required mechanical ventilation. Four were transferred to a psychiatric unit and thirteen have evolved well during observation in the emergency room.

**Conclusion:** Doctors should be aware of pregabalin toxicity and illicit misuage leading to dependency and furthermore to poisoning, although current data and opinion suggest that pregabalin has a very low potential for abuse. Moreover, larger surveys should be run regarding its accurate incidence.

**Compliance with ethics regulations:** Yes in clinical research.

### FC-135 Prognosis and outcomes of heatstroke in ICU: a multicenter retrospective study

#### Raphael Devanlay, Sébastien Moschietto, Pierre Ducq, Antoine Frouin, Florent Montini

##### Centre Hospitalier Henri DUFFAUT, 84000 Avignon, France

**Correspondence:** Raphael Devanlay - raphael.devanlay@gmail.com

*Annals of Intensive Care* 2021, **11(Suppl 1):**FC-135

**Rationale:** Heatstroke is the most hazardous heat induced illness. It mays be categorized as classic or exertional. This condition is quite rare and often misunderstood by Intensive Care Units (ICU) practitioners but seems to increase due to global warming. We wanted to study the epidemiological characteristics and the prognostic of heatstroke in ICU in the South of France.

**Patients and methods/materials and methods:** Multicenter retrospective observational study among patients hospitalized for heatstroke in ICU of two hospitals between May 2018 and September 2019. Data analyzed were: characteristics of heatstroke patients, pre-hospital care, ICU care, evolution and prognostic.

**Results:** We enrolled 15 patients who suffered a heatstroke. Median age was 42.5 years old. 14/15 were men. We observed 8 classics heatstroke and 7 exertionals. Only 20% had cardiac history, as hypertension or arrhythmia. 26.67% had psychiatrics conditions and 40% used to take psychotropic drugs. Patients were critical, with a SOFA score of 9.27 [6.223–12.310], 8 patients needed mechanical ventilation during pre-hospital care (PHC) and 2 more were intubated in the ICU. The mean Glasgow Score was 7.27 during prehospital care and 6.07 in ICU [3.366–8.767]. The length of ICU stay was 6.73 days (with 3 patients who stayed more than 18 days). We observed 63% of acute kidney injury (KDIGO ≥ 2), only one with severe hepatic cytolysis but 12 with prothrombin < 70%. 2/3 had criteria of disseminated intravascular coagulation (DIC). 3 patients died and one suffered from severe neurological disability. We found a tendency between initial temperature and SOFA score but our study lacked statistical power. We dichotomized patients between classical or exertional. Patients with classical heatstroke had more medical history, and morbi-mortality was higher. Exertional heatstroke patients were younger (36.57 years old), none hade medical history Only 6 patients had external cooling, with ice on the body every time. Cooling seems to reduce ICU stays but it was non-significative.

**Conclusion:** This is the first multicenter study, with only ICU patients suffering from heatstroke. Exertional and classical heatstrokes share the same physiology but are different on clinical, biological and prognostic plans. These results need to be confirmed with a bigger group on a prospective study in the South of France in the context of global warming.

**Compliance with ethics regulations:** Yes in clinical research.

### FC-136 Suicide attempts in intensive care unit: comparison of patients with and without mental disorders

#### Jihene Guissouma, Meriam Ksouri, Hatem Ghadhoune, Habib Brahmi, Hana Ben Ali

##### Hopital universitaire Habib Bougatfa, Bizerte, Tunisie

**Correspondence:** Jihene Guissouma - guissouma.jihene@gmail.com

*Annals of Intensive Care* 2021, **11(Suppl 1):**FC-136

**Rationale:** Suicide attempt (SA) is an urgent health problem worldwide. Mental disorders are known as the strongest risk factors for suicide attempts and deaths. In this study we aimed to compare the epidemiologic, clinical features and the outcome of suicide attempters with and without psychiatric history (PH) admitted in the intensive care unit (ICU).

**Patients and methods/materials and methods:** A 3-year (2018 to 2020) prospective analytic observational single-center study including all suicide attempters admitted in a 6-bed intensive care unit. The cohort was divided in 2 groups (group A: patients with PH and group B: patients without PH). Statistical analysis was performed using the statistic software SPSS 23.

**Results:** Among 81 patients enrolled 33 (41%) had history of mental disorder. The most common were depression and bipolar disorder. Mean age was 29 ± 13 years with female predominance (sex ratio = 0.37). There was no significant difference between the 2 groups; in the gender, socio-economic conditions, marital status and the type of intoxication. Severity of the initial clinical presentation (GCS, hypoxemia and hemodynamic instability), need of invasive ventilation and evolutionary complications were also comparable in the 2 groups. Mortality rate was 6% and doesn’t vary between them. However, there was a significant difference in the age (*p* = 0.001) as well as patients with PH were older (with mean age in group A = 37 ± 16 years and 23 ± 9 in group B). Besides patients with PH had more previous episodes of SA (*p* = 0.016) and the majority of them had no occupation (*p* = 0.007). Mean SAPS II was higher in group A than in group B(45 ± 19 versus 36 ± 12 with *p* = 0.023). Even though the need of invasive ventilation were similar in the 2 groups, its mean duration was longer in patients with PH (4 ± 6 days versus 2 ± 1 day with *p* < 10^−3^). The mean length of stay was longer in group A (6 ± 7 days versus 3 ± 2 days with *p* = 0.003).

**Conclusion:** Suicide attempters with mental disorder were older and recidivists compared with patients without PH. Even though there were no significant difference in the method of suicide and the severity of the initial clinical presentation; SAPS II, mean length of ventilation and ICU stay were significantly higher in patients with mental disorder. This emphasizes the importance of an optimal psychiatric care in order to prevent both relapse of mental disorders and recurrence of SA.

**Compliance with ethics regulations:** Yes in clinical research.

### FC-137 pre- and post-operative prognostic factors of very elderly patients (> 85 years) admitted in intensive care unit following unplanned surgery: a 5-year retrospective survey

#### Dominique Prat, Frédéric Jacobs, Olfa Hamzaoui, Marta Luperto, Charles Damoisel, Benjamin Sztrymf

##### Hôpital Antoine Béclère, Clamart, France

**Correspondence:** Benjamin Sztrymf - benjamin.sztrymf@aphp.fr

*Annals of Intensive Care* 2021, **11(Suppl 1):**FC-137

**Rationale:** “Old patients” represent the fastest growing subgroup of the population, with a subsequent increase in surgery demand. The usual pre-operative evaluation may not be applicable in the setting of surgical emergencies. This study addressed pre and post-operative prognostic factors of very elderly patients (> 85 years) admitted in ICU after unplanned surgery.

**Patients and methods/materials and methods:** Retrospective study over a 5-year period. All patients aged 85 years or more admitted in ICU after unplanned surgery were included. Demographic data, place of living, autonomy, chronic disability and frailty were registered in the pre-operative period. Type of surgery was registered. Worse ODIN score up to 48 h after ICU admission was registered, as well as SAPS II, occurrence of life sustaining treatment limitations (LSTL), ICU and hospital outcomes.

**Results:** 2613 patients were admitted during the study period among which 398 (15.2%) were aged 85 years or more and 69 patients were included. Age was 89.5 [87.1–92.4] years. ICU length of stay was 4 [1.8–10] days. Patients were included after digestive (*n* = 43) or orthopedic surgery (*n* = 26). Overall ICU and hospital survival were, respectively, 72.4% and 66.7%. Pre-operative assessment: more pre-frail and frail patients had a bad evolution (66% vs. 94.8%, *p* = 0.013). These patients also had a higher pre-operative ODIN score. In univariate analysis, frailty and pre-operative ODIN score were associated with survival. Only frailty was associated with outcome in multivariate analysis (9.55 [2.43–37.4]; *p* = 0.0013). Post-operative assessment: ODIN score and its kidney and haematologic components and SAPS II were associated to survival. In univariate analysis, SAPS II, post-operative ODIN score and the increase in ODIN as compared to pre-operative evaluation were associated with ICU survival. LSTL occured in 31 patients, consisting in 19 withholding and 12 withdrawing decisions. Age, baseline decreased autonomy and baseline kidney disease were associated with the occurrence of LSTL decision. LSTL decisions occurred as soon as the first ICU day in 18 patients. These early LSTL consisted in 11 withholding decisions leading to 3 deaths within 1 [1–3.3] days and 7 withdrawal decisions leading to 7 deaths within 1 [1–1.8] days

**Conclusion:** Overall ICU survival of very old patients after unplanned surgery was 72.4%. Frailty was a reliable and independent pre-operative prognostic factor. Managing patients in the post-operative setting in ICU can be very stressful and demanding. Therapeutic endpoints should be clearly defined together with patients and family whenever possible, to deliver the most appropriate care and avoid caregiver’s moral distress.

**Compliance with ethics regulations:** Yes in clinical research.

### FC-138 Admission for elderly patients (> 80 years old) in ICU by SAMU-SMUR

#### Kais Regaieg, Ammar Al Sheikhly, Mehdi Oulmouddane, Giulia Moratelli, Amir Al Harach, Laure Gazaigne, Dany Goldgran-Toledano

##### GHT GRAND-PARIS NORD-EST, Montfermeil, France

**Correspondence:** Kais Regaieg - kais.regaieg@gmail.com

*Annals of Intensive Care* 2021, **11(Suppl 1):**FC-138

**Rationale:** The French prehospital healthcare delivery system (SAMU-SMUR) allows the admission of patients directly in ICU. The objective of our study is to describe the epidemiological and clinical characteristic of elderly patients (> 80 years old) admitted in ICU by SAMU-SMUR and to verify that pre-hospital treatment, admission into ICU and the management were coherent.

**Patients and methods/materials and methods:** We conducted a retrospective observational study that included all elderly patients (> 80 years old) admitted to the intensive care unit (ICU) of general hospital between January 2018 and December 2020. We collected demographic and clinical data, prehospital treatment, management in ICU. Exploratory outcomes include univariate analyses of baseline characteristics associations with 90-day mortality. The statistical analyses were conducted in accordance with the predefined statistical analysis plan.

**Results:** During the study period, a total of 59 elderly patients admitted to the ICU by SAMU-SMUR. The mean of age was 84 (80–94). The sex ratio (M/W) was 1.18. 93% of patients had GIR score 5–6. The most common comorbidity was cardiovascular diseases (74%). The other co-morbidities were diabetes (33%), COPD (27%) and chronic kidney disease (15%). Reasons of calling SAMU-SMUR were respiratory distress (57%), cardio-pulmonary arrest (16%), coma (11%) and shock (10%). The prehospital diagnosis was confirmed by the hospitalisation report in 95% of cases. Prehospital management consisted of oxygenotherapy (74%), CPAP (28%), mechanical ventilation (27%) and fluid replacement ± vasopressors (20%). In ICU, 40% of patients were mechanically ventilated [(median duration: 6.4 days (1–40)], 40% of patients were given vasopressors, and 28% of patients underwent CPAP. The mortality in ICU was 32% and 90-day mortality was 37%. The mean stay in ICU was 6.8 days (1–60). The mean duration of hospitalization of the patients who survived was 17 days (1–110). On the basis of an univariate analysis: male, mechanical ventilation and shock were associated with increased mortality (*p* < 0.01).

**Conclusion:** Our results show that the admission of elderly patients in ICU as well as prehospital management seems justified. Others studies are needed on this subject.

**Compliance with ethics regulations:** Yes in clinical research.

### FC-139 Malignant nephroangiosclerosis in young patients with malignant hypertension

#### Côme Bureau^1^, Matthieu Jamme^1^, Juliet Schurder^1^, Mickaël Bobot^2^, Thomas Robert^2^, Aymeric Couturier^3^, Jean Michel Halimi^4^, Xavier Bellenfant^5^, Eric Rondeau^1^, Laurent Mesnard^1^

##### ^1^Assistance Publique – Hôpitaux de Paris, Urgences Néphrologiques et Transplantation Rénale - Hôpital Tenon, Paris, France; ^2^Assistance Publique – Hôpitaux de Marseille, Centre de néphrologie et de transplantation rénale - Hôpital de la Conception, Marseille, France; ^3^Service de néphrologie - CHU de Pointe à Pitre, Poite A Pitre, France; ^4^Service de néphrologie - CHRU de Tours - Hôpital Bretonneau, Tours, France; ^5^ Service de néphrologie - CHI André Grégoire - Hôpital de Montreuil, Montreuil, France

**Correspondence:** Côme Bureau - come.bureau@gmail.com

*Annals of Intensive Care* 2021, **11(Suppl 1):**FC-139

**Rationale:** The lesions of nephroangiosclerosis are the histopathological consequences of malignant hypertension (MH). The pathophysiology of MH is commonly extrapolated from the essential arterial hypertension and aging processes. Genetic causes are poorly understood, especially in young patients with renal impairment. Some authors even consider the renal disease per se as the very starting point of MH when associated with renal failure. The objective of our study is to identify both clinical and prognosis data in patients under 40 years old at enrollment receiving a kidney biopsy at their diagnosis of MH.

**Patients and methods/materials and methods:** Patients were identified retrospectively and prospectively through the pathology department databases of 8 French hospitals over 35 years (1985–2020) with the key words “nephroangiosclerosis” and “thrombotic microangiopathy”.

**Results:** 114 patients have been included, they were men (*N* = 77, 67%) aged from 34 years on average; 35% were Caucasians and 35% were African Americans. Diagnosis of an “indeterminate” vascular disease (IVD) was made in 52% of cases. The diagnosis of a primary glomerulopathy was eventually made for 24% of the patients; among them 39% were Berger’s disease. We observed serious events associated with the kidney biopsy procedure in 6%. In our selected population, only 7% of the patients had normal renal function, 33% required dialysis and 21% were eventually transplanted. Mortality was 1%. After multivariate analysis, the prognostics factors significantly associated with renal prognosis (dialysis at 6 months) was creatininemia at the time of admission to the hospital (OR = 1.56, 95% CI: [1.34–1.96], *p* < 0.001) and renal fibrosis > 30% (10.73, [1.53–112.03], *p* = 0.03). Astonishingly, presence of thrombotic microangiopathy lesion on renal biopsy was a protective factor (0.14, [0.02–0.60], *p* = 0.01). Moreover, no significant pathologic or prognostic differences were found between Caucasians and African-Americans patients.

**Conclusion:** IVD/nephroangiosclerosis has been found alone only in 52% of patients in this selected population. Our work shows that a kidney biopsy should be performed in any young patient suffering from malignant hypertension in order to highlight specific pathological features that may be considered as surrogate markers of the underlying renal disease. Indeed, the kidney biopsy led to the discovery of a superimposed renal disease in more than 24% of all cases. In this cohort, elevated creatininemia at the time of admission, fibrosis > 30% and thrombotic microangiopathy represents the only marker predictive of End Stage Renal Disease.

**Compliance with ethics regulations:** Yes in clinical research.

### FC-140 Kinetic glomerular filtration rate equations in patients with shock: comparison to the iohexol-based gold standard method

#### Maxime Desgrouas^1,2^, Hamid Merdji^3^, Anne Bretagnol^1^, Chantal Barrin-Le Guellec^2^, Jean-Michel Halimi^2^, Stephan Ehrmann^2^, Charlotte Salmon Gandonnière^2^

##### ^1^Centre Hospitalier Régional d’Orléans, Orléans, France; ^2^Centre Hospitalier Universitaire de Tours, Tours, France; ^3^Hôpitaux Universitaires de Strasbourg, Strasbourg, France

**Correspondence:** Maxime Desgrouas - maxime.desgrouas@orange.fr

*Annals of Intensive Care* 2021, **11(Suppl 1):**FC-140

**Rationale:** Static glomerular filtration rate (GFR) formulas are not suitable for critically ill patients because of non–steady-state GFR and variation in the volume of distribution. Kinetic GFR formulas remain to be evaluated against a gold standard. We assessed the most accurate kinetic GFR formula as compared with iohexol clearance among patients with shock.

**Patients and methods/materials and methods:** We conducted a retrospective multicentric study in three French intensive care units in tertiary teaching hospitals in 57 patients within the first 12 h of shock. On day one, we compared kinetic GFR formulas to iohexol clearance, with or without creatinine-concentration correction according to changes in volume of distribution and ideal body weight. We analyzed 3 static GFR formulas (Cockcroft and Gault, Modification of Diet in Renal Disease, Chronic Kidney Disease–Epidemiology Collaboration), urinary creatinine clearance, and 7 kinetic GFR formulas (Jelliffe, Chen, Chiou and Hsu, Moran and Myers, Yashiro, Seelhamer, Brater).

**Results:** We evaluated 33 variants of these formulas after applying corrective factors. The bias ranged from 12 to 47 mL/min/1.73 m^2^. Only the Yashiro equation had a lower bias than urinary creatinine clearance before applying corrective factors (15 vs 20 mL/min/1.73 m^2^). The corrected Moran and Myers formula had the best mean bias, 12 mL/min/1.73m^2^, but wide limits of agreement (−50; 73). The corrected Moran and Myers value was within 30% of iohexol clearance-measured GFR for 27 (47.4%) patients and was within 10% for 9 (15.8%); other formulas showed even worse accuracy.

**Conclusion:** Kinetic GFR equations are not accurate enough for GFR estimation in the first hours of shock. They can both under- or overestimate GFR. Applying corrective factors to creatinine concentration or volume of distribution did not improve accuracy sufficiently to make these formulas reliable. Clinicians should not use kinetic GFR equations to estimate GFR in patients with shock.

**Compliance with ethics regulations:** Yes in clinical research.

### FC-141 Incidence and risk factors of acute kidney injury in diabetic ketoacidosis

#### Jihene Guissouma, Hana Ben Ali, Hatem Ghadhoune, Ghada Abdou, Habib Brahmi

##### Hopital universitaire Habib Bougatfa, Bizerte, Tunisie

**Correspondence:** Jihene Guissouma - guissouma.jihene@gmail.com

*Annals of Intensive Care* 2021, **11(Suppl 1):**FC-141

**Rationale:** Acute kidney injury (AKI) is a common metabolic complication of the Diabetic ketoacidosis (DKA). Few studies have reported the incidence and characteristics of AKI in DKA. The aim of our study was to describe the epidemiologic, clinical and biological features of patients admitted for DKA in intensive care unit (ICU) in order to deduce the risk factors of developing AKI.

**Patients and methods/materials and methods:** A 7-year (2014–2020) retrospective analytic observational single-center study including all patients admitted for DKA. The KDIGO classification was adopted for the definition and classification of AKI. Statistical analysis was performed using the statistic software SPSS 20.

**Results:** Among 62 patients included, 32 had developed AKI on admission. According to KDIGO criteria patients were staged class I (13 cases), II (9 cases) or III (10 cases). The median age was 42 ± 15 years with a female predominance (sex ratio 0.77). The most common type of diabetes was type 1 (50%), followed by type 2 (31%) then inaugural diabetes (19%). The mean duration of diabetes was 10 ± 12 years. The most common comorbidity was hypertension (28%). The mean duration of symptoms before hospitalization was 6 days. The mean capillary blood glucose measurement was 5.86 ± 1.68 g. The mean IGS II and APACHE II were 33 ± 18 and 17 ± 10, respectively. On admission, 14 patients (22%) had shock. The mean levels of pH and bicarbonates were 7.13 ± 0.15 and 7 ± 4, respectively. Infection was the predominant precipitating factor of DKA (41%). The average of length of ICU stay was 8 days. The symptoms resolved after rehydration, insulin therapy, and antibiotics with a mean duration for the normalization of renal function of 3 ± 3 days. Only 16% required hemodialysis. Mortality rate was 16% and the onset of AKI did not have an impact on prognosis. In univariate analysis: the duration of diabetes (*p* = 0.02), hypertension (*p* = 0.027), SAPS II (*p* = 0.001), APACHE II (*p* < 10–3), high capillary blood glucose (*p* < 10–3) and shock (*p* = 0.03) were all predictors of AKI in our study. Besides; Only SAPS II (*p* = 0.003) and APACHE II (*p* = 0.002) were the independent risk factors in multivariate analysis.

**Conclusion:** Although the occurrence of AKI during DKA was frequent in our study, it resolved within a few days and did not worsen the prognosis. The duration of diabetes, co-morbidities, high glucose levels, and severity of the initial clinical presentation (severity scores and shock) were significantly associated to AKI. These results need to be confirmed by further larger studies.

**Compliance with ethics regulations:** Yes in clinical research.

### FC-142 High prevalence and poor prognosis of proximal tubular dysfunction in patients with COVID-19-related acute respiratory distress syndrome

#### Mickaël Bobot^1,2,6^, José Boucraut^4^, Howard Max^1^, Pierre Simeone^5^, Claire Stein^1^, Lionel Velly^5^, Nicolas Bruder^5^, Jean-Marie Forel^1^, Sami Hraiech^1^, Julien Carvelli^3^, Marc Gainnier^3^, Xavier Heim^4^, Jean-Louis Mège^4^, Noémie Jourde-Chiche^2,6^, Laurent Papazian^1^, Stéphane Burtey^2,6^

##### ^1^Service de Médecine Intensive Réanimation DRIS - Hôpital Nord - Assistance Publique Hôpitaux de Marseille, Marseille, France; ^2^Service de Néphrologie - Hôpital La Conception - Assistance Publique Hôpitaux de Marseille, Marseille, France; ^3^Service de Réanimation et Surveillance Continue - Hôpital La Timone - Assistance Publique Hôpitaux de Marseille, Marseille, France; ^4^Laboratoire d’Immunologie - Hôpital La Conception - Assistance Publique Hôpitaux de Marseille, Marseille, France; ^5^Département d’Anesthésie Réanimation - Hôpital La Timone - Assistance Publique Hôpitaux de Marseille, Marseille, France; ^6^C2VN, Aix Marseille Université, INSERM 1263, INRAE 1260, Marseille, France

**Correspondence:** Mickaël Bobot - mickael.bobot@ap-hm.fr

*Annals of Intensive Care* 2021, **11(Suppl 1):**FC-142

**Rationale:** Proteinuria and acute kidney injury (AKI) are frequent in patients with COVID-19 and associated with poor outcomes. Our objective was to measure markers of proximal tubular dysfunction in patients with acute respiratory distress syndrome (ARDS).

**Patients and methods/materials and methods:** We performed a detailed analysis of urinary markers of kidney dysfunction with urinary protein electrophoresis (UPE) in a prospective cohort of consecutive patients admitted to the Intensive Care Unit (ICU) for COVID-19-related ARDS at the onset of mechanical ventilation. We explored 55 consecutive patients admitted to an ICU for COVID-19-related ARDS.

**Results:** All patients had moderate-to-severe ARDS. No patient had a history of chronic kidney disease. Obesity was the most frequent medical condition. Only 2 patients had received a nephrotoxic medication. Median serum creatinine at the onset of mechanical ventilation was 84.5 µmol/L and only 12 (21.8%) patients displayed AKI. Serum uric acid level was low in 47.5% of patients, and 47 (96.2%) patients had glycosuria while only 15 (27.3%) were diabetic. Microscopic haematuria was detected in 50 (90.9%) patients. Median urinary protein/creatinine and albumin/creatinine were 576 and 68 mg/g, respectively. UPE showed: a tubular profile in 96% of patients, with a superimposed glomerular profile in 17% of patients. No patient displayed a solely glomerular injury. Analysis of urinary proteins showed a massive leakage of alpha-1-microglobulin (a1m, 97.1% of patients), beta-2-microglobulin (b2m, 95.8% of patients), free light chains lambda and kappa (FLCK) (respectively, 96.9% and 100%) and Vitamin-D-Binding-Protein (87.5% of patients). After a median follow-up in the ICU of 24 days, 14 (25.5%) patients had died, 32 (58.2%) had recovered, and 9 were still in critical condition; 28 (50.9%) had developed AKI (KDIGO stage 3 in 10, requiring dialysis in 3). Patients developing AKI had higher baseline serum urea (11.3 ± 1.1 vs 7.2 ± 0.6 mmol/L, *p* = 0.005), serum creatinine (132.9 ± 16.7 vs. 64.3 ± 4.1 µmol/L, *p* = 0.001) and serum phosphate (1.10 ± 0.09 vs. 0.88 ± 0.07 mmol/L, *p* = 0.03) levels and a1m/creatinine (140 ± 16 vs. 90 ± 18 mg/g, *p* = 0.05) ratio, and lower eGFR (64 ± 6 vs. 100 ± 4 mL/min/1.73 m^2^, *p* = 0.001) and serum bicarbonate (21.1 ± 0.6 vs. 23.7 ± 0.6, *p* = 0.003) at baseline. a1m/creatinine, b2m/creatinine and FLCK/creatinine ratios were higher in patients who died than in patients who recovered (*p* = 0.007, *p* = 0.005 and *p* = 0.02, respectively).

**Conclusion:** Patients who develop respiratory failure from COVID-19 nearly uniformly display proximal tubular dysfunction, which precedes clinical AKI. Proximal tubular damage seems an important mechanism of COVID-19-induced nephropathy and could be a marker of disease severity.

**Compliance with ethics regulations:** Yes in clinical research.
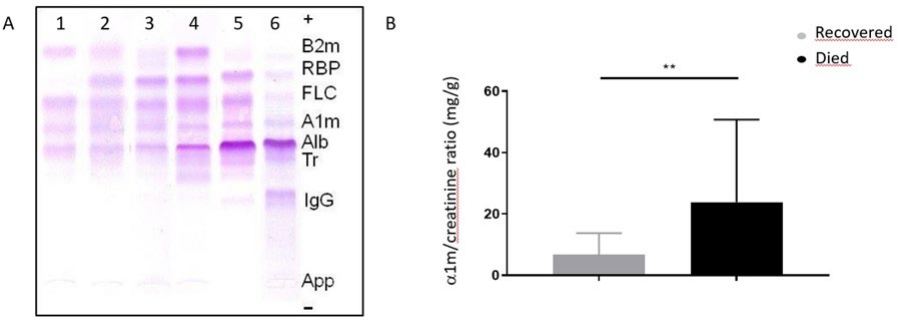


Proximal tubular dysfunction in patients with COVID-19 and ARDS. **A** Illustrative profiles of urinary protein electrophoresis. **B** Association of urinary alpha-1-microglobulin/creatinine ratio with survival in patients

### FC-143 Impact of chloremia on renal prognosis in intensive care unit

#### Matthieu Jamme^1,3^, Julie Boucquemont^3^, Anne Robert^2^, Marie Essig^2,3,4,5^, Guillaume Geri^2,3,4,5^

##### ^1^Centre Hospitalier Poissy Saint Germain en Laye, Poissy, France; ^2^Hopital universitaire Ambroise Paré - APHP, Boulogne Billancourt, France; ^3^Centre de recherche en Epidémiologie et Santé des Populations (CESP)/INSERM, Villejuif, France ^4^Université Paris Saclay, ^5^FHU Sepsis

**Correspondence:** Matthieu Jamme - matthieu.jamme@ght-yvelinesnord.fr

*Annals of Intensive Care* 2021, **11(Suppl 1):**FC-143

**Rationale:** Acute kidney injury (AKI) is one of the most common complications observed in ICU patients. Next to risk factors associated to AKI occurrence related to comorbidities and/or severity at ICU admission, several studies have found a protective renal effect favoring the use of balanced solution. This could have been explained by the deleterious effects of hyperchloremia acidosis induced by a highly concentrated solution in chlorine. We aimed to assess the effect of chloremia on AKI occurrence.

**Patients and methods/materials and methods:** We included all adults patients admitted to a tertiary ICU between June 1st 2006 and June 13th 2019. AKI was defined by stage ≥ 2 according to the KDIGO definition. We assessed the relationship between chloremia and AKI occurrence within ICU stay using the following methodological approaches: Cox cause-specific model using the first chloremia at ICU admission, time-dependant Cox cause-specific model using all chloremia recorded within ICU stay before AKI occurrence and latent class mixed model to identify and compared the different trajectories of chloremia.

**Results:** Our study population included 7464 patients (median age 65[51–76] years, 58.1% of male). At ICU admission, sepsis was observed in 63.2% of cases, acute respiratory failure in 47.2% and shock in 37.5%. AKI occurred in 1.358 (18.2%) patients 4[2–9] days in median after admission. 388 (7.9%) patients died in ICU without AKI. Using first chloremia at ICU admission, hypochloremia (chloremia < 95 mmol/L) and hyperchloremia (chloremia > 110 mmol/L) were both associated with AKI (CSH = 1.40 [1.07–1.09], *p* = 0.01 and CSH = 1.60 [1.09–2.39], *p* = 0.002), respectively). Considering all chloremia before AKI as time dependent covariates, we observed similar results (CSH = 1.86 [1.49–2.33], *p* = 0.02 and CSH = 2.39 [1.75–3.27], *p* < 0.001 for hypo- and hyperchloremia, respectively). Four trajectories of chloremia during ICU was identified: initially low and high increase (class 1, *n* = 55), initially normal and moderate increase (class 2, *n* = 448), initially normal and slow decrease (class 3, *n* = 5256) and initially high and moderate decrease (class 4, *n* = 1705). AKI was more frequently observed in class 1 (20%) and class 2 (14.3%) compared to patients with class 3 (7.8%) and class 4 (10.3%) suggesting a potential renal impact of increasing of chloremia.

**Conclusion:** The present study showed that chloremia is associated with AKI in ICU patients. If both hypochloremia and hyperchloremia at ICU admission were associated with AKI, increasing of chloremia seemed to strongly impact renal outcome within ICU stay.

**Compliance with ethics regulations:** Yes in clinical research.
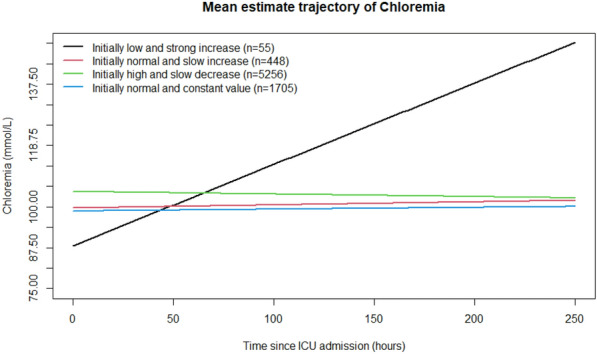


Class of mean chloremia estimate trajectory during ICU stay

### FC-144 Is phosphate monitoring a necessary measure in patients with severe COVID-19?

#### Malek Kharrat, Fatma Essafi, Imen Talik, Rihab Rajah, Moez Kaddour, Takoua Merhebene

##### Faculté de Médecine de Tunis, Tunis, Tunisie

**Correspondence:** Malek Kharrat - kharratmalek1@gmail.com

*Annals of Intensive Care* 2021, **11(Suppl 1):**FC-144

**Rationale:** Inorganic phosphate is important to multiple key physiological processes. Several studies have shown that either hypo or hyperphosphatemia are associated with increased morbidity or mortality in intensive care units (ICU). Recently, phosphate homeostasis disturbance was reported in severe COVID-19 patients. To assess the benefit of monitoring phosphate’s level in severe COVID-19 patients.

**Patients and methods/materials and methods:** This study was performed at the Zaghouan’s Hospital ICU, a 10-bed tertiary ICU in Tunisia. It was a retrospective study enrolled between 24th March and 31st December 2020. All COVID-19 patients in whom serum phosphate was measured at least once during their hospital stay were included. Patients were divided to 4 groups: G1: normophosphatemia [0.85–1.45] mmol/l, G2: hypophosphatemia, G3: hyperphosphatemia, and G4: mixed group showing both hypo and hyperphosphatemia. Patients who were hospitalized for less than 24 h are excluded. Phosphatemia was daily strictly monitored.

**Results:** Seventy-five patients were enrolled (median age was 63 ± years with a sex ratio of 2.12) with 569 phosphate measurements. 34(45.3%) had at least 1 episode of hypophosphatemia, 17(22.7%) had at least 1 episode of hyperphosphatemia and 14(18.7%) had showed episodes of mixed hypo and hyperphosphatemia. Demographic characteristics, co-morbidities and nutritional status were comparable between the 4 groups. All patients received steroids, vitamin D supplementation (2000 UI) and parenteral feeding. Hypophosphatemia was earlier onset than hyperphosphatemia (3.3 ± 2.5 day vs 8 ± 5.6 day). Patients who had developed hyperphosphatemia or mixed disorder have required more frequently invasive mechanical ventilation (82%, *p* = 0.001) with a difficult weaning in comparison of others groups. Those patients had also showed more healthcare-associated pneumonia (59%, *p* = 0.006), acute kidney failure (44%, *p* = 0.00001) and septic shock (40%, *p* = 0.001). The normophosphatemic group showed the lowest ICU length of stay with a median of 7 ± 3.26 days compared to the mixed group (8.5 ± 4.3), the hypophosphatemic group (11 ± 4.7) and hyperphosphatemic group (14 ± 6.6). ICU mortality was significantly higher in patients with hyperphosphatemia: 82% vs 30% in normophosphatemia group.

**Conclusion:** Phosphate disorder in severe COVID-19 patients was associated with poorer clinical outcomes and showed significant impact on ICU management. Therefore, phosphate measurement can be adopted as a severity marker in COVID-19 critically ill.

**Compliance with ethics regulations:** Yes in clinical research.

### FC-145 Hypophosphatemia in critically ill adults: Prevalence and associated risk factors

#### Malek Kharrat, Fatma Essafi, Rihab Rajah, Khaoula Ben Isamail, Moez Kaddour, Takoua Merhabene

##### Faculté de Médecine de Tunis, service de Réanimation Médicale Hôpital Régional de Zaghouan, Tunis, Tunisie

**Correspondence:** Malek Kharrat - kharratmalek1@gmail.com

*Annals of Intensive Care* 2021, **11(Suppl 1):**FC-145

**Rationale:** Hypophosphatemia is a metabolic disorder leading to serious complications. It occurs generally in Intensive Care Units (ICUs) and can be associated with poor prognosis. To identify prevalence and associated risk factors of hypophosphatemia in ICU and to investigate whether it is associated with higher mortality.

**Patients and methods/materials and methods:** A single-center retrospective study of all patients admitted to the Zaghouan’s ICU over the period of 1 year (Jan–Dec 2020), regardless of the reason for admission, in whom serum phosphate level was measured at least once during their ICU stay. Are excluded from the study patients who were hospitalized for less than 24 h. Patients were divided into 2 groups: G1 = hypophosphatemic group (patients with at least one phosphate value < 0.85 mmol/l) and G2 = normophosphatemic group: patients with explicitly normal phosphate values (0.85 < *P* < 1.45 mmol/l).

**Results:** A total of 300 adults patients were admitted to our ICU during the study period. One hundred and thirty-one patients (43%) met the inclusion criteria with a sex ratio 1.78 and a median age 57 ± 16 years old. The main reason for ICU admission was respiratory distress (78%). The phosphate median level measured in the first day was 0.97 ± 0.38 mmol/l. Eighty patients (61.1%) had developed at least one episode of hypophosphatemia during their hospital stay, median level was 0.69 ± 0.13. Hypophosphatemeia was noted in 30% of cases at ICU admission. Median of occurrence was at the third day after admission [1–22]. In multivariate analysis, independent risk factors of hypophosphatemia were: acute respiratory failure ((OR = 4.5, 95% CI 1.10–9.33, *p* = 0.05), parenteral feeding (OR = 3.2, 95% CI 0.25–2.51, *p* = 0.033), use of corticosteroids (OR = 2.6, 95% CI 0.14–1.2, *p* = 0.039) and vasopressors agents (OR = 3.08, 95% CI 0.18–1.90, *p* = 0.001). Significantly higher invasive mechanical ventilation was applied for patients in the hypophosphatemia group (53.8% vs 35.3%; *p* = 0.039) and it was also more often associated with more often weaning failure (47.5% vs 25.5%; *p* = 0.012). Patients with hypophosphatemia had showed more healthcare associated pneumonia (36.3% vs7.8% *p* < 0.05) and longer ICU stay 11 ± 6 vs 6.6 ± 4 day (*p* < 0.05). In comparison with normophosphatemic patients, hypophosphatemia was significantly associated with an increased risk of ICU-in-hospital mortality 31.7% vs 16.1% (OR = 1.94,95% CI 0.34–1.50, *p* < 0.05).

**Conclusion:** hypophosphatemia is a frequent and early metabolic disorder in critically ill adults. Identified risk factors were acute respiratory disease, parenteral feeding, use of vasopressors and corticosteroids. Phosphate monitoring seems to be necessary since it appears to be associated with poorer clinical outcome.

**Compliance with ethics regulations:** Yes in clinical research.

### FC-146 Severe hemoptysis: etiology, management and outcomes

#### Julien Dessajan, Antoine Parrot, Aude Gibelin, Vincent Labbe, Clarisse Blayau, Michel Djibre, Guillaume Voiriot, Muriel Fartoukh

##### Hôpital Tenon - APHP, Paris, Paris, France

**Correspondence:** Julien Dessajan - julien.dessajan@aphp.fr

*Annals of Intensive Care* 2021, **11(Suppl 1):**FC-146

**Rationale:** Limited data are available concerning severe hemoptysis (SH) in the intensive care unit (ICU). A French retrospective study identified the factors independently associated with in-hospital mortality, developed and validated a prognosis score in a cohort of patients from 1995 to 2009 (1). We aimed at describing the clinical characteristics, etiologies, management and outcomes of adult patients with SH admitted to our center since 2009, and at comparing their in-hospital mortality to that of the historical cohort (1995–2009).

**Patients and methods/materials and methods:** A monocentric analysis of a prospective registry of consecutive patients with SH admitted to the ICU of a tertiary referral center between March 2009 and March 2019.

**Results:** During the study period, 945 patients (75% males; age 53 ± 17 years) were included, 84% of whom had a performance status (PS) ≤ 1. The patients had numerous comorbid conditions, particularly respiratory (67%). On ICU admission, the mean volume of bleeding was 215 ± 184 ml, and 13% of patients required mechanical ventilation within the first 24 h. Lung cancer was the first cause of SH, followed by bronchiectasis, tuberculosis, pneumonia and aspergillosis (Fig. 1). Episodes of hemoptysis related to pneumonia had increased, as compared with the historical cohort (11% vs. 5%; *p* < 0.001). The pulmonary artery was involved in 110 cases (12%), mainly during pneumonia (23%), cancer or aspergillosis (20% for both). Noteworthy, the pulmonary arterial mechanism had increased, as compared with the historical cohort (12% vs. 5%; *p* < 0.001). Altogether, a procedure of vascular interventional radiology was performed in 81% of patients, allowing immediate bleeding control in more than 75% of cases, while emergency surgery was very rarely required (0.9%) for bleeding control. Finally, the in-hospital mortality rate had marginally increased, as compared to that of the historical cohort (8.7% vs. 6.5%; *p* = 0.08). Independent factors of in-hospital mortality were tobacco use, PS ≥ 1, infiltrates of ≥ 2 quadrants on the admission radiograph, mechanical ventilation within the first 24 h, and lung cancer or aspergillosis as the cause of bleeding (area under the ROC curve at 0.90).

**Conclusion:** During the past decade, the etiologies of SH have changed, lung cancer being the leading cause, and non-tuberculous pneumonia having an increasing role. The most frequent involvement of the pulmonary artery vasculature might be the result of these two latter etiologies. In-hospital mortality has also slightly increased, mainly due to the greater proportion of cancer-related episodes, as compared to that of the historical cohort.

**Reference**

1. Fartoukh M, Khoshnood B, Parrot A, Khalil A, Carette M-F, Stoclin A, et al. Early prediction of in-hospital mortality of patients with hemoptysis: an approach to defining severe hemoptysis. Respiration 2012;83:106–14. 10.1159/000331501.

**Compliance with ethics regulations:** Yes in clinical research.
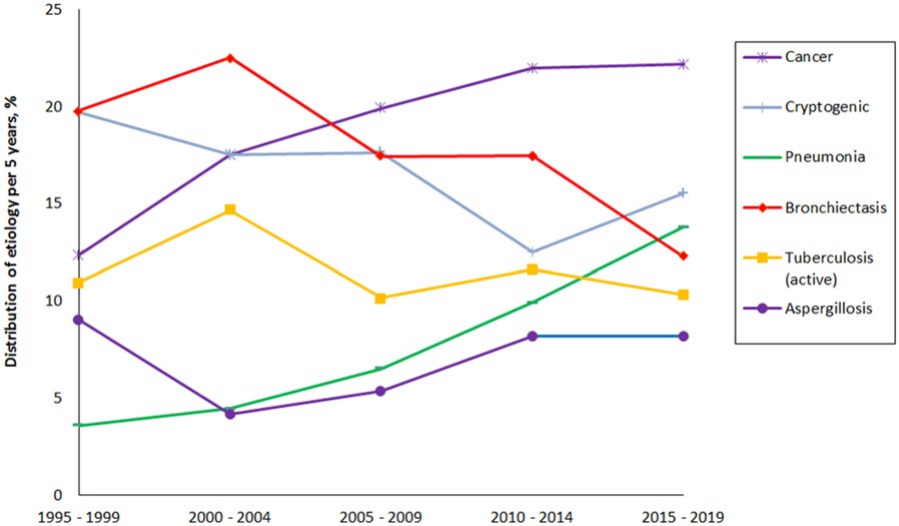


Fig. 1 Main etiologies of SH over a 24-year period (1995–2019)

### FC-147 Acute severe asthma: changes in patient characteristics, management, and outcomes over a period of 20 years (1997 to 2017), insights from the Cub-Réa database

#### Romy Younan^1^, Jean Loup Augy^1^, Bertrand Hermann^1^, Bertrand Guidet^2^, Philippe Aegerter^3^, Emmanuel Guerot^1^, Ana Novara^1^, Caroline Hauw-Berlemont^1^, Amer Hamdan^1^, Clotilde Bailleul^4^, Francesca Santi^1^, Jean Luc Diehl^1^, Nicolas Peron^1^, Nadia Aissaoui^1^

##### ^1^Hopital Européen Georges Pompidou, Paris, France; ^2^Hopital Saint Antoine, Paris, France; ^3^Université de Versailles Saint-Quentin-en-Yvelines, Versailles, France; ^4^Hopital Nord, Marseille, France

**Correspondence:** Romy Younan - romy_younan@hotmail.com

*Annals of Intensive Care* 2021, **11(Suppl 1):**FC-147

**Rationale:** While acute severe asthma (ASA) is the leading cause of emergency department visits and the third cause of hospitalization in children younger than 18 years old, there is a lack of data regarding adult patients admitted in intensive care units (ICU) for ASA. We aimed to describe the evolutions in epidemiology, management and outcomes of ASA, over a period of 20 years in the Greater Paris area ICUs (CUB-Réa Database).

**Patients and methods/materials and methods:** Demographics, severity and supportive treatments were collected. The primary endpoint was the prevalence of ASA by time periods, divided into four periods of 5 years. The secondary endpoints were ICU survival, hospital survival, the use of mechanical ventilation including non-invasive and invasive, renal replacement therapy (RRT) and catecholamine. Multivariable analysis was performed to assess predictors of ICU mortality.

**Results:** Of the 475 357 ICU admissions from January first, 1997 to January first, 2016, 7049 (median age 46 years old and 55% of female) were for ASA with a decreasing prevalence over time, respectively 2.8%, 1.7%, 1.1%, and 1.1% of total ICU admissions (*p* < 0.001). The baseline characteristics are reported in Table 1. The mean age was 46 years old [32–59], 3906 (55%) were female, the median SAPS II was 20 [13–28], and 1501 (21%) had mechanical ventilation. Over time, age increased as well as the SAPSII and the number of comorbidities. The use of invasive and non-invasive mechanical ventilation also increased over time, respectively, 13%, 21%, 34% and 28%, whereas the use of catecholamine decreased (*p* < 0.001). The use of RRT was low and remained unchanged over the period study. Interestingly, the median ICU length of stay increased from 2 to 6 days, (*p* < 0.001). The intensive care unit (ICU) survival rate improved from, respectively, 97% to 99% (*p* = 0.008). The predictors of ICU-Mortality were SAPSII (OR 1.06 [1.05, 1.07]), RRT (OR 6.78 [2.79, 16.49]), catecholamine (OR 2.6 [1.52, 4.47]), cardiac arrest (OR 13.8 [7.28, 26.14]), pneumothorax (OR 2.55 [0.19, 33.73], ARDS (OR 2.56 [0.73, 8.98]), sepsis (OR 5.15 [1.57, 16.86]) and IMV (OR 3.86 [2.18, 6.84]).

**Conclusion:** The incidence of adult ICU admission for ASA remains uncommon. Over the past two decades, despite an increasing severity of patients and the use of mechanical ventilation, the ICU survival rate is high and improved.

**Compliance with ethics regulations:** Yes in clinical research.
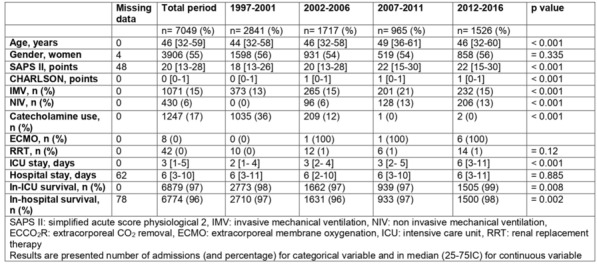


Table 1 Trends in demographic characteristics, management and outcome of ICU adults admitted for ASA according to period study

### FC-148 Acute severe asthma requiring invasive mechanical ventilation in the era of modern resuscitation techniques: a 10-year bicentric retrospective study

#### Adeline Grateau^1^, Antoine Binachon^1^, Nicolas Allou^1^, Cyril Ferdynus^1^, Jérôme Allyn^1^, Laurence Dangers^1^, Olivier Martinet^1^, Véronique Boisson^2^, Alexandre Gauthier^2^, Julien Jabot^1^, Romain Persichini^1^

##### ^1^CHU Félix Guyon, Saint-Denis, France; ^2^CHU Sud Réunion, Saint-Pierre, France

**Correspondence:** Adeline Grateau - ad.grateau@hotmail.fr

*Annals of Intensive Care* 2021, **11(Suppl 1):**FC-148

**Rationale:** Patients with acute severe asthma (ASA) may in rare cases require invasive mechanical ventilation (IMV). However, recent data on this issue are lacking.

**Patients and methods/materials and methods:** In this retrospective and bicentric study conducted on a 10 year period, we investigate the in-hospital mortality in patients with ASA requiring IMV. We compared this mortality to that of patients with other types of respiratory distress using a standardized mortality ratio (SMR) model.

**Results:** Eighty-one episodes of ASA requiring IMV were evaluated. Factors significantly associated with in-hospital mortality were cardiac arrest on day of admission, cardiac arrest as the rea- son for intubation, absence of decompensation risk factors, need for renal replacement ther- apy on day of admission, and intubation in pre-hospital setting. Non-survivors had higher SAPS II, SOFA, creatinine and lactate levels as well as lower blood pressure, pH, and HCO_3−_ on day of admission. In-hospital mortality was 15% (*n* = 12). Compared to a reference population of 2670 patients, the SMR relative to the SAPS II was very low at 0.48 (95% CI, 0.25–0.84). The only factor independently associated with in-hospital mortality was cardiac arrest on day of admission. In-hospital mortality was 69% in patients with cardiac arrest on day of admission and 4% in others (*p* < 0.01). Salvage therapies were given to 7 patients, sometimes in combination with each other: ECMO (*n* = 6), halogenated gas (*n* = 1) and anti-interleukin-5 antibody (*n* = 1). Death occurred in only 2 of these 7 patients, both of whom had cardiac arrest on day of admission.

**Conclusion:** Nowadays, the mortality of patients with ASA requiring IMV is low. Death is due to multi-organ failure, with cardiac arrest on day of admission being the most important risk factor. In patients who did not have cardiac arrest on day of admission the mortality is even lower (4%) which allows an aggressive management.

**Compliance with ethics regulations:** Yes in clinical research

### FC-149 Long-term survival of critically ill patients with lung cancer, a retrospective study

#### Pierre Chaffiotte, Leah Mailly-Giacchetti, Amel Boudjemaa, Antoine Morel, Emmanuelle Mercier, Marie Lecronier, Rusel Leon, Tommaso Maraffi, Jerome Cecchini, Tin-Hinan Mezdad, Frederique Schortgen

##### CHI CRETEIL, Creteil, France

**Correspondence:** Pierre Chaffiotte - pierre.chaffiotte@gmail.com

*Annals of Intensive Care* 2021, **11(Suppl 1):**FC-149

**Rationale:** Prognosis of lung cancer patients is poor; however, targeted therapies represent a significant progress in the treatment of non-small cell lung cancer (NSCLC). Care intensity and ICU admission must be considered in the light of the increased number of patients in whom new treatments are proposed. Whether the prognosis of critically ill lung cancer patients has change remains unknown. The aim of this study was to evaluate the long-term prognosis of ICU patients with a previously diagnosed lung cancer.

**Patients and methods/materials and methods:** We conducted a retrospective analysis of lung cancer patients with unplanned admission in our ICU from January 2017 to December 2019. Patients were identified by hospital discharge codes of lung cancer. Survival was recorded up to 12 months. We reviewed computer-based medical files and called the registry offices to identify the date of death, if necessary. Factors potentially associated with survival were recorded: characteristics and treatments of cancer, performance status, main diagnosis for ICU admission and severity of organ failures. Logistic regression models were constructed to identify significant factors associated with 3 months survival.

**Results:** 107 patients (5% of ICU admissions) were identified during the study period with similar repartition over the 3 years. 105 could be analyzed. Median age was 67 (60–74) years, 87% were NSCLC and 78% had metastasis. The median SAPS II score was 46 (37–60) and 38% received invasive organ support. Overall survival was 63% at ICU discharge, 33% at hospital discharge, 22%, 13% and 9%, at 3, 6 and 12 months, respectively. In multivariate analysis, independent factors associated with 3-months mortality were metastasis OR: 4.4 (CI 95%: 1.1–17.6) and poor performance status (PS 3 or 4) OR 12.6 (CI 95%: 1.5–105.5). The severity of acute disease was not associated with the outcome. Patients who could not receive oncologic treatment after ICU discharge had a significantly higher 3-month mortality (92% vs 29%, *p* < 0.001). Baseline characteristics are presented in the table below.

**Conclusion:** Survival of ICU patients with lung cancer remains particularly poor. Poor performance status and presence of metastasis were the two main factors associated with mortality. These data could help ICU physicians to assess which of these patients could benefit the most from intensive care.

**Compliance with ethics regulations:** Yes in clinical research.
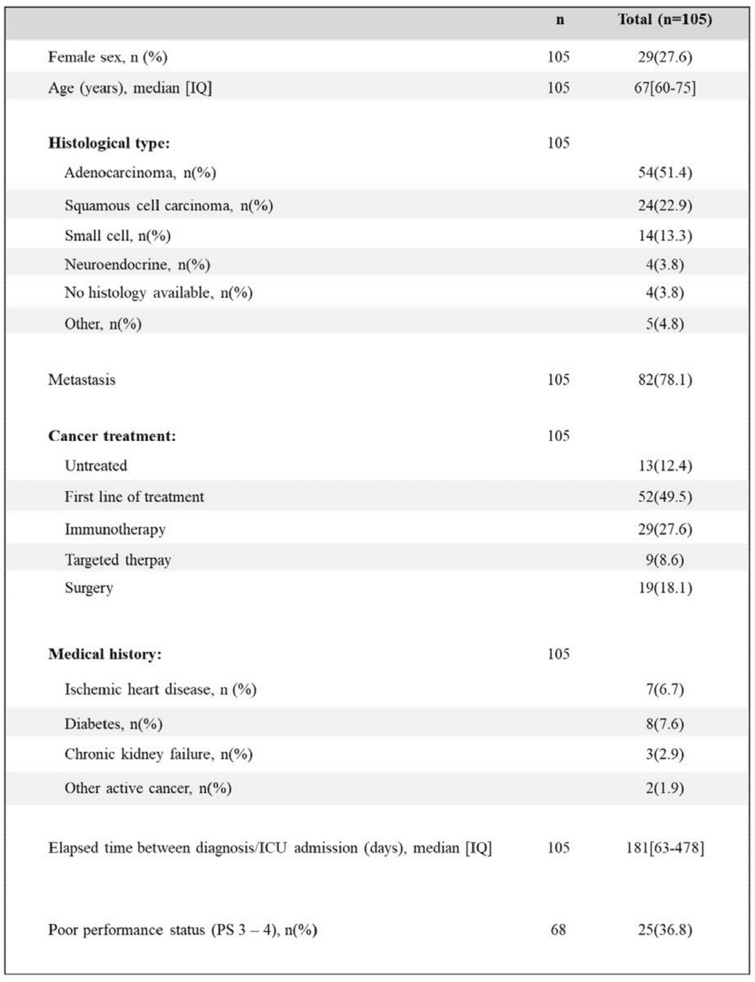


Baseline characteristics of the study population

### FC-150 Characteristics and outcomes of diffuse interstitial pneumonias discovered in ICU: a retrospective monocentric study (The IPIC study)

#### Damien Eckert, Sébastien Moschietto, Pierre Ducq, Lynda Di Vico, Antoine Frouin, Florent Montini

##### Centre hospitalier d’Avignon (Henri Duffaut), Avignon, France

**Correspondence:** Damien Eckert - eckert.damien@gmail.com

*Annals of Intensive Care* 2021, **11(Suppl 1):**FC-150

**Rationale:** Interstitial lung disease (ILD) is a heterogenous group of disorders characterized by an association of inflammatory and fibrotic abnormalities of the lung. An acute respiratory failure (ARF) may be the initial presentation of the disease. This study aims to highlight the diagnosis of ILD in the intensive care unit (ICU) and describe the epidemiologic, prognosis, and scanographic features of patients with ILD diagnosed in our ICU department.

**Patients and methods/materials and methods:** We conducted a single-center retrospective study. We reviewed every ICU admission between October 2017 and February 2020. Over 2547 patients, with 716 being admitted for ARF. Inclusion criteria were principal diagnoses of ILD. Exclusion criteria were other causes of ARF (ARDS, infectious disease, cardiogenic oedema). For each patient, clinical data and lung CT patterns were analyzed.

**Results:** Twenty-six patients were diagnosed with ILD in ICU over the period of study. Most of the patients were men (73%), with a mean age of 65.3 years. The most frequent diagnosis were connective-associated ILD (31%), idiopathic ILD (23%), and lymphangitic carcinomatosis (19%). The mean SOFA score was 4.9. Most of the patients were intubated (81%). The mean ratio of the partial ratio of oxygen (PaO2) to the fraction of inspired oxygen (FIO2) was 165 mmHg. Two patients received veinovenous ECMO, with no benefit of survival. The all-cause mortality rate was 65.4%. With univariate analysis, the mortality rate was lower among the subgroup of connectivity ILD (37.5%) and the subgroup of patients treated with high doses of intravenous corticosteroid (30%). On the other hand, mechanical ventilation was associated with 76.2% of mortality. CT data were available in 25 patients (96.2%). All lung CT were analyzed and lesion patterns are presented by frequency in Fig. 1.

**Conclusion:** We conducted a single-center cohort of patients diagnosed with ILD in the ICU. This rare cause of ARF is associated with a poor outcome in ICU, but connectivity-associated ILD seems to have a better prognosis. Lung high-resolution CT and identification of lesion patterns are the cornerstone of the diagnosis. Improved knowledge of ILD by the intensivist may result in an earlier diagnosis and eventually lead to a better treatment.

**Compliance with ethics regulations:** Yes in clinical research.
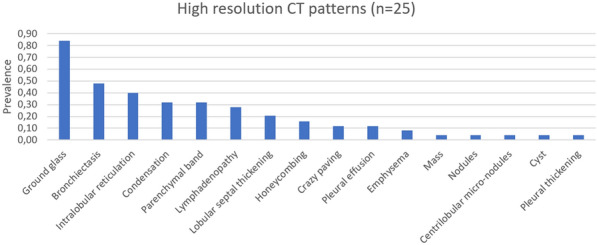


Fig. 1 High resolution CT patterns (*n* = 25)

### FC-151 Outcomes of severe systemic rheumatic disease patients requiring extracorporeal membrane oxygenation

#### Pierre Bay, Guillaume Lebreton, Alexis Mathian, Pierre Demondion, Cyrielle Desnos, Juliette Chommeloux, Guillaume Hékimian, Nicolas Bréchot, Ania Nieszkowska, Matthieu Schmidt, Fleur Cohen, Pascal Leprince, Charles-Edouard Luyt, Zahir Amoura, Alain Combes, Marc Pineton De Chambrun

##### CHU Pitié Salpétrière, Paris 13E Arrondissement, France

**Correspondence:** Pierre Bay - pierrebay53@yahoo.fr

*Annals of Intensive Care* 2021, **11(Suppl 1):**FC-151

**Rationale:** Systemic rheumatic diseases (SRDs) are a group of inflammatory disorders that can require intensive care unit (ICU) [1] admission because of multiorgan involvement with end-organ failure(s), whose may need a VA- or VV-ECMO support. Data focusing on ECMO in SRD patients are scarce and many questions persist. We undertook this study to determine the outcomes and unfavorable outcome-associated factors of severely ill SRD patients requiring ECMO support.

**Patients and methods/materials and methods:** This French monocenter, retrospective study included all SRD patients requiring venovenous (VV)- or venoarterial (VA)-ECMO admitted to a 26-bed ECMO-dedicated ICU from January 2006 to February 2020. The primary endpoint was in-hospital mortality. The secondary outcomes included patient’s characteristics (laboratory findings, in-ICU organ-failure treatment(s), SRD-specific manifestations and treatment(s), complications). Were compared the primary and secondary outcomes for the entire population and in the following subgroups: flare-/infection-related admission and VA/VV-ECMO.

**Results:** Ninety patients (male/female ratio: 0.5; mean age at admission: 41.6 ± 15.2 years) admitted to the ICU received VA/VV-ECMO, respectively, for an SRD-related flare (*n* = 69, *n* = 38/31) or infection (*n* = 21, *n* = 10/11). The flow chart reports patients’ outcomes according to the reason for admission and ECMO hook-up (Fig. 1). SRD was diagnosed in-ICU for 31 (34.4%) patients. In-ICU and in-hospital mortality rates were 48.9% and 51.1%, respectively. Nine patients were bridged to cardiac (*n* = 5) or lung transplantation (*n* = 4), or left ventricular assist device (*n* = 2). The Cox multivariable model retained the following independent predictors of in-hospital mortality: in-ICU SRD diagnosis, day-0 Simplified Acute Physiology Score (SAPS) II score ≥ 70 and arterial lactate ≥ 7.5 mmol/L for VA-ECMO–treated patients; day-0 SAPS II score ≥ 70, ventilator-associated pneumonia and arterial lactate ≥ 7.5 mmol/L for VV-ECMO-treated patients whereas vasculitis independently predicts hospital survey.

**Discussion:** The main analysis considered VA- and VV-ECMO patients jointly. The reasons for ICU admissions and ECMO cannulation, and the characteristics, management and outcomes of these patients obviously differ. However, the analysis aimed to present a comprehensive, real-life picture of ECMO treatment of SRD patients, with separate analyses of VA- and VV-ECMO subgroups thereafter.

**Conclusion:** ECMO support appears to be relevant for critically ill SRD patients, with 49% survival at hospital discharge. Herein, we report the largest series of ECMO-treated, severely ill SRD patients. Vasculitis was independently associated with favorable outcomes of VV-ECMO–treated patients. Further studies are needed to specify the role of ECMO for SRD patients.

**Reference**

1. Larcher R, Pineton de Chambrun M, Garnier F, Rubenstein E, Carr J, Charbit J, et al. One-year outcome of critically ill patients with systemic rheumatic disease: a multicenter cohort study. Chest. 2020.

**Compliance with ethics regulations:** Yes in clinical research.
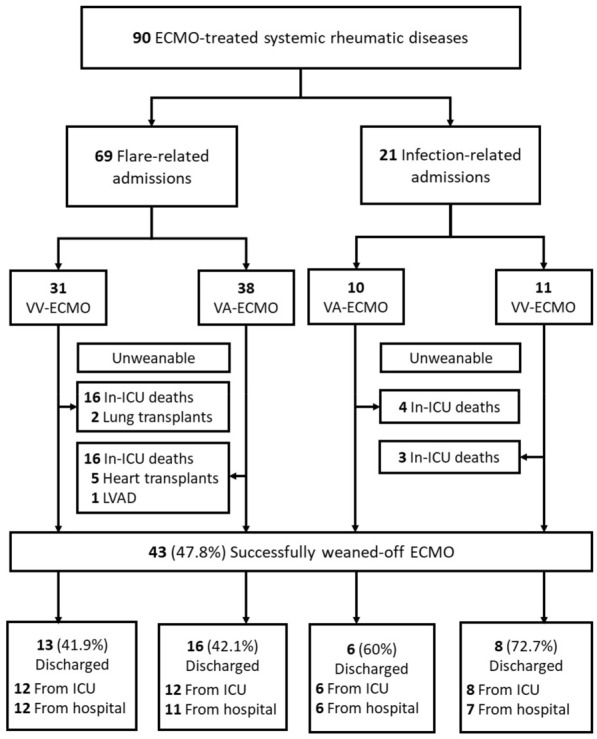


Flow-chart of the 90 patients with systemic rheumatic disease requiring extracorporeal membrane oxygenation

### FC-152 Seawater drowning: clinical features and prognostic factors

#### Jihene Guissouma, Malek Chroufa, Hatem Ghadhoune, Habib Brahmi, Meriam Ksouri, Hana Ben Ali

##### Hopital universitaire Habib Bougatfa, Bizerte, Tunisie

**Correspondence:** Jihene Guissouma - guissouma.jihene@gmail.com

*Annals of Intensive Care* 2021, **11(Suppl 1):**FC-152

**Rationale:** Drowning is a major health problem worldwide and especially in low-income countries. The most serious cases are admitted in intensive care unit and often require mechanical ventilation. The aim of our study was to describe the epidemiologic and clinical features of seawater drowning victims, the management and the evolution in the intensive care unit in order to deduce the independent prognostic factors of mortality.

**Patients and methods/materials and methods:** A 5-year (2016 to 2020) retrospective analytic observational single-center study including all victims of seawater-drowning admitted in a 6-bed intensive care unit. Statistical analysis was performed using the statistic software SPSS 23.

**Results:** Forty patients were enrolled. Mean age was 29 ± 17 years with sex ratio of 2,3. The main cause of drowning was exhaustion (38%) followed by current waves (32%), medical cause (20%), fall (5%) and suicide attempt (5%). Mean submersion time was 4.8 ± 4 min and mean time to first resuscitation was 15 ± 10 min. More than half of patients arrived to the emergency at an advanced stage of drowning (severe hypoxia-anoxia and stage 5 or 6 according to the szpilmann classification). Mean SAPS II and APACHE II scores were 35 ± 21 and 15 ± 10, respectively. On admission, 50% of all patients were comatose and 27% shocked. The main biological anomalies were hyperleukocytosis, hypernatremia, metabolic acidosis and rhabdomyolysis. Mean PaO_2_/FiO_2_ was 177 ± 113 mmHg and 60% of all cases required invasive mechanical ventilation. The mean length of stay was 7 days and the mortality rate was 20%. In univariate analysis: submersion time (*p* < 10^−3^), stage of drowning (*p* = 0.01), SAPS II (*p* < 10^−3^), APACHE II (*p* = 10^−3^), Glasgow Coma Scale (*p* = 0.002), severe hypoxemia (*p* = 0.025), metabolic acidosis (*p* < 10^−3^), and rhabdomyolysis (*p* < 10^−3^) were all predictors of mortality. Besides; submersion time (*p* = 0.003), severe hypoxemia (*p* = 0.025) and rhabdomyolysis (*p* < 10^−3^) were the independent prognostic factors in multivariate analysis.

**Conclusion:** Even though our study is limited by its retrospective design and the small number of patients; drowning outcome was closely related to the submersion time and the severity of the initial clinical presentation. Further researches are needed to confirm these results.

**Compliance with ethics regulations:** Yes in clinical research.

### FC-153 Characteristics and outcome of severe forms of COVID-19 treated in intensive care unit

#### Jihene Guissouma, Hana Ben Ali, Hatem Ghadhoune, Malek Kharrat, Habib Brahmi, Mariem Ksouri

##### Hopital universitaire Habib Bougatfa, Bizerte, Tunisie

**Correspondence:** Jihene Guissouma - guissouma.jihene@gmail.com

*Annals of Intensive Care* 2021, **11(Suppl 1):**FC-153

**Rationale:** Coronavirus disease 2019 (COVID-19) emerged first in December 2019 in China then it spread all over the world. The severity of clinical presentation varies from mild to critical cases requiring intensive care unit (ICU) hospitalization. The aim of this study was to describe the characteristics, the management, and the outcome of severe forms of COVID-19 treated in ICU.

**Patients and methods/materials and methods:** A 5 month (Sep 2020–Jan 2021) prospective descriptive single-center study including all severe forms of COVID-19 in a 6-bed ICU. Statistical analysis was performed using SPSS 20.

**Results:** Forty patients were included. Mean age was 62 ± 12 years with male predominance (sex ratio = 3). The most common comorbidities were obesity (57%), hypertension (50%) and diabetes (40%). Three patients had regular oral steroid with immunosuppressor treatment. The mean symptom duration before hospitalization was 5 days. The mean length of stay in various units (emergencies or COVID departments) prior to ICU admission was 5 days. The most common symptoms reported were shortness of breath (95%), fever (82%), flu syndrome (77%), cough (45%) and digestive symptoms (20%). The mean SAPS II and APACHE II were 32 ± 15 and 14 ± 6, respectively. The mean PaO_2_/FiO_2_ ratio was 150 ± 90 mmHg (mild, moderate and severe ARDS in 5, 19 and 16 cases, respectively). Lymphopenia was found in 67% of all cases. Chest computed tomography scan conducted for 31 patients (77%) showed bilateral peripheral multifocal ground glass opacities. The mean lung damage was 60 ± 20%. High-Flow Nasal Cannula oxygenation and noninvasive positive pressure ventilation (NIV) were prescribed in 2 and 27 cases, respectively. Invasive ventilation (IV) was initiated on admission in 11 cases and after NIV failure in 16 cases. The mean duration of IV was 12 ± 10 days, respectively. Prone positioning was done for 15 patients (11 with IV and 4 with NIV). Empiric antibiotic’s therapy was initiated upon admission in 75% of patients. All patients received curative doses of anticoagulant, steroids and multi-vitamins. Only three received monoclonal antibodies. Evolutionary complications occurred in 80% of cases. The most common were septic shock (47%) and healthcare associated pneumonia (40%). Thromboembolic complications were noted in 5 cases and hemorrhagic complications in 10 cases. Fourteen patients developed acute kidney injury and five required hemodialysis. The mean length of stay in the ICU was 14 ± 10 days. Mortality rate was extremely high reaching 82.5%.

**Conclusion:** Despite an active management of severe forms of COVID-19; evolutionary complications along hospitalization occurred and worsened the outcome with a high mortality rate in ICU.

**Compliance with ethics regulations:** Yes in clinical research.

### FC-154 Risk factors of mortality among COVID-19 patients admitted to intensive care unit: a retrospective cohort study

#### Iliass Ennour Idrissi, Taha Hounain, Youssef El Ouardi, Amina El Khayari, Mohammed El Khallouki

CHU mohamed VI marrakech, Marrakech, Maroc

**Correspondence:** Iliass Ennour Idrissi - ennouridrissiiliass@gmail.com

*Annals of Intensive Care* 2021, **11(Suppl 1):**FC-154

**Rationale:** Intensive care unit admission and mortality of coronavirus disease 2019 (COVID-19) exhibit spatial and temporal variability. Understanding risk factors for severe disease and mortality according to the context of care is critical for the management of patients admitted to intensive care units. In the present study, the aim was to evaluate the fatality rate and predictors of mortality among COVID-19 patients admitted to the intensive care unit of a major urban city during a critical phase of the pandemic.

**Patients and methods/materials and methods:** We conducted a preliminary retrospective study of consecutive patients admitted to the intensive care unit between August and October 2020 of a dedicated COVID-19 medical facility. Socio-demographic, clinical, biological and follow-up data were extracted from medical records. Cox proportional hazard models were used to estimate associations between risk factors measured at time of admission and mortality.

**Results:** During the study period, 59 patients were admitted to the intensive care unit for PCR confirmed (84.7%) or clinically suspected (15.3%) COVID-19 infection. Mean age of patients was 62.2 ± 14.1 years (range: 30.0–94.0 years) and 71.7% were males. The mean duration of symptoms before admission to the intensive care unit was 8.0 ± 3.0 days (median = 8 days; range 3–15 days). The overall case fatality rate was 52.5% and the median intensive care unit stay length of stay was 3.5 days (range 0–44 days). In univariate analyses, age (HR = 1.06 [1.02–1.09], *p* = 0.0017), history of chronic obstructive pulmonary disease (HR = 0.12 [0.03–0.44], *p* = 0.0013), history of cardiac disease (HR = 0.32 [0.11–0.96], *p* = 0.0416), respiratory rate (HR = 1.10 [1.01–1.20], *p* = 0.0237), heart rate (HR = 1.04 [1.01–1.08], *p* = 0.0058), white blood cells count (HR = 1.15 [1.07–1.25], *p* = 0.0003), neutrophils count (HR = 1.19 [1.09–1.30], *p* = 0.0001), D-dimers (HR = 1.04 [1.00–1.08], *p* = 0.0347), oxygen saturation (HR = 0.94 [0.90–0.97], *p* = 0.0007), and spontaneous ventilation with inspiratory support and positive expiratory pressure (VS AI PEP) (HR = 0.38 [0.17–0.86], *p* = 0.0200) were associated with mortality rate. In multivariate analyses, only neutrophils count (HR = 1.39 [1.07–1.80], *p* = 0.0136) was associated with mortality rate.

**Conclusion:** The overall case fatality rate in intensive care units is very high. While various clinical factors measured at time of admission may have indicated a worse prognosis, only neutrophils count was an independent predictor of mortality in our patients. These results indicate that patients’ admission in intensive care units occurs late in the course of the disease evolution and that some degree of secondary bacterial infection at the time of admission might be involved in the observed high mortality rate.

**Compliance with ethics regulations:** Yes in clinical research.

### FC-155 Postextubation laryngeal edema in severe COVID-19

#### Georges Abi Abdallah, Alexis Ferre, Antoine Gros, Christelle Simon, Fabrice Bruneel, Stéphanie Marque Juillet, Stéphane Legriel, Marine Paul

##### centre hospitalier de versailles, Le Chesnay, France

**Correspondence:** Marine Paul - marine.1604@hotmail.fr

*Annals of Intensive Care* 2021, **11(Suppl 1):**FC-155

**Rationale:** During the ongoing coronavirus disease 2019 (COVID-19) pandemic, 80% of patients admitted to intensive care units (ICU) require prolonged endotracheal mechanical ventilation (MV). Based on our experience, we hypothesized that the frequency of postextubation laryngeal edema (PLE) was particularly high in these patients. We conducted an observational cohort study to assess this hypothesis and to look for factors associated with PLE.

**Patients and methods/materials and methods:** We retrospectively included patients who received MV for severe COVID-19. Patients who died before the first extubation attempt were excluded. PLE was defined as audible stridor within 2 h after extubation. PLE was recorded and risk factors sought.

**Results:** Of 87 patients intubated between March and June 2020 due to severe COVID-19, 24 died before extubation, leaving 63 patients for the study. Among them, 17 (27%) had PLE. Females were more often affected than males, and re-intubation was more common in patients with vs. without PLE, but the differences were not significant. PLE was significantly associated with a shorter MV duration (median 11 days (IQR [8–18]) versus 24.5 days (IQR [13.0–38.5]), *P* = 0.02) and with persistently positive tracheal samples for syndrome coronavirus type 2 (SARS-CoV-2), as assessed by reverse transcriptase-polymerase chain reaction (RT-PCR), on the day of extubation (73.3% versus 24.3%, *P* = 0.02). Furthermore, the tracheal sample viral load on the day of extubation was higher in patients with than without PLE (median RT-PCR cycles 23.8 (IQR [23.2–29] vs. 36.1 (IQR [34.3–38]), respectively).

**Conclusion:** The frequency of PLE was high (27%) among patients with severe COVID-19. Persistent viral shedding on the day of extubation was associated with PLE. Further studies are needed to assess whether persistent viral shedding is a risk factor for PLE that might indicate preventive corticosteroid therapy.

**Compliance with ethics regulations:** Yes in clinical research.
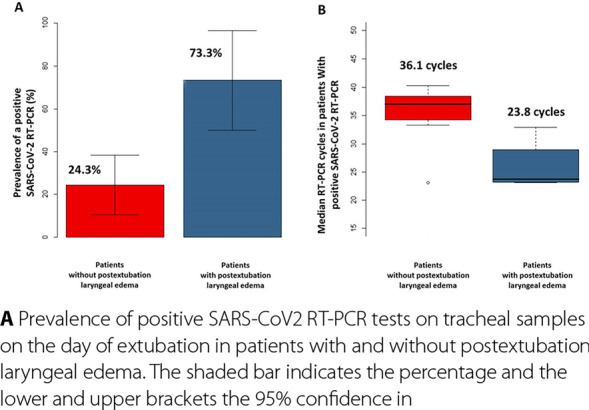


**A** Prevalence of positive SARS-CoV2 RT-PCR tests on tracheal samples on the day of extubation in patients with and without postextubation laryngeal edema. The shaded bar indicates the percentage and the lower and upper brackets the 95% confidence in

### FC-156 Long-term pulmonary function after veno-venous extracorporeal membrane oxygenation for COVID-19 patients

#### Charlotte Becker^2^, Amedée Ego^1^, Olivier Taton^1^, Fabio Silvio Taccone^1^, Romain Courcelle^2^

##### ^1^Hopital d’ERASME, Bruxelles, Belgique; ^2^Centres hospitaliers de Jolimont, La Louvière, Belgique

**Correspondence:** Romain Courcelle - romcourcelle@hotmail.com

*Annals of Intensive Care* 2021, **11(Suppl 1):**FC-156

**Rationale:** In a recent study, Huang et al. described 6-month long-term health consequences of COVID-19 in Wuhan. Lung pulmonary tests after extracorporeal mebrane oxygenation for severe COVID 19 pneumonia were not described.

**Patients and methods/materials and methods:** Between March 10 and April 30, 2020, a total of 92 COVID-19 patients were admitted into two Belgian intensive care unit (ICU); of those, 27 (29%) required veno-venous ECMO and 11/27 (41%) survived at ICU discharge (median age 56 years; 8/11 men; median body mass index 30.5 kg/m^2^). Duration of mechanical ventilation and ECMO therapy was 26 [ranges:17–50] days and 15 [8–36] days, respectively.

**Results:** Among the 11 survivors, pulmonary function tests were obtained in 9 patients after a median of 178 [72–232] days and 147 [55–211] days from ICU admission and ECMO weaning, respectively (Table 1). The results of these tests showed a preserved median forced vital capacity (FVC, 83 [51–99]% predicted) and forced expiratory volume in one second (FEV1, 82 [60–99]% predicted). Pulmonary volumes were also within normal ranges, as the median residual volume and the median total lung capacity were 100 [50–140]% and 90 [50–100]% predicted, respectively. Only the diffusion capacity of the lung for carbon monoxide (DLCO) was decreased (median 58 [37–95]% predicted). The 6-min walking test (6MWT) was performed in six patients and resulted in a median of 468 [365–625] m, corresponding to 69 [63–90]% predicted. All eleven patients were still alive on January 31, 2021.

**Discussion:** A recent study showing persistence of abnormality pulmonary function tests (1) in COVID-19 patients at hospital discharge, our data suggested that, in the most severe pulmonary involvement of COVID-19 requiring ECMO support, long-term diffusion disorders (i.e. 7/9 patients have DLCO < 80% of predicted) may persist without other remarkable abnormalities of their pulmonary function tests. These values are similar to those reported by Grasselli et al. in 18 patients treated with VV-ECMO for other causes than COVID-19 (2). As only a minor limitation on the 6MWT was observed, our findings may also suggest, with all the limitations of a test available only in 6 patients, that the diffusion pulmonary impairment would not significantly compromise mild physical efforts

**Conclusion:** As such, successful recovery from COVID-19 after ECMO support does not appear to compromise long-term respiratory and physical capacities of treated patients.

**References**

1. Mo X, Jian W, Su Z, Chen M, Peng H, Peng P, et al. Abnormal pulmonary function in COVID-19 patients at time of hospital discharge. Eur Respir J. 2020;55(6).
2. Grasselli G, Scaravilli V, Tubiolo D, Russo R, Crimella F, Bichi F, et al. Quality of Life and Lung Function in Survivors of Extracorporeal Membrane Oxygenation for Acute Respiratory Distress Syndrome. Anesthesiology. avr 2019;130(4):572–80.

**Compliance with ethics regulations:** Yes in clinical research.
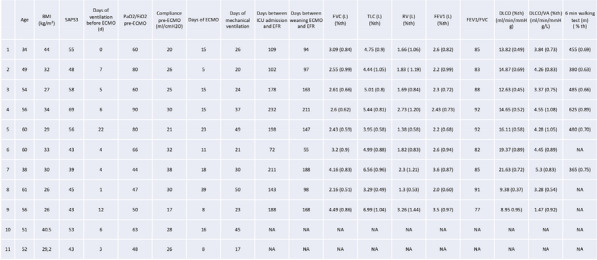


Characteristic and lung pulmonary function tests of survival patients after VV-ECMO for-severe COVID-19 pneumonia

### FC-157 Polygraph evaluation of sleep apnea syndrome in the intensive care unit survivors after SARS-COV-2 pneumonia

#### Ibrahim Traore, Frédéric Claude, Guillaume Eberst, Lucie Laurent, Aurelia Meurisse, Pauline Roux-Claudé, Cindy Barnig, Kevin Bouiller, Catherine Chirouze, Julien Behr, Franck Grillet, Ophélie Ritter, Sébastien Pili-Floury, Hadrien Winiszewski, Emmanuel Samain, Gilles Capellier, Virginie Westeel

##### Université de Franche-Comté, Besançon, France

**Correspondence:** Ibrahim Traore - traore.ibrahim180@gmail.com

*Annals of Intensive Care* 2021, **11(Suppl 1):**FC-157

**Rationale:** The link between sleep apnea syndrome (SAS) and the risk of developing a severe form of SARS-CoV-2 pneumonia has not yet been clearly established. The aim of this study is to characterize prevalence of SAS, 3-month after admission to intensive care units for severe SARS-COV-2 infection.

**Patients and methods/materials and methods:** Our patients were polygraphed, as part of a global follow-up started 3 months after the onset of their symptoms to estimate a reliable prevalence of SAS. We classified them according to the severity of apneic events. A comparison was carried out between patients without or with mild SAS those with moderate or severe SAS.

**Results:** We found a prevalence of sleep apnea syndrome of 91% with a large part for obstructive events (mean proportion of central events at 14.53%). Cardiovascular comorbidities had a significant prevalence for all patients (hypertension: 50%, hypercholesterolemia: 29.4%, type 2 diabetes: 23.5%. Obesity affected 30.9% of all patients (25% of non-apneic or mild SAS patients and 34% of moderate-to-severe SAS patients). There was no significant difference between subgroups for age, gender and for all co-morbidities including obesity, except for ischemic heart disease. Ischemic heart disease exclusively affected the moderate-to-severe SAS group (15.9%, *p* = 0.046). The mean duration of curarization was greater for patients with moderate-to-severe SAS than for the rest of them (mean duration: 9 days vs. 5 days, *p* = 0.025). Similarly for prone sessions, which was applied in 58.3% of cases for absent or mild SAS versus 79.5% for moderate-to-severe SAS (*p* = 0.09). There was no difference in the daily duration of sessions (*p* = 0.19). Intubation and curarization concerned the majority of patients in both subgroups with no significant difference (75% vs 88.6%, *p* = 0.177). All intubated patients (83.9%) have been curarized. There was a significant difference in the use of tracheotomy which concerned exclusively the moderate-to-severe SAS group with a prevalence of 18.2% (*p* = 0.04). A higher Apnea–Hypopnea Index (AHI) in our results did not seem to impact the length of stay in the intensive care unit (14.8 days vs 21 days; *p* = 0.31).

**Conclusion:** Our results underline the attention that should be paid to the management of SAS in the prevention of severe SARS-COV-2 infection due to its causal, secondary or associated comorbidities which represent independent, known risk factors for an unfavorable evolution of the infection.

**Compliance with ethics regulations:** Yes in clinical research.

### FC-158 Systematic evaluation of physical and psychological sequelae in critically ill patients with COVID-19 three to six months after discharge

#### Hiba Bachir, Kim Blanc, Aicha Hamdi, Fabienne Benetti, Bastien Grateau, Vincent Das

##### ^1^CHI André Grégoire GHT Grand Paris Nord Est, Montreuil, France

**Correspondence:** Vincent Das - vincent.das@ght-gpne.fr

*Annals of Intensive Care* 2021, **11(Suppl 1):**FC-158

**Rationale:** COVID-19 is a new disease. Data on long-term outcome of patients treated in ICU for severe COVID-19 are still scarce.

**Patients and methods:** We performed a retrospective monocentric observational study patients treated in ICU for severe COVID-19 with high-flow nasal oxygen (HFNO) or invasive mechanical ventilation in a 18-bed ICU in a non-teaching hospital. Survivors were proposed to have a systematic physical and psychological evaluation 3–6 months after discharge from ICU. Somatic assessment included: physical examination, renal function, chest CT scan, pulmonary function tests, muscle strength (MRC score), echocardiography. Psychological assessment included: anxiety and depression symptoms (HAD scale), post-traumatic stress disorder (IES-R scale). The study is not over. We present here the preliminary results of the survivors from the first epidemic phase (February–May 2020). Data are displayed as median (IQR25%–75%) or numbers (percentage). Univariate analyses with non-parametric tests are intended to identify the factors associated with sequelae persistence.

**Results:** Eight survivors denied assessment. Fourteen patients were assessed. On ICU admission, their age was: 58 (48–61) years, SAPS II was 29 (21–34), P/F ratio on day 1 was 85 (97–105). 5 patients (36%) required HFNO alone, whereas 9 patients (64%) were intubated (curare use: 8 patients (89%), prone position 3 patients (33%), vasopressors use: 9 patients (100%), renal replacement therapy use: 2 patients (22%), mechanical ventilation duration: 19 (14–24) days)). Assessment was performed 98 (90–182) days after discharge from ICU. Five patients (36%) reported dyspnea. Two patients (14%) had PaO2 below 80 mmHg. CT scan was normal in 1 patient (7%) and displayed mild anomalies in 13 patients (93%): mild ground-glass anomalies in 5 (35%) and mild reticular pattern in 12 (86%). No fibrosis pattern was observed. Pulmonary function tests were abnormal in 10 patients (71%): All had restrictive disorder and reduced DLCO. MRC score was 60 (57–60). HAD scale results (*n* = 12) were (Absent/Doubtful/Certain): Anxiety: 7 (58%)/4 (33%)/1 (8%); Depression: 9 (75%)/0 (0%)/3 (25%). IES-R scale results (*n* = 11) were (mild/moderate/severe PTSD): 7 (64%)/3 (27%)/1 (9%). The systematic assessment allowed to diagnose diabetes mellitus in one patient and a catheter-related arteriovenous fistula in one patient

**Conclusion:** Three to six months after ICU admission for severe COVID-19, survivors still have mild anomalies on CT scan, and abnormal pulmonary function tests. Anxiety, depression and PTSD seem to be as frequent as usually after ICU admission. A prolonged follow-up of these patients may be warranted.

**Compliance with ethics regulations:** Yes in clinical research.

### FC-159 COVID19-ARDS: what differs from a usual ARDS?

#### Ahlem Trifi, Eya Seghir, Asma Mehdi, Nabil Bouguezzi, Amal Meftah, Emna Abid, Cyrine Abdennebi, Yosr Touil, Foued Daly, Sami Abdellatif, Salah Ben Lakhal

##### Faculty of Medicine of Tunis, Tunis, Tunisie

**Correspondence:** Ahlem Trifi - trifiahlem2@gmail.com

*Annals of Intensive Care* 2021, **11(Suppl 1):**FC-159

**Rationale:** It is advocated that COVID-19 ARDS is an “atypical’’ ARDS because patients have a better compliance specified as “L” type ARDS. But in real practice, there are often similarities between them. The aim of this study was to explore the different clinical characteristics, ventilatory behaviors, management and outcome between ARDS related to COVID-19 and non-COVID-19 ARDS.

**Patients and methods/materials and methods:** A retrospective comparative double cohort study.

**Results:** we recorded 2 independent groups: COVID-19 ARDS group, *n* = 62 versus non-COVID-19 ARDS group, *n* = 59. Clinical characteristics: the median age of patients with COVID-19 was higher (63 vs 48 years, *p* < 10-3). Co-morbidities: it was showed more of hypertension in COVID group (55% vs 25.5%, *p* = 0.002) and more of chronic respiratory failure in non-COVID group (34% vs 18%, *p* = 0.04). The APACHE II score was higher in non-COVID group (17 vs 10, *p* < 10-3). Blood gas: at admission, the non-COVID group had had more respiratory acidosis (pH = 7.23 vs 7.42, *p* < 10-3 and PCO2 = 51 vs 41, *p* = 0.045) and were less hypoxic (P/F ratio = 122 vs 117, *p* = 0.014). Biology: CRP was higher in non-COVID group (185 vs 174 mg/l, *p* = 0.004) and conversely LDH was higher in COVID group (675 vs 513, *p* = 0.009). ARDS severity: the majority of patients among the 2 groups were classified as severe ARDS without difference (79% vs 84%, NS). Management: invasive ventilation was more required for non-COVID ARDS patients (98% vs 56%, *p* < 10-3). Non-invasive ventilation alternated to high nasal flow oxygen was used for almost all COVID patients. The prone position (PP) was more used in COVID group (60% vs 18%, *p* < 10-3). Steroids were more prescribed in COVID group (100% vs 62%, *p* < 10-3) and antimicrobials were more prescribed in non-COVID group (98% vs 77%, *p* = 0.034). Ventilatory behaviors and outcome: compliance did not differ at the start and in the middle of the follow-up, while it was higher at the end of the follow-up in COVID group. Pulmonary embolism was more common in the COVID group, septic shock and acute renal failure occurred more in the non-COVID group and mortality was high and similar in both groups (table attached)

**Conclusion:** There were many differences: COVID-19-ARDS patients were older with more hypertension and had lower pH, higher capnia, less use of ventilation, more PP and steroids use. The ventilatory behavior was marked by a compliance which increased throughout the follow-up and these data further distinguish the specificity of this type of ARDS.

**Compliance with ethics regulations:** Yes in clinical research.
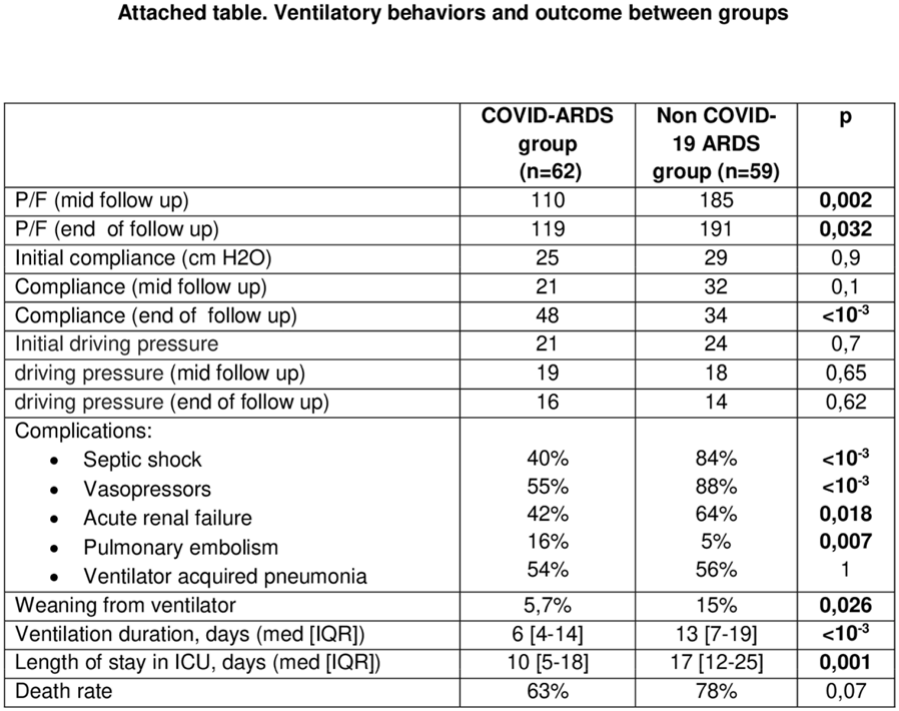


Ventilatory behaviors and outcome between groups

### FC-160 Assessment of extra vascular lung water and pulmonary vascular permeability index in mechanically ventilated SARS-CoV-2 patients: an ARDS just like the others

#### Fanny Garnier, Sonia Machado, Delphine Daubin, Racim Benomar, Philippe Corne, Vincent Brunot, Laura Platon, Liliane Landreau, Valérie Moulaire, Corinne Pelle, Noémie Besnard, Boris Jung, Kada Klouche

##### CHU Lapeyronie, Montpellier, France

**Correspondence:** Fanny Garnier - garnier.fanny26@gmail.com

*Annals of Intensive Care* 2021, **11(Suppl 1):**FC-160

**Rationale:** The clinical syndrome of coronavirus disease 2019 (COVID-19) has become a global pandemic mainly associated with acute respiratory distress syndrome (ARDS) leading to significant morbidity and mortality. Previous reports showed that severe acute respiratory syndrome coronavirus 2 (SARS-CoV-2) associated ARDS was characterized by poorly altered respiratory system compliance (CRS) and severe hypoxemia. Various ARDS phenotypes were also described and suggested. Though increase in pulmonary vascular permeability index (PVPI) associated with accumulation of excess extravascular lung water (EVLW) is the hallmark of ARDS, evaluation of such variables are scarce during SARS-CoV-2 related ARDS. We aimed therefore to measure and investigate these parameters in our critically ills SARS-CoV-2 patients.

**Patients and methods/materials and methods:** All consecutive SARS-CoV-2 patients under invasive mechanical ventilation and monitored by transpulmonary thermodilution system were prospectively included. Data provided by transpulmonary thermodilution was recorded within 2 h after intubation and then every 6 h during the first 72 h. Kinetics of EVLW and PVPI over time were assessed and were compared according to ARDS severity.

**Results:** From August to December 2020, 36 patients among 87 mechanically ventilated SARS-CoV-2 patients, were included with a median age of 68.0 [60.0–75.2] and a median SAPSII score of 42 [31–54]. At inclusion, median left ventricular ejection fraction was 50.0% [50.0–60.0] and norepinephrine infusion was instituted in 27 patients (75.0%). Median EVLW and PVPI were, respectively, 12.5 ml/kg [11.0–13.3] and 2.7 [2.3–3.2] at the first transpulmonary thermodilution measure. Monitoring of EVLW and PVPI over 72 h did not show any significant change. Positive and moderate correlation was found between worst EVLW and worst PVPI (r = 0.74, *p* < 0.01) but the negative correlation between worst EVLW and worst PaO2/FiO2 ratio (r = − 0.21, *p* = 0.21) was not significative as well as between worst PVPI and worst PaO2/FiO2 ratio (r = − 0.25, *p* = 0.14). EVLW and PVPI were not correlated to the severity of ARDS (Table 1).

**Conclusion:** In this study, mechanically ventilated SARS-CoV-2 patients had an EVLW more than 10 ml/kg and PVPI almost greater than 3. Our results strongly suggest that patients with SARS-CoV-2 related ARDS behave like ‘typical’ ARDS with an increased vascular permeability.

**Compliance with ethics regulations:** Yes in clinical research.
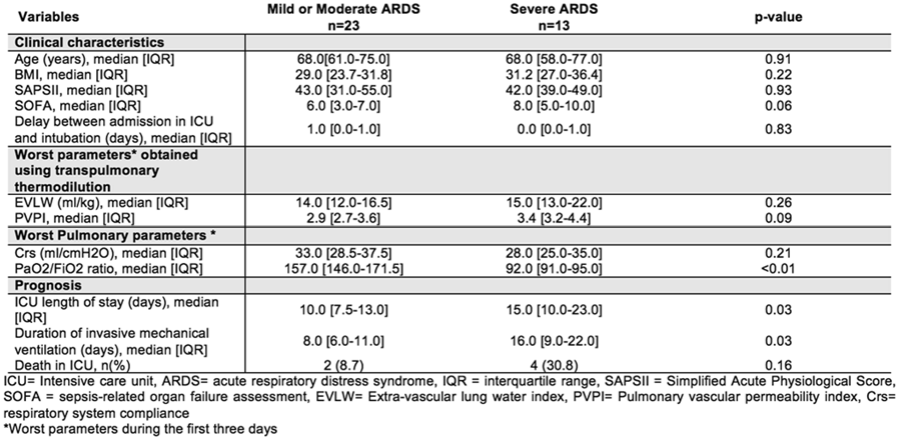


Characteristics of mechanically ventilated SARS-CoV-2 patients with ARDS based on the Berlin definition

### FC-161 Comparison of arterial carboxyhemoglobin levels in Covid-19 and non-Covid-19 ARDS patients

#### Sébastien Di Primio, Paul Paccaud, Philippe Hantson, Ludovic Gérard

##### Cliniques Universitaires Saint-Luc, Bruxelles, Belgique

**Correspondence:** Sébastien Di Primio - seb.diprimio@gmail.com

*Annals of Intensive Care* 2021, **11(Suppl 1):**FC-161

**Rationale:** Recent data suggest that critically ill patients with COVID-19 infection have increased levels of arterial carboxyhemoglobin (aCOHb)^1^. Since aCOHb is frequently increased among critically ill patients^2^, we aimed to investigate whether baseline aCOHb levels and the changes in aCOHb over time were different between COVID-related ARDS and COVID-unrelated ARDS patients.

**Patients and methods/materials and methods:** This is a monocentric retrospective observational cohort study. Consecutive patients with COVID-related ARDS, admitted between March 1st and May 31, 2020 were compared with a historic cohort of consecutive patients with COVID-unrelated ARDS, admitted between 2015 and 2018. All patients were under invasive mechanical ventilation and fulfilled ARDS criteria according to Berlin Definition.

**Results:** 33 COVID-related ARDS were compared with 42 COVID-unrelated ARDS. Baseline characteristics were not different between groups, except for the proportion of active smokers (3% in COVID-related vs 31% in COVID-unrelated group, *p* = 0.002). Despite lower baseline severity in COVID-related ARDS group (APACHE II and SOFA scores at 16 ± 6 and 5 ± 2 vs 24 ± 7 and 9 ± 3, respectively, *p* < 0.001), ICU mortality was similar (48% vs 52%, *p* = 0.73). Baseline aCOHb level was significantly lower in COVID-related ARDS group (0.9 ± 0.3 vs 1.5 ± 0.8%, *p* < 0.001), with no difference in peak aCOHb levels (2.5 ± 1.1 vs 2.2 ± 0.7%, *p* = 0.15). While there was an increase over time of aCOHb in both group, this increase was significantly larger among COVID patients (*p* = 0.004). aCOHb values (baseline, peak or delta peak—baseline) were not related with outcomes. Those findings persisted after adjustment for smoking status.

**Discussion:** Our data show that there is a significant difference in the changes over time of aCOHb between COVID-unrelated and COVID-related ARDS patients, with a lower baseline value and a larger increase over time in the latter group. Different kinetics in aCOHb could be related to increased CO production through a more sustained upregulation of the expression of the heme oxidase (the enzyme synthesizing carbon monoxide in cells) in COVID patients, possibly induced by prolonged oxidative stress. Alternatively, it could reflect a progressively impaired CO elimination through a more severely damaged alveolo-capillary barrier, paralleling the evolution of lung injury.

**Conclusion:** Time course of aCOHb is different between mechanically ventilated COVID-related and COVID-unrelated ARDS patients.

**References**

1. Scholkmann F, Restin T, Ferrari M, Quaresima V. The role of methemoglobin and carboxyhemoglobin in COVID-19: a review. Journal of clinical medicine. 2021; 10(1):50.
2. Scharte M, von Ostrowski TA, Daudel F, Freise H, Van Aken H, Bone HG. Endogenous carbon monoxide production correlates weakly with severity of acute illness. Eur J Anaesthesiol. 2006;23(2):117–22.

**Compliance with ethics regulations:** Yes in clinical research.
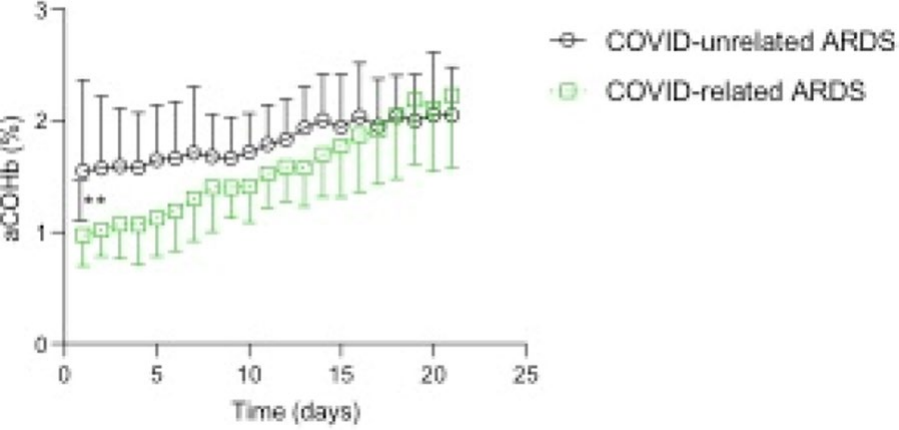


Fig. 1 Time course of aCOHb among COVID-unrelated (*n* = 42) and COVID-related ARDS patients (*n* = 33). ** indicates *p* < 0.005 in comparison of time trends between groups using generalized linear mixed model

### FC-162 Kinetics of FRC Change during prone ventilation in Covid-19 ARDS! A pilot study

#### Manel Lahmar, Iyed Maatouk, Oussama Saadaoui, Saoussen Benabdallah, Zeineb Hammouda, Fahmi Dachraoui, Fekri Abroug, Lamia Besbes

##### CHU Fattouma Bourguiba Monastir, Monastir, Tunisie

**Correspondence:** Manel Lahmar - manel.lahmar@rns.tn

*Annals of Intensive Care* 2021, **11(Suppl 1):**FC-162

**Rationale:** Severe ARDS is commonly managed by mechanical ventilation and prone positioning whichis recommended to reduce ICU mortality through reduction in pulmonary shunt and increase of FRC. However, there is a controversy on the nature of Covid-ARDS in particular whether it has the same features of pulmonary mechanics’ change and response to proning than usually managed ARDS. The aim of our study was to describe the changes in FRC throughout the prone position session.

**Patients and methods:** A prospective study was carried out betweenJanuary 2021 and February 2021 in the Intensive Care Unit of the University Hospital Fattouma Bourguiba of Monastir (Tunisia). We included 5 COVID-19 patients aged more than 18 years with severe ARDS (PaO2/FiO2 ≤ 150 mmHg). All patients received volume assist control ventilation. The FRC was measured in supine position and every 2 h during the prone position until the end of a 24-h session (multiple breath nitrogen washout method, ENGSTROMICU ventilator). At each FRC measurement, the following were recorded: PaO2/FiO2, Vt, respiratory rate, PEEP level, plateau pressure and pulmonary compliance.

**Results:** Five COVID-19 ARDS patients requiring mechanical ventilation were included in the study with a mean PaO2/FiO2 ratio of 78.43 ± 18.27. Four patients were male (80.1%). The mean age was 67.4 ± 8.1 years. The mean body mass index (BMI) was 30.8 ± 2.9 kg/m^2^. The mean SAPS II was 34.2 ± 8.1. The FRC increased from 2460 ± 1311 to 3138 ± 1463 ml from supine to prone position (Fig. 1). Dynamic strain at PEEP increased from 0.15 ± 0.08 to 0.20 ± 0.9 with the change from SP to PP.

**Conclusion:** Changes in pulmonary mechanics during prone-ventilation in Covid-ARDS has a similar pattern than that usually observed in other types of ARDS. Further studies should confirm these preliminary results.

**Compliance with ethics regulations:** Yes in clinical research.
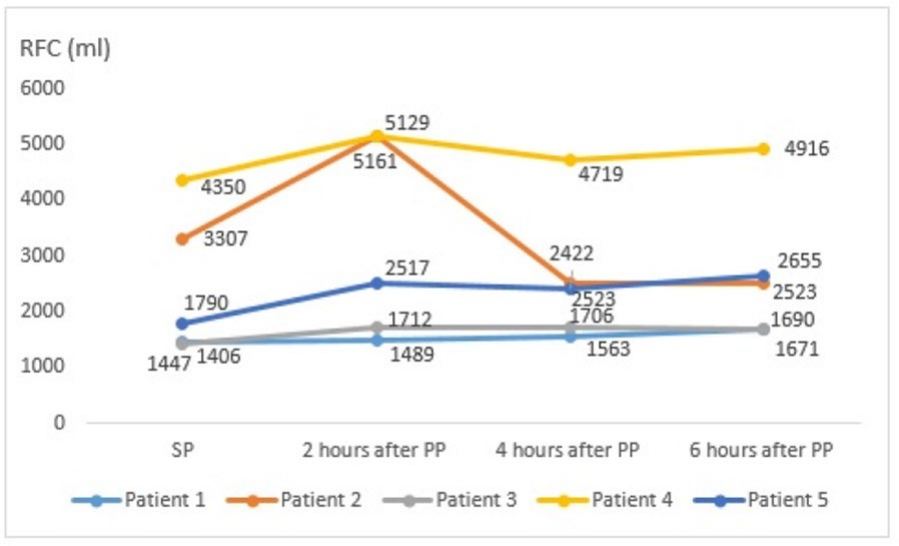


Variation in FRC from supine position (SP) to prone position (PP) for each patient

### FC-163 Airway closure phenomenon in COVID-19 related acute respiratory distress syndrome

#### Clément Brault, Yoann Zerbib, Loay Kontar, Thierry Soupison, Bertrand De Cagny, Maizel Julien, Slama Michel

##### CHU Amiens-Picardie, Amiens, France

**Correspondence:** Clément Brault - brault.clement@chu-amiens.fr

*Annals of Intensive Care* 2021, **11(Suppl 1):**FC-163

**Rationale:** Airway closure is a physiological phenomenon in which the distal airways are obstructed when the airway pressure drops below a given threshold during expiration. We assessed this phenomenon in coronavirus disease 2019 (COVID-19)-related acute respiratory distress syndrome (ARDS).

**Patients and methods:** This study was conducted in the intensive care department of Amiens university hospital (Amiens, France) from January to October 2020. We included mechanically ventilated patients with eligible criteria for moderate and severe ARDS and with a positive RT-PCR assay for SARS-CoV-2. We evaluated airway closure during low-flow (5 L/min) insufflation starting at a PEEP of 5 cmH2O. We defined airway closure as the presence of an inflection point on the time-pressure curve, and considered that the airway opening pressure (AOP) was the pressure at the inflection point. An AOP above 5 cmH2O was considered to be clinically significant.

**Results:** We included 27 patients with COVID-19-related ARDS, of whom 12 (44%) had an AOP above 5 cmH2O. The median AOP was 8 cmH2O (IQR, 7–10), with a maximum value of 17 cmH2O. Four patients had an AOP of 10 cmH2O or more. Three patients had a baseline positive end-expiratory pressure lower than AOP. The optimal PEEP was 21 cmH2O (IQR, 19–21) according to the ARDS network protocol and 17 cmH2O (IQR, 15–18) according to the Express protocol. In one patient, the PEEP level according to the Express protocol remained below the AOP and would have led to airway closure.

**Discussion:** Airway closure interferes with the assessment of airway pressure at end-expiration and exhaled tidal volume (causing errors in driving pressure measurement and in the interpretation of the recruitment to inflation ratio). Moreover, repeated opening and closure movements lead to bronchiolar damage and promote ventilation–perfusion mismatch. In some patients, the AOP may be high (up to 17 cmH2O, in our cohort) and so the PEEP recommended by the ARDS Network or the ExPress study might not be sufficient. However, one must to bear in mind that airway occlusion is not always complete and may only concern some lung areas (due to ARDS-induced lung inhomogeneity). That is why increasing the PEEP to stop airway occlusion might lead to alveolar overdistention.

**Conclusion:** Airway closure is a common phenomenon in COVID-19 related ARDS. The use of a low PEEP suggested by some clinicians exposes the patients with high AOP to consequences of airway closure. In these patients, it is therefore essential to maintain the PEEP above the AOP.

**Compliance with ethics regulations:** Yes in clinical research.

### FC-164 Contrast echocardiography study in COVID-19-related acute respiratory distress syndrome

#### Ahlem Trifi, Asma Ouihibi, Eya Seghir, Asma Mehdi, Nabil Bouguezzi, Emna Abid, Amal Meftah, Yosr Touil, Cyrine Abdennebi, Foued Daly, Sami Abdellatif, Salah Ben Lakhal

##### Medical ICU, la Rabta hospital, Faculty of Medicine of Tunis, Tunis, Tunisie

**Correspondence:** Ahlem Trifi - trifiahlem2@gmail.com

*Annals of Intensive Care* 2021, **11(Suppl 1):**FC-164

**Rationale:** It is reported that COVID-19-related acute respiratory distress syndrome (C-ARDS) to be a shunt pathology. Shunt across patent foramen oval (PFO) is commonly described in usual ARDS (H type). Also, an intrapulmonary shunt, considered as transpulmonary bubble transit (TPBT), may occur in unventilated perfused pulmonary areas secondary to the dilation of the pulmonary vessels. Our aim was to investigate C-ARDS patients using echocardiography contrast test in order to search for the existence of PFO or intrapulmonary (IP) shunt and to interpret these results according to the respiratory compliance.

**Patients and methods:** A cross-sectional study including patients diagnosed with C-ARDS that required mechanical ventilation. A transthoracic echocardiography (TTE) with contrast test up to 3 times [9.5 ml of gelatine solution (plasmagel*) containing 0.5 ml of air injected into an upper vena cava catheter] was performed within 24 h of intubation. A right/left shunt was looked for, evaluated and identified: the passage of micro bubbles in the 3 cardiac cycles diagnosed PFO shunt and TPBT in more than 3 cardiac cycles diagnosed IP shunt. The results were interpreted according to whether respiratory compliance was normal (type L) or altered (type H).

**Results:** 32 ventilated patients for C-ARDS were considered. PFO shunt was detected in 5 patients (15.6%): 3 quantified as important (bubbles > 30), 1 moderate (bubbles between 10 and 30) and 1 minimal (bubbles < 10). TPBT was detected in 9 patients (28%): 1 quantified as important, 3 moderate and 5 minimal. At the same time; the two shunts were detected in 1 patient (3%): important PFO shunt and minimal TPBT. All studied parameters are displayed in the attached Table, in all patients and according to whether their respiratory compliance was lower (type H) or higher (type L) than the median value of 22.5 L/cmH2O. No difference was showed in clinical, oxygen, biology, CT and echocardiography parameters. PFO shunt and TPBT were similar in both groups. The positive end-expiratory pressure (PEEP) tended to be higher in Type L-ARDS (10 vs 8 cmH2O, *p* = 0.08) and driving pressure was higher in type H-ARDS group (19 vs 15 cmH2O, *p* = 0.045).

**Conclusion:** A shunt was objectified in almost half of patients ventilated for C-ARDS and it was rather of the intrapulmonary type. No effect of the ARDS phenotype on its existence or its nature.

**Compliance with ethics regulations:** Yes in clinical research.
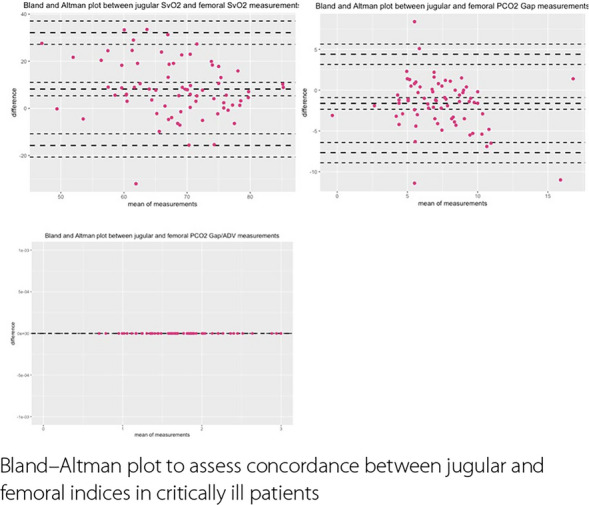


Attached table: Studied parameters in all patients and compared according to the respiratory compliance

### FC-165 High O2 flow rate is necessary to reach acceptable FiO2 in CPAP-treated patients with severe COVID-19

#### Marius Lebret, Emeline Fresnel, Guillaume Prieur, Jean Quieffin, Johan Dupuis, Bouchra Lamia, Yann Combret, Clement Medrinal

##### Groupe Hospitalier du Havre, Le Havre, France

**Correspondence:** Clement Medrinal - medrinal.clement.mk@gmail.com

*Annals of Intensive Care* 2021, **11(Suppl 1):**FC-165

**Rationale:** Covid-19 has been accompanied with a tremendous need for ventilatory supports. Some patients were not able to benefit from invasive mechanical ventilation, mainly because of comorbidities, or due to the lack of equipment availability in the ICU, and were installed with non-invasive continuous positive pressure (CPAP),) with the highest fraction of inspired oxygen (FiO2) as a ceiling treatment (1, 2). Turbines of CPAP devices may be compelled to develop high flows to achieve the set pressure level in patients with high respiratory demand, thereby increasing substantially the oxygen flow rate needed to achieve a high FiO2. We carried out a bench study to measure the oxygen flow rates needed to reach high FiO2, in a model aiming to reproduce the characteristics of severe COVID-19 patients.

**Patients and methods/materials and methods:** Three different set-ups were used: (i) Resistance (*R*) 5 cm H2O/l.s and compliance (*C*) 60 ml/cmH2O. (ii) *R* = 5 cm H2O/l.s and *C* = 40 ml/cmH2O. (iii) *R* = 5 cm H2O/l.s and *C* = 60 ml/cmH2O with deported leaks using a T-connector. Each experiment A, B and C were run at 22, 30 and 35 cycle per minute (cpm), with and without non-intentional leakage. FiO2 was measured at 5, 15, 30, 50, 60 and 80 l/min of O2 or the experiment was stop when 100% FiO2 was reached.

**Results:** An O2 flow rate of at least 30 L/min was necessary to reach a FiO2 > 95% regardless of the set-up, in the absence of unintentional leaks. In the set-ups including unintentional leaks, O2 flow rates of up to 80 l/min were required to reach a 100% FiO2. In those models, the higher the respiratory rate the higher the FiO2 for similar oxygen flows.

**Conclusion:** Regardless the experimental model considered, an O2 flow rate of at least 30 L/min, and up to 80 L/min, was required to reach a 100% FiO2. A reduced compliance and the presence of non-intentional leakage dramatically reduced FiO2. These results could be valuable for the community to guide O2 addition when using home CPAP in severe COVID-19 patients.

**References**

1. Alviset S, Riller Q, Aboab J, et al. Continuous Positive Airway Pressure (CPAP) face-mask ventilation is an easy and cheap option to manage a massive influx of patients presenting acute respiratory failure during the SARS-CoV-2 outbreak: a retrospective c.
2. Aliberti S, Radovanovic D, Billi F, et al. Helmet CPAP treatment in patients with COVID-19 pneumonia: a multicentre cohort study. Eur Respir J. 2020;56. 10.1183/13993003.01935-2020.

**Compliance with ethics regulations:** N/A.

### FC-166 Predictors of high-flow nasal oxygen therapy failure in COVID-19 patients: a retrospective study

#### Jennifer Catano^1^, Violette Suc^1^, Kais Regaieg^2^, Takoua Kzhouri^2^, Daniele Goldran-Toledano^2^, Vincent Das^1^

##### ^1^CHI André Grégoire GHT Grand Paris Nord Est, Montreuil, France; ^2^Groupe Hospitalier Le Raincy Montfermeil GHT Grand Paris Nord Est, Montfermeil, France

**Correspondence:** Vincent Das - vincent.das@ght-gpne.fr

*Annals of Intensive Care* 2021, **11(Suppl 1):**FC-166

**Rationale:** The prognosis of patients with severe COVID-19 treated with invasive mechanical ventilation is poor. High-flow nasal oxygen therapy has been recommended to avoid intubation. Data about HFNO efficacy in patients with severe COVID-19 are still scarce. The ROXX score has been shown to predict HFNO failure outside of the COVID-19 setting. Some authors have recommended using it in COVID-19 patients.

**Patients and methods/materials and methods:** We performed a retrospective bicentric study in the 18-bed and 20-bed ICUs in two non-teaching hospitals. All patients with COVID-19 treated with HFNO in the period February-May 2020 were included. Patients with do-not-intubate order on admission were not included. Criteria for intubation were not standardized. HFNO failure was defined as the need for intubation or hypoxic cardiac arrest. Data collected included: demographic, clinical, and biological usual variables on admission, symptoms duration, CT scan severity of disease, arterial blood gases, P/F ratio under HFNO, ROXX score. Data are displayed as median (IQR25%–75%) or numbers(percentage) Univariate analyses with non-parametrical tests were performed to identify predictors for HFNO failure.

**Results:** Fifty-two patients were included (43 male/9 female, on admission: age 60 (56–64) years, SAPS II 33 (30–38), symptoms duration 10 (6–13 days)). After 2 h under HFNO, required FiO2 was 80% (60–100), P/F ratio was 95 (76–120) and ROXX score was 3.8 (2.8–5.1). HFNO was successful in 19 patients (37%). No hypoxic cardiac arrest occurred under HFNO. Thirty-three patients (63%) required intubation. Only 6 patients were treated with corticosteroids. Comparison between patients with HFNO success or failure using univariate analyses showed that the following admission variables were not significantly different between the two groups: sex, age, BMI, disease severity on CT scan, Fine score, arterial blood gas results, respiratory frequence, biological variables (CRP, LDH). On another hand, symptoms duration before ICU admission was significantly shorter in HFNO failure group than in success group (9 (0–21) versus 11 (5–25) days, *p* = 0.03) P/F ratio (*p* = 0.39) and ROXX (*p* = 0.8) score after 2 h under HFNO were not different between the two groups

**Conclusion:** During the first COVID-19 phase, in our centers, HFNO allowed to avoid intubation in 37% of patients. A short duration of symptoms was predictive of HFNO failure, but not ROXX Score under HFNO.

**Compliance with ethics regulations:** Yes in clinical research.
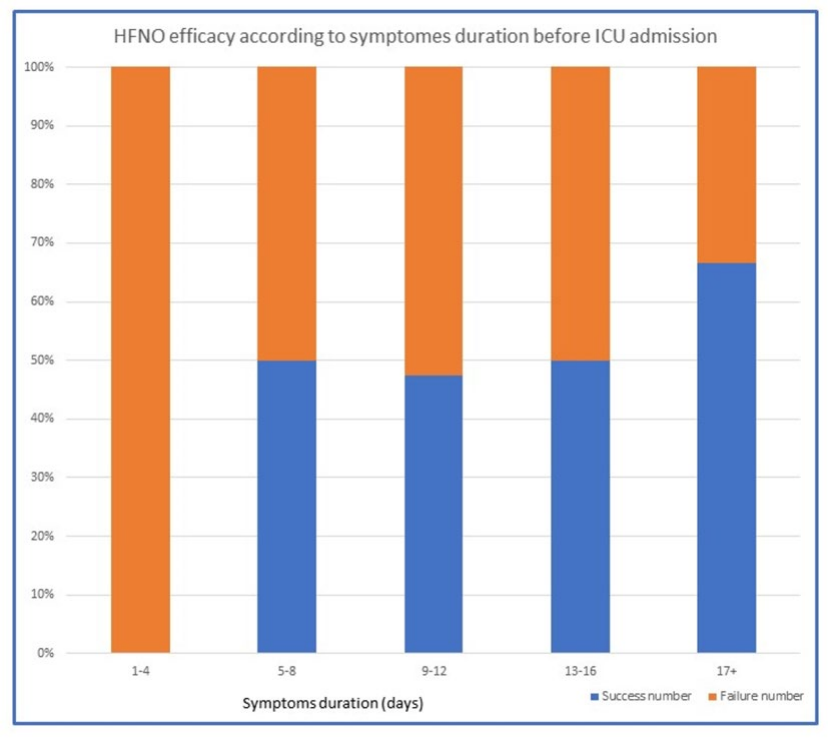


HFNO efficacy according to symptoms duration before ICU admission

### FC-167 Covid-19 severe hypoxic pneumonia: a clinical experience using high-flow nasal oxygen therapy as first-line management

#### Déborah Boyer, Philippe Gouin, Pierre Gildas Guitard, Dorothée Carpentier, Steven Grange, Benoit Veber, Christophe Girault, Fabienne Tamion, Gaetan Beduneau

##### CHU Rouen, Rouen, France

**Correspondence:** Déborah Boyer - deborah_bo@hotmail.com

*Annals of Intensive Care* 2021, **11(Suppl 1):**FC-167

**Rationale:** To report a French experience in patients admitted to Intensive Care Unit (ICU) for severe acute respiratory syndrome coronavirus 2 (SARS-CoV-2) requiring high fractional concentration of inspired oxygen and supported by high-flow nasal cannula (HFNC) as first-line therapy.

**Patients and methods:** Retrospective cohort study conducted in two ICUs of a French university hospital. All consecutive patients admitted during 28-day after the first admission for SARS-CoV-2 pneumonia were screened. Demographic, clinical, respiratory support, specific therapeutics, ICU length-of-stay and survival data were collected. At 3 months, a nutritional and functional evaluation and a medical exam has been performed.

**Results:** Data of 43 patients were analyzed: mainly men (72%), median age 61 (51–69) years, median body mass index of 28 (25–31) (kg/m^2^), median simplified acute physiology score (SAPS II) of 29 (22–37) and median PaO2/fraction of inspired oxygen (FiO2) (P/F) ratio of 146 100–189) mmHg. HFNC was initiated at ICU admission in 76% of patients. Median flow was 50 (45–50) L/min and median FiO2 was 0.6 (0.5–0.8). 79% of patients presented at least one comorbidity, mainly hypertension (58%). At day (D) 28, 32% of patient’s required invasive mechanical ventilation, 3 patients died in ICU. Risk factors for intubation were diabetes (10% vs 43%, *p* = 0.04) and extensive lesions at chest computed tomography (CT) (*p* = 0.023). Patients with more than 25% of lesions at chest CT were more frequently intubated during ICU stay (*p* = 0.012). At ICU admission (D1), patients with higher SAPS II and Sequential Organ Failure Assessment (SOFA) scores (respectively, 39 (28–50) vs 27 (22–31), *p* = 0.0031 and 5 (2–8) vs 2 (2–2.2), *p* = 0.0019), and a lower P/F ratio (98 (63–109) vs 178 (126–206), *p* = 0.0005) were more frequently intubated. Among not-intubated patients, the median lowest P/F was 131 (85–180) mmHg. There were four work stopping, which did not require hospitalization, following coronavirus 2 contaminations. Outcome at 3 months are presented in supplementary data for 21 patients at which an assessment could be carried out (Table 1).

**Conclusion:** Our clinical experience supports the use of HFNC as first line-therapy in patients with SARS-COV-2 pneumonia for whom face mask oxygen does not provide adequate respiratory support.

**Compliance with ethics regulations:** Yes in clinical research.
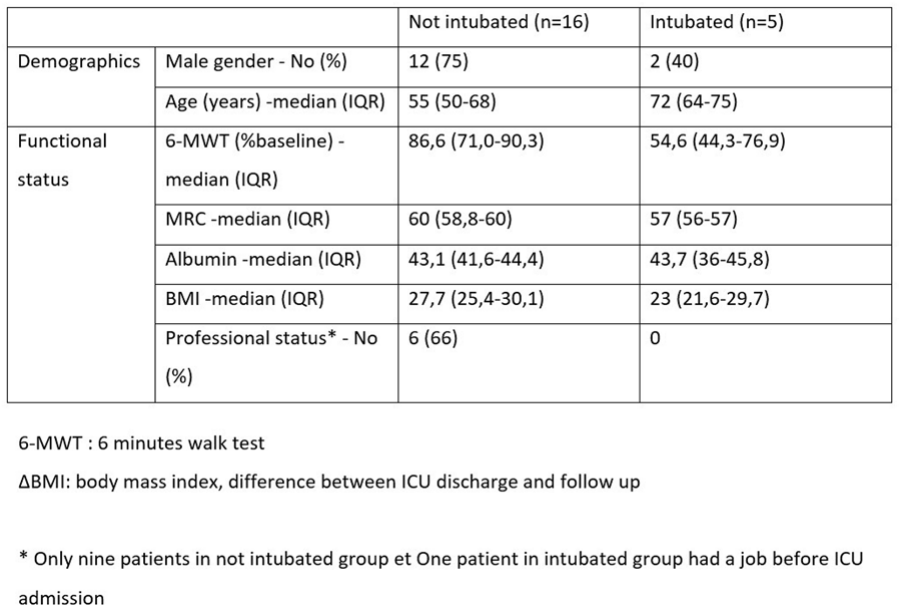


Table 1

### FC-168 Is non-invasive ventilation failure associated with mortality in patients hospitalized for Covid 19 infection?

#### Giulia Moratelli, Kais Regaieg, Sabrine Nakaa, Amir Al Harach, Dany Goldgran-Toledano

##### GHT GRAND-PARIS NORD-EST, Paris, France

**Correspondence:** Giulia Moratelli - giulia.moratelli@gmail.com

*Annals of Intensive Care* 2021, **11(Suppl 1):**FC-168

**Rationale:** The aim of this study is to evaluate the possible association between mortality and the failure of non-invasive ventilation treatments in ventilated patients.

**Patients and methods:** This observational retrospective study. We considered as non-invasive ventilation both high-flow nasal oxygen and classical non-invasive ventilation. We included in the study all Covid 19 patients hospitalized into ICU who needed mechanical ventilation between March 2020 and December 2020.

**Results:** 77 patients have been included in the study and the mortality rate was 42% (*n* = 32). Median age was 60.1 (29–84) years, median BMI was 29 (16–67.6) Kg/m^2^, median SAPS was 40.1 (23–67) and male sex patients were 70% (*n* = 54). 33 (43%) patients have presented an acute kidney injury at the admission and 22% (*n* = 17) have needed renal replacement therapy. 69% (*n* = 53) were shocked. 69% (*n* = 53) patients have needed oral intubation following non-invasive ventilation failure, ECMO was needed in 10% (*n* = 8) cases. After univariate and multivariate analysis it has been found an association between mortality and age, sex and shock utilization while NIV failure was not associated with mortality.

**Conclusion:** In this study it has been found that NIV failure doesn’t increase mortality in Covid 19 patients admitted for acute respiratory failure.

**Compliance with ethics regulations:** Yes in clinical research.

### FC-169 Non-invasive respiratory support in the COVID-19 patients with acute respiratory failure

#### Khedher Sana, Sarsout Ahmed, Mokni Narjes, El Safty Mohamed, Al Dossary Hassen Majid

##### AFH Wady Addawassir, Ryadh, Arabie Saoudite

**Correspondence:** Khedher Sana - sanakhedher@hotmail.fr

*Annals of Intensive Care* 2021, **11(Suppl 1):**FC-169

**Rationale:** Non-invasive ventilation (NIV), is currently enumerated as alternative approach in the management of acute respiratory failure in COVID-19 patients. Herein, we report our experience with the use of NIV. The objectives of our study were to evaluate the efficacy of NIV in COVID-19 patients with acute respiratory failure requiring admission in Intensive care unit (ICU).

**Patients and methods/materials and methods:** This was a prospective observational study was conducted between April 01, 2020 and August 31, 2020 in the ICU of the AFH Wadi Al Dawasir, Saudi Arabia. Consecutive patients aged ≥ 18 years with confirmed COVID-19 pneumonia and fulfilling the criteria of ICU admission were eligible for inclusion in this trial.

**Results:** A total of 36 patients were included. The mean age of the study population was 62 years. 20 were men. 17 patients are diabetic and 17 are hypertensive. Community-acquired pneumonia (83%) followed by pulmonary edema (14%) were the most common causes of respiratory distress. The ARDS criteria were found in 28 patients. The duration of illness prior to ICU presentation ≥ 7 days was encountered in 25%. NIV was used in 30 patients (83.3%), NIV with prone position (PP) in 47.2% and with High-Flow Nasal Cannula (HFNC) in 38.9%. Twenty-one subjects required intubation including 18 subjects, in which NIV was failed. The median time to intubation was 4 days. The underlying indications for intubation were refractory hypoxemia (19.4%) and psychomotor agitation (19.4%). The mortality rate was 40% in patients treated with NIV and 66.6 in intubated patients. Pao2/Fio2 ≤ 100 (*p* = 0.02) comorbidity (*p* = 0.01) and ICU delay admission (*p* = 0.038) are factors associated with NIV failure. The use of PP and HFNC combined with NIV decrease significantly endotracheal intubation. NIV failure is associated with in hospital mortality (*p* = 0.01).

**Conclusion:** In hypoxemic COVID-19 patients, NIV is feasible can decrease the rate of tracheal intubation and mortality particularly if combined with PP and HNFC.

**Compliance with ethics regulations:** Yes in clinical research.

### FC-170 Non-invasive ventilation in mild and moderate ARDS secondary to SARS-CoV-2

#### Hamza Lhoumadi, Ali Derkaoui, Brahim Bechri, Shimi Abdelkarim, Mohammed Khatouf

##### CHU HASSAN II FES,MAROC, Fes, Maroc

**Correspondence:** Hamza Lhoumadi - dr.lhoumadi@gmail.com

*Annals of Intensive Care* 2021, **11(Suppl 1):**FC-170

**Rationale:** During the COVID-19 outbreak, a very high number of infected patients developed pneumonia and many of them complicated with acute respiratory distress syndrome, Invasive mechanical has been associated with high mortality in COVID-19. The optimal management of respiratory failure still unknown. Non-invasive ventilation (NIV) is known to reduce intubation in patients with acute hypoxemic respiratory failure (AHRF). We aimed to assess the outcomes of NIV application in COVID-19 patients with acute respiratory distress syndrome.

**Patients and methods/materials and methods:** We treated 220 patients admitted to our University Hospital, from 15 July 2020 to 15 December 2020 with moderate-to-severe respiratory failure secondary to SARS-CoV-2 treated with NIV. Of the 220 patients with COVID-19, 120 patients were enrolled into the final cohort; Primary outcome was prevention of intubation; the failure of NIV was defined as intubation or death during the hospital stay The primary NIV mode was the Bi-level Positive Airway Pressure initially, PEEP set at 10 cm H2O and then adjusted according to SpO2 and clinical tolerance.

**Results:** The average age was 66.66 years, 54 (45%) were female, 63 patients (52.5%) were smokers. NIV was successful in 77 out of 120 patients (64.1%), 43 patients (35.8%) who failed NIV therapy were intubated, and among them 41 died. Patients who failed NIV were older, and had higher respiratory rate, PaCO2, D-dimer levels before NIV. No statistical difference between the two groups was found in CRP, lymphocytes and renal function. Duration of VNI wash statistically higher in NIV group. Incidence of hospital-associated/ventilator-associated pneumonia was 5%. Mortality and incidence of pneumonia was statistically higher in the progressed to intubation group.

**Conclusion:** This study suggests that the use of NIV is feasible in acute hypoxemic respiratory failure in patients with COVID-19, it is associated with a reduction in the rate of invasive mechanical ventilation and overall mortality, and it can be considered as a valuable option for the management of AHRF in patients not responding to conventional oxygen therapy.

**Compliance with ethics regulations:** Yes in clinical research.

### FC-171 Assessment of the correlation and the concordance between jugular and femoral central venous oxygen saturation and carbon dioxide (CO2)-derived indices in critically ill patients

#### Fanny Garnier, Arthur Chouaikhi, Boris Jung, Kada Klouche

CHU Lapeyronie, Montpellier, France

**Correspondence:** Fanny Garnier - garnier.fanny26@gmail.com

*Annals of Intensive Care* 2021, **11(Suppl 1):**FC-171

**Rationale:** Central venous oxygen saturation (S_cv_O_2_), veno-arterial difference in CO_2_ tension (‘PCO_2_ gap’) and the ratio of PCO_2_ gap with the arteriovenous difference of oxygen (PCO_2_ gap/O_2_ AVD) can be used to assess the circulatory status of critically ill patients. These parameters are validated and calculated from a venous blood sample taken from the superior cava territory. However, it is sometimes impossible to place a venous catheter in the superior cava territory in critically ill patients for several reasons. The value of these indices measured in the inferior vena cava is unknown. We aimed therefore to assess the link between these indices measured in superior and inferior cava territories.

**Patients and methods/materials and methods:** All consecutive critically ill patients who had an arterial catheter and a venous catheter in the superior and inferior vena cava were included. The correlation and the concordance between jugular and femoral S_cv_O_2_, PCO_2_ gap and PCO_2_ gap/O_2_ AVD were assessed using the linear regression, the Lin’s concordance correlation coefficient (rho_c_) and the Bland–Altman plot.

**Results:** From July to October 2020, 42 critically ill patients with a median SAPSII score of 50 [42–69] had the three catheters allowing the realization of 73 arterial gases and 73 venous blood gases in the both cava territories. All patients had norepinephrine infusion, 40 patients (95.2%) were under mechanically ventilation, and 13 (30.9%) had renal replacement therapy. The linear regression found a significantly linear link between jugular and femoral S_cv_O2 (jugular S_cv_O2 = 58.9 + 0.2 femoral S_cv_O2, *p* = 0.03), PCO_2_ gap (jugular PCO_2_ gap = 3.28 + 0.39 femoral PCO_2_ gap, *p* < 0.01) and PCO_2_ gap/O_2_ AVD (jugular PCO_2_ Gap/ADV = 0.11 + 1.03 femoral PCO_2_ Gap/ADV, *p* < 0.01). Low and moderate concordance were, respectively, found between jugular and femoral S_cv_O_2_ (rho_c_ = 0.18, 95% confidence interval (95% CI) 0.01–0.34) and jugular and femoral PCO_2_ gap (rho_c_ = 0.42, 95%CI 0.24–0.57). We found a perfect concordance between jugular and femoral PCO_2_ Gap/ADV (rho_c_ = 0.98, 95%CI 0.98–0.99). These same results have been observed on the Bland–Altman plot (Fig. 1).

**Conclusion:** In the present study, we reported a low and moderate concordance for jugular and femoral ScvO_2_ and PCO_2_ gap. Therefore, the interpretation of S_cv_O_2_ and PCO_2_ gap measured in the inferior vena cava must be very careful. Otherwise, we found a perfect concordance between jugular and femoral PCO_2_ Gap/O_2_ ADV, but it is necessary to confirm these results and determine the clinical relevance in a future study.

**Compliance with ethics regulations:** Yes in clinical research.
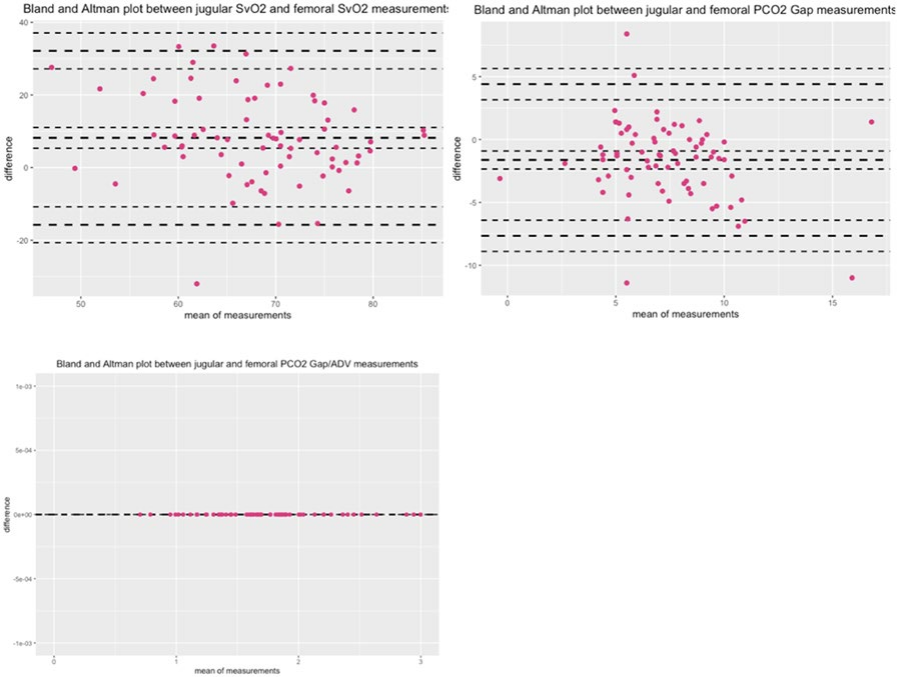


Bland–Altman plot to assess concordance between jugular and femoral indices in critically ill patients

### FC-172 Prognostic value of renal Doppler in acute decompensated precapillary pulmonary hypertension

#### Jérémie Pichon^1^, Charles Fauvel^2^, Anne Roche, Athénais Boucly^1^, Xavier Jais^1^, Mitja Jevnikar^1^, Nathan Ebstein^1^, Olivier Sitbon^1^, David Montani^1^, Marc Humbert^1^, Laurent Savale^1^

##### ^1^Hôpital Bicêtre APHP, Paris, France; ^2^CHU Charles Nicolle, Rouen, France

**Correspondence:** Jérémie Pichon - jeremie.pichon1@gmail.com

*Annals of Intensive Care* 2021, **11(Suppl 1):**FC-172

**Rationale:** Although cardiorenal syndrome has been identified as a major prognostic factor in right heart failure (RHF), the prognostic value of renal Doppler has never been studied in acute decompensated pulmonary hypertension (PH).

**Patients and methods/materials and methods:** The aim of our study was to analyze the prognostic value of the arterial renal resistance index (RRI) and the Doppler-derived renal venous stasis index (RVSI) in precapillary PH patients admitted in intensive care unit (ICU) for acute RHF at admission and at day 3. The primary endpoint was a composite endpoint including death, circulatory assistance or rehospitalization for acute RHF within 90 days following inclusion

**Results:** Among the 46 patients analysed (67% female, age 60 ± 15 years), all received intravenous diuretics and 27 (57%) required inotropic and/or vasopressor support. At admission, the median values of RRI and RVSI were, respectively, 0.75 [IQR, 0.69–0.86] and 0.48 [IQR, 0.27–0.6]. RRI > 0.75 was significantly associated with lower eGFR (*p* = 0.023) and lower systemic mean arterial pressure (*p* = 0.015). RVSI > 0.48 was associated with lower eGFR (*p* = 0.004), higher central venous pressure (*p* < 0.001) and higher NT-proBNP (*p* = 0.013). A primary endpoint event occurred in 12 patients (24%). In Kaplan–Meier analysis, the event rate was higher in patients with RRI > 0.75 at admission (*p* = 0.02) but not in patients with RVSI > 0.48. At day-3, a decrease in both RRI and RVSI was associated with better outcomes (*p* = 0.003 and *p* < 0.0001, respectively). No event occurs in patients who improved both RRI and RVSI at Day 3

**Conclusion:** Arterial and venous renal Doppler seems to be a relevant tool for prognostic evaluation of patients admitted in ICU for acute decompensated precapillary PH

**Compliance with ethics regulations:** Yes in clinical research.

### FC-173 Rate control of atrial fibrillation with landiolol is safe in critically ill patients with SARS-CoV-2 infections

#### Geoffroy Hariri, Tomas Urbina, Sandie Mazerand^1^, Naïke Bigé^1^, Jean-Luc Baudel, Hafid Aït-Oufella

##### Hôpital Saint Antoine - GH Sorbonne Université, Paris, France

**Correspondence:** Geoffroy Hariri - geoffroyhariri@hotmail.com

*Annals of Intensive Care* 2021, **11(Suppl 1):**FC-173

**Rationale:** Atrial fibrillation (AF) is a frequent condition in patients admitted in Intensive Unit Care (ICU) with acute circulatory failure leading to increased mortality. Landiolol is a short acting beta-blocker used for more than 15 years in AF patients either to control heart rate or to prevent supra-ventricular arrythmia occurrence in case of cardiac surgery. Here, we described in critically ill AF patients admitted in ICU for SARS-CoV-2 infections, our experience in using landiolol in term of efficacy and safety.

**Patients and methods/materials and methods:** We prospectively collected data from adult patients admitted for SARS-CoV-2 infections with persistent AF when heart rate was over 120 bpm. Landiolol was started at a minimum dose of 0.2 µg/kg/min and progressively increased to achieve 20% reduction in heart rate. Classical hemodynamic and parameters were recorded every 2 h during the first 24 h of drug infusion.

**Results:** Fifteen patients with SARS-CoV-2 infection (4/15, 27% women) were included during a 6-month period. Median age was 70 years-old [67–72] and median SOFA score was 11 [7–12]. Six patients (6/15, 40%) had a history of chronic AF, all treated with beta-blockers at home, and the other had recent AF resistant to electric cardioversion. Median left ventricular (LV) function was 55% [50–57] and no patient had LV ejection fraction < 40%. All included patients had mechanical respiratory support and eleven (11/15, 73%) required norepinephrine infusion. Time between ICU admission and landiolol initiation was 2 [0–5] days. Landiolol infusion was started at 0.2 µg/kg/min and dosage was increased slowly reaching 3.9 [1.6–7.0] µg/kg/min at 24 h. We report here an overall heart rate reduction of 23% (115 [108–117] vs 150 [138–160] bpm; *p* < 0.01) (Fig. 1) without any negative impact on global hemodynamic or tissue perfusion parameters. Interestingly, we found that following landiolol infusion, norepinephrine dosage decreased in 9/11 patients (81%), and mean norepinephrine dose significantly decreased (0.7 [0.2–1] vs 1 [0.4–1.5] vs µg/kg/min; *p* = 0.04).

**Conclusion:** In this observational study, we described our experience in using landiolol to control heart rate in severe Covid-19+ patients with AF. Landiolol tolerance and safety have been reported by several groups and was recently validated in a multicenter randomized trial in septic shock patients. In our department, Landiolol infusion was started at very low doses and dosage was increased slowly. Using this protocol, hemodynamic tolerance was excellent and we observed a decrease in norepinephrine need after the initiation of Landiolol.

**Compliance with ethics regulations:** Yes in clinical research.
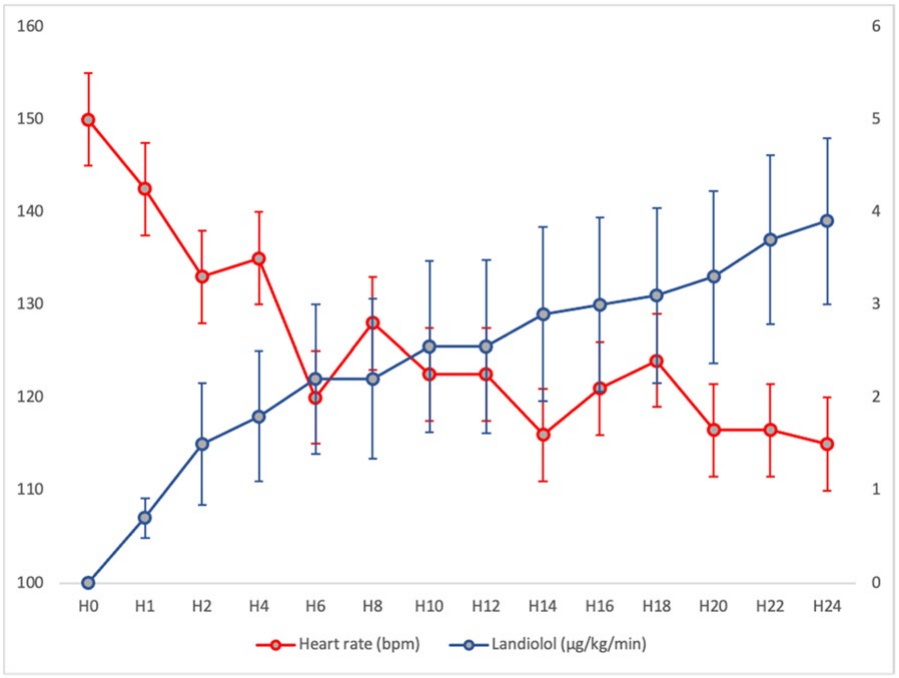


Heart rate (red) and landiolol dosage (BLUE) changes during the first 24 h of landiolol infusion

### FC-174 Predicting preload responsiveness by decreasing positive end-expiratory pressure in mechanically ventilated patients in intensive care unit: the PEEP-test study

#### Christopher Lai, Rui Shi, Jean-Louis Teboul, Alexandra Beurton, Francesca Moretto, Soufia Ayed, Arthur Pavot, Nadia Anguel, Xavier Monnet

##### CHU Kremlin-Bicêtre, Le Kremlin-Bicêtre, France

**Correspondence:** Christopher Lai - christopher.lai@hotmail.fr

*Annals of Intensive Care* 2021, **11(Suppl 1):**FC-174

**Rationale:** In mechanical ventilated patients, positive end-expiratory pressure (PEEP) decreases cardiac preload by increasing the right atrial pressure and increases right ventricle afterload. We hypothesized that decreasing the level of PEEP (PEEP-test) may be used as a preload challenge to detect preload responsiveness.

**Patients and methods/materials and methods:** In patients under mechanical ventilation with no spontaneous breathing and PEEP ≥ 10 cmH2O (“high level”), preload responsiveness was assessed by a passive leg raising (PLR) maneuver. Preload responsiveness was defined as an increase in cardiac index (CI) during PLR ≥ 10%. The PEEP-test consisted in reducing PEEP from high level to 5 cmH2O. Pulse contour-derived CI (PiCCO2) was monitored during PLR and PEEP-test.

**Results:** We enrolled 49 patients among whom 23 were preload responsive (median increase in CI during PLR: 14% (interquartile range (IQR): 12–16%). The median PEEP level at baseline was 12 (IQR: 10–15) cmH2O and the PEEP-test resulted in a median decrease of PEEP by 8 (IQR: 6–10) cmH2O, with no difference between preload responsive and unresponsive patients. Among preload responsive patients, PEEP-test induced an median increase in CI of 17 (IQR: 11–20)% (from 2.4 ± 0.7 to 2.8 ± 0.8 L/min/m^2^, *p* < 0.0001). In preload unresponsive patients, PLR and PEEP-test increased CI by, respectively, 4 (2–7)% and 7 (5–10)%, respectively. These changes were significantly lower than in preload responsive patients. Preload responsiveness was predicted by an increase in CI ≥ 8% during PEEP-test with a sensitivity of 96% (95% confidence interval: 78–99%) and a specificity of 73 (95%CI: 52–88)%. The mean area under the curve of PEEP-test for detecting preload responsiveness was 0.89 (standard deviation (SD): 0.05) (*p* < 0.0001 vs. 0.5). The Spearman’s correlation coefficient between the PLR and the PEEP-test-induced changes in CI was 0.80 (95%CI: 0.67–0.88, *p* < 0.0001).

**Conclusion:** The CI increases during a PEEP-test, which consists in reducing PEEP to 5 cmH2O, reliably detect preload responsiveness in mechanically ventilated patients with a PEEP level ≥ 10 cmH2O.

**Compliance with ethics regulations:** Yes in clinical research.

### FC-175 Tidal volume challenge to predict preload responsiveness in patients with acute respiratory distress syndrome under prone position

#### Rui Shi, Soufia Ayed, Francesca Moretto, Nello De Vita, Francesco Gavelli, Simone Carelli, Arthur Pavot, Christopher Lai, Xavier Monnet, Jean-Louis Teboul

##### ^1^Université Paris-Saclay, AP-HP, Service de médecine intensive-réanimation, Hôpital de Bicêtre, DMU CORREVE, Inserm UMR S_999, FHU SEPSIS, Groupe de recherche clinique CARMAS, Le Kremlin-Bicêtre, France, Le Kremlin-Bicêtre, France

**Correspondence:** Rui Shi - rui.shi@u-psud.fr

*Annals of Intensive Care* 2021, **11(Suppl 1):**FC-175

**Rationale:** Alteration of hemodynamics may occur in patients with acute respiratory distress syndrome (ARDS) during prone position. In this setting, testing preload responsiveness without requiring cardiac index (CI) measurements has been poorly investigated. Our study investigated the ability of pulse pressure variation (PPV) and its changes during a 1-min tidal volume (TV) challenge (TVC) to predict preload responsiveness in ARDS patients under prone position (Trials registration: NCT04457739).

**Patients and methods/materials and methods:** We prospectively included ARDS patients ventilated with a 6 mL/kg TV under prone position. Using a pulse contour analysis monitor, we measured PPV and changes in CI during a Trendelenburg maneuver (ΔCI_TREND_). After transiently increasing VT to 8 mL/kg, we measured first absolute changes in PPV during TVC (ΔPPV TVC_6–8_), and then changes in CI during end-expiratory occlusion (EEO) (ΔCI EEO_8_). Preload responsiveness was defined by both ΔCI_TREND_ ≥ 8% (according to Yonis et al^1^) and ΔCI EEO_8_ ≥ 5% (according to Gavelli et al^2^). Preload unresponsiveness was defined by both ΔCI_TREND_ < 8% and ΔCI EEO_8_ < 5%. We excluded from the analysis nine cases where only ΔCI_TREND_ ≥ 8% or only ΔCI EEO_8_ ≥ 5%.

**Results:** Fifty-nine sets of measurements were analyzed in 36 patients (22 with COVID-19), (62 ± 12 y.o.), under prone position for 10 (1–15) hours. The mean arterial pressure was 80 ± 13 mmHg (under norepinephrine in 45 cases at a dose of 0.3 (0.1–0.5) µg/kg/min). The driving pressure was 14 (11–17) cmH_2_O, the respiratory system compliance was 28 (22–39) mL/cmH_2_O and the positive end-expiratory pressure was 13 (10–15) cmH_2_O. In 29 cases, patients were classified as preload responders. The baseline PPV predicted preload responsiveness with an area under the receiver operating characteristic curve (AUROC) of 0.83 ± 0.05 (threshold 6%; sensitivity: 66%, specificity: 83%). The ΔPPV TVC_6–8_ predicted preload responsiveness with an AUROC of 0.90 ± 0.05 (threshold 2%; sensitivity: 97%, specificity: 87%). The grey zone of baseline PPV to predict preload responsiveness ranged from 4% to 9% and included 39 measurements. Analysis of these 39 cases shows that ΔPPV TVC_6–8_ predicted preload responsiveness with an AUROC of 0.87 ± 0.07 (threshold 2%, sensitivity: 95%, specificity: 84%) (*p* = 0.038 vs. baseline PPV: 0.61 ± 0.09).

**Conclusion:** In patients with ARDS under protective ventilation during prone position, it is possible to reliably predict preload responsiveness without the need for CI measurements, by measuring changes in PPV during a TVC. TVC is particularly helpful to correctly classify preload responders and nonresponders when PPV values at TV 6 mL/kg lie between 4 and 9%.

**References**

1. Yonis et al. Crit Care 2017;21:295.
2. Gavelli et al. Ann Intensive Care 2020;10:65.

**Compliance with ethics regulations:** Yes in clinical research.

### FC-176 Validation study on healthy volunteers of a capillary refill time measurement device prototype

#### Martin Ruste, Laure Cazenave, Jean-Luc Fellahi, Matthias Jacquet-Lagrèze

##### Hôpital Cardiologique Louis Pradel, Hospices Civils de Lyon, Bron, France

**Correspondence:** Martin Ruste - martin.ruste@gmail.com

*Annals of Intensive Care* 2021, **11(Suppl 1):**FC-176

**Rationale:** Capillary refill time (CRT) is a simple and relevant clinical sign to assess peripheral perfusion. It may be used as a diagnostic or prognostic tool and to guide resuscitation (1). However, it is susceptible to numerous factors as patient age, cutaneous temperature, ambience light and intensity or duration of the pressure applied. Dicartech company (Davézieux, France) is developing a fully automatized portable CRT measurement device. We aimed to evaluate the diagnostic performance of this first-generation prototype to detect a vascular occlusion test (VOT) on healthy volunteers.

**Patients and methods/materials and methods:** DiCART™ device is composed of a piston which is applied on the skin with a calibrated pressure. After withdrawal of the piston, a video of the skin recolouration is recorded in stable light condition. Finally, a mathematical algorithm determines CRT (CRT_DiCART_) from the video. Manual CRT (CRT_CLIN_) was measured as previously described (2). CRT determined by investigators after videos reviewing was named CRTVIDEO. CRT was measured three times by method before and after three different VOT: ”arterial VOT” (pneumatic tourniquet 50 mmHg above systolic arterial pressure); “venous VOT” (30 mmHg) and “control VOT”. VOT were performed successively in a randomized order, alternatively by one and the other limb. Investigator assessing CRT was blinded from VOT type. The whole protocol was first carried out on the upper limb and then on the lower limb. Statistical analysis: ROC curves were constructed to evaluate diagnostic performance to detect VOT. Agreement between CRT measurements methods was assessed by Bland–Altman method with calculation of percentage of error.

**Results:** 20 healthy volunteers were included. Five participants were excluded because of prototype dysfunction. CRT_CLIN_ had an ROC_AUC_ of 1.00 (95%CI: 1.00; 1.00), CRT_VIDEO_ 0.96 (95%CI: 0.88; 1.00) and CRT_DiCART_ 0.92 (95%CI: 0.79; 1.00) to detect arterial VOT on the upper limb. Results were not reproducible at the lower limb. CRT did not detect venous VOT with ROC_AUC_ > 0.75. Precision of CRT_CLIN_ and CRT_VIDEO_ was significantly higher than CRT_DiCART_ (0.18 and 0.20 vs 0.28 *p* < 0.05). For 3 measures average, Bland–Altman plots are reported in Fig. 1. The percentage of error was 76% between CRT_CLIN_ and CRT_VIDEO_ and 87% between CRT_CLIN_ and CRT_DiCART_.

**Conclusion:** The prototype developed by DICARTECH was accurate to detect an experimental arterial ischemia. However, precision was lower than with the manual method, and the agreement was not acceptable. Those results give leads to improve the mechanics and the algorithm of future devices.

**References**

1. Hernández G, Ospina-Tascón GA, Damiani LP, Estenssoro E, Dubin A, Hurtado J, et al. Effect of a Resuscitation Strategy Targeting Peripheral Perfusion Status vs Serum Lactate Levels on 28-Day Mortality Among Patients With Septic Shock: The ANDROMEDA-SHOCK.
2. Ait-Oufella H, Bige N, Boelle PY, Pichereau C, Alves M, Bertinchamp R, et al. Capillary refill time exploration during septic shock. Intensive Care Med. 2014;40(7):958–64.

**Compliance with ethics regulations:** Yes in clinical research.
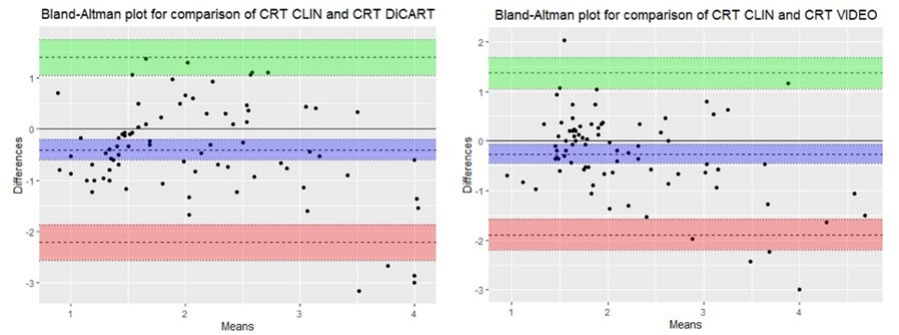


Fig. 1 Bland–Altman plots

### FC-177 Evaluation of left ventricular global longitudinal strain using transesophageal echocardiography in ventilated septic shock patients

#### Marine Goudelin, Sertac Karatas, Bruno Evrard, Anne-Laure Fedou, Thomas Daix, Arnaud Desachy, Amandine Sanson, Julien Vaidie, Bruno François, Philippe Vignon

##### CHU Dupuytren, Limoges, France

**Correspondence:** Marine Goudelin - marine.goudelin@chu-limoges.fr

*Annals of Intensive Care* 2021, **11(Suppl 1):**FC-17

**Rationale:** The prognostic value of global longitudinal strain (GLS) of the left ventricle (LV) in patients with septic shock remains debated (1,2). Although transesophageal echocardiography (TEE) in increasingly used for the hemodynamic assessment of ventilated patients in the ICU, the feasibility of GLS measurement through the transesophageal approach has not yet been assessed in an unselected cohort of critically ill patients. Accordingly, we sought to determine the feasibility of LV GLS measurement in mechanically ventilated patients assessed hemodynamically with TEE for septic shock over a 10-year period.

**Patients and methods:** This was a retrospective, observational, single-center study. Eligible patients were hospitalized between 2010 and 2020 for septic shock (Sepsis-3 definition) with a TEE examination performed within 72 h after ICU admission, digitally archived and suitable for LV GLS measurement. The primary endpoint was the feasibility of LV GLS using TEE. Secondary endpoints were the intra- and inter-observer reproducibility, agreement between LV GLS and LV ejection fraction (EF) and LV GLS value according to ICU mortality.

**Results:** Between 2010 and 2020, 291 patients were admitted to our ICU for septic shock. ICU mortality reached 35%. Of them, 204 patients were hemodynamically assessed using TEE within 72 h after admission. LV GLS could be measured in 143 of 204 patients (76%). Reasons for not measuring LV GLS were the lack of adequate software tracking in 44 patients (72%), non-sinus rhythm in 16 patients (26%) and mechanical mitral valve in one patient (2%). Median LV GLS was − 13.7% [− 17.4/− 11]. Both the intra- and inter-observer reproducibility were excellent (intraclass correlation coefficient: 0.94 [95% CI: 0.87–0.98] and 0.90 [95% CI: 0.72–0.97], respectively). There was a positive correlation between LV GLS and LVEF, with a Pearson coefficient of 0.673 (*p* < 0.001).There was no statistically significant difference between the median LV GLS in survivors and in deceased patients (− 13.6% [− 17.1/− 10.9] vs − 14.2% [− 17.8/− 10.9]: *p* = 0.603).

**Conclusion:** The measurement of LV GLS using TEE is feasible in three-fourth of ICU ventilated patients with septic shock. Intra- and inter- observer reproducibility are excellent. Although no relationship was found between LV GLS value and ICU mortality, further assessment is warranted in a larger prospective multicenter study.

**References**

1. Chang W-T and al. Left ventricular global longitudinal strain is independently associated with mortality in septic shock patients. Intensive Care Med. 2015;41(10):1791–9
2. Lanspa MJ and al. Associations among left ventricular systolic function, tachycardia, and cardiac preload in septic patients. Ann Intensive Care. 2017;7(1):17

**Compliance with ethics regulations:** Yes in clinical research.

### FC-178 Psychological distress in critically ill COVID-19 patients’ relatives during the first wave of the pandemic

#### Anne-Françoise Rousseau^1^, Jean-Marc Hougardy^1^, Sourour Chaabane^1^, Régine Hardy^1^, Virginie Deschamps^1^, Nicole Barthelemy^1^, Martine Devos^1^, Nadia Dardenne^2^, Malorie Bodart^1^, Benoit Misset^1^

##### ^1^CHU de Liège, Liège, Belgique; ^2^Université de Liège, Liège, Belgique

**Correspondence:** Anne-Françoise Rousseau - afrousseau@chuliege.be

*Annals of Intensive Care* 2021, **11(Suppl 1):**FC-178

**Rationale:** Hospitalization in an intensive care unit (ICU) is stressful for patients’ relatives. The objectives of this monocenter prospective study were to assess their psychological distress during the unique lockdown context and to search for some risk factors.

**Patients and methods:** The closest referents of adults admitted to ICU for COVID-19 pneumonia in March and April 2020 were consecutively included. Psychologists conducted the interviews by phone, one (M1) and three (M3) months following ICU discharge. Relatives completed the Hospital Anxiety and Depression scale (cutoff defined as ≥ 11/21 for anxiety and depression in the HADS-A and HADS-D subscales, respectively) and the Impact of Event Scale—Revised (IES-R: cutoff defined as defined as > 36/88 for post-traumatic stress disorder (PTSD)). Their psychological history, the co-existence of concomitant other stressful private events, and the mean of communication with ICU teams were noted. Relatives were divided into 2 groups according to patient’s status at ICU discharge: Deceased Group and Alive Group. Data were expressed as median [Q1–Q3] or percentages, and compared using Kruskal–Wallis, Wilcoxon, or Fisher tests as needed.

**Results:** We included 37 relatives of 57 [41–67]y at M1 and 33 completed the study at M3. In the whole population, HADS-A was higher at M1 compared to M3, respectively 8 [4.5–12] and 6 [4–9.5], *p* = 0.021. Similar observation was made for IES-R: respectively, 26 [17–45] and 24 [9.5–41], *p* = 0.002. HADS-D score was unchanged between M1 and M3, respectively, 5 [2–8.5] and 5 [2–7.5], *p* = 0.244. Proportion of relatives with depression and PTSD tended to decrease at M3 (28.6% for both conditions) compared to M1 (respectively, 31.2 and 37.5%) in the Deceased Group. At the opposite in the Alive Group, proportion of relatives with depression and PTSD tended to increase at M3 (respectively, 15.8 and 36.8%), compared to M1 (respectively, 4.8 and 28.6%). Videoconferencing (vs phone call alone) or concomitant stressful events did not influence any scores. Relatives with a history of anxiety and/or depression had higher HADS-A scores at M1 (*p* = 0.001) and at M3 (*p* = 0.002), The same was observed for IES-R at M1 (*p* = 0.02).

**Conclusion:** Psychological distress was observed in relatives of ICU survivors, as in relatives of deceased patients. A history of mental health disorders seemed to be a risk factor for anxiety and PTSD in relatives who experienced the ICU stay of their loved ones. Videoconferencing did not impact relatives’ psychological burden.

**Compliance with ethics regulations:** Yes in clinical research.

### FC-179 Critically ill COVID-19 patients: a physiology-based approach for ICU admission criteria

#### Samuele Ceruti^1^, Maira Biggiogero^1^, Pierandrea Maida^1^, Andrea Glotta^1^, Andrea Saporito^2^, Christian Garzoni^1^

##### ^1^Clinica Luganese Moncucco, Lugano, Suisse; ^2^Ospedale Regionale di Bellinzona e Valli, Bellinzona, Suisse

**Correspondence:** Ceruti Samuele - samuele.ceruti@moncucco.ch

*Annals of Intensive Care* 2021, **11(Suppl 1):**FC-179

**Rationale:** During the COVID-19 pandemic, strict and standard admission criteria to intensive care units (ICU) were needed. In our COVID-19 center we implemented standard criteria for ICU admission, a low-PEEP strategy and others associated features, like aggressive thromboembolism management. Primary outcome of this study was to analyze the mortality rate in patients evaluated for admission to the ICU comparing patients admitted vs patients not accepted and regularly followed in the medicine department; secondary outcome was a comparison of clinical and biological data between the two groups.

**Patients and methods:** A retrospective analysis was conducted on all consecutive patients with acute respiratory distress syndrome due to COVID-19 pneumonia who underwent to ICU consultation from March 16th to April 12th, 2020. SpO_2_ below 85% and/or dyspnea were admission criteria; exclusion criteria were DNR (do not resuscitate) orders.

**Results:** Thirty-six patients were consulted by the intensivist; 23 (64%) patients were admitted to the ICU; 13 (36%) were monitored and followed on Internal Medicine ward. Global mean age was 65 years (38–82), with a median SpO_2_ of 90% (88–94), PaO_2_ of 63.2 mmHg (51.35–76.55), pCO_2_ of 35.2 mmHg (31.9–39.1); no difference was found between admitted and rejected patients regarding SpO_2_ (*p* = 0.41), PaO_2_ (*p *= 0.91), PCO_2_ (*p* = 0.2), and oxygen-therapy (*p* = 0.18). All patients not admitted in the ICU improved their clinical condition and none of them died. Regarding patients’ distribution about SpO_2_ less than 92%, the ICU-group hold 17 (68%) patients while the rejected-group hold 9 (75%) patients (*p* = 1.0). Conversely, Chi-square analysis reported significant differences regarding dyspnea distribution (*p* = 0.03) as well as SpO_2_ less than 85% (*p* < 0.001).

**Conclusion:** Patients with COVID-19 interstitial pneumonia often presented tachypnea and desaturation, without dyspnea or severe neurological symptoms or any other organ damage. A conservative approach based on strict surveillance until the onset of dyspnea or desaturation with SpO_2_ 85% or even lower, before proceeding with ICU admission, resulted a good procedure to avoid ICU overload and to guarantee ‘ICU refused’ patients survival.

**References**

1. Swiss Academy Of Medical Sciences. COVID-19 pandemic: triage for intensive-care treatment under resource scarcity. Swiss Med Wkly. 2020;150(1314):w20229. https://smw.ch/article/doi/smw.2020.20229.
2. Bouadma L, Lescure FX, Lucet JC, Yazdanpanah Y, Timsit JF. Severe SARS-CoV-2 infections: practical considerations and management strategy for intensivists. Intensive Care Med. 2020;46(4):579–82.

**Compliance with ethics regulations:** Yes in clinical research.
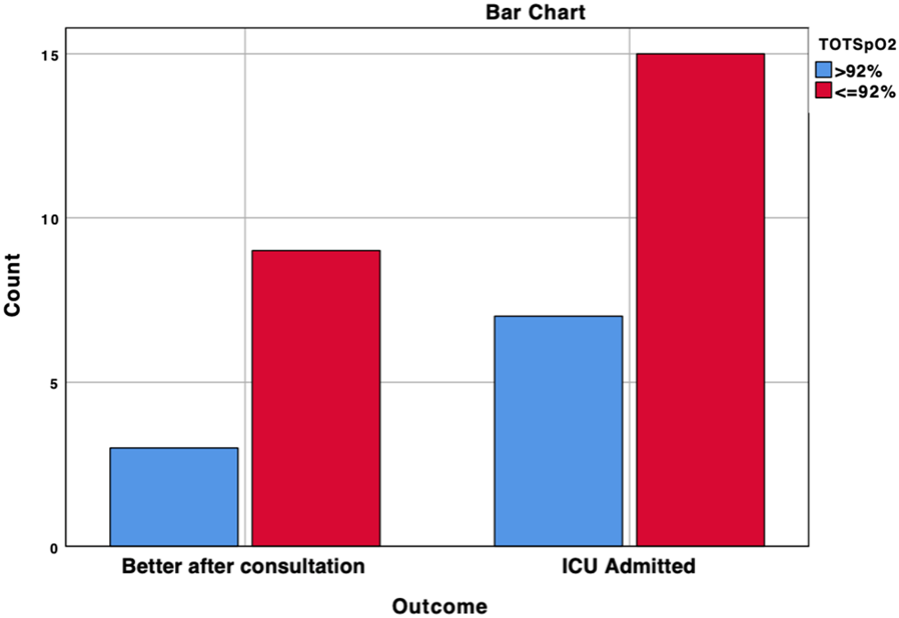


Prevalence of SpO2 distribution in patients at the Intensive Care consultation, stratified according to the outcome (ICU-admitted, improved without ICU). Please note that almost a quarter of patients presented to Intensive Care consultation, had SpO2 lowe

### FC-180 Psychological evaluation and support in COVID-19 critically ill patients: a feasibility study

#### Renaud Prével, Julien Coelho, Arthur Orieux, Audrey Fontaine, Aurélie Mollar, Anouck Chignac, Sarah Gimenez, Flore Mathias, Pierre Philip, Didier Gruson, Stéphanie Bioulac

##### CHU Bordeaux, Bordeaux, France

**Correspondence:** Renaud Prével - renaud.prevel@hotmail.fr

*Annals of Intensive Care* 2021, **11(Suppl 1):**FC-180

**Rationale:** Acute respiratory distress syndrome (ARDS) survivors frequently suffer from anxiety, depression or post-traumatic stress disorder (PTSD). COVID-19 pandemics has provoked an unprecedented strain on our health systems, especially on intensive care units with unknown consequences on patients’ mental health. The aim of this pilot study is to assess the feasibility of an early psychological evaluation and support in patients with COVID-19 hospitalized in intensive care unit (ICU) and describe their mental health outcomes during a 6-month follow-up.

**Patients and methods/materials and methods:** Psychological support was proposed to every COVID-19 survivor at invasive ventilation weaning or when conversation was feasible for patients receiving high-flow oxygen. Every patient was evaluated by a trained clinician psychologist during their hospitalization in ICU. Standardized evaluation occurred at day 7, week 6, 12 and 26 after ICU discharge with psychometric evaluation (HADS, PCL-5, ISI and SF-36 scales). Factors associated with insomnia, anxiety, depression and PTSD symptoms in follow-up were identified using linear mixed models.

**Results:** Thirty-eight patients were included (82% of men, mean age of 59 (± 11)). Three quarters of them (29/38) were intubated for an average length of 39 days (± 14). At discharge from hospital, 23/38 (60%) of them returned home while 15/38 (40%) went to rehabilitation centre. At day 7, 32 patients were evaluated: 5/32 (16%) had significant depression symptoms, 4/32 (13%) significant insomnia symptoms and 3/32 (9%) significant anxiety symptoms and at week 6, they were, respectively, 2/18 (11%), 3/18 (17%) and 2/18 (11%).At week 12, they were, respectively, 5/24 (20%), 4/24 (17%), 3/24 (13%), and 2/24 (8%) to have significant depression, insomnia, anxiety or PTSD symptoms and at week 26, respectively 2/19 (11%), 2/19 (11%), 4/19 (22%), and 1/19 (6%). History of sleep disturbances, length of sedation, length of stay in ICU, cumulative dose of sedative, of morphine and maximum dose of vasopressor were positively associated with insomnia and depression in follow-up.

**Conclusion:** In the context of emergency, the mental health dimension is often neglected. This study shows that, even in the context of a pandemic situation, it is possible to take care of the psychological aspects of critically ill patients. Without a control group, we cannot draw firm conclusions but critically ill COVID-19 patients in this cohort exhibit a lower frequency of psychiatric symptoms that what has been reported in hospitalized COVID-19 patients. Thus, early psychological assessment and support are possible in ICU and could be helpful for those patients.

**Compliance with ethics regulations:** Yes in clinical research.

### FC-181 Prevalence and risk factors of post-traumatic stress disorder in family member of intensive care unit patients

#### Chaouch Sabrina, Oussama Jaoued, Hajer Nouira, Wael Chamli, Rim Gharbi, Habiba Ben Sik Ali, Mohamed Fekih Hassen, Souheil Atrous

##### service de réanimation médicale hôpital Taher Sfar, Mahdia, Tunisie

**Correspondence:** Oussama Jaoued - oussamajaoued@gmail.com

*Annals of Intensive Care* 2021, **11(Suppl 1):**FC-181

**Rationale:** Having a family member hospitalized in the intensive care unit is a stressful event that can cause Post-traumatic stress disorder (PTSD), depression and anxiety. Many factors associated with PTSD were identified but these factors did not include reasons of intensive care admission. Aim: Prevalence and factors associated with post-traumatic stress disorder in family member of intensive care unit patients.

**Patients and methods:** This is a prospective analytic study. We included all family members of ICU patients with a duration of stay more than 3 days during 2020. The Arabic version of the Post-Traumatic Checklist Scale (PCLS) was used to identify family member with PTSD. Two groups were individualized. Group 1: family member with PTSD (defined as those with a total score of 50 or higher). Group 2: family member who did not meet the criteria for PTSD (those who scored lower than 50).

**Results:** Over the study period, 600 family members of patients admitted to the ICU were enrolled with a mean age of 43 ± 14 years. The family members were daughters in 22% of cases, sons in 19% of cases and sisters in 14% of cases. A Quarter of the family member had an academic level. The prevalence of PTSD was 38%. During this period, 119 patients were admitted to the ICU. Etiology of ICU admission was represented by acute respiratory failure in 56% of cases, coma in 17% of cases and shock in 15% of cases. Length of stay and the duration of invasive mechanical ventilation were significantly higher in group1 (21 ± 20 days vs. 16 ± 14 days (< 0.001) and 21 ± 17 days vs 16 ± 12 days (*p* = 0.01)). The mortality was significantly higher in group1 (36% vs. 16%, *p* < 0.001). ln multivariate analysis, factors independently associated with PTSD were: female gender of the family member (OR = 5.55; 95% IC (3.55–8.67) *p* < 0.001), non-invasive ventilation (OR = 1.664; 95%IC [1.027–2.695] *p* = 0.039), nosocomial infection(OR = 6, 17; 95%IC [1.73–22.009) *p* = 0.005) and delirium (OR = 4.1 95% IC % [5.06–33.18] *p* < 0.001).

**Conclusion:** Family members of patients admitted to the ICU are exposed to a high prevalence of PTSD. Higher risk of PTSD is common in female member family, family members of patients treated with non-invasive ventilation and family members of patient with nosocomial infection or delirium.

**Compliance with ethics regulations:** Yes in clinical research.

### FC-182 Post-intensive care syndrome as a burden for COVID ICU survivors at 6 months

#### Simon Butet, Félicie Belicard, Mélanie Cogne, Sébastien Cordillet, Yoann Launey, Jean-Marc Tadie, Isabelle Bonan

##### CHU Rennes, Rennes, France

**Correspondence:** Felicie Belicard - felicie.belicard@gmail.com

*Annals of Intensive Care* 2021, **11(Suppl 1):**FC-182

**Rationale:** Several studies have demonstrated that patients with COVID-19 associated ARDS were at risk of prolonged ICU stay along to ICU-acquired weakness (ICU-AW). Furthermore, patients hospitalized for severe form of COVID-19 are probably at risk for developing post intensive care syndrome. The objective of this cohort study was to describe the initial characteristics and the functional outcomes at 6 months after SARS-CoV-2 infection of the patients discharged from ICU according to their care course: discharge to Physical and Rehabilitation Medicine (PRM) Unit because of their dependence for activity of daily living, versus those who did not required rehabilitation’s care after ICU discharge.

**Patients and methods:** This study was conducted at the University Hospital of Rennes, France. All patients with COVID-19 needing ICU between March 1st 2020 and April 30st 2020 were included. Prior to ICU discharge, patients were screened for PRM eligibility. If they were insufficiently independent at ICU discharge, meaning a sub-score of transfer < 6 and/or of gait < 6 and/or of cognitive < 6 on the Functional Independence Measure (FIM) scale [1], patients were admitted to the PRM unit of this hospital (Group 1). If they were independent enough according to the FIM scale, they were discharged home from the hospital after a stay in a hospital ward (Group 2). Both groups were evaluated at 6 months according to the WHO Disability Assessment Schedule 12 items (WHODAS-12) [2].

**Results:** 74 patients were included. Among them, 5 (6.7%) died during ICU stay. 2 patients were admitted to another PRM facility with no data available. Thus, 67 patients were studied (36 patients in the group 1 and 31 patients in the group 2). The characteristics are shown in Table 1. The mean 6-month WHODAS-12 was 19.1 ± 31 in Group 1 and 8.9 ± 10.7 in Group 2, corresponding to a low level of participation’s restriction in both groups. Noteworthy, emotional affect due to their health condition was strongly impacted in both groups, with significantly lower scores in group 2. Psychological features of post intensive care syndrome show that group 2 patients have more difficulties to return to their previous mood since they experienced more anxiety, anger and trouble sleeping.

**Conclusion:** Patients admitted to ICU for severe form of COVID-19 necessitate physical rehabilitation care. Although recovery at 6 months appears satisfactory, mental and cognitive post-intensive care syndrome are frequent and should not be underestimated. Multidisciplinary care following ICU is needed to support COVID ARDS patients.

**References**

1. Ottenbacher KJ, Hsu Y, Granger CV, Fiedler RC. The reliability of the functional independence measure: a quantitative review. Arch Phys Med Rehabil. 1996;77(12):1226–32.
2. WHO| Developing the World Health Organization Disability Assessment Schedule 2.0. WHO. https://www.who.int/bulletin/volumes/88/11/09-067231/en/.

**Compliance with ethics regulations:** Yes in clinical research.
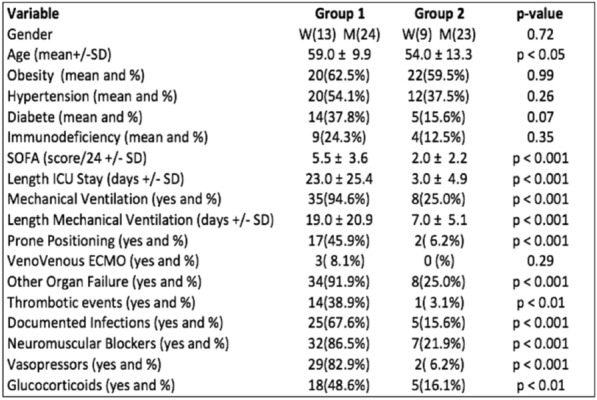


Table 1 Comparative characteristics of patients in Group 1 (ICU followed by PRM) and Group 2 (ICU without follow-up in PRM)

### FC-183 PICS detection in the COVID era: feasibility of a multimodal remote follow-up strategy with a smartphone web application

#### Charlotte Salmon, Youenn Jouan, Justine Cibron, Hélène Messet, Lionel Tchatat Wangueu, Piotr Szychowiak, Laetitia Bodet-Contentin, Stephan Ehrmann

##### CHRU de Tours, Tours, France

**Correspondence:** Charlotte Salmon- charlotte.salmon.gandonniere@gmail.com

*Annals of Intensive Care* 2021, **11(Suppl 1):**FC-183

**Rationale:** COVID-related ARDS is expected to induce a high post-intensive-care syndromes (PICS) incidence. Dedicated follow-up is recommended for early recognition and management of the different components of PICS. Implementation of a remote follow-up strategy, avoiding patients visits at the hospital as much as possible, may be relevant in the pandemic context with cross-contamination risks and pressure on the healthcare system.

**Patients and methods:** We implemented a multimodal follow-up strategy: combining teleconsultation and the development of a smartphone web application prompting patients to fill in questionnaires at home (screening for psychiatric and cognitive disorders, dyspnea, voice alteration, global mood and quality of life). Patients could request a teleconsultation through the web application. All patients discharged alive from a single ICU for COVID-19 pneumonia from 1st March 2020 to 31st December 2020 were assessed for remote follow-up, one month after ICU discharge. Initially, all patients were contacted; from July 2020, follow-up was focused on patients who underwent intubation or nasal high flow with FiO2 > 70%.

**Results:** 159 patients were assessed for follow-up in the period of interest. 22 patients could not be evaluated, and 43 did not meet the severity criteria. 94 patients were followed-up, at least once. 71 of those patients opened an account on the web application, of whom 40 filled in at least one questionnaire. 22 patients (55% of the participants using the web application) filled in several follow-up questionnaires over time. A total of 494 questionnaires were recorded and analyzed by the attending intensivist to guide follow-up. The median age was 63 years. 69 (73%) patients had been intubated. 52 patients (55%) did not recover their previous autonomy. All patients had at least one physical sequelae (dyspnea, dysphonia, functional limitation, etc.). 47% had a median walking distance below 1000 m. 65 (69%) had dyspnea (median mMRC score: 2). 32 (34%) reported pain. The median weight loss was 6 kg. 28 (30%) reported cognitive decline. 19 patients out of the 60 for whom psychiatric assessment was performed had psychiatric disorders (anxiety, depression, post-traumatic stress disorder. 64 specialized referrals were initiated for 36 patients, mostly physiotherapy, psychiatric, neuro-cognitive, pneumology and cardiology referral. In a satisfaction survey focusing on the web application, the large majority of them estimated the smartphone web application user friendly.

**Conclusion:** The feasibility of post-COVID remote follow-up based on teleconsultations and a dedicated web application for detection and management of PICS appeared promising given the large burden of post-COVID disease.

**Reference**

1. Carvalho-Schneider C, Laurent E, Lemaignen A, Beaufils E, Bourbao-Tournois C, Laribi S, et al. Follow-up of adults with noncritical COVID-19 two months after symptom onset. Clin Microbiol Infect. 2020.

**Compliance with ethics regulations:** Yes in clinical research.

### FC-184 Intensive care patients’ satisfaction

#### Imen Talik, Hedia Ben Ahmed, Fatma Essafi, Selma Essghaier, Moez Kaddour, Takoua Merhabene

##### hôpital régional Zaghouan, Zaghouan, Tunisie

**Correspondence:** Imen Talik - talik.imen2013@gmail.com

*Annals of Intensive Care* 2021, **11(Suppl 1):**FC-184

**Rationale:** Patients’ satisfaction is an important indicator for measuring quality of health care. The aim of our study was to assess determinants and level of patients’ perception for the intensive care unit (ICU) to create policies for improving health care system

**Patients and methods:** This study was performed in a 10-bed tertiary ICU. It was a prospective study enrolled between 1st July and 31st December 2020. We developed a local questionnaire that consisted of 34 questions in six domains (scientific level and origin of all patients, medical staff availability and quality of health care, pain management, hospital stay and discharge conditions). Answering possibilities ranged from 1 (very satisfied) to 5 (not concerned). All consecutive competent adult patients discharged after a stay in the Unit of over 24 h, and who agreed to participate in the project were enrolled; they were interviewed by phone or face to face. Patients with serious physical or mental pathologies, that could make the comprehension and completion of the questionnaire difficult, were excluded.

**Results:** During the study period 90 patients were enrolled (56% of all admission). Mean age was 52 ± 15 years [19–94] and sex ratio was 2.33. Majority of patients were admitted from the emergency room (47.8%) and 37.8% were admitted from the COVID unit. median (interquartile) length of hospital stay was 9 (5–15) days and all cases mortality rate was 37%. 63% of patients said they were very satisfied with quality of ICU care, and 32% satisfied. Patients were very satisfied of their relationships with medical staff (87.8%) and paramedical staff (81.1%). 83.3% of cases were satisfied about pain management. Hospital conditions as rooms cleanliness and comfort was very satisfactory in more than 70%. While satisfaction about hospital meals provided is less than 50%. 97.8% of patients recommended the intensive care unit to their entourage and they agree to return to intensive care unit if their state of health requires it in 93.3% of cases. There is no significant association between satisfaction level and patient characteristics, nor with the length of their stay.

**Conclusion:** Our study showed a good overall satisfaction. She revealed some shortcomings to be corrected quickly to ensure better patient care and install good practices.

**Compliance with ethics regulations:** Yes in clinical research.

### FC-185 What happens after a research project has been funded? Assessment of the scientific output following the award of a grant by the French Intensive Care Society

#### Bertrand Hermann^1^, Jean-Francois Llitjos^11^, Saber Davide Barbar^12^, Gwenaelle Jacq^6^, Laurent Poiroux^2^, Guillaume Decormeille^9^, Lamia Ouanes Besbes^10^, Toufik Kamel^7^, Kostas Bachoumas^8^, Nicholas Heming^4^, Gaël Piton^5^, Jean-Bapstiste Lascarrou^3^

##### ^1^Hôpital Européen Georges Pompidou, AP-HP, Paris, France; ^2^Centre Hospitalier Universitaire d’Angers, Angers, France; ^3^Centre Hospitalier Universitaire Nantes, Nantes, France; ^4^Hôpital Raymond-Poincaré, AP-HP, Garches, France; ^5^Centre Hospitalier Régional Universitaire de Besançon, Paris, France; ^6^Centre Hospitalier de Versailles André Mignot, Le Chesnay, France; ^7^Centre Hospitalier Régional d’Orléans, Orléans, France; ^8^Centre Hospitalier Départemental de Vendée, La Roche-Sur-Yon, France; ^9^Centre Hospitalier Universitaire de Toulouse, Toulouse, France; ^10^Hôpital Fattouma Bourguiba, Monastir, Tunisie; ^11^Gustave Roussy, Villejuif, France; ^12^Centre Hospitalier Universitaire de Nîmes, Nîmes, France

**Correspondence:** Bertrand Hermann - bertrand.hermann@aphp.fr

*Annals of Intensive Care* 2021, **11(Suppl 1):**FC-185

**Rationale:** Clinical and experimental research is costly and funding is key to the successfully conduct of academic projects. The French Intensive Care Society (FICS) offers several grants aimed at promoting high quality research in the field of intensive care. However, the scientific output following the award of a grant is not always known. We sought to determine the scientific productivity of grantholders funded by the FICS.

**Patients and methods/materials and methods:** The details of grant recipients and funded projects were obtained from a prospective database managed by the FICS. We contacted each grantholder through an online survey form (Google form) to collect data on the advancement of the funded project. Scientific output was defined as publication in a peer reviewed journal and/or abstract presentation in an international congress. Univariate and multivariate analyses were performed to investigate factors associated with scientific output.

**Results:** Between 2007 and 2019, the FICS awarded 104 grants to 95 recipients for a total amount of 1 572 500 euros. Recipients were mostly men (67%) of 34 [31–37] years of age. Most grants were awarded to recipients affiliated to public hospitals (94%) and universities (89%). Both clinical (58%) and experimental (48%) research projects were funded. Out of 86 (83%) recipients who answered the online survey form, 69 (80%) declared having produced scientific results, either through publication in a peer reviewed journal (54 (63%)) and/or through an abstract presentation in an international congress (52 (60%)). The majority of papers were published in high impact peer-reviewed journals (54% and 30% published, respectively, in the top 10% and top 25% journals within a discipline according to their impact factor). In a multivariate analysis, recipients affiliated to a university were more likely to achieve scientific valorization (12.0 [1.43–160], *p* = 0.033) while grants awarded between 2015 and 2019 were associated with less scientific output (0.15 [0.02–0.80], *p* = 0.042) as compared to years 2007–2010.

**Conclusion:** The FICS contributes to the funding of high quality clinical and experimental research projects, leading to publications in high-impact journals and abstract presentations in international congress.

**Compliance with ethics regulations:** N/A.

### FC-186 Iatrogenic events in intensive care unit: incidence, risk factors and impact on outcome

#### Imen Talik, Nejla Ben Slimen, Fatma Essafi, Boudour Ben Dhia, Moez Kaddour, Takoua Merhabene

##### hôpital régional Zaghouan, Zaghouan, Tunisie

**Correspondence:** Imen Talik - talik.imen2013@gmail.com

*Annals of Intensive Care* 2021, **11(Suppl 1):**FC-186

**Rationale:** Iatrogenic pathology is an important marker of health care quality. Iatrogenic events (IEs) are defined as an adverse event that occurs independently of the underlying disease. Mortality is reported to be as high as 13.6% in cohorts of hospitalized patients experiencing IEs. Several study reported that both length of stay (LOS) and cost of hospitalization are increased by IEs occurrence. Aim: to assess incidence and types of IEs in ICU, associated risk factors and impact on mortality rate.

**Patients and methods/materials and methods:** All patients hospitalized more than 24 h in the 10-bed ICU of a Tertiary hospital between the 1st January and 31st December 2020 were included. Collected parameters were: demographic characteristics, severity scores (SAPS II, APACHE II) on admission, reasons for hospitalization, iatrogenic events associated with procedure and care, need and duration for mechanical ventilation, LOS and mortality.

**Results:** Tow hundred ninety-nine patients were hospitalized during the study period. Mean age was 55 ± 17 years, sex ratio = 2, SAPS II = 31 ± 15, APACHE II = 12 ± 9 and LOS = 6 days [3–13]. Fifty-six per cent needed mechanical ventilation. Incidence of IEs was 57.85% with an incidence density of 83 for 1000 days-patients. IEs occurred in a median delay of 6 days [3–11], it was considered preventable in 39% of cases and severity was considered major in 35 cases. Patients with IEs were significantly severe on admission, with a longer duration of MV and LOS. Global mortality was 43%. Dead patients were significantly severe on admission and experienced more IEs than survivors. OMEGA score, duration of MV and LOS were significantly higher in the non-survivor group. In multivariate analysis, IEs were not independent factors of mortality (OR 1.38 IC 95% [0.682–2.807].

**Conclusion:** IEs are frequent, principal identified risk factors were severity on admission and multiplicity of ICU invasive supports. Despite its high frequency, our study showed that IEs had no impact on ICU mortality.

**Compliance with ethics regulations:** Yes in clinical research.

### FC-187 PICU for adults during the Covid-19 pandemic: a little help for intensivists, a big challenge for pediatricians

#### Maxence Fernandes, Charlie De Melo, Dominique Astruc, Anne-Sophie Guilbert

##### CHU de Strasbourg, Strasbourg, France

**Correspondence:** Maxence Fernandes - maxence.fernandes@chru-strasbourg.fr

*Annals of Intensive Care* 2021, **11(Suppl 1):**FC-187

**Rationale:** The Sars-Cov2 pandemic is without any doubt one of the greatest challenges any healthcare system has ever faced. During the first wave in March 2020, adult ICUs were quickly overwhelmed with a new and still unknown disease. While a large number of new Covid-19 units were simultaneously opening, Paediatric ICUs were not as deeply affected. As the situation worsened, we were asked to repurpose our unit for adult patients. This study aims at two objectives: describing the adult population hospitalized in our Paediatric Intensive Care Unit during the first wave of Sars-Cov 2, and the organization and challenges it required.

**Patients and methods/materials and methods:** We report characteristics of all patients we admitted in our PICU from March 21st to April 15th 2020 and the unit’s organization.

**Results:** 13 patients were admitted, median age was 68 years; 11 were men; 15% were active smokers, 46% were obese and 54% had known arterial hypertension. 2 patients died in our unit. All had severe ARDS, 69% required mechanical ventilation at admission, and 45% needed prone position. The median duration of mechanical ventilation was 16 days with 36% extubating failure rate. 5 patients died before hospital discharge. The most common adverse outcomes were the presence of nonspecific circulant anticoagulant (73%), nosocomial infection like centra line-associated infection and ventilator-associated pneumonias (62%) and multiple organ failure (46%). High-flow nasal cannula was used in 54% of our patients and corticosteroid as respiratory rescue for 38% before it became the accepted official recommendations. Concerning the organization, the medical day-to-day activity was accomplished by three of our usual physicians, 3 paediatric anaesthetists and 2 adult anaesthetists. Only 4 doctors participated in nightshifts. Up to 6 residents took part in the unit activity. The paramedical staff was composed of 5 nurses, day or night.

**Conclusion:** From March 21st to April 15th 2020, we treated up to 13 adult patients with severe Covid-19 infections in our PICU. Adult patient care was only possible thanks to the cooperation of volunteering actors at all level. Allowing the team to stay in a familiar setting seemed to facilitate quick adaptation to adult patients care. This unprecedented pandemic situation also revealed to be an opportunity to improve our skills, on theoretical and practical levels. While this activity proved to be physically and psychologically draining, it brought much needed support to our colleagues from adult ICUs.

**Compliance with ethics regulations:** Yes in clinical research

### FC-188 Transfer by sanitary train of serious COVID patients from Paris to west brittany a retrospective descriptive study

#### Pierre Kergoat, Mikael Moriconi

##### CHIC Quimper, Quimper, France

**Correspondence:** Pierre Kergoat - pierre.kergoat@gmail.com

*Annals of Intensive Care* 2021, **11(Suppl 1):**FC-188

**Rationale:** In 2020, the French Health care System was overwhelmed by the outbreak of the SARS-COV-2 pandemic leading to a shortage of Intensive Care Beds in Ile de France hospitals (Paris Area). A tremendous amount of critically ill patients had to be transferred from these hospitals to less overloaded units in a short time interval. This exceptional situation needed an exceptional answer and Sanitary Trains were chosen by the authorities as a mean of transport. We conducted a descriptive observational study in order to assess the safety and the consequences of this kind of transfer.

**Patients and methods/materials and methods:** The main objective was to study this particular population’s mortality. The secondary objectives were to evaluate the clinical tolerance of the transfers and complications during the ICU stay. Retrospective observational study. Data were collected from the departure of their former units until their new ICU discharge.

**Results:** In the beginning of April 2020, 51 patients were transferred to Western Brittany and 49 were included in the study. Five patients died (10.2%) and 19 patients (38.8%) worsened during the transfer (use of prone position within 24 h after arrival or a significant decrease of P/F ratio). After multivariate analysis we did not identify any factor related to mortality or worsening during the transfer. Main complications were: nosocomial infections (36/49; 73.5%), the need for renal replacement therapy (12/49; 24.5%), circulatory failure (16/49; 32.7%) and thromboembolic events (13/49; 26.5%).

**Discussion:** Mortality rate appears lower than others descriptive studies. This rate may be explained through various factors: age, co-morbidities and patient’s selection. However, a substantial number of patients worsened during the transfer. The risk benefit balance must be assessed.

**Conclusion:** The mortality of these critically ill COVID -19 patients, transferred through railway, is low. However, morbidity associated with transport seems to be important and should be investigated in further studies.

**Compliance with ethics regulations:** Yes in clinical research

### FC-189 Criteria deemed important by the ICU patients for designating a reference person

#### Nicolas Meunier-Beillard^4,5^, Elea Ksiazek^4^, Caroline Abdulmalak^6^, Samia Berrichi^7^, Hervé Devilliers^1^, Fiona Ecarnot^8^, Audrey Large^1^, Jean-Baptiste Roudaut^1^, Auguste Dargent^1^, Jean-Philippe Rigaud^7,9^, Jean-Pierre Quenot^1,2,3^

##### ^1^CHU Dijon, Dijon, France; ^2^Lipness Team, INSERM Research Centre LNC-UMR1231, Dijon, France; ^3^LabEx LipSTIC, University of Burgundy, Dijon, France; ^4^INSERM CIC 1432, Clinical Epidemiology, University of Burgundy, Dijon, France; ^5^DRCI, USMR, CHU Dijon Bourgogne, Dijon, France; ^6^Centre Hospitalier William Morey, Châlon Sur Saône, France; ^7^Centre Hospitalier de Dieppe, Dieppe, France; ^8^University Hospital Besancon, Besancon, France; ^9^Espace de Réflexion Ethique de Normandie, University Hospital, Caen, France

**Correspondence:** Jean-Pierre Quenot - jean-pierre.quenot@chu-dijon.fr

*Annals of Intensive Care* 2021, **11(Suppl 1):**FC-189

**Rationale:** We investigated the criteria that patients deem important for choosing the reference person best qualified to interact with the caregiving staff

**Patients and methods/materials and methods:** Exploratory, observational, prospective, multicentre study between 1st March and 31st October 2018 in 2 intensive care units (ICUs). A 12-item questionnaire was given to patients hospitalized in the ICU at admission. Patients were eligible if they understood French and if they had not appointed an official surrogate prior to ICU admission. Responses were given on a scale of 0 (criterion not important at all) to 10 (extremely important criterion). In order to be considered as “important”, an item had to receive an average score > 7.

**Results:** In total, 71 patients completed the questionnaire, average age was 64 ± 17 years, 70% were males. Average SAPSII score was 39.8 ± 16.5 and average Charlson comorbidity index was 2.5 ± 2.4. The items that were classed by the respondents as being the most important attributes for a reference person were the following: good knowledge of the patient’s wishes and values; having an emotional attachment to the patient; being a family member; and having an adequate understanding of the clinical status and clinical history. On average, women placed more importance than men did (*β* = 0.61; 95%CI 0.02; 1.21]) on the need for the surrogate to have good knowledge of the medical situation and the patient’s wishes.

**Conclusion:** This study identifies the attributes considered by patients to be most important for designating a reference person to interact with the ICU staff.

**Compliance with ethics regulations:** Yes in clinical research.

### FC-190 COVID-19 as a springboard for developing ICU

#### Marion Roux^1^, Ricardo Jose Rey Velarde^4^, Cyrille Venet^1^, Marta Clea Otero Domínguez^4^, Albrice Levrat^2^, Nerea Benitez Garcìa^4^, Robin Sejournant^3^, Virginia González Cordero^4^, Jérome Normand^1^, David Pestana^4^, Nicolas Terzi^1^, Lucia Valencia^4^, Boubou Camara^3^, Silvia Calvino-Gunther^1^, Abdou Salam Gueye^3^, Florian Sigaud^1^

##### ^1^CHU Grenoble Alpes, Grenoble, France; ^2^Centre Hospitalier Annecy-Genevois, Annecy, France; ^3^Organisation Mondiale de la Santé, Nouackchott, Mauritanie; ^4^ Hospital Universitario Ramón y Cajal, Madrid, Espagne

**Correspondence:** Nicolas TERZI - nterzi@chu-grenoble.fr

*Annals of Intensive Care* 2021, **11(Suppl 1):**FC-190

**Rationale:** COVID-19 pandemic spreads worldwide for more than a year. Beside the strongest health systems, many countries don’t have material and human resources to take care of the most severe patients. Mauritania is a western African country that counts 4 million inhabitants with only 17 physicians actually trained to anesthesiology and intensive care for the whole country. Starting in October 2020 Mauritanian health ministry and World Health Organization sent an international group of Spanish and French nurses and intensivists to evaluate the needs, organize and implement an adapted unit in order to face the crisis and share their experience in intensive care. This mission was supported by the French Society of Intensive Care (FICS).

**Patients and methods/materials and methods:** Because of structural difficulties as lack of reliable oxygen supply in main hospitals, it was decided to identify a new unit in one of the hospitals of the capital. Indeed a functional and fully armed unit of six ICU beds has emerged closed to a 6-bed conventional COVID-dedicated ward. This strategy issued from local and international cooperations and supported by Health Ministry and WHO was permitted by funding and donations. The second goal of the mission was to train, develop and emulate intensive care culture among health care local providers. Physicians, medical students and nurses received lectures and practical trainings in basics ICU from WHO consultants with impressive improving daily practices.

**Results:** The first COVID-19 patient was admitted on 2020/12/31 for invasive mechanical ventilation and ARDS management. Until 15th February 45 patients have been admitted and treated according to best practices and international guidelines. Mechanically ventilated ill patients’ mortality was ranging up to 100% before the intervention of the task force and is decreasing to 60%. Some patients have already been discharged and returned home. 120 h of lectures have been performed.

**Discussion:** Nevertheless, there are still many challenges to address to sustain a culture of modern intensive care in Mauritania after COVID crisis. Stronger political commitment seems to be necessary in order to warrant logistical supply and the training of dedicated staff. FICS teams of University Hospital of Grenoble, Annecy (France) and Las Palmas (Spain) are uniting their efforts to pursue the support in the area of Mauritanian staff mentoring. Next step of the partnership includes webinars programs for training and staff exchanges to continue sharing experiences in the coming years

**Conclusion:** COVID-19 pandemia has highlighted ressources and competencies discrepancies triggering new areas of cooperation network.

**Compliance with ethics regulations:** N/A.
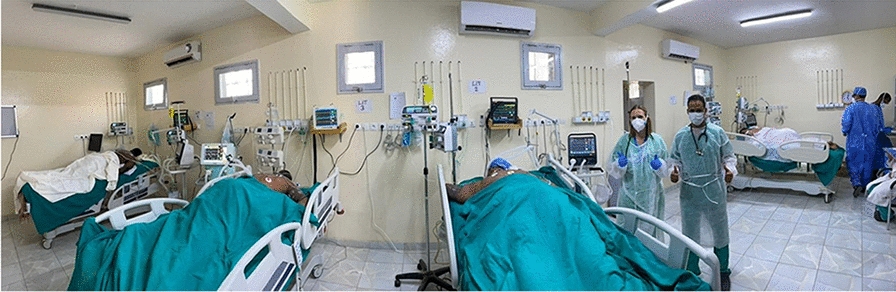


ICU unit

### FC-191 Intensive care unit-acquired urinary tract infections

#### Rania Ammar, Farah Zouari, Kamilia Chtara, Saba Makni, Chokri Ben Hamida, Mabrouk Bahloul, Mounir Bouaziz

##### faculty of medecine of Sfax,university of Sfax, Sfax, Tunisie

**Correspondence:** Rania Ammar - rania.ammarzayani@gmail.com

*Annals of Intensive Care* 2021, **11(Suppl 1):**FC-191

**Rationale:** To describe the microbiology for acquired urinary tract infections (UTIs) and antibiotic sensitivity.

**Patients and methods/materials and methods:** We conducted a prospective study over 3 months in a Tunisian intensive care unit. We included patients with (UTIs). UTIs were defined as at least 105 colony-forming units/ml of one or two organisms 48 h or more after ICU admission.

**Results:** We identify 40 episodes of UTIs in 31 patients. Mean (SD) age of patients was 43.68 ± 20.54. Mean (SD) ICU stay was 36.42 ± 17.710. Mean delay of UTIs was 18.54 ± 12.37. Mean (SD) bladder catheter length was 35.10 ± 17.05. The most common organisms isolated were Klebsiella pneumoniae in 8 patients (20%) following by Proteus mirabilis and Candida spp in 6 patients (15%). From the total of 11 isolates, Klebsiella pneumoniae was resistant to carbapenems in 4 episodes, resistant to ceftriaxone in 7 episodes. Pseudomonas aeruginosa were resistant to Ceftazidime and to carbapenems in 1 episode. Proteus mirabilis were resistant to ceftriaxone and to carbapenems (3 episodes). Acinetobacter baumannii were resistant to carbapenems in 3 episodes. Enterococcus faecium was vancomycin-resistant in 1 episode. The most frequent complication was septic choc in 3 patients (9.67%). The mortality rate was 29% (9 patients).

**Conclusion:** Development of an ICU-acquired UTI is common in critically ill patients is frequent with development of antibiotic resistance.

**Compliance with ethics regulations:** N/A

### FC-192 Risk factors for acquisition of Acinetobacter baumannii infections in the intensive care unit (ICU)

#### Khalid Khaleq, Khalil Aalloula, Mohamed Aziz Bouhouri, Driss Hamoudi, Rachid Harrar, Khalid Zerouali

##### CHU, Casablanca, Maroc

**Correspondence:** Khalid Khaleq - khaleq 20@gmx.fr

*Annals of Intensive Care* 2021, **11(Suppl 1):**FC-192

**Rationale:** Objective: To determine the risk factors associated with the acquisition of Acinetobacter (A) baumannii infections in the ICU.

**Patients and methods/materials and methods:** This is a retrospective case–control study involving patients admitted to the ICU of the IBN ROCHD Teaching Hospital from January 2016 to December 2019. Risk factors were determined using multiple logistic regressions.

**Results:** The study included 288 patients; 144 cases and 144 controls. 67.01% were males and the average age was 46.68. The prevalence and mortality rates were 10.3% and 41.66%, respectively. Mortality rate was higher in cases than in controls (56.94% versus 26.39%) Multivariate analysis showed that central venous catheter (OR = 0.543; IC95% = 0.366–0.805), prolonged ICU stay (OR = 4.274; IC95% = 1.32–12.078), prolonged duration of invasive procedures (OR = 1.15; IC95% = 0.327–4.049), mechanical ventilation (OR = 2.085; IC95% = 1.632–4.820), immunodepression (OR = 2.507; IC95% = 1.308–3.483), the use of several antibiotics (OR = 4.865; IC95% = 2.876–10.543), prior administration of amikacin (OR = 3.556; IC95% = 1.185–6.141) and imipenem (OR = 13.257; IC95% = 5.968–22.312), the use of corticosteroids (OR = 6.865; IC95% = 4.273–10.543), recent surgery (OR = 0.781; IC95% = 0.612–0.996) are strongly linked to the acquisition of A. baumannii infections in the ICU. Among the tested antibiotics, colistin was the most effective with a susceptibility rate of 99%.

**Conclusion:** The use of central venous catheters and mechanical ventilation, prolonged duration of invasive procedures, prolonged stay in the ICU, prior use of imipenem and amikacin, immunodepression, use of several antibiotics, recent surgery and the use of corticosteroids favor the acquisition of *A. baumannii* infections. In light of these results, the reinforcement of infection control measures especially with regard to patients at high risk remains paramount.

**Compliance with ethics regulations:** N/A.

### FC-193 Carrying for multi-resistant bacteria in the ICU: prevalence, risk factors and outcome

#### Emna Rachdi, Samia Ayed, Rim Jameli, Amira Jamoussi, Fatma Jarraya, Jalila Ben Khelil

##### Hopital Abderrahman Mami, Ariana, Tunisie

**Correspondence:** Emna Rachdi - e.rachdi@yahoo.fr

*Annals of Intensive Care* 2021, **11(Suppl 1):**FC-193

**Rationale:** The problem related to multidrug-resistant bacteria (MDR) is its occurrence in intensive care units, where antibiotics are widely prescribed. The persistence of colonization with MDR in patients may leads to their spread. The purposes of this study were to describe the prevalence of MDR carriage on admission and during the stay in the intensive care unit (ICU) and to analyze their risk factors and their impact on the outcome.

**Patients and methods/materials and methods:** It was a cross-sectional, descriptive and retrospective study, including 200 patients who were hospitalized in the ICU at Aberrahman Mami Hospital, during the year 2019. Sociodemographic data, clinical, biological and outcome data were collected.

**Results:** The included patients had an average age of 55 years and a gender ratio of 1.29. They were hospitalized in ICU after an average stay of 2.6 days mainly in the emergency department (58.5%). The reason for hospitalization was respiratory in 74% of cases. Screening for MDR on admission was done only in 38 cases and was positive in 29% of cases. MDR was detected in both rectal and nasal sites in 4 cases. The most frequently isolated bacteria was Klebsiella pneumoniae in 42% of cases (broad spectrum betalactamase producer in 4 cases and carbapenemase producer in 2 cases). During hospitalization, nasal and rectal swab was done in only 3 cases. Univariate analysis comparing the group of patients with positive MDR carriage and those without any, showed that recent surgery and history of neoplasia were significantly more frequent in the first group. In multivariate analysis, no independent risk factor for BMR carriage was identified. MDR carriage was associated with a significant increase in mortality (*p* = 0.034) in univariate analysis. In multivariate analysis, MDR carriage was an independent predictor of death (*p* = 0.038).

**Conclusion:** Screening for MDR on admission and during hospitalization in the intensive care unit remains insufficient, due to the logistical conditions of the hospital. MDR carriage is an independent risk factor for mortality in intensive care.

**Compliance with ethics regulations:** Yes in clinical research.

### FC-194 Epidemiology and profile of pathogens in an intensive care unit

#### Iliass Ennour Idrissi, Fahd Moussaid, Fouzia Douirek, Amra Ziadi, Mohamed Abdennaceur Samkaoui

##### CHU mohamed VI marrakech, Marrakech, Maroc

**Correspondence:** Iliass Ennour Idrissi - ennouridrissiiliass@gmail.com

*Annals of Intensive Care* 2021, **11(Suppl 1):**FC-194

**Rationale:** Incidence of nosocomial infections is high in intensive care units (ICU) and is associated with increased morbidity and mortality. Understanding the epidemiology of microorganisms in ICU is important for effective antimicrobial therapy. The aim of this study was to determine the profile of pathogens isolated in an intensive care unit of a major urban city among patients that presented a nosocomial infection during their hospitalisation.

**Patients and methods/materials and methods:** A retrospective study was conducted among consecutive patients admitted for more than 48 h to ICU during a 6 months period between July 2020 and January 2021 and that presented a hospital-acquired infection. Socio-demographic, clinical, bacteriological and follow-up data were extracted from medical records.

**Results:** During the study period, 34 patients developed a nosocomial infection during hospitalisation in ICU. Mean age of patients was 42.3 ± 18.3 years (range: 18.0–86.0 years) and 68% were males. Patients were mainly from an urban area (91%) and the majority were transferred from emergency department (88%). The main diagnoses were multiple trauma injury (47%) and thermal burns (18%). In terms of comorbidities, 9% of the patients presented diabetes. A septic shock appeared in 74% of the patients and the mean SOFA Score was 5.21 ± 2.29 (range: 0–13). The most common types of infection were central line-associated bloodstream infections (38%), bloodstream infections (35%), ventilator-associated pneumonia (32%), urinary catheter-related infections (29%), and soft tissue infections (21%). The most frequently isolated pathogens were *Acinetobacter baumannii* (25%), *Klebsiella Spp*.(12%), *Pseudomonas aeruginosa* (8%), *coagulase negative Staphylococcus aureus* (6%), *E. coli* (6%), *Providencia Spp.* (6%), *Enterococcus faecalis* (6%) and *Raoultella Terrigena* (4%). The mortality rate among those patients was 68% after a mean duration of stay of 20.4 ± 12.7 (range: 3.0–44 days).

**Conclusion:** ICU infections are common and often associated with invasive ICU manoeuvers. Gram-negative bacilli and polymicrobial infections were the most common cause of infection. Some uncommon microorganisms were encountered and require particular attention.

**Compliance with ethics regulations:** Yes in clinical research.

### FC-195 Is there a benefit of systematic arterial and central venous catheters tip culture in intensive care?

#### Jihene Guissouma, Amenne Alouini, Hana Ben Ali, Habib Brahmi, Mohamed Samet, Hatem Ghadhoune

##### univesité Elmanar Faculté de médecine de Tunis. Hôpital universtaire Habib Bougatfa Bizerte Tunisie, Bizerte, Tunisie

**Correspondence:** Hatem Ghadhoune - ghadhoune@yahoo.fr

*Annals of Intensive Care* 2021, **11(Suppl 1):**FC-195

**Rationale:** Catheter-related infection is a common complication in the intensive care unit. It increases the morbidity and mortality of patients in intensive care. The usefulness of systematically culturing all central arterial and venous catheters in intensive care settings is a controversial topic. The aim of this study was to assess the value of the systematic culture of all arterial and central venous catheters in intensive care as well as its impact on patient management.

**Patients and methods/materials and methods:** This study was prospective, descriptive and cross-sectional, from January 1, 2020 to June 30, 2020, including 86 catheters placed in patients hospitalized in a Tunisian medical intensive care unit

**Results:** This research covered a period of 6 months. It consisted of the study of 86 catheters in 18 patients. The mean age of the patients was 52.83 years with a minimum of 17 years and a maximum of 73 years. There was no significant difference in terms of the rate of suspected infection linked to the catheter, whether or not the catheter was placed during a night shift. In febrile patients, defervescence following a catheter ablation for a suspected catheter-related infection is significant compared to a defervescence following a routine catheter ablation. The majority of changes in antibiotic therapy after a catheter removal were due to suspected catheter-related infection. There was no significant difference in the positivity of catheter tip cultures between systematically ablated catheters and those ablated due to suspected catheter-related infection. The positivity of the central blood culture of the ablated catheter following a suspected catheter-related infection compared to that of a systematically ablated catheter was significant.

**Conclusion:** These results were against the systematic catheter tip culture. It also generates an additional cost and an increase in the workload. Indeed, in the majority of cases where the catheters were systematically ablated, even when the samples of the latter came back positive, the management of the patients remained unchanged. These results were in line with those found in the literature.

**Compliance with ethics regulations:** Yes in clinical research

### FC-196 Acute renal failure in postoperative sepsis

#### Khalid Khaleq, Meryem Baqloul, Mohamed Aziz Bouhouri, Driss Hamoudi, Rachid Harrar

##### CHU, Casablanca, Maroc

**Correspondence:** Khalid Khaleq - khaleq20@gmx.fr

*Annals of Intensive Care* 2021, **11(Suppl 1):**FC-196

**Rationale:** Acute renal failure is a frequent complication in resuscitation, mostly observed in septic conditions. Despite numerous technical innovations, mortality remains significant. The aim of this work is to study the incidence of acute renal failure in postoperative sepsis, its various epidemiological aspects as well as to evaluate the predictors of mortality of acute renal failure in postoperative sepsis.

**Patients and methods/materials and methods:** We conducted a two-and-a-half year descriptive and analytical retrospective study (between January 2018 and June 2020) on 70 cases of acute renal failure in post-operative sepsis, hospitalized at the P33 surgical emergency resuscitation department of IBN ROCHD University Hospital in Casablanca. Adult patients with normal pre-operative renal function who developed a complicated septic condition of acute renal failure were included in our study. The parameters studied are demographic, clinical, para-clinical, therapeutic and evolutionary data. Statistical analysis was carried out using the SPSS software, the evaluation of prognostic factors used univariate and multivariate analysis.

**Results:** The incidence of acute renal failure in our work was 9.15%. The average age of patients was 58 ± 14 years with a sex ratio of 2.5. The most common risk factors were: field-related factors (male gender, diabetes, chronic high blood pressure, smoking, nephrotoxic drugs) related to initial surgery (emergency surgery, intra-abdominal surgery site). Clinical signs were dominated by tachycardia (55.71%) polypnea (30%) hypotension (31.42%) consciousness disorder (20%), fever (24.28%), oligo-anuria (24.28%), 75.71% of the patients had septic shock and 24.28% severe sepsis, hemodynamic, hepatocellular and hematological failure were the most common. 54% of our patients were Class 3, 11.43% Stage 2 and 34.28% Stage 1 of KDIGO (Kidney Disease Improving Global Outcomes) the organic nature of acute renal failure was the majority with more than 85.71%, 21.42% of patients benefited from extracorporeal purification. The mortality rate was 81.43%, the prognostic factors emerged in multivariate analysis are: the history of high blood pressure, the history of diabetes, the presence of consciousness disorder, and arterial hypotension, hyperkalemia, the time of onset of acute renal failure greater than 48H

**Conclusion:** The occurrence of acute renal failure in sepsis in the midst of surgical resuscitation is a critical stage with a very pejorative prognostic significance. A better knowledge of its risk and prognostic factors is fundamental for a more effective management.

**Compliance with ethics regulations:** N/A.

### FC-197 Ventilator associated pneumonia in COVID-19 patients with acute respiratory distress syndrome: a retrospective study

#### Jeanloup Augy, Segolene Gendreau, Bertrand Hermann, Caroline Hauw-Berlemont, Nicolas Peron, Emmanuel Guerot, Francesca Santi, Jean-Yves Fagon, Jean-Luc Diehl, Romy Younan, Nadia Aissaoui, Ana Novara

##### HEGP, Paris, France

**Correspondence:** Jeanloup Augy - augyjeanloup@hotmail.com

*Annals of Intensive Care* 2021, **11(Suppl 1):**FC-197

**Rationale:** Recent studies reported an increased risk of ventilator associated pneumoniae (VAP) in case of COVID-19 that may be not fully explained by ventilation time. Previous studies reported that corticosteroids now recommended in COVID pneumonia may increase risk of VAP. We reported the epidemiology of VAP occurring in COVID-19 patients admitted in intensive-care unit (ICU) with acute respiratory distress syndrome (ARDS) without glucocorticosteroids nor immunomodulatory drugs.

**Patients and methods/materials and methods:** We conducted a retrospective monocentric cohort study comparing the COVID group, including all consecutive adult patients admitted in the ICU with COVID-19-related ARDS between 15 March to 31 April 2020, with a historical control group, including all consecutive patients admitted with all cause ARDS between 1st January to 31 December 2019. Exclusion criteria for both groups were: ICU stay of less than 24 h, admission from another hospital after 48 h of mechanical ventilation, corticosteroids or experimental drugs received during intensive-care hospitalization. The primary endpoint was the density of VAP. Secondary endpoints were ventilation time to VAP occurrence, number of VAP episodes, type and resistance of bacteria evidenced by microbiological samples.

**Results:** Fifty-one patients in the COVID group were compared to 48 patients in the control group. Incidence of VAP was 44.0/1000 ventilation days in COVID group vs 20.6/1000 ventilation days, *p* = 0.03. First VAP in COVID group occurred after 7.0 days of IMV [6.0–10.3] vs 11.0 days [7.75–12.50] *p* = 0.10. Five of the 28 VAP (18%) were early onset VAP in the COVID-group vs 1/8 (12%) in the control group, *p* = 0.7. Nine patients had a second episode of VAP (8 (16%) patients in COVID group vs 1(2%) patients, *p* = 0.019). Four patients had a third episode of VAP (3 (6%) patients in COVID group vs 1(2%) patients, *p* = 0.337. Microbiological findings did not differ between the two groups, see Table 1. In the multivariate Fine and Gray competitive risk proportional hazards model, only COVID infection was associated with VAP (adjusted OR 4.24 95% CI: 1.79–10.04, *p* < 0.001).

**Conclusion:** Amongst patients presenting with ARDS in the ICU, VAP were more frequent in COVID-19 without corticosteroids or immunomodulatory drugs than in ARDS of other causes, but as microbiological ecology seems to be like non-COVID-19 ARDS similar empirical antibiotics could be used.

**Compliance with ethics regulations:** Yes in clinical research.
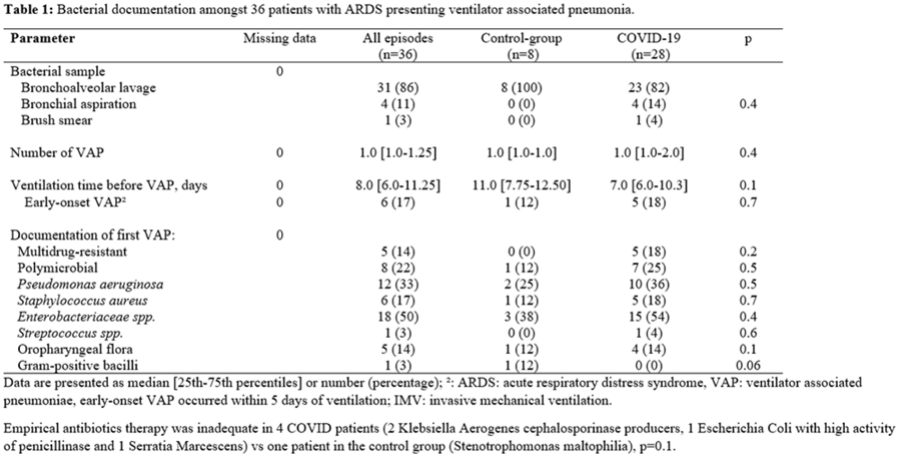


Table 1: Bacterial documentation

### FC-198 Is coronavirus disease 2019 (COVID-19) associated with emergence of nosocomial infection?

#### Boudour Ben Dhia, Imen Talik, Najla Ben Slimen, Fatma Essafi, Moez Kaddour, Takoua Merhabene

##### hôpital régional Zaghouan, Zaghouan, Tunisie

**Correspondence:** Imen Talik - talik.imen2013@gmail.com

*Annals of Intensive Care* 2021, **11(Suppl 1):**FC-198

**Rationale:** A large percentage of patients hospitalized for SARS-CoV-2 infection are admitted to the intensive care unit (ICU) and are thereby at significant risk of developing nosocomial infections (NI). Aim: To assess characteristics and outcome of ICU-acquired infections in severe COVID-19 patients.

**Patients and methods/materials and methods:** We conducted a retrospective study including all patients hospitalized in the ICU of a tertiary hospital, with a confirmed diagnosis of COVID-19, from 23 th March 2020 to 31st January 2021. We reported epidemiological, clinical and microbiological features, and outcome of ICU-acquired infections.

**Results:** We analyzed 121 patients admitted with acute respiratory failure due to COVID-19. Median age was 63 ± 12 years (IQR, 56–70), sex ratio = 1.5, Medians of SAPS II and APACHE II scores were, respectively, 26 ± 8 and 8 ± 3.8. Hypertension (48%) and diabetes (35%) were the most frequent underlying diseases. Fifty patients (41%) required invasive mechanical ventilation. All patients have received corticosteroids, azithromycin and beta-lactam at admission. We recorded a total of 59 episodes of confirmed NI occurring in 48 patients (40%) during ICU stay (7 patients have had 2 different episodes of NI and two patients have developed 3). NI occured in the majority of cases in ventilated patients (42%) especially those requiring invasive mechanical ventilation (76%). The median time to the onset of the first nosocomial infection was 7.5 ± 3 days (IQR 3–13). We observed 47 episodes of ventilator associated pneumonia (VAP), 8 urinary tract infections (UTI), 2 episodes of primary bloodstream infections (BSI) and 1 catheter-related bloodstream infection (CRBSI). VAP-causative pathogens were not documented in 22 cases (47%). Gram-negative bacilli were responsible for a large proportion of NI. Pathogens isolated were, respectively: *Acinetobacter baumannii n* = 8 (17%), followed by Pseudomonas aeruginosa *n* = 7 (15%) and *Klebsiella pneumoniae n* = 6 (13%). Candida albicans were isolated in 3 cases of UTI (6%). Occurrence of NI was significantly associated with longer length of ICU stay (13.9 ± 5.5 vs 7 ± 4 days, *p* < 0.05) and duration of invasive mechanical ventilation (8.5 ± 4.2 vs 4.5 ± 2.4 day, *p* = 0.003). ICU mortality in IN group was significantly higher (73% vs 24.7%, *p* < 10-3).

**Conclusion:** Our study showed that NI is a common complication of ICU admission in patients with severe COVID-19, it was associated with higher mortality and longer ICU length of stay.

**Compliance with ethics regulations:** Yes in clinical research.

### FC-199 High prevalence of unusual non-fermenting Gram-negative bacteria in mechanically ventilated COVID-19 patients

#### Laurent Camous^1^, Frederic Martino^1^, Amael Ouassou^1^, Jean-David Pommier^1,2^, Michel Carles^1^

##### ^1^CHU de guadeloupe, Les Abymes, France; ^2^institut pasteur guadeloupe, Les Abymes, France

**Correspondence:** Laurent Camous - laurent.camous@chu-guadeloupe.fr

*Annals of Intensive Care* 2021, **11(Suppl 1):**FC-199

**Rationale:** Recently, large data of Ventilator Associated pneumonia (VAP) in COVID-19 patients have been described. Interestingly, it was shown that late VAP were mostly secondary due to non-fermenting bacteria. Antibiotic management and diagnosis procedures should be conducted cautiously in that context. Previous reports have shown that VAP was reported in 11% to 37% of the mechanically ventilated patients with interestingly unusual emerging new pathogens.

**Patients and methods/materials and methods:** During the first wave of the COVID outbreak, all consecutive mechanically ventilated patients in our 36 beds unit were included in a prospective study of VAP. Late VAP were defined as a pneumonia diagnosed after 5 days under mechanical ventilation and diagnosed by bacterial cultures of blind distal protected aspirate (BPA).

**Results:** In our single-centre experience, 20% (5/21) of our Covid-19 patients developed an Achromobacter xylosoxidans (Ax) VAP among which 3/5 (60%) with concomitant bacteremia. Patients characteristics at admission and at VAP diagnosis are summarized in Fig. 1. Data on Ax cases were compared to all previous ICU Ax infections diagnosed in our unit in the last 5 years.

**Discussion:** Ax is a non-fermenting Gram-negative rod that mainly causes healthcare-associated infections and is particularly adapted to tropical climate and endemic in the West Indies. Most described cases are bacteremia in patients with immunosuppression. Primary bacteremia represents the most common clinical presentation. VAP due to Ax have been described in trauma patients with a low mortality rate. The clinical impact of Ax infection reported in the literature is not clear and is usually found associated to various bacteria (Pseudomonas aeruginosa in 30% of cases). In our report, Ax was found as single bacteria in all patients and bacteremia was present in 60% of the cases, strengthening the evidence of a systemic infection. In our 5-years retrospective database analysis, only 5% of the Ax infections have been diagnosed as VAP and happened mostly in immunosuppressed patients (Fig. 1).

**Conclusion:** Nosocomial pneumonia related to Ax is uncommon and its high prevalence in Covid-19 ARDS patients raise the concern of the immunoparalysis and damages done to the lungs This series of Ax cases doesn’t fit with an outbreak since no other case was diagnosed in non-Covid-19

**Compliance with ethics regulations:** Yes in clinical research

### FC-200 Nosocomial infections associated to COVID19 in critically ill patients: incidence and outcomes

#### Feriel Ben Aba, Hamdi Hemden Doghri, Yosra Ghali, Lilia Debbiche, Youssef Zied Elhechmi, Nabiha Borsali Falfoul

##### Université de Tunis El Manar, Faculté de Médecine de Tunis. Hôpital Habib Thameur. Service des urgences et de réanimation médicale, Tunis, Tunisie

**Correspondence:** Feriel Ben Aba - benaba.feriel@gmail.com

*Annals of Intensive Care* 2021, **11(Suppl 1):**FC-200

**Rationale:** The pandemic of the coronavirus disease has been a real challenge to the intensive care units. It is responsible for a long stay frequently complicated by nosocomial infections. Our objective was to assess the incidence of nosocomial bacterial infections and their impact on COVID 19 critically ill patient’s prognosis.

**Patients and methods/materials and methods:** Descriptive, retrospective and monocentric study including all patients admitted in an intensive care units (ICU) department between 09/07/2020 and 12/31/2020. All data including demographics, clinical, bacteriological, and outcomes were taken from patient’s medical files.

**Results:** During the period of the study, 67 patients infected by the SARS COV 2 were included. Sex ratio was 1.57 and median age was 64. Median SAPS II was 35. 30 patients had developed bacterial nosocomial infections during ICU stay, 19 developed at least two infections, and four developed three infections. Infection occurred after a median of 9 days [5–13] of admission. SOFA score and CPIS at the time of the infection had median value 7 and 4, respectively. There were 33 cases of pneumonia followed by bloodstream infections (*n* = 18) and urinary tract infection (*n* = 2). A microbiological investigation was made in 25 cases. The most frequent bacterium among patients was *Klebsiella pneumoniae* (*n* = 10), followed by *Enterococcus faecium* (*n* = 6), *Acinetobacter Baumanii* (*n* = 4), *Pseudomonas aeruginosa* (*n* = 4) and *Escherichia coli* (*n* = 1). The development of a nosocomial infection extended ICU stay by 10 days. This complication was significantly associated with acute kidney failure occurrence (*p* < 10-3), septic shock (*p* < 10-3), invasive mechanical ventilation (*p* < 10-3) and mortality (*p* < 10-3).

**Conclusion:** Bacterial nosocomial infection is a common complication of ICU admission in patients with COVID-19 and ventilator associated pneumonia remains the most frequent. Our study noticed an association with a higher morbidity and mortality.

**Compliance with ethics regulations:** Yes in clinical research

### FC-201 Diagnosis and risk factors of nosocomial Fungal infections in COVID-19 critically ill patients

#### Yosra Ghali, Hamdi Hemdene Doghri, Feriel Ben Aba, Montassar Bhouri, Imen Zaghdoudi, Ines Sedghiani, Nebiha Borsali-Falfoul

##### Université de Tunis El Manar, Faculté de Médecine de Tunis. Hôpital Habib Thameur. Service des urgences et de réanimation médicale, Tunis, Tunisie

**Correspondence:** Hamdi Hemdene Doghri - doghri.hamdi87@gmail.com

*Annals of Intensive Care* 2021, **11(Suppl 1):**FC-201

**Rationale:** The new coronavirus, SARS COV 2, is causing, in the most severe cases, an acute respiratory distress syndrome which requires prolonged hospitalization in intensive care units. In these patients, the incidence of nosocomial infections and particularly fungal infections deserves to be studied in view of their impact on morbidity and mortality. The aim of our study was to describe the epidemiological, biological and microbiological features of nosocomial fungal infection (FNI) and to search for risk factors to their occurrence.

**Patients and methods/materials and methods:** The study was descriptive, prospective and monocentric, including patients admitted to the medical intensive care unit for an SARS CoV-2 infection during the period from 07/09/2020 to 31/12/2020. Demographic, clinical, microbiological, therapeutic data and outcomes were collected.

**Results:** During the course of the study, we collected 22 patients who developed an NFI during their hospitalization in the intensive care unit. The median age in this group was 69 years [63,73] and the sex ratio was 1.3. The median body mass index was 29 [24,31]. 17 (74%) patients were diabetic and 14 (60%) had hypertension. The median IGSII, APACHE II and SOFA scores at admission were 33 [32–39], 17 [15–20] and 3 [2–4], respectively. The median time to NFI occurrence was 8 days [5,11]. The NFI was diagnosed with a positive mannan antigenemia in 11 cases (with positive anti-mannan serology in 8 cases), a positive colonization index (CI) with a high candida score in 9 cases and positive blood cultures in 2 cases. The CI was achieved in 21 patients and was positive in 20 cases. The most frequently positive site was urinary (30%) and cutaneous (30%). The most frequently isolated candida was *C. albicans* (73.3%), *C. krusei* (23.3%) and C. glabrata (4%). Length of stay had a median of 18 days [14–21]. The occurrence of NFI was significantly related to the presence of diabetes (*p* = 0.007), severe ARDS (*p* < 10^−3^), nosocomial bacterial infection (*p* < 10^−3^), vascular catheterization (*p* < 10^−3^), urinary catheterization (*p* < 10^−3^) and parenteral nutrition (*p* < 10^−3^) also to lower serum albumin level less than 23 (*p* = 0.005). No independent factors predictive of NFI were identified.

**Conclusion:** Fungal infection in patients admitted for Covid-19-related ARDS is frequent and should be investigated early. However, larger prospective studies are needed to confirm this finding.

**Compliance with ethics regulations:** Yes in clinical research.

### FC-202 Impact of secondary infection on COVID-19 patients outcomes

#### Sana Kharrat, Olfa Turki, Saba Makni, Rania Ammar, Malek Hafdhi, Houda Maayoufi, Hedi Chelly, Chokri Ben Hamida, Mabrouk Bahloul, Mounir Bouaziz

##### CHU Habib Bourguiba, Sfax, Tunisie

**Correspondence:** Sana Kharrat - sanakharrat15@hotmail.com

*Annals of Intensive Care* 2021, **11(Suppl 1):**FC-202

**Rationale:** The world is witnessing a global pandemic of coronavirus disease 2019 (COVID-19), which is considered to be related to infection by severe acute respiratory syndrome coronavirus 2 (SARS-CoV-2). Pulmonary diseases of viral origin are often followed by the manifestation of secondary bacterial infections, leading to further clinical complications and negative disease outcomes. The aim of this study was to determine the prevalence of bacterial infections developed after the onset of pulmonary viral infections in patients with COVID-19 and to assess its impact on clinical outcomes.

**Patients and methods/materials and methods:** We conducted a retrospective study including critically ill patients with confirmed SARS-COV2 infection in an intensive care unit between September 2020 and January 2021. Secondary infection was suspected clinical deterioration, chest X-ray and laboratory findings. The identification of the causative organism was performed with endotracheal aspirate (ETA) cultures.

**Results:** A total of 178 patients were admitted during the study period. The mean age was 62 ± 12.8 years, and 132 (74.2%) of patients were male. Among these patients, 84 (47.2%) had hypertension, 76 (42.7%) had diabetes and 17 (9.5%) had chronic lung disease. 40% of patients suffered from obesity. Mean SAPSII score on ICU admission was 34 ± 14.9 and mean SOFA score was 5 ± 2.7. The median rate of inflammatory markers at admission was 80 IQR [41–163] mg/L of C-reactive protein, 0.25 IQR [0.13–0.9] ng/ml of procalcitonin and 12800 IQR [9500–17375] cells/mm3 of leucocytes. Secondary infection was suspected in 61 patients (34.3%). The mean rate of C-reactive protein at infection suspicion was 242.7 ± 162. The most common isolated pathogens were Klebsiella pneumoniae and Acinetobacter baumannii. Secondary infection was significantly associated with higher need of non-invasive mechanical ventilation (48.5% vs. 86.6%; *p* < 10-3; OR = 6.8[3–15.8]), higher need of invasive mechanical ventilation (28.4%vs.76.6%; *p* < 10-3; OR = 9[4.3–18], higher mortality (37.6% vs. 80%; *p* < 10-3;OR = 6.7[3.5–14.1]) and higher length of stay (10.1 ± 6 vs. 6.1 ± 5 days; *p* < 10-3).

**Conclusion:** Secondary bacterial infections are common in covid-19 patients. Our data suggest that these infections are associated with poor prognosis.

**Compliance with ethics regulations:** N/A.

### FC-203 Incidence of EBV, CMV, and HHV-6 reactivations in severe COVID-19

#### Arthur Simonnet^1^, Ilka Engelmann^2^, Anne-Sophie Moreau^2^, Didier Hober^2^, Mercé Jourdain^2^

##### ^1^ch roubaix, Roubaix, France; ^2^CHU Lille, Lille, France

**Correspondence:** Arthur Simonnet - arthursimonnet@hotmail.com

*Annals of Intensive Care* 2021, **11(Suppl 1):**FC-203

**Rationale:** Herpesviruses reactivation is frequent in intensive care unit (ICU) patients, even in critically ill immunocompetent patients [1, 2]. Severity of illness, prolonged invasive mechanical ventilation, ICU length-of-stay, and use of anti-inflammatory drugs are well-know risk factors. Of note, these conditions are commonly encountered in ICU patients with severe acute respiratory syndrome-coronavirus 2 (SARS-CoV-2) infection. Data on severe Coronavirus disease-19 (Covid-19) and concomitant reactivation of herpesviruses are lacking.

**Patients and methods/materials and methods:** Retrospective monocentric study including 34 patients admitted to ICU for confirmed SARS-Cov-2 infection that had systematic testing for EBV, CMV and HHV-6 viremia while in the ICU. Analysis of frequency, timing, duration, co-occurence of viral DNAemia, and association with clinical outcome.

**Results:** DNAemia with EBV, CMV, and HHV-6 was detected in 28/34 (82%), 5/34 (15%), and 7/32 (22%) patients, respectively. EBV reactivation occurred early (median 4 days) after ICU admission and was associated with a longer ICU length-of-stay. CMV reactivation occured more latelely (median 12 days) after ICU admission, and required anti-CMV treatment in 3/5 (60%) patients. HHV-6 viremia was noticeable for a low viral load and a short duration. No association was found between ICU mortality (6/34, 18%) and EBV, CMV and HHV-6 reactivations. This was partly due to the small size of the cohort.

**Conclusion:** Our study shows that EBV, CMV and HHV-6 reactivations are frequent in severe COVID-19. The identification of risk factors and the association with clinical outcome require larger studies. The relationship between herpesviruses reactivation and host immune response deserves further attention.

**Reference**

1. Walton AH, Muenzer JT, Rasche D, Boomer JS, Sato B, Brownstein BH, et al. Reactivation of Multiple Viruses in Patients with Sepsis. PLoS ONE 2014;9:e98819. 10.1371/journal.pone.0098819.
2. MIPrea group, REALISM group, Mallet F, Perret M, Tran T, Meunier B, et al. Early herpes and TTV DNAemia in septic shock patients: a pilot study. Intensive Care Medicine Experimental 2019;7. 10.1186/s40635-019-0256-z.

**Compliance with ethics regulations:** Yes in clinical research.

### FC-204 MR Pro-adrenomedullin as a prognostic factor for severe SARS-CoV2 pneumonia

#### Etienne De Montmollin^1^, Katel Peoc’h^1^, Mehdi Marzouk^1^, Stephane Ruckly^2^, Tiphaine Robert^1^, Paul-Henri Wicky^1^, Juliette Patrier^1^, Pierre Jaquet^1^, Romain Sonneville^1^, Lila Bouadma^1^, Jean-Francois Timsit^1^

##### ^1^APHP- hopital Bichat, Paris, France; ^2^ICURESEARCH, Romans, France

**Correspondence:** Jean-Francois Timsit - jean-francois.timsit@aphp.fr

*Annals of Intensive Care* 2021, **11(Suppl 1):**FC-204

**Rationale:** Mid-regional proadrenomedullin (MR-proADM) is a peptide, an adipokine, generated by multiple tissues to stabilize the microcirculation and protect against endothelial permeability, both of which are widely acknowledged to play a significant role in the pathophysiological host response to sepsis. An elevated concentration of MR-proADM has been associated with both short-term mortality and complications in patients with sepsis and community-acquired pneumonia. Endothelial dysfunction plays an essential role in the pathophysiology of severe SARS-CoV-2 infection; therefore, we wonder if MR-proADM measured serially could be an informative prognostic biomarker in this disease.

**Patients and methods/materials and methods:** Consecutive biobanked samples of patients from the Outcomerea database admitted for severe SARS-CoV-2 infections in one ICU were tested. Day 1, day 3, and day 7 MR proADM were measured using an immunological assay with the TRACE technology (time-resolved amplified cryptase emission; B·R·A·H·M·S MR-proADM KRYPTOR assay), blindly from the clinical outcomes. Patients were followed until day 60 without follow-up losses.

**Results:** Of the 63 included patients (Table) (duration of ICU stay median 9 days [IQR 5;16]), 22 died at day 60 (34.9%). Bloodstream infections (BSIs) occurred in 18 (28.6%) cases after 5.5 days in median. Pulmonary coinfections or superinfections occurred in 29 (46%) cases after 2 days in the median. MR Pro-ADM at days 1,3, and 7 were significant predictors of BSI, pulmonary superinfections and mortality (Fig. 1). Other traditional biological outcome predictors are in the table. Only day 1 IL-6 and PCT concentrations were associated with prognosis. AUC of the ROC curve (0.801, *p* < 0.001) of Day 1 Pro ADM compared favorably with the AUC ROC of the day1 PCT (0.736, *p* < 0.001). Serial ProADM measurement and evaluation of proADM value did not add significant prognostic information. A day1 ProADM of 1 or higher is associated with a poor prognosis, *p* = 0.0047; logrank test

**Conclusion:** On the basis of this monocenter prospective study, MR proADM appears to be a promising predictor of coinfections and outcome in severe sars-Cov2 infections.

**Compliance with ethics regulations:** Yes in clinical research.
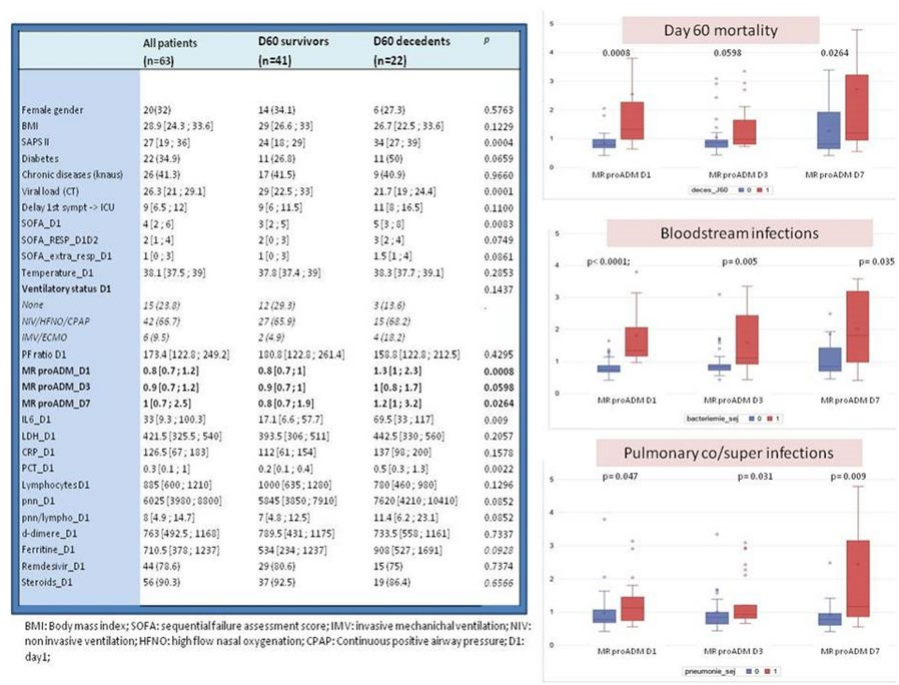


Patients and serial MR pro-adrenomedullin

### FC-205 Immature granulocytes: a robust biomarker of ventilator-associated bacterial co-infections in severe COVID-19 patients

#### Thomas Daix, Robin Jeannet, Ana Catalina Hernandez Padilla, Anne-Laure Fedou, Marine Goudelin, Philippe Vignon, Bruno François

##### CHU Dupuytren, Limoges, France

**Correspondence:** Thomas Daix - thomas.daix@chu-limoges.fr

*Annals of Intensive Care* 2021, **11(Suppl 1):**FC-205

**Rationale:** Intensive care unit (ICU) patients under mechanical ventilation for acute respiratory distress syndrome (ARDS) related to SARS-CoV-2 frequently develop ventilator associated pneumonia (VAP). Nevertheless, VAP confirmation is challenging in case of severe COVID-19 pneumonia, those two entities sharing almost the same diagnostic criteria (1). Specific biomarkers of bacterial insult could help clinicians in the diagnosis of VAP. Interestingly, bacterial insult leads to emergency granulopoiesis and then immature granulocytes (IG) regression in blood. We thus investigated if the frequencies of IG in severe COVID-19 patients could be associated with VAP.

**Patients and methods/materials and methods:** ICU patients mechanically ventilated for ARDS related to SARS-CoV-2 were eligible. The following populations were analyzed at ICU admission, on day 7 (± 2) and 15 (± 2): CD16low IG, monocyte HLA-DR expression (mHLA-DR), CD4+ and CD8+ T Lymphocytes. An independent committee blindly adjudicated VAP based on radiological findings, microbiological documentation and clinical symptoms.

**Results:** Seventeen consecutive patients were included (72 [65–76] y.o; SAPS II: 29 [25–35]; mortality rate: 35%). Ten had VAP (median diagnosis date of VAP: 6.5 [4.3–7.8] days). Severity and comorbidities were similar between patients with or without VAP. At ICU admission, mean IG percentage and absolute number were, respectively, 3.22 ± 3.78% and 0.4 ± 0.75 G/L regardless the occurrence of VAP during ICU stay. However, on day 7, patients with confirmed VAP had a major peak of IG compared to patients without VAP, both in percentage and absolute numbers (55.6 ± 26.6% vs 9.0 ± 5.9%, *p* = 0.0001 and 6.9 ± 4.72 G/L vs 0.95 ± 0.75 G/L, *p* = 0.0002; Fig. 1). On day 15, IG level had decreased and were close to those observed at ICU admission (8.7 ± 5.58% and 1.75 ± 1.13 G/L). Usual markers of immunosuppression such as mHLA-DR expression, CD4+ and CD8+ T cell lymphopenia were not significantly different between groups at any time during follow-up. A threshold of IG percentage at 18% or absolute number at 2.8 G/L showed an excellent performance with 100% specificity and sensitivity for the diagnosis of associated bacterial infection in COVID-19 patients mechanically ventilated for SARS-CoV-2-related ARDS.

**Conclusion:** Increased percentage of IG in peripheral blood could be used as a robust biomarker of VAP in ventilated COVID-19 patients. Diagnostic performance of IG for identifying VAP in COVID-19 patients is promising and should be confirmed in larger prospective cohorts.

**References**

1. François et al. Crit care 2020.
2. Manz et al. Nat Rev Immunol 2014.

**Compliance with ethics regulations:** Yes in clinical research.
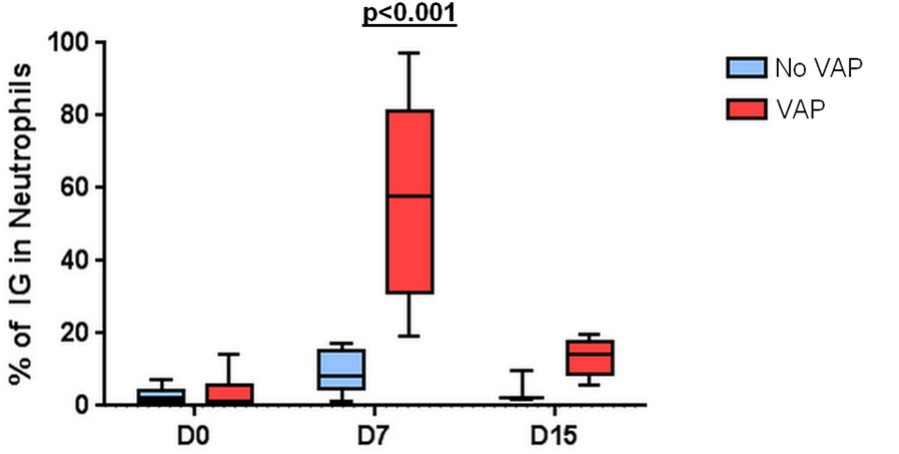


Fig. 1 Percentage of Immature Granulocytes in peripheral blood at D0, D7(± 2) and D15(± 2) in COVID-19 patients without VAP (blue) and patients with VAP (red)

### FC-206 Effects of glycemic characteristics on clinical outcomes of patients with severe COVID-19

#### Imen Talik, Najla Ben Slimene, Boudour Ben Dhia, Malak Kharrat, Moez Kaddour, Takoua Merhabene

##### hôpital régional Zaghouan, Zaghouan, Tunisie

**Correspondence:** Imen Talik - talik.imen2013@gmail.com

*Annals of Intensive Care* 2021, **11(Suppl 1):**FC-206

**Rationale:** Diabetes has emerged as an important risk factor for severe illness and death from COVID-19. There is a paucity of information on glycemic control among hospitalized COVID-19 patients with diabetes and acute hyperglycemia. The aim of our study was to identify prevalence of hyperglycemia in COVID-19 critically ill patients with and without prior diabetes and evaluate its association with COVID-19 course.

**Patients and methods/materials and methods:** This observational cohort study included all consecutive COVID19 patients admitted to the ICU of a tertiary hospital, from March 23rd 2020 to Januray 31st 2021. Patients were divided into three groups: GI known diabetic patients; G II hyperglycemia without diabetes and GIII reference group. Diabetes was defined as HbA1C ≥ 6.5%. Uncontrolled hyperglycemia was defined as at least one blood glucose value ≥ 9.9 mmol/L. Outcomes such as mortality, need of invasive mechanical ventilation (IMV), and length of ICU stay (LOS) were measured.

**Results:** 121 patients were enrolled, median age was 64 years (IQR, 56–70), sex ratio = 1.5, Medians of SAPS II and APACHE II scores were, respectively, 26 ± 8 and 8 ± 3. Hypertension (48%) and diabetes (35%) were the most frequent underlying diseases. Fifty patients (41%) required invasive mechanical ventilation and all patients have received corticosteroids at admission. Hyperglycemia was observed in 103 (85%) patients with a mean of 11 ± 1 mmol/l. Among them 61 cases was noted at admission. Considering glycemic values, patients’ repartition was 40.5% in G I; 44.5% in G II, and 15% in G III. Median glycemic values were, respectively, GI:13.7 mmol/l (IQR, 8.8–19); GII:11 mmol/l (IQR, 7.5–12);GIII: 7.2 mmol/l (IQR, 6.2–8.8).Global ICU mortality and need of IMV were, respectively, and significantly higher in patients assigned to GI and GII regrouped together compared to the reference group (49% vs 11%, *p* = 0.002), (46% vs 11%, *p* = 0.005), These results were also confirmed in separated groups (Table 1). Table 1. Outcomes of patients with diabetes/hyperglycemia compared with reference group

**Conclusion:** Hyperglycaemia is common in COVID-19 critically ill patients. These COVID-19 patients with diabetes and/or uncontrolled hyperglycemia had a longer LOS and markedly higher mortality. We recommend health systems which ensure that inpatient hyperglycemia is safely and effectively treated.

**Compliance with ethics regulations:** Yes in clinical research.
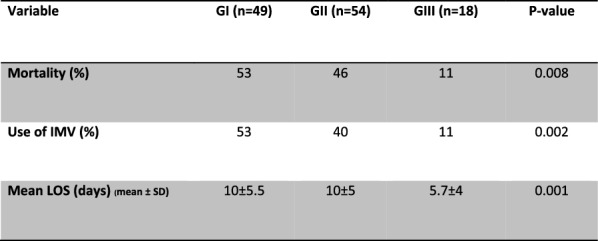


Table 1 Outcomes of patients divided into three groups.

Abbreviations: GI, known diabetic patients; GII, hyperglycemia without diabetes; GIII, reference group; IMV, invasive mechanical ventilation; LOS, length of stay; SD, standard deviation

### FC-207 Hyperglycemia in COVID-19-infected patients: incidence and impact on outcome

#### Rezk Ghorbel, Sana Kharrat, Olfa Turki, Kamilia Chtara, Najeh Baccouche, Saba Makni, Dorsaf Dlensi, Chokri Ben Hamida, Mabrouk Bahloul, Mounir Bouaziz

##### CHU Habib Bourguiba, Sfax, Tunisie

**Correspondence:** Sana Kharrat - sanakharrat15@hotmail.com

*Annals of Intensive Care* 2021, **11(Suppl 1):**FC-207

**Rationale:** Since December 2019, Coronavirus disease 2019 (COVID19) outbreak occurred and has rapidly spread worldwide. Previous studies showed hyperglycemia was a risk factor for high morbidity and mortality in those who contracted severe acute respiratory syndrome (SARS). Our objective was to identify prevalence of hyperglycemia in COVID-19 patients and its impact on mortality.

**Patients and methods/materials and methods:** We conducted a retrospective study including critically ill patients with confirmed SARS-CoV-2 infection in an intensive care unit between September 2020 and January 2021. Outcomes were the rate of infections, length of stay and in-hospital mortality.

**Results:** A total of 155 patients were admitted during the study period. The mean age was 62.17 ± 12.8 years, and 118 (76.1%) of patients were male. Among these patients, 73(47.1%) had hypertension, 68 (44%) had diabetes and 14 (9.7%) had chronic lung disease. 42% of patients suffered from obesity. Mean SAPSII score on ICU admission was 34 ± 15.6 and mean SOFA score was 5 ± 2.9. The mean rate of serum glucose on admission was 12.7 ± 6.3 mmol/l. Hyperglycemia occurred in 61 (39.4%) patients; 18 of these hyperglycemic patients (29.5%) had no prior history of diabetes. The mean PH and the mean HCO3- at admission were significantly lower in patients with hyperglycemia (7.36 vs. 7.41; *p* = 0.02 and 21.8 vs. 23.6; *p* = 0.04, respectively) The rate of bacterial infections and the length of stay were not significantly different between patients developing hyperglycemia or not. Mortality was significantly higher in patients developing hyperglycemia during hospitalization (62.2% versus 42.3% *p* = 0.01; OR = 2.2[1.1–4.3]).

**Conclusion:** Our data suggest that hyperglycemia could worsen the prognosis of patients and exposes them to higher mortality. For this reason, improving glucose control is a critical measure to improve outcomes.

**Compliance with ethics regulations:** N/A.

### FC-208 Serum cholinesterase activity in COVID-19 infected patients: a predictor of severity

#### Sana Kharrat, Najeh Baccouch, Salma Jerbi, Houda Maayoufi, Olfa Turki, Amal Triki, Dorsaf Dlensi, Hedi Chelly, Mabrouk Bahloul, Mounir Bouaziz

##### CHU Habib Bourguiba, Sfax, Tunisie

**Correspondence:** Sana Kharrat - sanakharrat15@hotmail.com

*Annals of Intensive Care* 2021, **11(Suppl 1):**FC-208

**Rationale:** Coronavirus Disease 2019 (COVID-19) attracted worldwide attention as an international public health emergency causing millions of deaths. Nevertheless, the disease course and predictors of mortality in critically ill patients are still poorly understood. We aimed to investigate whether serum cholinesterase (SChE) activity can be helpful for severity stratification of patients. This risk stratification may subsequently guide optimized management of these patients.

**Patients and methods/materials and methods:** We conducted a retrospective study including critically ill patients with confirmed SARS-COV2 infection in an intensive care during 4 months. Outcomes were: requirement of ventilator support, length of stay and in-hospital mortality.

**Results:** A total of 178 patients were admitted during the study period. The mean age was 62 ± 12.8 years, and 132 (74.2%) of patients were male. Among these patients, 84(47.2%) had hypertension, 76 (42.7%) had diabetes and 17 (9.5%) had chronic lung disease. 40% of patients suffered from obesity. Mean SAPSII score on ICU admission was 34 ± 14.9 and mean SOFA score was 5 ± 2.7. The median rate of inflammatory markers at admission was 80 IQR [41–163] mg/L of C-reactive protein, 0.25 IQR [0.13–0.9] ng/ml of procalcitonin and 12800 IQR [9500–17375] cells/mm3 of leucocytes. The mean serum cholinesterase activity rate was 5182 ± 1946 UI/l (ranging from 1403 to 11192 UI/l). The SChE activity rate was significantly higher in survivors (5977 ± 1992 versus 4505 ± 1636; *p* < 10-3). In our study, we found that a rate of SChE activity lower than 5000 was associated with a higher length of stay (10.1 ± 6.5 versus 6.5 ± 4 days; *p* < 10-3), greater need of ventilatory assistance (69.1% versus 24%; *p* < 10-3. OR = 7.1[3.3–15.8]) and higher mortality (75.4% versus 32.8%; *p* < 10-3;OR = 6.25[2.9–13.2]).

**Conclusion:** These findings suggest that the SChE scoring, could be useful to identify high-risk patients at ICU admission, activity, used in combination with the laboratory tests, clinical examination, and the disease severity.

**Compliance with ethics regulations:** N/A.

### FC-209 Biomarkers in critically ill COVID-19 patients

#### Feriel Ben Aba, Hamdi Hemdene Doghri, Montassar Bhouri, Amal Jebali, Ines Sedghiani, Imen Zaghdoudi, Nebiha Borsali-Falfoul

##### Université de Tunis El Manar, Faculté de Médecine de Tunis. Hôpital Habib Thameur, Service des urgences et de réanimation médicale, Tunis, Tunisie

**Correspondence:** Hamdi Hemdene Doghri - doghri.hamdi87@gmail.com

*Annals of Intensive Care* 2021, **11(Suppl 1):**FC-209

**Rationale:** Biology has a great contribution in the COVID-19 pandemic. Numerous biomarkers contribute to the COVID-19 diagnosis and are essential for the prediction of the severe forms and their complications in critically ill patients. Several reports have been interested in biomarkers associated to COVID 19 and their roles in the risk stratification and assessment of severity of SARS COV 2 infection. Our study objective was to describe biological markers in critically ill COVID-19 patients and to identify parameters associated to morbidity and mortality.

**Patients and methods/materials and methods:** Monocentric observational descriptive and prospective study including patients admitted in an ICU department between 09/07/2020 and 12/31/2020. Patients’ data (demographics, clinical, biological parameters and outcome) were taken from patient’s medical files. Evaluation criteria were severe adult respiratory distress syndrome (ARDS), non-invasive ventilation (NIV) failure and mortality.

**Results:** 67 patients infected by the SARS COV 2 were admitted in ICU department. Sex ratio was 1.57 and median age was 64. Several biological exams were conducted at admission and during hospitalisation. Complete blood count showed a leucocytes median value of 9.5 * 103/L [7500–14990] and a lymphocytes median value of 0.88 * 103/L [630–1250] respectively. The median value of lactate dehydrogenase (LDH) was 518 IU/L [375–693]. Inflammatory markers as C-reactive protein and fibrinogen had median value at admission, respectively, of 153 mg/l [84–240] and 4.87 g/l [3.64–6.03] and at maximal range, respectively, of 237 mg/l [129–302] and 5.68 g/l [4.24–7.12]. The median value of D-dimer at admission was 1202 µg/l [659–2723] and at most 2404 [1078–6626] µg/l. Troponins were positive at admission in 22 cases. A multivariate analysis had identified the CRP cutoff value above 150 mg/l as an independent risk factor associated to a severe form of ARDS(OR: 7.42; 95% CI: 1.29–42.58; *p* = 0.025). It was also associated to a NIV failure (OR: 4.85; 95% CI: 1.37–17.11; *p* = 0.014) and to a higher mortality (OR: 5.93; 95% CI: 1.37–17.11; *p* = 0.014). D-dimer rate above 1800 µg/l was an independent risk factor of a severe form of ARDS (OR: 5.33; 95% CI: 1.2–23.72; *p* = 0.028).Fibrinogen rate above 6.5 g/l was also an independent risk factor of an NIV failure (OR: 5.93; 95% CI: 1.59–22.07; *p* = 0.008) and of a higher mortality (OR: 4.85; 95% CI: 1.37–17.11; *p* = 0.014).

**Conclusion:** Our study reported two coagulation parameters and one inflammatory parameter as main factors associated to a critical clinical presentation. CRP and D-dimer were associated to a severe form of ARDS. CRP and Fibrinogen were associated to an NIV failure and mortality.

**Compliance with ethics regulations:** Yes in clinical research.

### FC-210 Prognostic value of inflammatory markers in patients with Coronavirus 2019

#### Sana Kharrat, Rania Ammar, Malek Hafdhi, Farah Zouari, Kamilia Chtara, Amal Triki, Hedi Chelly, Chokri Ben Hamida, Mabrouk Bahloul, Mounir Bouaziz

##### CHU Habib Bourguiba, Sfax, Tunisie

**Correspondence:** Sana Kharrat - sanakharrat15@hotmail.com

*Annals of Intensive Care* 2021, **11(Suppl 1):**FC-210

**Rationale:** The outbreak of coronavirus disease 2019 (COVID-19) is an emerging global health threat. Nevertheless, predictors of clinical severity and complications in critically ill patients are still poorly described. Recent studies have reported that inflammatory marker levels are elevated in patients with COVID-19 and may correlate with severity of disease and disease progression. We aimed to investigate the association between several biomarkers, including serum C-reactive protein (CRP), procalcitonin (PCT), and serum ferritin, and COVID-19 severity.

**Patients and methods/materials and methods:** We conducted a retrospective study including critically ill patients with confirmed SARS-COV2 infection in an intensive care unit during 4 months. Outcomes were defined as the need of ventilator support, length of stay and in-hospital mortality.

**Results:** A total of 178 patients were admitted during the study period. The mean age was 62 ± 12.8 years, and 132 (74.2%) of patients were male. Among these patients, 84(47.2%) had hypertension, 76 (42.7%) had diabetes and 17 (9.5%) had chronic lung disease. 40% of patients suffered from obesity. Mean SAPSII score on ICU admission was 34 ± 14.9 and mean SOFA score was 5 ± 2.7. The median rate of inflammatory markers at admission was 80 IQR [41–163] mg/L of C-reactive protein, 0.25 IQR [0.13–0.9] ng/ml of procalcitonin (PCT), 12800 IQR [9500–17375] cells/mm^3^ of leucocytes and 864 IQR [426–1380]μg/l of ferritin. In our study we proved that a rate of PCT higher than 0.3 µg/L was significantly associated with mortality (74.7% versus 32.8%; *p* < 10-3; OR = 6 [2.9–12.3] and a higher use of invasive ventilatory assistance (69% versus 20.6%; *p* < 10-3: OR = 8.5 [4–18.3]). We also found that a rate of CRP higher than 120 mg/l was significantly associated with mortality (67% versus 35.7%; *p* < 10-3; OR = 3.7 [1.9–7]) and a higher use of invasive ventilatory assistance (58.8% versus 30.4%; *p* < 10-3: OR = 3.2 [1.6–6.35]).

**Conclusion:** Inflammatory markers including C-reactive protein (CRP) and procalcitonin (PCT) have been identified as predictors of clinical severity and complications to provide reference for clinical treatment. More sensitive indicators able to reflect lung lesion changes and disease severity had to be explored.

**Compliance with ethics regulations:** N/A.

### FC-211 Intranasal ketamine for moderate-to-severe pain in the emergency department

#### Manel Kallel, Khadija Zaouche, Wejden Essbil, Ramla Baccouche, Hamida Maghraoui

##### RABTA HOSPITAL, Tunis, Tunisie

**Correspondence:** Manel Kallel - DR.MANEL.KALLEL@GMAIL.COM

*Annals of Intensive Care* 2021, **11(Suppl 1):**FC-211

**Rationale:** Known for its low dose analgesic properties, ketamine is molecule of choice in procedural sedation in emergency rooms. Several routes are being tested. The objective of our study was to assess the analgesic property of intranasal ketamine in moderate-to-severe pain in the emergency department

**Patients and methods/materials and methods:** It was a prospective study carried out in the emergency department during 1 year. We included patients over 18 years of age with moderate-to-severe acute pain with a visual analogue scale (VAS ≥ 5). We did not include patients with any of the following criteria: History of allergy or intolerance to ketamine, Structural or functional nasal occlusion. Ketamine was used intranasally at a dose of 1 mg/kg. Primary endpoint was obtaining a VAS ≤ 3 mm at 30 min from the administration of ketamine.

**Results:** We included 60 patients with a sex ratio of 1.14. The median age was 46 years old with extremes of 19 and 72. The fifth minute, One patient reported the complete disappearance of the pain. Seven patients (11.7%) had mild pain with a median VAS 2(3–1) [0–3].22 patients (36.7%) described moderate pain with a median VAS 5(5–4) [4–5], an increase of 28.3%, 31 patients (51.6%) described severe pain. The median VAS was 8(6.5–9) [6–10], a decrease of 40%. At 30 min, 40 patients (66.7%) had mild pain with a median VAS 2(3–0) [0–3], 14 patients (23.3%) described moderate pain with a median VAS 5 (5–4) [4–5] and 6 patients (10%) had severe pain with a median VAS of 7.5(8–7) [6–8], a decrease of 81.6%. Vertigo is the most common side effect. It accounts for 78.6% of all side effects. Twenty patients had more than one adverse event. No patients experienced dyspnea, sedation or hallucination.

**Conclusion:** Intranasal ketamine appears to be effective and safe in the management of acute moderate-to-severe pain in emergency department.

**Compliance with ethics regulations:** Yes in clinical research.

### FC-212 Low-dose intravenous ketamine as a single agent for acute pain management in the emergency

#### Manel Kallel, Khadija Zaouche, Mohamed Kilani, Ramla Baccouche, Hamida Maghraoui

##### RABTA HOSPITAL, Tunis, Tunisie

**Correspondence:** Manel Kallel - DR.MANEL.KALLEL@GMAIL.COM

*Annals of Intensive Care* 2021, **11(Suppl 1):**FC-212

**Rationale:** Ketamine used in sub-dissociative doses has emerged as an alternative to traditional opioids for pain control in emergency department. The aim of our study was to evaluate analgesic efficacy and safety of intravenous (iv) ketamine in emergency department (ED).

**Patients and methods/materials and methods:** This was a randomized, double-blind, 10-month prospective trial. Patients with moderate-to-severe acute pain, defined as a score on a numerical rating scale (NRS) of 6 or greater were randomized to receive either ketamine 30 mg iv or morphine 10 mg iv. Primary endpoint was pain reduction at 10 min. Informed consent was obtained.

**Results:** We included 120 patients; 54 received ketamine and 66 morphine. Mean age was 44 ± 15 years in morphine group and 43 ± 14 years in ketamine group *p* = 0.7. Thirty-two patients (26.6%) visited the ED for a trauma. Thirty-six percent presented chest pain, 21% limb pain and 18% abdominal pain. Overall NRS was at 8.7 ± 1.4 with no difference between groups. At 10 min, the same number of patients in both groups achieved an NRS lower than 4 (*p* = 0.09). However, the mean NRS was significantly lower in the ketamine group (*p* = 0.008). More adverse effects were noted in the ketamine group (*p* < 0.0001). No significant changes in vital signs were observed and no serious adverse events occurred. Nevertheless, patients in the ketamine group reported an increase in minor adverse events 15 min after drug administration.

**Conclusion:** Intravenous ketamine offers an analgesic efficacy comparable to that of intravenous morphine for short-term management of acute pain in the ED.

**Compliance with ethics regulations:** Yes in clinical research.

### FC-213 A simplified EEG montage and its interpretation for evaluation of patients with persistent unresponsiveness in the ICU

#### Sonia Abid, Grégory Papin^2^, Geoffroy Vellieux^2^, Etienne De Montmollin^2^, Paul-Henri Wicky^2^, Juliette Patrier^2^, Pierre Jaquet^2^, Lila Bouadma^2^, Anny Rouvel Tallec^2^, Jean-François Timsit^2^, Romain Sonneville^2^

##### ^1^Hôpital Saint Louis, Paris, France; ^2^Hôpital Bichât Claude Bernard, Paris, France

**Correspondence:** Sonia Abid - s.sonia.abid@gmail.com

*Annals of Intensive Care* 2021, **11(Suppl 1):**FC-213

**Rationale:** Performing EEG is recommended to assess brain function and outcome of comatose patients. Background rhythm and associated abnormalities (discontinuous, unreactive patterns) are important parameters to identify for prognostication. The objective of the study was to assess feasibility and interpretation of a simplified EEG montage performed by intensivists for prognostication.

**Patients and methods/materials and methods:** All consecutive patients admitted in the ICU from January to November 2019, for whom an EEG was requested for persistent unresponsiveness, were eligible. For each patient, a simplified (10 monopods) EEG montage was performed by one intensivist at bedside. The interpretation of EEG was performed by one intensivist and one neurophysiologist, on 3 specific patterns: (1) predominant background EEG frequency (Hz); (2) presence of a continuous or discontinuous background, including burst suppression; (3) background reactivity. Data are presented as median (interquartile range) or numbers (percentage). Agreement between the neurophysiologist and the intensivist was made using Cohen’s kappa coefficient or Pearson’s correlation, as appropriate. Statistical analyses were performed with SAS^®^ Software. The study was approved by the ethics committee of the Société de Réanimation de Langue Française (CE SRLF 19–24).

**Results:** Main EEG indications performed in 36 unresponsive patients (age 53 [40–68] years, male gender 72% (*n* = 26), SAPS II score 60 [39–74]) were: delayed awakening (*n* = 8, 22%)—seizure detection (*n* = 7, 19%)—anoxic encephalopathy (*n* = 15, 42%)—infectious encephalitis (*n* = 1, 3%)—status epilepticus (*n* = 5, 14%). EEGs were performed within 16 [8–34] h after indication; procedure duration in ICU room was 47 [43–53] min, including 21 min for monopods installation. Background EEG frequency ranged from 1 [1–2] to 5 [4–6] Hz, with predominant theta (39%) or delta (47%) activity. Continuous EEG background was observed in 28 (78%) cases, and preserved EEG reactivity to both nociceptive and auditory stimuli was observed in 4 (11%) cases. Agreements between the intensivist and the neurophysiologist are presented in the Table. Concerning background frequency and continuity, the correlation was satisfactory, whereas there were discrepancies for background reactivity analysis (Cohen’s kappa 0.37 and 0.44, respectively). Compared to the neurophysiologist, the intensivist diagnosed preserved reactivity more frequently, which could be influenced by clinical reactivity at bedside during stimulation.

**Conclusion:** A simplified EEG montage for prognostication of comatose patients in the ICU appeared technically feasible. Compared to physiologists’ interpretation, identification of background frequency and continuity by intensivist was satisfactory, whereas discrepancies were observed for background reactivity. These preliminary findings deserve validation in an independent cohort.

**Compliance with ethics regulations:** Yes in clinical research.
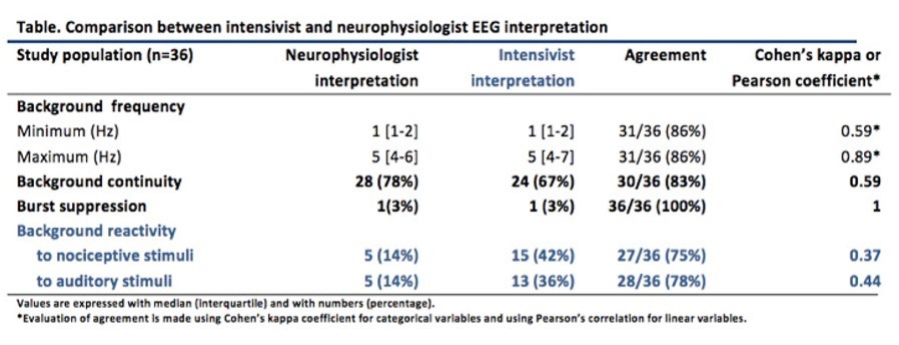


Table Comparison between intensivist and neurophysiologist EEG interpretation

### FC-214 Guillain–Barré syndrome: demographics, clinical features, and outcome in ICU: a retrospective study of 10 years

#### Rezk Ghorbel, Olfa Turki, Hanen Abid, Saba Makni, Kamilia Chtara, Hedi Chelly, Mabrouk Bahloul, Mounir Bouaziz

##### centre Hospitalo-universitaire Habib Bourguiba sfax, Sfax, Tunisie

**Correspondence:** Rezk Ghorbel - rezkghorbel3@gmail.com

*Annals of Intensive Care* 2021, **11(Suppl 1):**FC-214

**Rationale:** Guillain–Barré syndrome (GBS) causes acute neuromuscular weakness. Severe cases are life-threatening, requiring ICU hospitalization. Clinico-epidemiological features and prognosis of GBS in ICU have rarely been reviewed. The mainstays of treatment are intravenous immunoglobulin (IVIg) or therapeutic plasma exchange (TPE), with supportive care. Objective: We aimed to investigate the clinico-epidemiological features, treatments, clinical course and outcomes of GBS patients in our intensive care unit.

**Patients and methods/materials and methods:** A retrospective observational study was performed over 10 years (2010–2020). All GBS presentations admitted to ICU were included. Clinico-epidemiological features, treatments, clinical course and outcomes were assessed.

**Results:** We reviewed 43 GBS patients (58% male) with a mean age at 41 ± 15 years. Previous infection was identified in 72% of cases; and it was mainly secondary to flu-like syndromes (44%). The mean delay between symptoms onset and ICU hospitalization was 11 days. The major cause of hospitalization was acute respiratory failure (44%). The mean IGSII was at 16 and mean SOFA was at 2. Acute motor axonal neuropathy was the most common feature (42%) and acute inflammatory demyelinating polyradiculoneuropathy was found at electroneuromyography in 19% of cases. cerebrospinal fluid albumino-cytologic dissociation was present in 56%. Thirty percent received IVIg and 54% received TPE. Twenty-two patients (53%) needed mechanical ventilation with a mean delay of 12 days and a mean duration of 14 days. The major complication during ICU stay was pulmonary infection (42%), and pulmonary embolism was detected in 14% of cases. The mortality rate was at 21% in our population. The risk factors were higher SOFA score, infection and pulmonary embolism.

**Conclusion:** Respiratory failure in GBS merits more attention from caregivers. Understanding the risk factors of severe GBS requiring ICU admission may provide better treatment strategies and improve the outcome.

**Compliance with ethics regulations:** Yes in clinical research.

### FC-215 Risk management in a multi-organ donation unit: failure mode and effects analysis

#### Imen Kamoun

##### Hôpital Simone Veil, Eaubonne, France

**Correspondence:** Imen Kamoun - imen.kamouna@gmail.com

*Annals of Intensive Care* 2021, **11(Suppl 1):**FC-215

**Rationale:** Organ transplantation is the only alternative for many patients with terminal organ failures. It has become essential for developed and mature health care systems. While the demand for organ transplants is increasing, the rate of transplants is still low. Our multi-organ donation coordination focused on the quality and security of the “organ donation process” in potential brain-dead organ donors.

**Patients and methods/materials and methods:** We analyzed the organ donation process. We reviewed its steps and subsystems with the backward mapping. We identified potential failure modes of the procedure and their causes and effects and we recorded them in a specific Failure mode and effects analysis (FMEA) worksheet.

**Results:** The specific failure mode and effects analysis (FMEA) worksheet with the backward mapping of the “organ donation management” process identified 7 process steps, 30 sub-processes and 80 failure modes. By weighting the items at the management level (Fig. 1), we identified 3 risks in red (not acceptable) and 18 risks in orange (acceptable to assess). For each of these failures, we have proposed actions for improvement. The interveners are essentially the organ donation coordination, the resuscitation department and the quality and risk assessment department.

**Discussion:** The backward analysis of the risks allowed us: to analyze the current organization and operational modes; to identify risky situations in organ donation process; to propose actions to improve and secure these practices

**Conclusion:** As part of the hospital certification on the one hand and in response to the biomedicine agency’s audit report, we have been working on the organ donation process and risk management and have put in place corrective actions. A periodic assessment is necessary to update the criticalities of the risks and update the action plan.

**References**

1. ANSM, Bonnes pratiques relatives au prélèvement de tissus et de cellules du corps humain: système de management de la qualité; Approche par la gestion des risques. Février 2020.
2. HAS, Outils de visite à destination des experts visiteurs: Guide thématique don d’organes et de tissus à visée thérapeutique. Août 2017.

**Compliance with ethics regulations:** Yes in clinical research.
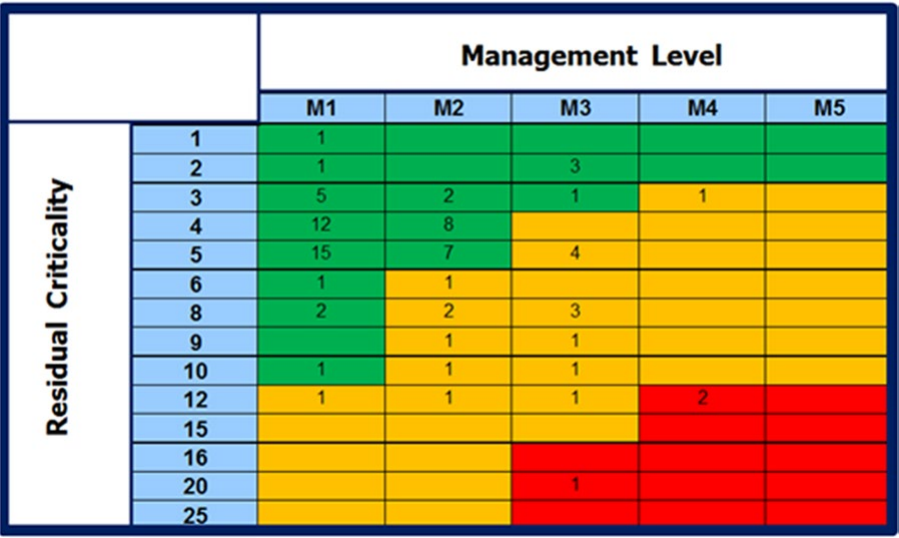


Fig. 1 Risk matrix

### FC-216 Absence of fear of dying predicts new organ failure

#### Aurélien Mazeraud

##### GHU paris Psychiatrie et Neurosciences, Paris, France

**Correspondence:** Aurélien Mazeraud - aurelien.mazeraud@gmail.com

*Annals of Intensive Care* 2021, **11(Suppl 1):**FC-216

**Rationale:** Intensity of anxiety at admission in intensive care unit (ICU) is associated with subsequent deterioration. The primary aim of this study was to assess the additive value of stressful fears and feelings to predict new organ failure within the first 7 days after ICU admission, after adjustment on critical illness severity and anxiety defined as a State–Trait Anxiety Inventory (STAI) ≥ 40.

**Patients and methods/materials and methods:** We conducted a prospective three-center cohort study of non-comatose patients without delirium or invasive mechanical ventilation. A twelve-item questionnaire was developed to assess stressful fears and feelings. Severity of illness was assessed using SAPS-II and SOFA scores. Intensity of chronic and acute anxiety was assessed with the ‘Trait’ and ‘State’ forms of the STAI. Patients were followed up for 7 days.

**Results:** From April 2014 to December 2017, 373 patients (median age 63 years [49–74]; 159 [41%] women; SAPS-II 28 [19–37]) were included. Feeling of vulnerability and fear of dying was reported in 209 (54%) and 178 (46%) patients, respectively. STAI ≥ 40 was observed in 192 (52%) patients. Ninety-four (25%) patients developed a new organ failure. Feeling of vulnerability (OR = 1.96, 95%CI: 1.12–3.43], *p* = 0.01) and absence of fear of dying (OR = 2.38, 95%CI: 1.37–4.17], *p* = 0.002) were associated with occurrence of a new organ failure after adjustment on STAI ≥ 40, SAPS-II and SOFA.

**Conclusion:** Absence of fear of dying is associated with occurrence of new organ failure within the seven post-ICU admission days. We hypothesize that fear of dying might be protective for subsequent deterioration by mobilizing patient’s homeostatic resources. Trial registration: ClinicalTrials.gov, NCT02355626

**Compliance with ethics regulations:** Yes in clinical research.
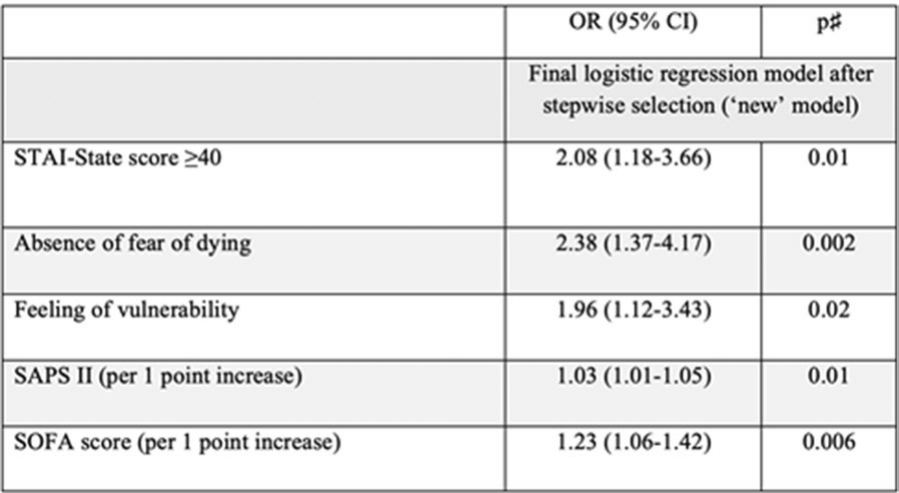


Multivariate analysis of factors associated with occurrence of new organ failure

### FC-217 Severe neurological complications in adult patients with hematological malignancies

#### Thimothée Le Jeannic, Thierry Berghmans, Lorenzo Ferlini, Lieveke Ameye, Marianne Paesmans, Jean-Paul Sculier, Anne-Pascale Meert

##### Institut Jules Bordet, 1000, Belgique

**Correspondence:** Anne-Pascale Meert - ap.meert@bordet.be

*Annals of Intensive Care* 2021, **11(Suppl 1):**FC-217

**Rationale:** Adult patients with hematological malignancies are at risk of neurological complications. The primary objective of this study was to characterize neurological complications leading to ICU admission in this population.

**Patients and methods/materials and methods:** Single-center retrospective study including 57 patients (66 admissions) admitted to the ICU between January the 1st 2009 and December 31st 2018 for a neurological complication.

**Results:** The population was mostly composed of patients with non-Hodgkin lymphomas (35%) and acute myeloblastic leukemia (33%), 35% had stem cell allografts. The causes of neurological deterioration were: 45% cerebrovascular complications (mainly stroke and subdural hematoma), 36% encephalopathies (primarily toxic), 11% infections and 9% tumoral invasion. ICU mortality was 17%. The median SOFA score upon ICU admission was 4.7 and SAPS II 37. Twelve percent of admissions required mechanical ventilation, 10% vasopressors, and 4% extrarenal epuration. Hospital mortality was 35%, survival of the 39 patients alive after hospitalization was 74% at 6 months and 62% at 1 year. In multivariate analysis, the factors associated with hospital mortality were age (OR: 1.09; 95% CI: 1.02–1.16) and SOFA score (OR: 2.05; 95% CI: 1.41–2.97).

**Conclusion:** Vascular complications and encephalopathies should be searched for in case of neurological deterioration leading to ICU admission in patients with hematological malignancies. Age and SOFA score are associated with higher hospital mortality risk.

**Compliance with ethics regulations:** Yes in clinical research.

### FC-218 Endothelial dysfunction as a component of SARS-CoV-2-related multisystem inflammatory syndrome in children with shock

#### Mehdi Oualha^1^, Richard Chocron^2^, Judith Chareyre^1^, Marion Grimaud^1^, Dominique Lasne^1^, Aurélien Philippe^2^, Charlyne Brakta^1^, Damien Bonnet^1^, François Angoulvant^1^, Julie Toubiana^1^, Maximilien Desvages^1^, Sylvain Renolleau^1^, David M. Smadja^2^, Delphine Borgel^1^

##### ^1^Hôpital Universitaire Necker, Paris (75015), France; ^2^Hôpital Européen Georges Pompidou, Paris (75015), France

**Correspondence:** Mehdi Oualha - mehdi.oualha@aphp.fr

*Annals of Intensive Care* 2021, **11(Suppl 1):**FC-218

**Rationale:** SARS-CoV-2-related multisystem inflammatory syndrome in children (MIS-C) is frequently associated with shock. Endothelial involvement may be one of the underlying mechanisms. We sought to describe endothelial dysfunction during MIS-C with shock and then assess the relationship between the degree of endothelial involvement and the severity of shock.

**Patients and methods/materials and methods:** This is an observational study conducted in a Pediatric Intensive Care Unit (PICU) in a tertiary hospital between April the 1st and May 31st, 2020. Patients aged less than 18 years with SARS-CoV-2-related MIS-C and shock, according to the Centers for Disease Control and Prevention criteria were included. Demographics, clinical, biological data including inflammatory and endothelial markers were collected. Endothelial markers were assayed in the first blood sample collected after PICU admission. Data were expressed as the median [interquartile range (IQR)] or the frequency (percentage). Correlations between the endothelial marker levels and shock severity were assessed by calculation of Spearman’s coefficient.

**Results:** Twenty-eight patients were included. The patients’ age was 9 years [8–11] with a median Pediatric Logistic Organ dysfunction-2 score of 3 [2–4]. Sixteen children presented with cardiogenic shock and distributive shock, 10 presented with cardiogenic shock only and 2 children presented with distributive shock only. The median left ventricular ejection fraction, troponin and lactate levels were at 40% [35–45], 261 ng/mL [131–390] and 3.2 mmol/L [2–4.2], respectively. Twenty-five children received inotropes and/or vasopressors with a median Vasoactive and Inotropic Score (VIS) of 8 [5–28]. Plasma levels of angiopoietin-2 (6.426 pg/mL [2.814–11.836]), sE-selectin (130.405 pg/mL [92.987–192.499]), von Willebrand factor antigen (344% [288–378]) and the angiopoietin-2/angiopoietin-1 ratio (1.111 [0.472–1.524]) were elevated and significantly correlated with the VIS (*r* = 0.45, *p* = 0.016; *r* = 0.53, *p* = 0.04; *r* = 0.46, *p* = 0.013; and *r* = 0.46, *p *= 0.012, respectively). All the children survived and were discharged from the PICU after a median length of stay of 5 days [4–7].

**Conclusion:** The findings of the present study highlighted the relationships between endothelial dysfunction, systemic hyperinflammation, and acute severe cardiovascular manifestations in SARS-CoV-2-related MIS-C. Endothelial dysfunction may constitute one of the mechanisms underlying SARS-CoV-2-related MIS-C with shock.

**References**

1. Iba T, Connors JM, Levy JH. The coagulopathy, endotheliopathy, and vasculitis of COVID-19. Inflamm Res 2020.
2. Belot A, Antona D, Renolleau S, et al. SARS-CoV-2-related paediatric inflammatory multisystem syndrome, an epidemiological study, France, 1 March to 17 May 2020. Euro Surveill 2020; 25.

**Compliance with ethics regulations:** Yes in clinical research.
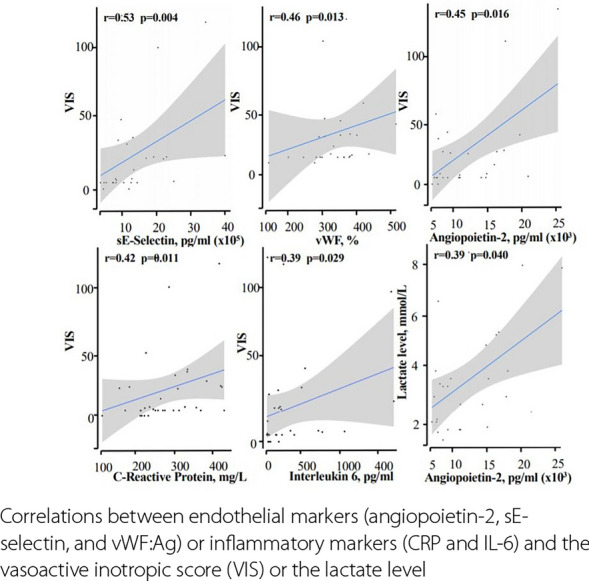


Correlations between endothelial markers (angiopoietin-2, sE-selectin, and vWF:Ag) or inflammatory markers (CRP and IL-6) and the vasoactive inotropic score (VIS) or the lactate level

### FC-219 Hemodynamic assessment by infrared thermography in children with cardiac surgery

#### Armelle Bridier^1^, Monisha Shcherbakova^2^, Atsushi Kawaguchi^1^, Nancy Poirier^1^, Rita Noumeir^2^, Philippe Jouvet^1^

##### ^1^Hôpital Sainte Justine, Montréal, Canada; ^2^Ecole de Technologie Supérieure, Montréal, Canada

**Correspondence:** Armelle Bridier - bridier.armelle@gmail.com

*Annals of Intensive Care* 2021, **11(Suppl 1):**FC-219

**Rationale:** The postoperative period of cardiac surgery is a crucial moment during which low cardiac output can occur. The assessment of hemodynamic status of critically ill children is challenging and the clinician’s estimate of cardiac output is often unreliable. Extremity heat is often used to evaluate peripheral perfusion but remains a very subjective sign. A high thermal gradient between core and peripheral temperature has been correlated with increased systemic vascular resistance and decreased cardiac output, but the results are controversial. The aim of this study is to assess the relationship between thermal gradient, obtained by thermography pictures, and oxygen extraction levels in children in post-operative cardiac surgery.

**Patients and methods/materials and methods:** Children admitted in a single pediatric intensive care unit after a cardiac surgery were included, after obtaining parental consent. Photos of patients with external cooling system, extensive skin disease and extracorporeal support were excluded as this could change the thermal gradient. Infrared images of the patients were taken using the FLIR OnePro camera within 24 h after surgery. The core temperatures (extracted from the internal epicanthus) and toes temperatures were extracted. Temperature gradient between core and extremities (thermal gradient) was calculated and compared to the simultaneous oxygen extraction ratio (O_2_ER: the ratio between arterial and mixed venous oxygen saturations). The correlation was established by the Spearman correlation coefficient.

**Results:** 29 patients of 5 (0–15) months-old were included in the study. One to three thermal images per patient were analyzed for a total of 66 photos. Our patients had cardiopathies that are frequently described in pediatrics. The thermal gradient median was 3.34 °C (1.6–4.3). The O_2_ER median was 35.5% (39–43). A low correlation was observed between thermal gradient and O_2_ER. The Spearman’s rho was 0.24 *p *= 0.049.

**Discussion:** The weak correlation between the two variables could be due to many factors, one of which is a small dataset. The frequent use of vasoactive amines (epinephrin, milrinone) in post-operative cardiac surgery can modify the peripheral circulation and influence on the thermal gradient. The central temperature should also influence the thermal gradient. This weak correlation can also be due to the infra-red technology itself like the Flir one camera lack of performance or other technical aspects.

**Conclusion:** Thermography is potentially an additional tool in multimodal monitoring after cardiac surgery. A more significant correlation would probably observe with a larger dataset, after taking confounding factors into account and with a more reliable technique of thermal image acquisition.

**Compliance with ethics regulations:** Yes in clinical research.
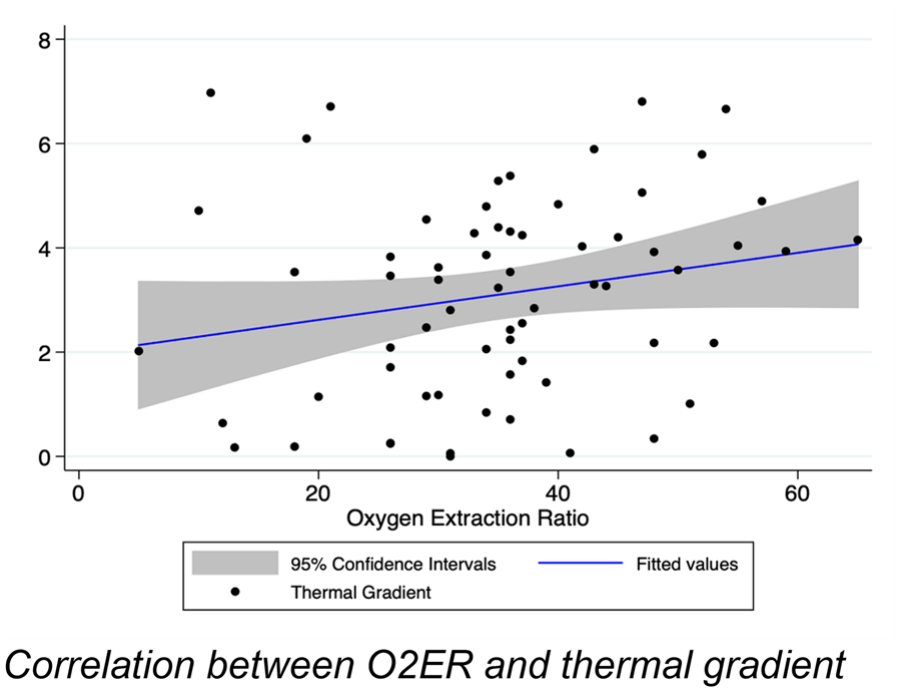


Correlation between O2ER and thermal gradient

### FC-220 Heart rate reduction with ivabradine in infants with tachycardia after open-heart surgery: a pilot study

#### Yael Levy^2^, Julien Jegard^2^, Jean-Bernard Selly^2^, Bernard Kreitmann^2^, Nadir Tafer^2^, Alexandre Ouattara^2^, Pascale Denoyes^2^, Karine Campistron^2^, Marie-Anne Gorry^2^, Elodie Prevot^2^, Jean-Benoit Thambo^2^, Philippe Mauriat^2^

##### ^1^CHU Armand Trousseau, Paris, France; ^2^CHU Haut-Leveque, Bordeaux, France

**Correspondence:** Yael Levy - l.yael@hotmail.fr

*Annals of Intensive Care* 2021, **11(Suppl 1):**FC-220

**Rationale:** Low cardiac output syndrome is a dreadful complication after pediatric cardiac surgery (PCS). While those infants adjust their cardiac output (CO) by increasing their heart rate (HR), catecholamines and systemic inflammatory response syndrome caused by cardiopulmonary bypass may further increase HR up to a potentially detrimental level. This study aimed to evaluate feasibility of HR reduction by oral ivabradine in infants receiving inotropes following PCS and more specifically its impact on CO.

**Patients and methods/materials and methods:** This retrospective, monocentric pilot study of a prospective, open-label cohort included all patients aged 0–18 years receiving inotropes and presenting with sinus tachycardia (HR ≥ 90th percentile for age) despite hemodynamic optimization within 3 to 48 h after PCS requiring cardiopulmonary bypass and aortic cross-clamping. The primary outcome was to evaluate CO 6 h (H6) after administration of ivabradine (baseline). The secondary outcomes were to assess tissue perfusion and to identify adverse events.

**Results:** Twenty-two patients were included. Median age was 3 [0–64] months. From baseline to H6, HR decreased by a mean of 12.3% (169.8 [155.2–179.7] to 148.9 [130.3–155.7] bpm, *p* < 0.0001), mixed venous oxygen saturation remained stable (67.9 [59.0–77.9] to 67.4 [64.5–77.8]%, *p* = 0.71), and subaortic velocity time integral increased significantly (7 [5.5–12] to 11 [7.2–13.1] cm, *p* < 0.05). In one patient, bradycardia (with reduced CO) required use of temporary atrial pacing.

**Conclusion:** Our results suggest that use of ivabradine in infants receiving inotropes following PCS leads to a significant reduction in HR with improved hemodynamics and tissue perfusion.

**Compliance with ethics regulations:** Yes in clinical research.

### FC-221 Risk factors for necrotizing enterocolitis in newborns with duct-dependent congenital heart disease

#### Fedoua El-Louali, Camille Prom, Chloé Allary, Caroline Ovaert, Fabrice Michel

##### Timone enfant, Marseille, France

**Correspondence:** Camille Prom - camille.prom@gmail.com

*Annals of Intensive Care* 2021, **11(Suppl 1):**FC-221

**Rationale:** Necrotizing enterocolitis (NEC) is a well-known complication in newborns. The main risk factors are prematurity and low birth weight. Congenital Heart Disease (CHD), especially those with duct dependent condition, also have an increased risk of NEC. The objective of this study was to identify risk factors for NEC in patient with duct-dependent congenital heart disease.

**Patients and methods/materials and methods:** This is a case–control, retrospective study in a pediatric intensive care unit. Newborns with duct-dependent CHD and NEC, defining the “CHD + NEC” group, were matched to 1:1 (on gestational age, birth weight and antenatal or postnatal diagnosis of heart disease and type of CHD) to those without NEC, a “CHD without NEC” group.

**Results:** Twenty-three infants were included in each group. In univariate analysis, lower diastolic blood pressure (PAD) was a risk factor for NEC (26.1 ± 3.9 mmHg vs. 30.3 ± 5.8 mmHg, *p* = 0.008) while a more negative inflow balance, peritoneal dialysis and adrenaline-pumping were significantly associated with a decreased risk of developing NEC (respectively, *p* = 0.008, *p* = 0.002, *p* = 0.007, *p* = 0.017). In multivariate analysis, diastolic blood pressure (PAD) ≤ 30 mmHg was an independent risk factor of NEC (OR-8.70; 95% CI [1.46–53.50], *p*-0.019) (Fig. 1). In CHD + NEC group, mortality, lenght of intensive care unit stay and lenght of hospital stay were significantly higher (34.8% vs. 8.7%, *p*-0.035; 28 [14–42] days vs. 9 [6–18] days, *p* < 0.0001; 59 [39–103] days vs 23 [20–45] days, respectively, *p* < 0.0001)

**Conclusion:** Diastolic blood pressure lower than 30 mmHg was an independent risk factor for NEC in newborns with duct-dependent CHD.

**Compliance with ethics regulations:** N/A.
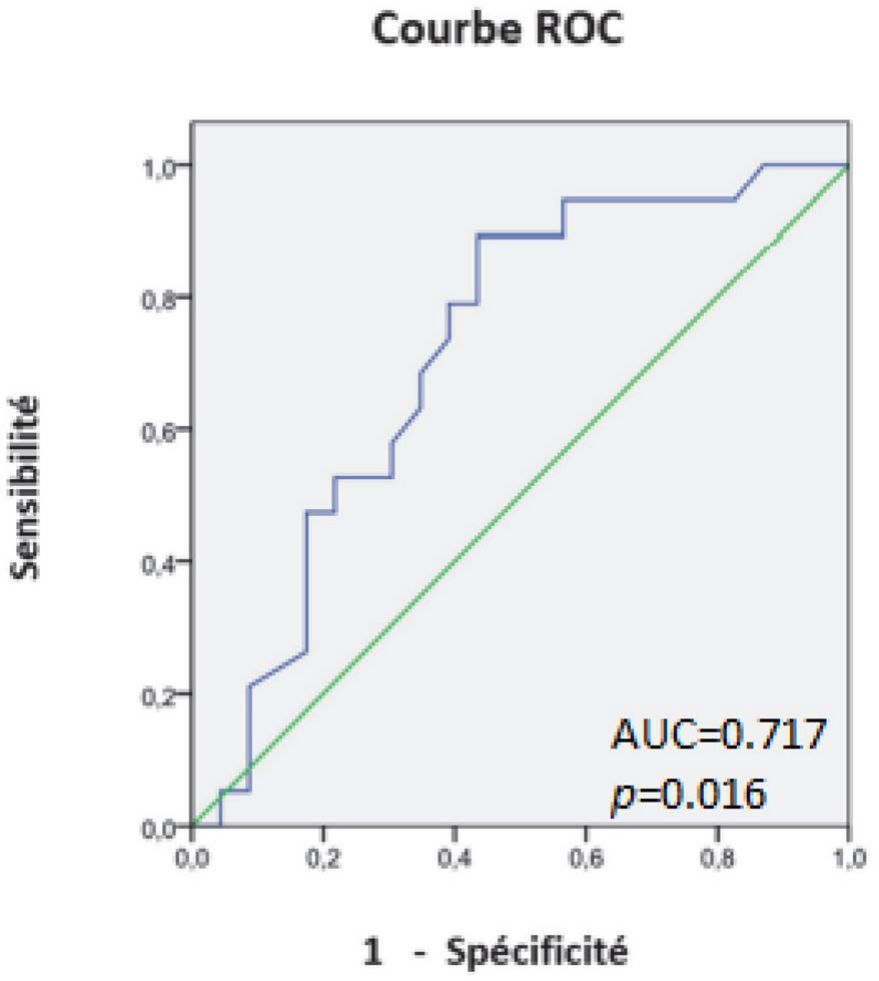


ROC analysis of lower diastolic blood pressure and risk of NEC

### FC-222 Severe acute respiratory distress syndrome in children: retrospective analysis of a 16-year cohort

#### Sophie Beldjilali, Fabrice Michel

##### APHM, Marseille, France

**Correspondence:** Sophie Beldjilali - sophie.beldjilali@ap-hm.fr

*Annals of Intensive Care* 2021, **11(Suppl 1):**FC-222

**Rationale:** The acute respiratory distress syndrome (ARDS) is a rapid progressive acute hypoxemic respiratory failure resulting from an alteration of the alveolar-capillary barrier by direct or indirect pulmonary aggression. The management of this rare and serious pathology in children is largely based on adult studies. There is a lack of pediatric evidence. Furthermore, few data are focusing on severe ARDS. The main objective of this study was to analyze epidemiology and treatments used for severe ARDS in children. The secondary outcome was to determine factors associated with mortality.

**Patients and methods/materials and methods:** We led a retrospective bicentric study, in two pediatric intensive care units (PICU). Inclusion criteria were children between 28 days and 18 years old hospitalized in PICU and having presented severe ARDS define as index oxygenation (OI) ≥ 16 or PaO2/FiO2 < 100 when OI was not available. Period of inclusion was going from 2003 to 2018.

**Results:** 148 children were included in the study, corresponding to a prevalence of 0.99%, stable over studied years. Etiologies were dominated by infections (55%). There was a stable prevalence of mortality with thirty-two percent over time despite a change in treatment practices. Multivariate analysis identified several factors independently associated with mortality: high weight (OR 1.1 IC95 [1.01; 1.13]; *p* = 0.019), liver failure (OR 31 IC95 [1.93; 505]; *p* = 0.015) and kidney failure (OR 21 IC95 [2.29; 190]; *p* = 0.007), maximum arterial CO2 (OR 1,04 IC95 [1.01; 1.06]; *p* = 0.002), maximum arterial lactatemia (OR 1.48 IC95 [1.13; 1.93]; *p* = 0.004), tidal volume (OR 3.1 IC95 [1.37; 6.8]; *p* = 0.006) and mean airway pressure (OR 1.2 IC95 [1.01; 1.38]; *p* = 0.036).

**Conclusion:** This study is the largest French retrospective cohort on children’s ARDS. We identified independent factors associated with mortality never described before in children, which are likely to be indicative of the initial severity of the clinical presentation. They could help clinicians for a better management and a more accurate parental information. These results require confirmation by prospective data.

**Compliance with ethics regulations:** Yes in clinical research.

### FC-223 Acute respiratory distress management in infants in intensive care unit

#### Abdeljabbar Marhfour, Elmehdi Soussane, Mohamed Anas Fahdi, Samira Kalouch, Khalid Yaqini, Chlilek Abdelaziz

##### CHU ibn rochd, Casablanca, Maroc

**Correspondence:** Abdeljabbar Marhfour - marabdo@gmail.com

*Annals of Intensive Care* 2021, **11(Suppl 1):**FC-223

**Rationale:** Acute respiratory distress in infants is a frequent and high-risk emergency in daily practice. It requires urgent analysis to assess the severity, to determine the aetiology and to start adequate treatment immediately. Our purpose is to evaluate the incidence, etiologies, management and evolution in ICU.

**Patients and methods/materials and methods:** Our work is based on a prospective descriptive study of 122 cases of respiratory distress in infants at the pediatric intensive care unit during 1 year from July 1st 2018 to June 30th 2019. We included all infants over 28 days of age and under 24 months with dyspnea with or not signs of struggle. All infants with bronchopneumopathy without dyspnea were excluded.

**Results:** The incidence was 7.26%. The average age was 8 months with a range of [1–24]. The sex ratio was 1.39. The diagnosis was guided by patient medical history, physical examination and chest X-ray. A chest X-ray was requested in all patients with pathological results in 83.6% of cases. The etiologies were mainly represented by viral bronchiolitis (25.6%), congenital heart disease (15.6%), whooping cough (10.7%), foreign body inhalation (9.0%) and acute severe asthma (6.6%). All infants received symptomatic and etiological treatment. Despite appropriate treatment, the mortality represented 15.7%.

**Conclusion:** Acute respiratory distress in infants is due to many causes and viral bronchiolitis remain the most frequent one. The diagnosis is clinical and guided by chest X-ray and should not delay the treatment.

**Compliance with ethics regulations:** Yes in clinical research.

### FC-225 Impact of dexamethasone use in severe COVID-19-induced acute kidney injury

#### Arthur Orieux, Pierre Khan, Renaud Prevel, Mathieu Acquier, Didier Gruson, Sébastien Rubin, Alexandre Boyer

##### CHU de BORDEAUX, Bordeaux, France

**Correspondence:** Arthur ORIEUX - arthurorieux@gmail.com

*Annals of Intensive Care* 2021, **11(Suppl 1):**FC-225

**Rationale:** Since the first wave of COVID-19, the management of patients with severe COVID-19 in intensive care units (ICU) has changed with the widespread use of dexamethasone (DXM). We have reported a high incidence of acute kidney injury (AKI) in severe COVID-19 patients. As lung injury, specific SARS-CoV-2 inflammatory process was previously suggested in AKI pathogenesis susceptible to be improved by DXM. We have reported a chronic kidney disease (CKD) incidence of 15%, 3 months after COVID-19-induced AKI. All of these suffered from acute kidney disease (AKD) before CKD highlighting the impact of this inflammatory period.

**Patients and methods/materials and methods:** Aim of this report was to evaluate clinical effect of DXM in the kidney involvement in severe COVID-19 patients. We carried out a prospective study in a medical ICU from March to December, 2020. All patients admitted for a severe COVID-19 were included. DXM was exclusively used during the second wave (6 mg/day, for 10 days). Patients treated by DXM who developed AKI before or in the first 24 h after corticosteroid administration were excluded from the analysis.

**Results:** 126 patients were enrolled. 21/126 patients (17%) were excluded because of AKI prior or in the first 24 h to DXM administration. DXM administration was used in all patients in the second wave and in none of the first wave patients. In the 105 patients included, median age was 63 ± 11 years, 64/105 (61%) suffered from hypertension and 10/105 (10%) had previous CKD. 61/105 (58%) patients developed AKI and 24/105 (23%) developed AKD (Table 1). In univariate analysis, the use of DXM (OR = 0.16 [0.07–0.37]), hypertension (odd ratio (OR) = 2.62 [1.18–5.95]), SAPSII (OR = 1.04 [1.01–1.07]), mechanical ventilation in the first 24 h (OR = 8.6 [3.58–22.42]), and intravenous fluid therapy in the first 24 h (OR = 1.59 [1.13–2.36]) were significantly associated with AKI. In multivariate analysis, mechanical ventilation in the first 24 h (OR = 4.42 [1.50–13.06]) and DXM use (OR = 0.24 [0.08–0.75]) were significantly associated with AKI. In multivariate analysis, only prior CKD (OR = 19.36 [3.17–118.36]) was significantly associated with AKD.

**Conclusion:** Our study suggests an independent effect of DXM in the risk of AKI in severe COVID-19 patients. This result needs to be confirmed in larger studies. It could suggest an important impact of inflammatory state in the pathogenesis of COVID-19-induced AKI. AKD is a period characterized by a highly inflammatory condition which seems to be the cornerstone of the risk of CKD development. Lack of effect of DXM in AKD incidence was probably due to a lack of power.

**Compliance with ethics regulations:** Yes in clinical research.

### FC-226 Incidence and risk factor of AKI in severe COVID-19: a time-dependent competing risk analysis

#### Antoine Marchiset, Valérie Serazin, Claire Pichereau, Omar Ben Hadj Salem, Lionel Lima Da Silva, Siu-Ming Au, Yann Loubières, Jan Hayon, Julia Gross, Hervé Outin, Matthieu Jamme

##### Centre hospitalier de Poissy - St Germain en Laye, Poissy, France

**Correspondence:** Antoine Marchiset - anmarchiset@yahoo.fr

*Annals of Intensive Care* 2021, **11(Suppl 1):**FC-226

**Rationale:** Main feature of the COVID-19 outbreak is an acute respiratory failure linked with diffuse alveolar damages. However, there are reports of increased rates of acute kidney injury (AKI) ranging from 5% to 36% in hospitalized patients and rising up to 52.2% in ventilated ICU patients ^1,2^. Mechanism, risk factors and outcome of AKI related to COVID-19 are not fully understood.

**Patients and methods/materials and methods:** We conducted an observational study to assess risk factors of AKI in patients admitted in ICU for COVID-19. All patients admitted in our ICU with COVID-19 confirmed by positive SARS-CoV-2 RT-PCR or COVID-19 serology between March 1st 2020 and May 30th 2020 were included. We collected all clinical and biological data within the 14 first days following admission. Primary outcome was severe AKI defined by a KDIGO stage ≥ 2. Statistical analysis was carried out by using a multivariate time-dependent Cox cause-specific proportional hazard (CSH) model. Missing values of covariates in the multivariable model were handled by multiple imputations with chained equations. All tests were two-sided, with *P* values of 0.05 or less denoting statistical significance.

**Results:** Seventy-seven patients were included in the study population (Table 1). Median age of patients was 70 [58–74] including 58 (75.3%) men and 19 (24.7%) women. Invasive mechanical ventilation were realized in 60 (77.9%) patients and vasopressors used in 45 (58.4%) patients. AKI occurred in 28 (36.4%) patients in a median time of 6 [3–8] days following ICU admission. Time-dependant Cox cause specific model showed that hemodynamic SOFA score (CSH = 1.63 [1.23–2.16], *p* < 0.001), arterial pCO2 (CSH = 1.2 [1.04–1.39], *p* = 0.02 per increase of 5 mmHg) and history of arterial hypertension (CSH = 2.46 [1.04–5.84], *p* = 0.04) were associated with AKI.

**Conclusion:** Our findings show that severe AKI is a common complication observed in ICU patients admitted for COVID-19. Respiratory status with hypercapnia, hemodynamic instability and history of hypertension are associated with higher risk of AKI in COVID-19 patients. Severity of patient’s respiratory status, highlighted by respiratory acidemia and hypercapnia, is a novel risk factor of AKI in ICU patients. Further studies are needed to confirm those findings and understand underlying mechanism of this pulmonary–kidney interaction.

**References**

1. Hirsch JS, Ng JH, Ross DW, et al. Acute kidney injury in patients hospitalized with COVID-19. Kidney Int. 2020;98(1):209–18. 10.1016/j.kint.2020.05.006.
2. Cheng Y, Luo R, Wang K, et al. Kidney disease is associated with in-hospital death of patients with COVID-19. Kidney Int. 2020;97(5):829–38. 10.1016/j.kint.2020.03.005.

**Compliance with ethics regulations:** Yes in clinical research.
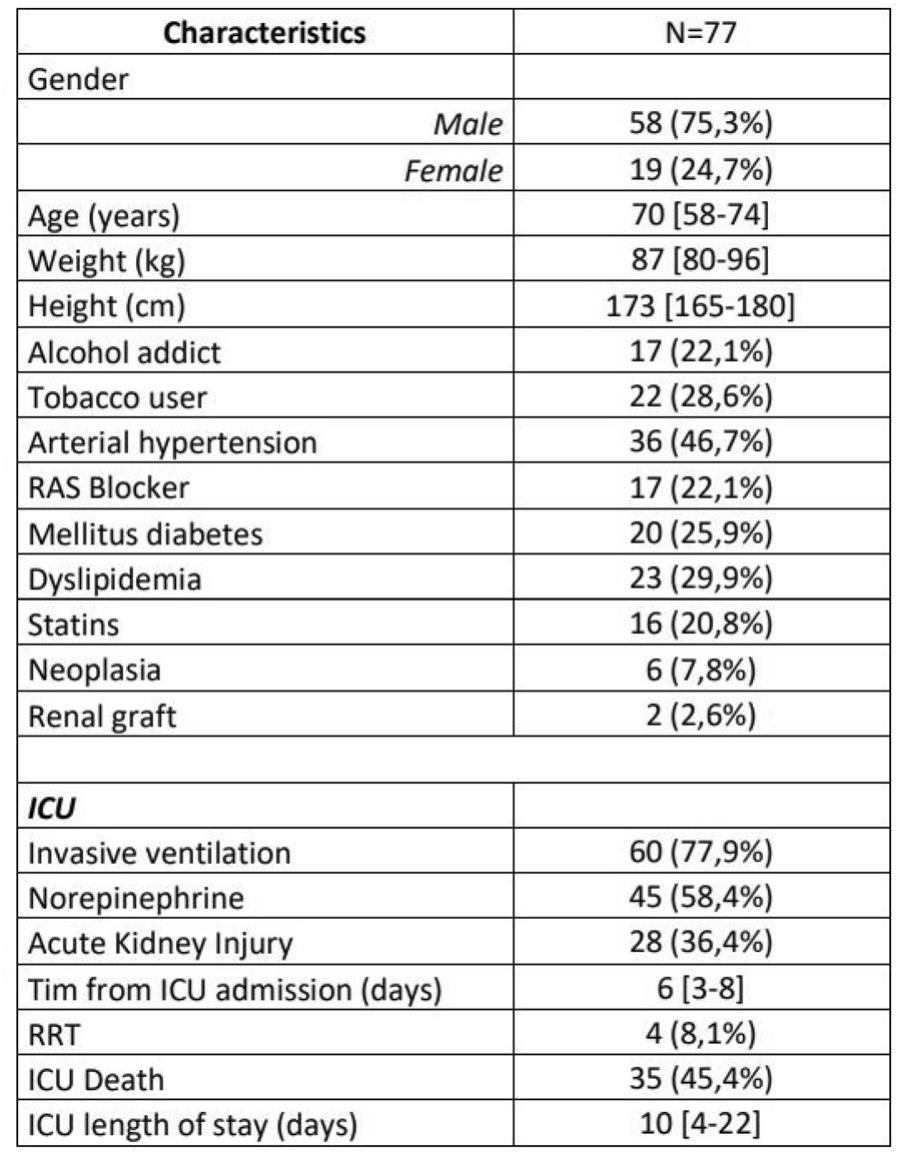


Table 1 Patients characteristics

### FC-227 Incidence and impact of acute kidney injury in COVID-19-infected patients

#### Sana Kharrat, Kamilia Chtara, Olfa Turki, Ilef Alila, Salma Jerbi, Najeh Baccouch, Hedi Chelly, Chokri Ben Hamida, Mabrouk Bahloul, Mounir Bouaziz

##### CHU Habib Bourguiba, Sfax, Tunisie

**Correspondence:** Sana Kharrat - sanakharrat15@hotmail.com

*Annals of Intensive Care* 2021, **11(Suppl 1):**FC-227

**Rationale:** Infection with COVID-19 is primarily a respiratory illness, but other organs including the kidneys are often affected. However, the incidence of acute kidney injury (AKI) reported in COVID-19 patients is extremely variable and the renal complications of COVID-19 are not yet well explored. This study aimed to identify the incidence of AKI in patients with COVID-19 and to evaluate it’s impact on patient outcomes.

**Patients and methods/materials and methods:** We conducted a retrospective study including critically ill patients with confirmed SARS-CoV-2 infection in an intensive care unit during 4 months. AKI was defined according to the serum creatinine criteria in the Kidney Disease: Improving Global Outcomes (KDIGO) guidelines.

**Results:** A total of 178 patients were admitted during the study period. The mean age was 62 ± 12.8 years, and 132 (74.2%) of patients were male. Among these patients, 84 (47.2%) had hypertension, 76 (42.7%) had diabetes and 17 (9.5%) had chronic lung disease. 40% of patients suffered from obesity. Mean SAPSII score on ICU admission was 34 ± 14.9 and mean SOFA score was 5 ± 2.7. During hospitalization, 86(48.3%) patients developed AKI. Among these patients, 33 (18.5%) had AKI stage 1, 29 (16.3%) had AKI stage 2, 24 (13.5%) had AKI stage 3 and 16 patients (9%) received renal replacement therapy. Risk factors for AKI in COVID-19 in our study were hypertension (*p* = 0.01), diabetes (*p* < 0.001), a SOFA score > 5 (*p* < 0.001), SAPSII > 35 (*p* < 0.001), the use of diuretic drugs (*p* = 0.023) and the use of vasopressors (*p* < 0.001). AKI patients were also more likely to require mechanical ventilation (69.4% versus 23%, *p* < 0.001; OR = 7.5 [3.8–14.8]) compared to COVID-19 patients without AKI. AKI was also significantly associated with death (80.2% versus 26%; *p* < 0.001, OR = 11.5 [5.6–23.3]); the mortality in AKI stage 1, stage 2, and stage 3 was 75%, 82% and 83%, respectively.

**Conclusion:** AKI is a common serious complication in critically ill patients and is associated with higher mortality, longer hospital stay and increases the need of ventilator support.

**Compliance with ethics regulations:** N/A

### FC-228 Acute kidney injury in severe SARS-CoV-2 infection: a report of experience

#### Guillaume Louis, Nicolas Leberre, Rostane Gaci, Yoann Picard, Noucham Mellati, Thibaut Belveyre, Vincent Dinot

##### CHR METZ THIONVILLE, Ars-Laquenexy, France

**Correspondence:** Guillaume Louis - gus_louis@yahoo.fr

*Annals of Intensive Care* 2021, **11(Suppl 1):**FC-228

**Rationale:** Acute kidney injury (AKI) has been reported as a severe complication of COVID-19 [1]. Few of studies have reported AKI stages.

**Patients and methods/materials and methods:** We report the incidence, risk factors, severity, and prognosis of AKI in an observational study including 333 patients with severe SARS-CoV-2 infection in three ICUs between March 4th and November 30th, 2020. AKI was diagnosed and classified according to the KDIGO classification. We collected demographic data, comorbidities, exposition to nephrotoxic agents, need for supportive therapy and death 28 days after ICU admission. We compared baseline patient characteristics between patients with or without AKI. We excluded patients with end stage kidney-disease (*n* = 7), patients hospitalized for < 48 h (*n* = 8), patients with missing date (*n* = 2), and patients transferred to another ICUs (*n* = 42).

**Results:** Among the 274 remaining patients, 117 (43%) presented AKI. The median time from ICU admission to highest AKI stage was 6 (IQR 2–13) days. Patients with AKI were distributed as follows: stage 1: 33%, stage 2: 21% and stage 3: 46%. A total of 33 patients required renal replacement therapy. By univariate analysis, age > 65 years, chronic hypertension, diabetes mellitus, chronic kidney disease, chronic use of ACEI or ARB, cardiovascular disease, mechanical ventilation, vasopressor therapy were predictors of AKI. Independent predictors, by multivariate analysis, included chronic kidney disease and mechanical ventilation. Mortality was significantly higher in AKI patients (58% vs 16% *p* < 0.001) compared to patient with normal renal function. Figure 1 shows that mortality increased along with AKI severity.

**Discussion:** As expected, our results show that AKI is frequent in COVID-19 critically ill patients and confer a poor outcome. However, our study is one of the largest published to date reporting AKI stages specially in critically ill patients. The present results underline the need of mechanical ventilation support as a risk factor for the development of AKI. Recent evidence has established that kidney involvement is parallel to the severity of the underlying lung involvement [2].

**Conclusion:** Due to its high prevalence and severity, early detection of impaired renal function, careful use of nephrotoxic drugs and targeted treatment of these potential pathogenic mechanisms can help prevent AKI and improve the prognosis of patients severely infected with COVID-19. The pathophysiological mechanisms of severe AKI specially in Covid-19 patients with severe ARDS, e.g., detrimental mechanical ventilation strategies such as high PEEP levels, warrant further research.

**References**

1. Gabarre P, Dumas G, Dupont T, Darmon M, Azoulay E, Zafrani L. Acute kidney injury in critically ill patients with COVID-19. Intensive Care Med 2020. 10.1007/s00134-020-06153-9.
2. Hirsch JS, Ng JH, Ross DW, Sharma P, Shah HH, Barnett RL, et al. Acute kidney injury in patients hospitalized with COVID-19. Kidney International 2020;98:209–18. 10.1016/j.kint.2020.05.006.

**Compliance with ethics regulations:** Yes in clinical research.
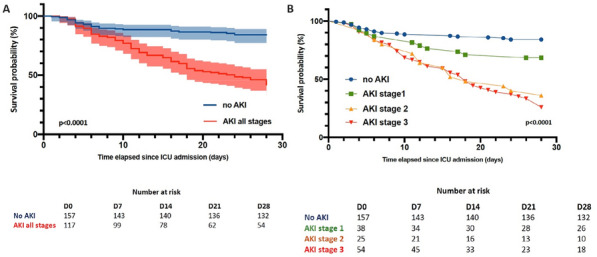


Fig. 1 Kaplan–Meier curves for day-28 survival in: **A** patients with AKI (*n* = 117) and without AKI (*n* = 157) and **B** with AKI stage 1 (*n* = 38), 2 (*n* = 25) and 3 (*n* = 54). Log rank test was performed, fraction survival error bars was expressed as 95%CI (**A**)

### FC-229 Acute kidney injury in COVID-19 patients: risk factors and prognosis

#### Selma Essghaier, Imen Talik, Hedia Ben Ahmed, Fatma Essafi, Moez Kaddour, Takoua Merhabene

##### hôpital régional Zaghouan, Zaghouan, Tunisie

**Correspondence:** Imen Talik - talik.imen2013@gmail.com

*Annals of Intensive Care* 2021, **11(Suppl 1):**FC-229

**Rationale:** Initial reports of COVID-19 focused on the severe acute respiratory syndrome seen in critically ill patients. However, it became apparent that many COVID-19 patients also displayed kidney abnormalities, primarily acute kidney injury (AKI). Incidence of AKI still poor documented in Tunisia. In this study, we report our experience with AKI in COVID-19 patients and asses its incidence, risk factors, and outcomes.

**Patients and methods/materials and methods:** In this single-center, retrospective cohort study, we analyzed data of COVID-19 patients admitted to the ICU of a tertiary hospital, from March 23th 2020 to January 31st 2021. Demographics characteristics, comorbidities, clinical, paraclinical, therapeutics and outcomes were collected. Patients younger than 18 years or those with end stage kidney disease were excluded. AKI was defined according to KDIGO criteria.

**Results:** 121 patients were enrolled, median age was 64 ± 12 years (IQR, 56–70), sex ratio = 1.5, Medians of SAPS II and APACHE II scores were, respectively, 26 ± 8 and 8 ± 3. Hypertension (48%) and diabetes (35%) were the most frequent underlying diseases. Fifty patients (41%) required invasive mechanical ventilation. Among 121 patients admitted with acute respiratory failure due to Covid-19, incidences of AKI reported on the first day of ICU admission was 19%. During hospitalization, 37 patients (30%) developed secondary AKI (10 kept their renal failure from admission). Hospital acquired AKI were classified on stage 1 of KDIGO classification in 19 cases (51%), stage 2 in 10 patients (27%) and stage 3 in 8 patients (22%). Identified risk factors of developing AKI were: history hypertension (HR 3.55; CI 0.32–39.34), use of angiotensin-converting enzyme inhibitors (HR 3.33; CI 0.34–32.05), elevated glycemia at admission ≥ 1.8 g/dl (HR 1.68; CI 0.46–6.1), lymphocyte count ≤ 950 elt/ml (HR 3.16; CI 0.78–12.8), extensive lung scan lesions (≥ 65%)(HR 1.64; CI 0.41–6.44), need for invasive mechanical ventilation (HR 1.20; CI 0.08–17.24), and shock (HR 34.5; CI 2.1–564.25). Occurrence of AKI was significantly associated with longer length of ICU stay (12 ± 5.8 vs 8.7 ± 5.4 days, *p* = 0.003) and extended duration of invasive mechanical ventilation (8.4 ± 4 vs 5.8 ± 3.8 day, *p* = 0.035). ICU mortality in AKI group was significantly higher (86% vs 25%, *p* < 0.001).

**Conclusion:** Our study showed that occurrence of AKI in ICU hospitalized COVID-19 patients, was common and carried a higher mortality and longer ICU length of stay.

**Compliance with ethics regulations:** Yes in clinical research.

### FC-230 Acute kidney injury during COVID-19 in ICU patients: incidence, risk factors and outcome

#### Ahlem Trifi^1^, Asma Mehdi^1^, Oussema Benjima^2^, Emna Abid^1^, Dorra Nouri^2^, Yosri Masseoudi^2^, Yosr Touil^1^, Sami Abdellatif^1^, Adel Ammous^2^, Salah Ben Lakhal^1^

##### ^1^Medical ICU, hospital of la Rabta, Faculty of Medicine of Tunis, Tunis, Tunisie; ^2^Department of Anesthesiology, hospital of la Rabta, Faculty of Medicine of Tunis, Tunis, Tunisie

**Correspondence:** Ahlem Trifi - trifiahlem2@gmail.com

*Annals of Intensive Care* 2021, **11(Suppl 1):**FC-230

**Rationale:** Acute kidney injury (AKI) is a clinical syndrome that complicates the course and worsens the prognosis of ICU patients. There are numerous potential causes of AKI, mainly related to a focal mismatch between oxygen delivery (due to impaired microcirculation) to the nephrons. The incidence, prognosis and determinants of AKI in ICU patients with COVID-19 remain unclear. The aim was to determine the incidence, the severity degree of AKI, the risk factors and prognosis during hypoxemic pneumonia related to COVID-19.

**Patients and methods/materials and methods:** Retrospective comparative case/control study. All medical records of patients hospitalized in ICU (medical and surgical) for COVID-19 pneumonia (complicated or not with AKI) were analyzed.

**Results:** Among 109 patients admitted in the 2 ICUs for COVID-19, 48 (44%) developed AKI during hospitalization: 33 were males (68%) and the median age was 69 years [61–77]. 11 (23%), 9 (19%) and 28 (58%) were classified as being at stage 1, 2 and 3, respectively. 8 patients (17%) received renal replacement therapy. AKI occurred within a median of 4 days [1–9]. Patients in the AKI group were older with an increased rate of white blood cells and CRP. Sepsis, nephrotoxic drug, vasopressors and invasive ventilation were more frequent in this group. Logistic regression showed that vasopressors use was an independent factor associated with AKI in COVID-19 patients (Table). Renal recovery was identified among 3 patients with AKI (6.25%). Duration of ventilation and ICU stay were similar in AKI group and non-AKI group (6 [3–8] vs 3 [2–8], *p* = 0.2 and 7 [5–10] vs 8 [4–15], *p* = 0.59). Mortality was significantly higher in AKI patients (85% vs 39.4%, *p* < 0.001) and AKI increased the death risk by 6 (*p* = 0.009). P/F ratio < 70 (at admission or during hospitalization with OR = 7.5 and *p* = 0.006), invasive ventilation (OR = 5.6, *p* = 0.017) and vasopressors use (OR = 16, *p* < 0.001) were also independent factors of mortality.

**Conclusion:** The incidence of AKI among patients with critical illness of COVID-19 was important. Several factors were shown to contribute to its occurrence and vasopressors use was an independent factor. This complication was highly associated with mortality.

**Compliance with ethics regulations:** Yes in clinical research.
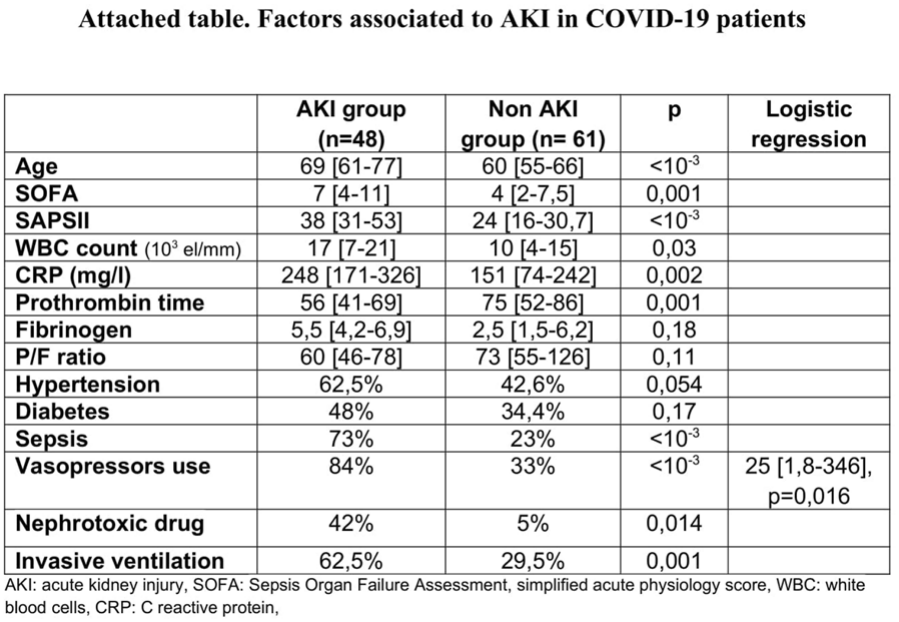


Factors associated to AKI in COVID-19 patients

### FC-231 Acute kidney injury (AKI) in critical ill patients with viral pneumonia: is SARS-CoV-2 at higher risk?

#### Antoine Morel^1^, Flavie Carniato^1^, François Fer^1^, Maxime Debache^2^, Alexis Hermans^1^, Léo Souletie^1^, Marie-Claire Diemoz^2^, Cecilia Tabra^1^, Emmanuelle Mercier^1^, Tinhinan Mezdad^1^, Camille Vissac^1^, Pierre Chaffiotte^1^, Rusel Leon^1^, Tommaso Maraffi^1^, Jerome Cecchini^1^, Frederique Schortgen^1^

##### ^1^Centre Hospitalier Intercommunal de Créteil, Réanimation Médicale, Paris, France; ^2^Centre Hospitalier Intercommunal de Créteil, Service d’Anesthésie, Paris, France

**Correspondence:** Antoine Morel - antoine.morel77@aphp.fr

*Annals of Intensive Care* 2021, **11(Suppl 1):**FC-231

**Rationale:** High incidence of both AKI and post-AKI chronic kidney disease have been reported in patients with SARS-CoV-2 infection. Specific mechanisms as Fanconi syndrome have been, therefore, supposed but remain unproven. No study had compared AKI incidence between critically ill patients with viral pneumonitis related to SARS-CoV-2 from other viral pneumonitis.

**Patients and methods/materials and methods:** Monocentric retrospective cohort study including 46 consecutive COVID patients in whom AKI incidence, characteristics and recovery were recorded. COVID patients were compared to an historical control group of patients admitted to viral pneumonia requiring respiratory support within the last 3 years. 53 controls were identified (with influenza virus in 74%). AKI definition was based on KDIGO creatinine criteria. Full renal recovery was defined as no AKI criteria (KDIGO < 1) among survivors. The risk of AKI occurrence during ICU stay was adjusted for patient’s severity and virus type.

**Results:** AKI occurred in 28 patients with COVID. Early AKI < 48 h (*n* = 14) was associated with more inflammation and rhabdomyolysis, while late AKI (*n* = 14) was associated with the severity of hemodynamic and respiratory failures. Proximal tubulopathy with complete criteria for a Fanconi syndrome was identified in one patient only. AKI was more frequent in COVID patients (61% vs 42%, *p* = 0.055) with KDIGO-3 (35% vs 8%, *p* = 0.001) and more renal replacement therapy (20% vs 0%, *p* = 0.001). Hospital mortality was similar between COVID-19 and no COVID-19 patients (22% vs 13%, *p* = 0.21). 17/18 COVID-19 and 15/18 non-COVID-19 survivors with AKI had full renal recovery with a mean follow-up of 63 ± 32 days. Characteristics of patients with and without AKI are indicated in the table. In multivariate logistic regression analysis, SARS-CoV-2 infection was not independently associated with AKI development (OR = 1.12, 95% CI 0.37–3.45, *p* = 0.84).

**Conclusion:** Compared to patients with viral pneumonitis from other virus, SARS-CoV-2 is not associated with a significant higher risk of AKI in critically ill patients. Despite a more severe form of AKI in COVID patients, the odds of recovery appear similar.

**Compliance with ethics regulations:** Yes in clinical research.
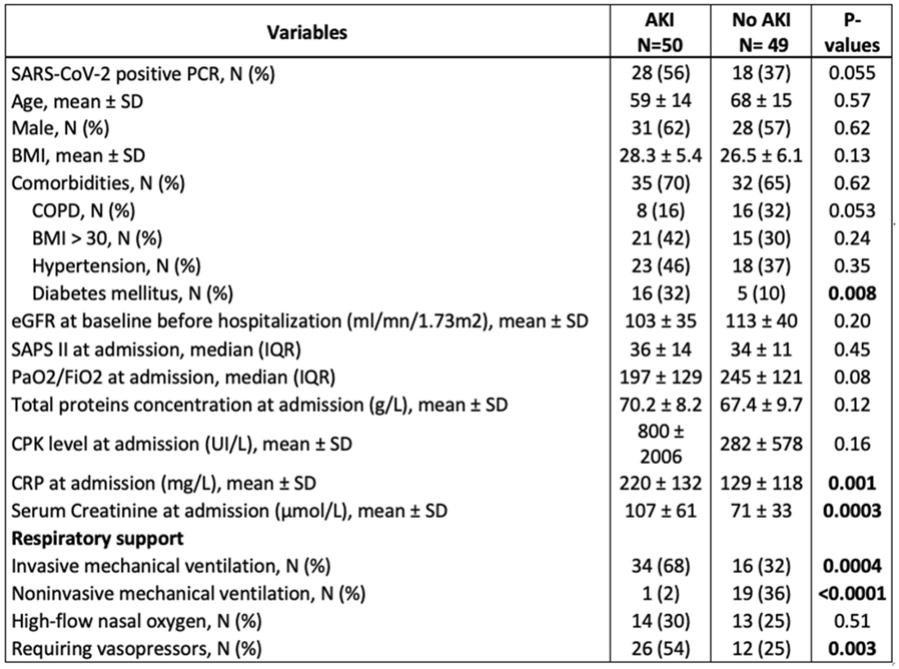


Table Comparison between patients with and without AKI among the entire cohort (*n* = 99)

### FC-232 High-flow nasal oxygen during breaks off non-invasive ventilation for severe exacerbation of chronic respiratory failure

#### Oussama Jaoued, Rim Merzougui, Hajer Nouira, Sabrina Chaouch, Rim Gharbi, Habiba Ben Sik Ali, Mohamed Fekih Hassen, Souheil Atrous

##### service de réanimation médicale hôpital Taher Sfar, Mahdia, Tunisie

**Correspondence:** Oussama Jaoued - oussamajaoued@gmail.com

*Annals of Intensive Care* 2021, **11(Suppl 1):**FC-232

**Rationale:** Non-invasive ventilation (NIV) is the first choice of ventilatory modality for acute exacerbation of chronic obstructive pulmonary disease. Standard oxygen therapy (SO) (nasal cannulas and simple masks) is conventionally used during breaks off NIV. High-flow nasal therapy (HFNT) is known to wash out nasopharyngeal dead space and to provide a small positive end-expiratory pressure. The aim of the study is to compare SO vs HFNT during breaks off NIV in severe exacerbation of chronic respiratory failure (CRF).

**Patients and methods/materials and methods:** It is a randomized controlled study performed between 2018 and 2019. Inclusion criteria: age > 18 years, acute NIV for an anticipated time > 12 h due to severe exacerbation of CRF. Exclusion criteria: usual contra-indications to NIV, inability to obtain consent and patient who underwent into tracheal intubation before the end of the first session of HFNT. Subjects were randomized (1:1) to receive SO or HFNT during breaks off NIV. The primary outcome was time to NIV weaning. Secondary outcomes included changes of respiratory rate (RR), and arterial blood gas (ABG) parameters before the second session of NIV (after the first session of SO or HFNT), on day 2, 3 and 4 of randomization.

**Results:** During the study 32 patients were included (15 assigned to SO group and 17 to HFNT group). The median age was 66 [62–74] years and the median APACHEII was 14 [10–17]. The common causes of severe exacerbation were tracheobronchitis (72%) and pneumonia (12.5%). NIV failure was observed on 8 patients (25%). The median duration of ABG normalization was 4 days. The median duration of NIV was 8 days. NIV failure was observed in 8 patients. Median length of stay was 11 days. At randomization, comorbidities, RR and ABG parameters were similar between groups. NIV duration was not different within HNFT and SO group (8 [6–10] vs 9 [5–12] days *p* = 0.6). RR was comparable in both HFNT and SO group during the first session of NIV (28 [24–28] vs 26 [22–28] *p* = 0.4) and before the second session of NIV (23 [22–28] vs 26 [24–28] *p* = 0.2). ABG parameters were similar among groups. There were no statically difference between HFNT group and SO group in PCO2 changes after the first break session (64 [60–70] mmHg in HFNT vs 70 [62–87] in SO group *p* = 0.1), on day 2, 3, and 4.

**Conclusion:** Our study shows that the use of HFNT instead of SO between the NIV sessions does not reduce the duration of NIV.

**Compliance with ethics regulations:** Yes in clinical research.

### FC-233 Oxygen reserve index for noninvasive early hypoxemia detection during endotracheal intubation in intensive care: the prospective observational NESOI study

#### Hugo Hille, Aurelie Le Thuaut, Emmanuel Canet, Jeremie Lemarie, Laura Crosby, Gregoire Ottavy, Charlotte Garret, Maelle Martin, Amelie Seguin, Pauline Lamouche Wilquin, Jean Morin, Olivier Zambon, Arnaud Felix Miaihle, Jean Reignier, Jean Baptiste Lascarrou

##### CHU Nantes, Nantes, France

**Correspondence:** Jean Baptiste Lascarrou - jeanbaptiste.lascarrou@chu-nantes.fr

*Annals of Intensive Care* 2021, **11(Suppl 1):**FC-233

**Rationale:** To evaluate the ability of the oxygen reserve index (ORI) to predict the occurrence of mild hypoxemia (defined as SpO2 < 97%) during endotracheal intubation (ETI) of patients in the intensive care unit (ICU).

**Patients and methods/materials and methods:** This observational single-center study included patients without hypoxemia (defined as SpO2/FiO2 > 214) who required ETI in the ICU. Patients were followed during preoxygenation and ETI then until hospital discharge and/or day 28. We recorded cases of mild hypoxemia, moderate (SpO2 < 90%) and severe (SpO2 < 80%) hypoxemia, moderate arterial hypotension (systolic arterial pressure < 90 mmHg), esophageal intubation, aspiration, cardiac arrest, and death.

**Results:** Between September 2018 and July 2020, 56 patients were included prospectively and 51 patients were analyzed. Twenty patients had mild hypoxemia between the end of preoxygenation and the end of intubation; in 10 of these patients, the decrease in SpO2 below 97% was preceded by an ORI < 0.4, the median time difference being 81 s (interquartile range, 34–146). By multivariable analysis, a higher ORI (by 0.1 increase) value during preoxygenation was associated with absence of hypoxemia (odds ratio, 0.76; 95% confidence interval, 0.61;0.95; *p* = 0.0141).

**Conclusion:** The 81 s [34–146] median time between the ORI decrease below 0.4 and the SpO2 decrease below 97% during apnea may allow preventive action. A higher ORI value during preoxygenation was independently protective against hypoxemia. Whether these findings also apply to hypoxemic patients, and the clinical impact of a preoxygenation strategy based on ORI monitoring, remain to be evaluated prospectively.

**Compliance with ethics regulations:** Yes in clinical research.

### FC-234 Pulse oximetry cannot reliably estimate or track changes in arterial oxygen saturation in patients with acute respiratory distress syndrome

#### Marion Helias^1^, Lisa Raia^1^, Estelle Nicolas^1^, Lee S. Nguyen^1,3^, Paul Jaubert^1^, Sarah Benghanem^1,2^, Zakaria Ait Hamou^1,2^, Pierre Dupland^1,2^, Julien Charpentier^1^, Frédéric Pène^1,2^, Alain Cariou^1,2^, Jean-Paul Mira^1,2^, Jean-Daniel Chiche^1,2^, Mathieu Jozwiak^1,2^

##### ^1^Hôpital Cochin, Paris, France; ^2^Université de Paris, Paris, France; ^3^CMC Ambroise Paré, Neuilly Sur Seine, France

**Correspondence:** Mathieu JOZWIAK (mathieu.jozwiak@aphp.fr)

*Annals of Intensive Care* 2021, **11(Suppl 1):**FC-234

**Rationale:** Ventilatory management of patients with acute respiratory distress syndrome (ARDS) includes pulse oximetry (SpO_2_) measurement. However, the accuracy of SpO_2_ measurement in critically ill patients is still debated. We investigated in patients with ARDS monitored with next-generation pulse oximeter (i) the accuracy and precision of SpO_2_ measurement and the different factors associated with it; (ii) the ability of SpO_2_ measurement to track changes in arterial oxygen saturation (SaO_2_) induced by a positive end-expiratory pressure (PEEP) trial and (iii) the ability of SpO_2_ measurement to detect hypoxemia.

**Patients and methods/materials and methods:** We prospectively included 55 consecutive patients with ARDS ventilated in pressure regulated volume control mode, in whom the attending physician decided to perform a PEEP trial. Simultaneous SpO_2_ and SaO_2_ measurements were performed at PEEP +5 and +15 cmH2O. SpO_2_ and SaO_2_ measurements were compared with Bland–Altman method and the bias was calculated as the mean difference between SpO_2_ and SaO_2_ measurements. The concordance rate between PEEP-trial induced changes in SpO_2_ and SaO_2_ was assessed with four-quadrant analysis and the inter-rater agreement kappa coefficient (*κ*).

**Results:** All patients had pulmonary ARDS, 47% were patients with COVID-19 and 31% were black. Overall, SpO_2_ and SaO_2_ measurements were significantly correlated (*r* = 0.70; *p* < 0.0001). The bias was +1.1%, the precision 3.4% and the limits of agreement ranged from −5.6 to +7.9%. Ethnic group, COVID-19 status and PaO_2_/FiO_2_ ratio were significantly associated with bias, with a poorer accuracy of SpO_2_ measurement in black, COVID-19 and severe ARDS patients. Overall, PEEP trial-induced changes in SpO_2_ were correlated to changes in SaO_2_ (*r* = 0.65, *p* < 0.0001), changes in respiratory system compliance (*r* = 0.44, *p* = 0.0007), changes in PaO_2_/FiO_2_ ratio (*r* = 0.35, *p* = 0.009) but not to changes in functional residual capacity (*r* = 0.09, *p* = 0.14). The concordance rate between PEEP trial-induced changes in SpO_2_ and SaO_2_ was 84% and the κ coefficient was 0.60. Only COVID-19 status was independently associated with the concordance rate between PEEP trial-induced changes in SpO_2_ and SaO_2_. The concordance rate between PEEP trial-induced changes in SpO_2_ and SaO_2_ was 69% in COVID-19 patients and 97% in non-COVID-19 patients. Overall, a SpO_2_ ≤ 92%, ≤ 89% and ≤ 88% detected with 100% specificity a SaO_2_ ≤ 95%, ≤ 92% and ≤ 90%, respectively. A SpO_2_ ≤ 90% detected a SaO_2_ ≤ 90% with a sensitivity of 60 [95% confidence interval (CI):39–79]% and a specificity of 95 [95% CI: 88–99]%.

**Conclusion:** In patients with ARDS monitored with next-generation pulse oximeter, SpO_2_ measurement could neither reliably estimate SaO_2_, nor reliably track PEEP trial-induced changes in SaO_2_.

**Compliance with ethics regulations:** Yes in clinical research.

### FC-235 Post-intubation tracheal stenosis: diagnosis and treatment

#### Hamza Mimouni, Brahim Housni

##### CHU MOHAMMED VI OUJDA MAROC, Oujda, Maroc

**Correspondence:** Hamza Mimouni - hamzamimouni10@gmail.com

*Annals of Intensive Care* 2021, **11(Suppl 1):**FC-235

**Rationale:** Post-intubation tracheal stenosis (PITS) is a complication of ventilatory support by intubation and/or tracheotomy. Most often the diagnosis of PITS is easy in the presence of inspiratory high located dyspnea in a patient with a history of intubation or tracheotomy. Endoscopic and radiological investigations make it possible to study the characteristics of stenosis in order to adapt therapy. The treatment of choice remains surgical; it is based on tracheal resection-anastomosis which guarantees satisfactory and reliable results in the long term.

**Patients and methods/materials and methods:** We reviewed retrospectively the medical records of 9 patients admitted to our ICU with PITS during February 2018 to February 2020. Ten of them were males with mean age 52 years old and seven females with mean age 61. In relatively stable patients, computed tomography and virtual tracheoscopy were used, followed by rigid (RB) or fiberoptic bronchoscopy. In emergency cases we used RB for diagnosis and treatment. All procedures in the operating room were done under general anesthesia.

**Results:** In 7 patients PITS had a diameter of 5 to 6 mm and produced dyspnea. Four of 7 patients had soft PITS that were dilated with rigid dilators; in another three patients with hard stenosis, balloon dilation was used. Emergency tracheostomy was performed in one case and intubation with small endotracheal tube after partial dilation in one case. The average duration of intubation was 9 days, 2 patients received tracheotomy 10 days on average after intubation. Respiratory dyspnea in 8 (88%) patients was the main finding of tracheal stenosis, clinical examination found signs of respiratory distress in all patients. Endoscopy, performed under general anesthesia, showed a single tracheal stenosis in 7 patients, and the appearance was diaphragmatic in 55.5% of cases. The lesion averaged 32 mm from the glottal plane and the mean extent was 10 mm. The dilatation was performed in all patients resection tracheal anastomosis was performed in 5 patients (55%).

**Discussion:** Laryngo-tracheal endoscopy is the essential part of exploration, to appreciate their complexity and to check the laryngeal mobility, but the radiological assessment is also an essential step in the management of tracheal stenosis. Endoscopic treatment plays an important role in the treatment of tracheal stenosis. In fact, endoscopy allows the practice of instrumental dilations.

**Conclusion:** Post-intubation tracheal stenosis should be considered in the differential diagnosis of any patient who has recently been intubated in an intensive care unit and who presents with exertional dyspnoea or monophonic wheeze.

**Compliance with ethics regulations:** Yes in clinical research.

### FC-236 Protective ventilation practice: a survey at Sidi Bel Abbes University Hospital

#### Abdelwahab Allem, Yahia Abbou, Mokhtar Talbi, Mustapha Achi, Samir Bouadjaj, Reda Boumlik, Mourad Messad, Amel Kada, Wahiba Ghomari, Soumia Benbernou

##### centre hospitalier universitaire, Sidi Bel Abbes, Algerie

**Correspondence:** Abdelwahab Allem - allemabelwahab1989@gmail.com

*Annals of Intensive Care* 2021, **11(Suppl 1):**FC-236

**Rationale:** Protective ventilation is increasingly used in anesthesia and ICU, because many studies have shown its benefits on the lung and advocate for its generalization to all patients. It combines a reduction in the current volume and a positive end-of-expiration pressure (PEP). Few studies have been carried out in Algeria in this field. In the face of this revolution, we asked ourselves what was the practice of this concept in anesthesia and resuscitation at the CHU Sidi Bel Abbes (west of Algeria). Our objective was to evaluate the use of protective ventilation in the operating room and in ICU in our center.

**Patients and methods/materials and methods:** We wanted to know the state of the practice of protective ventilation at the University Hospital Center of Sidi Bel Abbes during the month of December 2020. To this end we realized an observation grid and an audit. Initially, a collection of data was developed on the different ventilatory parameters in each operating unit and in the two ICUs. Secondarily, a questionnaire was sent to medical and paramedical professionals to collect their knowledge on protective ventilation. The model was designed according to the model used in the VENTILOP survey.

**Results:** 70 health professionals were interviewed, including 10 senior anesthesiologists/intensivists, 40 residents in anesthesiology/intensive care and 20 medical auxiliaries in anesthesiology/intensive care. The participation rate was estimated at 71% which 50 professionals in total (10 anesthesiologists/intensivists, 30 residents in anesthesiology/intensive care and 10 auxiliaries in anesthesiology/intensive care). Most answering machines have control over the tidal volume setting. On the other hand, more than two-thirds do not control the PEP setting, for the rest of the parameters (FIO2, I/E setting, recruitment maneuvers) more than half need continuous training in order to control the setting of all the parameters.

**Conclusion:** Despite the proven interest of protective mechanical ventilation in anesthesia and ICU, its use is not widespread in all services at our center. Half of the staff interviewed did not integrate it into their daily practice. Strict protocols must be applied to ban bad habits in mechanical ventilation

**Reference**

1. Fischer F, et al. Annales Françaises d’Anesthésie et de Réanimation. 2014; 33: 389–94.

**Compliance with ethics regulations:** Yes in clinical research.

### FC-237 Cardiac arrest as a cause of acute respiratory distress syndrome: respiratory mechanics, gas exchange and ventilator settings

#### Jean-Christophe Richard^1^, Pierre-Yves Olivier^1,2^, Hamid Merdji^3^, Bertrand Pavlovsky^1^, Juliette Meunier^2^, Christophe Desprez^1^, Antoine Studer^3^, Maëva Campfort^1^, Arnaud Lesimple^1^, Nicolas Mahr^4^, Christophe Guitton^2^, Dominique Savary^1^, Alain Mercat^1^, Ferhat Meziani^3^, François Beloncle^1^

##### ^1^Medical ICU, University Hospital of Angers, Vent’Lab, University of Angers,, Angers, France; ^2^ICU, General Hospital of Le Mans, Le Mans, France; ^3^Medical ICU, University Hospital of Strasbourg, University of Strasbourg, INSERM, UMR 1260, RNM, FMTS,, Strasbourg, France; ^4^Medical ICU, University Hospital of Besançon, University of Franche-Comté, Besançon, Besançon, France

**Correspondence:** François Beloncle - francois.beloncle@univ-angers.fr

*Annals of Intensive Care* 2021, **11(Suppl 1):**FC-237

**Rationale:** Specific respiratory mechanics alterations of patients ventilated after a cardiac arrest have never been systemically assessed. This study aimed at assessing respiratory mechanics, gas exchange and ventilator settings of patients with or without ARDS after non-traumatic cardiac arrest (post CA-ARDS and post CA-non ARDS).

**Patients and methods/materials and methods:** Respiratory mechanics and gas exchange were prospectively assessed within 36 h after intubation in patients admitted in two ICUs after a non-traumatic cardiac arrest. Incidence of post CA-ARDS and ventilator settings were assessed in another three centers retrospective cohort.

**Results:** Among the 43 patients included in the prospective physiological study, respiratory system compliance and end-expiratory lung volume (EELV) at positive end-expiratory pressure (PEEP) 5 cmH2O were lower in post CA-ARDS than in post CA-non ARDS patients but chest wall compliance was not different. The dead space fraction was higher in post-CA ARDS than in post CA-non ARDS patients. EELV was lower in the 10 (23%) patients with airway closure. Forty-three percent of the 244 patients included in the retrospective cohort presented a post CA-ARDS. Distribution of tidal volume, PEEP and PaCO2 were similar in post-CA ARDS and post-CA non-ARDS patients. Among patients with post CA-ARDS, 19% have a tidal volume > 8 mL.kg^−1^ predicted body weight and 66% a PEEP set ≤ 5 cmH_2_O. Prone positioning concerned only 10% of severe post CA-ARDS.

**Conclusion:** Respiratory mechanics features of post CA-ARDS patients significantly differed from those of non-ARDS patients. Post CA-ARDS is frequent and specific lung protective ventilatory strategies were scarcely applied. These observations suggest that post CA-ARDS should be identified as a specific entity.

**Compliance with ethics regulations:** Yes in clinical research.

### FC-238 Lung transplant for late refractory ARDS (LR-ARDS): primary experience

#### Guillaume Tachon, Elise Cuquemelle, Benjamin Zuber, Jérome Devaquet, Matthieu Glorion, Julien De Wolf, Ciprian Pricopi, François Parquin, Antoine Roux, Clément Picard, Morgan Leguen, Edouard Sage, Charles Cerf, FOCH TRANSPLANT GROUP

##### Hôpital Foch, Suresnes, France

**Correspondence:** Charles Cerf - c.cerf@hopital-foch.com

*Annals of Intensive Care* 2021, **11(Suppl 1):**FC-238

**Rationale:** In some patients with late refractory ARDS (LR-ARDS) on maximal support by ECMO, considering switch from “ECMO to recovery” to bridge to lung transplantation (LTx) could be an option (1). We report our preliminary experience in such strategy.

**Patients and methods/materials and methods:** Between 2016 and 2020 patients with LR-ARDS on ECMO to recovery strategy failure referred to our center for LTx evaluation were reviewed. Patients with pre-existing lung disease or other severe comorbidities before ARDS onset were not considered for evaluation. In all cases, lung damage irreversibility was established by multidisciplinary staff. Results are expressed in median [min;max]. This study was approved by ethic local committee.

**Results:** 13 patients (4 women; 9 men) aged of 56 years [20;63] were included. The etiologies were SARS-CoV-2 ARDS (9), toxic (2), anti-MDA5 interstitial pneumonia (1), and unknown (1). Only 6 patients were transferred to our center. Only those data were analyzed. Among 6 patients, 4 were transplanted and 2 died before LTx. ECMO duration, mecchanical ventilation (MV) duration and ICU stay before LTx were, respectively, 35 days [20;88], 38 [20;89], 34 [3;94], respectively. All patients had sarcopenia and severe ICU acquired paresis with MRC < 12. All patients were tracheotomized and none had bedsores. There was no difference between LTx and non-LTx patients. Contra-indications for LTx were severe extra-respiratory failure (1/6) and non-controlled infection (1/6). All transplant patients are alive with a follow-up of 445 days [95;1790]. Post-operative MV duration was 24 days [9;126]; length of stay in ICU was 59 days [29;202]. Every induction protocol included basiliximab and post-operative immunosuppression was as usual. 2 patients were informed of and accepted LTx surgery and 2 could not. Though, patients graft acceptance and treatment adhesion were good in all cases. Relatives were systematically extensively informed of risks and benefits, and accepted the strategy. All patients were listed in high emergency program after expert panel acceptation.

**Conclusion:** In our experience, LTx seems to be a reasonable option in irreversible LR-ARDS under maximal support therapy in very selected patients in an expert center with a specific task force. Incidence of such young potentially eligible patient is increased due to the current COVID-19 pandemic. As further evaluations are needed, we propose to create a national registry to evaluate this new strategy.

**Reference**

1. Robert B et al. Lung-Transplantation for Severe post-Coronavirus Disease 2019 Respiratory Failure. Transplantation; 2021.

**Compliance with ethics regulations:** Yes in clinical research.

### FC-239 Tracheotomy in intensive care unit: evaluation of practices

#### Dorra Sakis, Ines Fathallah, Nadia Kouraichi

##### HOPITAL REGIONAL BEN AROUS TUNIS, Ezzahra, TUNISIA

**Correspondence:** Dorra Sakis - dorra.sakis@gmail.com

*Annals of Intensive Care* 2021, **11(Suppl 1):**FC-239

**Rationale:** In spite being a common practice in intensive care unit (ICU), tracheotomy still a procedure that faces disparity. The divergence of views on its utility, indications, the appropriate timing, and its technique led us to conduct this study. The main aim of our study was to evaluate our practices.

**Patients and methods/materials and methods:** It was a descriptive analysis of a questionnaire with 34 items, sent via Google Forms platform evaluating tracheotomy practice in Tunisian intense care unit. We have referred to the latest recommendation of the scientific society (Société de Réanimation de Langue Française SRLF and Société Française d’Anesthésie et de Réanimation SFAR) [1].

**Results:** Fifty-five intensivists had answered to the questionnaire, 47 (85%) were practicing in university-affiliated hospitals. The participation rate was 30%. Thirty-nine (17%) was the number of medical intensivists. An informed consent signed by the legal guardian before each tracheotomy was required by 44% of the intensivists. The percutaneous tracheotomy was performed by 21 (38%) of those who answered. Twelve intensivists had already a standardized procedure of percutaneous tracheotomy in their department. Twenty-two disposed of care protocols defining the management of the tracheotomy, its supervision and its maintenance. Thirteen of the clinicians preferred the percutaneous tracheotomy by unique progressive dilatation. Forty-six consider deflating the balloon once the patient was spontaneous ventilated. The pressure of the balloon was measured daily by 35 (63%) of teams and every 8 h by 17 (31%) of them. During the stay in the ICU the change of the cannula was guided by clinical observation (*n* = 34) or systematically every 5 days (*n* = 15). Thirteen practitioners disposed of multidisciplinary protocol of decannulation. Among the Tunisian intensivists that answered to our questionnaire, 53% were compliant to the recommendation.

**Conclusion:** Tracheotomy practice is heterogeneous in Tunisian ICU and only 53% were compliant to the international recommendation.

**Reference**

1. Trouillet JL, Collange O, Belafia F, Blot F, Capellier G, Cesareo E, et al. Tracheotomy in the intensive care unit: guidelines from a french expert panel. Ann Intensive Care. 2018;8(1):37.

**Compliance with ethics regulations:** Yes in clinical research

### FC-240 Tracheostomy management in patients with severe acute respiratory distress syndrome receiving extracorporeal membrane oxygenation: an international multicenter retrospective study

#### Thomas Frapard^1^, Tài Pham^7,9^, Christoph Fisser^2^, Martucci Martucci^3^, Darry Abrams^4,5^, Konstantin Popugaev^6^, Antonio Arcadipane^3^, Cara Agerstrand^4,5^, Alexis Serra^5^, Sacha Rozencwajg^1^, Sergey Petrikov^6^, Thomas Mueller^2^, Daniel Brodie^4,5^, Alain Combes^1,8^, Matthieu Schmidt^1,8^

##### ^1^Assistance Publique–Hôpitaux de Paris, Pitié–Salpêtrière Hospital, Medical Intensive Care Unit, Paris, France; ^2^Department of Internal Medicine II, University Hospital Regensburg, Regensburg, Germany; ^3^Instituto Mediterraneo per i Trapianti e terapie ad alta specializzazione - Department of Anesthesia and Intensive Care, Palermo, Itlay; ^4^Department of Medicine, Columbia University College of Physicians & Surgeons, New York, USA; ^5^Center for Acute Respiratory Failure, New York-Presbyterian Hospital, New York, USA; ^6^Sklifosovsky Research institute of Emergency Medicine, Bolshaya Sukharevskaya squire, 3, Moscow, Federation De Russie, RUSSIA; ^7^Université Paris-Saclay, AP-HP, Service de médecine intensive-réanimation, Hôpital de Bicêtre, DMU CORREVE, FHU SEPSIS, Groupe de recherche clinique CARMAS, Le Kremlin-Bicêtre, France; ^8^Sorbonne Université, Paris 06, INSERM UMRS_1166-iCAN, Institute of Cardiometabolism and Nutrition, Paris, France; ^9^Université Paris-Saclay, UVSQ, Univ. Paris-Sud, Inserm, Equipe d’Epidémiologie respiratoire intégrative, CESP, Villejuif, France

**Correspondence:** Thomas Frapard - t.frapard@gmail.com

*Annals of Intensive Care* 2021, **11(Suppl 1):**FC-240

**Rationale:** Current practices regarding tracheostomy in patients treated with extracorporeal membrane oxygenation (ECMO) for acute respiratory distress syndrome are unknown. We aim to assess the prevalence and the impact of the timing of the tracheostomy (i.e. during or after ECMO decannulation) on related complications, sedative, and analgesic use.

**Patients and methods/materials and methods**: International, multicenter, retrospective study in four large volume ECMO centers during a 9-year period.

**Results:** Of the 1,168 patients treated with ECMO for severe ARDS (age 48 ± 16 years, 76% male, SAPS II score 51 ± 18) during the enrollment period, 353 (30%) and 177 (15%) were tracheostomized during ECMO or after ECMO decannulation, respectively. The incidence and the severity of early tracheostomy complications were uncommon and similar for the two groups, whereas local bleeding or oozing after 24 h was four times more frequent when the tracheostomy was performed during ECMO (25 vs 7%, *p* < 0.01). Cumulative sedative consumption decreased more rapidly after the procedure for patients tracheostomized after ECMO decannulation with sedative doses almost negligible 48–72 h after the tracheostomy when performed after ECMO decannulation (*p* < 0.01). A significant increase of the RASS was observed within 72 h after the tracheostomy in the “after ECMO” group whereas it was unchanged in the “during-ECMO” group.

**Conclusion:** Contrary to the patients undertaking tracheostomy after ECMO withdrawal, tracheostomy during ECMO was not associated with a prompt decrease of the sedation and analgesic levels and an increase of the RASS. This finding and a higher local bleeding risk during the days following the procedure reinforce the need for a case-by-case discussion of the balance between risks and benefits when performed during ECMO.

**Compliance with ethics regulations:** Yes in clinical research.

### FC-241 Post-extubation non-invasive ventilation: application, modalities and predictive failure factors

#### Amira Jamoussi, Lynda Messaoud, Lilya Debbiche, Samia Ayed, Fatma Jarraya, Emna Rachdi, Jalila Ben Khelil, Mohamed Besbes

##### CHU Abderrahmen Mami, Tunis, TUNISIA

**Correspondence:** Amira Jamoussi - dr.amira.jamoussi@gmail.com

*Annals of Intensive Care* 2021, **11(Suppl 1):**FC-241

**Rationale:** Non-invasive ventilation (NIV) is a supportive therapy that may improve mortality in acute respiratory failure. Currently, it belongs to the therapeutic arsenal used during weaning of patients from mechanical ventilation. Nevertheless, NIV application modalities in post-extubation are not accurately codified. The aim of this study was to describe post-extubation NIV use in intensive care unit clinical practice and to identify factors associated with failure.

**Patients and methods/materials and methods:** Retrospective study carried out in respiratory medical intensive care unit of Abderrahmen Mami teaching hospital between January 2017 and December 2018. It included patients who required more than 48 h of invasive mechanical ventilation and who received NIV after extubation. NIV application modalities were recorded and predictive NIV failure (death under NIV or re-intubation within 72 h after extubation) factors were identified.

**Results:** During study period, 60 patients were included with mean age of 56 ± 18 years, sex ratio of 3.6 and COPD history in 43% patients. Extubation was scheduled in 48 patients (80%) and unplanned in 12 (20%). Post-extubation NIV was: preventive (*n* = 36; 60%), therapeutical (*n* = 5; 8%) and systematic (*n* = 19; 32%). Pressure support was the most frequently adopted mode (93%) with a mean inspiratory pressure support level of 12.9 ± 2.9 cmH2O and the mean positive expiratory pressure of 6.2 ± 1.7 cmH2O. NIV was started meanly 3.6 h [1–30] after extubation and discontinuously applied in 70% of cases with a mean rhythm of one session every 4 h. The mean whole duration of NIV was 2.87 days [1–13]. NIV rate failure was 20%, mean length of stay was 18 days and overall mortality was of 25%. Multivariate analysis identified two independent predictors of NIV failure: Age > 69 years (OR = 6.13, 95% CI [1.03–36.5], *p* = 0.046) and duration of NIV at day 1 > 9 h (OR = 10.18, 95% CI [1.65–62.59], *p* = 0.012).

**Conclusion:** Non-invasive ventilation is an effective technique to avoid re-intubation. Age over 69 years and need to NIV for more than 9 h during the first day are significantly associated to NIV failure after extubation.

**Compliance with ethics regulations:** Yes in clinical research.

### FC-242 Non-invasive ventilation vs. high-flow nasal oxygen after extubation of obese patients in ICU: a post hoc analysis of a randomised controlled trial

#### Arnaud Thille^1^, Rémi Coudroy^1^, Mai-Anh Nay^2^, Arnaud Gacouin^3^, Maxens Decavèle^4^, Romain Sonneville^5^, François Beloncle^6^, Chistophe Girault^7^, Laurence Dangers^8^, Alexandre Lautrette^9^, Quentin Levrat^10^, Anahita Rouzé^11^, Emmanuel Vivier^12^, Jean-Baptiste Lascarrou^13^, Jean-Damien Ricard^14^, Keyvan Razazi^15^, Guillaume Barberet^16^, Christine Lebert^17^, Stephan Ehrmann^18^, Alexandre Massri^19^, Jeremy Bourenne^20^, Gael Pradel^21^, Pierre Bailly^22^, Nicolas Terzi^23^, Jean Dellamonica^24^, Guillaume Lacave^25^, René Robert^1^, Stéphanie Rgot^1^, Jean-Pierre Frat^1^

##### ^1^CHU de Poitiers, Poitiers, France; ^2^CH d’Orléans, Orléans, France; ^3^CHU de Rennes, Rennes, France; ^4^Hôpital de la Pitié Salpétrière, Paris, France; ^5^Hôpital Bichat, Paris, France; ^6^CHU d’Angers, Angers, France; ^7^CHU de Rouen, Rouen, France; ^8^CHU Félix Guyon, Saint Denis De La Réunion, France; ^9^CHU de Clermont-Ferrand, Clermont-Ferrand, France; ^10^CH de La Rochelle, La Rochelle, France; ^11^CHU de Lille, Lille, France; ^12^Hôpital Saint Joseph Saint Luc, Lyon, France; ^13^CHU de Nantes, Nantes, France; ^14^Hôpital Louis Mourier, Colombes, France; ^15^Hôpital Henri Mondor, Créteil, France; ^16^CHR Mulhouse Sud Alsace, Mulhouse, France; ^17^CH Départemental de Vendée, La Roche Sur Yon, France; ^18^CHRU de Tours, Tours, France; ^19^CH de Pau, Pau, France; ^20^CHU de Marseille La Timone 2, Marseille, France; ^21^CH Henri Mondor d’Aurillac, Aurillac, France; ^22^CHU de Brest, Brest, France; ^23^CHU de Grenobles Alpes, Grenoble, France; ^24^CHU de Nice, Nice, France; ^25^CH de Versailles, Le Chesnay, France

**Correspondence:** Arnaud Thille - aw.thille@gmail.com

*Annals of Intensive Care* 2021, **11(Suppl 1):**FC-242

**Rationale:** In intensive care units (ICUs), the use of non-invasive ventilation (NIV) immediately after extubation may prevent reintubation in patients at high-risk of extubation failure. Prophylactic use of NIV after extubation has been poorly assessed in obese patients. We hypothesized that NIV may be particularly effective in obese patients by decreasing the risk of reintubation compared with high-flow nasal oxygen.

**Patients and methods/materials and methods:** Post hoc subgroup analysis focusing on obese patients included in a multicenter, randomised, controlled trial comparing NIV alternating with high-flow nasal oxygen versus high-flow nasal oxygen alone within the first 48 h after extubation. The main outcome was reintubation rates within the 7 days following extubation according to oxygenation strategy.

**Results:** Among 628 patients extubated in 30 ICUs, 206 (33%) had obesity including 112 patients (54%) treated with NIV and 94 (46%) treated with high-flow nasal oxygen alone. The reintubation rate at day 7 was 6% (7 of 112 patients) with NIV and 18% (17 of 94 patients) with high-flow nasal oxygen alone (difference − 12%; 95% CI, − 21 to − 3; *p* = 0.008). Whereas the reintubation rate was exactly the same in non-obese patients treated with high-flow nasal oxygen (18%, 37 of 201 patients; difference 0.3% compared with obese patients; 95% CI, − 10% to 9%; *p* = 0.92 using log-rank test), the reintubation rate in patients treated with NIV was significantly lower in obese (6%, 7 of 112 patients) than in non-obese patients (15%, 33 of 221 patients, difference, − 9%; 95% CI, − 15 to − 1; *p* = 0.02 using log-rank test).

**Conclusion:** Prophylactic NIV alternating with high-flow nasal oxygen immediately after extubation of obese patients significantly decreased the rate of reintubation as compared with high-flow nasal oxygen alone. NIV was particularly effective in obese patients with reintubation rates significantly lower than in non-obese patients treated with NIV.

**Compliance with ethics regulations:** Yes in clinical research.

### FC-243 High-flow oxygen during spontaneous breathing trial for patients at high risk for weaning failure: a pilot randomized controlled trial

#### Guillaume Fossat^1^, Mai-Anh Nay^1^, Sophie Jacquier^2^, Dalila Benzekri^1^, Armelle Mathonnet^1^, Anne Bretagnol^1^, Marie Skarzynski^1^, Gregoire Muller^1^, Toufik Kamel^1^, Francois Barbier^1^, Nicolas Bercault^1^, Isabelle Runge^1^, Emmanuelle Desmalles^1^, Thierry Boulain^1^

##### ^1^CHR ORLEANS, Orleans, France; ^2^chu tours, Tours, France

**Correspondence:** Guillaume Fossat - guillaume.fossat@chr-orleans.fr

*Annals of Intensive Care* 2021, **11(Suppl 1):**FC-243

**Rationale:** T-piece spontaneous breathing trial (SBT) is commonly performed as the final step of mechanical ventilation (MV) weaning to assess whether a patient is eligible for extubation. However, T-piece SBT appears as a highly demanding ventilation strategy that may lead to delayed weaning and extubation failures when compared to Pressure Support SBT. High-flow oxygen (HFO) therapy could be usefully implemented during SBT, as in hypoxemic respiratory failure it reduces the work of breathing, maintains a low level of positive end-expiratory pressure, exerts a wash-out effect on the anatomical dead-space, and has no hemodynamic side-effect. This study aimed to investigate whether HFO SBT would accelerate MV weaning while not increasing the rate of re-intubation when compared to standard T-piece SBT in patients at risk for extubation failure.

**Patients and methods/materials and methods:** In this single-ICU pilot randomized controlled trial, patients under MV for > 24 h who had chronic pulmonary and/or cardiac disease and in whom an SBT was ordered, were randomly allocated (1:1) to T-piece SBT or HFO SBT using a connecting device attached to the endotracheal tube. Primary outcome measures were (1) the rate of extubation within 7 days after the first SBT and (2) the rate of re-intubation within 7 days after extubation. Secondary outcome measures were (1) the rate of trial success and (2) arterial partial pressure of oxygen (PaO2) at the end of first SBT.

**Results:** 106 patients (mean age 69.4 [10.4] years, male sex 73.6%, chronic pulmonary condition 59,4%, chronic cardiac condition 68.9%, median duration of MV 5 [3–9] days) were randomized to T-piece or HFO SBT (53 per group). Rates of extubation within 7 days following the first SBT were 94.3% (50/53) in the T-piece group and 96.2% (51/53) in the HFO group. The rates of reintubation within 7 days after extubation were 16% (8/50) in T-piece group and 3.9% (2/51) in HFO group (*p* = 0.051 Fisher exact test). Success rates of first SBT were not different between groups (73.6% [39/53] in the T-piece and 86.8% [46/53] in the HFO; *p* = 0.14). PaO2 values at the end of first SBT were higher with HFO than with T-piece (76 [69–97] mmHg versus 69 [69–97] mmHg; *p* = 0.003].

**Conclusion:** In patients at-risk for extubation failure, HFO SBT neither hastened MV weaning nor increased the rate of re-intubation compared to T-piece SBT. This pilot study may have been underpowered to assess these endpoints, HFO was associated with improved arterial oxygenation at the time of SBT completion.

**Compliance with ethics regulations:** Yes in clinical research.

### FC-244 Ventilatory weaning failure of cardiac origin according to the variation of hemoglobin and protidemia

#### Walid Sellami, Ines Ben Mrad, Iheb Labbene, Mustapha Ferjani

##### Department of critical care medicine and anesthesiology, Military Hospital of Tunis, Tunisia

**Correspondence:** Walid Sellami - drsellamiwalid@yahoo.fr

*Annals of Intensive Care* 2021, **11(Suppl 1):**FC-244

**Rationale:** Weaning failure of cardiac origin can be diagnosed by the elevation of the left ventricular (LV) filling pressure. This hydrostatic pulmonary edema is associated with hemoconcentration du to hypooncotic fluid movement from the vascular compartment to the interstitium. The aim of this study was to search for a correlation between the protidemia and the hemoglobin elevation during the weaning test and the presence of LV filling pressure elevation.

**Patients and methods/materials and methods:** This prospective observational study was conducted during 24 months (between January 2018 and January 2020). Every patient with weaning failure was included. The variation of the biological and ultrasound criteria between the periods before and after the ventilator weaning was analysed.

**Results:** We included 60 patients which failed during the first weaning test. 20 patients (33%) had an elevation of the LV filling pressure during the second weaning test with E/A > 0.95 and E/Ea > 8.5 at the end of the test period and within these 20 patients, 12 (60%) failed this second weaning test. When compared to the 40 patients who didn’t present a pulmonary edema, these 12 patients required additional delay for the weaning: 2.5 + 3.7 days versus 0.75 + 2.4 days (*P* = 0.023). the unique predictive factor associated with the occurrence of pulmonary edema was positive body weight difference between the admission in the ICU and the inclusion in the study (4.6 + 5.6 versus 3.5 + 7.3, *p* = 0.004). The was no significant variation of the hemoglobin and protidemia during the weaning for the patients who presented a pulmonary edema.

**Conclusion:** During this study we did not find a correlation between the hemoglobin or the protidemia variation during the weaning period and the ultrasound criteria of th LV filling pressure elevation. The difference of body weight between the admission in the ICU and the inclusion, which reflect a positive fluid balance, was the unique factor associated with weaning failure of cardiac origin. Its control could allow a more frequent weaning success and the diminution of the morbidity and mortality due to the diminution of the mechanical ventilation duration

**Compliance with ethics regulations:** Yes in clinical research.

### FC-245 Prolonged invasive mechanical ventilation risk factors

#### Ameni Abidi^1^, Foued Daly^2^

##### ^1^Service de urgences, Centre d’Assistance Médicale Urgente et de Réanimation, Tunis, Tunisie; ^2^Service de réanimation, CHU la Rabta, Tunis, Tunisia

**Correspondence:** Ameni Abidi - drabidiameni@gmail.com

*Annals of Intensive Care* 2021, **11(Suppl 1):**FC-245

**Rationale:** Risk factors for a prolonged invasive mechanical ventilation greatly vary from one study to another. The aim of our study was to determine the risk factors for prolonged mechanical ventilation in an intensive care unit.

**Patients and methods/materials and methods:** It was a retrospective, monocentric and descriptive study, conducted during the year 2018, from January to November in the intensive care unit of La Rabta University Hospital. Patients over 15 years of age, who required invasive mechanical ventilation for more than 48 h with a length of stay of at least 7 days were included. Prolonged mechanical ventilation was defined as 7 days of mechanical ventilation.

**Results:** Fifty-eight patients were included during the study period. The average age was 44 (± 15.4) and the gender ratio was 1.3. The most frequent reason for admission was coma in 35 cases (60). The mean IGS II and APACHE II scores at admission were 33 (± 15.6) and 14 (± 6.6), respectively. The median duration of invasive mechanical ventilation was 10 days (3.75–14.75). The most frequent complications during the stay were shock (57%) and ventilator-associated pneumonia (52%). Mortality rate was of 47%. Fifteen patients (26%) had a prolonged mechanical ventilation. Risk factors for prolonged invasive mechanical ventilation identified by multivariate logistic regression analysis were the IGS II score on admission superior to 33 (*p* = 0.016; ORa = 15.08 et IC95% = 1.76–149.36), P/F ratio under 278 mmHg on admission (*p* = 0.015), CRP level on admission superior to 56 mg/L (*p* = 0.050; ORa = 7.11 et IC95% = 1.01–50.32) on admission and complications during stay as acute respiratory distress syndrome (ARDS) (*p* = 0.031; ORa = 16.52 et IC95% = 1.29–210.85) and ventilator-associated pneumonia (VAP) (*p* = 0.013; ORa = 14.08 et IC95% = 1.76–112.43).

**Conclusion:** Prolonged mechanical ventilation is frequent in intensive care units (26%). Identified risk factors are a high score of severity on admission, hypoxemia, high biological markers level and complications during stay as ARDS and VAP.

**Compliance with ethics regulations:** Yes in clinical research

### FC-246 Clinical phenotyping by machine learning in critically ill coronavirus-2019 infected patients

#### Mathieu Carpentier^1^, Yoann Zerbib^1^, Jack Richecoeur^2^, Olivier Bonef^3^, Clément Brault^1^, Michel Slama^1^, Julien Maizel^1^

##### ^1^CHU Amiens, Amiens, France; ^2^CH Beauvais, Beauvais, France; ^3^CH Saint-Quentin, Saint-Quentin, France

**Correspondence:** Mathieu Carpentier - mathieu.carpentier@chu-angers.fr

*Annals of Intensive Care* 2021, **11(Suppl 1):**FC-246

**Rationale:** As the vaccination campaign has begun, we are still trying to determine best which patients benefit the most of an admission in intensive care and bring them the most adapted care. The goal of our study was to use an unsupervised clustering approach to determine clinical phenotypes among critically ill COVID-19 patients.

**Patients and methods/materials and methods:** A retrospective observational multicentric cohort study from February 2020 to May 2021, taking place in Intensive Care Unit of several hospitals in the northern part of France, was conducted. Patients with proven SARS-CoV-2 infection diagnosed by PCR on fluid samples, meeting intensive care admission eligibility criteria, were included. Any patient who expressed disagreement in participating in the study was not included. The primary objective was to emphasize differences between clusters extracted by Machine Learning on a COVID-19 population. In a second time, we sought correlation between the patients’ evolution, needs in terms of treatment and cluster appurtenances. At last, we wanted to discuss the relevance of these clusters by linking them to the pathophysiology of COVID-19.

**Results:** Promising preliminary results were obtained with 44 patients from the Intensive Care Medicine Unit of Amiens University Hospital. Three clusters were obtained after hierarchical clustering on principal components (HCPC) (Fig. 1), labelled inflammatory, mild respiratory and severe respiratory. Outcomes were different according to cluster appurtenance in terms of respiratory failure management, duration of stay and need of extracorporeal renal replacement (*p* < 0.05). There were no difference in terms of mortality (*p* = 0.74). These are preliminary results; inclusions are ongoing and we plan to include approximatively 200 patients.

**Discussion:** As in former studies on subjects as sepsis shock [1], machine learning helped us to confirm the intuition that different presentations of COVID-1 9exist by using a innovative tool. On the other hand, we did not observe a difference in mortality and no specific parameter was isolated as a strong marker of cluster appurtenance

**Conclusion:** Using HCPC on our severe COVID-19 population allowed us to extract three clusters with independent phenotypes (inflammatory, severe respiratory, mild respiratory). More inclusions are needed to enhance our model, as inclusions are pursued.

**Reference**

1. Miailhe AF, LEPTOREA. Severe leptospirosis in non-tropical areas: a nationwide, multicentre, retrospective study in French ICUs. Intensive Care Med. 2019;45(12):1763–73. 10.1007/s00134-019-05808-6. Epub 2019 Oct 25. PMID: 31654079.

**Compliance with ethics regulations:** Yes in clinical research.
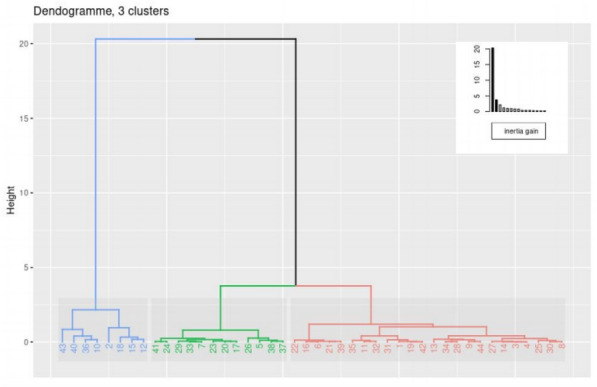


Fig. 1 Hierarchical tree: three clusters obtained

### FC-247 Influence of underlying lung disease on the progression and outcomes of COVID-19 in critically patients

#### Ahlem Trifi^1^, Emna Abid^1^, Rym Karaborni^2^, Bedis Jeribi^2^, Bedis Tlili^1^, Issam Saddem^2^, Asma Ouhibi^1^, Oussama Benjima^2^, Yosr Touil^1^, Foued Daly^1^, Sami Abdellatif^1^, Adel Ammous^2^, Salah Ben Lakhal^1^

##### ^1^Medical ICU, la Rabta hopital, Faculty of Medicine of Tunis, Tunis, Tunisie; ^2^Surgical ICU, la Rabta hopital, Faculty of Medicine of Tunis, Tunis, TUNISIA

**Correspondence:** Ahlem Trifi - trifiahlem2@gmail.com

*Annals of Intensive Care* 2021, **11(Suppl 1):**FC-247

**Rationale:** Despite the improvement in the clinical experience with COVID-19, little is known about COVID-19 in patients with underlying lung disease (ULD) and was it really associated with a poor outcome. We aimed to evaluate the influence of ULD on both clinical and para-clinical presentation and outcomes of COVID-19.

**Patients and methods/materials and methods:** Retrospective comparative cohort study including all patients hospitalized in ICU for COVID-19. Two groups were studied according to the presence or not of ULD. We collected data of demographics, clinical presentation, laboratory results, cardiac ultrasound and CT scan findings, treatments and outcomes. The primary outcomes were the use of mechanical ventilation (MV), ICU length of stay (LOS) and death.

**Results:** 143 patients were included of which 102 were males (ULD group, *n* = 31 versus no ULD group, *n* = 112). ULD corresponded to sleep apnea (SA, *n* = 8), chronic obstructive pulmonary disease (COPD, *n* = 7), asthma (*n* = 6), diffuse interstitial lung disease (ILD, *n* = 6) and mixed disorder in 4 cases (asthma/SA, *n* = 2 and COPD/SA, *n* = 2). Home oxygen or ventilation was required in 3 cases. The ULD group was more likely exposed to tobacco (45% vs 15%, *p* = 0.001) and have more history of bacterial pneumonia (22.5% vs 3%, *p* = 0.001). No difference was showed in the clinical presentation (cough, dyspnea, struggle signs, respiratory rate, digestive signs, myalgia, etc.). Severity scores and CURB-65 were similar. For laboratory results, only LDH (552 vs 402 IU/L, *p* = 0.035) and AST (60 vs 38 IU/L, *p* = 0.016) differed and it were higher in no ULD group. A CT scan involvement greater than 50% was more observed in no ULD group (47% vs 15%, *p* = 0.011). The follow-up of blood gas, compliance and driving pressure did not reveal any differences between the 2 groups. The 2 groups developed similarly ARDS, thrombo-embolic events and septic shock. Requirement of MV not differed (51% vs 44%, *p* = 0.41) as well as the duration of MV (6,6 vs 6,5 days, *p* = 0.9), the ICU-LOS (9.1 vs 10, *p* = 0.4) and the death rate (51.6% vs 55%, *p* = 0.68).

**Conclusion:** Unexpectedly, we did not find any major difference (regarding the clinical presentation, severity scores at admission and CURB-65, and the entire disease’s course: ARDS, MV requirement and mortality) between critical COVID-19 patients with ULD versus without ULD. The differences were showed with LDH and AST levels and CT scan lesions which were most marked with patients without ULD.

**Compliance with ethics regulations:** Yes in clinical research

### FC-248 Accuracy of laboratory indices as earlier indicator for stage-3 ARDS in COVID-19 patients

#### Ahlem Trifi^1^, Amal Mefteh^1^, Hichem Cherif^2^, Asma Mehdi^1^, Chokri Omri^2^, Karim Mabrouk^2^, Cyrine Abdennebi^1^, Foued Daly^1^, Yosr Touil^1^, Sami Abdellatif^1^, Adel Ammous^2^, Salah Ben Lakhal^1^

##### ^1^Medical ICU, la Rabta hospital, Faculty of Medicine of Tunis, Tunis, Tunisie; ^2^Surgical ICU, la Rabta hospital, Faculty of Medicine of Tunis, Tunis, TUNISIA

**Correspondence:** Ahlem Trifi - trifiahlem2@gmail.com

*Annals of Intensive Care* 2021, **11(Suppl 1):**FC-248

**Rationale:** COVID-19 is associated with a variety of complications. Rapid deterioration to acute respiratory distress syndrome (ARDS) occurs in about 10–15% of patients who develop dyspnea. Early identification of indicators for ARDS facilitated the access to the ICU. The improvement of acknowledgment in the field of COVID-19 reported that in addition to the metabolic co-morbidities; some biological indices occurred frequently in severe COVID-19 patients. We aimed to assess the utility of the most well-known disturbed laboratory parameters in COVID-19 to predict progression to stage 3-ARDS requiring mechanical ventilation (MV).

**Patients and methods/materials and methods:** We performed an analytical retrospective cohort study. Primary outcome: progression to the stage 3 (or severe) ARDS. After determining the cut-offs using the ROC curves and Youden’s indices, we selected the followings indices: neutrophil-to-lymphocyte ratio (NLR), D-dimers, C-reactive protein (CRP), fibrinogen, ferritinemia, prothrombin time (PT) and LDH.

**Results:** 143 patients were enrolled with a median age = 64 years [57–71], sex ratio = 2.48 (102/41), median Body mass index (BMI) = 27 kg/m^2^ [24–31] and SOFA = 4 [3–8]. Among them, 103 (72%) developed ARDS, within a median delay of 6 days [1–10], including 76/103 (74%) categorized as stage 3 (or severe) ARDS requiring MV. When each parameter analyzed apart, none of the above indices had a significant area under the curve (AUC): the best value was found with ferritinemia followed by LDH and fibrinogen (attached table). An additional analysis was then performed by combining the previous seven indices. 46 patients have, at the same time, all the abnormalities of the tested variables. Among them 34/46 (74%) developed severe ARDS requiring MV while 42 patients presented severe ARDS among 96 not having combined indices (44%) with an OR = 2.15 [1.28–3.61], *p* = 0.001. When all indices combined, that predict the progress to a stage-3 ARDS with AUC/ROC = 0.633 [0.541–0.724], *p* = 0.006 and a specificity at 85%.

**Conclusion:** We can be guided earlier by the laboratory results to predict the progression to stage 3 ARDS. The accuracy of predicting progression to stage 3 ARDS seems much better if we combine the seven indices analyzed above with good specificity. Establishing a score composed of these items will be of major practical interest since it may detect patients potentially requiring ICU and MV.

**Compliance with ethics regulations:** Yes in clinical research.
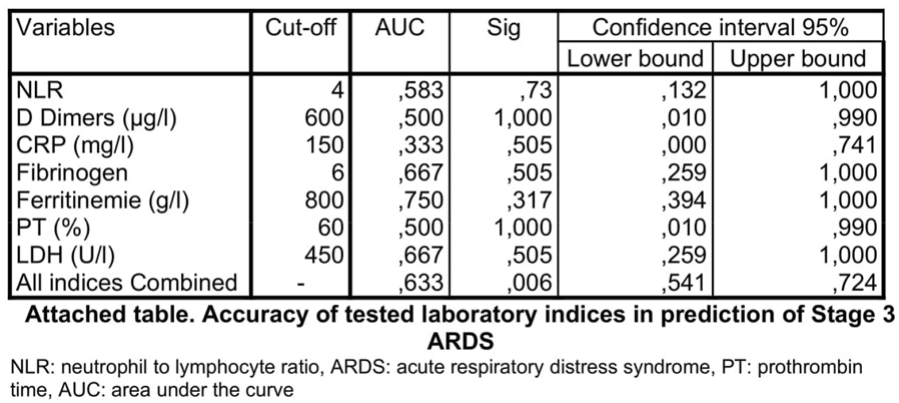


Attached table. Accuracy of tested laboratory indices in prediction of Stage 3 ARDS

### FC-249 Chest CT better than neutrophil-to-lymphocyte ratio for early discrimination of critical forms of COVID-19 among oxygen requiring patients

#### Martin Mombrun^1^, Richard Descamps^2^, Michel Ramakers^3^, Frédéric Godde^4^, Emmanuel Piednoir^4^, Damien Du Cheyron^2^

##### ^1^CH Lisieux, Lisieux, France; ^2^CHU Côte de Nacre, Caen, France; ^3^CH Saint-Lô, Saint-Lô, France; ^4^CH Avranches-Granville, Avranches, France

**Correspondence:** Martin Mombrun - mombrun.martin@gmail.com

*Annals of Intensive Care* 2021, **11(Suppl 1):**FC-249

**Rationale:** While the COVID-19 pandemic is still ongoing, there is a need to identify risk factors for the need for unconventional oxygen therapy in the context of limited medical resources. Neutrophil-to-lymphocyte ratio (NLR) has been identified as a marker correlated with COVID-19 severity and outcome.

**Patients and methods/materials and methods:** In a retrospective multicenter cohort study, we analyzed medical charts of patients with a severe pulmonary form of COVID-19 documented by real-time reverse transcriptase-polymerase chain reaction (RT-PCR) in 5 different hospitals. We used a multivariate model including clinical, biological variables including the NLR and chest CT variables to assess the risk factors for developing a critical form of COVID-19, in-hospital mortality, and ICU mortality. Our primary judgement criterion was the association between the NLR and evolution to a critical form of COVID-19.

**Results:** Of the 149 cases identified, 51% developed a critical form, 36.2% were admitted to ICU, 32.2% required mechanical ventilation, and 22.8% died. NLR was associated to evolution to a critical form of COVID-19 in univariate analysis but not in multivariate analysis. The risk factors independently associated with critical COVID-19 were a SOFA score at day 1 greater than 2, chest CT lesions exceeding 50% of lung area, the presence of pulmonary embolism, and the absence of crazy-paving of chest CT was a protective factor (respective ORs: 18.7, 9.1, 9.4 and 0.33). The risk factors independently associated with mortality were a Charlson comorbidity index greater than 4, CT scan damage greater than 50%, NLR at day 1 greater than 7, and diabetes mellitus (respective ORs: 10.3, 4.9, 2.7, 2.8). A critical form risk score of COVID-19 was obtained from the multivariate model, which showed an AUC of 0.843 on the ROC curve.

**Conclusion:** In our multicenter cohort study, the NLR was not the best tool to identify evolution to a critical from of COVID-19. Other risk factors identified by early chest CT were better to grade risk of progression to a critical form of COVID-19.

**References**

1. Ruch Y, Kaeuffer C, Ohana M, Labani A, Fabacher T, Bilbault P, et al. CT lung lesions as predictors of early death or ICU admission in COVID-19 patients. Clin Microbiol Infect. 2020.
2. Qin C, Zhou L, Hu Z, Zhang S, Yang S, Tao Y, et al. Dysregulation of immune response in patients with COVID-19 in Wuhan, China. Clin Infect Dis. 2020.

**Compliance with ethics regulations:** Yes in clinical research.

### FC-250 Prognostic contribution of respiratory mechanics and gas exchange in moderate-to-severe COVID-19 ARDS

#### Nicolas Péron^1^, Bertrand Hermann^1^, Romy Younan^1^, Jean-Loup Augy^1^, Emmanuel Guérot^1^, Ana Novara^1^, Caroline Hauw-Berlemont^1^, Clotilde Bailleul^1^, Nadia Aissaoui^1^, Jean-Luc Diehl^1,2^

##### ^1^Université de Paris, Intensive care unit, AP-HP, Georges Pompidou European Hospital, Paris, France; ^2^Université de Paris, Innovative Therapies in Hemostasis, INSERM, F-75006 Paris, France, 2 Intensive care unit and Biosurgical research lab (Carpentier Foundation), AP-HP, Georges Pompidou European Hospital, Paris, France

**Correspondence:** Nicolas Péron - nicolas.peron12@gmail.com

*Annals of Intensive Care* 2021, **11(Suppl 1):**FC-250

**Rationale:** SARS-CoV-2 acute respiratory distress syndrome (ARDS) pathophysiology and its differences with ARDS of other etiologies remain unclear. A few studies described respiratory mechanics and gas exchange in COVID-19 moderate-to-severe ARDS, mainly in the first 24 h. The aim of our study is to describe the prognostic contribution of respiratory mechanics and gas exchange in the first 48 h after ARDS onset and its evolution at day 7.

**Patients and methods/materials and methods:** Between March 1th and May 30th 2020, we identified all patients with positive SARS-CoV-2 PCR and moderate-to-severe ARDS hospitalized in our ICU within 48 h of ARDS onset and for whom respiratory mechanics data were collected. All patients were ventilated on a GE Carescape R860 with a metabolic module. Respiratory mechanics including dead space fraction (Vd/Vt) and gas exchange parameters were collected in the first 48 h of ARDS onset and at day 7 ± 24 h.

**Results:** Among 103 patients admitted in our unit, 48 patients met the inclusion criteria. At day 7, only 32 patients were analyzed (died before day 7 *n* = 7, alive and transferred in other unit/hospital *n* = 9). In the first 48 h, Survivors had a significantly lower driving pressure than deceased patients (8,5 (8;10,75) vs 10,5 (9;11,75), *p* = 0.042). At day 7, survivors had a significantly lower driving pressure (10(9;13) vs 14(13;18), *p* = 0.027) and a significantly higher respiratory system compliance (42(36;55) vs 31(26–36) *p* = 0.002). Moreover, the evolution of physiological dead space, end-expiratory lung volume and ventilatory ratio between the first 48 h and day 7 was significantly different.

**Conclusion:** In this monocentric retrospective study, higher driving pressure seems to be associated with ICU mortality in the first 48 h and at day 7. During ICU stay, dead space parameters also have opposite trend between survivors and deceased patients.

**Compliance with ethics regulations:** Yes in clinical research.
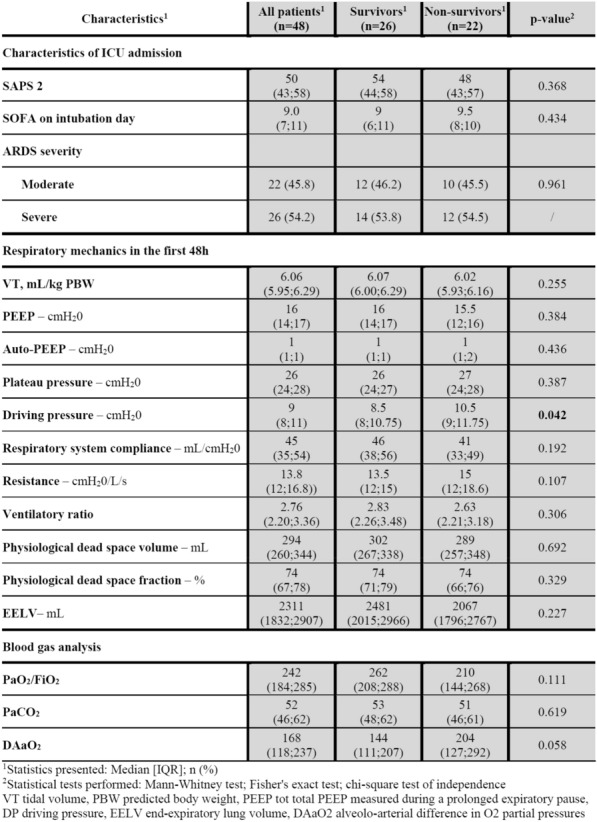


Characteristics of ICU admission, respiratory mechanics and gas exchange in the first 48 h of ARDS onset according to ICU mortality

### FC-251 COVID-19: chest CT findings and their correlations with clinical severity

#### Sarra Zarrouk, Emna Rachdi, Fatma Jarraya, Amira Jamoussi, Samia Ayed, Jalila Ben Khelil

##### hôpital Abderrahmen Mami, Tunis, Tunisia

**Correspondence:** Sarra Zarrouk - sarrabenzarouk@gmail.com

*Annals of Intensive Care* 2021, **11(Suppl 1):**FC-251

**Rationale:** The COVID-19 led to a global health disturbance. Since the lesions due to this infectious disease with respiratory tropism are specific, the chest CT scan was of great help in the diagnosis. The objectives of our study were to describe the CT scan abnormalities in COVID 19 positive patients and to seek a correlation between the extent of lesions and the clinical severity of patients.

**Patients and methods/materials and methods:** This was a prospective and analytical descriptive study carried out in the intensive care unit of Abderahmen Mami Hospital from March 2020 to October 2020. We included all COVID-19 positive patients who have undergone chest CT scan.

**Results:** During study period, 60 COVID-19 patients were admitted, 41 were included. Mean age was 61 years ± 14 (28–90 years) and sex ratio was 1.64. The clinical severity scores IGS II and APACHE II scores were, respectively, 24 ± 12 and 7.64 ± 4. Eighteen patients (40%) had hypertension and 14 (31%) had diabetes. At admission, 20 patients (44%) had an ARDS, light in 6 cases (13%), moderate in 9 (20%) and severe in 5 (11%). For scanning data, frosted glass images were present in 34 patients (75%), condensations in 28 patients (62%), bronchiectases in 16 cases (35%) and one case of crazy paving. Pulmonary embolism was noted in 5 patients (11%) and reticular images in 3 patients (6%). The images were bilateral and peripheral in 100% of cases. The extent of scanning lesions was less than 50% in 11 patients (25%), between 50 and 75% in 16 patients (37%) and greater than 75% in 5 patients (11%). Described lesions were without significant difference between patients with the ARDS and those without as well for frosted glass images (*P* = 0.28), condensation (*p* = 0.22) and bronchiectases (*p* = 0.74)). However, a scanning extent of less than 50% was significantly more common in patients without ARDS (*p* = 0.026). Furthermore, a scanning range greater than 50% was significantly associated with mortality (*p* = 0.026). No correlation was found between the severity of the ARDS and the extent of the lesions.

**Conclusion:** Chest CT scan is a reliable diagnostic tool. The extent of more than 50% of CT lesions was not correlated with clinical severity. A larger sample study is required.

**Compliance with ethics regulations:** Yes in clinical research.

### FC-252 Chest computed tomographic features in coronavirus disease-19 critically ill patients

#### Sondes Mhamdi, Thomas Janson, Ramy Elkolali, Florent Bavozet, Kais Mhamdi

##### Centre Hospitalier Victor Jousselin, Dreux, France

**Correspondence:** Kais Mhamdi - drkaismhamdi@gmail.com

*Annals of Intensive Care* 2021, **11(Suppl 1):**FC-252

**Rationale:** Radiological examinations have a significant role in the diagnosis and management of Coronavirus disease 2019 (COVID-19). Many COVID-19 patients in Intensive Care Unit (ICU) show typical and uncommon chest computed tomography (CT scan) features. We report the spectrum of chest CT imaging findings in COVID-19 infected patients in ICU.

**Patients and methods/materials and methods:** Retrospective descriptive study comprising 82 COVID-19 patients with respiratory symptoms hospitalized from March 2020 to January 2021 in ICU. Data of initial chest CT were retrospectively collected. Prevalence, distribution, extent and type of lung findings were recorded.

**Results:** 82 patients were hospitalized in ICU for COVID-19 infection between March 2020 and January 2021: 48 males (58.6%) and 34 females (41.4%) aged between 34 and 82 years with mean age of 63 years. A total of 80 chest CT scan were evaluated. We observed lung parenchymal abnormalities in 79 cases with incidental chest findings in 5 cases. In only one case, we noted isolated vascular abnormality (bilateral pulmonary embolism).Among the patients with abnormal CT finding, bilateral 68 (85%), multilobar 55 (68.7%) lung involvement with a predominant peripheral 63 (78.75%) and posterior distribution were commonly observed. With regards to the type of opacity, ground glass opacity (GGO) was the dominant abnormality found in all 72 (90%) cases. Pure GGO was observed in 42 (52.5%), GGO with crazy paving pattern was seen in 8 (10%) and GGO mixed with consolidation was noted in 22 (27.5%). Uncommon finding were: pleural effusion 11 (13.75%), pericardial effusion 5 (6.25%). Pulmonary embolism was seen in 5 (6.25%) cases on initial CT. Severity score of parenchymal involvement was at least a grade 2 (25–50% of parenchymal involvement) in 70 (87.5%) cases.

**Conclusion:** COVID-19 critically ill patients tend to manifest typical imaging features on chest initial CT scan. The most common chest imaging finding was bilateral, peripheral and predominantly basal ground glass opacities. Despite of some atypical images, these typical findings can help in the diagnosis and triaging before PCR diagnosis. Lung damage is often severe and severity score of parenchymal involvement is correlated to clinical severity.

**Compliance with ethics regulations:** N/A.

### FC-253 High incidence of thromboembolic events in anticoagulated COVID-19 patients

#### Khaoula Ben Ismail, Boudour Ben Dhia, Najla Ben Slimene, Hedia Ben Ahmed, Moez Kaddour, Takoua Merhabene

##### Hopital regional de Zaghouan, Zaghouan, TUNISIA

**Correspondence:** Khaoula Ben Ismail - khaoula87@hotmail.fr

*Annals of Intensive Care* 2021, **11(Suppl 1):**FC-253

**Rationale:** Coagulopathy is a common abnormality in patients with COVID-19. It may predispose to both venous and arterial thromboembolism due to excessive inflammation, hypoxia and diffuse intravascular coagulation. It has been associated with multiple direct and indirect cardiovascular complications. We aim to evaluate incidence and outcome of cardiovascular complications (CVC) and thromboembolic events (TEE) in anticoagulated COVID-19 patients.

**Patients and methods/materials and methods:** We performed this retrospective cohort study in COVID-19 patients hospitalized in our ICU between 24th March 2020 and 31st January 2021 to assess the incidence and the composite outcome of symptomatic CVC and TEE in all COVID-19 patients admitted to the ICU. Pulmonary embolism was systematically searched in patients with persistent hypoxemia or secondary respiratory deterioration.

**Results:** 126 patients were enrolled. Mean age was 62 ± 12 years [34–92] with a sex ratio 1.5. 47% of patients were over 65 years. Seventy-nine patients (65.3%) were obese (IMC ≥ 30 kg/m^2^) and 30.6% were smokers. Mean SAPS II, Charlson and APACHE II scores were 25.9 ± 8 [6–50]; 2.8 ± 1 [0–7]; 7.7 ± 3 [1–27], respectively. Comorbidities were present in 66.9% of cases; it was mainly cardiovascular (60.3%) and diabetes (35.5%). All patients were treated with therapeutic anticoagulation. Twenty-eight patients developed TEE and/or CVC in our study (incidence of 23%).This group was significantly older (66.9 ± 10 vs 61.3 ± 12.8 years, *P* = 0.038). Thromboembolic events occurred in most cases in COVID-19 patients treated with preventive anticoagulation (76.9%), this complication occured at of day 5 ± 4 of hospitalization. PE was the most frequent thrombotic complication (*n* = 8, 28.6%).It was classified at high risk of mortally in 50% of cases (*n* = 4), intermediate and low risk in 25% (*n* = 2) of cases each one. Two patients have developed acute limb ischemia: the first was in the hand complicating implement of radial arterial line and the other was in the lower limb complicated with rhabdomyolysis and multiorgan failure. Only one patient have presented cerebrovascular accident complicating non-ST elevation acute coronary syndrome On the other hand, COVID-19 infection has been associated with multiple CVC including: acute myocardial injury in 4.9% of cases (ST +*n*=5 and ST −*n* = 1), myocarditis in 0.8% of cases and arrhythmias in 6.6% of cases. Treatment of pulmonary embolism with high risk was thrombolysis with success in 50%. In comparison of total patients, those who developed CVC ou VEE have higher mortality (60.7% in vs 48%, *P* = 0.038)

**Conclusion:** Incidence of CVC in COVID-19 infections in patients who were treated with therapeutic anticoagulation is remarkable. Our findings reinforce the recommendation to strictly apply pharmacological thrombosis curative in all COVID-19 patients admitted to the ICU.

**Compliance with ethics regulations:** Yes in clinical research.

### FC-254 Characteristics and mortality prognostic factors of patients with severe COVID-19 pneumonia admitted in an intensive care unit of a tertiary general hospital

#### Marie Baron^1^, Luis Ensenyat-Martin^1^, François-Xavier Laborne^1^, Karim Chergui^1^, Pierrick Cronier^1^, Sabina Djouhri^1^, Nicolas Maziers^1^, Danielle Reuter^1^, Maria Aroca^1^, Delphine Ceraudo^3^, Pascale Labedade^2^, Mathieu Desmard^1^, Guillaume Chevrel^1^

##### ^1^CH Sud Francilien, Corbeil-Essonnes, France; ^2^CHU Henri Mondor, Créteil, France; ^3^CHU Avicenne, Bobigny, France

**Correspondence:** Marie Baron - marie.baron19@gmail.com

*Annals of Intensive Care* 2021, **11(Suppl 1):**FC-254

**Rationale:** Severe COVID-19 pneumonia is a relatively unknown pathology. Our objective was to contribute to a better knowledge by describing the characteristics and prognostic factors of patients admitted to intensive care unit in our tertiary general hospital during the first and second waves.

**Patients and methods/materials and methods:** We performed a descriptive retrospective single-center study using the register of all patients admitted for severe COVID-19 to our intensive care unit between 6 March 2020 and 28 December 2020. The following parameters were recorded for each patient: demographic characteristics, past medical history, clinical and biological parameters on admission and during hospitalization, ventilation modes, use of extra-corporeal membrane oxygenation, renal replacement therapy, treatments, complications and vital status at day 28. We performed an univariate analysis; the medians of the continuous variables were compared by the Wilcoxon test and the percentages of the categorical variables with their 95% confidence interval were compared by Fisher’s exact test. Due to missing data, and in order to obtain an analysis of the least biased and most precise parameters, we performed a multivariate MICE (multivariate imputation by chained equations) analysis.

**Results:** 212 patients with severe pneumonia were included, after confirmation of COVID-19 by RT-PCR (211 patients), with suggestive symptoms associated or not with a chest CT-scan (194 patients). Median age was 61 years, female/male ratio was 34%/66%, there were 69% of intubated patients. Day 28 mortality was 34% and 36% of deaths happened in a context of withholding or withdrawing of life sustaining treatment. After univariate analysis, significant variables of deceased patients were included in the multivariate analysis with the following results: older age (adjusted OR 1.09, CI [1.03–1.14], *p*-value < 0.01); more prevalent cardiovascular SOFA score ≥ 3 (adjusted OR 3.82, CI [1.28–11.4], *p*-value 0.02) and higher troponin (adjusted OR 3.21, CI [1.02–10.08], *p*-value 0.049). There was no significant difference in mortality for the patients who received corticosteroids (10% of patients on the first wave and 100% on the second wave).

**Conclusion:** Our study revealed several prognostic factors of mortality in patients with severe COVID-19 pneumonia. We continue to complete our register in order to enrich the results of the multivariate analysis.

**Compliance with ethics regulations:** Yes in clinical research.

### FC-255 Pulmonary barotrauma and COVID-19: epidemiological and clinical characteristics and outcomes

#### Feriel Ben Aba, Hamdi Hemden Doghri, Nadia Zarouane, Ines Sdiri, Imen Zaghdoudi, Chiraz Chamakhi, Nabiha Borsali Falfoul

Université de Tunis El Manar, Faculté de Médecine de Tunis. Hôpital Habib Thameur. Service des urgences et de réanimation médicale, Tunis, TUNISIA

##### **Correspondence:** Feriel Ben Aba - benaba.feriel@gmail.com

*Annals of Intensive Care* 2021, **11(Suppl 1):**FC-255

**Rationale:** Pulmonary Barotrauma (PB) have been reported as a complication of COVID-19. It may reveal the SARS-CoV2 infection, or secondarily, it may be related to the mechanical ventilation (MV) in critically ill patients. Our objective was to describe epidemiological and clinical characteristics and outcomes of PB in COVID-19 patients admitted in the intensive care unit (ICU).

**Patients and methods/materials and methods:** Observational descriptive and prospective study including patients admitted in ICU between 09/07/2020 and 12/31/2020. Patient’s data were taken from patient’s medical files.

**Results:** 67 patients infected by the SARS-CoV 2 were included. Sex ratio was 1.57. Age and SAPSII score had as median value, respectively, 64 and 35. 5 patients had history of respiratory illness (2 patients had asthma and 3 had chronic obstructive pulmonary disease (COPD).15 patients (22.4%) had smoking habits. Non-invasive ventilation (NIV) was performed in 47 cases. The number of ventilation sessions per day had a median value of 3| 2.3] with a median duration of 4 [4.12] h per session. The average of the positive end-expiratory pressure PEEP was 8 cm H2O ± 1.16. 35 patients needed an invasive mechanical ventilation (IV). PEEP, the plateau pressure and the driving pressure had a mean value, respectively, of 11.2 CmH2O ± 1.5, 28 CmH2O ± 3.7 and 16 CmH2O ± 4.1. During their hospitalisation, 12 patients presented a PB. It was revealed by a chest X-ray in 6 cases, by the chest computed tomography (CT) scan in 4 cases and by physical exam in 2 cases. It was a subcutaneous emphysema, a pneumomediastinum and a pneumothorax in, respectively, 6, 5 and 1 case. Median duration before the complication occurred was 6.5 days. 7 patients were under IV and 5 were under NIV at the time of diagnosis. A respiratory worsening occurred in 11 cases. 2 patients needed IV and we stopped NIV temporarily in 3 patients. Only one patient was treated with chest drain insertion: It was a bilateral tension pneumothorax complicated by a cardiopulmonary arrest that needed exsufflation and bilateral chest drain. In the Chest CT scan, lung damage superior to 75% of the parenchyma was identified as an independent risk factor of PB (OR: 8.22; 95% CI: 1.73–39.07; *p* = 0.008). PB was significantly associated to an extended duration of MV (*p* = 0.01), an extended duration of stay (*p* = 0.05) and an increased mortality (*p* = 0.01).

**Conclusion:** PB may worsen prognosis in critically ill patients. It extends the MV duration, the length of stay and it increases mortality.

**Compliance with ethics regulations:** Yes in clinical research.

### FC-256 Barotrauma in critically ill Covid-19 patients: prevalence, risk factors and prognosis

#### Sabrina Chaouch, Oussama Jaoued, Hajer Nouira, Meyssem Abdelkarim, Rim Gharbi, Habiba Ben Sik Ali, Mohamed Fekih Hassen, Souheil Artous

##### service de réanimation médicale hôpital Taher Sfar, Mahdia, TUNISIA

**Correspondence:** Oussama Jaoued - oussamajaoued@gmail.com

*Annals of Intensive Care* 2021, **11(Suppl 1):**FC-256

**Rationale:** Barotraumas are a known complication of mechanical ventilation. In SARS-CoV-2 infection, some studies showed that 10 to 20% of patients developed a pneumothorax, probably caused by a rupture of the alveolar wall. The aim of the study is to determine the clinical characteristics, risk factors and outcomes of patients with COVID-19 pneumonia complicated by a barotrauma.

**Patients and methods/materials and methods:** This is a retrospective study. We included all patients admitted in the intensive care unit with a confirmed COVID-19 pneumonia. The diagnosis of COVID-19 was confirmed with reverse transcriptase PCR. The diagnosis of acute respiratory distress syndrome was based on Berlin criteria. All patients were ventilated with a tidal volume equal to 6 ml/kg and a driving pressure less than 14 cmH2O. We recorded: comorbidities, clinical parameters, laboratory, chest X-ray and CT scan findings, SAPSII score, driving pressure and PEEP.

**Results:** A total of 114 patients with a mean age of 62 ± 12 years and with a mean SAPSII score 28 ± 12 was included. At admission, 24(21%) patients were on invasive mechanical ventilation, 6 patients were receiving non-invasive mechanical ventilation. The prevalence of barotrauma was 21% (pneumothorax 16 (14%) cases, subcutaneous emphysema 17(15%) patients and pneumomediastinum in 3 patients). The traditional tracheostomy tube was performed in 16 patients. The mortality rate was 45%. The driving pressure and the plateau airway pressure were similar between patients with and without barotrauma. In multivariate analysis, the predictor factors of barotrauma were SAPSII score (OR: 1.2, 95%IC [1.02–1.58] *p* = 0.01) and the presence of diabetes (OR = 2.1, IC95% [1.20–2.32, *p* = 0.022). In patients with barotrauma, the mortality rate was 80% (19 patients). In multivariate analysis, the only predictive factor of mortality was barotrauma (RR = 6.9; 95%IC [1.9–18.9], *p* = 0.002).

**Conclusion:** Patients with a COVID-19 pneumonia developed barotrauma in 1/5 cases. Patients with barotrauma had a high mortality rate. It’s necessary to pay attention to diabetic patients with an elevated SAPSII score.

**Compliance with ethics regulations:** Yes in clinical research

### FC-257 Extracorporeal membrane oxygenation (ECMO) in refractory acute respiratory distress syndrome (ARDS) due to COVID-19: an observational study

#### Camille Le Breton, Sébastien Besset, Santiago Freita-Ramos, Didier Dreyfuss, Jean-Damien Ricard, Damien Roux

##### ^1^Assistance Publique Hopitaux de Paris - hôpital Louis Mourier, Colombes, France

**Correspondence:** Damien Roux - damien.roux@aphp.fr

*Annals of Intensive Care* 2021, **11(Suppl 1):**FC-257

**Rationale:** Several studies suggest a survival benefit of ECMO in ARDS. However, its place remains uncertain for severe COVID-19-related ARDS, with a very high mortality observed in first studies (up to 80%). Our objective was to analyze the evolution of patients who presented with refractory ARDS requiring venous–venous ECMO (VV ECMO) during the COVID-19 pandemic.

**Patients and methods/materials and methods:** Single-center French retrospective study. All patients admitted for ARDS COVID-19 and treated with ECMO VV were analyzed. The ethics committee of Paris University Hospitals approved this study (CEERB Paris Nord. IRB 00006477).

**Results:** Between March and April 2020, 83 patients were hospitalized for SARS-CoV-2 pneumonia. Thirteen patients required VV ECMO for severe refractory ARDS despite prone position, use of neuro-muscular blockers, and inhaled nitric oxide. At the time of ECMO implantation, the median duration of mechanical ventilation (MV) was 6 days and the median PaO2/FiO2 ratio was 59 mmHg. The median tidal volume was 5.25 ml/kg with a median positive end-expiratory pressure (PEEP) of 12 cmH2O. The median plateau pressure was 32 cmH2O with a driving pressure of 20 cmH20. All patients were hypercapnic (median PaCO2 65 mmHg). Seven major adverse events occurred in 4 patients, including 3 major hemorrhages requiring massive transfusion, 2 cannula infections with Enterococcus faecalis bacteremia, and 2 circuit changes (device thrombosis or circuit-related thrombocytopenia). All 13 patients were weaned from ECMO after a median of 13 days. Two patients died under MV: a 41-year-old man, Jehovah’s Witness (information obtained after implantation), with deglobulization < 5 g/dL (bleeding and hemolysis under ECMO) without any possibility of transfusion leading to early explantation; the second patient died of major right ventricular failure with probable pulmonary embolism 7 days after ECMO explant. After 3 months, the 11 other patients were discharged from the ICU with a median length of stay of 34 days and a median duration of MV of 29 days.

**Conclusion:** Despite the retrospective nature of the study and the small number of patients, these results are very encouraging and contrast with previous studies. The rigorous selection of patients, in consultation with a reference center, could explain this difference. ECMO should be considered as a lifesaving therapy in selected patients who develop severe refractory COVID-19-related ARDS despite optimal conventional treatment.

**Reference**

1. Combes A, Hajage D, Capellier G, Demoule A, Lavoué S, Guervilly C, et al. Extracorporeal Membrane Oxygenation for Severe Acute Respiratory Distress Syndrome. N Engl J Med 2018;378:1965–75. 10.1056/NEJMoa1800385.
2. Ramanathan K, Antognini D, Combes A, Paden M, Zakhary B, Ogino M, et al. Planning and provision of ECMO services for severe ARDS during the COVID-19 pandemic and other outbreaks of emerging infectious diseases. Lancet Respir Med 2020;8:518–26.

**Compliance with ethics regulations:** Yes in clinical research.

### FC-258 Association between body mass index and pneumonia outcomes in critically ill patients with coronavirus disease-19: an international multicenter retrospective cohort study

#### Mikael Chetboun^1^, Arthur Simonnet^8^, V. Raverdy^1^, Julien Labreuche^1^, Florent Wallet^9^, Cyrielle Caussy^9^, M. Antonelli^10^, A. Artigas^11^, G. Goma^11^, F. Meziani^12^, Julie Helms^12^, Eleftherios Mylonakis^13^, Mitchell Levy^13^, N. Latronico^14^, Simone Piva^14^, Charles Cerf^7^, M. Neuville^7^, Kada Klouche^4^, Romaric Larcher^4^, Fabienne Tamion^19^, Emilie Occhiali^19^, Morgane Snacken^15^, Jean-Charles Preiser^15^, Loay Kontar^6^, Antoine Riviere^21^, Stein Silva^2^, Benjamine Sarton^2^, Raphael Krouchi^22^, Victoria Dubar^22^, Leonidas Palaiodimos^20^, Dimitrios Karamanis^16^, Juliette Perche^8^, Erwan L’her^5^, Luca Busetto^17^, Dror Dicker^18^, Shaul Lev^18^, Alain Duhamel^1^, Francois Pattou^1^, Mercedes Jourdain^1^

##### ^1^CHU, Lille, France; ^2^CHU, Toulouse, France; ^3^CHU, Strasbourg, France; ^4^CHU, Montpellier, France; ^5^CHU, Brest, France; ^6^CHU, Amiens, France; ^7^Hopital Foch, Suresnes, France; ^8^CH, Roubaix, France; ^9^CHU, Lyon, France; ^10^Fondazione Policlinico Universitario A, Roma, ITLAY; ^11^Corporacio Sanitaria Universitaria Parc Tauli, Sabadell, SPAIN; ^12^Hopitaux Universitaires de Strasbourg, Strasbourg, France; ^13^Warren Alpert Medical School of Brown University, Providence, USA; ^14^Spedali Civili University Hospital, Brescia, Italy; ^15^Erasme Hospital, Université Libre de Bruxelles, Brussels, Belgium; ^16^University of Piraeus, Attica, GREECE; ^17^University of Padova, Padova, Italy; ^18^Hasharon Hospital-Rabin Medical Center, Tel Aviv, ISRAEL; ^19^CHU, Rouen, France; ^20^Albert Einstein College of 106 Medicine, New York, USA; ^21^CH, Abbeville, France; ^22^CH, Dunkerque, France

**Correspondence:** Mercedes Jourdain - mercedes.jourdain@univ-lille.fr

*Annals of Intensive Care* 2021, **11(Suppl 1):**FC-258

**Rationale:** Identifying risk factors associated with Coronavirus disease-2019 (COVID-19) severity is essential. An overall relation between body mass index (BMI) and pneumonia severity in patients with COVID-19 was suggested. The objectives of the present study are to examine the relation between BMI and the need for invasive mechanical ventilation (IMV) in critically ill patients with COVID-19, across multiple countries, and the impact of age and sex on this relationship.

**Patients and methods/materials and methods:** A retrospective cohort study combining individual COVID-19 patient data from intensive care units (ICUs) in 21 centers in France (13), Italy (3), USA (2), Israel (1), Belgium (1), and Spain (1), between February 19th and May 19 th, 2020. The exposure of interest was BMI (weight in kilograms divided by the square of height in meters) measured at the time of admission in ICU. The primary outcome was the need for IMV. The secondary outcome was 28-day all-cause mortality rate. The covariates were age, sex, and pre-specified metabolic risk factors, including diabetes, hypertension, dyslipidemia, and current smoking.

**Results:** 1,461 patients with COVID-19 were included (median age 64 years (Interquartile range (IQR), 40.9–72.0); 73.2% males; median BMI 28.1 kg/m^2^ (IQR, 25.4–32.3); 1080 patients (73.9%) required IMV and the 28-day mortality rate was 36.1% (95% confidence intervals (CI), 33.0–39.5). Adjusted mixed logistic regression model showed a significant relation between BMI and the need for IMV: odds ratio (OR) 1.27 (95% CI, 1.12–1.45) per 5 kg/m^2^ in the whole cohort, and 1.65 (95%CI, 0.97–2.79) per 5 kg/m^2^ in females under 50 years. Adjusted Cox proportional hazards regression model with random center effect showed a significant association between BMI and mortality, which was only increased in obesity class III (≥ 40 kg/m^2^) (hazard ratio 1.68 (95% CI 1.06–2.64).

**Conclusion:** We observed a linear association between BMI and the need for IMV, more pronounced in younger females, and a non-linear association between BMI and mortality risk in critically ill patients with COVID-19. The relation of higher BMI with severe COVID-19 disease should foster the development of more effective interventions to prevent and treat obesity worldwide. TRIAL REGISTRATION: ClinicalTrials.gov Identifier: NCT04391738 224

**Compliance with ethics regulations:** Yes in clinical research.

